# Connexins during 500 Million Years—From Cyclostomes to Mammals

**DOI:** 10.3390/ijms22041584

**Published:** 2021-02-04

**Authors:** Svein-Ole Mikalsen, Sunnvør í Kongsstovu, Marni Tausen

**Affiliations:** Faculty of Science and Technology, University of Faroe Islands, FO-100 Tórshavn, Faroe Islands; sunnvork@setur.fo (S.í.K.); marni.tausen@gmail.com (M.T.)

**Keywords:** cartilaginous fishes, connexins, evolution, gap junctions, lamprey, phylogeny, vertebrates

## Abstract

It was previously shown that the connexin gene family had relatively similar subfamily structures in several vertebrate groups. Still, many details were left unclear. There are essentially no data between tunicates, which have connexins that cannot be divided into the classic subfamilies, and teleosts, where the subfamilies are easily recognized. There are also relatively few data for the groups that diverged between the teleosts and mammals. As many of the previously analyzed genomes have been improved, and many more genomes are available, we reanalyzed the connexin gene family and included species from all major vertebrate groups. The major results can be summarized as follows: (i) The same connexin subfamily structures are found in all Gnathostomata (jawed vertebrates), with some variations due to genome duplications, gene duplications and gene losses. (ii) In contrast to previous findings, birds do not have a lower number of connexins than other tetrapods. (iii) The cyclostomes (lampreys and hagfishes) possess genes in the alpha, beta, gamma and delta subfamilies, but only some of the genes show a phylogenetic affinity to specific genes in jawed vertebrates. Thus, two major evolutionary transformations have occurred in this gene family, from tunicates to cyclostomes and from cyclostomes to jawed vertebrates.

## 1. Introduction

Connexins are transmembrane proteins that aggregate into hexameric structures called connexons, and connexons from adjacent cells further line up with each other to form a channel that can connect the cytoplasms in the two neighboring cells (reviewed by Harris [1]). The channels further aggregate into smaller or larger plaques, called gap junctions, named after the small gap between the neighboring cell membranes, which contrasts with the tight apposition of the membranes in the tight junctions. These channels are regulated by many mechanisms, including numerous cell-signaling systems [2], which may affect the connexins at every stage in their life cycle, from gene expression via the gating of the channels (opening and closing behaviors) to their degradation [1]. The physiological importance of connexins is indicated by the links with several genetic diseases [3] but, also, by being implicated in major human diseases like cancer and heart and coronary diseases [4,5,6].

In humans, the connexin family consists of 21 potentially functional members, including the little studied *GJE1* (*connexin23*) [7], and some additional pseudogenes [8,9]. The Human and Mouse Gene Nomenclature Committees have decided that the genes should be named according to their subfamilies [10,11]: gap junction protein gene subfamily alpha (*GJA*), beta (*GJB*), gamma (*GJC*), delta (*GJD*) and epsilon (*GJE*), followed by a number indicating (more or less) their chronology of detection. The proteins are generally named “connexin”, followed by a number constituting the protein’s predicted molecular weight in kDa, which is independent of the subfamily. Formally, only humans and rodents have received a Greek nomenclature, but as stated by the Zebrafish Nomenclature Conventions: “… genes should be named after the mammalian ortholog whenever possible” [11]. Here, we understand orthologs as genes originating from a single ancestral gene in the last common ancestor of the compared genomes [12,13], i.e., it is the same gene in different species. The remaining genes in the gene family are called paralogs [12,13]. For convenience, here, we refer to the genes (and their corresponding mRNAs) as “connexin” genes, but when referring to specific genes, we will use their abbreviated formal names (or suggested amended names, where these are formally more correct). We also note that a novel connexin nomenclature was recently proposed for eutherian mammals [14], with *CXN* followed by a letter in alphabetical order (*CXNA* to *CXNU*). However, other vertebrates than placental mammals were not considered in this suggestion [14].

In the early years of connexin research, it was difficult to find a consistent interspecies pattern of family relationships for all the connexin genes, and it was suggested that several connexins were species-specific and group-specific [15]. However, a study of connexin genes across mammals indicated that some of the presumed species–specific connexins could in fact be found in many species [8]. Subsequent studies were extended into birds (chicken), amphibians (*Xenopus tropicalis*) and teleost fishes [16,17,18], and it became clear that, although several gene losses or gene duplications occurred during evolution, the basic structure of the connexin family was well-established in teleosts [17]. Between the divergence of teleosts 250–350 million years ago [19,20,21] until the appearance of placental mammals around 100 million years ago [22], several gene losses and gene duplications occurred in the connexin family. Furthermore, the previous results suggested that birds had a smaller connexin family than other vertebrates, with only 16 members [17], which seemed to follow the pattern of supposed gene loss in birds [23,24,25].

Although the previous analyses indicated a relatively clear gene family history among the vertebrates from teleosts to mammals, there were some gaps, as there were no available genomes from certain vertebrate groups (like several reptile groups, lobe-finned fishes and non-teleost ray-finned fishes), or the number of genomes was limited to one animal per group (like birds and amphibians) [16,17]. Even more importantly, the early evolution of the connexin family is unclear. The tunicates are the most primitive organisms where connexins genes have been found [17,26,27], but the tunicate connexins did not display any evident subfamily structure similar to that found in vertebrates, and they did not have any strong phylogenetic affinity to any of the vertebrate genes [17]. The only information available between the unorganized connexin family in tunicates and the highly structured connexin family with a full-blown subfamily structure in teleosts (the “standard” bony fishes) is a single glimpse of a connexin from a cartilaginous fish: O’Brien et al. [28] cloned *Cx35* from little skate (*Raja erinacea;* now *Leucoraja erinacea*), a connexin that had high resemblance to the subsequently identified mammalian *Cx36* (*GJD2*) [29]. Otherwise, there seems to be a lack of studies in cartilaginous fishes to this day, and we do not know whether this group of fishes has a well-expanded and structured connexin family or not.

During the last decade, many genomes from different vertebrate groups have become available. Despite that many of the assemblies have been subject to a high level of automatic annotation of genes, i.e., the predicted genes are present in the major databases, most of the predicted genes have not been manually curated or analyzed. The many new genome assemblies make it possible to get a clearer picture of both the early evolution of this gene family and the changes that subsequently occurred. Here, we extend the previous analyses [17,18]. First, we increased the number of representatives in amphibians and birds. Second, and much more importantly, we added several vertebrate groups that have not been analyzed before, including several Reptilia groups (Crocodylia [30,31]; Testudines (turtles) [32]; Squamata (lizards and snakes) [33]); the lobe-finned fish *Latimeria* [34]; the non-teleost ray-finned fish groups Holostei (with spotted gar as the representative [35]) and Chondrostei (consisting of the paraphyletic groups Acipenseriformes with sterlet sturgeon [36] and Polypteriformes with reedfish as representatives, respectively); Chondrichthyes (with whale shark [37], little skate [38,39] and elephant shark [40] as the main representatives) and, finally, Cyclostomata (with three species of lamprey [41,42,43] and one species of hagfish as the representatives). These analyses of the connexin gene family constitute the widest collection of vertebrate groups performed to date.

## 2. Results

**Figure 1:**
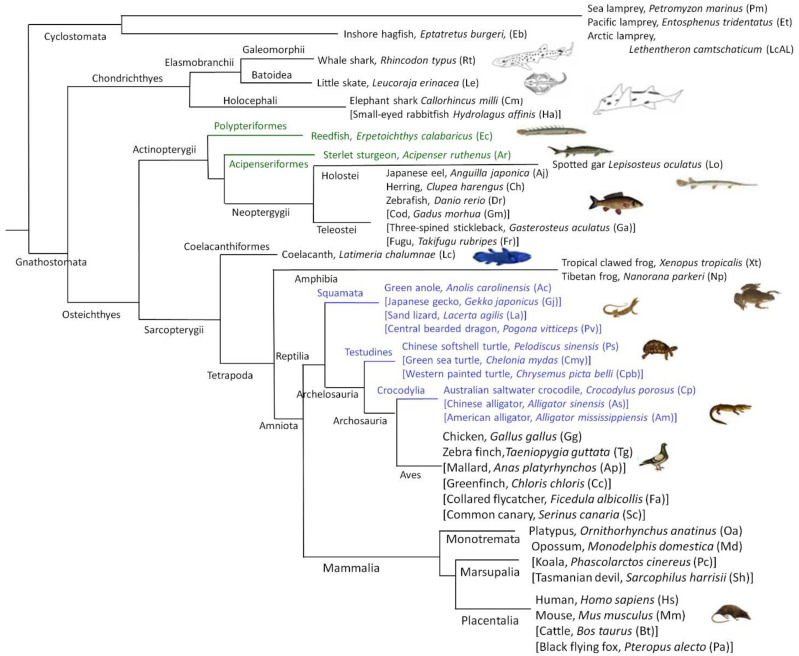
Simplified overview of the relationships between the involved species. The abbreviations given in parentheses (rightmost) refer to the species abbreviations used in the present work. The groups outlined in green and blue font belong to the paraphyletic groups Chondrostei and Reptilia, respectively. Note that, formally, Aves also belongs to the Reptilia group. The tree itself is loosely based on https://en.wikipedia.org/wiki/Chordate, https://en.wikipedia.org/wiki/Vertebrate and https://en.wikipedia.org/wiki/Tetrapod, from which many of the animal illustrations are taken. The main species from which we tried to collect all their connexins sequences are indicated without square brackets, while the supplementary sequences were collected from the species in square brackets.

The simplified chart of the involved species is shown in Figure 1. The mammalian genomes are considered as the starting points of the annotation and naming of genes and, by extension, also the gene family structures and substructures. We therefore used the mammalian connexin genes and family structures as the comparative basis for this investigation. For the nonmammalian connexin groups, we mainly followed our recently suggested nomenclature [9], which is consistent with the established mammalian nomenclature, and any deviation will be specified.

All sequences with accession numbers (where appropriate) or scaffold accession numbers and positions (where appropriate) are found in Supplementary Figures S1–S43. The conserved domains, which are used in the phylogenetic analyses, are marked on the sequences, and (where appropriate) further comments are also given. As a general remark, we would like to point out that, in some species, there are certain connexin-like genes that are not included in the present phylogenetic analyses, and some of them might constitute another subfamily (for example, Ensembl zebrafish ENSDARG00000086255; Rachel Lukowicz and Adam Miller, personal communication).

A typical compressed phylogenetic tree for the connexin gene family in vertebrates (excluding the cyclostomes) is shown in Figure 2. The expanded branches are found in Supplementary Figures S44–S68. The *GJE1* orthogroup was excluded from this tree, as its quite deviating sequence caused long-branch attraction [44,45] and ripped the *GJC3* group apart and, further, caused other positional rearrangements in the tree. A tree with the *GJE1* group can be seen in Supplementary Figure S69, and the expanded *GJE1* branch is seen in Supplementary Figure S70.

Several tree-building methods and substitution matrices were used, using both amino acid sequences and nucleotide sequences (with only positions 1 and 2 in the codons). The results are summarized in Supplementary Table S1. The phylogenetic analyses strongly indicated that the vertebrate groups that have not been previously investigated (crocodiles, turtles, lizards, *Latimeria*, the two Chondrostei groups, Holostei and cartilaginous fishes), or previously only been represented by a single species (birds and amphibians), did follow the patterns previously established [8,9,17,18] for the connexin subfamilies. A vertebrate group or a single species within a vertebrate group could lack a gene found in closely related species or groups, or, vice versa, gene duplications could have occurred within a specific species or vertebrate group.

The different methods and models generally gave quite similar results. Some orthogroups were always united: *GJA1*, *gja2*, *GJA3*, *GJA5*, *GJA8*, *GJA9*, *gja14*, *GJB1*, *GJB3*, *GJB7*, *gjb9*, *gjb11*, *GJC2*, *GJD3*, *GJD4*, *GJD5*, *gjd6* and *GJE1* (upper case letters indicate the inclusion of mammalian sequences in the orthogroup). Most of these orthogroups had representatives from (nearly) all vertebrate groups, but some of them had more restricted representation from only some of the vertebrate groups, and *gjb11* only contained sequences from cartilaginous fishes.

For a few orthogroups, some of the models made specific vertebrate groups (like teleosts) split off from the remaining orthogroup (*GJA4*, *GJA10*, *gja11*, *GJB4*, *GJB5*, *gjb8*, *gjb10*, *GJC1*, *GJC3* and *gjc4*), but overall, we are still convinced that the sequences do belong to the same orthogroup, because (i) other the models did not split these orthogroups and (ii) synteny considerations support orthology (data not shown). Therefore, the orthology makes biological sense.

In some models, especially combined with a gamma value of 0.7–0.9 (as indicated by the model selection using all vertebrate sequences), a few of the orthogroups became highly fragmented. This especially concerned *GJD1*, but setting a gamma value of 1 (or higher) caused the sequences from the different vertebrate groups to collect into more united orthogroups. Additionally, there were single sequences that sometimes split off from their normal orthogroup and were located as singletons. However, the lack of consistent behavior (i.e., the sequence did not always split off; sometimes, other sequences split off, and the split-off sequence is not always located in the same way) makes us consider these sequences as members of the orthogroup that they are usually located within (or adjacent to).

Below, we will only point out some of the most interesting features, starting with genome duplications and ohnologies.

### 2.1. On Genome Duplications and Ohnology

The early teleosts went through genome duplication around 250 to 350 million years ago [19,20,21]. Signals from that genome duplication are found as duplicated genes on different chromosomes, called ohnologs [46,47]. Several teleost connexins show ohnology [9,17]. The exact number of detected teleost ohnologous pairs varies with the species but are often in the area of 10–14 pairs [9]. The average teleost ohnolog identities (as measured by the full-length nucleotide sequences) were around 82% in eels (range 71.4–90.7%) (Supplementary Table S2), 76% in herring (range 69.1–82.8%) (Supplementary Table S3) and 72% in zebrafish (range 67.3–82.0%) (Supplementary Table S4).

According to several recent papers [36,48], sturgeons have gone through an independent genome duplication, and in certain species of sturgeons, there could even have been several genome duplications [49]. Consistent with that notion, we found duplicated sequences for nearly all sterlet sturgeon connexins, with 23 ohnolog pairs (Supplementary Table S5) (note that there are more than two genes in some “pairs”, but we do not know whether this is a genetic reality or caused by erroneous genome assembly). The average ohnolog identity in the sterlet was nearly 97% at the nucleotide level (range 88.4–99.2%; Supplementary Table S5). This strongly supported fully independent genome duplications in teleosts and in sturgeons, but the estimates of the timing of the sturgeon genome duplication vary widely, from 21 [48] to 180 [36] million years ago. Due to the independent genome duplications, we use upper case “A/B” for the sturgeon ohnologs and lower case “a/b” for the teleost ohnologs. For simplicity, we only used the sterlet sturgeon ohnolog “A” in the phylogenetic analyses (with a single exception for *gje1*, where the A ohnolog might be a pseudogene), but all sequences and their genomic locations are found in Supplementary Figure S28 and Supplementary Table S5. Please see Discussion for further details about naming the “a” and “b” ohnologs.

### 2.2. Chondrichthyes, Actinopterygii (Chondrostei, Holostei and Teleostei) and Sarcopterygii

The ray-finned fishes, Actinopterygii, consist of four subgroups: (i) Teleostei, which by far constitutes the most species-rich group within bony fishes and that went through a genome duplication before the species radiation [19,20]. (ii) Holostei, which diverged just before the teleost genome duplication. There are also two earlier diverging paraphyletic subgroups of Chondrostei, consisting of (iii) Polypteriformes and (iv) Acipenseriformes (see Figure 1 for overview).

Based on previous analyses including only mammalian and teleost sequences, we recently suggested that the teleost-specific genes best known as *cx32.2*, *cx32.3*, *cx34.5*, *cx32.7*, *cx28.1, cx28.9*, etc. (using zebrafish as the example), which all are closely related and located in the vicinity of each other on the same chromosome, should be classified as *gja11* (*cx34.5* and *cx32.7*), *gja12* (*cx28.1* and *cx28.9*) and *gja13* (*cx32.2* and *cx32.3*) [9]. In the present work, we point out that *gja11* is also found in non-teleost fishes (including cartilaginous fishes and in the lobe-finned fish *Latimeria*; the only exception being the polypteriformian reedfish). *Gja11* is thus common to nearly all groups of cartilaginous and bony fishes. On the other hand, *gja12* and *gja13* were only found in teleosts. With the close positional link between *gja11*, *gja12* and *gja13* on a single teleost chromosome and their sequence similarities, it is very likely that *gja12* and *gja13* were generated by two gene duplications (and, in some species, like zebrafish, more than two gene duplications) from *gja11* after the teleost genome duplication. As the *gja11*/*gja12*/*gja13* names already are in use for new zebrafish entries in GenBank (since November 2020), here, we will stick to these names, although we are presently inclined to suggest that *gja12* and *gja13* should be fused into *gja12* and, hence, be named *gja12.1*/*.2*/*.3*/*.4*, etc. In zebrafish, with its additional gene duplication(s), the suggested names would be *gja12.1.1*, *gja12.1.2*, *gja12.2.1*, *gja12.2.2*, etc. Since *gja13*, for the time being, is occupied, a new *gja5*-related connexin that has (as yet) only been found in Holostei, Acipenseriformes and *Latimeria* will be called *gja14*.

Chondrichthyes have a new gene in the beta subfamily, which we suggest naming *gjb11*. This gene is located phylogenetically at the very base of the beta subfamily, and we do not know whether this gene has evolved into one or more of the previously recognized *gjb* genes that are found in Actinopterygii or higher vertebrates. Considering that chondrichthyans had genes in both main arms of the *gjb* part of the phylogenetic tree (i.e., *GJB1* and *gjb8*/*GJB2*/*GJB6* in the one arm and *GJB7* and *GJB3*/*gjb9*/*gjb10*/*GJB4*/*GJB5* in the other arm), we think it is most likely that *gjb11* either was lost before the actinopterygian divergence or that the gene was generated in the line leading to chondrichthyans.

Chondrichthyes had three sets of *gjb3*-related genes closely positioned on the same chromosome (Supplementary Table S6), where two of the sets were phylogenetically always located together with *gjb3*, while the third set was variably located together with *gjb3* or towards *gjb10*. Note that *gjb3*, *gjb9* and *gjb10* are chromosomally linked in all investigated teleosts (chromosome 17 in zebrafish) and, also, in other vertebrate groups. These genes are further discussed in Section 2.6, in addition to *gjb8*/*GJB2*/*GJB6*.

Otherwise, in addition to *gja12* and *gja13* mentioned above, the following connexin genes were specific for Actinopterygii (ray-finned fishes):

- *gjb9* (variably called *gjb4like*, *cx28.6* or *cx30.9*): a gene generally located below the *GJB3*, *GJB4* and *GJB5* orthogroups in the phylogenetic trees, but note the comment that we have on *gjb9* in Section 2.6 below.

- *gjd6* (often called *gjd2like* or *cx36.7*): a gene diverging from the branch leading to *GJD1* and *GJD2.* Note that there appeared to be a *gjd6* ortholog in the cyclostomes (see Section 2.5 for more results from cyclostomes).

### 2.3. Nonmammalian Tetrapods: Amphibia and the Greater Group of Reptilia

The nonmammalian tetrapods (except crocodiles) and fishes possessed *gja2*, a *GJA3*-related gene. In the alpha subfamily, it was reconfirmed [17] that *gja4* went through a gene duplication in the amphibian lineage and generated the classic (*Xenopus*) *cx38* and *cx41*, which, thereby, could be called *gja4.1* and *gja4.2*. In all investigated Amphibia with genome assemblies having scaffolds of reasonable lengths, the two genes were located 10–100 kbp apart (for example, in *Rana temporaria*, *Pyxicephalus adspersus* and *Bufo gargarizans*).

Among the tetrapods, only the amphibian *gjb2/6*-related sequences were located together with the fish *gjb8* sequences. All the other tetrapods had *gjb2*- and *gjb6*-related sequences that were located together with the mammalian *GJB2* and *GJB6* sequences or very close to them. These genes are further discussed in Section 2.6.

In our previous analyses, the chicken genome was found to possess only 16 connexins [16,17]. Birds are often considered to have “streamlined” their genomes and, therefore, showed a considerable fraction of the gene losses compared with the other vertebrate groups [23,24,25]. The low number of connexins in the chicken genome was seemingly consistent with this notion. However, the chicken genome has now become more complete, and several other birds had their genomes sequenced, so the notion of a low number of genes may have to be revised [50]. As predicted [16], we show here that the number of connexins in birds is at the same level as for other tetrapods. The major difference within the four subgroups of Reptilia is the lack of *gja2* in Crocodylia. Reptilia (including birds) was the only vertebrate group to completely lack *gjd5*.

### 2.4. Mammalia

It is well-known that some mammals lack certain connexin sequences and that certain connexins have gone through gene duplications in certain mammalian lineages [8]. The major unexpected observation in this work was the apparent lack of *GJD2* in the platypus, while it seemingly had two *GJD1* sequences placed on different chromosomes (5 and 13). As of today, the platypus is the only monotreme with a sequenced genome. It is therefore uncertain whether the peculiarities seen in the platypus are features of the Monotremata group or specific to the platypus. They could also be due to assembly errors or incomplete genome coverage.

### 2.5. Cyclostomata: Lampreys and Hagfish

The present evidence suggests that hagfish and lampreys are monophyletic, i.e., they evolved from a single ancestor that diverged from the other (to be) vertebrates around 500 million years ago [51,52]. Some 70–100 million years later, the lampreys and hagfishes diverged from each other [51]. We will describe the results for lampreys and hagfish separately, as we consider the lamprey data more solid, having genome assemblies from three lamprey species available, while there is only one hagfish genome assembly available.

Lampreys did possess multiple connexin genes in the alpha, beta, gamma and delta subfamilies, but the gene family was evidently different from the higher vertebrates (Figure 3). In some cases, the lamprey sequences were phylogenetically located close to one of the orthologous groups from the other vertebrates. When the amino acid identities were calculated (using only the conserved domains in MUSCLE [53] or Clustal Omega [54] alignments), there were clearly higher identities to the co-locating chondrichthyan sequences than to the neighboring chondrichthyan sequences. In these cases, we suggest that the lamprey sequences are named according to the closest/most identical connexin orthogroup(s). In other cases, the phylogenetic relationship seemed less obvious, indicated both by their positions in the tree and by identity calculations. In these cases, we tentatively suggest naming the sequences by the unspecific term “gen” (which could either mean “general” or “gene”) followed by a number (example: *gja-gen1*). Additionally, we will use the “.1”/”.2” nomenclature for genes that are phylogenetically in the same clade and can be shown to be located closely on the same scaffold or chromosome in at least one of the lamprey species and, thus, probably have been generated by gene duplication. We will, in the present section, use the “a”/”b” nomenclature for genes that are phylogenetically in the same clade but are located on different chromosomes or different (but still long) scaffolds, despite not having any evidence that these genes were in fact generated by genome duplication. In principle, such genes could have been generated by retrotransposition or by tandem duplication followed by chromosomal rearrangements.

Thus, in the alpha subfamily, lampreys possessed four sets of connexin genes: *gja3* (although the precise location in the trees was not always as a first-degree neighbor of the *GJA3* orthogroup), *gja8*, *gja9/10* and *gja-gen1.1/gen1.2/gen1.3*. The three former sets of genes contained one sequence from each of the three lamprey species, while the fourth, *gja-gen1*, had three sequences from the same chromosome in sea lamprey and one sequence from Pacific lamprey.

The lamprey beta subfamily possessed two sets of connexin genes. The first was *gjb-gen1*, which consisted of three genes in sea lamprey (all on the same chromosome) and two genes in Pacific lamprey and one in Arctic lamprey. Phylogenetically, these sequences are located below *GJB3*/*4*/*5*, *GJB7, gjb9* and *gjb10*. The second beta set was located phylogenetically between *GJB1* and *gjb8*; we suggest calling this set *gjb1/8*. *Gjb1/8* consisted of three genes in sea lamprey (two on the same chromosome and the third unplaced) and two genes from each of the Pacific and Arctic lamprey (on a single chromosome/scaffold in both species).

In the delta subfamily, four lamprey gene sets were found, all of them with one representative from each of the three lamprey species. One set contained two genes from each species and was located between *GJD1* and *GJD2* and with approximately equal amino acid identities to both chondrichthyan *gjd1* and *gjd2* (around 92% to 93% identities for the conserved sequences used in the phylogenetic analyses). As the two lamprey genes were on different chromosomes/scaffolds, we called them *gjd1/2a* and *gjd1/2b*. The second set was located just below *GJD1* and *GJD2*, but the identities belonging to *GJD1* and *GJD2* were evidently lower (around 83–85%). This set of genes was on a different chromosome than those of *gjd1/2a* and *gjd1/2b*, and we called it *gjd-gen1*.

The third delta subfamily set was located in the *GJD3* branch of the tree, with two genes from each of the lamprey species. *GJD3* is one of the most variable connexin genes, and this colocation could have been caused by long-branch attraction. However, this set of lamprey genes was more identical to *GJD3* than to any of the other genes in the delta subfamily. Additionally, when all other *GJD3* sequences were removed, leaving only the lamprey sequences, they essentially positioned themselves in the same way in the tree. Thus, long-branch attraction was excluded. Chromosomally, the two lamprey *gjd3* genes were located close to each other in the sea lamprey and Pacific lamprey chromosomes (Arctic lamprey had too-short scaffolds to tell) and were, thus, likely generated by a gene duplication. Consequently, we suggest calling them *gjd3.1* and *gjd3.2*. The fourth set of lamprey genes in the delta subfamily was located in the *gjd6* branch and was therefore called *gjd6*.

The lamprey genes located in the gamma subfamily were not evidently more similar to any particular chondrichthyan gamma subfamily members. We suggest that the genes that are phylogenetically located between *GJC1* and *GJC2* should be called *gjc-gen1*, while we suggest calling the remaining genes that aggregate in a loose cluster *gjc-gen2*–*gjc-gen5*. Those genes where the chromosomal position could be settled in a lamprey species were indeed located on different chromosomes. Many of the *gjc-gen2*–*gjc-gen5* genes were unplaced in the chromosomal assemblies, and as some of them showed duplicates on different scaffolds, we will not disregard the possibility of misassembly—in particular, in sea lampreys (*Petromyzon marinus*).

Hagfish connexin genes were grouped closely together with lamprey genes, and they were distributed into all the four main subfamilies: alpha, beta, gamma and delta. However, some of the lamprey gene groups were left without hagfish partner genes, but we think that a partial explanation is a less complete genome assembly in hagfish. We found several sequences that contained a single conserved domain, or parts of a single conserved domain, but without finding the other conserved domain. These sequences were not included in the phylogenetic analyses, but they suggest that hagfish have a higher number of connexins than those that are included in the present phylogenetic analyses.

We were not able to find any sequences in the three lamprey species or in hagfish that showed evident similarities with *gje1*.

As the detailed substructure of the gene family in the Cyclostomata differed from the other vertebrate groups, it was of interest to take a preliminary look into the genomic organization (Supplementary Table S7). The Cyclostomata genome assemblies were rather fragmented, with many of the sequences on quite short scaffolds. Thus, only a few genes could be compared across the species, even within the Cyclostomata. The only genes that could be followed across the three lamprey species and hagfish were *gja3* and two *gjb1/8* genes. These genes were located at chromosome 49 in sea lamprey (*Petromyzon marinus*), chromosome 39 in Pacific lamprey (*Entosphenus tridentatus*; *gja3* was found on the unplaced scaffold JAAXLI020001559), scaffold KE993718 in Arctic lamprey (*Lethenteron camtschaticum*) and scaffold FYBX02010228 in inshore hagfish (*Eptatretus burgeri*). We believe that the *gjb1/8* sequence could be the origin of both *gjb1* and *gjb8*, and the latter further give rise to *gjb2* and *gjb6*. Looking at the genome location of these genes, *gja3* and *gjb8* are found closely linked in all fishes, and when *gjb2* and *gjb6* later evolved, they maintained the close chromosomal link with *gja3* during evolutionary history (in human, these three genes are found on chromosome 13), while *gjb1* is located on another chromosome (on the X chromosome in mammals).

As the lamprey and hagfish connexin genes did distribute into the alpha, beta, gamma and delta subfamilies, but only some of the lamprey genes showed affinities to the different orthologous sets of connexins (i.e., the lamprey genes only partly followed the finer subfamily structure details), we wondered if the lamprey and hagfish sequences could tend to aggregate together with the previously detected tunicate connexins [17]. However, adding the tunicate connexins to the vertebrate connexins (including the cyclostome sequences) did not prominently change the location of the lamprey and hagfish sequences in the phylogenetic tree or the relationship between the lamprey and hagfish sequences, and the tunicate sequences had a strong tendency to aggregate outside the vertebrate sequences. Thus, we did not find any obvious association between the tunicate connexins on the one side and the lamprey and hagfish connexins on the other side.

However, there is an intriguing biological phenomenon that could bring some uncertainty to determining the real number of connexin genes, and, for that matter, also the number of genes in other gene families, in the cyclostomes. The cyclostomes have a considerable degree of chromosome elimination [55,56], but we have not yet investigated whether this affects any connexin genes.

### 2.6. Comments on the Relationships between Gjb8 and GJB2/GJB6, and Gjb10 and GJB4/GJB5

Commonly, the teleosts genes in the *gjb8* orthogroup have received names like *gjb2*, *gjb2like* or *gjb6like*. We think it is likely that *gjb8* is the ancestor of *GJB2* and *GJB6* [17]. By the availability of many new genomes filling the gaps between teleosts and mammals, this suggestion is strengthened. The arguments for this suggestion are as follows: (i) In teleosts with two (or more) *gjb8* genes, the genes are located on distinct chromosomes. (ii) Depending on the species in non-teleost ray-finned fishes, there is one gene (spotted gar), one ohnologous pair (sterlet; Supplementary Table S5) or two genes (reedfish) on the same chromosome (chromosome 4 (scaffold NC_041397.1), positions 239894584–239893787 for *gjb8.1* and 239402441–239403241 for *gjb8.2* in opposite directions). (iii) In cartilaginous fishes and in amphibians, there is only one *gjb8* gene. (iv) There are three genes in the same direction on a single scaffold (JH12755) in *Latimeria*. (v) Mammalian *GJB2* and *GJB6* are chromosomally closely linked (in humans: chromosome 13, positions 20188901–20189581 for *GJB2* and positions 20222695–20223480 for *GJB6*, both in the reverse direction). We also found these genes closely linked in chicken *Gallus gallus* (chromosome 1, positions 180296063–180296740 for *gjb2* and positions 180276266–180277057 for *gjb6*) and on the same scaffold in the turtle *Pelodiscus sinensis* (NW_005871027.1, positions 544225–543548 for *gjb2* and positions 595742–594951 for *gjb6*, both in the same direction) and the lizard *Lacerta agilis* (chromosome 4, positions 21768411–21767731 for *gjb2* and positions 21798438–21797647 for *gjb6*, both in the reverse direction).

In short, the most likely possibility is that a single *gjb8* gene duplicated after the divergence of Amphibia but shortly before the divergence of Reptilia, which gave rise to the closely linked *GJB2* and *GJB6* in Reptilia and Mammalia. The short coexistence of the *GJB2* and *GJB6* genes before the split between Reptilia and Mammalia explains that both these genes tend to split into mammalian and reptilian phylogenetic clades.

**Figure 2:**
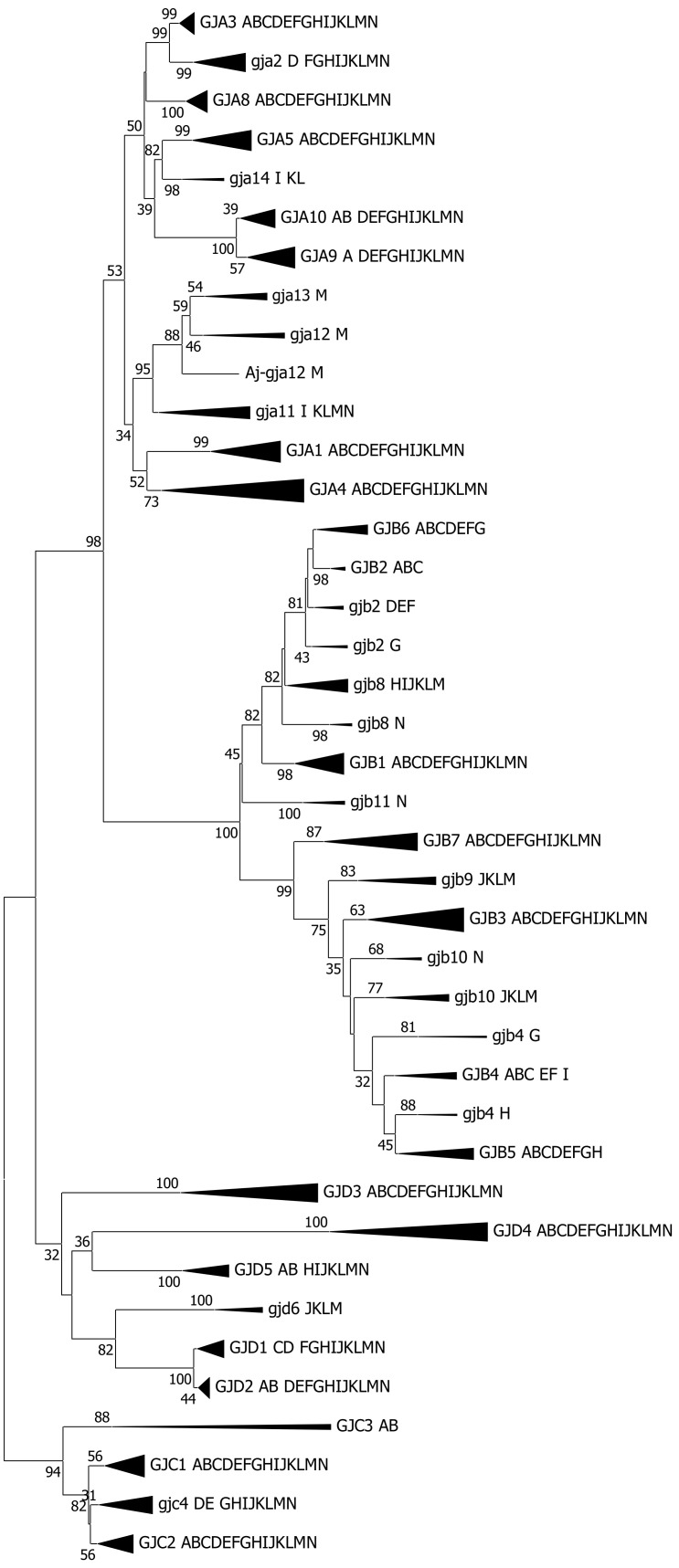
Compressed phylogenetic tree for connexins, except *GJE1*, in jawed vertebrates. The orthologous gene groups are indicated by their Greek nomenclature, using upper case letters if mammalian sequences are included in the orthogroup and lower-case letters if mammalian sequences are not included in the group. Sequences belonging to the *gje1*/*GJE1* group are not included in this tree (see text). The letters at each branch signify the vertebrate groups as follows: A, Placental mammals, B, Marsupials, C, Monotremata (platypus), D, Aves (birds), E, Crocodylia, F, Testudines (turtles), G, Squamata (lizards and snakes), H, Amphibia, I, Lobe-finned fish (*Latimeria*), J, Chondrostei—Polypteriformes (reedfish), K, Chondrostei—Acipenseriformes (sturgeons), L, Holostei (spotted gar), M, Teleostei and N, Chondrichthyes (cartilaginous fishes). A space is left in the sequence of the letters to make it more evident that one or more groups are lacking that particular gene. The tree was constructed on the basis of the amino acid sequence (205 positions and 559 sequences) of the conserved domains by the Neighbor-Joining method using the Jones–Taylor–Thornton (JTT) substitution matrix and with the rate variations among the sites corresponding to a gamma value = 1.1. Bootstrap statistics are shown at the branching points (500 iterations). Bootstrap values below 30 are not shown. The compressed branches are shown in expanded form in Supplementary Figures S44–S68.

We believe the situation is somewhat similar, but less clear-cut, for the relationship between *gjb10*, *GJB4*/*GJB5* and *GJB3. GJB3, GJB4* and *GJB5* are closely linked on human chromosome 1 (position 34757331–34758152 for *GJB5*, position 34761255–34762055 for *GJB4* and position 34784763–34785575 for *GJB3*, all in a forward direction). The closely linked conditions for the corresponding genes are similar for most of the species that are considered here. First, let us start pre-Chondrichthyes. All vertebrates have representatives in the *GJB3* orthogroup. At an early stage, just before the divergence of the cartilaginous fishes, we suggest that *GJB3* duplicated twice, generating *gjb9* and *gjb10.* The cartilaginous fishes diverged so early that the two newly generated genes did not acquire the characteristics of *gjb9* and *gjb10* before the divergence. In our phylogenetic analyses, one of these genes, chondrichthyan *gjb10*, tended to split off from *GJB3* and, depending on the model and gamma value, sometimes grouped together with *gjb10* from *Latimeria* (in Figure 2, this gene is located in the *GJB4* orthogroup, also seen in Supplementary Figure S55). Note that the consequence of this proposal is that the chondrichthyan *gjb9* corresponds to the gene in Supplementary Figure S55 called *gjb3.2.* In ray-finned fishes, *gjb10* evolved more and formed an orthogroup that phylogenetically always stuck together. Thus, all fishes possess *GJB3* and *gjb10*.

Now, let us approach these genes from the tetrapod side. There is little doubt that reptiles possess *gjb4* and *gjb5*, as mammalian and reptilian sequences generally stuck together for both orthogroups (although we have not found any bird *gjb4* sequences). Amphibia have two sets of genes that both phylogenetically group together with *GJB5*. Which of these two genes was located closer to the main GJB5 group varied according to whether nucleotides or amino acids were used in the tree building. We therefore looked at their positioning in the genome assemblies. In *Xenopus tropicalis*, the genes are on chromosome 2 in the forward direction: XM_018091588 (called *gjb4* in GenBank) in position 85000098–85000901, XM_004911610 (called *gjb5* in GenBank) in position 85009978–85010784 and NM_001112900 (*gjb3*) in position 85060961–85061713. In *Nanorana parkeri*, the genes are on scaffold NW_017306584 in the reverse direction: XM_018558022 (called *gjb5like* in GenBank) in position 893574–892810, XM_018558030 (also called *gjb5like* in GenBank) in position 874434–873634 and XM_01855031 (*gjb3*) in position 805315–804509. Thus, with regards to the positioning in the genome assemblies, *Xenopus* XM_018091588 and *Nanorana* XM_018558022 correspond to *gjb5*, and *Xenopus* XM_004911610 and *Nanorana* XM_018558030 correspond to *gjb4*.

We believe that the most likely evolutionary events for *gjb10* is that a gene duplication occurred just before the divergence of Amphibia, and the two resulting genes did not have the time to evolve the full characteristics of *GJB4* vs. *GJB5* at the time of splitting. The alternative possibility is that independent gene duplications occurred in the amphibian lineage and in Amniota, but from the principle of parsimony, we think this is less likely.

## 3. Discussion

The present phylogenetic analyses of the connexin genes include a wider range of vertebrate groups than in previous works [8,9,14,16,17,18]. This work confirms earlier observations of the connexins being divided into alpha, beta, gamma, delta and epsilon subfamilies in mammals, birds and teleosts and extends this to crocodiles, turtles, lizards, the lobe-finned fish *Latimeria*, ray-finned fishes in general and cartilaginous fishes. Therefore, the previous observations and conclusions are extended to the wider vertebrate society. Different aspects of these observations are discussed below.

### 3.1. On Genome/Gene Duplication and Naming

Our previous analyses [9,17] clearly indicated the presence of ohnologs of different connexin genes in teleosts. For sterlet sturgeon, we could, in the present work, define 23 ohnologous pairs, and for only three sequences, we found no signs of an ohnolog. Furthermore, the ohnologs in sterlet sturgeon had a high degree of sequence identity, with 19 ohnologous pairs >95% identical and four ohnologous pairs <95% identical at the nucleotide level (full length), clearly higher than those of the present teleosts that ranged from 72% to 82%, fully consistent with a more recent genome duplication in Acipenseriformes.

How to decide which ohnologs should be named “a” or “b”? One could theoretically wish to name the original gene “a” (or “.1” for a tandem gene duplication), provided that the original gene can be identified. However, that is often not the case [57], and this also seems to apply for the connexins. There are several possible approaches for deciding the naming, some more systematic than others. A few zebrafish ohnologs were named “a” or “b” in GenBank entries when we initiated this work [9], and in our suggestions, we took these into consideration (new entries or entries updated after September 2020 are not included). We also took several teleost species into consideration (see Table 2 in [9]) to minimize the effects of potential chromosomal reorganizations that may have occurred in certain subgroups of teleosts, giving the results shown in Supplementary Tables S2–S4. However, the consequence is that, in some species, “a” and “b” ohnologs will reside on the same chromosome. Using other criteria could change the “a”/”b” naming for several of the ohnologous pairs in the different species.

Independent gene and genome duplications have some bearings on orthology and the names given to the different genes. One main principle is that orthologous genes should have the same name in different species. However, gene naming rapidly becomes complicated considering independent gene duplications and gene losses, independent genome duplications (as in sturgeons and teleosts), reduplications of genomes (as in salmonids [58]) and species hybridization (as in *Xenopus laevis* [59]). Assume that a gene randomly called *gene1* goes through a gene duplication in the lineage leading to zebrafish and an independent later gene duplication in the lineage leading to pufferfish. In both species, this duplicated set of genes should then be called *gene1.1* and *gene1.2*, according to the Zebrafish Nomenclature Conventions [11]. As the genes are the results of independent gene duplications, they should not have identical names in the two species.

Similar problems arise with independent genome duplications, like the ones that did occur in the early teleosts and the much later one that occurred in Acipenseriformes. The ohnologous pairs in each of the species are to be called *gene1a* and *gene1b*, but *gene1b* in zebrafish and in sturgeon are not orthologs, assuming that *gene1a* can be defined as the original gene (which might not be possible). The simplest, but unsatisfactory possibility that violates the intentions of gene naming conventions [10,11,60] is the acceptance that certain types of non-orthologous genes may have the same name. This is already extensively practiced today [9].

### 3.2. The Number of Connexin Genes in Gnathostomata

Our previous work [17] suggested that birds, represented by chicken (*Gallus gallus*), have 16 connexin sequences, which is a lower number than in mammals with 19–22 functional genes and Amphibia represented by *Xenopus tropicalis* with 23 genes. Seemingly, the lower number of connexins is in line with the view that birds have streamlined their genome and lost 300 to 700 genes [23,24,25]. However, analyzing the transcriptome from five bird species [50], more than 80% of the presumed missing genes were recovered as transcripts, suggesting that many avian genes are located in areas that are difficult to sequence or assemble in a proper way. We show here that birds are in fact on par with the other tetrapods regarding the number of connexin genes, although the exact number of connexin genes may not be the same for each species within each vertebrate group. We found a total of 22 connexin genes in birds vs. 22 in placental mammals (23 with the *GJA1* gene duplication that generates functional *GJA6* in rodents and some other mammals), 21 in marsupials (22 if a marsupial-specific gene duplication of *GJC3* is included), 20 in platypus, 21 in crocodiles, 23 in turtles, 22 in lizards, 23 in Amphibia (24 if the amphibian-specific gene duplication of *gja4* is included), 25 in the lobe-finned fish *Latimeria,* 26 in both the polypteriformian Chondrostei and in Holostei and 23 in Chondrichthyes. The acipenseriformian Chondrostei (sturgeons) and teleosts have, so far, the highest numbers of connexins genes due to genome duplications (occurring independently), with 39–46 genes in teleosts and 49 in sterlet sturgeon. A brief overview is shown in Supplementary Table S8. Although not investigated, high numbers are also expected in *Xenopus laevis*, which is a 17-million-year-old hybrid between two closely related frogs [59] and in salmonid fishes [58] that went through an additional genome duplication around 80 million years ago, occurring on the top of the teleost genome duplication around 350 million years ago [19].

### 3.3. The Connexin Gene Family Substructure

It was previously shown that teleosts contained a fully developed connexin gene family, with essentially the same subfamily structures as found in mammals [9,17]. It is therefore not surprising that other ray-finned fishes that diverged before the teleost genome duplication in fact also show the same subfamily structure, with only a few differences, mainly due to gene duplications and gene losses. It was also easy to distinguish the independent genome duplications that occurred in the early teleosts on one side and the later genome duplication that occurred in the sturgeon lineage at a later point of time (see, also, the discussion on ohnology above). Here, we show that cartilaginous fishes also have a fully developed subfamily structure, and there are only a few gene duplications and gene losses that distinguish cartilaginous fishes from the ray-finned fishes. Most orthogroups where sequences from ray-finned fishes are found also contain cartilaginous fish sequences (as before, if the gene name is in upper case letters, the gene group contain mammalian representatives): *Gja2*, *GJA3*, *GJA4*, *GJA5*, *GJA8*, *GJA9*, *GJA10* and *gja11*; *GJB1*, *GJB3*, *GJB7*, *gjb8* and *gjb10* (see Results); *GJC1*, *GJC2* and *gjc4*; *GJD1*, *GJD2*, *GJD3*, *GJD4* and *GJD5* and, finally, *GJE1*. In a single gene group, only representatives from the cartilaginous fishes are found, namely *gjb11*. On the other hand, there are three gene groups where only sequences from ray-finned fishes are found: *gja12*, *gja13* and *gjb9* (see Section 2.6). If we add the lobe-finned fish *Latimeria* to the ray-finned fishes, we can also add two more gene groups: *gja11* and *gja14*. *Gjd6* is unique in the sense that it was found in ray-finned fishes and in cyclostomes but not (yet) in chondrichthyans. We conclude that all Gnathostomata, from Chondrichthyes and upwards, have the same connexin gene family substructure, although the details may differ due to group-specific gene duplications and gene losses.

### 3.4. Cyclostomata vs. Gnathostomata Connexin Gene Families

Historically, Cephalochordata (i.e., amphioxus) have often been considered as having diverged later than the tunicates, but this does not reflect reality [61]; tunicates are the more closely related to the vertebrates. No connexins are found in amphioxus, while tunicates have connexins [17,26]. A previous work showed that tunicate connexins (i) had low sequence identities, (ii) could not be clearly associated with any known vertebrate connexin sequence group and (iii) did not appear to have any recognizable subfamily structure [17]. That work left a gap between the tunicates and the teleosts. It is now possible to fill the gap due to the recent availability of genomes from non-teleosts Actinopterygii, Chondrichthyes and Cyclostomata. The present work clearly shows that the vertebrate groups from mammals to chondrichthyans do show the classic gene family substructures. Thus, there are two obvious jumps in the evolution of the subfamily structures: first, from tunicates to Cyclostomata, and then, from Cyclostomata to Chondrichthyes, where the subfamily structure is established.

The early vertebrate went through two genome duplications [62,63,64,65], and the timing of the genome duplications is of course relevant for the two jumps in the connexin family structure. The genome duplications occurred after the split between tunicates and vertebrates, but there is somewhat conflicting evidence as to whether genome duplications occurred or were completed before the Cyclostomata–Gnathostomata split [66,67] or after [68], although the present evidence seems to weigh in at the earlier point of time [69]. Thus, there were ample possibilities of divergence for the connexin genes between the tunicate–vertebrate split and the Cyclostomata–Gnathostomata split, creating the primitive connexin family structure we find in Cyclostomata with members within the alpha, beta, gamma and delta subfamilies. Some of the cyclostome connexins have acquired an affinity to specific ortholog group(s), concerning both (i) their phylogenetic position in the tree and (ii) their higher sequence similarity with the most closely positioned gnathostome connexin. This concerns *gja3*, *gja8*, *gja9/10*, *gjb1/8*, *gjd1/2*, *gjd3* and *gjd6*. However, for a number of cyclostome genes, it could not be determined which gnathostome connexin gene subfamily they were most closely related to. For example, there are two clusters of cyclostome genes in the gamma subfamily: a smaller cluster that we called *gjc-gen1*, which tends to be located closer to *gjc1* and *gjc2* and a larger cluster that we called *gjc-gen2*–*gjc-gen5*, which tends to be located closer to *gjc4*. While chondrichthyan *gjc1*, *gjc2* and *gjc4* have 70–80% sequence identities (for amino acid sequences in the conserved domains) within a specific species, the corresponding sequence identities for lamprey *gjc-gen1*–*gjc-gen5* are within 60–70%. For comparison, the sequence identity between the conserved domains of human *GJC1* and *GJC2* is around 76%. Although not definitive, this comparison suggests that the characteristics for the three gnathostome gamma family members, *gjc1*, *gjc2* and *gjc4*, started to evolve after the cyclostome–gnathostome split.

Based on the differences in the connexin family between the cyclostomes and gnathostomes, it would be tempting to suggest that the second early vertebrate genome duplication (partly) occurred after the cyclostome–gnathostome split [68], although newer and more heavy evidence weighs against this possibility [52]. Thus, the most likely option is that several gene duplications occurred in the gnathostomal lineage before the divergence of the chondrichthyans. The resulting early vertebrate genome should then have a tendency for closely related connexins to be closely linked on a chromosome, as this is the most common result of a gene duplication. This was investigated in three chondrichthyan chromosomal assemblies (with more complete assemblies than those of elephant shark, little skate and whale shark) (Supplementary Table S6). There are some examples of chromosomally linked genes that are closely related, like *gja1* and *gja11*, and *gjb3* and *gjb10*. However, in other cases, more distantly related genes are tightly linked, like *gjb1* and *gja2*, *gjb8* and *gja3* and *gja4* and *gjb3.2*. In these cases, we note that there is one beta gene and one alpha gene, and especially, *gjb1* and *gjb8*, and *gja2* and *gja3* are closely related. Thus, if we exclude the possibility of genome duplication after the cyclostome–gnathostome split, some regional duplications followed by several chromosomal rearrangements must have occurred before the divergence of the chondrichthyans. We note that this may be consistent with an alternative hypothesis of a single genome duplication before the cyclostome–gnathostome split together with several segmental duplications occurring partly before and partly after the cyclostome–gnathostome split [70].

## 4. Materials and Methods

### 4.1. Databanks and Collection of Sequences

The species included and their evolutionary relationships are indicated in Figure 1. In many cases, we included two or more species in each vertebrate group, as this would more reliably support the presence or absence of connexin sequences, especially if the sequences were somewhat deviating. Occasionally, we also searched in other species in the same vertebrate group—in particular, in the following situations:

- if an expected sequence was not found in a species,
- if only a partial sequence was found, we tried to find the corresponding sequence in a closely related species and
- if a single sequence from a certain species phylogenetically was located in an unexpected way.

If no similar sequence was found in other species belonging to the same vertebrate group, the sequence was excluded from the analyses. Examples of sequences excluded for this reason are XM_008107785 (*gjb6like*) from green anole (*Anolis carolinensis*) and XM_007898730 (*gjb5like*) from elephant shark (*Callorhinchus milii*).

Additionally, some sequences were excluded if considerable parts of both conserved domains were lacking or we could only find one conserved domain.

Databanks—in particular, Ensembl and GenBank—were searched for connexin sequences. The previously identified connexins [8,9,17] used in the present work were re-checked for any potential updates or major revisions of their sequences. If there were only a few positions with single nucleotide differences between the databases or between our historical sequences and those in the present databases, we, in general, did not modify our sequences, even if we adopted the relevant accession number. Pseudogenes were excluded in these analyses. Newly predicted connexins found in the databanks were excluded if:

- They were very similar to the sequences already collected, with only a few nucleotide differences. The reasons for not including all sequences are (A) the sequences may come from different individuals of the same species, thus having small individual genetic variations, and do not reflect unique genes, (B) there could be errors in the genome assemblies, predicting gene duplications where there are, in reality, no gene duplications and (C) a gene duplication may have occurred in a single species, but in the present work, we are focusing on the larger picture. Examples of such excluded sequences are the zebra finch (*Taeniopygia guttata*) XM_002198742, XM_002186636 and XM_012571095 and the Chinese softshell turtle (*Pelodiscus sinensis*) XM_006125970.
- We were not able to define the proper conserved domains, including the cysteine codon signatures. Several predicted sequences did not contain the proper cysteine codon patterns in one or both of the conserved domains or the translation into a protein showed that the expected transmembrane domains deviated too much from the expected nonpolar sequences. An example of such excluded sequences is chicken (*Gallus gallus*) XM_015299660.

Genome assemblies (indicated in the Supplementary Figures and/or Tables) were searched using the conserved areas of previously identified connexins as bait. The hits were collected and systematized, and their complete sequences were built, if possible. All sequences are reported in the Supplementary Materials, including the accession numbers if the known or predicted sequences were present in the databases. In addition to the vertebrate species in Figure 1, the amphioxus genome was also researched, but we did not find any connexin genes.

### 4.2. Alignment of Conserved Domains

The conserved domains were determined and aligned as described previously [17,71] with 15 nucleotide extensions, as stated in [9]. This is also indicated with yellow and grey in the sequences given in the Supplementary Materials, and the conserved codons for the extracellular cysteines are indicated in green.

### 4.3. Nomenclature Harmonization by Orthology Consistency

The trees were constructed using the conserved domains [9,17,71] and MEGA7 software [72]. Before the major statistical analyses, we harmonized all names and investigated the consistency of the sequence localization (in essence, inferred orthology) in the tree. The names were harmonized by a tree-building process as follows. The trees were constructed by the Neighbor-Joining method using amino acids sequences, the Jones–Taylor–Thornton (JTT) substitution matrix, the gamma parameter was set to 1 and with pairwise deletion for the missing data treatment. No statistical test for phylogeny was used in this part of the work. The trees were built from three different starting points, adding group-by-group of the species (except Cyclostomata). For each step, phylogenetic trees were built. This was to ascertain that specific sequences were generally kept within the same group (i.e., showing consistent orthologies), whether there were a few or many sequences that aggregated into a specific orthologous group. The three building processes were: (i) Beginning with mammals, sequences were gradually added “downwards”, but non-teleost bony fishes and cartilaginous fishes (Chondrichthyes) were added before teleosts. This process was used for the initial name harmonization. Using these harmonized names, it was easy to see in the subsequent two building processes whether the sequences had consistent localizations in the trees. (ii) Beginning with teleosts, the other actinopterygian non-teleosts were first added, then the Chondrichthyes, followed by “upwards” building. (iii) Beginning with Chondrichthyes, the trees were gradually built “upwards”, except that teleosts were added as the last group.

The phylogenetic grouping generally showed a high degree of consistency during the whole process in all three cases, although certain sequences could change their detailed affiliations between neighboring gene groups as more sequences were added.

The harmonizing of gene names simplified the comparisons of the sequences during the text. All sequences were named according to previously established connexin sequences using the “Greek” nomenclature as determined by the Human and Mouse Nomenclature Committees [10,60] and indirectly by the Zebrafish Nomenclature Conventions [11]. We amended many sequence names to become more consistent, so all the sequences in an orthologous group have the same name. The names used were constructed as follows:

- If the sequence corresponded to a predicted or confirmed sequence in GenBank: (species abbreviation)-(potentially amended “Greek” name)-(accession number)-(database entry name if different from the amended “Greek” name). The species abbreviations are given in Figure 1. Example: for a species with the Latin name “*Latin name*” (abbreviated Ln), a predicted gene found in GenBank with the accession number “XM_number” may have the name *gjd2like*, and we find in our phylogenetic analyses that this sequence is a *gjd5* sequence. In the phylogenetic trees, the sequence will be called Ln-gjd5-XM_number-gjd2like, while, in the text, the same sequence will be called Ln-*gjd5*.
- If the sequence corresponded to a sequence predicted in Ensembl (but not in GenBank): (species abbreviation)-(potentially amended “Greek” name)-(abbreviated Ensembl gene number)-(database entry name if different from the amended “Greek” name).
- If the sequence was not predicted in any database, but a transcript with the name could be found in the GenBank Transcriptome Shotgun Assembly (TSA) database: (species abbreviation)-(amended “Greek” name)-(scaffold accession number:start position direction)-(TSA name).
- If the sequence was not predicted in any database, nor found in the TSA database, but could be found in the genome assemblies: (species abbreviation)-(correct “Greek” name)-(scaffold accession number:start position direction).

These names are also found in the Supplementary Materials. For better consistency of the detailed nomenclature, we used upper case letters for the specific mammalian sequences (including mouse, despite the recommendations given by the Mouse and Rat Nomenclature Committees) [60], and for all other species and groups, we used lower case letters. We also used upper case letters when referring to orthologous gene groups that contained mammalian sequences, while lower case letters were used for orthologous gene groups that did not contain any mammalian sequences.

### 4.4. Model Selection and Phylogenetic Analyses

**Figure 3:**
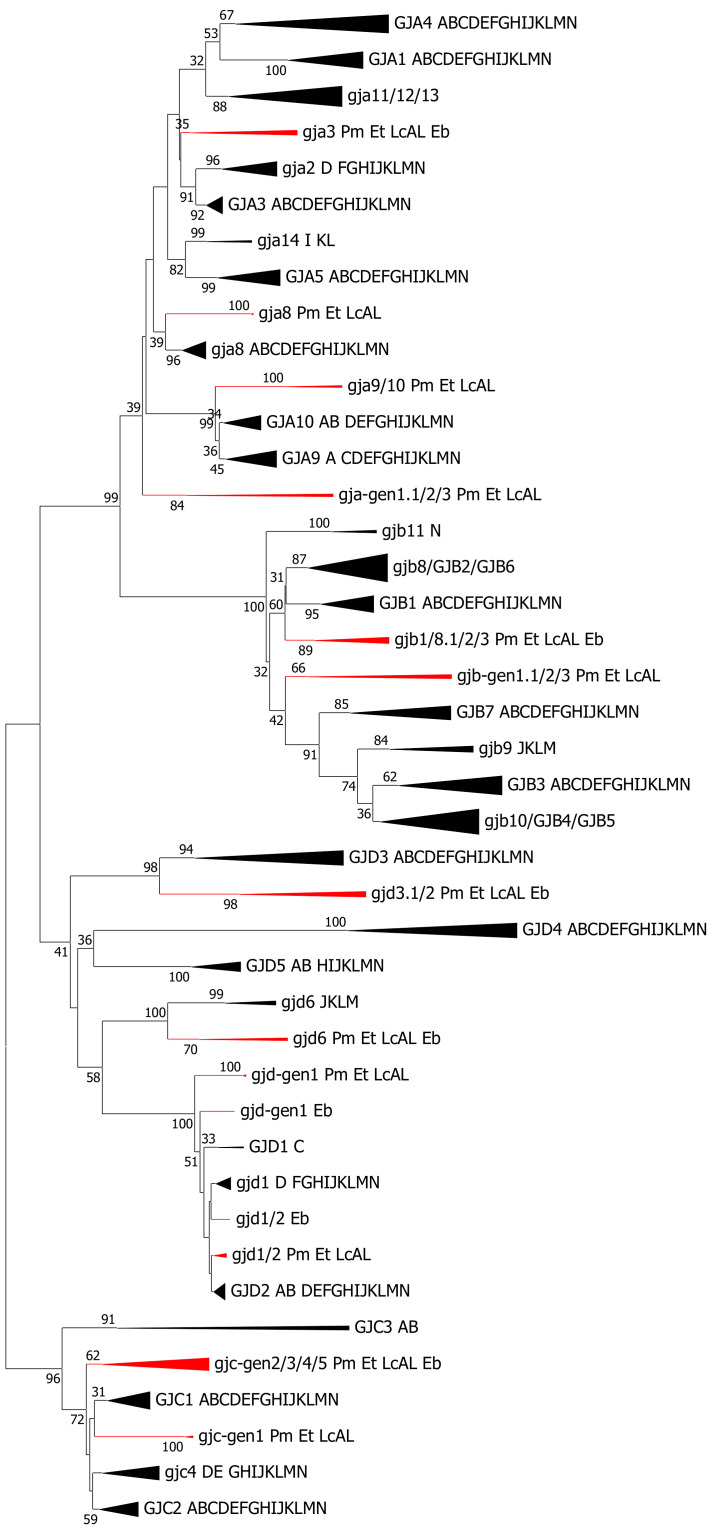
Compressed phylogenetic tree for connexins, except *GJE1*, in cyclostomes and gnathostomes. The annotation for the vertebrates is as described in the legend of Figure 2. The cyclostome sequences are indicated with their preliminary gene name (defined as described in the text) followed by the abbreviation describing the cyclostome species: Pm, *Petromyzon marinus*, sea lamprey; Et, *Entosphenus tridentatus*, Pacific lamprey; LcAL, *Lethentheron camtschaticum*, Arctic lamprey and Eb, *Eptatretus burgeri*, inshore hagfish. The cyclostome branches are indicated in red. The tree was constructed on the basis of the amino acid sequence (205 positions and 635 sequences) of the conserved domains by the Neighbor-Joining method using the JTT substitution matrix and with the rate variations among sites corresponding to a gamma value = 1.1 (same as in Figure 2) and with bootstrap statistics (500 iterations). Bootstrap values below 30 are not shown. Some of the orthogroups were united to save space and for giving a better overview. A Neighbor-Joining tree including *GJE1*, using the same JTT substitution matrix but with a gamma parameter = 0.8, is shown in Supplementary Figure S71.

Two sets of model selections were performed, to see if certain substitution matrices and certain levels of the gamma parameter were more relevant for the major statistical analyses. First, it is well-known that some of the orthologous groups of connexins are more conserved (for example, *gja1* and *gjc1*) and others are less conserved (for example, *gjd3* and *gjd4*) [8,17]. Therefore, we performed a model selection for amino acids for several of the orthogroups across the vertebrates (except lamprey and hagfish). There was generally little difference between the more advanced amino acid substitution matrices, like JTT, Dayhoff and WAG for each of the connexin groups, but the gamma parameter varied considerably, from less than 0.3 to more than 1.6, between the different orthogroups. Second, an overall model selection was performed both on the nucleotide level and amino acid level, and we used these models during the building for most of the phylogenetic trees. The details of the parameters are found in Supplementary Table S1. However, we do note that, when the gamma parameter was below 1, some of the orthogroups tended to split up in various ways that did not seem to have any biological meaning. Therefore, in Figure 2 and Figure 3, we used a gamma value = 1.1, keeping most orthogroups united. For a comparison of the different gamma values, Supplementary Table S1 contains data for three trees using amino acid sequences, the Neighbor-Joining method with a JTT substitution matrix and with three gamma values (0.8, 1.0 and 1.1).

## 5. Conclusions

- Cyclostomes do have a subfamily structure with alpha, beta, gamma and delta genes, but only a few of the genes show a phylogenetic affinity to genes that are found in the higher vertebrates. Thus, at the cyclostome–gnathostome split, an early version of the subfamily structure was established. The subfamily structure in cyclostomes is evidently more advanced than in tunicates [17].
- The connexin subfamily structures (alpha, beta, gamma, delta and epsilon) were established in cartilaginous fishes, and these subfamilies can be followed throughout the vertebrates. Most of the connexin genes in cartilaginous fishes can be classified into orthogroups together with genes from teleosts and other ray-finned fishes.
- It is not obviously evident how the gene family evolved from the “immature” version now found in cyclostomes to the “mature” version found in cartilaginous fishes. As it is assumed a whole genome duplication did not occur after the cyclostome–gnathostome split, the most likely possibility is a combination of gene duplications (that normally generates closely spaced duplicates) and regional duplications followed by rearrangements (that are more likely to generate similar genes on different chromosomes).
- There is no major change in the gene family in the transition from fishes to tetrapods.
- Birds have approximately the same number of connexin genes as other vertebrates (when genes generated from genome duplications or species hybridization are not considered).
- Due to the gene duplications and gene losses in certain vertebrate groups, the exact complement of the genes varies between the vertebrate groups. For example, *gjd5* was lost in the Reptilia early after the divergence, as none of the four vertebrate groups Squamata, Testudines, Crocodylia or Aves possess this gene, but it is found in all fish groups and in other tetrapods, including placental mammals and marsupials.
- There are few connexin genes that are specific to a single taxonomic group of vertebrates. *Gjb11* is specific to cartilaginous fishes. *Gja12* and *gja13* in teleosts are most likely generated by gene duplications from *gja11*, which, on the other hand, is found in most ray-finned fishes and cartilaginous fishes. For being more consistent with the nomenclature conventions, *gja12* and *gja13* should be fused into a single gene name, *gja12*, and hence, the genes should be detailed as *gja12.1*, *gja12.2*, *gja12.1.1*, *gja12.1.2*, etc. As a further consequence, the gene group that we call here *gja14* (found in non-teleost ray-finned fishes and *Latimeria*) could then be called *gja13*.
- We suggest that *GJB2* and *GJB6* were generated by gene duplication from *gjb8* shortly before the divergence of the Reptilia. Similarly, *GJB4* and *GJB5* were generated by gene duplication from *gjb10* shortly before the divergence of the Amphibia.

## Supplementary Materials

Supplementary materials can be found at https://www.mdpi.com/1422-0067/22/4/1584/s1.
