# Supplementary material for: Connexins during 500 Million Years—From Cyclostomes to Mammals
Source: Int J Mol Sci. 2021 Feb 4;22(4):1584. doi: 10.3390/ijms22041584 (PMC7914757; doi:10.3390/ijms22041584)
Supplement: Supplementary file 1 [file ijms-22-01584-s001.zip › Additional File 1 Supplementary Figures S1-S43.docx]

**Supplementary Materials**

**Additional File 1: Connexin sequences**

**Supplementary Figures S1 – S43**

**Mikalsen, SO; í Kongsstovu, S; Tausen, M.**

**Connexins during 500 million years – from cyclostomes to mammals**

**Common to all sequences in this document, is the following color coding:**

**Color coding: Yellow, Conserved domain as defined by Cruciani and Mikalsen (2007) Biol. Chem. 388, 253-264; Green, Conserved cysteine codons (cysteine signature); Grey, 15 nt extension at the ends of the conserved domains; Turquoise, Splice site. Other colors are explained where needed.**

Contents

[Supplementary Figure S1. Human (*Homo sapiens*) connexins. 4](#_Toc62483422)

[Supplementary Figure S2. Cattle (*Bos taurus*) *GJA9*. 11](#_Toc62483423)

[Supplemenatry Figure S3. Mouse (*Mus musculus*) connexins. 12](#_Toc62483424)

[Supplementary Figure S4. Black flying fox (*Pteropus alecto*) *GJD5*. 18](#_Toc62483425)

[Supplementary Figure S5. Opossum (*Monodelphis domestica*) connexins. 19](#_Toc62483426)

[Supplementary Figure S6. Tasmanian devil (*Sarcophilus harrisii*) *GJC3.* 26](#_Toc62483427)

[Supplementary Figure S7. Koala (*Phascolarctos cinereus*) *GJC3*. 27](#_Toc62483428)

[Supplementary Figure S8. Platypus (*Ornithorhynchus anatinus*) connexins 28](#_Toc62483429)

[Supplementary Figure S9. Chicken (*Gallus gallus*) connexins. 34](#_Toc62483430)

[Supplementary Figure S10. Zebra finch (*Taeniopygia guttata*) connexins. 41](#_Toc62483431)

[Supplementary Figure S11. Mallard (*Anas platyrhyncos*) *gjb5*, *gjb7* and *gjd3*. 49](#_Toc62483432)

[Supplementary Figure S12. Atlantic canary *(Serinus canaria*) *gjd5*. 50](#_Toc62483433)

[Supplementary Figure S13. Collared flycatcher (*Ficedula albicollis*) *gjc1*. 50](#_Toc62483434)

[Supplementary Figure S14. Australian saltwater crocodile (*Crocodylus porosus*) connexins. 51](#_Toc62483435)

[Supplementary Figure S15. Chinese alligator (*Alligator sinensis*) *gja3*. 58](#_Toc62483436)

[Supplementary Figure S16. American alligator (*Alligator mississippiensis*) *gjb4*. 59](#_Toc62483437)

[Supplementary Figure S17. Chinese softshell turtle (*Pelodiscus sinensis*) connexins. 59](#_Toc62483438)

[Supplementary Figure S18. Green sea turtle (*Chelonia mydas*) *gja2*. 66](#_Toc62483439)

[Supplementary Figure S19. Western painted turtle (*Chrysemus picta belli*) *gja2,* *gja3* and *gjd3*. 67](#_Toc62483440)

[Supplementary Figure S20. Green anole (*Anolis carolinensis*) connexins. 68](#_Toc62483441)

[Supplementary Figure S21. Some sand lizard (*Lacerta agilis*) connexins. 75](#_Toc62483442)

[Supplementary Figure S22. Japanese gecko (*Gekko japonicus*) *gja2*, *gja9* and *gjb7*. 79](#_Toc62483443)

[Supplementary Figure S23. Central bearded dragon (*Pogona vitticeps*) *gja4* and *gjb2*. 80](#_Toc62483444)

[Supplementary Figure S24. Tropical clawed frog (*Xenopus tropicalis*) connexins. 81](#_Toc62483445)

[Supplementary Figure S25. Tibetan frog (*Nanorana parkeri*) connexins. 89](#_Toc62483446)

[Supplementary Figure S26. Coelacanth (*Latimeria chalumnae*) connexins. 99](#_Toc62483447)

[Supplementary Figure S27. Reedfish (*Erpetoichthys calabricus*) connexins. 108](#_Toc62483448)

[Supplementary Figure S28. Sterlet sturgeon (*Acipenser ruthenus*) connexins. 117](#_Toc62483449)

[Supplementary Figure S29. Spotted gar (*Lepisosteus oculatus*) connexins. 136](#_Toc62483450)

[Supplementary Figure S30. Japanese eel (*Anguilla japonica*) connexins. 145](#_Toc62483451)

[Supplementary Figure S31. Atlantic herring (*Clupea harengus*) connexins. 160](#_Toc62483452)

[Supplementary Figure S32. Zebrafish (*Danio rerio*) connexins. 177](#_Toc62483453)

[Supplementary Figure S33. Atlantic cod (*Gadus morhua*) *gja11*, *gja12* and *gja13*. 191](#_Toc62483454)

[Supplementary Figure S34. Three-spined stickleback (*Gasterosteus aculeatus*) *gja11*, *gja12* and *gja13*. 192](#_Toc62483455)

[Supplementary Figure S35. Japanese pufferfish (*Takifugu rubripes*) *gja11*, *gja12* and *gja13*. 193](#_Toc62483456)

[Supplementary Figure S36. Whale shark (*Rhincodon typus*) connexins. 194](#_Toc62483457)

[Supplementary Figure S37. Little skate (*Leucoraja erinacea*) connexins. 202](#_Toc62483458)

[Supplementary Figure S38. Elephant shark (*Callorhinchus milii*) connexins. 210](#_Toc62483459)

[Supplementary Figure S39. Small-eyed rabbitfish (*Hydrolagus affinis*) *gja5*. 219](#_Toc62483460)

[Supplementary Figure S40. Sea lamprey (*Petromyzon marinus*) connexins. 220](#_Toc62483461)

[Supplementary Figure S41. Pacific lamprey (*Entosphenus tridentatus*) connexins. 230](#_Toc62483462)

[Supplementary Figure S42. Arctic lamprey (*Lethentheron camtschaticum*) connexins. 237](#_Toc62483463)

[Supplementary Figure S43. Inshore hagfish (*Eptatretus burgeri*) connexins. 243](#_Toc62483464)

# Supplementary Figure S1. Human (*Homo sapiens*) connexins.

The chronology of the sequences is according to the Greek nomenclature. Size nomenclature is indicated in parentheses. GenBank accession number is given for each entry.

>Hs-GJA1-NM_000165 (Cx43)

atgggtgactggagcgccttaggcaaactccttgacaaggttcaagcctactcaactgct

ggagggaaggtgtggctgtcagtacttttcattttccgaatcctgctgctggggacagcg

gttgagtcagcctggggagatgagcagtctgcctttcgttgtaacactcagcaacctggt

tgtgaaaatgtctgctatgacaagtctttcccaatctctcatgtgcgcttctgggtcctg

cagatcatatttgtgtctgtacccacactcttgtacctggctcatgtgttctatgtgatg

cgaaaggaagagaaactgaacaagaaagaggaagaactcaaggttgcccaaactgatggt

gtcaatgtggacatgcacttgaagcagattgagataaagaagttcaagtacggtattgaa

gagcatggtaaggtgaaaatgcgaggggggttgctgcgaacctacatcatcagtatcctc

ttcaagtctatctttgaggtggccttcttgctgatccagtggtacatctatggattcagc

ttgagtgctgtttacacttgcaaaagagatccctgcccacatcaggtggactgtttcctc

tctcgccccacggagaaaaccatcttcatcatcttcatgctggtggtgtccttggtgtcc

ctggccttgaatatcattgaactcttctatgttttcttcaagggcgttaaggatcgggtt

aagggaaagagcgacccttaccatgcgaccagtggtgcgctgagccctgccaaagactgt

gggtctcaaaaatatgcttatttcaatggctgctcctcaccaaccgctcccctctcgcct

atgtctcctcctgggtacaagctggttactggcgacagaaacaattcttcttgccgcaat

tacaacaagcaagcaagtgagcaaaactgggctaattacagtgcagaacaaaatcgaatg

gggcaggcgggaagcaccatctctaactcccatgcacagccttttgatttccccgatgat

aaccagaattctaaaaaactagctgctggacatgaattacagccactagccattgtggac

cagcgaccttcaagcagagccagcagtcgtgccagcagcagacctcggcctgatgacctg

gagatctag

>Hs-GJA3-NM_021954 (Cx46)

atgggcgactggagctttctgggaagactcttagaaaatgcacaggagcactccacggtc

atcggcaaggtttggctgaccgtgctgttcatcttccgcatcttggtgctgggggccgcg

gcggaggacgtgtggggcgatgagcagtcagacttcacctgcaacacccagcagccgggc

tgcgagaacgtctgctacgacagggccttccccatctcccacatccgcttctgggcgctg

cagatcatcttcgtgtccacgcccaccctcatctacctgggccacgtgctgcacatcgtg

cgcatggaagagaagaagaaagagagggaggaggaggagcagctgaagagagagagcccc

agccccaaggagccaccgcaggacaatccctcgtcgcgggacgaccgcggcagggtgcgc

atggccggggcgctgctgcggacctacgtcttcaacatcatcttcaagacgctgttcgag

gtgggcttcatcgccggccagtactttctgtacggcttcgagctgaagccgctctaccgc

tgcgaccgctggccctgccccaacacggtggactgcttcatctccaggcccacggagaag

accatcttcatcatcttcatgctggcggtggcctgcgcgtccctgctgctcaacatgctg

gagatctaccacctgggctggaagaagctcaagcagggcgtgaccagccgcctcggcccg

gacgcctccgaggccccgctggggacagccgatcccccgcccctgccccccagctcccgg

ccgcccgccgttgccatcgggttcccaccctactatgcgcacaccgctgcgcccctggga

caggcccgcgccgtgggctaccccggggccccgccaccagccgcggacttcaaactgcta

gccctgaccgaggcgcgcggaaagggccagtccgccaagctctacaacggccaccaccac

ctgctgatgactgagcagaactgggccaaccaggcggccgagcggcagcccccggcgctc

aaggcttacccggcagcgtccacgcctgcagcccccagccccgtcggcagcagctccccg

ccactcgcgcacgaggctgaggcgggcgcggcgcccctgctgctggatgggagcggcagc

agtctggaggggagcgccctggcagggacccccgaggaggaggagcaggccgtgaccacc

gcggcccagatgcaccagccgcccttgcccctcggagacccaggtcgggccagcaaggcc

agcagggccagcagcgggcgggccagaccggaggacttggccatctag

>Hs-GJA4-NM_002060 (Cx37)

atgggtgactggggcttcctggagaagttgctggaccaggtccaggagcactcgaccgtg

gtgggtaagatctggctgacggtgctcttcatcttccgcatcctcatcctgggcctggcc

ggcgagtcagtgtggggtgacgagcaatcagatttcgagtgtaacacggcccagccaggc

tgcaccaacgtctgctatgaccaggccttccccatctcccacatccgctactgggtgctg

cagttcctcttcgtcagcacacccaccctggtctacctgggccatgtcatttacctgtct

cggcgagaagagcggctgcggcagaaggagggggagctgcgggcactgccggccaaggac

ccacaggtggagcgggcgctggcggccgtagagcgtcagatggccaagatctcggtggca

gaagatggtcgcctgcgcatccgcggagcactgatgggcacctatgtcgccagtgtgctc

tgcaagagtgtgctagaggcaggcttcctctatggccagtggcgcctgtacggctggacc

atggagcccgtgtttgtgtgccagcgagcaccctgcccctacctcgtggactgctttgtc

tctcgccccacggagaagaccatcttcatcatcttcatgttggtggttggactcatctcc

ctggtgcttaacctgctggagttggtgcacctgctgtgtcgctgcctcagccgggggatg

agggcacggcaaggccaagacgcacccccgacccagggcacctcctcagacccttacacg

gaccaggtcttcttctacctccccgtgggccaggggccctcatccccaccatgccccacc

tacaatgggctctcatccagtgagcagaactgggccaacctgaccacagaggagaggctg

gcgtcttccaggccccctctcttcctggacccaccccctcagaatggccaaaaaccccca

agtcgtcccagcagctctgcttctaagaagcagtatgtatag

>Hs-GJA5-NM_005266 (Cx40)

atgggcgattggagcttcctgggaaatttcctggaggaagtacacaagcactcgaccgtg

gtaggcaaggtctggctcactgtcctcttcatattccgtatgctcgtgctgggcacagct

gctgagtcttcctggggggatgagcaggctgatttccggtgtgatacgattcagcctggc

tgccagaatgtctgctacgaccaggctttccccatctcccacattcgctactgggtgctg

cagatcatcttcgtctccacgccctctctggtgtacatgggccacgccatgcacactgtg

cgcatgcaggagaagcgcaagctacgggaggccgagagggccaaagaggtccggggctct

ggctcttacgagtacccggtggcagagaaggcagaactgtcctgctgggaggaagggaat

ggaaggattgccctccagggcactctgctcaacacctatgtgtgcagcatcctgatccgc

accaccatggaggtgggcttcattgtgggccagtacttcatctacggaatcttcctgacc

accctgcatgtctgccgcaggagtccctgtccccacccggtcaactgttacgtatcccgg

cccacagagaagaatgtcttcattgtctttatgctggctgtggctgcactgtccctcctc

cttagcctggctgaactctaccacctgggctggaagaagatcagacagcgatttgtcaaa

ccgcggcagcacatggctaagtgccagctttctggcccctctgtgggcatagtccagagc

tgcacaccaccccccgactttaatcagtgcctggagaatggccctgggggaaaattcttc

aatcccttcagcaataatatggcctcccaacaaaacacagacaacctggtcaccgagcaa

gtacgaggtcaggagcagactcctggggaaggtttcatccaggttcgttatggccagaag

cctgaggtgcccaatggagtctcaccaggtcaccgccttccccatggctatcatagtgac

aagcgacgtcttagtaaggccagcagcaaggcaaggtcagatgacctatcagtgtga

>Hs-GJA8-NM_005267 (Cx50)

atgggcgactggagtttcctggggaacatcttggaggaggtgaatgagcactccaccgtc

atcggcagagtctggctcaccgtgcttttcatcttccggatcctcatccttggcacggcc

gcagagttcgtgtggggggatgagcaatccgacttcgtgtgcaacacccagcagcctggc

tgcgagaacgtctgctacgacgaggcctttcccatctcccacattcgcctctgggtgctg

cagatcatcttcgtctccaccccgtccctgatgtacgtggggcacgcggtgcactacgtc

cgcatggaggagaagcgcaaaagccgcgaggcggaggagctgggccagcaggcggggact

aacggcggcccggaccagggcagcgtcaagaagagcagcggcagcaaaggcactaagaag

ttccggctggaggggaccctgctgaggacctacatctgccacatcatcttcaagaccctc

tttgaagtgggcttcatcgtgggccactacttcctgtacgggttccggatcctgcctctg

taccgctgcagccggtggccctgccccaatgtggtggactgcttcgtgtcccggcccacg

gagaaaaccatcttcatcctgttcatgttgtctgtggcctctgtgtccctattcctcaac

gtgatggagttgggccacctgggcctgaaggggatccggtctgccttgaagaggcctgta

gagcagcccctgggggagattcctgagaaatccctccactccattgctgtctcctccatc

cagaaagccaagggctatcagctcctagaagaagagaaaatcgtttcccactatttcccc

ttgaccgaggttgggatggtggagaccagcccactgcctgccaagcctttcaatcagttc

gaggagaagatcagcacaggacccctgggggacttgtcccggggctaccaagagacactg

ccttcctacgctcaggtgggggcacaagaagtggagggcgaggggccgcctgcagaggag

ggagccgaacccgaggtgggagagaagaaggaggaagcagagaggctgaccacggaggag

caggagaaggtggccgtgccagagggggagaaagtagagacccccggagtggataaggag

ggtgaaaaagaagagccgcagtcggagaaggtgtcaaagcaagggctgccagctgagaag

acaccttcactctgtccagagctgacaacagatgatgccagacccctgagcaggctaagc

aaagccagcagccgagccaggtcagacgatctaaCCGTATGA

>Hs-GJA9-NM_030772 (Cx58)

atgggggactggaatctccttggagatactctggaggaagttcacatccactccaccatg

attggaaagatctggctcaccatcctgttcatatttcgaatgcttgttctgggtgtagca

gctgaagatgtctggaatgatgagcagtctggcttcatctgcaatacagaacaaccaggc

tgcagaaatgtatgctacgaccaggcctttcctatctccctcattagatactgggttctg

caggtgatatttgtgtcttcaccatccctggtctacatgggccatgcattgtaccgactg

agagttcttgaggaagagaggcaaaggatgaaagctcagttaagagtagaactggaggag

gtagagtttgaaatgcctagggatcggaggagattggagcaagagctttgtcagctggag

aaaaggaaactaaataaagctccactcagaggaaccttgctttgcacttatgtgatacac

attttcactcgctctgtggttgaagttggattcatgattggacagtaccttttatatgga

tttcacttagagccgctatttaagtgccatggccacccgtgtccaaatataatcgactgt

tttgtctcaagaccaacagaaaagacaatattcctattatttatgcaatctatagccact

atttcacttttcttaaacattcttgaaattttccacctaggttttaaaaagattaaaaga

gggctttggggaaaatacaagttgaagaaggaacataatgaattccatgcaaacaaggca

aaacaaaatgtagccaaataccagagcacatctgcaaattcactgaagcgactcccttct

gcccctgattataatctgttagtggaaaagcaaacacacactgcagtgtaccctagttta

aattcatcttctgtattccagccaaatcctgacaatcatagtgtaaatgatgagaaatgc

attttggatgaacaggaaactgtactttctaatgagatttccacacttagtactagttgt

agtcattttcaacacatcagttcaaacaataacaaagacactcataaaatatttggaaaa

gaacttaatggtaaccagttaatggaaaaaagagaaactgaaggcaaagacagcaaaagg

aactactactctagaggtcaccgttctattccaggtgttgctatagatggagagaacaac

atgaggcagtcaccccaaacagttttctccttgccagctaactgcgattggaaaccgcgg

tggcttagagctacatggggttcctctacagaacatgaaaaccgggggtcacctcctaaa

ggtaacctcaagggccagttcagaaagggcacagtcagaacccttcctccttcacaagga

gattctcaatcacttgacattccaaacactgctgattctttgggagggctgtcctttgag

ccagggttggtcagaacctgtaataatcctgtttgtcctccaaatcacgtagtgtcccta

acgaacaatctcattggtaggcgggttcccacagatcttcagatctaa

>Hs-GJA10-NM_032602 (Cx62)

atgggggactggaacttattgggtggcatcctagaggaagttcactcccactcaaccata

gtggggaaaatctggctgaccatcctcttcatcttccgaatgctggtacttcgtgtggct

gctgaggatgtctgggatgatgaacagtcagcatttgcctgcaacacccggcagccaggt

tgcaacaatatctgttatgatgatgcattccctatctctttgatcaggttctgggtttta

cagatcatctttgtgtcttctccttctttggtctatatgggccatgcactttataggctc

agggcctttgagaaagacaggcagaggaaaaagtcacaccttagagcccagatggagaat

ccagatcttgacttggaggagcagcaaagaatagatagggaactgaggaggttagaggag

cagaagaggatccataaagtccctctgaaaggatgtctgctgcgtacttatgtcttacac

atcttgaccagatctgtgctggaagtaggattcatgataggccaatatattctctatggg

tttcaaatgcaccccctttacaaatgcactcaacctccttgccccaatgcggtggattgc

tttgtatccaggcccactgagaagacaattttcatgctttttatgcacagcattgcagcc

atttccttgttactcaatatactggaaatatttcatctaggcatcagaaaaattatgagg

acactttataagaaatccagcagtgagggcattgaggatgaaacaggccctccattccat

ttgaagaaatattctgtggcccagcagtgtatgatttgctcttcattgcctgaaagaatc

tctccacttcaagctaacaatcaacagcaagtcattcgagttaatgtgccaaagtctaaa

accatgtggcaaatcccacagccaaggcaacttgaagtagacccttccaatgggaaaaag

gactggtctgagaaggatcagcatagcggacagctccatgttcacagcccgtgtccctgg

gctggcagtgctggaaatcagcacctgggacagcaatcagaccattcctcatttggcctg

cagaatacaatgtctcagtcctggctaggtacaactacggctcctagaaactgtccatcc

tttgcagtaggaacctgggagcagtcccaggacccagaaccctcaggtgagcctctcaca

gatcttcatagtcactgcagagacagtgaaggcagcatgagagagagtggggtctggata

gacagatctcgcccaggcagtcgcaaggccagctttctgtccagattgttgtctgaaaag

cgacatctgcacagtgactcaggaagctctggttctcggaatagctcctgcttggatttt

cctcactgggaaaacagcccctcacctctgccttcagtcactgggcacagaacatcaatg

gtaagacaggcagccctaccgatcatggaactatcacaagagctgttccattctggatgc

tttctttttcctttctttcttcctggggtgtgtatgtatgtttgtgttgacagagaggca

gatggagggggagattatttatggagagataaaattattcattcgatacattcagttaaa

ttcaattcataa

>Hs-GJB1-NM_001097642 (Cx32)

atgaactggacaggtttgtacaccttgctcagtggcgtgaaccggcattctactgccatt

ggccgagtatggctctcggtcatcttcatcttcagaatcatggtgctggtggtggctgca

gagagtgtgtggggtgatgagaaatcttccttcatctgcaacacactccagcctggctgc

aacagcgtttgctatgaccaattcttccccatctcccatgtgcggctgtggtccctgcag

ctcatcctagtttccaccccagctctcctcgtggccatgcacgtggctcaccagcaacac

atagagaagaaaatgctacggcttgagggccatggggaccccctacacctggaggaggtg

aagaggcacaaggtccacatctcagggacactgtggtggacctatgtcatcagcgtggtg

ttccggctgttgtttgaggccgtcttcatgtatgtcttttatctgctctaccctggctat

gccatggtgcggctggtcaagtgcgacgtctacccctgccccaacacagtggactgcttc

gtgtcccgccccaccgagaaaaccgtcttcaccgtcttcatgctagctgcctctggcatc

tgcatcatcctcaatgtggccgaggtggtgtacctcatcatccgggcctgtgcccgccga

gcccagcgccgctccaatccaccttcccgcaagggctcgggcttcggccaccgcctctca

cctgaatacaagcagaatgagatcaacaagctgctgagtgagcaggatggctccctgaaa

gacatactgcgccgcagccctggcaccggggctgggctggctgaaaagagcgaccgctgc

tcggcctgctga

>Hs-GJB2-NM_004004 (Cx26)

atggattggggcacgctgcagacgatcctggggggtgtgaacaaacactccaccagcatt

ggaaagatctggctcaccgtcctcttcatttttcgcattatgatcctcgttgtggctgca

aaggaggtgtggggagatgagcaggccgactttgtctgcaacaccctgcagccaggctgc

aagaacgtgtgctacgatcactacttccccatctcccacatccggctatgggccctgcag

ctgatcttcgtgtccacgccagcgctcctagtggccatgcacgtggcctaccggagacat

gagaagaagaggaagttcatcaagggggagataaagagtgaatttaaggacatcgaggag

atcaaaacccagaaggtccgcatcgaaggctccctgtggtggacctacacaagcagcatc

ttcttccgggtcatcttcgaagccgccttcatgtacgtcttctatgtcatgtacgacggc

ttctccatgcagcggctggtgaagtgcaacgcctggccttgtcccaacactgtggactgc

tttgtgtcccggcccacggagaagactgtcttcacagtgttcatgattgcagtgtctgga

atttgcatcctgctgaatgtcactgaattgtgttatttgctaattagatattgttctggg

aagtcaaaaaagccagtttaa

>Hs-GJB3-NM_024009 (Cx31)

atggactggaagacactccaggccctactgagcggtgtgaacaagtactccacagcgttc

gggcgcatctggctgtccgtggtgttcgtcttccgggtgctggtatacgtggtggctgca

gagcgcgtgtggggggatgagcagaaggactttgactgcaacaccaagcagcccggctgc

accaacgtctgctacgacaactacttccccatctccaacatccgcctctgggccctgcag

ctcatcttcgtcacatgcccctcgctgctggtcatcctgcacgtggcctaccgtgaggag

cgggagcgccggcaccgccagaaacacggggaccagtgcgccaagctgtacgacaacgca

ggcaagaagcacggaggcctgtggtggacctacctgttcagcctcatcttcaagctcatc

attgagttcctcttcctctacctgctgcacactctctggcatggcttcaatatgccgcgc

ctggtgcagtgtgccaacgtggccccctgccccaacatcgtggactgctacattgcccga

cctaccgagaagaaaatcttcacctacttcatggtgggcgcctccgccgtctgcatcgta

ctcaccatctgtgagctctgctacctcatctgccacagggtcctgcgaggcctgcacaag

gacaagcctcgagggggttgcagcccctcgtcctccgccagccgagcttccacctgccgc

tgccaccacaagctggtggaggctggggaggtggatccagacccaggcaataacaagctg

caggcttcagcacccaacctgacccccatctga

>Hs-GJB4-NM_153212 (Cx30.3)

atgaactgggcatttctgcagggcctgctgagtggcgtgaacaagtactccacagtgctg

agccgcatctggctgtctgtggtgttcatctttcgtgtgctggtgtacgtggtggcagcg

gaggaggtgtgggacgatgagcagaaggactttgtctgcaacaccaagcagcccggctgc

cccaacgtctgctatgacgagttcttccccgtgtcccacgtgcgcctctgggccctacag

ctcatcctggtcacgtgcccctcactgctcgtggtcatgcacgtggcctaccgcgaggaa

cgcgagcgcaagcaccacctgaaacacgggcccaatgccccgtccctgtacgacaacctg

agcaagaagcggggcggactgtggtggacgtacttgctgagcctcatcttcaaggccgcc

gtggatgctggcttcctctatatcttccaccgcctctacaaggattatgacatgccccgc

gtggtggcctgctccgtggagccttgcccccacactgtggactgttacatctcccggccc

acggagaagaaggtcttcacctacttcatggtgaccacagctgccatctgcatcctgctc

aacctcagtgaagtcttctacctggtgggcaagaggtgcatggagatcttcggccccagg

caccggcggcctcggtgccgggaatgcctacccgatacgtgcccaccatatgtcctctcc

cagggagggcaccctgaggatgggaactctgtcctaatgaaggctgggtcggccccagtg

gatgcaggtgggtatccataa

>Hs-GJB5-NM_005268 (Cx31.1)

atgaactggagtatctttgagggactcctgagtggggtcaacaagtactccacagccttt

gggcgcatctggctgtctctggtcttcatcttccgcgtgctggtgtacctggtgacggcc

gagcgtgtgtggagtgatgaccacaaggacttcgactgcaatactcgccagcccggctgc

tccaacgtctgctttgatgagttcttccctgtgtcccatgtgcgcctctgggccctgcag

cttatcctggtgacatgcccctcactgctcgtggtcatgcacgtggcctaccgggaggtt

caggagaagaggcaccgagaagcccatggggagaacagtgggcgcctctacctgaacccc

ggcaagaagcggggtgggctctggtggacatatgtctgcagcctagtgttcaaggcgagc

gtggacatcgcctttctctatgtgttccactcattctaccccaaatatatcctccctcct

gtggtcaagtgccacgcagatccatgtcccaatatagtggactgcttcatctccaagccc

tcagagaagaacattttcaccctcttcatggtggccacagctgccatctgcatcctgctc

aacctcgtggagctcatctacctggtgagcaagagatgccacgagtgcctggcagcaagg

aaagctcaagccatgtgcacaggtcatcacccccacggtaccacctcttcctgcaaacaa

gacgacctcctttcgggtgacctcatctttctgggctcagacagtcatcctcctctctta

ccagaccgcccccgagaccatgtgaagaaaaccatcttgtga

>Hs-GJB6-NM_001110219 (Cx30)

atggattgggggacgctgcacactttcatcgggggtgtcaacaaacactccaccagcatc

gggaaggtgtggatcacagtcatctttattttccgagtcatgatcctcgtggtggctgcc

caggaagtgtggggtgacgagcaagaggacttcgtctgcaacacactgcaaccgggatgc

aaaaatgtgtgctatgaccactttttcccggtgtcccacatccggctgtgggccctccag

ctgatcttcgtctccaccccagcgctgctggtggccatgcatgtggcctactacaggcac

gaaaccactcgcaagttcaggcgaggagagaagaggaatgatttcaaagacatagaggac

attaaaaagcagaaggttcggatagaggggtcgctgtggtggacgtacaccagcagcatc

tttttccgaatcatctttgaagcagcctttatgtatgtgttttacttcctttacaatggg

taccacctgccctgggtgttgaaatgtgggattgacccctgccccaaccttgttgactgc

tttatttctaggccaacagagaagaccgtgtttaccatttttatgatttctgcgtctgtg

atttgcatgctgcttaacgtggcagagttgtgctacctgctgctgaaagtgtgttttagg

agatcaaagagagcacagacgcaaaaaaatcaccccaatcatgccctaaaggagagtaag

cagaatgaaatgaatgagctgatttcagatagtggtcaaaatgcaatcacaggtttccca

agctaa

>Hs-GJB7-NM_198568 (Cx25)

atgagttggatgttcctcagagatctcctgagtggagtaaataaatactccactgggact

ggatggatttggctggctgtcgtgtttgtcttccgtttgctggtctacatggtggcagca

gagcacgtgtggaaagatgagcagaaagagtttgagtgcaacagtagacagcccggttgc

aaaaatgtgtgttttgatgacttcttccccatttcccaagtcagactttgggccttacaa

ctgataatggtctccacaccttcacttctggtggttttacatgtagcctatcatgagggt

agagagaaaaggcacagaaagaaactctatgtcagcccaggtacaatggatgggggccta

tggtacgcttatcttatcagcctcattgttaaaactggttttgaaattggcttccttgtt

ttattttataagctatatgatggctttagtgttccctaccttataaagtgtgatttgaag

ccttgtcccaacactgtggactgcttcatctccaaacccactgagaagacgatcttcatc

ctcttcttggtcatcacctcatgcttgtgtattgtgttgaatttcattgaactgagtttt

ttggttctcaagtgctttattaagtgctgtctccaaaaatatttaaaaaaacctcaagtc

ctcagtgtgtga

>Hs-GJC1-NM_005497 (Cx45)

atgagttggagcttcctgactcgcctgctagaggagattcacaaccattccacatttgtg

gggaagatctggctcactgttctgattgtcttccggatcgtccttacagctgtaggagga

gaatccatctattacgatgagcaaagcaaatttgtgtgcaacacagaacagccgggctgt

gagaatgtctgttatgatgcgtttgcacctctctcccatgtacgcttctgggtgttccag

atcatcctggtggcaactccctctgtgatgtacctgggctatgctatccacaagattgcc

aaaatggagcacggtgaagcagacaagaaggcagctcggagcaagccctatgcaatgcgc

tggaaacaacaccgggctctggaagaaacggaggaggacaacgaagaggatcctatgatg

tatccagagatggagttagaaagtgataaggaaaataaagagcagagccaacccaaacct

aagcatgatggccgacgacggattcgggaagatgggctcatgaaaatctatgtgctgcag

ttgctggcaaggaccgtgtttgaggtgggttttctgatagggcagtattttctgtatggc

ttccaagtccacccgttttatgtgtgcagcagacttccttgtcctcataagatagactgc

tttatttctagacccactgaaaagaccatcttccttctgataatgtatggtgttacaggc

ctttgcctcttgcttaacatttgggagatgcttcatttagggtttgggaccattcgagac

tcactaaacagtaaaaggagggaacttgaggatccgggtgcttataattatcctttcact

tggaatacaccatctgctccccctggctataacattgctgtcaaaccagatcaaatccag

tacaccgaactgtccaatgctaagatcgcctacaagcaaaacaaggccaacacagcccag

gaacagcagtatggcagccatgaggagaacctcccagctgacctggaggctctgcagcgg

gagatcaggatggctcaggaacgcttggatctggcagttcaggcctacagtcaccaaaac

aaccctcatggtccccgggagaagaaggccaaagtggggtccaaagctgggtccaacaaa

agcactgccagtagcaaatcaggggatgggaagacctccgtctggatttaa

>Hs-GJC2-NM_020435 (Cx47)

atgagctggagcttcctgacgcggctgctggaggagatccacaaccactccaccttcgtg

ggcaaggtgtggctcacggtgctggtggtcttccgcatcgtgctgacggctgtgggcggc

gaggccatctactcggacgagcaggccaagttcacttgcaacacgcggcagccaggctgc

gacaacgtctgctatgacgccttcgcgcccctgtcgcacgtgcgcttctgggtcttccag

attgtggtcatctccacgccctcggtcatgtacctgggctacgccgtgcaccgcctggcc

cgtgcgtctgagcaggagcggcgccgcgccctccgccgccgcccggggccacgccgcgcg

ccccgagcgcacctgccgcccccgcacgccggctggcctgagcccgccgacctgggcgag

gaggagcccatgctgggcctgggcgaggaggaggaggaggaggagacgggggcagccgag

ggcgccggcgaggaagcggaggaggcaggcgcggaggaggcgtgcactaaggcggtcggc

gctgacggcaaggcggcagggaccccgggcccgaccgggcaacacgatgggcggaggcgc

atccagcgggagggcctgatgcgcgtgtacgtggcccagctggtggccagggcagctttc

gaggtggccttcctggtgggccagtacctgctgtacggcttcgaggtgcgaccgttcttt

ccctgcagccgccagccctgcccgcacgtggtggactgcttcgtgtcgcgccctactgaa

aagacggtcttcctgctggttatgtacgtggtcagctgcctgtgcctgctgctcaacctc

tgtgagatggcccacctgggcttgggcagcgcgcaggacgcggtgcgcggccgccgcggc

cccccggcctccgcccccgcccccgcgccgcggcccccgccctgcgccttccctgcggcg

gccgctggcttggcctgcccgcccgactacagcctggtggtgcgggcggccgagcgcgct

cgggcgcatgaccagaacctggcaaacctggccctgcaggcgctgcgcgacggggcagcg

gctggggaccgcgaccgggacagttcgccgtgcgtcggcctccctgcggcctcccggggg

ccccccagagcaggcgcccccgcgtcccggacgggcagtgctacctctgcgggcactgtc

ggggagcagggccggcccggcacccacgagcggccaggagccaagcccagggctggctcc

gagaagggcagtgccagcagcagggacgggaagaccaccgtgtggatctga

>Hs-GJC3-NM_181538 (Cx31.3) Splice site

atgtgtggcaggttcctgcggcggctgctggcggaggagagccggcgctccacccccgtg

gggcgcctcttgcttcccgtgctcctgggattccgccttgtgctgctggctgccagtggg

cctggagtctatggtgatgagcagagtgaattcgtgtgtcacacccagcagccgggctgc

aaggctgcctgcttcgatgccttccaccccctctccccgctgcgtttctgggtcttccag

gtcatcttggtggctgtacccagcgccctctatatgggtttcactctgtatcacgtgatc

tggcactgggaattatcaggaaaggggaaggaggaggagaccctgatccagggacgggag

ggcaacacagatgtcccaggggctggaagcctcaggctgctctgggcttatgtggctcag

ctgggggctcggcttgtcctggagggggcagccctggggttgcagtaccacctgtatggg

ttccagatgcccagctcctttgcatgtcgccgagaaccttgccttggtagtataacctgc

aatctgtcccgcccctctgagaagaccattttcctaaagaccatgtttggagtcagcggt

ttctgtctcttgtttacttttttggagcttgtgcttctgggtttggggagatggtggagg

acctggaagcacaaatcttcctcttctaaatacttcctaacttcagagagcaccagaaga

cacaagaaagcaaccgatagcctcccagtggtggaaaccaaagagcaatttcaagaagca

gttccaggaagaagcttagcccaggaaaaacaaagaccagttggacccagagatgcctga

>Hs-GJD2-NM_020660 (Cx36) Splice site

atgggggaatggaccatcttggagaggctgctagaagccgcggtgcagcagcactccact

atgatcgggaggatcctgttgactgtggtggtgatcttccggatcctcattgtggccatt

gtgggggagacggtgtacgatgatgagcagaccatgtttgtgtgcaacaccctgcagccc

ggctgtaaccaggcctgctatgaccgcgccttccccatctcccacatacgttactgggtc

ttccagatcataatggtgtgtacccccagtctttgcttcatcacctactctgtgcaccag

tccgccaagcagcgagaacgccgctactctacagtcttcctagccctggacagagacccc

cctgagtccataggaggtcctggaggaactgggggtgggggcagtggtgggggcaaacga

gaagataagaagttgcaaaatgctattgtgaatggggtgctgcagaacacagagaacacc

agtaaggagacagagccagattgtttagaggttaaggagctgactccacacccatcaggt

ctacgcactgcatcaaaatccaagctcagaaggcaggaaggcatctcccgcttctacatt

atccaagtggtgttccgaaatgccctggaaattgggttcctggttggccaatattttctc

tatggctttagtgtcccagggttgtatgagtgtaaccgctacccctgcatcaaggaggtg

gaatgttatgtgtcccggccaactgagaagactgtctttctagtgttcatgtttgctgta

agtggcatctgtgttgtgctcaacctggctgaactcaaccacctgggatggcgcaagatc

aagctggctgtgcgaggggctcaggccaagagaaagtcaatctatgagattcgtaacaag

gacctgccaagggtcagtgttcccaattttggcaggactcagtccagtgactctgcctat

gtgtga

>Hs-GJD3-NM_152219 (Cx31.9)

atgggggagtgggcgttcctgggctcgctgctggacgccgtgcagctgcagtcgccgctc

gtgggccgcctctggctggtggtcatgctgatcttccgcatcctggtgctggccacggtg

ggcggcgccgtgttcgaggacgagcaagaggagttcgtgtgcaacacgctgcagccgggc

tgtcgccagacctgctacgaccgcgccttcccggtctcccactaccgcttctggctcttc

cacatcctgctgctctcggcgcccccggtgctgttcgtcgtctactccatgcaccgggca

ggcaaggaggcgggcggcgctgaggcggcggcgcagtgcgcccccggactgcccgaggcc

cagtgcgcgccgtgcgccctgcgcgcccgccgcgcgcgccgctgctacctgctgagcgtg

gcgctgcgcctgctggccgagctgaccttcctgggcggccaggcgctgctctacggcttc

cgcgtggccccgcacttcgcgtgcgccggtccgccctgcccgcacacggtcgactgcttc

gtgagccggcccaccgagaagaccgtcttcgtgctcttctatttcgcggtggggctgctg

tcggcgctgctcagcgtagccgagctgggccacctgctctggaagggccgcccgcgcgcc

ggggagcgtgacaaccgctgcaaccgtgcacacgaagaggcgcagaagctgctcccgccg

ccgccgccgccacctccgccaccggccctgccctcccggcgccccggccccgagccgtgc

gccccgccggcctatgcgcacccggcgccggccagcctccgcgagtgcggcagcggccgc

ggcaaggcgtcaccggccaccggccgccgagatctggccatctag

>Hs-GJD4-NM_153368 (Cx40.1) Splice site

atggaaggcgtggacttgctagggtttctcatcatcacattaaactgcaacgtgaccatg

gtgggaaagctctggttcgtcctcacgatgctgctgcggatgctggtgattgtcttggcg

gggcgacccgtctaccaggacgagcaggagaggtttgtctgcaacacgctgcagccggga

tgcgccaatgtttgctacgacgtcttctcccccgtgtctcacctgcggttctggctgatc

cagggcgtgtgcgtcctcctcccctccgccgtcttcagcgtctatgtcctgcaccgagga

gccacgctcgccgcgctgggcccccgccgctgccccgacccccgggagccggcctccggg

cagagacgctgcccgcggccattcggggagcgcggcggcctccaggtgcccgacttttcg

gccggctacatcatccacctcctcctccggaccctgctggaggcagccttcggggccttg

cactactttctctttggattcctggccccgaagaagttcccttgcacgcgccctccgtgc

acgggcgtggtggactgctacgtgtcgcggcccacagagaagtccctgctgatgctgttc

ctctgggcggtcagcgcgctgtcttttctgctgggcctcgccgacctggtctgcagcctg

cggcggcggatgcgcaggaggccgggaccccccacaagcccctccatccggaagcagagc

ggagcctcaggccacgcggagggacgccggactgacgaggagggtgggcgggaggaagag

ggggcaccggcgcccccgggtgcacgcgccggaggggagggggctggcagccccaggcgt

acatccagggtgtcagggcacacgaagattccggatgaggatgagagtgaggtgacatcc

tccgccagcgaaaagctgggcagacagccccggggcaggccccaccgagaggccgcccag

gaccccaggggctcaggatccgaggagcagccctcagcagcccccagccgcctggccgcg

cccccttcctgcagcagcctgcagccccctgacccgcctgccagctccagtggtgctccc

cacctgagagccaggaagtctgagtgggtgtga

>Hs-GJE1-NM_001358410 (Cx23) Splice sites

ATGTCTCTAAATTACATCAAAAACTTCTATGAAGGATGTGTTAAACCTCCAACTGTGATT

GGTCAATTCCACACCCTTTTCTTTGGATCGATCCGAATATTCTTCCTCGGGGTGCTAGGC

TTTGCAGTTTATGGGAATGAGGCCTTGCACTTCATTTGCGATCCAGACAAAAGAGAAGTA

AACCTCTTCTGTTACAATCAGTTCAGGCCAATCACTCCACAAGTAAGTTTTTCTGCATTA

CAACTAGTTATTGTCCTGGTTCCTGGAGCTCTTTTCCACCTTTATGCTGCATGTAAAAGC

ATCAATCAAGAATGCATTCTTCAAAAGCCTATCTACACTATAATTTATATACTCTCTGTT

TTATTAAGAATTAGTCTAGCGGCAATAGCATTCTGGCTTCAGATTTACCTCTTTGGTTTC

CAAGTAAAATCTCTTTACCTGTGTGATGCTAGATCTCTTGGGGAAAACATGATTATAAGA

TGCATGGTTCCAGAACACTTTGAAAAAACCATTTTTCTCATTGCAATAAATACATTTACA

ACAATTACAATTTTATTATTTGTTGCTGAGATTTTTGAGATCATATTTAGAAGATTATAC

TTTCCATTCAGACAATGA

# Supplementary Figure S2. Cattle (*Bos taurus*) *GJA9*.

The sequence was obtained from GenBank.

>Bt-GJA9-XM_010803798

atgggggactggaatttccttggaggcattctggaggaggttcacatccactccacggtg

attggaaagatgtggctcaccatcctgttcatatttcgaatgcttgttctgggtgtagca

gctgaagatgtctggaatgatgagcagtctggcttcatctgtaatacagaacaacccggc

tgcagaaatgtatgctatgatcgggccttccctatctccctcattagatactgggttttg

caggtgatctttgtgtcttcaccatccctggtctacatgggccatgctttgtaccgactg

agagttctggagaagcagaggcagatgaagaaggagcaactgagaggtgaactggaggag

gcagagcttgaaacgcctggggatcggaggcgactggaggaagaactgtgtcagctggag

caaaggaaagtaaataaagttccactcagaggaaccttgctttgcacttatgtgatacac

attttcactcgctctgtggttgaagttgggttcatgattggacagtatcttttatatgga

tttcacttagagcctctatttaaatgccacggccacccatgtccaaatacaattgattgt

tttgtctctagacccacagaaaagacaatattcctattattcatgcaatccatagcaact

gtttcacttttcttaaatgttttagaaattttccatctaggttttaaaaagattaaaaga

gggctttggggacaatataaattgaaggatgaacataatgaattttgtaccaagtcaaaa

caaaaccttgccaaatatcaaaacacatctgcaaactcactgaaacgactctcttctgta

cctgattatagtctgttagtggacaagcaaacacacacagcagggtatcctagtttaaat

ccctctgcatttcagacagaccctgataaccatattggaaatgatgacaaaggcattttg

gatgaacaggaaactctactttctgagatgtgcacatttagtacggcctgtggtcatctt

caaaatatcagctcaagtaataaagaagacactcataaactatctggaaaagaagttaat

ggtaaccagttgaggggaaaaagagaaactgatggcaaagacagcaaaagaaatcactac

tttaaaggtcactgttccagtccaggtgatgctatagagcttaacaaccacgtgggacag

tcaccccaaacagctttctctctgccagctaactgcacctggaaaccgaagtggcctcgg

gctacccggggtccctctgcagaaagtgaaaaccaggcattacctcctaaaggttacctc

aagggccagctcagggagagcacaaccagaatccttcctccttcacagggagactttcaa

ccacttgacattccagacactcctgattcttcgggagggctgtcctttgaatccaagttg

gtcagaacctgcagtaaccctactgcttgtactccaactcatttggtgtcactgacaaac

aacctcattggaaggcgggctcccacagaccttcagatctga

# Supplemenatry Figure S3. Mouse (*Mus musculus*) connexins.

The chronology of the sequences is according to the Greek nomenclature. Size nomenclature is indicated in parentheses. GenBank accession number is given for each entry.

>Mm-gja1-NM_010288

atgggtgactggagcgccttggggaagctgctggacaaggtccaagcctactccacggcc

ggagggaaggtgtggctgtcggtgctcttcattttcagaatcctgctcctggggacagcg

gttgagtcagcttggggtgatgaacagtctgcctttcgctgtaacactcaacaacccggt

tgtgaaaatgtctgctatgacaagtccttccccatctctcacgtgcgcttctgggtcctt

cagatcatattcgtgtctgtgcccacactcctgtacttggctcacgtgttctatgtgatg

agaaaggaagagaagctgaacaagaaagaagaggagctcaaagtggcgcagaccgacggg

gtcaacgtggagatgcacctgaagcagattgaaatcaagaagttcaagtatgggattgaa

gaacacggcaaggtgaagatgagaggtggcctgctgagaacctacatcatcagcatcctc

ttcaagtctgtcttcgaggtggccttcctgctgatccagtggtacatctatgggttcagc

ctgagtgcggtctacacctgcaagagagatccctgcccccaccaggtggactgcttcctc

tcacgtcccacggagaaaaccatcttcatcatcttcatgctggtggtgtccttggtgtct

ctcgctctgaatatcattgagctcttctatgtcttcttcaagggcgttaaggatcgcgtg

aagggaagaagcgatccttaccacgccaccaccggcccactgagcccatccaaagactgc

ggatctccaaaatatgcttacttcaatggctgctcctcaccaacggccccactctcacct

atgtctcctcctgggtacaagctggtcactggtgacagaaacaattcctcctgccgcaat

tacaacaagcaagccagcgagcaaaactgggcgaattacagcgcagagcaaaatcgaatg

gggcaggccggaagcaccatctccaactcccacgcccagccgtttgatttccctgacgac

agccaaaatgccaaaaaagttgctgctggacacgaactccagcccttagctatcgtggat

cagcgaccttccagcagagccagcagccgcgccagcagcagacctcggcctgatgacctg

gagatttaa

>Mm-gja3-NM_016975

atgggcgactggagcttcctggggcggctgctggagaacgcacaggagcactctacagtc

atcggcaaagtgtggctgaccgtgctgttcatcttccgcattctggtgttaggggcggca

gccgaggaggtgtggggcgacgagcaatcggacttcacctgcaacacacagcagccaggc

tgtgagaacgtctgctacgaccgcgctttccccatttcgcacatccgcttctgggcgctg

caaatcatcttcgtgtctacgcccaccctcatctatctgggccacgtgctacacatcgtg

cgcatggaggagaagaagaaagagcgggaggaagagctgctgaggagagacaaccctcag

cacggccgtggtcgcgagccaatgcgtacagggagcccgcgggaccctccactacgcgat

gaccgtggcaaggtgcgcatcgcaggtgcgctgctgcggacctacgtcttcaacatcatc

ttcaagacactcttcgaagtggggttcatcgcgggccagtactttctatacggcttccag

ctgcagccactttaccgctgcgaccgctggccctgccccaacactgtggactgtttcatc

tccaggcccacagagaagaccatctttgtcatcttcatgctggctgtggcctgtgcgtca

ctggtactcaacatgctggagatttaccacctgggctggaagaagctcaagcagggagtt

actaaccacttcaacccagatgcctcagaagccaggcacaagcccttggaccccctaccc

acggccaccagctctggcccgcccagcgtctccatcgggttcccaccttattacacacac

cctgcctgtcccacagtacaggcaaaggccatagggtttcctggggccccactatcacca

gcagacttcacagtggtgactctaaacgatgctcaaggcagaaaccacccagtcaaacac

tgcaatggccaccacctgacgacagagcagaactggaccaggcaagtggcagagcagcag

actccagccagcaagccctcttcagcagcatccagccctgatggccgcaaggggctcatt

gacagcagtggcagcagcttacaggagagtgccttggtagtgacgccagaggagggggaa

caggctttggccaccacagtggagatgcactcgccaccgttggtcctcctggacccagga

aggtccagcaagtccagcaacggacgtgccagaccaggtgacttggccatctag

>Mm-gja4-NM_008120

atgggcgactggggcttcctggagaagttgctagaccaggtccaggaacactcgaccgtg

gtgggcaagatctggttaacggtgctcttcatcttccgcatcctcatcctggggctggct

ggcgagtcggtgtggggcgacgagcagtctgattttgagtgtaacacagcccagccgggc

tgcaccaacgtctgctatgaccaggccttccccatctcccacatccgatactgggtgctg

cagttcctcttcgtcagcacacccaccctgatctacctgggccacgtcatctacctgtct

cggcgggaagagcggttgcggcagaaagagggagagctccgggcgctgccatccaaggac

ctacatgtagagcgggcactggctgccatcgaacatcagatggccaagatctcggtggca

gaggacggtcgtcttcggattcgtggggcgctcatgggtacctatgtggtcagcgtgctg

tgtaagagtgtgctggaggcaggcttcctctatggccagtggcgcctctatggctggacc

atggagccggtgtttgtgtgccagcgtgcgccctgcccccacatcgtggactgctatgtc

tctcgacccactgagaagactatcttcatcatcttcatgctggtggtaggagtcatctcc

ctggtgctcaacctgctggagctggttcacctgctgtgtcggtgtgtcagccgggagata

aaggcacgaagggaccacgacgcccgcccggcccagggcagtgcctcagacccttaccct

gaacaggttttcttctacctccccatgggcgagggaccctcttccccaccgtgtcccacc

tacaacgggctctcatccactgagcagaactgggccaacttgaccacagaggagagactg

acctcttccagacctcccccatttgtaaacacagctccccagggtggccgaaagtcccct

agccgccccaacagctctgcatccaagaagcagtatgtgtag

>Mm-gja5-NM_001271628

atgggtgactggagcttcctgggggagttcctggaggaggtccacaagcactccacagtc

atcggcaaggtctggctcactgtcctgttcattttccgcatgctggtcctgggcaccgct

gctgagtcctcctggggagatgagcaggccgacttccggtgcgataccattcagcctggt

tgccaaaatgtctgctatgaccaagccttccccatctcccacattcgttattgggtactg

cagatcatctttgtgtccacgccttctctagtgtacatgggccatgccatgcacactgtg

cgcatgcaggaaaagcagaaattgcgggatgctgagaaagctaaagaggcccaccgcact

ggtgcctatgagtacccagtagccgaaaaggccgagctgtcctgctggaaagaagtagat

gggaagattgtcctccagggcaccctactcaacacctatgtctgcaccattctgatccgc

accaccatggaggtggccttcatcgtaggccagtacctcctctatgggatcttcctggat

accctgcatgtctgccgcaggagtccctgtccccacccagtcaactgttatgtttcgagg

cccacggagaagaatgtcttcattgtctttatgatggctgtggctggactgtctctgttt

ctcagcctggctgaactctaccacctgggctggaagaagatccgacagcgctttggcaag

tcacggcagggtgtggacaagcaccagctgcctggccctcccaccagcctcgtccagagc

ctcactcctccccctgacttcaatcagtgcctaaagaacagctccggagagaaattcttc

agcgacttcagtaataacatgggctcccggaagaatccagacgctctggccactggggaa

gtgccaaaccaggagcagattccaggggaaggcttcatccacatgcactatagccagaag

ccagagtacgccagtggagcctctgcgggccaccgccttcctcagggctaccatagtgac

aaacggcgccttagtaaggccagcagcaaagcaaggtcagatgacctgtcagtgtga

>Mm-gja6-NM_001001496 (corresponds to human gja6p/gja1pX/cx43pX)

atgagtgattggagtgccttacaccagctcctagaaaaggttcaaccctactccacagct

ggaggaaaggtatggatcaaggttcttttcattttccgcatcctgctcctgggcactgct

atcgagtcggcttggagtgacgagcagtttgagttccattgcaacactcagcagcctggt

tgtgaaaatgtctgctatgaccatgccttcccaatctctcacgtgcgcctctgggtcctc

caggtcattttcgtatctgtgcctattctcttatacctggcacatgtgtactatgtggtt

cgacagaataagaagttgaacaagcaagaggaagaactggaagctgctcattttaatgag

gccagcgtggaaaggcacttggagacaattgcaggagagcagttcaagtgtggcagtgaa

gaacagagtaaggtgaaaatgagaggcagattgctgctaacctacatggccagcatcttc

ttcaagtctgtcttcgagatggccttcctcctgatccagtggtacatttatggatttact

ctgagtgccctttacatctgtgagcagtctccttgcccacgtcgggtggactgcttcctc

tctcgccccaccgagaaaaccatcttcatcctcttcatgtttgtggtgtcagtggtgtct

tttgtcttggatatcattgagctgttctatgtcttatttaaggctattaagaatcgtatg

agaaaagcggaggatgaggtttactgtgatgagctaccatgcccttcccatgtctcttca

tcaactgttctcaccaccatagattctagtgagcaggcggttccagtggaactttcttca

gtttgtatttaa

>Mm-gja8-NM_008123

atgggcgactggagtttcctgggaaacatcttggaagaggtgaatgagcactccactgtc

atcggcagagtctggctcacagtgctcttcatcttccgcatcctcatcctcgggacagca

gcggagtttgtgtggggcgatgagcaatctgattttgtatgcaacacccagcagccaggc

tgtgagaatgtctgctacgatgaggcctttcccatctcacacatccgcctctgggtgctg

cagatcatcttcgtctccactccatcgctgatgtacgtggggcacgcggtacaccacgtt

cgcatggaggagaagcgaaaggaccgtgaagctgaggagctctgtcagcagtcgcgcagc

aacgggggtgagagggtaccaatcgccccagaccaggccagcatccggaagagcagcagc

agtagcaaaggcaccaagaagttccggctggagggcacactgctaaggacctatgtctgc

cacatcatcttcaagaccctctttgaggtgggcttcatcgtgggccattacttcctgtat

ggtttccgcatcctgcccctctatcgctgcagccggtggccctgccccaatgtggtagac

tgctttgtatcccggcctactgagaagaccatcttcatcctcttcatgttatcagtcgct

tttgtgtcactcttcctcaacatcatggagatgagccacctgggcatgaaaggaatccgg

tctgccttcaagaggcctgtagagcaaccactgggggagattgctgagaagtccctccac

tccattgcagtttcctccatccagaaagccaagggctaccagcttctagaagaagagaag

atcgtatcacactatttccctttgacagaggttggaatggtggagaccagccctctttcg

gccaagccttttagtcagtttgaggagaagatcggcacaggacccctggcagatatgtca

cggagttaccaagaaaccctgccttcttatgctcaggtgggggtccaggaagtggagcgg

gaagagccgcctatagaagaggctgtggaaccggaagtgggagagaagaagcaagaagca

gagaaggtggccccagaagggcaggagacagttgcagtgccagacagggagagagtagag

acccctggagtggggaaggaggatgagaaagaagagctgcaagctgaaaaggtaaccaag

caagggctgtctgctgagaaggcaccctcactctgtccggagctgacaaccgatgacaat

cggcccttgagcaggctgagtaaagccagcagcagggccaggtcagatgatctcaccata

tga

>Mm-gja10-NM_010289

atgggagattggaatttactgggtggcatcctagaggaagtccactcccactccactata

gtggggaagatctggctgaccatcctcttcatcttccgaatgctggtacttggtgtcgct

gctgaggacgtctgggatgatgagcagtccgcctttgcctgcaacacccagcagcccggt

tgcaacaatatctgttacgatgatgctttccccatctctttgatcagattctgggttttg

cagatcatctttgtgtcttccccttctttggtgtatatgggccatgccctttatagactc

agggactttgagaagcagaggcagaagaagaagttataccttagagcccagatggagaat

ccagagctcgacctggaggagcaacaaagggtagataaagagctgaggagactcgaggag

cagaagaggattcataaagtccctctgaaaggatgtctgctgcgcacctatgtcttacac

atcctgaccagatcagtgctagaagtagggttcatgataggccaatatattctctatggg

tttcaaatgcaccccatttacaagtgcacccaagccccctgccccaattcagtggactgc

tttgtttccaggcccacagagaagaccattttcatgcttttcatgcacagcattgcagcc

atctccttgttactcaatatcctggaaatatttcatctcggcatcaggaaaatcatgagg

gcactcgatggcaaatccagcagtgggaacactgagaacgaaacaggccctccattccat

tcaacaaactactcagggacccagcagtgtatgatctgttcttctttacctgaaagaatc

tcactacttcaagccaacaataaacagcaagtcatccgagtcaatataccacggtctaaa

agcatgtggcaaattccacaccccaggcaacttgaggtagatgtatcctgtggcaaaaga

gactgggctgagaaaattgagagctgtgcacagctccacgtccacagcccctgcccacat

gaccgcagtgccagaattcagcaccctggacagcaaccgtgccattctgtctttggcccc

aagaatgcaatgtctcagtcttggttcggtacaatgacggcttctcaacaccgtccatca

tctgcgttagaaacctgggagcgatcccagggcccagaagcttcagggagatctctcaca

gatcgccagagtcacttccaaggcagtgacggcagtgcaagagagagtggggtttggaca

gacagattaggcccaggaagtcgcaaggccagctttctatcgaggctgatgtcagaaaag

ggacaacggcatagtgactcaggaagctcacggtctctgaatagttcctgcttggatttt

tcacacggagaaaatagcccatcacctctgccgtctgccactgggcacagagcatcgatg

gtaagtaaaagcagccatgttgattcacctcctcactcttctttcatcatacatgagaca

tatgtatatgtgtattaa

>Mm-gjb1-NM_008124

atgaactggacaggtctatacaccttgctcagtggcgtgaatcggcactctacagccatt

ggccgagtatggctgtctgtcatcttcatcttcagaatcatggtgctggtggtggctgct

gagagcgtgtggggggatgagaagtcctctttcatctgtaacaccctccagccgggctgc

aacagcgtctgctatgaccattttttccccatctcccacgtgcgcctatggtccctgcag

cttatcttggtttccaccccagctctcctcgtggcaatgcacgtagctcaccaacagcac

atagaaaagaaaatgctacggcttgaggggcatggggacccccttcacctggaagaggta

aagagacacaaggtgcacatctcagggacactgtggtggacctatgtcatcagtgtggtg

ttccggctgctgttcgaggctgtcttcatgtatgtcttctatctgctctaccccggctat

gccatggtgcggctggtcaagtgtgaagccttcccctgccccaacacagtggactgcttc

gtgtcccgccccaccgagaaaaccgtcttcactgtctttatgctcgcagcctccggcatc

tgcattatcctcaacgtggcggaggtggtgtacctcatcatccgggcctgtgcccgccgt

gctcagcgccgctccaatccgccctcccgcaagggctcgggcttcggccaccgcctctca

cctgaatacaagcagaatgagatcaacaagctgctgagcgagcaggatggctctctgaaa

gacatactgcgccgcagccctggcacaggggccgggctcgctgaaaagagcgaccgatgc

tcagcctgctga

>Mm-gjb2-NM_008125

atggattggggcacactccagagcatcctcgggggtgtcaacaaacactccaccagcatt

ggaaagatctggctcacggtcctcttcatcttccgcatcatgatcctcgtggtggctgca

aaggaggtgtggggagatgagcaagccgattttgtctgcaacacgctccagcctggctgc

aagaatgtatgctacgaccaccacttccccatctctcacatccggctctgggctctgcag

ctgatcatggtgtccacgccagccctcctggtagctatgcatgtggcctaccggagacat

gaaaagaaacggaagttcatgaagggagagataaagaacgagtttaaggacatcgaagag

atcaaaacccagaaggtccgtatcgaagggtccctgtggtggacctacaccaccagcatc

ttcttccgggtcatctttgaagccgtcttcatgtacgtcttttacatcatgtacaatggc

ttcttcatgcaacgtctggtgaaatgcaacgcttggccctgccccaatacagtggactgc

ttcatttccaggcccacagaaaagactgtcttcaccgtgtttatgatttctgtgtctgga

atttgcattctgctaaatatcacagagctgtgctatttgttcgttaggtattgctcagga

aagtccaaaagaccagtctaa

>Mm-gjb3-NM_001160012

atggactggaagaagctccaagacctattgagtggcgtgaaccagtactccacggcattt

gggcgcatctggctgtcagtagtgttcgtcttccgggtgctggtgtatgtggtggctgcc

gagcgtgtgtggggtgacgagcaaaaagactttgactgtaacaccaggcaacccggctgt

accaacgtgtgctatgacaacttcttccccatctccaacatccgactctgggccctgcag

ctcatcttcgtcacgtgtccgtctatgctggtcatcctgcatgtagcctaccgcgaggag

cgggaacggaagcatcgccagaagcacggggagcaatgcgccaaactgtacagccacccg

ggcaagaagcatggcggcctgtggtggacctacttgtttagcctcatcttcaagctcatc

atcgaattggtcttcctgtacgttctccacacgctctggcatggcttcaccatgccgcgt

ctggtacagtgcgccagcatagtaccctgccccaacaccgtggattgctacatcgctcgg

cccacggagaagaaggtctttacctacttcatggtaggcgcttctgccgtctgcattatt

ctcaccatctgtgagatctgctacctcatcttccacaggatcatgcgaggcataagcaag

ggcaagtccacaaagagcatcagctccccgaagtcctccagccgggcctccacctgtcgc

tgtcaccacaagctgctggagagtggcgatccggaagcagacccagccagtgaaaagctg

caggcttcagcgcccagcctgacccccatttga

>Mm-gjb4-NM_008127

atgaactggggatttctccagggaatcctgagtggtgtgaacaagtactccacggcactg

ggccgcatctggctgtctgtggtcttcatcttccgggtgctggtgtatgtggtggcggca

gaggaggtgtgggacgacgatcaaaaggatttcatctgcaataccaagcagccaggctgc

cccaacgtctgctatgatgagttcttccccgtgtcccacgtgcgcctctgggccctgcag

ctcatcctggtcacctgtccttccctgttagtggtcatgcatgtggcctatcgtgaagag

cgagaaaggaaacatcgcctcaaacatgggcccaatgccccagccctgtacagcaacctg

agcaagaagaggggtggcctgtggtggacatacctgctgagtctcatcttcaaggctgct

gtggactctggctttctctacatcttccattgcatttacaaggactatgacatgccccga

gtggtagcttgctctgtgactccctgcccccacactgtggactgttacatcgcccgaccc

acagagaagaaggtcttcacctacttcatggtagtcacggcagccatttgtattctactc

aacctcagtgaggtcgtctacctggtgggcaagagatgcatggaggtcttccgtccccgg

cgccggaaagcttccaggaggcaccaactgccagatacgtgcccaccgtatgtgatctcc

aaaggaggtcaccctcaagatgagagcgtgatcctaacaaaggccgggatggccacggtg

gatgcaggtgtgtatccatga

>Mm-gjb5-NM_010291

atgaactggagtgtttttgagggactcctgagcggagtcaacaagtactccacagccttt

ggtcgcatctggctgtctctggtcttcgtcttccgtgtgctggtgtacctggtgacagct

gagcgcgtgtggggagacgaccagaaggattttgactgcaacaccaggcaaccgggctgt

accaatgtctgctacgatgagttcttccccgtgtctcacgtgcgcctctgggctctgcag

ctcatcctggtcacctgcccctctttgcttgtggtcatgcatgtggcctatcgaaaggct

cgagagaagaagtaccaggaaaagattggtgaaggttacctttacccgaatcccggcaag

aagcggggtggactctggtggacatacgtcttcagcctctcgttcaaggccaccatagac

attatcttcctctacctcttccacgcattctatcccagatataccctcccttctatggtc

aagtgccatgcggagccgtgtcccaacacagtggactgcttcattgccaagccctccgag

aaaaacatcttcattgtcttcatggtggtcacggccgtcatctgcatcctgcttaacctt

gtggagctgatctacctagtgattaagcggtgttctgagtgtgcgcagctgaggagacca

cccactgcacatgcaaagaatgacccaaactgggccaactctcctagcaaagagaaggac

ttcctctcatgcgacctcatctttctgggctcggacgctcacccgcctctgttaccagac

cgccctcgagcccacgtgaagaaaaccattctgtgaa

>Mm-gjb6-NM_001010937

atggactgggggaccctgcacaccgtcatcggtggcgtgaacaagcactctaccagcata

gggaaggtgtggatcacggtcatctttattttccgagtcatgatcctagtggtggctgcc

caggaagtgtggggtgatgagcaggaggactttgtctgcaacactctgcagccagggtgc

aagaacgtctgctatgaccatttcttcccggtgtctcacatccggctctgggccctgcag

ctgatctttgtgtctaccccagccctgttggtggccatgcacgtggcctactacagacat

gaaactgcccgaaagtttatacgtggggagaagagaaacgagtttaaagacctggaggac

atcaaacggcagaaggtgcgcattgagggctccctgtggtggacgtacaccagcagcatt

ttcttccgcatcatcttcgaagccgccttcatgtatgtgttctacttcctctacaatggg

taccacctaccctgggtactgaaatgtggcattgacccctgccccaatctcgtggactgc

ttcatttcgaggccaactgagaaaacggtgttcactgtttttatgatttccgcatccgtg

atttgcatgctgctcaatgtggccgagttgtgttacctgctgcttaaattgtgctttagg

agatccaaaagaacacaggcgcagagaaaccaccccaaccatgccctgaaagagagcaag

cagaatgaaatgaatgagctgatctcagatagtggccagaatgcaatcacaagtttccca

agttaa

>Mm-gjc1-NM_008122

atgagttggagcttcctgactcgcctgctagaggagatccacaaccattcgacatttgta

gggaagatctggctcactgtgctgattgtctttcgaattgtcctaactgctgtaggagga

gagtccatctactatgatgaacaaagcaaatttgtgtgcaacacagagcagccgggctgt

gagaatgtctgctatgatgcctttgccccgctctcccacgtgcgcttctgggtattccag

atcatcctggttgcaactccctctgtgatgtacctgggatatgctattcataagattgcc

aaaatggagcatggcgaggcagacaagaaggcagctcggagcaaaccctatgccatgcgt

tggaaacagcaccgggctctggaagaaacggaagaggaccatgaagaggatcctatgatg

tatccagagatggagttagaaagcgaaaaagaaaataaagagcagagccaaccaaaacct

aagcatgatggccgacgacgaattcgagaggatgggctcatgaaaatctatgtgttgcag

ctgctggccaggactgtgtttgaggtgggctttctaatagggcagtatttcctgtatggc

ttccaagtccacccattttatgtgtgcagcagacttccttgccctcataagatagactgc

tttatttctagacccactgaaaagaccatcttccttctgataatgtatggtgtcacaggc

ctctgcctattgcttaacatttgggagatgcttcacttagggtttgggacaattcgagac

tcactaaacagtaaaaggagggaacttgatgatccgggtgcttataattatcctttcact

tggaacacaccctctgctccccctggctataacattgctgtcaaaccagatcagatccag

tacactgagctgtccaatgctaagattgcctacaagcaaaacaaagccaatattgcccag

gaacagcagtacggcagccacgaggaacacctcccggctgatctggagactctgcagcgg

gagatcagaatggctcaggaacgcttggacctagcaatccaggcctaccatcaccaaaac

aacccccatggtcctcgggaaaagaaggccaaagtggggtccaaatctgggtccaacaaa

agcagtattagtagcaaatcaggggatgggaagacctccgtctggatttaat

>Mm-gjc2-NM_080454

atgagctggagcttcctgacgcggctgctggaggagatccacaatcattccaccttcgtg

ggcaaagtttggctcactgtgctggtggtcttccgcattgtgctgacagccgtcggtggt

gagtccatctattcagatgagcaatccaagttcacctgcaacacgcggcaaccgggttgt

gacaacgtctgctatgacgcctttgcgcccctgtctcatgtgcgcttctgggtcttccag

atagtggtcatctccacaccttctgtcatgtacctgggctatgcagtccaccgcttggcg

cgggcctcggaacaggagcgcagacgcgctctccgacgtcgccctggcacccggcgcttg

cccagggcgcagctgccaccgccgccacctggctggccggacaccaccgatctgggagag

gcggagcccatattggctctagaggaggatgaggacgaggagccgggggcgcccgagggc

cccggagaagacacggaggaggagcgagcggaggatgtggctgccaaagggggcggaggt

gatggcaagacggtggtcactcctggcccggccgggcagcacgatgggcggcggcgcatc

cagagggagggcctgatgcgtgtgtacgtggctcagctggtggttagggcggccttcgag

gtggcctttctggtgggccagtacctactgtacggcttcgaggtgccacccttctttgcc

tgcagccgccagccttgcccccacgtagtggattgcttcgtgtcgcggccgaccgagaag

acggtcttcttgctggtcatgtacgtggttagctgtctatgcttgttgctcaacctctgt

gagatggcgcacctgggtctcggcagtgcgcaggatgctgtgcgcggccgtcggggagcc

tcagcggcggggcctggccccacgccgcgcccaccgccctgcgctttcccggccgcggcc

gccggcctggcttgccctccagactacagcctggtggtgcgtgcagctgagcgcgcgcga

gcgcacggccagaacttggcgaacctagcgctgcaggcgttgcgcgatggggcggcggtg

gcggcggtttccgcggaccgcgacagtccgccgtgcgctgggctcaatgcaacctctcgg

ggggcacccagggtgggcggcctagcttccggaaccggcagcgccacgtcggggggcacc

gttggggagcagagccggccgggagctcaggaacaactggccactaagcccagggctggc

tctgaaaagggcagtacaggcagcagagacggcaaggccaccgtgtggatctga

>Mm-gjc3-NM_080450

atgtgcggcaggttcctgagacagctattggctcaggagagccagcactccacccctgtg

gggcgcttccttcttcccatgctcatgggattccgtctcctgatcttggtttccagtgga

cctggggttttcggcaatgatgagaatgaattcatatgtcatttagggcagccaggctgc

aagaccatttgctatgatgtcttccgccctctctctccattgcgcttctgggccttccaa

gtcattctgatggctgtacccagtgccatttatgtggctttcactctgtatcatgtgatt

ggatactgggaggtaccaggaaaggaaaacaaggagcaagagacccagattagcaaaggg

gatcatagcaaggatgtctcaggggctaaaagcctcaagcttctctgggcctatgtggca

caccttggggtacggctggcccttgagggagcagctctaggtgttcagtacaatctgtat

ggtttcaagatgtccagcacttttatatgtcgtgaggatccttgtattggcagcacaacc

tgtttccagtctcacccctctgagaagaccatcttcctcaacatcatgtttgggatcagc

ggggcctgtttcttatttattttcttggagcttgcgcttttgggtttagggaggttttgg

aggatatacaagcacaaactttccttcttaaagaagttgccaacttcagagagctctgta

agatccaaggacacaaccgatgaattgtcagtggtggaggcaaaagagccattttga

>Mm-gjd2-NM_010290 Splice site

atgggggaatggaccatcttggagaggctgctggaagccgcggtgcagcagcactccact

atgattgggaggatcctgttgactgtggtggtgatcttccggatactcattgtggccatt

gtaggggagacggtgtacgatgatgagcagaccatgtttgtgtgcaacaccctacagccc

ggctgtaaccaggcctgctatgaccgcgcctttcccatctcccatatacgttactgggtc

ttccagatcataatggtgtgcacccccagtctctgttttatcacctattctgtgcaccaa

tccgccaagcagcgagaacgccggtactctactgtcttcctagccctggacagagaccct

gctgagtctatagggggacctggaggaactgggggtgggggcagcggagggagcaaacga

gaagataagaagttgcaaaatgccattgtcaatggggtgctgcagaacacagagaccacc

agtaaggagacagaaccagattgcttagaggttaaagagctgactccacatccatctggg

ctgcgcacagcagcaaggtccaagctccgaagacaggaaggtatctcccgcttctacatc

atccaagtggtgtttcgaaatgctctggagattgggtttctggtgggccagtactttcta

tatggcttcagtgttccagggttgtatgagtgcaaccgttacccctgcatcaaggaggta

gaatgttatgtgtctagacctaccgagaagacagtctttctggtgttcatgtttgctgtg

agcggcatttgtgtggtgctcaatctggctgaacttaaccatctgggatggcggaagatc

aaactggctgtccggggagcccaggccaagaggaagtcagtctatgagatacgtaacaaa

gatctgcctcgagtcagtgttcccaatttcggcaggactcagtccagtgactctgcctat

gtgtga

>Mm-gjd3-NM_178596

atgggggagtgggcgttcctaggctccctgctggacgcggtgcagctacagtcgccgctc

gtgggtcgtctctggctggtgatcatgctgatcttccgcatcctggtgctggccacggtg

ggaggtgcggtgttcgaggacgagcaggaggagttcgtgtgtaacacgttgcagcccggc

tgtcgccagacctgctacgatcgcgccttcccggtgtcccactaccgcttctggctcttc

cacatcctgctgctgtcggcgccgccggtgctgttcgtcatctactccatgcaccaggcc

agcaaggaggcgggtggtgcgcagctggccccgccgtgcgcgcgcgggcgtgccgaggcg

ccgtgctccccgtgcgccctgcgcgctcgccgcgcgcgccgctgctacctgctgagcgtg

gctctgcgcctgctcgccgagctggctttcctgggcggccaggcgctgctctacggcttc

cgcgtggacccgcactacgcgtgcgccgggccaccttgtccgcacacggtcgactgtttc

gtgagccggcccaccgagaagaccgtcttcgtggtcttctacttcgccgtcgggctgctg

tcggcgctgctcagcgtggcggagctgggtcacctgctctggaagggtcgccagcgcgcc

aagctgctcccgccgccgccgccgtcgccctctttgccatcgcagcgcggggaccccgac

cctttcggcccgccagcctacgcgcaccgctcaccggcaggcgacagcgagggcgaaggc

ggcagcggccacagcaaagcgtcgctggctaccgtgcgccaggacctggccatctag

>Mm-gjd4-NM_153086 Splice site

atggagaagttgaacttgttgggattcctcatcatcaccttaaactgtaacgtgaccatc

atgggcatgatctggctgatcgtggaggtcttgctgaggatgctagtggtggtcttggca

gggtcacctatctatgaggatgaacaagagaggtttatttgcaacacactgcaaccagga

tgtgccaacgtttgctacgacctcttttccccagtgtcaccgctgcgattctggctagtg

cagagcctggccttgcttctgccttcggtggtctttggcacttacaccctacaccgcggt

gcgaagctggctgcagtggggggagcctgcaggccccaggtgcccgacctgtctactgcc

tacctggtgcacctactgctgcgcatgctgctggaggccgggctggccttcctgcactac

tttctctttggcttttctgtgcccgcccgcgtgtcttgctcgcatgtaccctgctcaggg

gctgtggactgctacgtgtcgcggcccacggagaagtcactcctgatactattcttttgg

gcagtgagtgcgctatccttcctgctcagcttggctgacctgctttggatcctgccgagg

agaaagacactgaggaccacgcagtgggtgaatggagaggctagaccagtctgtgaagta

cctgcacctcccccttgcctcttacaaaacccccagggctatcttagccaaggtcaggtg

gaccaagaggacagacaggaggaacaagttgtgcctgagttcccctgcatgtggacagca

gggcagagtgacaacagcaatgttggtcaggcctgtgtgtcggggctgctggaacattca

gaccaagatgctagtgaggccacttcctcagctggtgacaggctaacagtggctcacaca

gcacatgagctcagattccacagagagacttcactggacctggggggcaaaaacacccag

gcagatgaactctccttggctacccagagccacctggccagacacagttcagccagcaag

cctcaagctccatgccggctgaccacctcaggcagtgctccccatttgagaaccaaaaaa

tctgagtgggtgtga

>Mm-gje1-NM_029722 Splice sites

atgtctctaaattacatcaagaacttctatgaaggatgtgttaagcctccaactgtgatc

ggccagttccacactctcttcttcggctcagtgcggatgttcttcctcggagtgctgggc

tttgctgtctacgggaatgaggcgttgcacttcagctgtgacccagacaagcgagagata

Aacctgttctgttacaatcagttccggccaataactccccaagtgttctgggcattgcag

ctagtgattgtcctgcttcctggagctattttccacctgtatgctgcatgcaaaagcatc

aatcaagactgcattcttcagaagcccgtgtacactgtgatttacgtcctctcggtcttg

ttaagaatcagcctggaggtgttcgcattctggcttcagattcacctcttcggcttccaa

gtgaagccgatatacttgtgtgatactgaatctcttggtaaaaaaccaaatattctaaaa

tgcatggttccagagcactttgaaaagactattttcctcattgcaatgtacacatttact

gtgatcacgatggtattatgtgttgctgaggtttttgagatcatatttagaagatcatgt

tttctctttaaacgatga

# Supplementary Figure S4. Black flying fox (*Pteropus alecto*) *GJD5*.

The sequence was obtained from GenBank.

>Pa-GJD5-XM_006925175-GJD2like

atgagtgactggtcgttcctgggcaggctcctgacacaagtgcaaaaccattccaccgtg

gttggcaaggtgtggctcaccgtccttctggtcttccgcattctgctggtcaccctggtg

ggagatgcagtctatggggatgagcagtccaagttcacctgcaatactctgcagcctggc

tgcacaaatgtctgctatgaccgcttctcacctgtctcccacttccgcttctgggttttt

cagattgtgctggtagccactccctctatcttctatgtcatctatgtcctgcatcagata

gcaagggaggaaagagtagatatggagagggagtacctgctggatacattgcgaaagcta

gcatctgggggagccctgcagagacccaagctggggctcttggcgtcttctcacttccta

gagggacagctgctggtgggagagagggttctccccaaatgccttggtgctgcagccagg

aatccaggcctgcggtcacaaagagtcttggccatctacattgcccatgtggtgctgcgg

gccttcatggaactggctttcctggtggggcaatactatctgtttgggtttgatgttcca

tacttgtttcactgccactcctatccctgtcctactagtactgactgctttgtatccagg

gccacagagaagatgattttcctgaacttcatgtttggggtcggggtgggctgcttcctc

ctgaacctggtggagttgcactacctgggctgggtcttcacctaccggttcctctttgca

gcctgcaccagttgctgccacttctgtgggcagcctgcccatcctccactgctctactct

gatgaggacagtactgggctccaggtgtattcctgcctttacctcactagttgccacgcc

tacaccgtcaagggcttcctcccaccctttgcttgttcatccttctggggagctctgccc

tggcaggcttcagagcccaactaa

# Supplementary Figure S5. Opossum (*Monodelphis domestica*) connexins.

The chronology of the sequences is according to the Greek nomenclature. Size nomenclature is indicated in parentheses. GenBank accession number is given for each entry.

>Md-GJA1-XM_007484502

ATGGGGGATTGGAGTGCCTTAGGCAAACTCCTTGACAAAGTACAGGCTTATTCTACTGCT

GGAGGGAAGGTGTGGCTCTCCGTCCTCTTCATTTTCCGAATCTTGCTATTGGGAACCGCG

GTGGAATCAGCTTGGGGTGATGAACAGTCTGCCTTTCGATGTAACACTCAGCAGCCAGGT

TGTGAAAATGTATGCTATGACAAATCCTTCCCAATCTCTCACGTGCGATTCTGGGTCCTG

CAAATCATCTTTGTGTCTGTGCCAACCCTCCTGTACCTGGCACACGTGTTCTATGTGATG

CGTAAAGAAGAGAAGCTGAACAAGAAAGAAGAAGAGCTCAAAGTCGCCCAAACGGACGGT

GCCAATGTGGATATGCACTTGAAACAAATTGAAATCAAGAAATTCAAATATGGAATTGAA

GAACATGGCAAAGTGAAAATGCGTGGAGGGTTACTGCGTACCTACATCATCAGCATCCTT

TTTAAGTCTGTGTTCGAGGTGGCCTTCCTCCTGATTCAGTGGTACATCTATGGCTTCAGC

TTGAGCGCGGTCTATACTTGCAAGCGGGATCCCTGCCCTCATCAAGTGGACTGCTTCCTC

TCCCGTCCCACCGAGAAAACGATCTTCATTATCTTCATGCTGGTTGTGTCCTTGGTGTCT

CTTGCCTTAAATATCATTGAGCTCTTCTATGTGTTCTTCAAGGGTGTCAAGGATCGCGTG

AAGGGAAAAAGCGACCCTTACCACACTACAGCCGGCCCACTGAGTCCCAGCAAAGATTGC

GGTTCTCCTAAATATGCTTATTTCAATGGCTGTTCCTCCCCAACCGCCCCCTTGTCACCC

ATGTCTCCTCCAGGGTACAAGCTCGTGACCGGAGATCGAAATAATTCTTCTTGCCGTAAC

TACAACAAGCAAGCCAGTGAGCAAAACTGGGCCAACTATAGTGCTGAACAGAATAGAATG

GGGCAGGCTGGAAGCACCATCTCCAATTCCCATGCTCAGCCTTTTGATTTTCCAGATGAT

AACCAGAATTCAAAAAAACTAGCCGCCGGCCATGAGCTACAGCCACTTGCCATTGTGGAC

CAAAGGCCTTCCAGTAGAGCCAGCAGTAGGGCCAGCAGCCGACCTCGACCCGATGACTTG

GAGATCTAA

>Md-GJA3-XM_007495190

ATGGGTGACTGGAGCTTTCTGGGGAGATTATTAGAGAATGCGCAAGAACACTCTACCGTG

ATTGGCAAAGTTTGGCTGACTGTTCTGTTCATCTTCAGAATCCTGGTGCTAGGTGCCGCC

GCAGAAGAAGTCTGGGGAGATGAACAGTCCGATTTTACGTGTAACACTCAGCAACCAGGT

TGTGAGAATGTCTGCTATGACAAGGCTTTTCCTATTTCCCACATCCGCTTCTGGGTCCTG

CAGATCATTTTCGTCTCTACCCCCACCCTCATTTATCTGGGCCATGTGCTGCACATTGTA

CGCATGGAGGAAAAAAAGAAGGAAAAAGAAGATCTCCTTAAGAAAGACAACCTGCACCAG

GGGGTAGAGCTCAGTGGCCCCAACAGCCATCGAGAACCACTCAGCAAAAAGGAGAGCGCC

AGGATGGAAAAGAAAGAGAGACCCCCAATCCGAGATGATCGAGGCAAAGTCCGGATAGCT

GGTGCCCTGCTCCGCACCTACGTCTTTAACATCATCTTCAAGACTCTGTTTGAAGTGGGC

TTCATTGTGGGGCAATATTTCCTCTACGGGTTTGAGTTAAAGCCACTCTACCGATGTGAC

CGCTGGCCCTGCCCCAACACAGTGGATTGCTTTATCTCCAGACCCACAGAAAAGACCATA

TTCATTATTTTCATGTTAGTGGTGGCTTGTGTGTCTCTCTTGCTCAACATGCTGGAGATC

TATCACCTGGGCTGGAAAAAGCTTAAACAGGGTATGACCAACCATTACAATAGCCCAGAT

TCTCCAGAAGCCAAAGTCCTGCCCTCCAAATGTAGTAGCATTGGCCCACTTCTGCTCTCT

CCCCATTCTGCCCCTCCTGCCGTTGGATTCCCACCATACTATACTCAGTCTGCCTCTTCC

CTAGGACAGGCAACCACTACAGGTTATCCTGGGGCTCCTCCACAACCCACAGAATTCAGA

ATGGTAACCCTCCCTGAGGAGCGCAGTAAGGCAGCCCCAGCCAAATATTATGGTGGCAAC

CACCACCACATCTTAATGACTGAGCAGAACTGGGCCAACCAGGAGGCAGAGCAACAGACT

TTTGAAAGGAAGTCTTCCCCAAGCAATACCACCCCTGGCACTCCCACCACCCCAAGCAGT

GTTCGGCAGCTCCTCCAAGAGCGAGGAGACAGTGGTGGGGGGAGCAAAGTGCCCTTGCTG

GTGATTAATGGGAGTAGCAGCAGTTTAGGGGCCACTAAATCAGAGGTAACCTCTGAAGGG

GAGAAACAGCCAGGTACCACCACAGTTGAGATGCATGCACCGCCATTGCTTCTCGTTGAT

TCAAGACGGTTAAGCAAGGCTAGCAAGGCTAGCAGTGGCAGAGCTCGATCGGATGACTTA

GCCATCTAA

>Md-GJA4-XM_007492764

ATGGGTGACTGGGGCTTCCTAGAGAAACTGCTAGACCAAGTCCAGGAGCACTCCACAGTG

ATAGGCAAGATCTGGCTGACCGTACTGTTCATCTTCCGGATCCTCATCCTGGGGCTGGCC

GGTGAGTCTGTCTGGGGAGACGAGCAATCAGACTTCGAATGTAACACGGCCCAGCCGGGC

TGCACCAACGTGTGCTATGACCAGGCCTTCCCCATCTCCCACATTCGCTACTGGGTCCTG

CAGTTCCTCTTTGTCAGCACGCCCACCCTGGTCTACCTGGGCCACGTCATCTACCTGTCC

CGCCGAGAAGAGAAGCTGCGGCAGAAGGAGAGCGAGCTGCGGGCGCTTCAAGCCAAGGAC

CCACGAGTGGAGCAGGCGCTGGCCACGGTGGAGCGGCAGATGGCAAAGATCTCTGTGGCC

GAGGATGGGCATCTTCGCATCAGGGGAGCCCTGATGGGCACCTACGTGGCCAGCGTGGTC

TGTAAGAGCCTGCTGGAGGCTGGCTTCCTCTATGGACAGTGGCGCCTGTACGGCTGGATG

ATGGAGCCCGTGTACGTGTGCCGACGCTTCCCCTGCCCACACCTGGTGGACTGCTTTGTG

TCCCGCCCCACAGAAAAGACCATCTTCATCATCTTCATGCTGGTCGTGGGGATGATTTCC

CTAGTACTCAACCTCCTGGAGCTGGCACATCTTGGCTTTCGGTGTATGGGGCACAAGCTG

AGGGCCCGGCAGACCCGGGCTCGGCTGGGGCCTTACTCTGCTGCCCTGGGGGATGGGGCT

GGTATGGGTAGTTCTGGGGACCCCTATTCGGATCGGATGTTCTTCTACCTCCCCATGAAC

GAGACACCTACGTCGCCTCCCTGCCCCTCTTACAACAAGCTGTCTAGTGAACAGAACTGG

GCTAACCTGAATACAGAAGAGAGCCTGGCCACCCAGAAGCAGGGCATGCTCCCTGGGCCG

GGGCCCCTGCTGACTCATCCCGAGGGCCCCTACCTGGCTCAGCCCCCCCAGAACGGAGAC

AATGCCCTGAGTCGTCCTAGTAGCTCAGCTTCCAAGAAACAGTATGTGTAA

>Md-GJA5-XM_007485330

ATGGGTGACTGGAGCTTCCTCGGGGAGTTCCTGGAAGAGGTCCACAAGCATTCCACAGTG

ATTGGCAAGGTTTGGCTGACTGTCCTCTTCATCTTCCGTATGCTAGTGCTGGGCACAGCG

GCGGAGTCCTCGTGGGGGGACGAGCAAGCCGACTTCCAGTGTGACACGCTACAGCCTGGT

TGTGAGAACGTCTGCTATGACCAAGCCTTCCCCATCTCCCACATCCGATACTGGGTGCTG

CAGATCATCTTCGTCTCCACTCCATCCCTGGTGTACATGGGCCACGCGATGCACACCGTG

CGCATGGAGGAGAAGAGGAAGCTGAAGGAGGCTGAGAGGGCCAAGGATTCCAAGGGTGCA

GACACCTATGAGTATCAGGCAGAGAAGGCTGAGCTTTCCTGCCGGGAAGAGCTGAGTGGA

AGGATTATCCTTCAAGGCACCCTTCTCAACACCTATGTCTGCAGCATTCTCATTCGCACG

GCCATGGAGGTGGCTTTCATCGTGGGGCAGTACCTCCTCTACGGGGTCTTCCTGGAGACC

CTATACATCTGTCGCCGAAAACCCTGCCCTCACCCGGTCAACTGCTATGTGTCTCGGCCC

ACTGAGAAGAATGTTTTTATCGTATTCATGCTGGCTGTGGGGGGTCTGTCCCTCTGCCTC

AGTCTGGCTGAACTCTACCATCTGGGCTGGAAGAAAGCCAAGCGGCACTTCAACCAGAAC

TGCCCAGGCAGGGTGGAGGCCAGCCTGCCCACAGCAGGGGTGGCCCAGAACTGCACGCCA

CCCCCAGACTTCAATGAGTGTGTAGAGGGCAGCCCCAATCAGAAGTTCTACAGCAGTCAT

TTCAGCAATAATATTGCCTCCCGGCAGAATACAGACAACTTGGCCACAGAGCAGGTCCAA

GGCCAGGAGGAGGCTGTAGGGAGGGCCTTTTTGCACATGCAGTATGCAGAGGGGCCCGAG

GTGGCTAACGGGATGTCCAATGCACATCGGTTCCCCCATAGTTACCATGCTGACAAGCGC

CGCCTGAGCAAGGCTAGCAGCAAAGCCAGGTCAGATGACTTGTCGGTGTGA

>Md-GJA8-XM_001363421

ATGGGTGATTGGAGTTTCCTGGGGAATATCTTGGAGGAGGTAAATGAGCACTCCACGGTC

ATCGGCAGGGTCTGGCTCACCGTCCTCTTCATCTTCCGGATTCTGATCCTGGGCACAGCA

GCAGAATTTGTGTGGGGTGACGAGCAGTCGGACTTTGTATGTAACACCCAACAACCAGGT

TGTGAGAATGTCTGCTACGATGAGGCCTTCCCCATCTCCCACATCCGTCTCTGGGTCTTA

CAGATCATCTTCGTGTCCACTCCATCCCTGGTGTATGTGGGCCATGCTGTGCACCATGTA

CGTATGGAAGAAAAGCGGAAGGAGAGGGAAGCTGAGGAGGTATGCCAGCAGTCTGGGGGC

AATGGGGAGAGGCTGCCATTAGCCCTGGATCAAGGAAGTACCAAGAAGAGCAGCAACAGT

ACCAAAGGTACCAAAAAGTTCCGACTGGAAGGAACCCTCTTGAGGACCTACATCTGCCAC

ATCATCTTCAAGACTCTCTTCGAGGTGGGCTTCATCGTGGGCCATTACTTCTTGTATGGC

TTTCGGATCCTGCCCTTGTATCAGTGCAGCCGTTGGCCTTGCCCCAATGTGGTGGACTGC

TTCGTGTCTAGGCCCACCGAGAAGACCATTTTTATCCTTTTCATGCTCTCGGTGGCCTCT

GTGTCCCTCTTCCTCAATATCATGGAAATCAGCCACCTGGGCCTGAAGAGAATTCGATCT

GCTTTCAATAGACCTGCTGAGCAGCCACTGGGGGAGATCCCTGAGAAATCCCTCCACTCC

ATTGCTGTCTCGTCCATCCAAAAGGCCAAGGGCTACCAGCTCCTAGAAGAAGAGAAAATC

GTGTCCCACTACTTCCCCCTGACCGAGGTTGGGGTGGTGGAGACAAGACCACTTGCTGCC

ACCCCTTTCAGCCGTTTTGAAGAGAAGATCAGCACTGGGCCTCTGGGAGATCTGTCCCGG

GCTTACACGGAAACGCTGCCCTCCTATGCTCAGGTGGGAGAACAAGAAGTAGAGGCAGAA

CGTGAAGAAGAGGAGGCAGAGGCCCCGCCAGAGGAGGAAGAGAAGAGGCAGGAATCAGAG

ACAGGGATCCCGGAGGGGCAGGAAGCCCCTCCTGTAGGGCAGGAGGAAGAGAAGGAGAAA

GCAGGGACCCCCGTTGAAATGAAGGCAGGAGACAAGCAAGAGCTGCCAGTGGAGAAGATA

CCCCTGTGCCCAGAGCTGGCAGCGGATGACACCCGACCCCTCAGCCGGCTAAGCAAAGCC

AGCAGTCGAGCAAGGTCAGATGATTTGACTGTATGA

>Md-GJA10-XM_007484323

ATGGGAGACTGGAATTTGCTGGGCAGCATTCTAGAGGAAGTCCACTTCCATTCAACCTTG

GTGGGGAAGATCTGGCTGACCATGCTATTCATATTCCGTATGCTGGTGCTTGGAGTGGCA

GCAGAGGATGTTTGGGATGATGAACAGTCAGCATTTATCTGTAACACCCAACAGCCTGGC

TGCAGCAACATTTGTTATGATGATGCTTTCCCCATTTCTTTGATCAGGTACTGGGTTTTA

CAGATTATCTTTGTATCTTCTCCCTCTCTTGTATACATGGGCCATGCACTTTATAGACTC

AGGGCCTTTGAAAAGGAGAGGCAGAGGAAGAAAGTACAACTTCGTGCCCTGCTGGAAGAA

CCTGAGCATGACCAAGAGGAGCACCAAAGAATTGAGAAGGAACTGAGGAGATTAGAGGAA

CAGAGGAAGGTACACAAGGCACCCCTGAAAGGATGCTTGCTACGTACTTATATATTGCAT

ATCTTGACCAGATCTGTATTGGAAGTAGGGTTTATGACAGGGCAGTATATTCTCTATGGG

TTTCAAATGCACCCCCTTTACAAATGCAGTCGATTTCCTTGCCCCAATTCAGTGGACTGC

TTTGTGTCCAGGCCCACAGAGAAGACCATTTTCATGTTGTTCATGCACACCATAGCAGCT

ATCTCCTTGTTCTTAAACATTTTGGAAATATCTCACTTGGGAATCAGGAAAATCACCAAG

GCATTATATGGTGGGTCTAGTAGTGAGAGTGAAAGGGAACTGTATCACTCAAAGAAAAAT

TCAGTGACCCAGCCCTGTGTAACTCACTCTTTATTACATGAGAGCATCCCTTTGTCCCAG

CCTCCTAACCACTTTTGGCTAGAGAAACAAGTAATTGGAACTGGTAATACAGAGTATAAA

ACAGCGTGGCAACCCAACCATCACAACCAGACTGAGGGAGCACCTCCACCTGGCAAGAAG

AACTGGTCCAAGAGGGATCGGCATCATAGAGTGTTTCAATTTAGCTCTCATTGTCCTCAG

GATCTTGATTATGGTGTCCAGCACTTACAACACTGCAAACATCAGAACCATGGACAGCAC

CCACGTTCTTCCTTCAGTAAAAGGGGCATTCTTTCCAAATCGCATCAGGCCAATCTTACA

AATTGCTCTTCTTGCACACTAAGTCCAGGGGAACAGCCCTACAGTCTTTGGAGCCCAAGC

AGGTCCTCAATAGACTTGACTGCTCACTGCAAGACAAGTGATAACATCAAGTCAGAGTGT

TTTGACTCGGCAGAGAAAGGGTCTCACCCAGGTAGCCGTAAAGCCAGTTTCCTATCTAGG

CTTTTGTTTGAGAAGGGCCAGTTATCCAAAGCTTCAGAAAGCTCTGATTCCCAGCATAGC

TCTATCTTGGACTTCCAGCACTGGGGTGAAGATGACCATGCCTCCAGCTCTCCGCCTCCA

GGCACTGGGCGCAGAATGTCAA**TGAA**AATGCTCTTAAAACTTTCATCTATCATGAAAAAA

TAA

>Md-GJB1-XM_007507588

ATGAACTGGACAGGCCTGTACGCCTTACTCAGTGGTGTAAACCGGCACTCTACCGCCATC

GGGCGCGTCTGGCTCTCAGTCATCTTCATCTTTCGCATCATGGTGCTGGTGGTGGCCGCT

GAGAGCGTGTGGGGAGATGAGAAGTCCTCCTTCATCTGCAACACCATGCAGCCTGGCTGT

AACAGTGTGTGCTATGACCACTTCTTTCCCATCTCCCACGTGCGCCTGTGGGCCCTGCAG

CTCATCCTGGTGTCCACACCGGCCCTGCTTGTGGCCATGCATGTGGCCCACCAGCAGCAT

ATGGAGAAGAAGCTGTTGCGACTTGAGGGCCACGGGGACCCCCTGAGCCTGGAAGAGGTC

AAACGGCACAAGGTGCACATCTCAGGCACACTGTGGTGGACCTATGTCATCAGTGTGCTC

TTTCGCCTGCTCTTTGAGGCTGTCTTCATGTATGTCTTCTACCTGCTCTACCCAGGCTAC

GCCATGGTGCGCCTGGTCAAGTGTGACAGCTACCCTTGCCCCAATGTGGTGGACTGCTTC

GTGTCACGGCCCACTGAGAAGACCGTCTTCACCGTCTTCATGCTGGCTGCCTCAGGCATC

TGCATTGTGCTCAACGTGGCAGAGCTGGTGTACCTCGTTGTCCGTGCCTGTGCCAGGCGG

GCCCAGCACCGCTCCAACCCACCCTCACGCAAGGGTTCGGGATTTGGCCACCGGCTGTCC

CACGAGTGCAAACAGAACGAGATCAACAAGCTGCTCAGTGACCAAGATGGCTCCCTCAAA

GACATACTGCGGCGCAGCCCGGGCACTGGAGCTGGCCTCACTGAGAAGAGTGACCGCTGC

TCTGCCTGTTGA

>Md-GJB2-XM_007495197

ATGGACTGGAGTACTCTACAGACTATTTTGGGGGGTGTCAACAAACACTCCACCAGCATA

GGCAAAATCTGGCTCACTGTCCTCTTCATTTTCCGCATTATGATCCTGGTCGTGGCTGCT

AAGGAGGTGTGGGGAGACGAGCAGGCTGATTTTGTCTGCAACACTCTCCAGCCCGGATGT

AAAAATGTGTGCTACGACCACTTTTTCCCCATCTCGCACATCCGCCTCTGGGCTCTGCAG

CTGATCTTTGTGTCGACCCCCGCGCTCTTGGTGGCCATGCACATTGCTTACCGGAGGCAC

GAGAAAAAGAGAAAGTTCATCAAGGGAGAGATAAAGTCTGAATACAAAGACATAGAAGAA

ATCAAGAAACAAAAGGTTCGCATTGAAGGAGCCTTGTGGTGGACCTACACAAGCAGCATT

TTCTTCCGAGTCGTCTTTGAAGCGGTCTTCATGTACGTGTTCTATTTTATGTACAACGGA

TTCTCCATGACTCGAATGGTGAAATGTAATGCTTGGCCCTGTCCCAATACTGTGGACTGC

TTTATTTCCCGACCCACTGAAAAGACAGTGTTCACTGTGTTCATGATTTCGGTGTCTGGA

ATTTGCATACTGCTAAATGTCATTGAATTGTCTTATCTGCTGATAAGATATTGTTCTGGG

AAGTCCAAGAAGCCAGTTTAA

>Md-GJB3-XM_016422482

ATGGACTGGAAGACTCTGCAGTCACTCCTGAGTGGTGTCAACAAGTACTCCACAGCGTTC

GGCCGGATCTGGCTGTCGGTGGTGTTCGTCTTTCGCTTGCTGGTGTACGTGGTGGCGGCG

GAGCGCGTGTGGGGGGATGAGCAGAAGGATTTTGACTGCAATACACGCCAGCCCGGCTGC

ACCAATGTCTGCTACGATCACTTCTTCCCCATCTCCAACATCCGCCTGTGGGCCCTGCAG

CTCATCTTCGTCACCTGCCCCTCGCTGCTGGTCATCATGCACGTGGCCTACCGGGAGGAG

CGGGAGCGGCGGCACCGGGAAAAGCACGGGGACAAATGCGCCCGGCTCTACGAAAACGCC

AGCAAGAAGCATGGCGGGCTCTGGTGGACCTACCTGCTGAGCCTCTTCTTCAAGTTCATC

GTGGAAGTCGTCTTCCTCTACATTCTGCACACGCTGTGGTACGGTTTCTTCATGCCCCGC

CTGGTGCAGTGTGCCGGCGTGAGCCCCTGCCCCAACACCGTGGACTGCTACATCGCCAGG

CCCACCGAGAAGAAGATCTTCACCTACTTCATGGTGGGGGCCTCAGCCATCTGCATTGTC

CTGACCATCTCTGAGATCTGCTACCTCATCTCTAAGAGACTATCCCGGAGCTTCTGCCAA

AAGAAGCACAAGCGCTCTGCCCTGCACTCTCCCTCCTCCAGCAGGGCATCCACCTGCCGC

TGCCACCATCTGCTGACCCACCATGGGGGCGAAGAGGGCATGGTCCAAGGCAAGGGGGCA

GAGGCCCTTCGGGCCTCCGCACCCAACCTGACTCTCATCTGA

>Md-GJB4-XM_016422483

ATGAACTGGGCATTCCTCCAGGGCCTCCTGAGTGGGGTCAACAAGTACTCCACGGTTCTT

GGCCGAGTCTGGCTGTCAGTGGTACTGATATTTCGAGTACTGGTATACGTAGTGGCAGCA

GAGGAGGTGTGGGACGATGAACAAAAGGACTTCGACTGCAACACTCGACAGCCAGGTTGT

GCGAATGTTTGCTACGACCACGTCTTCCCCATTTCCCACGTCCGCTTGTGGGCCCTTCAG

CTCATTCTGGTCACATGTCCCTCGCTGCTCGTCGTCATGCACGTAGCCTATCGAGAGGAG

CGGGAGCGGAGGCACCGGATGAAGCATGGCCCCCAGGCCAGGCCCCTCTATGGCAACCCA

GGGAAAAAACGTGGAGGTCTCTGGTGGACCTACCTGCTAAGCCTTATATTCAAGGCTGGC

GTTGATGCCACCTTCCTGTACATCTTCCATCGCCTCTATAATAACTATGACATGCCCCGT

GTGGTGCACTGCTCCGTGGACCCCTGCCCCAATGTGGTAGATTGCTTCATCTCCCGGCCC

ACGGAAAAGAAGGTCTTTTCCTACTTCATGGTGGCCACAGCTGTCATTTGCATCCTGCTC

AATCTAGGGGAAGTGTCCTACCTGATCTGTAAGAGAGCCCAGGAGCTCCTAGGGCCACAG

AACTCGAAACAGCCTCGGCGGCACCATCGGAGGCATGGCCACCATGGCCCCTTGGGGACC

CTGCAGGATGCCTGCCCCCCTTATGCTCCTGCCCAGGCTTTGTCCCAAGGTGACCCCACC

AAGGAGGCTACATTGCCTTGCTAA

>Md-GJB5-XM_007492760

ATGAACTGGGGAATCTTCGAGGATCTGCTGAGCGGCGTCAATAAGTATTCTACAGCTTTC

GGCCGCATCTGGCTCTCCCTGGTCTTCATCTTCCGCGTGCTGGTCTACCTAGTGACCTCC

GAGAAGGTGTGGAGCGATGACCACAAAGACTTCGACTGCAACACGCGCCAGCCGGGCTGC

TCCAACGTCTGCTACGACCACTTCTTCCCCATCTCCCACGTCCGCCTGTGGGCGCTGCAG

CTCATCCTGGTCACGTGCCCCTCGTTGCTCGTCATCATGCACGTGGCGTACCGGCAGGCC

CGGGAGCTGAGACACCTGGAGCAGGTGGGCGAAGGAGGCGGGCGCCTCTATCCAAACGTA

GGCAAGAAGCGCGGAGGGCTCTGGTGGACCTACGTCTTCAGTCTGGTCTTCAAGGCCAGC

GTGGATTCAATCTTCCTCTACGCGTTCTACCGCCTCTATCAAAACTACCTGCTCCCGCAC

GTGGTCTTCTGCAGCGAGGACCCCTGCCCCCATACCGTGGACTGCTTCATCTCCAAGCCC

ACGGAGAAGAACATTTTCACACTCTTCATGGTGGCTACCGCCATCGTCTGCATCTTGCTC

AACCTGGTAGAACTGGGTTACTTGGTCAGCAAGAGGTGCTGGGAGTGCCGGGAGGTTGGG

AGAATGGATACCAAGAAGGATTTGCTGTCCGGGGGCGATCTCATCTTCCTGGGTACCGAC

CCCAAGCCACCGCTGCTGCCTTTTTCTCCTGACTCCCCCCGAGACCAGGTGAAGAAAACC

ATGGTATAA

>Md-GJB6-XM_007495198

ATGGACTGGAGTACACTGCATACTTTCATTGGAGGCGTAAATAAACACTCCACCAGCATA

GGGAAGGTTTGGATCACCGTCCTCTTCATTTTTCGAGTCATGATCCTTGTCGTAGCTGCT

CAAGAAGTATGGGGAGATGAGCAAGAAGATTTTGTCTGTAACACACTGCAGCCAGGATGC

AAAAATGTGTGCTATGACCACTTCTTCCCTGTTTCTCATATCAGACTTTGGGCTCTTCAA

CTAATCTTTGTCTCCACTCCAGCACTTCTGGTAGCCATGCATGTAACTTACAATAGACAT

GAGAAGGAAAGACAGTTTAGGAAAGGGGAGAAAGGGATTGAATTCAAAGACTTAGAAGAA

ATTAAAAAACAAAGGGTACGAATTGAGGGGTCTTTGTGGTGGACTTACACTAGCAGTATT

TTCTTTAGGATTATCTTTGAAGCCTCCTTTATGTATGTGTTTTACTTTCTTTACAATGGC

TATAACCTGCCCTGGGTGGTGAAATGCAGTATTGATCCTTGTCCCAATATTGTGGACTGC

TTTATTTCAAGACCCACTGAAAAGTCTGTTTTTACCATTTTCATGATTGCTGCATCTGTG

ATTTGCATGCTGTTAAATGTGGCTGAATTATGTTACTTGCTCATGAAACTGTGCTTCAGA

AGATCCAGAAGAGCACAGGTTCAAAGAAATCACCCTAATCATGCCATAAAAGAAAGCAAA

CAGAATGAAATGAATGAGCTGATTTCAGACAGTGGACAAAATGCAATCACAGGTTTCCCA

AGTTAA

>Md-GJB7-XM_007484297

ATGACTTGGATGCTCCTCAGAGATCTCCTAAGTGGAGTAAATAAATATTCAACAGGAATT

GGTCGAATCTGGCTGGCTGTCATCTTTATGTTCCGTTTGCTGGTCTACATGGTAGCTGCA

GAACATGTTTGGAAAGATGAACAGAAGGAATTTGAATGTAACATTAGGCAGCCTGGTTGT

GAAAATGTCTGTTTTGACTACTTCTTCCCCATCTCCCAGGCTAGGCTTTGGGCCTTGCAG

CTGATCATGGTCTCTACTCCTTCTCTTCTGGTTGCTTTGCATGTGGCCTACCGTTTGGGC

CGTGAAAAAAGGCACAATAAGAAATTTTATGTTAGTCCAGGTAGCAAGGATGGGGGCCTA

TGGTACACTTATATCATTAGCCTTGTTGTCAAAACTGGTTTTGAAATTGGCTTCCTGGCT

TTGTTTTACAAGTTATATGATGGATTTAGAGTACCCTACCTTGTGAAATGTGATATAAGA

CCTTGCCCTAACACTGTGGATTGCTTTATCTCCAAACCTACTGAGAAGAAAATCTTCCTT

TACTTCTTGTTAGTCACATCATGTCTGTGCATTGTTTTGAATATCGTTGAGTTAGGTTAT

CTGGTTCTCAAGAGTTTTGTAAAGTGCTGCCTTCAACGATATGCTCAGAATTTCAAATCT

TCAGCTTATAAGTGTCATAACCTTGATTATGCCATGTGCAATGAGATTGTCCCGAAACTG

CACCAGAAGCACAACTCTGACTGTTCCAGAAGCATTCCTCAAAAACTTGATAGAAGTGAT

TTGCAAGAATGGTGA

>Md-GJC1-XM_007482452

ATGAGTTGGAGCTTCCTGACTCGACTGCTAGAGGAGATCCACAACCATTCCACATTTGTG

GGGAAGATTTGGCTCACCATATTGATAGTTTTCCGGATAGTCCTCACTGCTGTGGGTGGG

GAGTCTATCTACTATGACGAGCAGAGCAAATTTGTATGCAACACAGAACAGCCCGGCTGC

GAGAATGTCTGCTATGACGCTTTTGCCCCACTGTCCCATGTCCGCTTCTGGGTATTCCAG

ATCATCCTTGTGGCCACCCCCTCGGTGATGTATCTTGGCTATGCCATCCACAAGATTGCC

AAGATGGAACATGGTGAGGCAGACAAGAAGGCATCAAGAAGCAAGCCCTACGCAATGCGC

TGGAAGCAGCACCGGGCCCTGGAAGAAACTGAGGAGGACCACGAGGAGGACCCCATGATG

TATCCGGAGATGGAGTTGGAGAGTGAGAAAGAGAACAAGGAGCAGAGTCAGCCTAAACCC

AAGCACGATGGTCGGCGGCGGATTCGGGAAGATGGCCTTATGAAAATCTACGTGCTACAG

TTGCTAGCAAGGACTTTGTTTGAGGTGGGCTTCCTGGTGGGGCAGTATCTTCTCTATGGC

TTCCAGGTCCGCCCATCTTATGTGTGCCGCAGAATCCCCTGCCCTCATGAAATAGACTGC

TTCATTTCTAGGCCCACCGAAAAGACCATCTTCCTGCTAATAATGTACGGTGTGACGGGC

CTCTGTCTGATCCTCAACATTTGGGAGATGCTCCATTTGGGGTTTGGGACTATCCGTGAC

TCACTAAATAGCAAAAAGAGAGAACTGGAAGATTCGGGTGCTTATAACTATCCTTTCACT

TGGAATACTCCATCCGCTCCACCTGGCTATAACATTGTGGTCAAACCAGATCAGATCCAG

TACACCGAACTGTCCAATGCAAAGATCGCCTACAAGCAGAACAAGGCCAACATTGCTCAG

GAGCAGCAATACGGGAGCAATGAGGAGAACCTTCCCGCAGACCTCGAGATGCTGCAGCGG

GAAATCAAAGTGGCCCAGGAACGCCTGGATCTGGCCATCCAGGCCTACAACCACCAGAAC

AACCGCCACGGCCCCCGGGAAAAAAAGTCCAAAGCTGGGTCCAAAGCTGGGTCCAACAAA

AGCAGTGCCAGTAGCAAATCTGGGGATGGGAAGAATTCTGTCTGGATTTAA

>Md-GJC2-XM_007499765

ATGAGCTGGAGCTTTCTGACGAGGCTGTTGGAGGAGATCCACAACCATTCCACCTTTGTG

GGCAAGGTTTGGCTGACCGTGCTGATCGTCTTCCGGATAGTGCTGACCGCAGTAGGGGGG

GAGTCCATCTACTCGGACGAGCAGAGCAAATTCACCTGTAACACCCGCCAGCCGGGCTGC

GACAACGTGTGCTATGACGCCTTTGCCCCCCTCTCCCACGTTCGCTTCTGGGTCTTCCAG

ATCGTGGTCATCTCTACGCCGTCCGTCATGTACCTTGGCTATGCCATCCATCGTCTGGCG

AGAGCCTCGGAGGAGGAGCGGCGCCGGGCCAGGCGCGGGCGCCAGGGTGGCCGCGGGGGC

CGCAGGCGTCCCCAGAGGAGGAGGCTGCCGCCGATGGCCCACCCGGGCTGGGCAGACACC

CCGAATGGCGGAGAGGAGGAGCCCATGATTGGCCTTGGGGCTGGCATGGGGGAAGAAGAG

GAGGCTGGCGATGGGAGCAGGGAGGACAGAGAGCAAGAAGAAGAAGAAAAGGAGGCCTTG

GCGGGGGAGAAAGGCAAGGGGGCTCCGGAGGCCGGGCCTGCCAACCAGAAGCATGATGGG

AGGCGAAGGATCCAGCAGGAGGGGCTGATGAAGATCTACGTGTTCCAGCTGTTGGCTCGG

GCCTCCTTTGAGATCTGCTTCCTGGTCGGGCAGTACCTCCTGTATGGCTTCGAGGTGCAA

CCTGTCTTCCGCTGCAGCCGAGATCCGTGCCCTCACACCGTTGACTGCTTTGTGTCTCGG

CCCACAGAGAAGACTGTCTTCCTCCTGGTGATGTACGTGGTCAGCTGCCTGTGCCTCATC

CTGAATCTCTGTGAGATGGCCCACCTGGGCCTGGGCAGCTTGCAGGATGCGGTGCGGAGC

CGCAGGGTCGGGGGCCGCGACCAGGGTTCGGCCGGCTATCCTTCCCCCCCACCCGCGCCC

CGGCAGCTACCCCATGGCTACCTGTACGCTCGCAACATCTCCTGCCCTCCTGAGTACAAC

ATGGTGGTCAGGAATGAGAGGGCAGCGGCAGCCGGGCGCCTCGTGCCTGGAGGCCTCCTG

GCCCACGAGCAGAACCTGGCCAATGGCGTCCTGCAGGAGCTGCAAGAGCTTCAGGGCCCT

GGCTCAGAGGAGAATCCTCCACCCATGGATCTGGCTGCTGCCTTCCGAGCTACTCATCAT

CGGGCTGGCACCCAGGACCCTGCCCCTGGGGTCAGGAGCAACGGCTTTCCTGCTTATACA

GCTCAGGTCGGGCCACTACCTTCAAGGACTGACAGCCCAGCCTCGGCAGGCACCATTGTG

GAGCAGAATCATTCCGATGGTGCCCCAGGGCAGCAAGGGGCCAAAGCCAAATCCAACTCG

GAGAAAGGCAGCAGCGTCAGCAGCAAAGATGGCAAGACATCTGTATGGATATGA

>Md-GJC3.1-XM_007499115-GJC1like

ATGAGCTGGGCGTTCCTGACGAGATTGCTGGAGGCTGTGACCCAGCACTCCACTCTGGTG

GGGAAGCTCTGGCTCTCCGTGCTGGTCGTTTTCCGACTAGTGCTCCTGGCCGTGGGTGGG

GAGGCCATTTACCGGGACGAGCTCAGTGGATTCGTGTGTAACACTCGGCAGCCTGGATGC

CAAAACGTCTGCTACGACACCTTCGCACCCCTCTCGCATGTGCGCTTCTGGGTATTCCAG

ATCATTCTCGTCACCGCGCCCACCGTGTTCTATTTGGGCTATGCCGTGCACCATCTGTCC

CGGCGCCAGATGACCCAGAAGCAAGAGAAGGAAGAGGAGGAAGGCGAGAAGGAGCCTATG

ATCAAAGAGAAGAAGCCTCAGATGTCCCCTGACGATGGGGCCCATGACGGTCGCAGGAGG

ATCCGCAGGGATGGGCTCCTAGGGGCCTATGTTGCGCAGTTGCTGGTGCGGACTGCCTTA

GAGGTGGCCTTCCTAGCGGGCCAGTATCTGCTCTTTGGCCTGAAAGTGCCTCCCGACTAT

GACTGCAAGCAGAATCCTTGCCCTCATGTCGTCGTCTGCTATATATCTCGTCCCACGGAG

AAGACCATCTTCCTGCTGGTCATGTATGCAGTCAGTGGCCTCTGCCTCCTGCTCAATCTG

ATCGAGTTGCTGCACCTGGGCCTGGGGAGCGTGAAAAATGGTGGAAGCCGCCCTGACCCA

GCGCCCCATTCCAAGAGTCTCAAGCTGGAGCTGCAGCAAATGCAGAAACAACTGCACCTG

GTCCAGAAGCAAATGGACATGGGACTGCATCTGGGCACACCTTTGGTCACAGTGCCTACC

TGTTCAGGGCAGCCTCCCACCTACAATCTCTCTACTCAGCAAAATCGGCACAACCAAGCC

CAGAAAGAGACCTGGCTCTCCAAGACAGGTACGTAG

>Md-GJC3.2-XM_001370819-GJC2like

ATGAGCTGGGCGTTCCTGACCAAGTTGCTGGAGGCTGTGACCCAGCACTCCACCCTGGTG

GGGAAGCTCTGGCTCTCCGTGCTGGTCGTTTTCCGACTAGTGCTCCTGGCCGTGGGTGGG

GAGGCCATTTACCGGGACGAACTCAGTGGCTTCTCCTGCAACACAGCACAACCTGGCTGC

CTAAATGTTTGCTACGATACCTTCGCGCCTCTCTCCCACGTGCGCTTCTGGGTATTCCAG

ATCGTCCTGGTCACTGCGCCCACCGTGTTTTATCTGGGCTATGCCGTGCACCATCTGTCC

CGGCGCCAGATGACCCAGAAGCTAGAGGAGGAGGAGTCTCTGATCCCGGGAAAGAAGTCC

TGTAAGGATGGGGCCCACGATGGTCACAGGAGGATCCGCAGGGATGGGCTCCTAGGGGCC

TATGTCGCCCAGTTGCTGGTGCGGACTGCCTTAGAGGTGGCCTTCCTAGCGGGCCAGTAT

CTGCTCTTTGGCCTGAAAGTGCCTCCCGACTATGACTGCAAGCAGAATCCTTGCCCTCAT

GTCGTCGTTTGCTATATATCTCGTCCCACGGAGAAGACCATCTTCCTGCTGGTCATGTAT

GCAGTCAGTGGCCTCTGCCTCCTGCTCAATCTGATCGAGTTGCTGCACCTGGGCCTGGGG

AGCATAAGAAATGGTAGAAGCCTGAAAAATGGTggacatcaccctgccccatctcctcat

tccaaggctggcaagctagaacaaatgcagagacagttgaatctggtccaggagcaaatg

ggcatggtattcaatcagggccctcttttggctacagaacctacctctacagggcagtct

ccagactacagtttatttgctcagcaaaactggcataaccaagcccagaaatag

>Md-GJD2-XM_003340035 Splice site

atgggggaatggaccatcttagagaggctgctggaagccgccgtacagcaacattccact

atgattgggaggatcctgctgacagtggtggtgatcttccggatccttatcgtggccata

gtgggtgagacagtgtatgatgatgagcagaccatgtttgtatgcaataccctgcagcca

ggctgcaaccaagcctgctatgaccgagctttccctatctctcatatccgctactgggtc

ttcnaaatcatcatggtgtgcactcctagcctctgcttcatcacctactcagttcatcaa

tctgccaagcgagccgggggtgggagcagtggtgggggcaagcggttagacaataagaag

ctgcaaaatgccattgtaaatggagtactacaaaatacagagaatactagcaaggagact

gagccagactgtctagaggttaaggagttggccccacacccatctgggttacgtacagca

gcccgttctaagctccgtcggcaagagggcatctcccgcttctacattattcaggttgtt

ttccgtaatgccctagaaattgggttcctggtaggccaatacttcctttatggcttcagt

gtccctggactgtatgagtgtgaccgctatccctgtatcaaggaggtagaatgctatgtt

tcccggcccacagagaagacagtcttcctcgtgttcatgttcgctgtgagtggcatttgt

gttgtcctcaatctggctgaactcaatcacctgggctggcgcaagattaagctggctgtg

cgtggtgcacaagccaagaggaagtcagtctatgagatccgcaacaaggacctgcctcgg

gtcagtgtaccgaattttggcaggactcagtcaagtgactctgcctatgtgtga

>Md-GJD3-XM_001365802

ATGGGGGAATGGGCCTTCCTGGGCTCGCTGCTGGACTCGGTGCAGCTGCAGTCCCCCCTG

GTGGGCCGCCTGTGGCTGGTGGTCATGCTGATTTTCCGGATCCTGGTGTTGGCCACGGTC

GGGGGGGCCGTGTTCGAGGATGAGCAGGAGGAGTTCGTGTGTAATACGCTGCAGCCTGGG

TGTCGTCAGGCCTGCTATGACCGGGCCTTCCCCATCTCCCATTACCGCTTTTGGCTTTTC

CACATCTTGCTGCTCTCTGCTCCTCCCGTGCTGTTCATCATCTACTCCATGCACCAGGCC

AGCAAAGGCCCCAGACCCCAAGGAGATGGGATGAAGGAGCAGGAGGAGTGGAGCGGCAGC

CTGGGCCAGCGCCTGGCAGGGCCCGGGGCTCGCCGCTGCTACCTGCTGAGCGTGGCCCTG

CGTCTGCTGGCTGAGCTGGGCTTCCTGGTAGGGCAGACCCTGCTGTACGGCTTCCGGGTG

GCCGCCCGTTTTCCCTGCACCCAGACCCCGTGCCCGCACGTGGTGGACTGCTTTGTCAGC

CGCCCAACGGAGAAGACGGTCTTTGTGATTTTCTACTTTGCTGTGGGGCTTCTCTCTACC

CTCCTCAGCGTGGTCGAGCTGGGCCATCTCTTCTGGAAGGGGCGCCGAAGTCAGAACAAG

GGTGCCCTTTGGCTAGGCAAGGTGGTCACAGGAGAGAAAGACAACCACTGCAACCAGGAG

CAGGAAGAGGCCCGGAAGCTTCTCCTGCCACTCTCCCCACCAAAAAGGGCCCCTCCACCC

AGCCCCATGCAGGGCGCCCCACCTGCCTATGCCCACAAGTTGCCACAGGGTGGCAGCGAG

GGCAGCAGCAGCAGGAGCAAGTGCTCGCTGTCCACAGCCAGAGAGGACCTCTCCATCTAA

>Md-GJD4-XM_0013474328 Splice site

ATGGAACGTTGGGATTTGCTGGGGTTTCTGATCATCACATTGAACTGCAATGTGACAATT

GTGGGGAAGATCTGGCTCATCATCATGATAATGCTGAGGATGGTGGTAATTATTTTGGCA

GGCTATCCAATCTACCAAGATGAACAAGAGAGATTTATTTGCAATACTTTGCAACCAGGA

TGCTCCAATGTATGCTATGACATCTTCTCTCCTGTATCTCACTTCCGATTTTGGCTGATC

CAGAACGTATCTGTTCTCCTACCTTATGCCATGTTCAGTGTTTATGTCTTTCACAAAGGA

GCCCTACGTGCTGCAATGGGGGCCCGCCAACCAGATTGCTGTAAAGGAAGGAATATTCTC

CCTGACCAGAGAGGAGTGAAGGGGTTTGGCTATTCTCATCTAAATTTCCCTGACTTTTCC

ACTGCATATATAATACAACTTCTTCTGAGGATCCTGACCGAAGCTGCTTTTGGTGGTGCC

CAATACTATCTCTTTGGATTCTGGGTTCCAAAGCAATTCTCCTGCTACCATTCTCCTTGT

ACAAGTGTGGTGGATTGCTATATCTCCCGGCCCACAGAGAAATCAATAATGATGCTTTTT

ATTTGGGGAGTCAGTGCTCTATCCTTTCTTCTAAGTTTTGTTGACCTAATTTGTTGCATG

CAGAGATGGCTGAGACGGAAGCATTTGGCTAAACGGATGATGAAAAATGTTTGTGTCAGT

GAAGAGCATGGCTCCCCATACATCCCTCCTGGTCAAGCCAAGCACTGTTTGACCTCAGAG

GTAAGGCAGGAACCGCCACAGCTGGTGAAAAGGTTCAGTCAGATTAGTGAGAATGCTGGG

TGGCCTGAGTCTAGAGGGGAAGAAGATATATCTCTTCATCCTATGGTGTGGCCCAAAGAT

GTAGCTTCCAGGTCAAACCTTAATAGCCCAGGTCATAAGTCTTGCTCATCTGGAAGAATG

ACTTTTCCAGATGAAGATGGCAGCGAAGTGATGTCCAATGGCAGTGAACAGCAAGGGATA

GCTTCTAAAGAGATGCAAAGCAGGCCTTTCAAAGAGGTTCCTAGGTCCAAGGACAGCTCC

CAGCTTGGAGAGTTTGCCTCTGCCCCTCGAAGCCGACTTGGAGGACATTATTCATCCAGT

GAGCTGAAACCTTCTGCCTCTCAGGTGAGCTGTGGCAGCCCTAGCTACCTAAGGGCCAAA

AAGTCTGAATGGGTATGA

>Md-GJD5-XM_001376506-GJD2like

ATGGGTGACTGGTCATTTCTGGGCCGGCTTCTGAATGAAGTCCAGAACCATTCCACTGTC

ATCGGTAAGATCTGGCTCACCGCCCTCCTCATCTTTCGGATTCTCCTGGTCACATTGGTG

GGCGATGCCATCTACGGAGATGAGCAGTCTAAGTTCACCTGTAACACCCTCCAGCCGGGC

TGCACCAACGTCTGCTACAATAGTTTTGCTCCTATCTCCCACCTTCGCTTCTGGATCTTC

CAGATTGTTCTGGTGGCCACACCATCCATTTTCTACATCGTGTGTGTGTTGCATCAGGTG

GCCTTGGAGGAGAGGATGGATGTGGAGAGGGACCGTCTGCTGGAGCTGTGGCGAAGACTG

GTGCCAGCCACTGGGGAAGTCCTTCCTGGAGTGGGGTCTGGGGTCCTAGTGCCCTCTAGC

TCCTTTGAAGGCCACAGTCTGGATGAGGAGGAGGTCCTTCCAAAGCACCTCCAGTCCACC

TCTCAGGACCCCATCTACCTGGCCAACCGGGCATTGATCATTTACATCGCCCACGTGGTG

CTGAGGGCCTTCCTGGAGTTGGGATTCCTAGTGGGGCAATACTACCTGTTTGGGTTTGAT

GTGCCTCATTTGTATCGCTGTGAAACCTACCCATGTCCCACCAAGACGGACTGCTTTGTC

TCCAGGGCGACAGAGAAAATGATCTTTCTGAATTTTATGTTCGGCGTTGGGCTTGGCTGT

TTTCTTCTGAACTTGGCAGAATTGCACTACCTGGGATGGCTCTTTACCTTCCGGATGCTC

TTCAAGGCTTGTGTCAATTGCTGCCAATATCTAGGCAAAGCACCCCCATCCCACAGGTCC

CGACTGCTGCCTCTTCTGGACTCAAGTCAGGAGAGGATCCTTCTGGAGGCCTCCTTGCTG

CCTGCGTGGGGGTCAGGGCATCTCAGGGCCCAGCACACTGTGATACCTCTGACAATCGAT

GCAGCCCAGGCCACTTTGGACCACAAATCTCAACAGGAAAAGTCCTCCAGGATGCCCCCC

AGCAAGAAATCTTGGCTCTGA

>Md-GJE1-XM_001380882 Splice sites

atgtctctaaattacatcaaaaacttctacgaaggatgtctcagacctcctacagtgatc

ggtcagttccacacgcttttctttggctcagttcgaatgttcttccttggagtgttgggc

tttgcagtctatgggaatgaagccttgcattttagctgtgatccagacaagagggaaatc

aatctcttctgttacaaccagttcaggccaataactccacaggtattctgggcattacag

ctagtgactgtactggttcctggagctgcttttcatctttatgctgcatgtaaaagcatc

gatcaagaaagcatacttcagagacccatctacactgtcttttatatcctctctgcctta

ttaagaataattcttgaggtgatagccttttggctacaaagtcatctttttggttttcaa

gtaaaatctctttatcattgtgatgctagtttgcttgaaaaaagattgggtatcataaga

tgcctggtcccggaacattttgaaaaaaccatatttctcattgccatgtacacatttact

gcagtcacagtggcattgggatttgctgaagtttttgagatcttatgtagaagattaggg

tttttaagtggatag

# Supplementary Figure S6. Tasmanian devil (*Sarcophilus harrisii*) *GJC3.*

The sequence is collected from GenBank.

>Sh-GJC3-XM_003761914-GJC1like

atgagctgggcgttcctgacgaagttgctggaagctgtgacccagcattctaccttggtg

gggaaactgtggttgtccgtgctggttgtgtttcgcctagtgctgctggcggtgggtgga

gaggccatttaccgggacgagttaagtggcttctcctgtaatacagcgcaaccgggttgc

caaaatgtttgctatgacgccttcgcacccctctcccacgttcgcttctgggtgttccag

atcatcctggtcactgcgcccaccgtattctacttgggctatgcggtgcaccacctgtcc

cggcgccggaggatccagaagcaagaagaggaagaggagcccatgctcaaaaagaagtcc

gagaagtcccatgatgatggagcccacgatggtcgcaagaggatccgcagggatgggctc

ctggggccctatgttgggcagttactggtgcggactgccttggaggtggccttcctacta

ggccagtacctgctctatggcctggaggtgcctccctcctatgtctgcgtgcgtaagcct

tgcccccacaccgtggattgctttgtttctcgtcccacggagaagaccatcttcctgctg

gtcatgtatgcagtaagtggcctctgtctcctgctcaacctgatcgagttgctgcatctg

ggtctgggaagtttgaagaaggatagaagtcacaatgctccactttctcattccaagaat

ttcccactggagcagatgcagaaacagctgcatctggtccaggagcacctggacatggca

ttgcatatgagcacacctttggtcacagcgcctacctatgcaggccagtctccttcctac

aacatatatgctcagcaaaatctgcataatcaaagcgaaaaagagcctcatttctccaaa

acagatcagtga

# Supplementary Figure S7. Koala (*Phascolarctos cinereus*) *GJC3*.

The sequence is collected from GenBank.

>Pc-GJC3-XM_020995466-GJC1like

atgagctgggccttcctgacgaggttgctggaggctgtgacccagcactccaccctggtg

gggaagctctggctgtccgtcctggtggtgttccgcctggtactcctggccgtgggcggg

gaggccatttaccgggacgagctaagtggcttctcctgcaacacagcacagccaggctgc

caaaatgtttgctacgacgccttcgcacccctctcccacgtacgcttctgggtgttccag

atcatcctagtcactgcacccaccgtgttctacctgggctatgccgtgcaccatttgtcc

cggcgccaaatgacccaggaacaagaggaggaagagagggagcccatgatcaaagagaag

tccaagaagtccgctgaggatgaagcccacgatggtcgccggaggatccgcagggacggc

ctcctgggagcctatgtggggcagttgctgatccgatctgccttagaggtggccttcctg

atgggccagtacctgctctacggcctggaagtgcctccctcctatatctgccagcgcagc

ccttgcccccacaccgtggactgctttgtatctcgtcctactgagaagaccatcttcctg

ctggtcatgtatgcagtcagtggcctctgcctcctgctcaacctgattgagttgctgcac

ctgggcctgggaagcatgaagaagggtagaaaccaccctgccccaactcgtcattccaag

agtctccagttggagcaaatgcaaaaacagttgcatctggtccaggagcacctggacttg

gcggtgcatttgagcacacctttggccacggtgcctgcctattcaggccagtctccatcc

tacagcacatatgttcagcaaaaccggcacatccaagcccaaaaagaggctctattgtct

aagacaggtatatttggggtagattaa

>Pc-GJC3-XM_020995580-gjc1like

atgagctgggccttcctgacgaggttgctggaggctgtgacccagcactccaccctggtg

gggaagctctggctgtccgtcctggtggtgttccgcctggtgctgctggctgtgggtggg

gaggccatttaccgggacgaactgagcggattcgtgtgtaacacccggcagccaggctgc

caaaatgtttgctacgacgccttcgcacccctctcccacgtacgcttctgggtgttccag

atcatcctagtcactgcacccaccgtgttctacctgggctatgccgtgcaccatttgtcc

cggcgccaaatgacccaggaacaagaggaggaagagagggagcccatgatcaaagagaag

tccaagaagtccgctgaggatgaagcccacgatggtcgccggaggatccgcagggacggc

ctcctgggagcctatgtggggcagttgctgatccgatctgccttagaggtggccttcctg

atgggccagtacctgctctacggcctggaagtgcctccctcctatatctgccagcgcagc

ccttgcccccacaccgtggactgctttgtatctcgtcctactgagaagaccatcttcctg

ctggtcatgtatgcagtcagtggcctctgcctcctgctcaacctgattgagttgctgcac

ctgggcctgggaagcatgaagaatgggggatacagttctgccgcacctccgcattccaag

ggtttcaaacaggagctggagcaaatgcagaaacagctgcatctggtccagaagcaaatg

gacgtggcattgcatctgggcacacctctggcaacagcccctgagttttcaggtcggcct

ccaacctacaatatctctactcagcaaaaccgccccaaccaagcccagaaagagacccgg

ctctccaagacagagctgtga

# Supplementary Figure S8. Platypus (*Ornithorhynchus anatinus*) connexins

The chronology of the sequences is according to the Greek nomenclature. Size nomenclature is indicated in parentheses. GenBank accession number is given for each entry.

>Oa-GJA1-XM_029057743

ATGGGGGACTGGAGCGCCTTGGGCAAGCTCCTCGACAAAGTCCAGGCCTACTCGACGGCG

GGCGGGAAGGTCTGGCTGTCGGTCCTGTTCATCTTCCGGATCCTGCTCCTGGGGACGGCG

GTGGAGTCGGCCTGGGGGGACGAGCAGTCGGCCTTCCGGTGCAACACGCAGCAGCCCGGG

TGCGAGAACGTCTGCTACGACAAGTCCTTCCCCATCTCCCACGTGCGCTTCTGGGTCCTG

CAGATCATCTTCGTCTCGGTGCCCACCCTCCTCTACCTGGCCCACGTCTTCTACGTCATG

CGCAAGGAGGAGAAGCTGAACAAGAAGGAGGAGGAGCTGAAGGTGGCCCAGACGGACGGG

GTCAACGTGGACATGCACCTGAAGCAGATCGAGATCAAGAAGTTCAAGTACGGCATCGAG

GAGCACGGCAAGGTGAAGATGCGTGGCGGCCTGCTCCGCACCTACATCATCAGCATCCTC

TTCAAGTCCGTCTTCGAGGTGGCCTTCCTGCTGATCCAGTGGTACATCTACGGGTTCAGC

CTCAGCGCCGTGTACACGTGCCAGAGGGCCCCCTGCCCGCATCAGGTCGACTGCTTCCTC

TCCCGGCCCACGGAGAAGACCATCTTCATCATCTTCATGCTGGTGGTGTCCCTGGTGTCC

CTGGCCCTGAACATCATCGAGCTCTTCTACGTCTTCTTCAAGGGGGTGAAGGACCGCGTC

AAGGGCCGGATCGACCCCTACCTCGCGGCCACCGGCCTCCTGAaccggggcgccctgctt

caggtgatggagcgggaaatcacacccctcaggagagcgctggactttatttctggaacg

gtccagggaaaccccagtagtgggaatgaggagccactggaggaaattgaggagagagaa

agtttggtagaaagcggggaagaatctttccccaaggaagtgggataa

>Oa-GJA3-XM_001518776

ATGGGTGACTGGAGCTTTTTGGGGCGCCTACTAGAGAATGCCCAGGAGCATTCCACGGTC

ATCGGCAAGGTGTGGCTGACAGTGCTGTTCATCTTCAGGATCCTGGTGCTGGGGGCTGCG

GCCGAGGAGGTCTGGGGGGATGAACAGTCGGACTTCACGTGCAACACGCAGCAGCCCGGT

TGTGAGAATGTGTGCTACGATGAGGCCTTCCCCATCTCACACATCCGCTTCTGGGTGCTG

CAGATCATCTTTGTATCCACACCGTCACTCATCTACCTGGGCCATGTGCTGCACATTGTG

CGCATGGAGGAGAAGAAGAAGGAGAAGGAGGAGGAGCTGCTGAAAAAGACCGATCCGCAG

GCCGGGCCTGTGGGCCAGGGAGCTCCCGGCAGCCCCCGTGAGCCACTGCTCGGGAGGGAG

GTGGCCGGTGGGGGCAAGGAGAGGCCCCCAGTGCGGGATGAGTGGGGCAAGGTCCGCATT

GCCGGTGCCCTGCTCCGCACCTACGTCTTCAACATCGTCTTCAAGACCCTGTTTGAAGTT

GGCTTCATCGTGGGGCAGTACTTCCTGTACGGTTTTGAGTTGAAGCCCCTCTACCGGTGC

AGCCGCTGGCCCTGCCCCAACACCGTGGACTGCTTCATCTCCAGGCCCACCGAGAAGACC

ATCTTCATCGTTTTCATGCTGGCCGTGGCCTGCGTGTCCCTACTGCTCAACATGCTGGAG

ATCTACCACTTGGGCTGGAAGAAGTTGAAGCAGGGCATGACCAACCGTTACAGCCCCGAC

TCCCCCGAGGCCCAGCGGGGACTGGCGAGGCCGGATCAGCTCACCCCCTTCCTCTTGCCC

TCCCATGCGCCCCCGCCCCCCACCGTGACCATTGGGTTCCCCCCATTTTACAGCCAGTCT

TCCTCCTCCCTAAGGCAAGCTACCCCCTCGGGCTACCCCGGGGCCCCGGGGCCCCCGACA

GAGTTCAAAATGGCAGGGCTGCCCAAGGAGCGCAGCCGGGCCGCCTCCGCCCAGGCCTTC

AGCAGCAACCAGCACATCCTGACAGCAGAGCAGAACTGGGCCAACCAGGAGGCCGAGCAG

CAGACCCTCGAACGGAAGTCCCTCACTGCCCCTGGCGCCCCGTCTTCCCCAATCAGCACT

CAGCAGAACCCCCCGGAGGTGGGGGGAGGAGGTGAGGGTAGCGAGGCTCCCCTGCTGCTG

GTAAATGGCGGGAGCTTGGGAGGTGGCAGCGGTGGCGAGGCCGAGGCCGGGGAGGAGTGG

CCTGGGACCACCCGTGTGGAGATCCACGAGTCCCCGCTGCTCCTCCTGGACCTGAGGCGG

CTCAGCAAGGCCAGCAAGGCCAGCAGCTGTCGAGCCAGATCAGATGACTTGGCCATTTAG

>Oa-GJA4-XM_001507680

ATGGGCGACTGGGGCTTCCTGGAGAAGCTCCTGGACCAGGTGCAGGAGCACTCGACGGTC

ATCGGCAAGATCTGGCTGACCGTCCTCTTCATCTTCCGTATCCTCATCCTGGGTTTGGCG

GGCGAGTCGGTCTGGGGGGACGAGCAGTCGGACTTCGAGTGCAACACGGCCCAGCCGGGC

TGCACCAACGTCTGCTACGACAAGGCCTTCCCCATCTCCCACATCCGCTACTGGGTCCTC

CAGTTCCTGTTCGTCAGCACGCCCACCCTGATCTACCTGGGCCACGTCATCTACCTGTCC

CGTCGGGAGGAGAAGCTGAAGCAGAAGGAGAGCGAGCTGAGGGCCCTGCAGCTCAAAGAC

CTGCAGGTGGAGAACGCCTTGGCCGTACTGGAGAAGAAGCTCGCCAAGATCTCCGTGGCT

GAGGACGGGCGGGTGAAGATCCAGGGGGCCCTCATGGGCACCTACGTCACCAGCGTCGTC

TGCAAGAGCATCCTGGAGGCCGGCTTCCTCTTCGGCCAGTGGTACCTGTATGGCCTGACC

ATGGTGCCCAGGTTTGTGTGCGAGCGGGACCCCTGCCCCCACAAGGTGGACTGCTTCCTC

TCCCGCCCCACCGAGAAGACCATCTTCATCCTCTTCATGTGGGTCGTGGGCATTATCTCC

CTGCTGCTCAACCTCCTGGAGCTCTTCCACCTGTGCTGCAGGAAGCTGGTCCAGAGAGCA

AAGAGGGCGACCAAGGAGGGCGAGGCTGTCTATGACCCGGCCCTTAGGGAAGCCCCACCT

GGGGATTCCTATGCGCACCACCAAGAGAAGACCTTCTTCTACCTCCCCATGGTGGAGAAC

CCCTCCTCCCCGCCCTACCCCACCTACAACAAGCTGTCCTCCAGTGAGCAGAACTGGGCC

AACTTCACCACTGAAGAGAGCCTGGCGCTGCAAAAGAGGAACAGCTAG

>Oa-GJA5-XM_029080937

atgggcgactggagcttcctgggggagttcctggaggaggtgcacaagcactccaccgtg

gtgggCAAGGTCTGGCTGACCGTCCTCTTCATCTTCCGCATGCTGGTGCTGGGCACGGCG

GCCGAGTCCTCGTGGGGCGACGAGCAGGCCGACTTCCAGTGCGACACGCTGCAGCCCGGC

TGCGAGAACGTCTGCTACGACCAGGCCTTCCCCATCTCCCACATCCGCTACTGGGTCCTG

CAGATCATCTTCGTGTCCACGCCGTCGCTCGTGTACATGGGCCACGCCGTGCACCACGTG

CGCATGGAGGAGAAGCGGAGGGAGCGCCGGGCCGGGGCGGGAGAGAAGGAGGGAAGGGCC

GGGGAGGGAGACCAGGCCCGCCGGGGACCCGAGGAGGGCAGGATCGTGCTCCGGGGCGCC

CTGTTGCACACCTACGTGTGCAGCATCCTGGTGCGCACGGCCGTGGAGGTGGCTTTCCTG

GTGGGCCAGTACCTCCTGTACGGGGTCTTCCTGCAGACGCTGCACGTGTGCCGCCGCGCC

CCCTGCCCCCACGCCGTCAACTGCTACGTGTCACGGCCCACGGAGAAGAACGTGTTCATC

GTGTTCATGTTGGCCGTGGCGGCGCTGTCGCTGGGCCTCAGTCTGGCCGAGCTCTACCAC

CTGGGCTGGAGGAAGGCCCGGGGCCGGCTGCGGGGGCCGGGCGCGGGGGCCGAGGGCGAC

CTCTTCTTCGGGCCCGCGCTGGCGGCACGGCAGAACACAGCCAACCTGGCAGCTGAGCGT

GGGGGGCGAGCCCTGCCGGGCCCAGAGGCGGCCAGGGACAAGCGGCGTCTCAGCCACGCC

AGCAGCAAGGCCCGCTCGGACGATCTGTCCGTGTAG

>Oa-GJA8-XM_029080938

ATGGGTGATTGGAGCTTTCTGGGGAACATTCTAGAGGAGGTGAACGAGCACTCGACCGTC

ATCGGCCGGGTCTGGCTGACCGTCCTGTTCATCTTCCGCATCCTGGTGCTGGGTACGGCG

GCCGAGTTTGTCTGGGGCGACGAGCAGTCGGACTTCGTGTGCAACACGCAGCAGCCCGGC

TGCGAGAACGTGTGCTACGACGAGGCCTTCCCCATCTCCCACATCCGCCTCTGGGTCCTG

CAGATCATCTTCGTGTCCACGCCGTCGCTCGTGTACGTGGGCCACGCCGTGCACCACGTG

CGCATGGAGGAGAAGCAGAAGGAGCGCGAGGCGGAGGCGGCGGCCCGGAGGCCAGCGGGA

GAAGGGGATCGCTCACCCCCGGCCGGTGGCGGCGACCAGGGCCACGGGAAGCGGGGGCCG

TCCAAGGGCACCAAAAAGTTTCGGCTGGAAGGCACGCTTCTCCGCACCTACATTGGCCAC

ATCGTCTTCAAGACGCTGTTCGAGGTGGGCTTCGTTGTGGGCCACTATCTCCTGTACGGC

TTCCGGATCCGGCCCCTCTACCGCTGCAGCCGCTGGCCCTGCCCCAACGTCGTGGACTGC

TTCGTGTCCCGGCCCACCGAGAAGACCATCTTCATCCTCTTCATGCTGGCCGTGGCCTCC

GTGgccctcttcctcaacctcctggagatcggccacctgggcctcaagagggtccgggcg

gccttcggccggccggccgaaccccggtccccggggggccccgccgagacgtccgtccgc

tccgtcgccgtgtcctcggtgcagaaggccaagggctaccagctcctggaggaggagcag

atcgtgtcccggtacttcccgctgaccgacgtcggggtggtggagacgggggccctgggg

gacctgggtccggtctacgccgagaccctcccttcctacgcgcaggtggaggggcgggag

gtcggtggggaggagcaggaagagaagccaagcccagtggagaatcaggggctgcgggag

aagcctgggctgggggatacaccgggacttggggagaccctgggactggaagagaagctg

aggctgcaggagaagctggggttggaagagaagctgggactggagaggctggggctggga

ccagcagccgccgagaagctcccttcgctgccttccgagtggacgtccgactcccccagg

cccctcagcaggctcagtggggccagcagccgggcccggtcagatgatttgactgtctga

>Oa-GJA9-59-XM_001512804

ATGGGGGACTGGAATTTCCTGGGAGGCATTCTGGAGGAGGTCCACATCCACTCCACCATC

ATCGGAAAGATCTGGCTCACCATCCTCTTCATATTCCGGATGCTCGTCCTTGGAGTGGCA

GCTGAAGACGTCTGGAACGACGAGCAGTCCGGGTTCGTCTGCAACACCGAGCAACCGGGC

TGTAGAAATGTCTGCTACGATCGGGCCTTTCCCATCTCCCTCATCAGATACTGGGTCCTG

CAAGTCATATTTGTGTCTTCCCCATCTCTGGTGTACATGGGCCATGCTTTGTACAGACTG

AGGGCCCTGGAGAAGGACAGGCAAAGGAAGAAAGCTCAACTCAAAGGGGAACTGGAGGCC

ACTGAGTTTGAAATGGTCGAGGATCGGAGAAAGCTGGAGCGAGAACTCCGTCAGCTCGAG

CAAAGGAAACTCAATAAAGCACCACTGAGAGGGGCCTTGCTTTGTACCTACGTGATACAC

ATTTTAACTCGGTCTGCAGTTGAAATTGGATTCATGATTGGCCAGTATCTTCTCTATGGA

TTTCGGCTCGATCCTCTCTTTAAGTGTCATAGAGATCCATGTCCAAATACAGTCGACTGC

TTTGTATCGAGGCCGACAGAGAAGACGGTCTTCCTGTTATTTATGCAATCCATAGCGGCT

GCCTCTCTTCTTTTAAATGTTCTAGAAATTGTCCATCTCGGTTTAAAAAGAATCAAAAAG

GGATTTGAGAGACCCAAATATAAATATAAAATGAATGATGAATGTGAGGACTCTGATTTG

AGCGAGGCAAAAATAATTTCTGCAGCACAACCACCTTGTCTGGGCACAGCTACCAATCCA

CCCAAGACGCTCCCTTCTGCGCCTATTGGCTACACCGTGTTAAGGGAAAAACAAATGAAT

CCCACAGTGTACCCCGGCTTCAGTTCACCTTCAGTACTGCAGGCCCTTCAGGAAACTGGC

AAGAAAAGTCCGGCTAGTGACGAGAGAAATAAACCGTCTGACGAGACGTGCCTGAGTAGC

ATCGTGGAGGGTTATCCTGCGAACGCCAACTTGAGTCACAGCGAAGGGATAATCAGCGTG

GTGAGGGCAGAGATCGGTGGCACTCACAAGCAAGAGAACACCACCACCGGCCACCAGGCT

AATTTCGAAGCTGCCGTCCATCTGGGCTATCGGCCGGAGATGCCATTGGGGGCTGCCCTA

TACCCTTCACTGCAACCCGAAATGACTTTCTCCCCGCCAACCGATTGCACCCGCACAGCA

CGGAGCCTAAGCGACCCCTGGAACGGTTCGACGGGGGGTCTTCAGAGCAGAGGGTCACCT

CCCAGAGGTAACCTCAGGAGACAGAGCCGAGGGAGCACCGGCAGACCCCGAGCCCTCTCC

CGGGCGGACTCCCGACCACCCAGTAGGTCAAACAGCTCAGACTCTCCGGGGGAGATGAGC

TCGGGATCCAAACCCAGCAAAAGCTGTGACAGTCCTAGGGTTTTCCCACTTTCTCGGCGA

ATCTCTCTGGCGACTTGCAGCGTTAGCAGCAGGCGGGCCCCGACCGACCTTCAGATCTGA

>Oa-GJB1-XM_029067793

atgaactgggcgggcctgtacaccttgctgagcggcgtcaaccgccactccacggccatc

gggcgcgtgtggctgtcggtcatcttcatcttccgcatcatggtcctcgtggtggccgcc

gagagcgtgtggggggacgagaaggcggccttcacctgcAACACGCAGCAGCCCGGCTGC

AACAGCGTCTGCTACGACCACTTCTTCCCCGTGTCCCACGTGCGGCTCTGGTCCCTGCAG

CTCATCCTGGTGTCCACCCCGGCCCTCCTGGTGGCCATGCACGTGGCCCACCAGCAGCAC

GCCGAGAAGAAGCTCCTGCGCCTGGAGGGCCGCGGCGACCCCCTGCACCTGGAGGAGGTC

AAGCGTCAGAAGGTGCGCATCTCCGGCACGCTGTGGTGGACCTACGTGGTCAGCGTGGCC

TTCCGGCTGCTCTTCGAGGCCGTCTTCATGTACGTCTTCTACCTGCTCTACCCGGGCTAC

GCCATGGTCCGGCTGGTCAAGTGCGAGGCCTACCCCTGCCCCAACACGGTGGACTGCTTC

GTCTCCCGCCCCACGGAGAAGACCGTCTTCACCGTCTTCATGCTGGCCGCCTCGGGCCTC

TGCATCGCCCTCAACGTGGCCGAGCTGGTCTACCTGGTGGTCCGGGCCTGCGCCCGCCGG

GCCCGCCGCCGCCGGGCCGGCCCCCCGGCCCGCAAGGGCTCGGGCGGCTTCTCCCACCGC

CTCTCGCCCGAGTACAAGCAGAACGAGATCAACAAGCTCCTGGGCGAGCAGGACGGCTCC

CTCAAGGACCTCCTACGCCGCGGGCCGGGCTCGGCCCCGGCCGAGAAGGGTGACCGCTGC

TCCGCCTGCTGA

>Oa-GJB2-XM_001516318

ATGGATTGGGGAACACTGCAGACCATTTTGGGAGGCGTCAACAAGCACTCCACCAGCATT

GGGAAGATCTGGCTGACCGTCCTCTTCATCTTCCGAATTATGATCCTGGTGGTGGCCGCA

AAGGAAGTGTGGGGGGATGAGCAGGCCGACTTTGTCTGCAACACGCTACAGCCGGGATGC

AAGAATGTGTGCTACGACCACGTCTTCCCCATCTCGCACATTCGCCTGTGGGCTCTGCAG

CTCATCTTCGTCTCTACCCCGGCCCTGCTGGTGGCCATGCACATCGCCTACCGGAGACAC

GAGAAGAAGAGGAAGTTCATCAAGGGCGAGACAAAGAGCGAGTACAAAGATATCGAGGAG

ATCAAGAGGCAAAAGGTCCGGATCGAGGGAGCCTTGTGGTGGACCTACACCAGCAGCATT

TTCTTCAGAATCATCTTCGAGGCGGTCTTCATGTACGTGTTCTACTTCATGTACAACGGG

TTTTCTATGACCCGGGTGGTGAAGTGTAATGCCTGGCCCTGCCCCAACACAGTGGACTGT

TTCGTGTCCAGACCCACTGAGAAGACCGTGTTTACAGTCTTCATGATCTCCGTGTCTGGA

ATCTGCATATTGCTAAATGTCGTTGAGCTGTGCTACCTTCTGATCAGATACTGCTCTGGA

AAGTCCAAAAAGCCCGTGTAG

>Oa-GJB3-XM_0015008022

ATGGACTGGAAGACGCTCCAGGCCCTCCTGAGCGGCGTCAACAAGTACTCCACCGCCTTT

GGCCGCATCTGGCTGTCGGTTGTCTTCGTCTTCCGGGTGCTGGTGTACGTGGTGGCCGCC

GAGCGGGTGTGGGGCGACGAGCAGAAGGACTTCGACTGCAACACGAAGCAGCCGGGCTGC

ACCAACGTCTGCTATGACCATTTCTTCCCCATCTCCAACATCCGCCTGTGGGCCCTGCAG

CTCATCTTCGTCACCTGCCCGTCCCTGCTGGTCATCATGCACGTGGCCTACCGGGAAGAC

CGGGAACGCAAACACCGGGAGAAGCACGGGGAGGCGTGCCCCAGGCTCTACTCCAACACG

GGCAAGAAGCACGGGGGGCTGTGGTGGACCTACCTGCTCAGCCTCTTCTTTAAGCTCATC

ATCGAGATCGTCTTCTTGTACATCCTGAACAGGCTCTGGTACGGGTTCGACCTGCCCCGC

CTGGTGCAGTGTGCCTACGTGGCCCCCTGCCCCAACATCGTCGACTGCTACATCGCCCGC

CCCTCCGAGAAGAAGGTCTTCACCTACTTCATGGTGGGCGCCTCGGCTGTCTGCATCGTC

TTGACCGTCTGCGAGATCGCCTACCTCGTCTTCAAGCGAGTGGTGCGGTGTGCGCAGAAG

GGAGAGAAGCGGAAGGCCTACCCTGCCCCTTCGGCTAGCAGGGCCTCCACCTGCCGCTGC

CCCCACCGGCTGCTGGGCCCCGAGGGGGACCAGGCCGCGACCAAGTCTCCTGAGGGGCTG

CGGGCCTCTGCCCCCAACCTGACTCTGATCTGA

>Oa-GJB4-XM_029081152

ATGAACTGGGCGTCATTCCAGGAGCTCCTGAGCGGCGTCAACAAGTACTCAACTGCCCTG

GGCCGCATCTGGCTGTCAGTTGTGCTCGTCTTCCGAGTGCTGGTGTACGTGGTGGCGGCC

GAGGAGGTCTGGGACGACGACCACAAGGATTTCGACTGCAACACCCGCCAGCCAGGCTGC

ACCAATGTCTGCTTCGACCACTTCTTCCCCATCTCCCACGTCCGCCTGTGGGCCCTGCAG

CTCATCCTGGTGACCTGCCCTTCCCTGCTGGTCGTCATGCACGTGGCCTACCGGCAGGAC

AGAGAACGCAAACATCGGGAGAAGCACGGGGAGCACTGCGCGCAGCTTTACCCCAACCCG

GGCAAGAAGAGGGGGGGACTCTGGTGGACCTACCTGCTGAGCCTGCTCTTCAAGGCCGCC

GTGGACATCGGCTTCCTCTACGTCTTCCATCGCCTGTACGAGAACTACGACCTGCCTCGG

GTGGTGGGCTGTGCCGTGCCCCCCTGCCCCAACGTCGTCGACTGCTACATCGCCCGGCCC

ACCGAGAAGAAGGTCTTCACCTACTTCATGGTGGCCACCTCCTTGGCCTGCATCCTCCTC

AACCTGGGCGAAGTGGCATACCTGGTCGGCAAGAGATGCTCAGAGCTGCTCAGGCCACGG

CGACGGAGGCTGGTGGCAGTCCCCCGGGCGGCCTTGCCCGATGGCTTCCCCCCATACGTT

GTTCCCCAAGCCAGTCCCTCTCAGCGGGATGACCCATCCCCCGGCAAGGCCGACAATTCA

GCGTCCCCTCTGATGAGCTCGGGACACTTGAGATAA

>Oa-GJB5-XM_001507939

ATGAACTGGGGGATCTTTGAGGGGCTGCTCAGCGGGGTCAACCAGTACTCCACGGCCTTC

GGCCGCATCTGGCTGTCCCTGGTCTTCATCTTCCGGGTGCTGGTGTACGTGGTGGCGGCC

GAGCGGGTGTGGAGCGACGACCACAAGGACTTCGACTGCAACACCCGCCAGCCCGGCTGC

ACCAACGTCTGCTTCGACCACTTCTTCCCCGTCTCCCATGTCCGCCTGTGGGCCCTGCAG

CTCATCCTCGTCACCTGCCCGTCCCTGCTGGTTGTCATGCACGTGGCCTATCGGCAGGCC

CGGGAAAAAAAGCACCGGGAGCTGGTGGGAGAGGGCGGGGCTCGGCTGTACCTGGCCCTG

GGCAAGAAGCGAGGGGGGCTGTGGTGGACGTACCTCTTCAGCCTGATCTTCAAGGCCGGT

GTGGACTCGGTGTTCCTCTACATCTTCCACCGCCTGTACCGGAATTACACCCTGCCCCCC

CTCGTCAAATGTTCCCTCACCCCCTGCCCCAACATCGTGGACTGCTTCATCTCCAAGCCC

TCGGAGAAGAACATCTTCACCCTCTTCATGGTCATCACGGCCGCCGTCTGTATCCTGCTC

AACCTGGTGGAGGTAGCCTACTTGGTGGGCAAGCGAGGCCGGGAGTGCTGGGCGGTGGGG

GGACGCAAGCACGGGGACCCCGGGATCATGCTGCACGAGCCGGACTGCCCCTCCTCCCTC

AAAGAGCAAGAATTCCTCCTCCAACCGCACGTCCGGCTCCTCCGGGAGTCCGACTGTGAC

CCCCCCGGGGCCACGCTCCCCGCCGACATCCCGTGGGACCACGTGAAGAAGACTCTGGTG

TGA

>Oa-GJB6-XM_029047280

ATGGACTGGAGCACACTGCACACCTTCATTGGAGGTGTCAACAAGTACTCCACCAGCATC

GGGAAGATCTGGATCACTGTCATCTTCATCTTCCGCGTGATGATCTTGGTCGTGGCAGCT

GAGGAAGTGTGGGGGGATGAGCAAGAGGACTTTGTCTGTAACACGCAGCAGCCAGGGTGT

GAGAACGTGTGCTACGACCACTTCTTCCCTGTGTCACATATCCGTCTGTGGGCTCTGCAG

CTCATCTTCGTCTCCACCCCGGCCCTGTTGGTGGCCATGCATGTGGCCTACAGCCGGCAT

GAGAAGGCCAGGAAGTTCAGGAGGGGAGAGAAGAAGGGCGAGTTCAAAGATATCGAGGAC

ATCAAGAGGCAGAAGGTCCGGATCGAGGGAGCCTTGTGGTGGACCTACACCAGCAGCATT

TTCTTCAGAATCATCTTCGAGGCGTCCTTCATGTATGTGTTCTACTTCCTGTACAATGGC

TACAACCTACCGAGGGTGTTGAAGTGCAGCGCCAATCCCTGCCCCAACGTTGTGGACTGC

TTCATCTCGCGGCCCACCGAGAAGACCGTCTTCACCATCTTCATGATCTCGGCCTCGGTG

ATCTGCATGCTGCTGAATGTGGCGGAACTCTGTTACCTGCTGATGAAGGTCTGCTTCAGG

CAGTCCAAGCGGGCTCAGAACCAGGGGAGCCACCCAGGCCCGCCCACAAAGGAGGCCAAG

CAAAACGAGCTGAACGAGCTGATTGCGGAGGGCGCGTCGAACGCCATTCCAGGCTTCCCG

AGTTAG

>Oa-GJB7-XM_007670738

ATGAGCTGGGTCTTCCTCAGAGACCTCCTAAGTGGAGTAAATAAATACTCCACCGGAATT

GGCCGGATATGGTTGGCGGTCGTGTTCCTATTACGTCTACTGGTCTACATGGTGGCTGCG

GAACACGTTTGGAAGGATGAGCAGAAGGAGTTTGAGTGCAACACCCAGCAGCCCGGCTGT

GAAACTGTCTGTTTTGATCACTTCTTCCCCATCTCCCAGGCCAGGCTTTGGGCCCTGCAG

CTGATCATGGTCTCCACCCCTTCCCTGCTCGTGGCCCTCCACGTGGCATACCGTGAGAGC

CGCGAAAAGCGGCACAAGCAGCAGCTCTACTCCAACCCGGGGAGCATCGATGGTGGGCTC

TGGTGCACTTACCTCATCAGCCTCGTTTTTAAAACCGCCTTCGAAGTCGGCTTCCTGGCC

TTGTTCTATCAGTTGTACGGCGGGTTCAACGTGCCCCGCCTCGTGAAGTGTGATGTCAGG

CCTTGCCCCAGTACCGTGGACTGCTTTGTCTCCAAGCCTACCGAGAAGAAAATATTCATC

CACTTTCTGGTGGTCACCTCCTGCCTGTGCGTCGTTCTGAATGTCAGTGAGCTGAGCTAC

TTGGTGTTCACACGCTGTGTAAAGTGCTGCCTCCGCCGCTACCGCACCAGAGCAGAGGCT

TCGGCCTGCAAGTGCCACCAGCTCAACTGTGCCCGTGGCAGAGAGGTCACCACCTCCGCT

CTGATCCCGACTCACGTTCCGGATCCGGCCTTTCGCGTCCTCGCAGCGACTGAAGGGAAG

CTGCCGGAGGGCTCGTTGGAGGGATGA

>Oa-GJC1-XM_029074507

ATGAGCTGGAACTTCCTGACCCGCCTGCTAGAGGAGATCCACAACCACTCGACCTTCGTG

GGGAAGATCTGGCTGACGGTGCTCATCGTGTTCCGCATCGTGCTGACGGCCGTGGGCGGC

GAGTCCATCTACTACGACGAGCAGAGCAAGTTCGCGTGCAACACGGAGCAGCCGGGCTGC

GAGAACGTCTGCTACGACGCCTTCGCCCCGCTCTCCCACATGCGCTTCTGGGTCTTCCAG

ATCATCCTGGTGGCCACCCCGTCGGTCCTCTACCTGGGCTACGCCATCCACCGGATCGCC

AAGATGGAGCGCGGCGACGGGGACGGCAAGGGGGCGCGGGGCGGGCCCCACGCCGTGCGC

TGGAAGCCGCACCGGGCCCCGGAGGAGACGGAGGAGGACCGGCGGGAGGACCCCGCGATG

CTCCCGGAGGCGGACCCGGAGGGCGCCCCGGACGGCCAGGAGCCCGGCCCGCCCAAGCCC

AAGCACGACGGCCGGCGGCGCATCCGGCGAGACGGCCTGATGCGCGTCTACGTGGTCCAG

CTGCTGGCCCGCACCTGCTTCGAGGTGGCCTTCCTGCTGGGCCAGTACTTCCTGTACGGC

TTCCGGGTCAGCCCCTTCTACGTCTGCGGCCGCAGCCCCTGCCCCCACAAGATCGACTGC

TTCATCTCCCGGCCCACGGAGAAGACCGTCTTCCTGCTGATCATGTACGCCGTGAGCGGC

CTCTGCCTCGTCCTCAACCTCTGGGAGATGCTGCACCTGGGCTTCGGGACCCTGCGGGAC

TCGCTGCGCGACCCGAGGCCCCGGCCGGACCCGCCCCGCGGGACCCCGGACCCCTGGGAC

GCCCCGTCGGCGCCCCCGGGCTACAACATCGTCGTCAGGCCGGACCCGGGGGGCCCCTAC

ACGGAGCTGGCCAGCGCCAAGATCGCCTCCAAGCAGAACAAGGCCAACATCGCCCAGGAG

CGGCAGCAGTTCGGCAGCCACGAGGAGAACCTCCCGGCCGACCTGGAGGCCCTGCAGAGG

GAGATCCGCGTGGCCCAGGAGCGCCTGGACCTGGCCATCCGGGCCTACAACCACCACCGG

CACCACCACCCCCCGCACCGGCACCTCCCCGCCGGGCAGGAGAGGAAGGCACGGACCGGC

TCCAAGGGCAGCTGCGCCAGGAGCAGCGTCAGCAGCAAGTCGGGGGACGGGAAGGCCTCC

GTCTGGATCTGA

>Oa-GJC2-XM029078237

atgcccaacatgagctggagcttcctgacgcggctgctggaggagatccaccaccactcc

accttcgtgggcaaggtgtggctgacggtgctggtggtcttccgcatcgtgctgacggcc

gtgggcggcgagtccatctactcggacgagcagagcaagttcacctgcaacaccaagcag

ccgggctgcgacaacgtctgctacgacgccttcgcgcccctgtcgcacgtgcgcttctgg

gtcttccagatcgtcctcatctccacgccggccgtggtctacctgggctacgccgtgcac

cggctggcccgggccgccgacccccggccgcgggggcccccccgccgcccccgccgccgg

gggtcgggccgccggcgcccccgcgccccgcaggaccggccgggcccccgacgaggagcg

gccgatgctggacgggcggcggccaaggggcgcgcgacggcgacgggccacccccgcgac

gggcgccaggccggcgccgccggcgacgaggacgacgaggacgacgagcggngaggcggg

gggggcggcggggcgaccccggcggtccccggcggcccctcgcggcggcgggccgacggg

cggcggcgcatcgtgcgcgcggggttgatgcgcgtctacgtggcgcagctggtggcccgg

gccgggctggaggtgggcttcctggtgggccagtacctgctgtacggcttcgaggtgcgg

cccttcttccgctgcggccgggccccctgcccccacctggtcgactgcttcgtctcccgg

cccaccgagaagaccgtcttcctgctggccatgtacctcgtcagctgcctctgcctcctg

ctcaacctgggcgagatggcccacctgggctgcggcgccctgcgcgacgccgtgcgcctc

cgccgggcccccgggccgtccgggccccccgcctcccccgggccccccgactacgacctg

gtggtccgggccgagaagcccccgcgccctgggcccgtccccgtccccgtcccgccgggc

ctcccccgcggccccgagcccggcagctccagcagcagggacgggaagacctcggtgtgg

atctga

>Oa-GJD1.1-XM_029066083-gjd2like The first 67 nt of predicted cds (grey font) are somewhat deviating, and are not included in the analyses. This sequence is on platypus chr 5, while GJD1A is on platypus chr 13, but as there is no sign of genome duplication, we use the ".1/.2 nomenclature

atgggggagtggaccatcctggagaggctcctggaggcggcggtgcagcaacactccacc

atgattggcaggatcctgctgacggtggtggtcatcttccgcatcctgatcgtggccatc

gtgggcgagacggtgtacgaggacgagcaggccatgttcctgtgcaacacgctgcagccc

ggctgcgcccaggcctgctacgaccgcgccttccccatctcgcacatccgctactgggtc

ttccagatcatcctggtgtgcacgcccagcctctgcttcatcacctactccgtccaccag

gccgccaagcagcgggaggagcggcccgccgaccccccgccgccaccccggcctccgctc

aacggcatcctggcccacgggggccccgacgagccggtctgccccgaggtcaaggagccg

ggggcggcggggtcccgccggggtcccgccggcgcctcccgcttctacgtggtgcaggtg

gtgttccggaacgtgctggagctgggcttcctggccggccagtacctgctctacggcttc

cgcgtgccggccgtggtggcctgcggccgctacccgtgcctccgtgaggtggagtgctac

gtgtcccgccccaccgagaagagcgtcttcctggtgttcatgttcgccgtcagcggggtc

tgcgtcctgctcaacctggccgagctcaaccacctgggctggcggaaggtgcggggggcc

gtcaggggcgcccgggggcgcagaaaggcgcccggccgcgggggccagagggaccgggtg

cccctgcaccccgccctgggccgcacccagtccagcgagtcggcttacgtgtga

>Oa-GJD1.2-XM_029078184-GJD2

atgggagaatggaccatcctggagaggctgctggaggcggcggtgcagcagcactccacc

atgatcgggaggatcctgctgacggtggtggtcatcttccgcatcctgatcgtggccatc

gtgggcgagacggtgtacgatgacgagcagaccatgttcgtgtgcaacacgctgcagccg

ggctgcaaccaggcctgctacgaccgcgccttccccatctcgcacatccgctactgggtc

ttccagatcatcctggtgtgcacgcccagcctctgcttcatcacctactcggtgcaccag

gccgccaagcagcgggagcgccgctgctccgccgtcctgctggccctggagcgggagccc

cccgagcccgccggccccgccgcccccgccgccgccgacggcaagaagcggcccgtggtc

aacggcatcctgcagcccggggcccccgacccgccgcccgggcccgaggccaaggagccg

tcggccgtcgccgcccccggcccggcgggtcccccgggcccccgaggcctgcgcccgccc

ggccgggccaagctgcgtcgccaggagggcatctcccgcttctacgtcatccaggtgttt

ttccgcaacgccctggagctgggcttcctcctgggccagtacctgatgtacggcttccgc

gtgccgcgcctcttcgagtgcgaccgctacccctgcatcaaggaggtggagtgctacgtg

tcccgccccaccgagaagagcgtcttcctggtgttcatgttcgccgtcagcggcgtctgc

gtcctgctcaacctggccgagctcaaccacctgggctggcggaagatccggctggccgtg

cggggcgccagggccaagcgcaagtccgtctccgacctccgcgcccgggacctgccccgc

ctcggcctgcccgccttcggccggactcagtccagcgactcggcttacgtgtga

>Oa-GJD3-XM_029075902

atgggcgagtggaacttcctgagctccctgctggacgtgatccaggaccagtcgccgctg

gtgggccgcttctggctggtggtgatggtcatcttccgcatcctggtgctggccacggtg

ggcgacgacctcttcgaggacgagcaggacatgttcgtgtgcaacacggcccagcccggc

tgcaaacaggcctgctacgacctggccttccccatctcccactaccgcttctgggtcttc

cagatcgtgctgctctcgttgcccgccgtcctctacgtcgtctattcggtgcaccgggcc

accaaaggcccgggggccgggcccgggcagcccgcgcctcccggcccggcccgcccgggc

cgctgctacgtgctgagcgtcctggggcgccttctggccgagggggcattcctggccggg

caggcgctgctctacgggttccgggtggcaccgcggttcctctgcaggcgggccccgtgc

ccgcacctggtggactgctacgtcagccgccccaccgagaagacggtcttcctcatgttc

tacttctgcgtggggctgctgtcggccgccctcagcctggccgagctgggccacctggcc

tgcaagagccggccctggtgggtgggcacccccggccctgccccttcctcgccctcggcc

gacgccctcagggacaactgctgcaaccgggcgcaggagcagaagtcccagcctctcctc

accctggggcaccaggccgaggagaagcccccggccccgggacccgactcccccccggcc

tacagccgctgccaacccacggtatcctcggggtcgctgcccggagcgcggggggggctg

ctcatctag

>Oa-GJD4-XM_029077799

ATGGAACGCTGGGACGTGCTGGGGTTTCTGATCATCACACTGAACTCCAATGTAACCATG

GTGGGAAAGATGTGGCTCATCTTAATGATACTACTGAGGATGGCAGTGCTTGTTCTTGCA

GGCTATCCCATCTACCAGGATGAGCAGGAGAGATTCGTCTGCAATACTTTACAGCCGGGG

TGTTCCAATGTTTGTTATGACATTTTCTCTCCAGTGTCCCACTTGAGGTTCTGGCTGATC

CAGAGTGTTTCTGTGCTCCTTCCGTATGTGGTGTTCAGTGTCTACGTCTTGCACAGAGGG

GTCACGCACATTAGAACGGGGCCTTCTCCACCAGACAGCTGTAAAGGGGGTGACTCTCTG

ATGGGCCCCAGAGCTCCAACCTCCCCCAGATCACACTGCCTGACCCCCGGGAGTGTGGAC

ATTCCTGATTTTTCCTCAGCGTACACCCTACAGCTCATTCTGAGAACTCTGCTTGAAGTG

GCGTTCAGCCCTGGACAATATTATCTCTTTGGATTTCTGGTTCCCGAACAGTTTTCCTGC

TACCGCTCGCCTTGCACGAGCATGGTGGATTGTTATATCTCCCGTCCTACTGAGAAATCC

ATCCTGGTGCTTTTCATGTGGGGAGTCAGCGGCCTCTCCTTTGTCCTCAGCGTGGTCGAC

CTGGCCTGGGCCCTGCAGCGAAAGGTGGTGAGGAAAAGCCTCGCGAGACTGGGGACGGCA

CCATCCCACGCCCTGGACACGATTAATCAATCCCAGCTCCTTCCCGGGCCCCACAGGGAG

TGGGCTGCTGCTCCAGTTGAGCGAAATACCCTTTACCTGGAGGGGATGTCTAGTCAGGCT

AGTGAGAACCCTGGGTGGCCACAGCCCAGACAAGAAGTCACCATCCAGTCAACCACAGGG

TTGAACATACCCGGCGATAAGTCCAGTGTACTGGGGAGTGTGGATGATGGCAGCGAAGTG

ATCTCTTGTGTGAGTGAACGTCCTGGCCTCTCCCGCAGACAGTCCAGGGCTCAGCACGTG

AAAGAGGCAGCATTCTCTCTGCAACTTGACGAGAAATCTCAGCTGGGACGAGGCTCCTCG

GCAGCTTCAAGCCGGCTGGAGGGGCAACACCCACTCGGTGGGCGCAAGTCTTCTGAGGGG

CAGTTACTGTGTAGCAGCCCCAGCTACCTGAGATCAAAAAAGTCAGAATGGGTGTGA

>Oa-GJE1-XM_029052303

atgtctctaaattacatcaagaatttctatgaaggatgtctcaggccacctacagtgatc

ggtcaattccatacactttcctttggatcagttcgaatgttcttccttggggtactaggc

tttgcggtctatgggaatgaagccttgcatttcagctgtgaccctgacaagagggaaatc

aatctcttctgttacaaccagttcaggccaataactcctcaggtattctgggctttacag

ctagtgatagttcctggagctgtgttctatctttatgctgcatgtaaaaacatcgatcaa

gaaaacatccttcaaaggcccatctacacggtcttttatatcctttctgttctgtttagg

attatccttgaggtggtagcattttggcttcaaagccatctctttggttttcaagtaata

cccctttatcagtgtgacactgggtcacttgacaaaaatttaNagctcactagatgtgtg

gtaccagaacactttgaaaagaccatcttccttattgccatgtacacttttactgtgatt

actgtgttactgtgtgtggcagaggtctttgagatcttatgtagaaggttaggcttctta

agcagaccatga

# Supplementary Figure S9. Chicken (*Gallus gallus*) connexins.

The chronology of the sequences is according to the Greek nomenclature. Size nomenclature is indicated in parentheses. GenBank accession number is given for each entry.

>Gg-gja1-NM_204586-GJA1

ATGGGTGATTGGAGTGCCCTGGGAAAACTTCTTGACAAGGTTCAAGCTTATTCTACTGCA

GGAGGAAAAGTGTGGCTGTCTGTCCTCTTTATTTTCCGAATCCTGCTATTGGGGACAGCA

GTCGAGTCTGCTTGGGGAGATGAACAGTCTGCTTTTCGGTGCAATACTCAACAGCCTGGT

TGTGAGAACGTCTGCTATGACAAGTCCTTCCCCATCTCCCACGTGCGTTTCTGGGTTCTG

CAGATCATATTTGTGTCTGTACCAACCCTCTTGTACCTGGCGCACGTGTTCTACGTGATG

AGGAAAGAAGAGAAGCTGAACAAAAGAGAGGAAGAGCTCAAGGTGGTCCAGAATGATGGT

GTGAATGTGGATATGCACCTCAAACAAATAGAAATTAAGAAATTCAAGTATGGAATTGAA

GAGCACGGCAAGGTAAAGATGCGTGGAGGACTGCTTCGTACTTACATCATCAGCATCCTG

TTTAAGTCTGTCTTCGAGGTGGCTTTCCTGCTGATACAGTGGTACATTTATGGGTTTAGC

CTGAGTGCCATCTACACCTGTGAGCGAGACCCATGCCCTCACAGGGTGGACTGTTTCCTG

TCCCGTCCAACTGAGAAAACCATTTTCATCGTCTTCATGCTGGTAGTGTCTTTGGTGTCT

CTTGCCTTGAACATCATTGAGCTTTTCTACGTGTTCTTCAAGGGTGTCAAGGATCGTGTG

AAAGGAAAAACCGACCCCTACTCCCACAGTGGCACGATGAGCCCTTCCAAGGACTGTGGT

TCCCCCAAATATGCGTATTACAATGGCTGTTCCTCACCGACCGCCCCCTTGTCTCCCATG

TCTCCCCCGGGGTACAAGCTTGTTACCGGAGACAGGAACAATTCCTCCTGTCGTAACTAC

AATAAGCAAGCCAGCGAGCAAAACTGGGCCAACTACAGTGCGGAGCAGAATAGGATGGGG

CAGGCTGGCAGCACCATCTCCAACTCTCATGCCCAGCCCTTCGACTTCGCCGATGAGCAC

CAGAACACGAAAAAACTGGCGTCGGGACACGAGCTGCAACCCCTCACCATTGTGGACCAG

AGGCCTCCCAGCAGAGCCAGCAGCCGAGCCAGCAGCAGGCCTCGACCTGATGACCTGGAG

ATCTAA

>Gg-gja2-XM_015278408-GJA3like atgggggactggagcctgctggggaggctgctggagaacgcccaggagcactccacggtg

gtggggaaggtttggctcaccgtcctcttcgtcttccgcatcctggtgctgggggcggcc

gcggaacgggtgtggggtgacgagctggccggcttctcctgcgacacgcagcagcccggc

tgccagaacgcctgctacgacagcaccttccccatctcccacctgcgcttctgggtcctg

cagatcatcttcgtctccacccccagcctggtgtacctgggccacatcctgcacctggcg

cacctggaggagaaggcgcagcagcaggtggcggcgcggggtggcggcggggcccggcgg

cagcagggacagccccggatcccaacacgggacacctggggccgggtccgcatgcgtggg

gccatcctgaggacgtacgtctgcagcgtcgtcttcaaggctctctttgagacgggcttc

attgcggggcagtacgccttgtacgggcttgagctgaaacccctctacacctgcagccgc

tggccctgccccaacaccgtcaactgctacatctcccggcccaccgagaagaccatcttc

atcctcttcatgctgggggtggcctgcgtgtctctgctgctcaacctggtagagatttac

cacctgggcctcaccaagtgccggcagcggccacgtcccagatcccgcatcctgcccggc

cctggtggccttgcggggtcacacggtgcccgcttcaccttgcctggtggtggcaacccc

gtggatgccagcagaccccacctggcacccctggggaaggctggtgcgtggctgggggtg

gccagcgggtcccgtgggaaggcgcgagctggggacctggcagtgtga

>Gg-gja3-XM_015278214-GJA3

ATGGGTGACTGGAGCTTTCTGGGGAGGCTGCTAGAGAACGCGCAGGAGCACTCCACAGTT

ATTGGCAAGGTTTGGCTGACAGTACTGTTTATCTTCAGGATACTGGTGCTGGGGGCCGCT

GCTGAGGAAGTCTGGGGAGACGAGCAGTCAGACTTTACATGCAACACTCAGCAACCTGGT

TGCGAAAATGTTTGCTATGACAAAGCCTTCCCCATTTCTCATATCCGCTTCTGGGTGCTG

CAGATCATTTTTGTCTCCACTCCAACCCTCATATACCTGGGCCACGTGCTGCACATTGTA

CGCATGGAAGAGAAGAGGAAAGAAAAAGAGGAAGAGCTGAAAAAGAGGGGAAGTGTCAAA

GACAACAACTACCCAGGAGCAGCAACGTCTGGTGGTGGTAGTGGAGGTGGCAATAACTTC

AAGGATCCTCCTATCAAAATGGGAAAGGAGAAGCTTCCGATCCGTGATGAGCGTGGTAGA

ATCCGTATGGGAGGTGCTCTGCTCCGTACCTACATCTTCAACATCATTTTCAAGACACTG

TTTGAGGTAGGCTTCATTGTGGGCCAGTACTTCCTTTATGGCTTTGAACTAAAGCCAGTC

TACCAGTGCAGCCGTCCACCTTGTCCACACACTGTGGACTGCTTCATCTCACGGCCCACT

GAGAAGACAATCTTCATCATCTTCATGTTGGTGGTGGCCTCTGTCTCCCTGCTGCTGAAC

ATGCTTGAGATATATCACTTGGGATGGAAGAAGCTTAAGCAGGGCATGACAAGCCAGTAC

AGCCTTGAGATGCCTGTTACAACACTGACACCGGTCATGGTAACAGGGGAATCCAAACCA

GTTTCCTTGCCACCGCCAGCACCACCTGTGGTGGTAACGACCACTGCACCTGCTCCTGTT

CTGCCTGACACGCGTGCTGTCACACCGCTGCTGGCTCCAGTGACCATGGCACCCTACTAT

GCTGCGGCTGCTCCTAGGACACGACCCCCTTCCAACACTGCCTCCATGGCAAGCTACCCT

GTTGCTCCACCAGTTCCAGAGAACAGGCACCGTGCTGTCACCCCTACACCCGTCTCCACT

CCTGTCACCATCCCAACGCCCATCCCCACACCCACACCAGCCATCATCAACTACTTCAAC

AGCAAAAGCAATGCCCTGGCAGCCGAGCAGAACTGGGTCAACATGGCAGCTGAGCAGCAA

GGCAAGGCACCCTCCAGCTCAGCAGGTTCCTCCACGCCTAGCAGTGTCCGGCATCCCCTT

CCAGAGCAGGAAGAGCCCCTGGAGCAGCTTCTCCCACTCCCAGCTGGGCCGCCCATCACC

ACAACCAACAGTGGCAGCAGCACCAGCTTGAGCGGAGCAAGTGGCAGCAAATGGGATGTG GAAGGCGAGGAGGAGCTGGCAGAGGAACGGCCCATCTCAGCCACCTGCACCACCGTGGAG

ATGCATGAGCCGCCGTTACTTGTAGACACACGGCGTTTGAGCAGGGCCAGTAAGTCGAGC

AGCAGCAGAGCCAGGTCAGATGACTTGGCAGTGTAG

>Gg-gja4-NM_206984-GJA4

ATGGGCGACTGGGGATTCCTGGAGAAACTGCTGGACCAAGTCCAGGAGCACTCCACCGTG

ATCGGGAAGATCTGGCTCACCGTCCTCTTCATCTTCCGTATCCTCATCCTGGGCTTGGCT

GGTGAGTCCGTGTGGGGGGACGAGCAGTCGGACTTCGTGTGCAACACCAAGCAGCCGGGC

TGCACCAATGTCTGCTATGACAAAGCCTTCCCCATCTCCCACATCCGCTACTGGGTGCTG

CAGTTCCTCTTTGTCAGCACCCCGACCCTCATTTACCTGGGCCACGTTGTCTATCTCTCG

CGGAAAGAGGAGAAGCTGAAACAGCAGGAGAATGAGCTCCGGGCTATTCACAGCAAGGAC

CCAAAGATTGAGCAAGCATTGGCAGCTGTAGAAAAGAAGATGTCCAAGATCTACGTGACA

GAAGATGGGCGGCTCAAGATCCGAGGGGCACTGATGTGGACGTACGTCATCAGTGTGATC

TGCAAGAGCATCTTTGAGGCAGGCTTCCTCGTTGGCCAGTGGTACCTGTATGGCTTCTCC

ATGGTGCCCCGCTACGTGTGCAGGAGGGACCCCTGCCCGCACCAGGTGGACTGCTTCATC

TCCCGCCCCACCGAGAAGACCATCTTCATCATCTTCATGATGGTGATGGGCCTCATTTCC

TTAATCCTCAACCTCCTGGAGCTCTTCCACCTCTGCTGCAAGAGCCTGCTCAGCAGCATC

AAGAAGGTGCCGGTGCCGGTTGGCCCGAGCCAGAGTGCCTTTGCTGATGACATGGGCTCG

GGTCCCTACCCAGCCAAGCATTACCCCTTCCTGCCCATGGCTGAGAGCCACACACCGCCC

TACCAGGCTTACAACAAGCTCTCCAGCGAGCAGAACTGGGCCAACTTCCGCAACGAGGAG

AACCTGGCACTGGGCAGCGGCACCAGGCCCCTCTCGGACCCCTACGCCCCCAGGGCTCCA

GATGCCCCCATCCCTGAGGAGAAGCAGTGCAGCCGGCCCAGCAGCTCAGCATCCAAGAAG

CAGTATGTTTAG

>Gg-gja5-NM_205504-GJA5

ATGGGGGACTGGAGTTTCCTGGGAGAGTTCCTTGAGGAGGTCCACAAGCACTCCACAGTG

GTGGGGAAGGTCTGGTTGACTGTGCTGTTCATCTTCCGGATGCTGGTGCTTGGTACAGCA

GCTGAGTCCTCTTGGGGGGATGAGCAGTCCGATTTCATGTGTGACACCCAGCAGCCTGGC

TGCGAAAACGTCTGCTACGACAAGGCCTTCCCCATTTCCCACGTCCGCTTTTGGGTCCTT

CAGATCATCTTTGTCTCCACTCCATCCCTGGTGTATATGGGCCACGCGATGCATACGGTG

CGCATGGAGGAGAAGAGGAAGATGAAGGAGGCAGAAAGAGAGGCCCAGGAGATGAAAAAC

AGTGGTGACACATACTATCAGCAGAAGTGCCCCGTGGCAGAGAAGACTGAGTTGTCTTGC

TGGGATGAGTCAGGAGGCAAAATTATACTCAGAGGCAGTCTGCTAAACACCTATGTCTAC

AGTATTTTGATTCGCACTGCCATGGAAATCGCCTTCATTGTGGGGCAATACATCTTGTAT

GGGATCTTCCTGGAGACCCTGTATATCTGCCAGCGGGCACCTTGCCCCCACCCTGTCAAC

TGCTACGTTTCCCGTCCCACAGAGAAGAACGTGTTCATCATCTTCATGCTTGCTGTGGCT

GTGCTCTCCCTGTTCCTCAGCCTGGCTGAGTTATACCACCTGGGCTGGAAGAAAGCCAAA GAGAGGTGCTCCCGTGCTTACAAGCCCAGCCCTAGCACAGCTCCTCGAAGGCTGGAGTCC

GCCCCACAGGTAGAAAGGGCCCAGATGTACACTCCCCCACCAGATTTTAATCAGTGTTTG

GCAAGTCCCAATGGGAAGTTCATCAGCCCCTTCAGCAACAAGATGGCCTCGCAGCAGAAC

ACAGCCAACTTTGCCACTGAGAGGGTCCACAGCCAGGAGGATGCTGCCGGGGAAGGGCCC

TTCATGAAGTCCAGCTACATGGAAAGCCCAGAGGTGGCCAGTGAATGTGCGGCACCCGCC

CTCCCTGAGAGCTACTTCAATGAGAAACGCCGCTTCAGCAAGGCCAGCCGGGCCAGCAGC

AAGGCGAGGTCAGACGATTTGTCGGTGTGA

>Gg-gja8-XM_015295800-GJA8

ATGGGTGACTGGAGTTTCTTGGGGAACATTTTAGAGCAGGTGAACGAGCAGTCCACTGTC

ATCGGGAGAGTTTGGCTCACGGTGCTCTTCATTTTCCGCATCCTGATCCTGGGAACAGCT

GCTGAACTAGTATGGGGAGATGAACAGTCAGACTTTGTGTGCAACACCCAGCAACCTGGT

TGTGAGAACGTCTGCTATGATGAGGCCTTCCCCATCTCCCACATCCGGCTCTGGGTCCTA

CAGATCATTTTTGTATCCACGCCTTCGCTAGTGTACTTTGGGCATGCGGTGCACCACGTC

CGCATGGAGGAGAAGAGGAAAGAGAGGGAGGAAGCTGAGAGGCGTCAGCAAACTGAGGTG

GATGAAGAGAAGCTGCCCCTAGCTCCAAATCAAAACAAGGGCAACAACCCAGATGGGACC

AAGAAGTTTCGCCTGGAGGGGACCCTCCTGAGAACCTACATCCTCCACATCATTTTCAAA

ACTCTCTTTGAAGTGGGATTCATTGTTGGCCAGTATTTCCTGTATGGCTTCCGTATTCTC

CCCCTTTACCGCTGTGGGCGGTGGCCCTGTCCCAACCTAGTGGACTGTTTTGTCTCCAGG

CCCACAGAGAAGACCATCTTTATTATGTTCATGCTTGTGGTGGCTGCTGTGTCCCTCTTC

CTCAACCTGGTGGAGATCAGTCACTTGATCCTGAAAAGGATCCGGAGGGCTCTGAGAAGA

CCAGCAGAGGAGCAGATGGGGGAGGTGCCAGAGAAGCCCCTCCATGCTATTGCAGTGTCC

TCCATCCCGAAGGCCAAAGGCTACAAGCTGCTAGAAGAAGAAAAGCCAGTGTCCCACTAT TTCCCTCTCACGGAAGTAGGGGTTGAGCCCAGTCCCCTTCCATCAGCCTTCAATGAGTTT

GAGGAGAAGATTGGGATGGGGCCACTGGAAGATCTCTCCAGGGCATTTGATGAGAGGTTA

CCATCGTATGCACAAGCGAAGGAACCGGAAGAGGAGAAGGTAAAAGCAGAGGAGGAAGAG

GAACAAGAAGAGGAGCAGCAAGCACCTCAGGAAGAGCCAGGGGTGAAGAAAGCAGAGGAG

GAGGTGGTGAGCGATGAAGTGGAAGGGCCTTCAGCACCTGCTGAACTTGCCACCGATGTG

AGATCCCTCAGCAGGCTAAGTAAAGCCAGCAGCCGGGCCAGGTCAGACGATCTGACTGTA

TGA

>Gg-gja9_XM_025143016-GJA9

atgggagactggaatttcctcgggggcattctggaggaggtccacattcactccactatt

attggcaagatttggctaaccatcctcttcgtgttccgaatgctcgtccttggagtggcc

actgaggacgtctggaatgatgaacagtcagaatttatttgcaatactgagcaacctggc

tgtaggaacgtgtgctatgatgaggcctttcccatctctctcataagatactgggtcttg

caagttatctttgtgtcttctccttctttggtgtatatgggtcatgccttatacagacta

agagccttagagaaagagagacagaagaagaaagctcaggtgagagtggaacttgaaagc

actgaattagaaatgaccgaatatcggaaaaggctggagagggaacttcggcagctggac

caaaggaagctgaacaaagcacccctgcgaggttcactgctctgcacttacgtgatacac

attttcacgaggtctgcagtggaggtcggtttcatgattgggcagtatctgctttacggc

tttcgcctggatcccctttacaaatgtcagagagatccatgcccaaacgtagtcgactgc

tttgtatcacggccaacagaaaagacagtgttcatcctattcatgcagtcaatagcgact

gtatcactgcttttaaacgtcttagagatcatccacctcgggttccgaaaaattaaaata

ggtctctgtgggcaggaggacaacaaggatgaccgtggcaacttctacacaaacaaatcc

aagaagtactctgtgataccccgctcctctttgggaatatctgcaactcctcaaaagact

ctcccttctgtacttagcggttatgccttcttaatggagaagcaaaccgacactgccatc

tacccagtcttaaattctgctcccgtgttccagcctgtccaaaataaccacacagaaaat

aggggcaattacacccatcaccatcaggaaaacaaactgccgaagaaaaggccagctaca

aatgctgtagctggtcagactcagaatgctggcataaactgcagtgagggcttgctgggt

aggcctgggactgaaaggaggagttcccagaaagaagctgatcagaaacacttcctcgct

ggcgctcagaatgcagatacagctccaagaagctttgccgagatgctgtcgcagccccct

ctgcagccttttcctgttgccagcctcagaagacagcacggagtcgtctcctcctggaac

tgctccgcagcggccgagagcgtcgggtcctccacaaactccctcacgaagagcagcagc

aggaggcggagcagccttagaacgagccaaggccaactgccccccaaagccgacagccgg

caccccagccgtcccgacacccccgactcggcaggggaggccagcactggctccaagcag

agccgaggctgtggcagccctcaaccactgcccctatccggacgtctgtcactgtcgagc

agtgcgagcagccggcgggcccccaccgacctgcagatatag

>Gg-gja10-XM_001235096-GJA10

ATGGGCGACTGGAACTTGTTGGGCAGCATCCTTGAAGAAGTGCACATCCACTCCACTATA

GTTGGCAAAATCTGGCTCACGATCCTGTTCATATTCCGGATGCTAGTGCTGGGAGTGGCT

GCTGAGGATGTCTGGGATGACGAGCAGTCGGAGTTCATCTGCAACACAGAGCAACCTGGC

TGCAGCAATATATGTTATGACAAGGCCTTCCCTATCTCTTTGATCAGATACTGGGTATTG

CAGATCATATTTGTATCTTCTCCATCTCTTGTTTACATGGCCCATGCACTGTACAGATTA

AGGGCTCTAGAGAAAGAGCGACAGAAGAGGAAAGCCCACTTGCGGGCTCAGCTAGAAGAT

CTGGAGCCCATGCCTGAAGAACACCGGAGAGTGGAGAGGGAGCTGCGAAAGCTGGAAGAA

CAGAAGAAAGTGAATAAGGCACCCTTGAGAGGGTCTCTGCTGCGCACCTATGTCCTACAT

ATCCTGACCCGCTCAGTGGTTGAAGTGGGCTTTATGATAGGTCAGTATCTTTTGTATGGA

TTTCACATGTCACCCCTTTACAAATGTACTCGGCCCCCTTGCCCTAACACAGTGGATTGT

TTTGTGTCCCGACCCACAGAGAAGACCATCTTTATGGTCTTCATGCACAGCATTGCTGCA

GTCTCCCTATTCCTCAACATCCTAGAAATTGCCCACCTGGGCCTCAAGAAGATCCACAAG

ACCCTCTACGGGCGACTGCGGCAGCCCGTGGGCCCTGAGTATGACGAGGCCAGTCCTTAC

AACTCCAAGAAGAACTCCGTGGTCCCACCGGCCTGCCCGGCCGCCGACACCTCCCCTTCA

CGCTCTGCCGCGGCCCCGCCGCCTCCCAACGCTCCCTCGGCCACCCCCGCCCCTCCCTCC

CCTCCTGGGCAGCCGGACCATCAGCACCAGGGAGAGCTCCGAGCCACCGCCCCGCCGCGC

CGCCGGCACAGCGCGGCTCAGCACCACGGACAGCAGCCACCGCCGTCCTCCAGCAGCGAG

GAGGCGCCGCGGGCGGCGGGGCCGGGGACGGCGGGGCCCGGAACGGGGGCTGGCCGCCGA

GTGCTCCGTAAGCAGAGCCGCGTGAGCGTCTGTCGCGACCTGGAGGAGAACCGCGGGGAA

TCCCCGGACAGCGGGCACTGCCCGGGCACCCGCAAATCCAGCTTCCTCTCCCGGGTGCTC

TCCGAGAGCCGGGCGGGCAGCGACAGCGAGAGCGCCGCCTCCCGCCGAGGCTCCGGTCCC

GGCTCCGCCTCCTGTTCCGGCTCCGAGGGGCAGCGCCACGAGGAGGGCAGCCCGGCCGGC

AGCCCGCCGCCGCCCGCGGCCATGGGACGCCGCGTGTCGATGGCAAGTAGTGGCCCCGGG

GAGCGGGCGGGGGGTACTGACAGCCGGGGCAGCTCTTCTAAGTTTCCTGGCACCCCCACG

GCTATGATCGTGCCCCGCACCCTTCGTGGCCTTCGGCGGGGAAAGCAGAAGCGCTGCAGA

ACTGCGTTTCCATCAGCTGTTCATGTCGTTTGGCTGGATGTTCTAAACTTGCTTATACTC

AGTGTTAATGTCATGCTTCCCTCCCTGAACAAATGA

>Gg-gjb1-NM_204371-GJB1

ATGAACTGGGCCGGCCTCTACGCGGTGCTGAGCGGGGTGAACCGCCACTCCACCGCCATC

GGGCGCATCTGGCTCTCCGTCATCTTCATCTTCAGGATCATGGTGCTGGTGGTGGCGGCC

GAGAGCGTCTGGGGGGACGAGAAGTCCGCCTTCACCTGCAACACGCAGCAGCCGGGCTGC

AACAGCGTCTGCTACGACCACTTCTTCCCCATCTCTCACATCCGCCTGTGGGCCCTGCAG

CTCATCCTGGTCACCACGCCGGCCCTGCTCGTGGCCATGCACGTGGCCTACCAGCAGCAC

CAGGAGAAGAAGCTGCTGGTGATGACGGGCCACGGGGACGCCAAGCACCTGGAGGAGGTG

AAGAAGCACAAGATGCGCATCGCGGGATCGCTGTGGTGGACGTACGTCTGCAGCGTGGTC

TTCCGGCTGATCTTCGAGGCCGTCTTCATGTACCTCTTCTACATGATCTACCCCGGCTAC

CAGATGGTGCGGCTGGTCAAGTGCGAGGCCTACCCCTGCCCCAACACCGTCGACTGCTTC

GTCTCGCGGCCCACCGAGAAGACCATCTTCACCGTCTTCATGCTGGTCACCTCCAGCGTC

TGCATCGTGCTCAACATGGCCGAGTTGGTCTACCTGGTGGTGCGGGCCTGCACGCGCCAC

AGCCACCACAACTCCAACCCTTCCTCGGGGAAAGGCTCCTTCTACGGCCACAAGATCTCC

TCTGAGTACAAGCAGAACGAGATCAACCAGCTGCTGAGCGAGCAGGACGGATCCCTCAAG

GACATGCTGCGCCGCAACCCCGGCCTGCAGGAGAAGGGCGACCGCTGCTCCGCCTGCTGA

>Gg-gjb2-NM_001270816-GJB2

ATGGATTGGGGAACTCTGCAGGCTGTTTTGGGAGGTGTAAATAAACACTCCACCAGCATT

GGGAAGATATGGCTCACAGTCCTGTTCATCTTCCGTATCATGATCCTGGTTGTGGCTGCG

GAGAGAGTCTGGGGAGATGAACAGCAAGATTTTGTCTGCAATACGCTTCAACCTGGGTGT

AGAAATGTTTGCTATGATCACTTTTTCCCCATCTCTCACATCAGACTCTGGGCCCTGCAG

CTGATCTTCGTCTCTACCCCTGCACTGCTGGTGGCCATGCACGTGGCTTACACCAGGCAC

GAGAGGAAAAGGCGATTTAGAAGTGGTGATAAAATTAATATTGAAGAGCTGAAAAATGAA

AAGATCCACATTCGAGGTCCCCTGTGGTGGACGTATACCTGCAGCATCTTCTTCAGGATC

GTCTTTGAAGCAGTCTTCATGTACGTGTTCTACTACATGTACGACGGGTACCAGATGCCT

CGTCTGGTGAAGTGTGATGCGTGGCCCTGCCCCAATGTCGTGGATTGTTTCGTGTCTCGG

CCTACTGAGAAAACCACGTTTACTATTTTTATGCTTGCTGTATCTGGGATCTGCATGATG

TTGAATCTGGCTGAGTTGTGTTACTTAGTGATAAAAGTTTGCCTGAAAGATTCTGGGAAA

ACAACAGTTTTAAAGTAA

>Gg-gjb3-XM_004947781-GJB3

atggattggaagacgctgcaggggctgctcagtggggtcaacaaatactccacagccttc

ggtcgcatttggctctccgtggtcttcgtcttccgagtcctggtctatgtggtggcagcc

gaacgggtctggggggacgagcagaaggactttgactgcaatacacgccagccgggctgc

accaacgtctgctacgaccacttcttccccatctcccacatccgcctctgggctctgcag

ctcatctttgtcacttgtccctccctgctggtcatcatgcacgtggcttaccgggaggac

cgtgagaaaaagaacagggagaagaatggggagaattgtcccaagctgtacagcaacacg

ggcaagaagcacggcgggctgtggtggacctacctgttcagcctcttcttcaagctcatc

atagagatcctgttcctctacctcctccacaagatgtgggacagcttcgacttgccacgg

ctggtcaagtgcagcagcgtggagccctgtcccaacactgtagactgctacattgctcgg

ccaaccgagaagagagtcttcacttatttcatggtcggggcctcttccatctgcatcgtc

ctcactgtctgtgagatcttctacctcatcttcaagcgggttgtccggagcacacgcaag

ttgaagaagtccatcaagcgctctatgagctacagcaaagcctccacctgccagtgccac

ctcaaggcggaggagagggagaacaaggccctgaatagaggagaggagctgtga

>Gg-gjb5-XM_004947782-GJB5

ATGAACTGGTCGGTGTTTGAGGGGCTGCTCAGTGGGGTCAACAGATACTCCACAGCCTTC

GGCCGCATCTGGCTCTCCCTCGTCTTCATCTTCCGCCTTCTGGTCTACGTGGTGGCAGCC

GAGCGCGTCTGGAGCGACGACCACAAAGACTTCGACTGCAACACGCGGCAGCCAGGCTGC

ACCAACGTCTGTTATGACCACTTCTTCCCCATCTCCCACATCCGCCTGTGGGCTCTGCAG

CTCATCCTGGTCACCTGTCCCTCCCTGCTGGTCATCATGCACGTGGCTTACCGCCAAGCC

AAGGCGCTGCAGCACCGCGCCGAGAGGGGGGACAGCTACCGCTCCCGCTTCCCCAACCCC

GGCAAGAAGCGAGGCGGGCTGTGGTGGACGTACCTGTTCAGCCTCATCGTCAAGGCTGGC

GTGGACATGGTTTTCCTCTACATCTTCTACCACATCTACAGGAACTACACCCTGCCCCGC

TTGGTGAAGTGCCAGCTGCCCCCCTGCCCCAACATCGTGGACTGCTTCATCTCCCGGCCC

ACTGAGAAGACTATATTCACCCTCTTCATGGTGGTCACCACCTGCATCTGCATCGTGCTC

AGCCTGGTAGAGGCCGCCTACCTGATTGGCAAGCGGTGTCGGGAGCGCCTGCAGGCCGGC

ACTGGGGAGAGCCACTGCCACAGCCATGACTGTGCCGAGTCTGCAAGCACAGGGGGACAG

GTGTTCCATGGGGTGGGGTACAAAGCCCCCACAGCCTCCATCCCCAGTGCCTCCATCCCT

ACAACCTCCATCCCCACTGCCTCCATCCCTACAACCTCCATCCCCACCGCCTCCATCCCT

ACAACCTCCATCCCCACCACACTGCCCGAGCAGGAGAAGACTCCTTCCTAG

>Gg-gjb6-NM_204931-GJB6

ATGGATTGGGGAGCTCTGCAGACCATCTTAGGAGGTGTAAATAAACACTCCACCAGCATT

GGGAAGATATGGCTCACAGTCCTGTTCATCTTCCGTATCATGATCCTGGTTGTGGCTGCA

GAGAGAGTCTGGGGAGATGAACAAGATGACTTTATCTGCAACACTTTGCAACCTGGTTGC

AAAAATGTTTGCTATGATCACTTTTTCCCCATCTCTCACATCAGACTCTGGGCCCTGCAG

CTGATCTTCGTCTCTACCCCTGCGCTGCTGGTGGCCATGCACGTGGCTTACAGGAGACAC

GAGAAGAAAAGGCAGTTCAGAAAGGGAGACCAGAAATGCGAGTATAAGGACATTGAAGAA

ATCAGAACCCAGAGGTTTCGTATTGAGGGCACCCTGTGGTGGACGTACACTTGCAGCATC

TTCTTCAGATTGGTCTTTGAAGCAGTCTTCATGTATGCATTTTATTTCATGTATGATGGG

TTCCGAATGCCTCGCTTAATGAAATGTAGTGCTTGGCCCTGCCCCAACACAGTAGACTGC

TTTGTTTCTCGACCTACTGAAAAGACAGTGTTCACTATTTTCATGATTGCTGTGTCCAGC

ATTTGCATTCTTTTAAATGTGGCTGAATTATGTTATTTACTGACAAAATTTTTTCTCAGA

AGGTCTAGAAAAGCTGGAAATCAGAAACACCACCCCAACCATGAGAATAAAGAAGAAACC

AAACAAAATGAAATGAACGAGTTAATATCTGATAGCTGTCAGAACACAGTTATAGGATTC

ACAAGTAGCTAA

>Gg-gjc1-XM_015299213-GJC1

ATGAGTTGGAGCTTTCTGACCCGTCTATTAGAGGAGATCCACAACCACTCCACCTTCGTT

GGCAAAATCTGGCTGTCGGTGCTGATCGTCTTTCGGATCGTTCTGACTGCTGTGGGAGGC

GAATCCATTTATTACGATGAGCAAAGCAAGTTTGTCTGCAACACAGAGCAGCCCGGCTGT

GAGAACGTTTGTTACGACGCTTTTGCTCCTCTTTCTCACGTGAGATTTTGGGTGTTCCAG

ATCATTCTCGTCGCCACTCCATCCGTCATGTATTTGGGCTACGCCATCCATAAGATTGCC

CGCATGGTGGAGCACAGTGACGTTGACAGAAGGTTCAGAAGCAAAAGCTTTTCCACGCGC

TGGAAACAGCACCGCGGCTTGGAGGAGGCCGAGGATGACCACGAGGAGGACCCGATGATG

TACCCAGAGATAGAGCTGGAAAGCGAACGGGAGAACAAGGAGCAGCAGCCCCCCGCCAAG

GCCAAGCACGACGGGCGGCGGCGGATCCGCGAGGACGGGCTGATGAGGATCTATGTGCTG

CAGCTGCTGGTGAGGGCGACGTTCGAGGTCGGCTTTCTGATCGGTCAGTACCTCCTGTAT

GGCTTTGAGGTCAGCCCCGTGTTTGTGTGCAGCAGGAAGCCCTGCCCCCATAAGATAGAT

TGCTTCATTTCAAGGCCGACCGAAAAGACCATTTTCCTGCTCATAATGTACGGGGTGAGC

TGTATGTGTTTGCTTTTGAATGTGTGGGAGATGCTCCATTTAGGGTTTGGCACGATCCGG

GACACGTTGAACAACAAGAGGAAAGAGCTGGAGGACTCTGGTACCTACAACTACCCCTTT

ACCTGGAACACACCCTCTGCCCCTCCTGGCTACAACATTGCGGTCAAACCCGATCAGATG

CAATACACAGAACTGTCCAACGCCAAGATGGCCTACAAGCAGAACAAAGCCAACATCGCC

CAGGAGCAGCAGTACGGCAGCAACGAGGAGAACATCCCCGCCGACCTGGAAAACCTGCAG

AGGGAAATCAAAGTGGCTCAGGAACGCCTGGACATGGCCATCCAGGCGTACAACAACCAA

AACAACCCCGGCAGCAGCTCCAGAGAGAAGAAGTCCAAGGCGGGCTCCAACAAAAGCAGC

GCCAGCAGCAAATCGGGGGACGGGAAGAACTCTGTCTGGATTTAA

>Gg-gjc2-NM_001199581-GJC2

ATGAGCTGGAGCTTTCTCACCCGCCTGCTAGAAGAGATCCACAACCACTCCACCTTCGTG

GGGAAGGTCTGGCTCACTGTGCTCATCGTCTTCCGCATCGTCCTAACGGCGGTCGGTGGA

GAATCCATCTATTCTGATGAGCAAAGCAAGTTCACTTGCAACACCAAGCAGCCCGGCTGC

GACAACGTCTGTTACGATGCCTTCGCCCCGCTGTCACACGTTAGGTTTTGGGTCTTCCAG

ATCATCATGATATCCACGCCTTCAGTCATGTACCTGGGCTATGCCATCCACCGGATCGCC

CGGTCAGCGGAGGAGGAGAAGAAGTTCAAGGGCTTCAAGAAGAAGAAGCAGTTCGCTTTG

AACTGGCAGGCGGTGCGCAACATGGAGGATGCGATGGAGGCGGATGAAGAGGAGCCCATG

ATCTCCGATGACGCAGCAGAAAATGAGAAAGCCAAAGCCAAGCCCAAGAGCAAAGAGCAG

CAAAAGCACGATGGGAGGAGGCGAATCCAACAGGAAGGACTGATGAAAATCTACGTCTTC

CAGCTGCTTACCAGAGCTTCATTTGAAGTTTGTTTTTTGATAGGGCAGTATTTGCTCTAT

GGTTTTGAGGTAGAAGCTTATTTTGTCTGCAACAGACTGCCTTGTCCTCACACGGTGGAC

TGCTTTGTGTCCCGGCCAACAGAGAAGACAATCTTTCTGCTGGTGATGTACGTGGTGAGC

TGTCTGTGCTTGCTGCTGAACATGTGCGAGATGTTCCATCTGGGCTTCGGGACCATCCGA

GATGCCATCCGCAACCGGAAAATCAACAGCTTCAGGCAGCCCCCCTACAACTACGCCTAC

CCTAAGAACATCTCCTGCCCCCCCGAGTACAACCTGGTGGTGAAATCAGAGAAGTCCACC

AAGATCCCCAACAGCCTGATGGCTCACGAGCAGAACTTGGCTAACGTTGCTCAGGAGCAG

CAGTGCACCAGCCCGGATGAGAACATCCCAGCAGACTTGTCCACCCTCCACAAACACCTG

CGAGTGGCCCAGGAGCAGCTGGACATAGCATTTCAGAGTTATGGCAGCAGCCAGGGCAAT

GCACAGCCCTCCCGGACCAGCAGCCCTGCCTCGGGTGGCACGGTGGTAGAGCAGAATAGG

GCCAACACCGCCCAGGAGAAGCAAAGCGCTAAGCCCAAGGCGTCCTTGGAGAAAGGCAGC

TCCAGCAGTAAGGATGGAAAGACTTCTGTGTGGATATAG

>Gg-gjc4-XM_015289707-gjc1like

atgagctggagcttcctgacgcggctcctggaggagatcaacaaccactcgaccttcgtg

ggcaagatctggcttagcgtcctcatcatcttcaggatcgtgctgacggccgtggggggc

gagtccatctactacgacgagcagagcaagttcgtctgcaacacgcagcagccgggctgc

gagaacgtctgctacaacgccttcgccccgctctcccacgtccgcttctggatcttccac

atcatcatggtggccaccccctcggtgctctacctgggcttcgccatgcaccgcatcgcc

cgcatgcccgagtcctcgcggcgccgggcgccggcagctcagcgcggccgcatgccggtg

gtgcggcgcggagccgggcgcgactacgaggaggcggaggacgataacgaagaggacccc

atgatcttcgaggagatcgaggtggagaaggagaagggcgccgagggcggcgagaagcac

gacggccggcgccgcatcaagcgggacgggctgatgcgcgcctacgtgctgcagctgctg

tgccgcatgctgctggagtcggccttcctcttcgggcagtacctgctgttccgctttaag

gtcagcccctcgtacgtgtgcagccacagcccctgcccgcacatggtcgactgcttcgtc

tcgcgccccaccgagaagaccatcttcctgctcatcatgtacgccgtcagcgggctctgc

ctcttcctcaacatctgcgagctcctgcacctgggcgtggggcgcatccgcgacgcgctg

agccagcccaacggcccacccgccggcagccccgcgccgcactacgccaagaagcccccc

agcgcgccgcccacctaccactccctgaagaaggagccgctgaaagccccgctgcccgac

ggcaagctggactacagggagaacctggccaactcggcggccgagcgcttcgcgctggct

ggcgctccccgcgagcacgagctggagcggctgcgcgagcacctgcgcctggcccaggag

cacctggagatggccttccacctgcagccgccccctcgggcccccagcccctcgcgcagc

agcagccccgaggccaacggcatggctgccgagcagaaccgcctcaacctggcgcacgag

aagggcagcggctgtgagcgcagcacagggctgtga

>Gg-gjd1-XM_025144461-GJD2like gray font probably belongs to intron

atggcgatggcactgcgggtggcggcactgctggtggcggtggtggcggtggtggcaatg

gcggtggggacgatggcggccgcccccaagcaccgcccccccgcgctgtgtccccgcccc

acagccgctgcgcggggccgcgtttggggtgaaaggcaccgggttcgggcgccggacggc

ggtgggcgcagcgcggccgctgtgttttggggtggggggagaaaggggggcgaggggaag

cacagacgcttcccggctgctccgcagcgcatcgccgcagcgtcccggcacgcagcagca

gcggtgccccccgtgcgaccccccgaagcccgcaaccccccccagtgccccccgtgccca

cgccatccccgcaggatcctgctgacggtggtggtgattttccgcatcctgatcgtggcc

atcgtgggcgagacggtgtacgaggacgagcagaccatgttcatgtgcaacaccctgcag

ccgggctgcaaccaggcgtgctacgacaaagccttccccatctcccacatccgctactgg

gtcttccagatcatcctggtctgcacgcccagcctctgcttcatcacctactcggtgcac

caggcggccaagcagcgcgagcgccgctactccttcctttaccccctcctggagcgcgac

gccaagaaggcgcggacgctcaacggcgtgttggcacccggcgccgacggcggcaccaag

gatgagcccgagtgcctggaggccaaggagctgcccagcacgccgccgcgccgcgccaag

gccaagcggcaggagggcatctcgcgcttctacgtcatccaggtggtgttccgcaacgcg

ctggagatcggcttcctggccgggcagtacttcctgtacggcttcaacgtgcccgccatc

ttcgagtgcgaccgctacccctgcgtcaaggaggtggagtgctacgtctcgcggcccacc

gagaagaccgtcttcctggtgttcatgttcgccgtcagcgggatctgcgtgctgctcaac

ctggccgagctcaaccacctgggctggcgcaaggtgaaggcggccgtgcgcggcgtgcag

gcgcggcgcaaatccctggtggaggtgcgcaagaaggacggcgccgcgctgacccccgcg

cccaccctgggccgcacgcagtccagtgagtccgcctacgtttaa

>Gg-gjd2-NM_204582-GJD2 Splice site

ATGGGGGAATGGACTATTCTAGAGAGGCTACTGGAAGCTGCCGTGCAGCAGCACTCTACT

ATGATAGGGAGGATCCTGCTGACCGTGGTGGTGATCTTCAGGATCCTCATTGTGGCCATT

GTAGGGGAGACGGTGTACGATGACGAGCAAACTATGTTTGTCTGTAATACGCTGCAGCCA

GGCTGCAATCAGGCTTGTTACGACCAAGCTTTCCCTATTTCTCACATAAGGTACTGGGTG

TTCCAGATCATCATGGTGTGCACTCCCAGCCTGTGCTTCATAACGTACTCTGTTCACCAG

TCTGCTAAGCAGAGGGAACGGAGGTACTCCACTGTCTTCCTCACCTTGGAGAGAGACCAG

GATTCAATGAAGCGTGAGGACAGTAAGAAAATCAAGAACACGATTGTCAATGGGGTGCTG

CAGAACACTGAGAACTCCACCAAAGAGGCAGAACCAGACTGCTTAGAAGTGAAGGAGATC

CCCAATCCTGCGATCAGAACTACAAAATCAAAGATGAGGAGGCAAGAAGGCATTTCTCGA

TTTTATATCATCCAAGTGGTCTTTCGAAATGCCCTAGAGATTGGATTCTTAGTCGGGCAG

TATTTTCTGTATGGATTCAATGTCCCTTCCATGTACGAATGTGACAGATACCCTTGCATT

AAAGAAGTAGAGTGCTATGTCTCTAGACCCACTGAGAAGACTGTGTTCTTGGTGTTCATG

TTTGCTGTCAGTGGCATTTGTGTGGTGCTCAATTTGGCAGAACTGAACCACTTGGGCTGG

AGAAAGATCAAAATGGCAGTGAGAGGAGTACAGGCAAAAAGGAAATCCATATACGAAATC

AGAAATAAGGACCTGCCAAGAATGAGCATGCCTAACTTTGGCAGGACTCAGTCAAGTGAC

TCAGCTTATGTGTGA

>Gg-gjd4-XM_015282128-GJD4 Splice site

ATGGAGCACTGGGACTCGCTGGGATTTTTGGTTGTCACTCTCAACTATAATGTGACAATT

ATAGGAAAGATCTGGCTAATGCTGGTGGTTCTGCTGCGAATGGCAGTGGTGGTGTTAGCA

GGCTATCCGCTCTATCAGGACGAACAGGAGCGCTTCGTCTGCAACACCCTGCAGCCTGGA

TGCTCCAATGTTTGCTATGACTTGTTCTCTCCCATATCTCACTTCAGGTTCTGGCTCATT

CACACTGTGTCTATCTTGCTACCTTACACTGCATTCAGCATTTATGTTTTGCACAAGGTA

GCAACGCACATTGTAAGAAAGCACTGTTTGGTGTATGGATGCAGAGGGAATAAGGGTTTA

TCAAGTCCCAGAGACCTGAAGGAACTCTGTAGGAGTGATCTTGTCAATAGATTGGATTGT

GATGTGGACAGCCTAAGTGTCCTCAATTTTTCTGGGGCATATACTGTTCATCTATTTTTT

AGGACACTGATTGAGGCTGCTTTTGCAGCTGCACAGTATTTCCTCTTTGGATTTTTTGTT

CCCGAGCGCTTTTCCTGCTACCATTCACCTTGTACAAGCACAGTTGATTGCTACATCTCC

CGGCCCACTGAGAAATCCATCATGATGGTTTTCATCTGGAGTGTCACCAGTCTGTCCTTT

CTGCTCAGCCTTGCTGACCTTGTCTGTGCTCTCCGGCAAATGACAGCAAGAAGCCAGAGG

AACAAGCAGCTGGCCAACCCCCACAGAGAGAATGAGGGCCTTTTAAATAATCCCCCAGCA

CAGTCTGGTAGCTCTTCCCCACCCCAAACTCAAGACTGCCCAGCATTGAATGGCAGCCAG

ACCAGTGACGGCTCCTGCTCACTTCTCTCTGAAGAGGAAGAGGAGGCTACTCTTCATCCT

GAAGAGGTCTCTCAGCAAGCTGCCAGCACTAACCTTAACAGCAACAGCCATAAGCCTTGT

GCGTCAGGAGATCTTGCTGTTAAGCAAGATAGCATTGAAGAACCCCTGTGTGCTGATGAC

CAGCAAGGAACTGCATGCCAGCAAGTGAGACCTGGGCTCCAGCAAAGCTTTGTTAAAGAT

ACTGCCCTGACTTTGAGACCTCAGATCAAGTCTCACCTTGGAGGTTCTTCCTCTGTAGTT

CACAGCAAGCTTTTAGGATACTACCCTTCAGCTGAGCTAAAAAATCCCGATGCACAATCA

AATTATAGCAGTACTAGTTGCTTGAGATCGAAAAAGTCTGAATGGGTATAG

# Supplementary Figure S10. Zebra finch (*Taeniopygia guttata*) connexins.

The chronology of the sequences is according to the Greek nomenclature. Size nomenclature is indicated in parentheses. GenBank accession number is given for each entry. As we initially collected the sequences from Ensembl, may the sequences contain SNPs relative to the given GenBank entry.

>Tg-gja1-XM_002189379-GJA1

ATGGGTGATTGGAGTGCCTTAGGAAAACTTCTTGACAAAGTTCAAGCCTATTCTACTGCA

GGAGGGAAAGTGTGGCTGTCTGTCCTCTTCATTTTCCGAATCTTGTTATTGGGAACAGCA

GTTGAATCTGCTTGGGGAGATGAACAGTCTGCATTCCGGTGTAACACTCAACAGCCTGGT

TGTGAGAACGTCTGCTATGACAAGTCCTTTCCCATCTCCCATGTACGCTTTTGGGTTCTG

CAGATCATATTTGTGTCTGTACCTACCCTTTTGTACCTGGCACATGTGTTCTACGTAATG

AGGAAAGAAGAGAAGCTGAACAAAAGAGAAGAAGAGCTTAAAGTAGTGGCAAATGATGGT

GTGAATGTGGATATGCATCTCAAGCAAATAGAAATTAAGAAATTCAAGTATGGGATCGAA

GAGCATGGCAAAGTGAAGATGCGTGGCGGACTGCTCCGTACTTATATCATCAGCATCCTA

TTTAAATCTGTCTTCGAGGTGGCTTTCTTGCTGATACAGTGGTACATCTACGGATTTAGC

CTGAATGCCATCTACACCTGTGAGCGAGACCCATGCCCACATAGAGTGGACTGTTTCCTC

TCCCGTCCAACTGAGAAAACCATCTTCATCCTCTTCATGCTGGTGGTGTCCTTGGTGTCT

CTTGCCTTGAACATCATTGAGCTTTTTTACGTGTTCTTCAAGGGTGTCAAGGATCGTGTG

AAAGGGAAAACCGACCCCTACTCCCACAGCGGTGCCATGAGCCCTTCCAAGGACTGTGGC

TCCCCCAAATACGCTTATTACAATGGCTGCTCGTCACCGACCGCCCCCTTGTCTCCCATG

TCTCCCCCAGGGTACAAGCTTGTTACTGGAGACAGGAACAACTCCTCCTGTCGTAACTAC

AATAAGCAAGCCAGCGAGCAAAACTGGGCCAACTACAGTGCAGAGCAGAACAGAATGGGG

CAGGCTGGCAGCACCATCTCCAACTCGCACGCCCAGCCCTTCGACTTCTCTGATGAGCAC

CAGAACACGAAAAAACTGGCGTCAGGACATGAGCTGCAGCCCCTCACCATTGTGGACCAG

AGGCCTCCCAGCAGAGCCAGCAGCCGAGCCAGCAGCAGGCCTCGACCCGACGACCTGGAG

ATCTAA

>Tg-gja2-XM_002187811-gja3

ATGGGGGACTGGAGCCTGCTGGGCCGGCTGCTGGAGAATGCCCAGGAGCACTCCACAGTG

GTGGGGAAGGTCTGGCTCACCGTCCTCTTCGTCTTCCGCATCCTGGTGCTGGGGGCAGCC

GCGGAGCGGGTCTGGGGTGACGAGCTGTCTGGCTTCTCCTGTGACACGCAGCAGCCCGGC

TGCCAAAATGCCTGCTATGATAGCACCTTCCCTATCTCCCACCTCCGCTTCTGGGTCCTG

CAGATCATCTTTGTCTCCACCCCCAGCCTTGTGTATCTGGGCCACATCCTGCACCTGGTG

CATCTGGAGGAGAAGGCACAGCAGCAAAAGGCAGCACGGGCCAGCAGTGGGGCCAGGCGG

CAGCGCCCCAGGCAGCCCCAGCTCCCTGCAGGGAACACGAGGGCACGGGTCTGCATGCAG

GGGGCCATCCTGAGGACATACATCTGCAACATCGTCTTCAAGGCTCTCCTGGAAGTGGGT

TTCATTGTGGGCCAGTACACCTTGTATGGGTTCCAGCTGAAGCCCCTCTACACCTGCAGC

CGCTGGCCCTGCCCCAACACAGTCAACTGCTACATCTCCCGGCCCACTGAGAAGACCATC

TTCATCCTCTTCATGATGGGGGTGGCCTGCGTGTCCCTGCTGCTCAACCTGGTGGAGATC

TACCACCTGGGTCTCACCAAGTGCCGTCAGGGGCCAGGCCCCAAGTCCCGCATGCTGACC

GCTCCTGGTGGGCCTGTAGGGCCTGGCAGCACCTATGTCACTCTGCCCAGGGGTGGAAGC

CCCCTGGCTGCTGGCAGACCCCCCCGTGGCCTGGCACCGCTGAAGAAGGCAGGTGCATGG

CTAGGGGTGGTTGGTGGGTCCTACAGGGACACGCGAATGGCAGACCTGTCAGTGTAA

>Tg-gja3-XM_030275626-GJA3

ATGGGTGACTGGAGCTTTCTGGGGAGACTCTTAGAGAATGCGCAGGAGCACTCCACGGTT

ATTGGCAAGGTTTGGCTGACGGTACTGTTTATCTTCAGGATCTTGGTGCTGGGGGCTGCT

GCTGAGGAGGTCTGGGGAGACGAGCAGTCGGACTTTACATGCAACACTCAGCAACCTGGT

TGTGAAAATGTTTGCTATGACAAAGCCTTCCCCATTTCTCACATCCGCTTCTGGGTGCTG

CAGATCATTTTTGTCTCCACTCCAACCCTCATCTACCTGGGCCACGTCCTGCACATTGTA

CGCATGGAGGAGAAGAGGAAAGAGAAAGAAGAGCTGAAAAAGAAGGGAAACAGCAAAGAT

GGCAACTACCCAGTAGCAGCAGCATCTGTCAGCGGTGGTGGAGGAGCCAGCAGTAACGTC

AAAGATCAATCTATTGTCAAAAGGGGGAAGGAGAAACTCCCAATCCGTGATGAACGTGGT

AGAATTCGTATGGGGGGTGCCTTGCTCCGTACCTATGTCTTCAATATCATATTCAAGACA

CTCTTCGAGGTGGGCTTCATTGTGGGCCAATACTTCCTATATGGCTTCGAGCTAAAGCCA

GTCTACCAGTGCAGCCGCTCACCTTGTCCACACACTGTGGACTGCTTCATCTCGAGGCCC

ACTGAAAAGACAATCTTCATCATCTTCATGTTGGTGGTGGCCTCAGTCTCCCTGCTGCTG

AACATGCTTGAGATATATCACTTGGGGTGGAAGAAGCTTAAGCAGGGCATGACGAGTCAG

TACATCCTAGAGATGCCTGTTGCAACAATGACGCCAGTTATGGTAACAGGGGAGTCCAAA

CCTGTTTCCCTGCCGCCACCAGCACCACCTGTGGTGGTCACGACTGCCACACCACCCCCT

GTTCTGCCTGACACCCGTGCTGTCACACCACTGCTGGCCCCAGTGACCATGGCATCGTAC

TATGCTACAGGTGCTCCAAGACCACGGCCCCCCTCCAACACGACGTCCATGGCAAGCTAC

CCTGCTGCTCCACAGGTTCCTGAGGAGAGGCACCATGCTGTGACTCCCACGCCCATCTCC

ACTCCCGTCACCATCCCGACCCCCATCCCCACACCCACGCCAGCCGTCATCAACTACTTC

AACAGCAGCAGCCGTGCCCTGACATCTGAGCAGGACTGGGTCAACATGGCAGCCGAGCAC

CAGGGGAAGGTTTCCTCCAGCTCAGCAGGCTCCTCTACCCCCAGCAGCGTCCGGCATCCC

CTTCCTGAGCAGGAAGAACCGCTGGAGCAGTTGCTTCCACCCCCAGCTGTGCTGCCCATT

GCCTCAGCCAACAGCGGCAGCAGCACCAGCCTGAGCGGGGCGAGCGGCAGCAAGTGGGAT

GTGGAGGGCGAGGCAGAGCTGTCGGGGGCACGGCCCGTCTCGGCTGCCTGCACCACGGTG

GAGATGCACGAGCCGCCGCTGCTCGTAGACACGCGGCGCCTGAGCAGGGCCAGTAAGTCG

AGCAGCTGCAGGGCCCGGTCAGACGACCTGGCCGTGTAA

>Tg-gja4-XM_030290404-GJA4

ATGGGTGACTGGGAATTCCTGCAGAAACTGCTGGACCAAGTGCAGGAGCATTCGACGGTG

ATCGGGAAGATCTGGCTCACCGTGCTCTTCATCTTCCGCATCCTCATCCTGGGCCTGGCC

GGGGAGTCTGTGTGGGGGGACGAGCAGTCGGATTTCGTGTGTAACACCAAGCAGCCAGGC

TGCACCAACGTCTGTTACGACAAAGCCTTCCCCATCTCCCACATCCGCTACTGGGTGCTG

CAGTTCCTCTTCGTCAGCACCCCGACCCTGATTTACCTGGGCCATGTTGTCTATCTCTCC

CGCAAGGAGGAGAAGCTGAAGGAGAAGGAGAGCAAGCTTCGGGCTGTCCACAGCAAGGAC

CCCAAGATTGAGGAGGCCCTGGCAGACCTGGAGAAGAAGATGTCCAAGATCTACGTGACA

GAGAGTGGGCGGCTGAAGATCCGGGGGGCGCTGATGTGGACGTACATCACCAGTGTCATC

TGCAAGAGCATCTTTGAGGCCGGTTTCCTCGTGGGCCAGTGGTACCTGTATGGCTTCTCC

ATGGTGCCCCGCTACGTGTGCAAGAGGGACCCGTGCCCGCATCAGGTGGACTGCTTCATC

TCCCGCCCCACCGAAAAGAGCATCTTCATCATCTTCATGCTGGTGATGGGCCTGATCTCC

TTGATCCTGAACCTCCTGGAGCTCTTCCACCTCTGCTGCAAGAACCTGCTTAGCAACATC

AAGAAGGTGTCGGAGCCAGCTGGACCGAGCCAGGACACCTTTGTGGATCCCATGGTCTCG

AGTCCCTACTCCCCCAAGCACTATCCCTTCCTGCCCATGGCCGAGAGCCACGCGCCCTCC

TACCAGACCTACAACAAACTCTCCAGCGAGCAGAACTGGGCCAACTACCGCAACGAGGAG

AGCCTGGCACTGGGCAGCGGCAGCAGGCCCCTGTTGGACCCCTATGCCCCCGGGGCTGCC

GAAGCCTCCGCCCCGGAGGAGAAGCCGGGCAGCCGGCCCGGGAGCTCAGCCTCCAAAAAG

CAATACGTGTAG

>Tg-gja5-XM_030263295-GJA5

ATGGGGGAGTGGAGTTTCCTGGGAGACTTCCTCGAGAAGGTCCACAAACACTCCACAGTG

GTGGGGAAAGTCTGGCTGACTGTGCTCTTCATCTTCCGGATGCTGGTGCTGGGAACAGCC

GCGGAGTCCTCCTGGGGGGACGAGCAGTCTGACTTCATGTGTGACACCCAGCAGCCCGGC

TGCGAGAACGTCTGCTACGACAAGGCCTTCCCCATCTCCCACGTCCGCTTTTGGGTCCTC

CAGATCATCTTCGTCTCCACCCCGTCCCTGGTGTACATGGGCCACGCCATGCACACGGTG

CGCATGGAGGAGAAGAGGAAGATGAAGGAGGCAGAAATAGAGGCCCGAGAGAACAAAAAC

GGTGGTGACACATACTACCAGCAGAAGTGCTCCGTGGCAGAGAAGGAGACCAAGCTGTCT

GGCTGGGATGAAGCAGGAGGCCAAATCATACTCAGGGGCAGCCTGCTGAACACCTACGTC

TACAGCATTTTGATTCGAACTGCCATGGAAGTGGCCTTCATTGTGGGGCAGTACGTCCTG

TACGGGATCTTCCTGGAGACCCTGTACATCTGCCAGCGGGCACCGTGCCCCCACCCTGTC

AACTGCTACGTGTCCCGCCCCACGGAGAAGAACGTGTTCATTGTCTTCATGCTGGCTGTG

GCAGTGCTCTCCCTCTTCCTCAGCCTGGCCGAGCTGTACCACTTGGGCTGGAAGAAAGCC

AAGGAGAGGTGCTCCAGGTCTTACAAAGCCAGCCCCAGCACAGCCCCCGGCAGGCTGGAG

TCTGCCCCGCAGGCGGACAGGCCGCAGATGTACACCCCACCGCCGGATTTTAACCAGTGC

TTGTCAAGTCCCAACGGGAAGTTCATCAGCCCCTTCAGCAACAAGATGGCCTCCCAGCAG

AACACCGCCAACTTCGCCACCGAGAGGGTCCACGGCCAGGAGGATGCTGCTGGGGAAGGG

CCCTTCATTAAGTCCAGCTATGTGGAGAGTCCAGAGGTGGCCAGTGAATGTGCGGCACCT

GCCTTCCCCGAGAACTACTTCAACGAGAAACGCCGGCTCAGCAAGGCCAGCCGTGCCAGC

AGCAAGGCAAGGTCGGATGATTTGTCTGTGTGA

>Tg-gja8-XM_030263291-GJA8

atgggtgactggagtttcttggggaacattttagagcaggtgaacgagcaatccactgtc

atcgggagagtttggctcacggtgctcttcattttccgcatcctgatcctgggcacggct

gccgagctcgtgtggggagacgagcagtcagactttgtgtgcaacacccagcaaccaggt

tgtgagaacgtctgctacgatgaggccttccccatctcccacatccggctctgggtcctg

cagatcatttttgtatccaccccttcactaatgtacttcgggcatgctgtgcaccacgtc

cgcatggaggagaagaggaaggagaaggaggaagccgagaggcgccagcaagccgaggtg

gatgaagagaagctgcccctggctccaaaccaaaacaaaggcaacaaccctgatggaacc

aagaagtttcgcctggaaggtactctcctgagaacgtacatcttccacatcattttcaaa

accctctttgaggtgggattcatagtaggtcagtacttcctgtatggcttccgaattctc

cccctttaccgctgcgggcggtggccctgtcccaatcttgtggactgttttgtctccagg

cccacagagaagaccatcttcattatgttcatgctggtggtggcctccgtgtccctcttc

ctcaacctggtggagatcagtcatttgatcttgaaaagaatccggaaggctctgaggaga

ccggcggaggagcagcttggagaggtcccagagaagcccctccatgccatcacagtccct

tccatcccaaaggccaaaggctacaagctgctggaagaagagaagccagtgtcccactat

ttccctctcacggaagtaggggttgagcccagcccccttccatcagcctacaatgagttt

gagaagattggaatgggaccactggaagatctctccagggcattcgatgagaggttacca

tcgtatgcgcaagcaaaggaaccagaagaggagaaggtacgagcagaggaggaggaacga

gaggaggagcagccagggcctcaggaagagccggggatgaagaaagcagaagaggaggtg

gtgagagatgaagtggaagggccttcagcacctgctgagcttgctgctgatataagaccc

ctgagcaggctaagtaaagccagcagccgggccaggtcagatgatttgactgtatga

>Tg-gja9-XM_012570685-GJA9-59 This sequence has been removed as a result of standard genome processing, and has been replaced by XM_032752650. However, the new sequence lack the two indicated nucleotides in the second conserved domain. Without these two nucleotides, there is a frame shift, causing an unexpected 3'end of the second conserved domain. For the time being, we stick with the old sequence.

ATGGGAGACTGGAATTTCCTTGGAGGCATTTTAGAGGAGGTCCACATTCATTCCACTATT

ATTGGAAAGATTTGGCTAACGATCCTCTTCATATTTCGAATGCTTGTCCTTGGAGTGGCA

ACCGAGGATGTTTGGAATGATGAACAATCAGAATTTATATGCAATACTGAGCAGCCTGGT

TGCAGAAATGTGTGCTATGATGAGGCCTTTCCCATCTCTCTCATAAGATACTGGGTTTTG

CAAGTTATATTTGTGTCTTCCCCTTCTTTGGTGTATATGGGTCATGCCTTATACAGACTA

AGAGCATTGGAAAAAGAGAGGCAAAAAAAGAAAGCTCAGGTAAGGGTGGAACTTGAAAGC

ACTGAATTAGAAATGACTGAAAATCGTAAAAGACTGGAGAGAGAGCTCCGGCAATTGGAT

CAAAGAAAGCTAAACAAAGCACCCCTAAGAGGCTCTTTGCTCTGCACTTATGTGATACAT

ATTTTCACAAGATCTGCAGTGGAAGTTGGCTTTATGATTGGGCAGTATCTTCTTTATGGT

TTTCACTTAGATCCCCTTTATAAATGTCAGAGAGACCCATGTCCAAACACAGTTGACTGC

TTTGTATCTAGACCAACAGAAAAGACAGTGTTTATATTATTTATGCAATCAATAGCAACT

GTATCATTGCTTTTAAATATTCTAGAAATTATCCACTTAGGATTCCGTAAAATTAAAATG

GGACTCTGTGAGCAGAATAAAAACAAAGATGACTCCGACAATTTCTACATAAACAAATCT

AAGAAATACTCTGCGATACCACACTCTTCTTTGGGAATATCCACCACCCCTCAGAAAACA

CTTCCTTGTGCTCTTAGCAGTTATACCTTTTTGATGGAAAAGCAAACTGACACTATGCTC

TACCCAGTTTTAAATTCTCCTTCTATGTTTCAGTCTGTGCAAAATAACCGTACAGAAAGC

AGCAGCAATTACACCCATTGCAATCAGGAAAATAAATCTCCAAAGAAGAGGCCAGCTACA

AATGCTTTAGACAATCAGACTCAAAATGCTAGCACAAATAATAATGAAGGCTTTCTTGGT

GACCTTTGGACTGAGACACATGATGCACAAGAAGAGGCTGAAAAGAAACATTTTCTTGTT

GGTACTCAGAGTGCAGATACTGCCTCAAACATGTACTTGAGAAGCTTTGCTGAGTTGCCA

TGTCAAACTTCATTGCAACCTGGTACAACTTTTCCTAGTGCCAGTTTTAGACAACAGACA

ATGGTTGAGGACTCAGGAACATTAACAAATTCTCTTCCAAAGAACAGGGACAGAAGACAG

AGCAGTTTCAGTGTAAACAAAGCCCAAATTCCTTACAATGCTGATGGAAAAAATTCCAGC

CGACCAGACACTTCTGATTCTATGGGGGTGGTGAGCTCAGAATCTACACAAAGCAGAAAT

TGGAATAGTCCTAAGCTTTTGTCTCTGTCTAGGCAACAGTCACTGTCAAGTAATGCCAGC

AGCAGGCGTGCCCCAACTGATCTTCAAATATAG

>Tg-gja10-XM_030269352-GJA10

ATGGGGGACTGGAACTTGTTGGGCAGCATCCTTGAAGAAGTGCACATCCACTCCACCATA

GTTGGCAAAATCTGGCTCACAATCCTCTTCATATTCCGGATGCTGGTGCTGGGAGTGGCT

GCTGAAGATGTCTGGGATGATGAGCAGTCCGAGTTCATCTGCAACACGGAGCAACCTGGC

TGCAACAATATCTGTTACGACAAAGCCTTCCCCATTTCTTTGATCAGATACTGGGTACTT

CAGATCATATTTGTCTCCTCCCCATCTTTGGTGTACATGGGCCACGCGCTCTACAGGTTA

AGGGCGCTGGAGAAAGAGCGACAGAACAGGAAAGCCCACCTGCGGGCTCAGCTCGAGGGC

CTGGAGCCCATGCTCGAGGAACACAAGAGAGTGGAGAGAGAACTGCGTAAGCTGGAGGAA

CAGAAGAAAGTGAATAAGGCACCCTTGAGAGGGTCCCTGCTGCGCACCTATGTCCTACAT

ATCCTCACCCGGTCGGTGGTCGAAGTGGGCTTTATGATAGGTCAGTATCTTTTATATGGG

CTTCACATGTCTCCCCTTTACAAATGCACTCGGCCCCCTTGCCCTAACACGGTGGATTGT

TTTGTGTCCCGACCCACAGAGAAGACCATCTTTATGGTTTTCATGAACAGCATCGCCGCC

GTCTCCCTCTTCCTCAATATCCTCGAAATCGCCCACCTGGGCATCAAGAAGATCCGGAAG

AGCCTGTGCGGGCAGCCGCTGTGGCCGCCGGGCCCGTCCGAGGAGGATCCCAGCGCGTAC

AACTCCAAGAAGAGCTCGGTGGTGCCGCCGCCGCCCTGCCTGGCCAGCAACGccagcgag

gaggcgccgcgggccgcgccggggggcggcggggcgggggcggccgccgcggcccgccgg

gctccccgccggcacagccgagtgagcgcctgccgcgatctgggtgaggagcgcggcgac

tccccggacagcgggcactgccccggcacccgcaagtccagcttcctctcccgcgtcctc

accggcagccgggcgggcagcgacagcgagagcaccgcctcccgcggcggccccggcccc

ggccccagccccggccccggctccggctccggctccggctcggaggggaagctccgctcg

gagggcgcgcccagcccgccgcccgccgcctcgggacgccgcgtgtccatggcaagtagc

gcccgggacagcccgcgggaggggggcggcggggggcatcggcatccagtgccggccctg

gggaggagggggcgagccagggggcagaacggctcgggcaggacttccaacacagcccca

gctcctcgcggctccccggacagagccaagctcccaaacgctccgcctccttctgccgtg

aaagcaggaataccagggaattactga

>Tg-gjb1-NM_001245217-GJB1

ATGAATTGGGCCGGCCTCTACACGGTGCTGAGCGGGGTGAACCGCCACTCCACCGCCATC

GGCCGCATCTGGCTCTCTGTCATCTTCATCTTCCGGATAATGGTGTTAGTGGTGGCAGCC

GAGAGTGTCTGGGGGGATGAGAAATCTGCCTTCACCTGCAACACCCAGCAGCCTGGCTGC

AACAGTGTCTGCTATGACCACTTCTTCCCCATCTCCCACATCCGTCTGTGGGCCCTGCAG

CTCATCCTCGTCACCACACCAGCCCTTCTCGTGGCCATGCACGTGGCCTACCAGCAGCAC

CAGGAGAAGAAGCTGCTGGTGCTGACAGGGCATGCGGATGCCAAGCACATGGAAGAGGTC

AAGAAGCACAAGATGCGCATAGCGGGTTCACTGTGGTGGACATACGTTTGCAGCGTGGTC

TTCAGGCTGCTCTTCGAGGCCGTGTTCATGTACATCTTCTACATGCTCTACCCCGGCTAC

CAGATGATGCGGCTGGTTAAGTGTGAGGCTTACCCCTGCCCCAACACTGTCGACTGCTTC

ATCTCCCGGCCCACTGAGAAGACCATCTTCACTGTCTTCATGCTGGTCACCTCCAGTATC

TGCATCATCCTCAACATGGCAGAGCTGGTCTACCTGGTGGTGCGGGCTTGTGCCCGCCGA

AGCCAGCACCACGCCAACCCTTCATCGGGGAAGGGCTCCTTCTATGGGCACAAGCATTCC

TCCGAGTACAAGCAGAATGAGATCAACCAGCTGCTGACAGAGCAGGACGGTTCCCTCAAG

GACATGCTGCGTCGCAACTCTGGGCTGCAGGAGAAGGGTGACCGCTGCTCTGCCTGCTAG

>Tg-gjb2-XM_030275618-GJB2

ATGGACTGGGGAACTCTGCAGGGTGTTTTGGGAGGTGTAAATAAGCACTCCACCAGTATT

GGGAAGATATGGCTCACAGTCCTCTTCATCTTCCGTATCATGATCCTGGTTGTGGCTGCA

GAGAGAGTCTGGGGAGATGAACAACAAGATTTTGTCTGCAACACACTTCAGCCTGGTTGC

AGAAATGTTTGCTATGATCACTTTTTCCCCATCTCTCACATCAGACTCTGGGCCCTGCAG

CTGATCTTTGTCTCCACACCTGCACTACTGGTGGCCATGCATGTGGCTTACACCAGACAT

GAGAAGAAAAGGCGGTTCAGAAATGGTGAGAAAATTGATATTGAAGAGCTAAAAAATAAA

AAGATTCACATTCGGGGCCCCTTGTGGTGGACGTACACCAGCAGCATCTTCTTCAGGATC

ATCTTTGAAGCCGTCTTCATGTACGTGTTCTATTACATGTACGATGGCTACCAGATGCCC

CGCCTGGTGAAGTGCGACGCTTGGCCCTGCCCCAACACTGTGGATTGTTTTGTGTCTCGG

CCCACTGAGAAAACCACATTTACTATTTTCATGCTTGCTGTATCTGGGATCTGCATGATG

TTGAATCTGGCTGAGTTGTGTTACCTAGTGATAAAAATTTGCATGAAA

GAATCTGGGAAAACAACAGTTTTGAAATAA

>Tg-gjb3-XM_030290507-GJB3

ATGGATTGGAAAACGCTGCAGGCGCTGCTCAGCGGGGTCAACAAGTACTCCACAGCCTTC

AGCCGCATCTGGCTCTCCATGGTCTTTGTCTTCCGTGTGCTGGTCTACGTGGTGGCGGCC

GAGAAGGTCTGGGGCGACGAGCAGAAGGATTTTGATTGCAACACACGGCAGCCGGGCTGC

ACCAACGTGTGTTATGACCACTTCTTCCCCATCTCCCACATCCGCCTCTGGGCCCTGCAG

CTCATCTTTGTCACTTGTCCTTCCCTCCTGGTCATCATGCACGTGGCCTACAGGGAGGAC

CGCGAGAGGAAGAACCGGGAGAAGAATGGAGAGAATTGCCCCAAGCTCTACAGCAACACG

GGCAAGAAGCACGGCGGGCTGTGGTGGACCTACCTGTTCAGCCTCATCTTCAAGCTTGTC

ATAGAGATCCTGTTCCTCTACCTCCTCCACAAGATGTGGGACAGCTTCGATTTGCCACGG

CTGGTCAAGTGCTCCAACGTGGATCCCTGTCCCAACACCGTGGACTGCTACATCGCCCGG

CCAACCGAGAAAAGGGTCTTCACCTATTTCATGGTCGGAGCCTCCTCCATCTGCATTGTC

CTCACCGTCTGTGAGATCTTCTACCTCATCTTCAAGCGAGTTGTCCAGAGGGCAAGGAAA

TGGAGGAAATCCACCAAGCGCTCTGTCAGCTACAGCAAGGCCTCCACCTGCCACTGTCAC

ACCAAGTCAGAGGAGAAGGACAACAAGTCCCAGCCCAGGTGTGTGGCAGCCCTAACTGCT

GTCTGA

>Tg-gjb6-XM_002188618

ATGGATTGGGGAGCTCTGCATACCATTTTAGGAGGTGTAAATAAGCACTCCACCAGTATT

GGGAAGATATGGCTCACAGTCCTCTTCATCTTCCGTATCATGATCCTGGTTGTGGCTGCA

GAAAAAGTCTGGGGAGATGAACAAGATGACTTTGTCTGCAACACACTTCAGCCTGGTTGC

AGAAATGTTTGCTATGATCACTTTTTCCCCATCTCTCACATCAGACTCTGGGCCCTGCAG

CTGATCTTTGTCTCCACACCTGCGCTGCTGGTGGCCATGCATGTGGCTTACAGGAGGCAT

GAGAAGAAAAGGCAGTTCAGAAAAGGAGACCAGAAATGTGAATTCAAGGACATCGAAGAA

ATCAGGAAACAGAGGTTTCGTATTGAAGGCTCCTTGTGGTGGACGTACACCAGCAGCATC

TTTTTCAGACTGGTCTTTGAAGCCGTCTTCATGTACGCGTTTTATTTCATGTACGATGGG

TTCCGAATGCCTCGCTTAATGAAGTGTAATGCCTGGCCCTGCCCCAACACTGTAGACTGC

TTTGTTTCTCGACCTACTGAAAAGACAGTGTTCACTATTTTCATGATTGCTGTGTCCAGC

ATTTGCATTCTTTTAAATGTGGCTGAATTGTGTTACTTACTGACAAAGTTTTTCCTCAGA

AGGTCTAAAAGAGCTGGAAATTCGAAACAGCATCCCAACCATGAGAATAAGGAAGAAACC

AAACAAAATGAAATGAACGAGTTAATATCTGATAGCTGCCAGAACACAGTTATAGGGTTT

TCAAGTAGCTAA

>Tg-gjb7-XM_032747236-GJB7

ATGAGCTGGGCGTTCCTACGTGATCTGCTGAGTGGAGTGAATAAATACTCAACAGGAATT

GGAAGAATTTGGGTAGCTGTGGTGTTCCTGTTCCGATTACTGGTTTATCTTGCTGCCGCA

GAAAATATCTGGAAACATGAACATGATGAATTTGAGTGCAACATCAAGCAGCCTGGTTGT

GAAAATGTCTGCTTTGACCATTTCTTCCCTGTCTCTCACATCAGACTTTGGGCTCTGCAG

TTAATCATGGTCTCCACCCCTTCACTCTTGGTTGTCTTTCATGTTGCTTACAGAGAAAAC

AGAGAGAAACGCCACAACCAGAAACTTTACAAAAATACAAGAAAGACAGATGGTGGGTTG

CTGTGCACTTACCTTATCAGCCTCATTTTAAAAACAGGGCTTGAAATATTTTTTCTTGTT

CTGTTCTATAAACTGTACAACGGCTTCAAAATACCACGTCTTGTGAAATGTGACATAAAA

CCATGTCCCAATACTGTAGACTGTTACATTTCCAAACCCACAGAGAAGATGATTTTCCTC

TATTTTCTGGTGGCAACTTCATGCCTGTGCATCATATTAAATCTAAGTGAACTGAGTTAT

CTTCTGTTCAAATACTCCATAAAATGTTATCTGAAGAGACACATGAAGAACAAGCAAGGC

TCAAAAGCAATTGCTGTGAATCAGAACTCATCAGGCACAACAGAGCAGCAGCTGCAGGAT

GACTCCACAACAGCTCATCATCTTTGCCTCTGA

>Tg-gjc1-XM_030256121-GJC1

ATGAGTTGGAGTTTTCTGACCCGCCTGTTAGAGGAGATCCAGAACCACTCAACCTTTGTT

GGCAAAATCTGGCTGACAGTGTTGATTGTGTTTCGGATTGTCCTAATTGCTGTGGGAGGA

GAATCCATTTATTACGACGAACAAAGCAAGTTTGTGTGCAACACGGAGCAGCCTGGCTGC

GAGAACGTCTGCTACGACTCCTTTGCCCCTCTCTCGCACGTCAGGTTCTGGGTGTTCCAG

ATCATTCTTGTGGCCACTCCCTCCGTGCTGTACTTGGGCTATGCCATCCATAAGATCGCC

CGGATGGTGGAGCACAGCGAAGCCAGCAGGAGAGCCAGGAGGAGGAGCTTGCCCATCCGC

TGGAAACAGCACCGGGGCTTGGAGGAAGCCGAGGGTGACCACGAGGAAGACCCAATGATG

TACCCAGAGATCGAGGTGGAGAGCGTCCGGGAGAGCAAAGAGAAGCAGAGCCCTGCCAAA

GCCAAGCACGACGGGCGGCGGCAGATCCAGGAGGACGGGCTCATGAGGATTTACGTGCTG

CAGCTCCTGGCCAGGGCCCTGTTTGAAGTTGGCTTCCTGGTGGGGCAGTACTTCCTGTAC

GGCTTCCAGGTGAGCCCCGTGTTCGTGTGCAGCAGGAAGCCCTGCCCGCACAACGTCGAT

TGTTTCATCTCCAGGCCCACCGAAAAGACCATTTTCCTGCTGATAATGTACGGGGTGAGC

TGCATGTGTCTGCTGCTGAACGTCTGGGAGATGCTGCACTTGGGCTTTGGCACCATCCGG

GACACGCTGAAGGCCAAGAAGAAGGCGCTGGTTGACTCTGGGACCTTCAACTACCCCTTC

ACCTGGAACACACCCTCAGCTCCCCCGGGCTACAACATCACACTGAAGCCGGAGCAGATG

CAGTGCACGGAGCTGTCCAACGCCAGGATGGCCTACAGGCAGAACAAAGCCAACATAGCT

CAGGAACAGCAGTACGGCAGCAGCGAGGGGAACATTCCCGCCGACCTGGAGATCCTGCAG

AGGGAAATCAAAGTGGCTCAGGACCGCCTGGACCTTGCCATCCAGGCTTACAACAACCAA

AACAACCCCAGCAGCTCCAGAGGGAAGAAATCCAAAGCGGGCTCAAACAAAAGCAGTGCC

AGTAGCAAGTCAGGAGATGGGAAGAACTCGGTCTGGATTTAA

>Tg-gjc2-XM_030264133-GJC2

ATGAGCTGGAGCTTCCTCACCCGCCTGCTAGAAGAGATTCACAATCACTCCACCTTCGTG

GGGAAGGTCTGGCTCACCGTGCTCATCGTCTTCCGCATCGTCCTGACGGCGGTGGGGGGC

GAGTCCATCTACTCCGATGAGCAGAGCAAGTTCACCTGCAACACCAAGCAGCCGGGCTGC

GACAACGTCTGCTACGATGCCTTCGCGCCGCTGTCGCACGTCCGCTTCTGGGTCTTCCAG

ATCATCGTCATCTCCACGCCCTCCGTCATGTACCTGGGCTACGCCATCCACAGGATCGCC

CGCTCGGCCGAGGAGGAGAAGAAGTTCAAGGGGTTCAAGAAGAAGAAGCAGTTTGCCCTG

AACTGGCAGGCCGTGCGCAACATGGAGGATGCCATGGAGGCCGACGAGGAGGAGCCCATG

ATCTCCGACGACGCGGCCGAGCACGAGAAGGCCAAGGCCAAGCCCAAGTGCAAGGAGCAG

CAGAAGCACGACGGGAGGAGGCGCATCCAGCAGGAGGGACTGATGAAAATCTACGTCTTC

CAGCTCCTCACCCGGGCTTCCTTCGAGGTTTGCTTTTTGATAGGGCAGTATTTGCTCTAC

GGTTTCGAGGTGGAAGCTTATTATGTCTGCAACAGGGTCCCTTGTCCCCACACCGTGGAC

TGCTTTGTGTCCCGGCCCACGGAGAAGACCATATTCCTCCTGGTGATGTACGTGGTGAGC

TGCCTGTGCCTGCTGCTGAACATGTGCGAGATGTTCCACCTGGGATTCGGCACCATCCGC

GACGCCATCCGCAACCGGAAAATCAACAGCTTCCGGCAGCCCCCCTACAACTACGCCTAC

CCCAAGAACATCTCCTGCCCTCCCGAGTACAACCTGGTGGTCAAATCCGAGAAGTCCACC

AAGATCCCCAACAGCCTGATGGCCCACGAGCAGAACTTGGCCAACGTGGCTCAGGAGCAG

CAGTGCACCAGCCCCGACGAGAACCTGCCGGCGGATCTGTCCACGCTGCACAAGCACCTG

CGGGTGGCCCAGGAGCAGCTGGACATCGCCTTCCAGAGCTACAGCAGCACCCAGGCCAAC

ACCCAGCCCTCCCGGACCAGCAGCCCCGCCTCGGGGGGCACCGTGGTGGAGCAGAACAGG

GCCAACACCGCCCAGGAGAAACAAGGTGCTAAACCCAAGGCCTCCCTGGAGAAAGGCAGC

TCCAGCAGCAAAGATGGGAAGACGTCTGTGTGGATATAG

>Tg-gjc4-XM_030277371-gjc1

atgagctggagtttcctgacgcggctcctggaggagatcaacaaccactcgaccttcgtg

ggcaagatctggctgaccgtcctcattgtcttccgcatcgtgctgacggccgtgggggga

gagtccatctactacgacgagcagagcaagtttgtttgcaacacgcagcagccgggctgt

gagaacgtctgctacgacgcctttgcgcccctgtcccacgtccgcttctgggtcttccag

atcatcatggtggcgacgccatcggtgctctacctgggctttgccatgcaccgcatcgcg

cgcatgcctgagtcctcgcggcgccggccaccgtcagcccggcgggcacgcatgcccgtg

gtgcgccggggagccgggcgagactatgaggaggcagaagatgacaatgaagaagacccc

atgatctttgaggagatcgaggtggagaaggagaagagcccagagggcggagagaagcac

gatggccggcgccgtatcaagcaggacgggctgatgcgtgcctatgtgctacacttgctg

tgccgctcagtgctggagatggttttcctcttcgggcagtacctgctgtaccgctttgag

gtgagcccctcctacgtctgcagccgcagcccctgcccacacactgtcgactgctttgtc

tcccgccccactgagaagaccatcttcctcctcatcatgtacgccgtcagcgggctctgc

ctcttcctcaacctctgcgagctcttgcatcttggtgtggggcgcatccgtgatgccctg

agccaggctgatggccccccgctcccagctggcgacagtcccgcaccacaataccccaag

aaagcccccagcgccccgcccacctaccactccctgaagaaggagctgccgcaagcccca

ctgaccaacagcaagctggactaccgggagagcctggcccaggggcgcttcgccctggct

ggagctcccccggtgcacgagctggaccggctgcgtgagcacctgcggctggcccaggag

cacctggaggtggccttccacctccagcccccgccacggccccccagtcctgcccgcagc

agcagtcccgaggccaacggcatcgccgccgagcagaaccgcctcaacctcgcccacgag

aaggggaccgccgcctgcgacaggaccacggggctgtga

>Tg-gjd2-XM_030274566-GJD2 Splice site

atgggggaatggactattctagagaggctactggaagctgccgtgcagcagcattctact

atgatagggagGATCCTGCTGACCGTGGTGGTGATCTTCAGAATTCTCATTGTGGCCATT

GTAGGGGAGACGGTGTACGATGACGAGCAAACGATGTTTGTCTGTAACACGCTGCAGCCA

GGCTGCAACCAGGCTTGTTATGACCAGGCTTTCCCCATTTCTCACATAAGGTACTGGGTG

TTCCAGATCATCATGGTGTGCACTCCCAGCCTGTGCTTCATAACATACTCCGTTCACCAG

TCTGCTAAACAAAGGGAACGGAGGTACTCCACTGTCTTCCTTACCTTGGAAAGGGACCAG

GATTCAATGAAGCGTGAGGACAGTAAGAAAATCAAGAACACGATTGTCAATGGGGTGCTG

CAAAACACTGAGAACTCCACCAAAGAGGCAGAACCAGACTGCTTAGAAGTGAAGGAAATC

CCCAACCCTGCTATCAGAACTACAAAATCAAAGATGAGGAGGCAAGAAGGCATTTCTCGG

TTTTATATCATCCAAGTGGTCTTTCGAAATGCCCTAGAGATTGGATTCTTAGTGGGACAG

TATTTTCTGTACGGATTCAATGTCCCTTCCATGTACGAATGTGACAGATACCCTTGCATT

AAAGAAGTAGAGTGCTATGTTTCTAGACCCACTGAGAAGACTGTATTCTTGGTGTTCATG

TTTGCTGTCAGTGGGATTTGTGTGGTGCTTAATTTGGCAGAACTGAACCACTTGGGCTGG

AGAAAGATCAAAATGGCAGTGAGAGGAGTACAGGCAAAAAGGAAATCCATTTATGAAATC

AGAAATAAGGACCTGCCAAGAATGAGCATGCCTAACTTCGGCAGGACTCAGTCAAGTGAC

TCAGCTTATGTGTGA

>Tg-gjd3-XM_030256013-GJD3

atgggcgagtggggcttcctgagctcgctgctggacgccgtgcaggagcactcgcccatg

gtgggccgcttctggctggtggtgatgctgcttttccgcatcctggtcctggccaccgtg

ggcagcgacgtcttcgaggacgagcaggaggagttcgtgtgtaacacgcagcagccgggc

tgcaaacccgtgtgctacgacgccgccttccccatctcccactatcgcttcctcgtcttc

cacgtcgtcgtgctctcggcgcccgccgctctcttcgtcatcttcgccgtgcaccaggcg

gccaagccggggcgcgggggggctcccggccagcgcgcccgccgcctgcagcccttctac

gtgggcagcgtggtggcccggatcgccgccgagctgggcttcctgctgggccaggcgctg

ctctacggcttcagggtgcagccgctcttcgtgtgccgccgccggccctgcccgcaccgc

gtcgactgcttcgtctcccgccccaccgagaaaaccgtcttcatccacttctacttcgtg

gtggggctggtgtcggcgctgctcagcctggccgagctcgcccacctcctgcgcaagggg

cccccggcgcggccgcaggagcggggcccggcgcccgggcagccggcgggggctgcggag

ggaccctgcgctccccacccgcggggggacctcaccgtgtgacaccgcggggacgagctg

tgcccgggtt

>Tg-gjd4-XM_030264319-GJD4 Splice site

atggagcactgggactcactgggatttttgatagtcacactgaactataatgtgactatt

gtagggaagatctggctgatgttaataattctgctgcgcatggcagtggtggtattggca

ggctaccccctgtaccaagatgagcaggagcgctttgtctgcaacaccctgcaaccaggg

tgctccaacgtttgctatgacctgttctctcccgtgtctcactttaggttctggctcatt

cagaccgtggctatcctgctgccctatgctgcattcagcatttatgttttgcacaaggta

gctatgtacattgtaagaatgcactgtctggtgcatgggtgcaagaggaacaaaggttta

tcaagccccaaaggcgtgaaggagctctgtagaagtgcagttgtcaatagagtagattgt

ggtgcagacgacctaagtgtcctcaatttttcaggggcatataccattcatctttttatt

aggaccctacttgaggttgcctttgcatctgtgcaatactttctttttggattttttgtt

cctgaccgctattcatgctaccactcaccttgcacaagcactgttgactgttacatctcc

cggcccactgagaaatccatcatgatgattttcatctggggggtcagcagtctgtccttc

ctgctcagccttgctgatcttgtctgtgctctccggagaatgacagcaagaaaccaaaag

aacaagctgctggcaaacctctgcacagacaaggagtgcatgatagatcttcccccagtc

cagcacagtagctcctctccacctccaaatggggctgcaaatatctgtgagaccagtgac

gactcctgctcacttctccctgatgaggaagaagaggctgttcttcatcctggaggtgtc

cctcagcaaagtggcagcactaacctcagcagcagcagcagcagcaataagtgctgtgta

tcaggagaccttgctgttaaacaacagagcactgaagagtccttgtgtgccagcgaccgc

caaggaactccctgcagccaagtgagacccagacttcagcaagacttcattaaagatact

gccctggctttgagacctcaggttgagtttccccttggcgtttcttcctctgttgttcag

agcaagcttttagggttctacccttcaactgagataaaaagccctgatgcacaatcaaat

tatagcagcactagctgcctgaggtcaaaaaagtctgaatgggtgtag

>Tg-gje1-XM_012572489-GJE1

ATGTCCCCCAACTACATCTGGAGCTTCTCGGAGGGATGTCTCAGGCCGCCAACAGTAATT

GGACAATTCCACACTCTTTTTTTTGGCTCAATTCGAATGTTTTTCCTTGGTGTTTTGGGC

TTTGCTGTTTATGGAAATGAGGCCTTGCATTTCAGCTGTGACCCAGACAAGAGAGAGGTT

AATCTTTTCTGCTACAACCAGTTCAGACCTATAACTCCTCAGGTATTCTGGGCATTACAG

CTGGTGATTGTACTGGTACCTGGAGCCATTTTTCACCTTTATGCTGCTTGTAAGAGCATC

AAACAAGAAGATATTCTCCAAAAGTCATCCTACACTGGTTTTTATATTTTCTCTGTTTTC

TTAAGGATTGTCCTTGAAGTTGTGGCCTTTTGGCTTCAGATTCAGCTCTTTGGTTTCAAA

GTGAATGCAATCTACATGTGTGATGTGGGAGCACTGGATAAAAAGTTTAACATTACCCGA

TGTATGGTGCCAGAACACTTTGAAAAGACAATCTTTCTTATTGCAATGTACACATTTACT

GTGATTACAGTGATCTTGTGTGTTGCTGAAATTTTTGAGATCTCATGTAGAAGGCTAGGT

TTCTTAAAAACTCAGTGA

# Supplementary Figure S11. Mallard (*Anas platyrhyncos*) *gjb5*, *gjb7* and *gjd3*.

The sequences were obtained from GenBank.

>Ap-GJB5-XM_027443685

atgaactggggggtattcgaggggctcctcagcggggtcaacaagtactccacggccttc

ggccgcatctggctctccctcgtcttcatcttccgcctgctggtctacgtggtggcggcc

gagcgcgtctggagcgacgaccacaaggacttcgactgcaacacacggcagcccggctgc

accaacgtctgcttcgaccacttcttccccgtctcacacatccgcctctgggccctgcag

ctcatcctggtgacctgtccctccctgctggtcatcatgcacgtggcctaccgcgaggcc

aagcagcagaggcgccgggccgccgcggggagcgactaccgctgcctctaccccaacccc

ggcaagaaacggggtgggctgtggtggacctacctgctcagcctcatcttcaaggccggc

gtggacatcatcttcctctacatcttctaccgcttgtacaggaactacaccctgccctcc

gtggtgaagtgcgagctgcccccgtgccccaacaccgtggactgcttcatctcccggccc

accgagaagaacatcttcaccatcttcatggtggtcaccacctgcatctgcatcgcgctc

agcctggtcgaggccacctacctgatcggcaagcggtgctgtgagtgcctccaggcccgc

agcggggagagctgccagcacagccgggactgcgcgctgtcctgcgccgagcctgaggac

acagaggggcagggtatccccccggtggactgcaagccccccacaacctccatccccacg

gcttccatccccaaaacctccatccccacggcctccgtccccacagcccccatccccaca

gcctccgtccccacgacctccgtccccacatcctccgtccccaagcctgagcaggagcag

gttcctccctag

>Ap-GJB7-XM_013093494 Anas platyrhynchos

atgagctgggcattcctgcgtgacctgctgagtggagtgaataaatattcaacaggaatc

ggaagaatctgggtagcagttgtgttcatattccgcttgctggtttacgttgcggctgca

gaaaacatctggaagtacgaacacgatgaatttgaatgcaacatcaagcagcccggctgt

gaaaatgtttgctttgaccattttttccctgtctctcacatcaggctctgggctttgcag

ttaatcctggtctccaccccctcgctcttggttgttttccatgttgcttatcgagagaac

agagaaaaaaagcacaaccagacactttacaaaagcccaggtgagatagacggtgggttg

ctgtgcacttacctcatcagtcttgttttaaaaacaggatttgaaatagtttttctcatt

ctgttttacaaattgtacaacggattcaaaataccacatctcgtcaaatgcgacgtgaga

ccgtgtcctaatactgttgactgctatatctccaagccgactgagaagatgattttcctc

tactttctggtggcaacttcatgtctgtgcattgtattaaatttaagtgaactgagttac

ctcctttgcaaatactccgtgaagcgttatctgaagaggtacaccaaggaacatcgaggc

tcagcaagcggctgccacaaatcgggaaccatcagtcacaggagagcagcagctgcaaga

ccagcccatgacagccccctgtccttgcctctgaatgaacaagatggacaggaaaatatc

tccccactgacttga

>Ap-GJD3-XM_027445248

atgggcgagtggggcttcctgagctcgctgctggacgcggtgcaggagcactcgcccatg

gtgggccgcttctggctggtggtgatgctcctcttccgcatcctggtgctggccacggtg

ggcagcgacgtcttcgaggacgagcaggaggaattcgtctgcaacacccagcagccgggc

tgcaagccggtgtgctacgacgccgccttccccatctcccactaccgcttcctcgtcttc

cacgtcgtggtgctctcggcgcccgccgccctcttcgtcatcttcgccgtgcaccaagcg

gccaaaccggggcgcggggggaccccgggggaccgtgcccgtcgcctgcaggttttctac

gtgggcagcgtggtggctcgcatcgcggccgagctggctttcctgctgggccaggcgctg

ctctacggttttcgggtgcagcccctcttcgtgtgccgcagccggccctgcccgcaccgc

gtcgactgcttcgtctcccggcccaccgaaaaaaccgtcttcatccacttctacttcgtg

gtgggcttggtgtcggcgctgctcagcctggccgagctcgcccacctcctgcgcaagagc

cgagagccccgagcccccccccgaagcccccggccaggggagcgggcaccagcccccggg

cagccggcgggggtcccggaggagccgtgccccccgccacgaggagccctggctgtgtag

# Supplementary Figure S12. Atlantic canary *(Serinus canaria*) *gjd5*.

The sequence was obtained from GenBank.

>Sc-gjd1-XM030239650-gjd2like Grey font: probably intron Splice site

tcactctctctgtccctcctcaggatcctgctgactgtggtggtcattttccgcatcctg

atcgtggccatcgtgggcgagacggtctacgaggacgagcagacgatgttcatgtgcaac

acgctgcagccgggctgcaaccaggcctgctatgacaaagccttcccgatctctcacatc

cgctactgggtgttccagatcatcctggtggcacgcccagcctctgcttcatcacctac

tcggtgcaccaggcggccaagcagcgcgagcgccgctgctccttcctgcagccgctgctg

gagccgcgccggcacaaggccaacggcggcccgggcaccgagggggcacccaaggaggag

cccgagctggggctgggcatggggagggcaccgcggagctccaaggtcaaacggcaggag

ggcatctccaggttctacgtcatccaggtggtgttcaggaacgcgctggagatcggtttc

ctgggcgggcagtacttcctctacggcttcaacgttccggccatctttgagtgcgaccgc

tacccgtgcgtcaaggaggtggagtgctacgtgtcgcggcccaccgagaagagcgtcttc

ctggtcttcatgttcgccgtctcaggcgtctgcgtgctgctcaacctggccgagctcaac

cacctgggctggcgcaaggtgcgcgccgccgtgcgcggggcccgcgcgcgccgcaagagc

ctgggggaggcgcggcgcaaggagacggcggcgacggcagcggcaacggcgggccagcgc

tgggcaggacacagagcagcgagtcggcctacgtgtgagggggggaaattgggattggga

tgggagtgggattggattggattgggattgggatgggatgggatcagggccagcgctggg

caggacacagagcagcgagtcggcctacgtgtgaggggggggaactgggatgggattggg

attggagtgggattggggtgggatcaggatcgggatgggatcagagcctgtgctgggcag

gcagagcagcaagtcggcacacgtgtgaggggattgggattggggttgggattggattgg

atcaggatcgggatcaggattggagtgggattgggactggggtcaggattgggattggga

ttgaggtcaggatcagatcaagattag

# Supplementary Figure S13. Collared flycatcher (*Ficedula albicollis*) *gjc1*.

The sequence was obtained from GenBank.

>Fa-GJC1-XM_005059597

ATGAGTTGGAGTTTTCTGACCCGCCTGTTAGAGGAGATCCAGAACCACTCAACCTTTGTT

GGCAAAATCTGGCTGACAGTGTTGATTGTGTTTCGGATTGTCCTAATTGCCGTGGGAGGA

GAATCCATTTATTATGACGAACAAAGCAAGTTTGTGTGCAACACGGAGCAGCCTGGCTGT

GAGAACGTCTGCTACGACTCCTTCGCCCCTCTCTCGCACGTCAGGTTCTGGGTGTTCCAG

ATCATTGTGGTGGCCACTCCCTCGGTGATGTACCTGGGCTATGCCATCCATAAGATCGCC

CGGATGGTGGAGCACAGCGAAGCCAGCAGGAGAGCCAGGAGGAGGAGCTTGCCCGTCCGC

TGGAAACAGCACCGGGGCTTGGAAGAAGCTGAGGGTGACCACGAGGAAGACCCGATGATG

TACCCAGAGATAGAGATGGAGAGCGTCCAGGAGAGCAAAGAGAAGCAGAGCCCCGCCAAA

GCCAAGCACGACGGCCGGCGGCAGATCCGGGAGGACGGGCTCATGAGGATTTACGTCGCG

CAGCTCCTGGCCAGGGCCCTGTTAGAGGTTGGCTTCCTGGTGGGGCAGTATTTCCTGTAT

GGCTTCCAGGTGAGCCCCGTGTTCGTGTGCAGCAGGAAGCCCTGCCCGCACAACGTCGAC

TGTTTCATTTCCAGGCCGACCGAAAAGACCATTTTCCTGCTGATAATGTACGGGGTGAGC

TGCATGTGTCTGCTCCTGACTGTCTGGGAGATGCTGCACTTGGGCTTTGGCACCATCCGG

GACACGCTGAACGCCAAGAAGAAGGAGCTGGTTGACTCTGGCGCCTTTAACTACCCCTTC

ACCTGGAACACGCCCTCGGCCCCGCCGGGCTACAGCGCGGCGCTGAAGCCGGAGCAGATG

CCGTGCACGGAGCTGTCCAACGCCAGGATGGCCTTCAGGCAGAACAGGGCCAACACCGCC

CAGGAGCAGCAGTACGGCAGCAGCGAGGGCAACGTTCCCGCCGACCTGGAGATCCTGCAG

AGGGAAATCAAAGTGGCTCAGGACCGCCTGGACCTTGCCATCCAGGCTTACAACAACCAA

AACAACCCCAGCAGTTCCAGAGGGAAGAAATCCAAAGCGGGCTCCAACAAGAGCAGTGCC

# Supplementary Figure S14. Australian saltwater crocodile (*Crocodylus porosus*) connexins.

The chronology of the sequences is according to the Greek nomenclature. Size nomenclature is indicated in parentheses. GenBank accession number is given for each entry.

>Cp-gja1-XM_019537972-GJA1

atgggtgactggagtgccctaggaaaacttcttgacaaagttcaagcctattctaccgca

ggagggaaagtatggctctctgtcctcttcattttccgaatcttgttgctggggacagca

gttgaatctgcctggggagatgagcagtctgcttttcggtgtaacactcagcaacccggt

tgtgaaaatgtctgctatgacaagtcctttcccatctcccatgtccgcttctgggttctg

cagataatatttgtgtctgtgcctaccctcttgtatctggcacatgtattctatgtgatg

cggaaagaagagaaactaagcaagagagaagaagagcttaaggttgtccaaaatgatggt

gggaatgtggaaatgcacctaaaacaaatagaaattaagaaattcaagtatgggattgaa

gagcatggcaaagttaaaatgcgtgggggactgctccgtacttatgtcataagcatcctc

tttaaatctgtctttgaagtggccttcttgctgatacaatggtatgtctatgggtttagc

ctgaatgccatttacacttgtgagcgagatccatgcccccacagagtagactgcttcctt

tcccgtccaactgagaaaaccatcttcattatcttcatgctggtagtgtctttagtatca

cttgccttgaacatcattgagcttttctatgtattcttcaagggcgtcaaggatcgtgtg

aaagcaaaacctgacccttactctcccaccagtgccatgagtccctccaaggagtgtgga

tccccgaaatatgcatatttcaatggctgttcctcaccaactgctcccttgtcacccatg

tctcccccagggtacaagcttgttactggagaaaggaataattcttcctgtcgtaactac

aacaagcaggccagtgaacaaaactgggcaaattacagtgcagagcagaataggatggga

caggctggcagcaccatctccaactctcatgcccagccctttgacttccctgatgatcat

cagaacactaaaaaactggcatctgggcatgagctgcagcctctcaccactgtggaccaa

aggccacctagcagagccagcagcagagccagtagcagacctagacctgatgatctggag

atctaa

>Cp-gja3-XM_019530205-GJA3

atgggtgactggagctttctggggagactattagagaatgcacaggagcactccacggtc

attggcaaagtttggctgaccgtactgtttatcttcagaatcctggtgctgggagctgct

gctgaggaggtctggggagatgagcaatcggactttacctgcaacactcagcaacctggt

tgcgaaaatgtctgctatgacaaagccttccctatctcccatatccgtttctgggtgctg

cagatcatctttgtgtccactccaactcttatctacttgggccacgtgctccacattgtg

cgcatggaggagaagaagaaacagaaagaggaagagctgaaaaggaaggggagctgcaaa

gacatcatccatttggggacagcagctggaggcaacaacaactgtgtcagcaaaaaggag

ccacctcccaagaagaaagagaagctaccgatccgtgatgagcgtggtaagatccgcatt

gggggtgccctgctccgcacctatgtcttcaacataattttcaaaactctttttgaagtg

ggcttcattgtgggccagtatttcctgtatggctttgagctgaagcctctctaccgctgc

agccgctggccctgccccaacatcgtggactgcttcatttctcggcccactgagaagacc

attttcatcatctttatgctggtggtggcctgtgtctcactgctgctcaacatgcttgag

atctaccacctgggatggaagaagctcaagcagggcatgaccaaccactacacccctgac

actatcacagctgtggaaccagttataatagaaggagatgccaagcctgcatccatgcct

ccaccaccaccagtagtgataacagctcatgctgcaccgcctgttctgcctgacacccga

gctgtcacccctctgctcactccagctgctgtgccaccttactatgctgagtcccctgtt

aaggcacgaccatctgcctcaccttctgtggccagctatcctgtggctcccccagttgct

gaggagcagcagaaaactgccacccccaccccaacctcctcaccaatcccaacgccaacc

cccatcccaacgcccacgctggctgtcatcaattacttgaatgccagcagccaagcttca

gctgctgagcagaactgggccaacatggtagttgaggagcagaagaagccaccgccttcc

agctccgcagcttcctctacccccagcagtgtccgacaacagcttccagagacagaggag

ccagctgagcagccaccactaccaccgcctccagagctacctgtggcagcagccaacagt

gtgggcagcagcaccagcctgagcgggggaagtggaagcaagtgggatatagaaggggaa

gaggaacctttggaggaaaggcctgtttcacctgcctctcctgtatcacctgcctcacct

gtcttgcctgcatcacccatgtcacctgcctgtaccacagtggagatgcctgatccagcg

ctgctcatagacacacggcgcttaagcagggctagtaagtccagcagcagcagagccagg

tcagatgacttggctgtgtaG

>Cp-gja4-XM_019547764-GJA4

atgggagactggggcttcctggagaaactgctggaccaggtccaggagcactcgacagtg

attgggaagatctggctgacggtgctgttcatcttccggatcctcatcctgggcctggca

ggggagtcggtgtggggagatgagcagtcagacttcttgtgcaacaccaagcagccaggc

tgcaccaatgtctgctacgacaaagccttccccatctcccacatccgctactgggtgctc

cagttcctctttgtcagcacccccaccttaatctacctgggccatgtcatctacctctcc

cgcctggatgagaagctgaagcagaaagagagcgagctgcgagctgtgccaatcaaggac

ctgaaggttgagcaggccctggcagtggtagagaaaaagatttccaagatctgtgtcgca

gaggatggacacatcaagatccgggggcccctcatgtgttcctatatcaccagcgtgatt

tgcaagagcatctttgaggctggcttcctcttgggtcagtggtacctgtatggtttctcc

atgccagctgtcttcgtgtgtgagagagtcccctgccctcacagggtggactgctttgtc

tcccggcccactgagaagaccatcttcattattttcatgctggtggtgggtctcatctct

cttggtctcaacctcatggagctcatccacctctgctgcaagaacatgctcaccggctta

aagaagatctcatcccggtcccctgccagcctgggtgaggagtccttcccggatgaaaag

ctgtgtgaggtgcctggtggcccataccagcccaagcagtaccactatctgcccatgagc

acggctcaggctcctccctaccagtcttacaataagctgtccagtgagcagaactggacc

aacttcAacacggaggagaacctggccctgcgcaatggtagcagacctttgcccaacagc

tatgctgctaatgcccagtccctggaggagaagcagaccagtcgtcccagtagctccgct

tctaaaaaacagtatgtctga

>Cp-gja5-XM_019528720-GJA5

atgggtgactggagtttcctgggagagttccttgaggaggtccacaaacactccacagtg

gtggggaaagtctggctgacagtgctcttcatcttccggatgctggtgcttggcacggca

gcagagtcctcctggggagacgagcagtccgacttcatgtgtgacacccagcagcctggt

tgtgagaatgtctgctacgacaaggcttttcccatctcccatgtcaggtactgggttctt

cagatcatctttgtctctaccccatcactgctgtacatgggacatgcaatgcacaaggtg

cgcatggaagagaaaagaaaaatgaaggaggcagaactgagtaggaaggagatgaaaagt

ggtggtgactcataccaccaaaaggaatatcctgtagcagagaaagttgagtttgtctat

caagaggaatcaagtggaagaatattactcagaggcaacctgctgaacacctatgtctac

agcattttgatccgcaccgctatggaagtggccttcattgttggacaatacatcctgtat

gggatcttcctggagaccctgtacatctgtcaacgggcaccatgcccccaccctgtgaac

tgctatgtctcacggcccacagagaagaatgtcttcatcgtctttatgcttgctgtagca

gtgctgtcactgttattgagtgtagctgagctctaccacctgggttggaaaaaagcaaag

gagaaatgtgttagatcatacaaacccagtcccagtatggtcagcatcaagccagagact

gcaccacaagtggagatggcccaaagctgcactcctcctccagattttaatcagtgcttg

gcaaacgccaacgggaaattcatcagccctttcagcaacaaaatggcctctcagcagaat

acagccaactttgccactgaaagggtccataaccaagacgatgcagtcagggaagagcaa

tttattcagatcaactatgcacagagtcctgaagtagccaacgaatcttctcctcacctg

cctgccaatggctacttcaatgagaaacgccggctcagcaagaccagccgcaccagcagc

aaggctaggtcagacgacctgtcagtgtga

>Cp-gja8-XM_019528784-GJA8

atgggtgactggagcttcttggggaacattttagaacaggtgaatgagcaatccaccgtc

attgggagggtttggctcacagtgctcttcatctttcgaatcctgatcctgggaacagct

gccgaattagtctggggagatgaacagtcagactttgtgtgcaacacccaacagcccggt

tgtgagaatgtctgttatgatgaagccttccccatttcccacatccgtctctgggtcctt

cagatcatctttgtctccacaccttcgctcgtgtactttgggcacgctgtgcatcacgca

cgaatgaaggagaagaagaaagagaaggaggaagctgagagacgtcggctaggtgagatg

gagaatgaggggctaaacttagctccaatccaaatgaggggggacaagaagttcccactg

gaaggaacattattgagaacctatgtctgccacatcattttcaaaaccctcttcgaggtt

gggttcattgtaggccagtactacctgtatgggttccgaattctgcccctctaccgctgt

ggccggtggccctgtcccaaccttgtcgactgctttgtatccagacccactgagaagaca

gtcttcattatgtttatgctggcggtggcctcattatcgctcttcctcaacctcgtggag

atcagtcatttgctcatgaaaagggtgcggctggctttctggaagcctgcggagccacca

ctgggggagatctcagacaaacccctacattcccttgcagtgacttccattcagaaggtc

aagggctacaagttgctggaggaggaaaagccagtgtctcactatttccccctaaccgaa

gtgggggttgaagctagccaccttccatcctccttcaatgactttgatgagaagattggt

atggggcctctagaagaactatccagggcctttgatgagcggttaccatcctatgcccaa

acaaaagagcaggaagacagcagggaagcaggaggtgagcaagaagaggaacaagagatg

ctgaaaagagatgggcagggagaggtgaaagggttggaagagcgggagccatcagcacct

gctgaactgacagctgatatgagacccctcagcaggcttagtaaagccagcagccgggcc

aggtcagatgatttgactgtatga

>Cp-gja9-XM_019547877-GJA9

atgggagactggaatttccttggtggcattttggaggaagtccacattcattccactatt

atcggaaagatctggttaactatcctattcatatttcgaatgcttgtccttggagtggca

gctgaagatgtctggaatgatgaacagtcagaattcatatgcaatacagagcaacctggc

tgcagaaatgtttgctatgatgaggcctttcctatctctcttataagatactgggtcttg

caagttatatttgtgtcttcaccttctttggtatatatgggccatgccttatacagacta

agagcattagaaaaagagagacaaaagaaaaaggcacgggtcagactggagcttgaaggt

gttgaattagaaatgactgaaattcgaaaaagactggagagcgagcttcgtgaactgggc

caaaataagctaaacaaagcacctctaagaggctccttgcttggcacatatgtgatgcat

atttttactagatcagcagttgaagttgggtttatgattgggcagtatcttctttatggt

tttcagctcgatcccctctacaagtgtcacagagatccatgtccaaatacggttgattgt

tttgtgtcgaggccaacagagaagactgtattcatattatttatgcagtcgatagcaacc

gtgtcactctgtttaaacatattggaaattatccaccttggattcagaaaagttaaggaa

ggaatttttgggcaggatgagaaaaaggatgacattgatgacttctatgtaagtaaatca

aaggcaaactccatagtaccgcagtcttccatgggaacctcttcaactccacaaaaaaca

cttccttctgcactcagtgattacaccttgctgatggaaaagcctcctgacacagtggtc

tgtccagttttaaattcaccttctgtgttccagtctattcaaaacagccagacagaaagt

agtgataattgcctcccaattgaacaaaaaaataaacggtcagaagggatgcagacttct

aatgctgggaacagtcagcctggcaagactagctcaaatgataatgaaactttaattaaa

gtatttgagtcagaaagcatttatattcagaaacaagatgaagaaaaacttttccccaat

ggtactttttgtggtcgtatagatgctgccagaagtatgtgttcaaacagtcttgttgac

ctgccacctgggtgttctctgcaatctgatattgcatccattgcttcaaccgatggcctg

gccaagccaggtgaaactccttccttgtggaacttctctgcggtgtttgcgagtgtaggg

tctccagcaaattctcccatgggcaagaatggaaaacaacccagtttcagtggaagcaaa

gccctgcctgtttgcaagatggactgcaaaacatccagcagaccagacactcctgattct

ctgggcaagctgagctcagactccaaacagtacaaggactacgatagtcctcaggctctc

tctccctctcggcaaatgtccttggcgaggaacaccagcagcaggcgttctcctaccgat

ctccaaatctaa

>Cp-gja10-XM_019538173-GJA10

atgggggattggaacttgctgggcagcatcctcgaagaagtgcatatccactccaccatt

gtgggcaaaatctggctcaccatcctgttcatattccggatgctggtgctgggagtggct

gcagaagatgtgtgggatgatgagcaatctgagttcatttgtaacactgagcaacctggt

tgcagcaacatctgttatgataaagctttccctatctctctgatcagatattgggtgttg

caaattatctttgtctcatccccatccctcatttacatggggcatgcactttacagggta

agggcccttgagaaagagaagcagcaacagaaagcccatctcagagcccagttagaagat

gcagaccctgtcttggaagagcacaagagaatggagagggagctgaggatgttagaagac

caaaagaaagtgaacaaggcacccttgagagggtccctcctgcgcacttatgtcttgcat

atcttgacccggtcggtgttagaagtgggttttatgataggccagtatcttttatatggg

tttcaaatgtaccctctttacaaatgtactcatcctccctgccctaacacagtggattgt

tttgtgtccagaccaacagagaaaaccatctttatggttttcatgcatggtattgctgcc

gtctccctgttcttgaacatcctggagattattcatctgggtgtcaagaagataaagaaa

actcttcctggaaaccagaggaggcaaggaggcatcactgaggaagagacaagcatttat

gagtcccagaagaactcaatggtccatcaagtttgcatccttagcaactcctccccacaa

agcggctatacacttttgccagaccagcaggtggggctaccactttaccttcccctacaa

caaggatacaaagtgctccaggtgtctgctgatcagggcttgcttgcagtgaggatgggt

gccgagcaacagcagtgcagcatggagagaaacaggggcgaccagcaccacagacagtgc

caccgcgcctacagacacttggcccaccctctagcaagtcaccaggagcagagaccgctc

ccgaatcagcacgtgggacagcaccacaatgaccagcagcaagagccttcctctagcagc

ggggacacccagaagcagccccgcttgggctgtgaggactgctcagaaagtgagcagcac

acccaggagcagcccaggaaacctatttatttggctaagcccccctgtagtgccaaccag

atggagatcccccaagccatccacaatgcccttcgcaaacacaactgggtgagcagctgc

aaggaccttggggaggtttgctgtgactctccagacagcgggcactatcagggcaaccac

cactccagctttctgtctgaggtgttatctcagaaccacctggccagtgacttggacagt

tctgactccaggaatggctccggctcggaaaacaaacacggagaggagagcagcccctcc

gccaccccgccacctctttctgcaataggacgtagagtgtcaatagcaagtagcatcaag

tggccttccctctaa

>Cp-gjb1-XM_019536358-GJB1

atgaactgggccggcttgtatgcggtgctgagcggcgtgaaccgccactcgacggccatt

gggcgcatctggctctcggtcatcttcatcttccgcatcatggtgctggtggtggcagcc

gagagcgtgtggggtgacgagcgctcggccttcacctgcaacacgcagcagccgggctgt

aacagtgtctgctatgaccacttcttccccatctcccatgtgcgcctgtgggccctgcag

ctcatcctggtcaccacgccagccctgctcgtggccatgcacgtggcctaccagcagcac

caggagaagaagctgctgctgctgacgggccatggtgaccccaagcatctggaggaggtc

aagaagcacaagatgcgcatctcgggtgcgctgtggtggacctatgtcagcagtgtggcc

ttccggctcctcttcgaggctgccttcatgtacatcttctacatgctctatcctggctac

cagatggtgcggctggtcaagtgcgaggcctacccctgccccaacacggtcgactgtttc

atctcccggcccaccgagaagaccgtcttcaccgtcttcatgctgaccgcatcggccatc

tgcattgtgctcaacctggctgagctggtctatctggtggtgcgtgcctgtgcccgccgc

gcccaccacagcagcaacccctcctcagccaagagctccttctatggccacaagctctcg

tccgagtacaagcagaacgagattaaccagctgctcacggagcaggacggctcccttaag

gacatgctgcgacgcaacccgggcctgcaggaaaagggcgaccgttgctctgcctgctag

>Cp-gjb2-XM_019530204-gjb6like

atggactggggatcactgcatgccgtgttgggaggtgtgaataaacactccaccagtatc

ggaaagatctggctcactgtcctgttcgtttttcgtatcatgatccttgtagtggctgct

gagaaggtctggggcgatgaacagcaagattttatctgcaacactctgcagcctggatgc

aaaaatgtttgctatgaccactttttccccatctcccacatcagactctgggcccttcag

ctcatctttgtctcgactcctgcacttttggtggtgatgcatgttgcctacagacgccat

gagaagaagcagcagttaagaaatggggagaagtttgatattgaagagctgaaacgaaca

aggtttcatattcgggggcccttgtggtggacatacactagcagcatcttcttcagagtc

ctctttgaagccgtcttcatgtatgcattttactttatgtatgatgggtacaaaatgcca

cgcttactgaaatgtagtgcttggccctgcccaaacacagtggattgctttgtctctcga

cccactgagaaaaccatgtttactgtcttcatgcttgttgtgtctgctgtttgcatgctg

ctgaatgtgacagaattgtgttacctggtggtaaaagtttgtgtgaaagagcctaggaaa

acagcagctttaaaataa

>Cp-gjb3-XM_019547768-GJB3

atggactggaagacacttcaagccctgctaagtggggtgaacaagtactccacggccttt

gggcgcatctggctctccatagtcttcgtcttccgggtgctggtctacgtggtggctgca

gagcgtgtgtggggggatgaacagaaggattttgactgcaacaccaagcagcccggctgc

accaacgtctgctacgaccactacttccccatctcccacatccgcctctgggccctgcag

ctcatctttgtcacctgcccctctttgctggtcatcatgcatgtggcctatcgggaagac

cgggagaggaagaaccgggagaagaatggggaaggctgccccaagctctacagcaacacg

ggcaagaagcatggcgggctgtggtggacctacatactgagcctcttcttcaagctgacc

atcgagatcaccttcctctacctgctccactggatgtgggacagctttgacctgccgcgc

ctggtcaagtgcagcaacgtagacccctgccccaacatcgtggactgctacattgcacgg

cccacggagaaaaaggtcttcacctacttcatggtcggggcttcctccatctgcatcgtc

ctcacggtctgcgagatcttctacctcatcttcaagagggtggtccgccacacgcgcaag

tgggggaagacttccagcaagccctcggtcacctacagtaaagcttccacctgccggtgt

cacatcaagctggagcagcaggacagccaagtcaagaacaagcccttggatgtcctcagg

gcctctgctcccaacctgaccgtgatctga

>Cp-gjb4-XM_019547537-gjb4like First nt in first Cys codon of first conserved domain is probably wrong. We have here exchanged the C with a more likely T (red).

atgaactggtcctcactgcaggacctcctgagtggggtgaacaagtaccccacagccctg

gggtgcatctggctctctgtggtctttgtcttccgcttcctggtctatgtggtagcggcg

gagcaggtgtgggctgatgatcagcgggactttgagTgcagcactctccagcctggctgc

actaatgtctgctacaaccatttcttccccgtctcgcacatccgcctctgggccctgcag

ctcatcttcgtcacctgcccttccctcctggtgaccatgcgtgtggcttaccgggaagat

cgggagcagaagcataggaagacaggagcagggaagaacagccagcggctctatctcaac

ccaggcaagaagcagggaggcctgcggtggacttacctgctcagcctggtcttcaaggct

tccacagatgcagctttcctctacatcttccaccggctctatgccaactttgacatgcct

cgagtcacggagtgctctgtgccaccttgccctcatgtggtcgattgcttcattgcaagg

cccaccgagaagaaactcttcacctatttcatggtggccaccgctaccctgcgcattgtc

ctcaacctctgtgagatatcctacctggtcaccaagaggtgctgggagaaggtcagatct

cagagggggtcattcaggtctgcaaagagatcaacaccagcaaagtgctgcttaagcctg

atggaaacaatctag

>Cp-gjb5-XM_019547770-gjb5like

atgaactgggccgtatttgaagggctcctgagtggggtgaacaagtactccaccgtcttt

ggccgcatctggctctccctggtcttcatcttccgggtgatggtctatgtagtggcagcc

gagcgggtgtggagcgatgaccacaaggactttgactgcaacacgcgccagccgggctgc

acaaatgtctgcttcgaccactacttccctgtctcccacatccgcctctgggccctgcag

ctcatcctggtcacctgcccgtccctgctggtcatcatgcacgtggcctatcgagaagcc

caggaccagaagtgccggcaggcagaggggcagggcaaacggcggttctatcttaacccc

ggcaagaagcggggaggcctgtggtggacctacctgttcagcctcatcttcaaggccgcc

gtggatgtggtgttcctctacatcttccaccgattctacaagaactataccctgcccagc

gtggtgaggtgtgagatccccccctgccccaacatcgtggactgctacatctccagaccc

accgagaagaatatcttcaccttcttcatggtgatcacagcctgcgtgtgcatcctgctc

agcctcatggaggcctgctaccttgtggggaaaaggtgcagggagaagctgtctaagaga

aaaggcagcaggagggatgagagacttcttgacaaggactcttgctgtgcccagggcgag

caaagcagtctcgaggcgggcaggcagattttccatggggctgattacaagctacccagt

gctccaatccctgccacctcccagaagggaacagaggctgttccctaa

>Cp-gjb6-XM_019530202

atggattggggatcactgcaagctgtggtgggaggtgtgaataaacactccaccagcatt

ggaaaggtctggctcactgtcctgttcattttccgtattatgatcctcatagtggctgca

gagaaagtctggggagatgaacaagaagactttgtctgtaacacactacagcctggatgc

aaaaatgtttgttacgatcactttttccccatctctcatattagactctgggcccttcag

ttgatctttgtctcaactcctgcactgttggtggtaatgcatgttgcctacagacgacat

gagaggaagaagcaattcaggaagggagatcaaaattgtcaatataaagacattgaagag

ataagagcccaaagatttcatattgagggacccttgtggtggacatacactggcagcata

ttcttcaggttgatttttgaagctgccttcatgtatgcattttacttcgtgtatgatgga

ttccgaatgccacgtctagtgaaatgtagtgcttggccttgcccaaacacagtggactgt

tttgtttctcgacctactgaaaagacggtgttcactatttttatgattggtgtgtctggt

atttgcattatgttaaatgtggctgaattctgttatctgctgataaaatgtttccttaga

aagtctagaagagcaggatctcaaagacattcccccagccttgagaataaagaagaaact

aaacaaaatgaaatgaatgagctaatatctgatagctgtcagaacaccgttccagcattt

tcaaagtag

>Cp-gjb7-XM_019538219-GJB7

atgagctggggattcctccgagatctcttaagtggagtgaataaatattcaacagtaatg

ggccggatttggttggctattgtgttcatatttcgcctgcttgtttatatggtggcagcc

gaacatgtctggaaagatgaacagaaggagtttgagtgcaatgtccagcaacctggatgt

gaaaatgtctgctttgaccacttctttcctgtttctcacatcaggctttgggctttgcaa

ctgatcatggtttctaccccatcacttctggttgctttccatgttgcctatcgtgagagc

agagagaaaaagcacaaccagaaactttacaagaatccaggaagcatggatggtggactg

ctgtgcacttacctcatcagtctaattttcaaaacaggttttgaaattggttttctagtt

ttgttttacaagttgtatgatggatttaatgtaccacgccttatgaaatgtgatgttaga

ccatgccccaatactgtagattgctacatctccaagcctacagagaagaaaatcttcctc

gttttttgtggtataacttcatgcttatgcattgcattaaacttaagtgaattgggctat

ctgactttcaaacactctttgaagtgtctaaagaggtatgtcaagagaggtcataccttg

aaacgcgagtgtcatgagctgaaatgcactgcttacaatggaacatcagctagagttctg

acccataccaatgcttcacccatgcttttcagtctccttgataaatctgaaagctgtcct

cccgatttgtcaggataa

>Cp-gjc1-XM_019554551-GJC1

atgagttggagtttcttgacccgcctgttagaggagattcacaatcattcaacctttgtt

ggcaaaatctggttgacagtattgattgtctttcggattgttcttactgcggttggtggg

gaatccatctattatgatgaacaaagtaagtttgtgtgcaacacagaacaaccaggctgt

gagaacgtttgctatgacgcttttgctcctctttcacatgtgagattttgggtgtttcag

atcattcttgtagccactccatctgtgatgtatttgggctatgccattcataaaattgcc

agaatggttgaacatagtgaaactgacaaaagaaccagaagtaaacccttttcagtgcgc

tggaaacagcatcgtggcttggaggagacagaggatgatcatgaagaagatccaatgatg

tacccagaaatagagatagaaagtgagaaggagaacaaggaacaaccacatcccaccaaa

gctaaacatgatggcaggcgacgaattcgagaggatggactcatgaaaatctatgtgcta

caacttcttgctagaactatgtttgaagttggctttcttattggtcagtattttttatat

ggttttgaagtcagcccaacgtttatatgcagcaggaaaccatgcccgcacaagatagat

tgtttcatttcaaggccaacagaaaagaccatttttctattaataatgtatggggtgagc

tgcctatgtttatttttgaatgtctgggagatgctccatttgggatttggaactattcga

gacgcactaaacaataaaagaagagagctggaggactctggtacctataactaccctttt

acttggaacacaccatctgctcctcctggctataatattgcagtcaagccagatcaaatc

caatatactgaattgtccaatgccaaaatggcttacaagcaaaacaaagccaacatagct

caggaacagcagtatgggagcaatgaagaaaacattccagctgacctggaaaatttgcag

agggaaattaaagtggctcaggaacgcttggaccttgcaatccaggcttacaataaccaa

aacaatcccaacacttcccgagaaaagaagtcaaaagcaagctcaaacaaaagtagtgcc

agtagcaaatcaggagatgggaagacgtctgtgtggatttaa

>Cp-gjc4-XM_019554186-gjc1like

atgagctggagtttcctgacccggctcctagaggagatcaacaaccactcgaccttcgtg

ggcaagatctggctgacggtgcttatcgtcttccgcatcgtgctgacggcggtgggcggc

gagtccatctactacgacgagcagagcaagttcgtgtgcaacacgcagcagccgggctgc

gagaacgtgtgctacgacgccttcgcgcctctgtcccacgtccgcttctggatcttccag

atcatcatggtggccaccccgtccgtgctctatctgggcttcgccatgcaccgcatctcc

cgcacccctgacccgcggcgccgggcacgcatgcccgtcgtgcaccgcggcgctgggcgg

gactacgaggaggcagaggacgataatgaggaggaccccatgatcttcgaggagatcgag

gctgagcgggagaagaagaagggcggggaggacggcagcgagaagcacgacgggcgccgg

cgcatcaagcaggacgggctgatgcgggcctacgtgctgcacctgctgtgccgcgcgggg

ctggaggtggccttcctgggcgggcagtacctgctttaccagttggaggtcagcccctcc

tatgtgtgctcccgcagcccctgcccccacactgtcgactgcttcatctcgcggcccacc

gagaagaccatcttcctgctgatcatgtacgcggtcagcgggctgtgcctcctgctcacc

ctctgcgagctctgccacctgggcgtgggctgcctccgggacgcgctgggacgtggcagc

gagctcccgcccccggacgacacccgccccacgccgcactacgccaagaaggcccccagc

gcgccccccacctaccactcgctgaagaaggagcccctggccctgaaggccccgctggcc

cacgacaagctggactacggggaccccctgggtggtgtgggggcccctccaaagcacgag

ctggagcggctgtgcaagcacctgcggctggctcaggagcacctggagatggccttccac

ctgcagcccacactggagaccgccagcccctcgcgtagcagcagccccgaggccaacggc

atcgctgccgagcagaaccgcctcaacttcgcccacgaaaaggagggtggcctctgcgag

cgggtcacagggctgtga

>Cp-gjc2-XM_019529411-GJC2

atgaccaacatgagctggagtttcttaacccgtctgctagaagaaattcacaatcactcc

acctttgtggggaaggtctggctcacggtgctcattgtcttccggatcgtcctgacagct

gttgggggggagtccatctactccgatgaacagagcaagttcacgtgcaacaccaagcag

ccgggctgtgacaacgtgtgctacgatgcctttgcgccgctgtcacacgtccgattctgg

gtctttcagatcatcatgatatccaccccatccgtcatgtacctgggctatgccatccac

aggattgctaggtccacggaggaggagaagcggtttcgggggttcaaaaagaagaagaag

tttgctttgaactggcaggatgtccggaacttggaagaccccatggaagcggatgaagag

gagcccatgatctccgatgacacgggggaaaacaacgagaaggccaaagacaaggagaag

agcaaggagccgcaaaagcacgatgggaggagacgcatccagcaagaagggctgatgaag

atttacgtcttccagctccttgccagagcttcatttgaagtctgcttcttgatagggcag

tatcttctctatggcttcgaggtggaagcttactacgtctgtaaccgagccccttgccct

cacacggtggactgctttgtgtcccggccgacggagaaaaccatcttcctcctggtgatg

tacgttgtgagctgcctgtgcctgttgctcaacatgtgcgagatgttccacttggggttt

gggaccattcgagatgccattcgcaaccgaaagatcaatagcttcaggcagcccccctac

aactactcctacccaaagaacatctcctgccctccggagtacaatctggtcgtgaaagcc

gagaagtcgaccaagatccccaacagcctgatggcgcacgagcagaacttggctaacgtt

gcccaggaacagcagtgcacgagcccggacgagaacatcccgaccgacctgtccaccctc

cataaacacttgcgggtcgcccaggagcaattagacatcgcgtttcagagctataacacc

ccgcaagccaacacgcagccctctaggaccagcagcccgacttcgggtgggactgtggtg

gaacagaacagggccaacaccgctcaggagaaacaaggcacgaaacccaaagctagttca

gagaaaggcagctctagcagcaaagatggaaagacatctgtatggatatag

>Cp-gjd2-XM_019551208-GJD2

atgggggaatggaccatcctagagcgcctgctggaagcggccgtgcagcagcactccacg

atgatcgggaggatcctgctgaccgtggtggtgatcttcaggatcctcattgtggccatt

gtgggggaaacggtttacgacgacgagcagacgatgttcgtgtgtaacacgctgcagccg

ggctgcaaccaggcttgttacgaccaggcgttccctatttctcacatcaggtactgggtg

ttccagatcatcatggtttgcacccccagcctctgcttcatcacctactctgtccaccag

tccgccaagcagcgggagaggaggtactccaccgtcttcctcaccttggacagggaccag

gactccatgaaacgagacgacagcaagaagatcaagaacaccattgtcaacggagtgctg

cagaacacggagaactccaccaaagaagctgagcccgactgcctggaagtgaaggaaatc

cccaacccggccatcagaaccaccaagtccaagatgaggaggcaagaaggcatctcccgg

ttttatatcatccaggtggtctttcgcaatgcgctcgagatcggattcttagtggggcag

tacttcctctatggcttcaacgtcccttccatgtacgaatgtgacagatacccttgcatc

aaggaagtcgagtgctacgtctccagacctacagagaagactgtcttcttggtattcatg

tttgcggtgagtgggatttgtgtggtgctcaacctggctgaactgaaccacctgggctgg

agaaagatcaaaatggcggtgagaggagtacaagccaaaaggaaatccatatatgaaatc

cgaaacaaagacctgccaagaatgaacatgcctaactttggcaggactcagtcaagtgac

tcagcttatgtgtga

>Cp-gjd3-XM_019550512-GJD3

atgggggagtggggcttcctgagctcgctgctggatgcagtgcaggagcactcgcccatg

gtggggcgcttctggctgggggtgatgctgatcttccgcatcctgatcctggccacggtg

ggcggggatgtgttcgaggacgagcaggaggagttcgtgtgcaacacgcggcagccaggt

tgcaagcaggtctgctatgaccgggccttccccgtctcccactaccgcttcctagtcttc

catgtggtggtcctctcggcccccgctgtgctctttgtcatctactccatgcaccagact

gccaaaatggctagggctgggaaggttgggtcccagtgcggtgcagggccacagcccgcc

cagcggcgccgccatatccgggccgtctacgtggccaatgtcatcacacggatcctggct

gaggttggcttcctgctggggcagtggcagctgtatggcttcgaggtagagaccacgtac

gtgtgccagcgtgcaccctgcccccaccgtgtggattgcttcgtgtcccggcccaccgag

aagaccatcttcatccacttctactttgtagtgggaatggcatctgcggggctcagcctg

gtggagctggcccacctgctgtacaagggccagtgccggcgacgggaatccgtcagcccc

tcgaacccagctggtgccactgctgctgccactgaacagcaagacaactggtctaaccag

gcccaggagcaggcccagtgcttcctgccccctgagagtgggagcacccaggggtcaccc

tgcagcagtgcccccgagcactacaaggccgcagccccattccagcccaaagaccctatc

ccacacagtaaggcctccccctccatgagcagggacgatctcaccatctag

>Cp-gjd4-XM_019530902-GJD4

atggagcgctgggattcactggggtttttgattatcactctcaactacaatgtgaccatt

gttggaaagatctggctgatgttagtgatcttgctgagaatggcggtgatagtgttagca

ggatatccactctaccaagatgagcaggaacggttcatctgcaacaccttgcagcctgga

tgctccaatgtgtgctatgacattttttctcctgtctctcattttagattttggctcatt

cagacggtgtctgtactgttaccttatgctgtgttcagtgtctatgttctgcataaggta

gccatgcacctagcaagagcatactgtttgccgtgtagacataaagggattaaagacttt

gctagccacaaagctttgaaggaacccagcagaagtgctgccatcactagaccagcctgt

aagactgactacttgagcatccctgatttttctgaggcttatactgttcaccttttccta

aggacattggctgaggctgcctttggagctggacactactatctctttggattttttgtt

cccaagcgcttttcctgctaccattctccttgtacgagcatggttgattgctacatctcc

cggcccactgagaaaactatcatgatgattttcatctggggagtcagtggcctctccttt

ctccttagtcttattgacctagcttttgccatccagcgaatggcagtaagaaatcagcca

aaaaagcaactgattacaagtctctatgaagagaatgagctcaattcagacctgcctcca

actgacaagcatggaggctcttcaccttttcaggaagacagaggccatccacctccagca

atgaatgataatcccgtcagtgaaggttcttgttcactcctctctgaagaggaagaagaa

tctgcttttcatcctcacgttctctcccagcaaaccgccagctcaaaccttaacagtaac

agcaacaagccctacacagcagatggccttgccgctaatcacacgggcaatgaaacacac

ttatgtggcagcaatcaacaagggattccataccggcagtcaggacctgggtatcatcag

ggcttcaccaaagatcctgtccttgctttgagcccccaggccaagtcaccgctcggagtt

tattcctctgtagttcaaagcaagcttttagggcaatattcatcagctgagctaaaagca

gcagatgtgcaatcaaattgtagcagttctggttacttaaggtcaaaaaaatctgaatgg

gtttaa

>Cp-gje1-XM_019550162-gje1like

atggggcgccccaacaacccggcgccggggatgcggctgctcaggccgccaacagtaatt

ggtcagttccacacgcttttctttggctcagttcggatgtttttccttggcgttctgggc

tttgcagtttatggaaatgaggccttgcacttcagctgtgatccagataagagagagctt

aatctcttctgttacaatcagttcaggccaattactcctcaggtattctgggcattacag

ctagtgactgtactggtacctggagcagtgtttcatctttatgcagcatgtaaaaccatc

aatcaggaagatatcctccaaaagcctatctacactgttttttatatcctcactgttttg

ttaagggttgtccttgaagttgtgtctttttggcttcagagtcacctctttggtttccag

gtgaacccagtctacaggtgtgatgcaggagccctcaataaaaaatttaatattacccgt

tgcctggtgccagaacactttgaaaagacaattttccttattgctatgtacacatttacc

gtgattacagtggtgttgtgtgttgctgagatttttgagatcttatgtaggaggctgggt

ttcttaaacaatcagtga

# Supplementary Figure S15. Chinese alligator (*Alligator sinensis*) *gja3*.

The sequence was obtained from GenBank.

>As-gja3-XM_025196654-GJA3

atgggtgactggagctttctggggagactattagagaatgcacaggagcactccacggtc

attggcaaagtttggctgaccgtgctgtttatcttcagaatcctggtgctgggagctgct

gctgaggaggtctggggagatgagcaatcggactttacctgcaacactcagcaacctggt

tgcgaaaatgtctgctatgacaaagccttccccatctcccatatccgcttctgggtgctg

cagatcatctttgtgtccactccaacacttatctacttgggccacgtgctccacattgtg

cgcatggaggagaagaagaaacagaaagaggaagagctgaaaaggaaggggagctgcaaa

gacatcatccatttggggacagcagctggaggcaacaacaactgtgtcagcaaaaaggag

ccacctcccaagaagaaagagaagctaccgatccgcgatgagcgtggtaagatccgcatt

gggggtgccctgctccgcacctacgtcttcaacataattttcaaaactctttttgaagtg

ggcttcattgtgggccagtatttcctttatggctttgagctgaagcctctctaccgctgc

agccgctggccctgccccaacatcgtggactgcttcatttctcggcccaccgagaagacc

atcttcatcatcttcatgctggtggtggcctgtgtctcactgctgctcaacatgcttgag

atctaccacctgggatggaagaagctcaagcagggcatgaccaaccactacacccctgac

accataacagctgtggaaccagttataatagaaggagatgccaagcttgcatccatgcct

ccaccaccaccagtagtgataacagcccatgctgccctgcctgttctgcctgacactcga

gctgtcacccctctgctcactccagctgctgtgccaccttactatgctgagtcccctgtt

aaggcacaaccatctgcctcaccttctgcagccagctatcctgtggctcccccagttgct

gaggagcagcagaaaactgccacccccaccccagcctcctcaccaatcccaacgccaacc

cccatcccaacgcccacactggctgtcatcaattacttgaatgccagcagccaagcttca

gctgctgagcagaactgggccaacatggcagttgaggagcagaagaagccaccaccttcc

agctcagcagcttcctctacccccagcagtgtccgacaacagcttccagagccagaggag

ccagttgagcagccaccactaccaccacctccagagctacctgtggcagcagccaacagt

gtgggcagcagtaccagcctgagtgggggaagtggaagcaagtgggatatagaaggggaa

gaggagcctttggaggaaaggcctgtttcacccgcctctcctgcatcacctgcctcacct

gtcttgcctgcgtcacccatgtcacctgcctgtaccacagtggagatgcctgatccagcg

ctgctcatagacacacggcgcttaagcagggctagtaagtccagcagcagcagagccagg

tcagatgacttggctgtgtag

# Supplementary Figure S16. American alligator (*Alligator mississippiensis*) *gjb4*.

The sequence was obtained from GenBank.

>Am-gjb4-XM_006269885-GJB4

atgaactggtcctcactgcaggacctcctgagtggggtgaacaagtactccacagccctg

gggcgcatctggctctctgtggtctttgtcttccgcttcctggtctacgtggtagcggcg

gagcaggtgtgggctgatgaccagcgggactttgagtgcaacactctccagcccggctgc

accaatgtctgctatgaccatttcttccccatctcacacatccgcctctgggccctgcag

ctcatcttcgtcacctgcccttccctcctggtgaccatgcacgtggcttaccgggaaggc

cggaagcagaagcgtaggaagacaggagcagggaagaacagccagcagctccatctcaac

ccaggcaagaagcggggaggcctgtggtggacttacctgctcagcctggtcttcaaggct

tccacagatgcagctttcctctacatcttccaccggctctacaccaacttcgacatgcct

cgagtcatggaatgctctgtgccaccttgccctcatgtggtcgactgcttcatcgcaagg

cccaccgagaagaaactcttcacctacttcatggtggccaccgctaccctgtgcattgtc

ctcaacctctgtgagatatcctacctggttgccaagaggtgccgggagcttgccacccag

caccagaccaaggggctccctagcaagccagtagagaacgtgactaatagctggtctcat

tccacccaggagaagaaccaggttgttaatataacctcctcaggacagactacattcagg

actgtccacttgatatag

# Supplementary Figure S17. Chinese softshell turtle (*Pelodiscus sinensis*) connexins.

The chronology of the sequences is according to the Greek nomenclature. Size nomenclature is indicated in parentheses. GenBank accession number is given for each entry.

>Ps-gja1-XM_006131857-GJA1

ATGGGTGACTGGAGTGCCTTGGGCAAACTTCTTGACAAGGTTCAAGCTTATTCTACTGCA

GGAGGGAAGGTATGGCTCTCTGTCCTCTTTATTTTCCGCATCCTACTTTTGGGGACAGCA GTGGAATCAGCCTGGGGAGATGAGCAGTCTGCTTTCCGGTGCAACACTCAACAGCCTGGT TGTGAGAATGTCTGCTATGACAAGTCCTTCCCAATCTCTCATGTGCGCTTCTGGGTTCTG CAGATCATATTTGTGTCTGTACCTACCCTCTTGTATCTAGCACATGTGTTCTATGTGATG CGGAAAGAAGAGAAACTGAACAAGAAAGAAGAAGAGCTCAAGGTTGTCCAAAATGATGGT GTGAATGTGGAGATTCACCTCCAACATATACAAAAAAAGAAATTCAAGTATGGAATTGAA GAGCATGGTAAAGTTAAAATGCGTGGAGGACTGCTCCGTACTTACATCATCAGCATCCTC TTTAAATCTGTCTTTGAGGTAGCCTTCTTGGTGATACAATGGTACGTCTATGGGTTTAGC CTGAGTGCTATTTACACTTGTGAGCGATATCCATGCCCACACAGAGTGGACTGCTTCCTC TCCCGTCCAACTGAGAAAACCATCTTCATTATCTTTATGCTGGTCGTCTCTCTAGTATCG CTTGCCTTGAACATTATTGAGCTTTTCTATGTGTTCTTCAAGGGTGTCAGAGATCGTGTG AAAGGAAAACCAGACCACTACTCTCCCACCGGTACAGTGAGTCCCTCCAAGGATTGTGGA TCCCCCAAATATGCTTATTTCAATGGCTGTTCCTCTCCCACTGCTCCTCTATCACCCATG TCTCCACCAGGGTACAAGCTCGTTACTGGTGACAGGAACAATTCCTCCTGTCGTAACTAC AATAAACAAGCCAGTGAGCAAAACTGGGCAAATTACAGTGCGGAGCAGAACAGAATGGGA CAGGCTGGCAGCACTATCTCCAACTCTCATGCCCAGCCCTTTGATTTCCCCGATGATCAC CAGAACGCTAAAAAACTGGCATCGGGGCTTGAGCTGCAGCCCCTCACCCTTGTGGACCAA AGGCCACCTAGCCGTGCCAGCAGCCGAGCCAGTAGCAGACCTAGACCTGATGACCTGGAG

ATCTAA

>Ps-gja3-XM_025179383-GJA3 Likely not the suggested intron in the intracellular loop indicated by XM_014581446 (when compared with genomic sequence)

ATGGGTGACTGGAGCTTTCTGGGGAGACTATTAGAGAATGCACAAGAGCACTCCACGGTT

ATTGGCAAAGTTTGGCTGACGGTACTATTTATCTTCAGGATCCTGGTGCTGGGGGCTGCT GCTGAGGAGGTCTGGGGAGATGAACAATCGGACTTTACATGCAATACACAGCAACCTGGT TGTGAAAATGTCTGCTATGACCAAGCCTTCCCCATTTCTCACATCCGCTTCTGGGTGCTG CAGATCATTTTTGTCTCCACACCAACTCTCATCTATCTGGGCCATGTGCTGCACATTGTG CGCATGGAGGAAAAGAAGAAAGAGGCTGAGCTAAAAAAGAAGGGAAGCAGCAGGANNNNN NNNNNNNNNNNNNNNNNNNNNNNNNNNNNNNNNNNNNNNNNNNNNNNNNGTGGGAGAGGG AGTAGCAACAACAACAGCACCACTAAGGAGGAACCCCCAAAGAAGGAGAAGCCACCAATC CGTGATGAGCGGGGTAAAATCCGCATTGGGGGGGCTTTGCTCCGCACTTATGTCTTTAAC ATCATTTTCAAAACTCTGTTCGAGGTGGGCTTCATCGTGGGCCAGTATTTCCTGTATGGC TTTGAACTAAAGCCACTCTACCGTTGCAGCCGCTGGCCTTGCCCCAACACTGTGGACTGC TTCATTTCACGGCCCACTGAAAAGACCATTTTCATCATCTTCATGCTGGTGGTGGCATGT GTCTCGCTGCTGCTCAACATGCTTGAGATGTACCACCTGGGATGGAAGAAGCTCAAGCAG GGCATGACCAACCAATACAGACCTGATCCACACCCCGCTGCCATGACGCCAGCTACCACT GTGAGGGACTCCAAACCTCTGACTCTGCCACTGCCATCTCCGGCAGTGCTCCCTGCTGTC CCACCTGCTCTGTCGGATTCCCGTGCCATCACGCCACTGCTCTCGTCACAGACTGTGCCA CCTTACTATGCTGAAGCCACTGCTAGGGCACAACCAGCCACCGCAGTTTCCTTGGCTGGC TACTCCGGGGCTCTTCCATTTGCCGAGGAACTACACAAAACTGCCACTCCCATTCCAGCC TCAACCCCAATCCCAACTCCAACTCTCGTCCCAACGCCTGTGCAAGCCATCACCTGCTAC TTCAACGGCAGCAGCCAGGCCTTAGCTGCTGAGCAGAACTGGGCCAATCTGGCAGTAGAG CAGCAGAGGAAGCCACCAGCCCGCTCAGCAACCTCCTCTTCCCCCAGCAGCACCCGACAA CAATCTCCAAAGCAAGAGGAGGCCAGTGAGCTGCCGCTGCCATTGCCACCTCTTTTGCCA CCTGTAGCAGCTGTCAGTAGTAGCAGCAGCACCAGCCTGAGCAGGGGAAGCAGCAGCAAG TGGGATGCAGAAGGTGAAGAGGAGGCCACAAAGGCATGGCCATTGTCAGTTGCCTGCACC ACAGTAGAAATGCATGAGCCGCCGCAGCTCATAGACACTCGGCGTTTAAGCAGGGCTAGT AAGTCAAGCAGCAGTAGAGCCAGGTCAGATGACTTAGCTGTGTAG

>Ps-gja4-XM_006125965-GJA4

ATGGGGGACTGGGGCTTCCTGGAGAAGCTGCTGGACCAGGTCCAGGAGCACTCGACGGCG

ATTGGGAAGATCTGGCTGACGGTGCTCTTCATCTTCCGGATCCTCATCCTGGGGCTGGCC GGGGAGTCGGTGTGGGGGGATGAGCAGTCGGACTTCCTATGCAACACCAAGCAGCCGGGC TGCGTCAACGTGTGTTACGACAAAGCCTTCCCCATCTCCCACATCCGCTACTGGGTGCTG CAGTTCCTCTTCGTCAGCACCCCCACCCTGGTCTACCTGGGCCATGTCATCTACCTCTCG CAGCGGGAGGAGAAGCTGAAGCAGCGGGAGAGCGAGCTCCGGGCGGTGCAGGTCAAAGAC GTGAAGGCGGAGCAGGCGCTGGCGGCGGTGCAGAAGAAGATGGACAAGATCTGCGTGGCA GAGGACGGGCACATCAAGATCCGTGGCTCCCTGATGTGGACCTATGTCATCAGCGTGGTC TGCAAGAGCATCTTCGAGGCCGGCTTCCTCTTAGGCCAGTGGTACCTGTATGGCTTCTTC ATGCCGCCCATCTTTGTGTGTGAGAGGGACCCCTGCCCCCACAAGGTGGACTGCTTTGTC TCCCGCCCCACGGAGAAGACGATCTTCATCATCTTCATGATGGTGGTGGGGCTGATCTCC CTGGGCCTCAACCTTCTGGAGCTCTTCCACCTCTGCTGCAAAAACCTGCTCAGCAGCCTA AGGAAGGCCTCTTCCTCCTCTCCCTGCCCAGCTGGGGATGTGGACCCCTTCCCAGACAGT AAGACATGCCACTACCTCCCCCTGAGCGACAGCCACTCCCCTCCCTACCTGACCTACAAC AAGCTCTCCAGCGAGCAGAACTGGGCCAACTTCAACACCGAGGAGAACCTGGCGCTGCGC AATGGGAACAAACCCACCTCAAATGGCTACGCCCTGGCCACCCAGCCCGTGCAGGAGAGG CAGTCCAGCCGGCCCAGCAGCTCTGCCTCCAAGAAGCAGTATGTCTAG

>Ps-gja5-XM_006124245-GJA5

ATGGATAACTGGAGCTTCTTGGGCGAATTCCTTGAAGAAGTCCACAAGCATTCCACTGTG

GTGGGGAAAGTCTGGCTAACTGTGCTTTTCGTCTTCCGGATGCTGGTACTGGGCACAGCA GCGGAGTCCTCATGGGGAGATGAGCAATCTGACTTCATGTGTGACACCCGGCAGCCTGGC TGCGAGAATGTTTGTTATGATAAGGCTTTCCCCATATCTCATATTAGGTACTGGGTCCTT CAGGTCATTTTCGTCTCTACCCCTTCCCTGGTGTACATGGGGCATGCAATGCACACGGTG CGCATAGAGGAAAAGAGAAAAATAAAGGAGGCAGAAATGAGGGCAAAGGGGATTAGAAGT AGCGGTGACTCTTACCACCAACAGGAGTATCCTGCAGCAGAGAAGGCAGAGTTCTCCAGT CGGGAGGAATCAAATGGGAAAATAATACTCCAGGGTAGCTTGCTGAACACCTATGTCTAC AGCATTTTGATTCGCGTTGCTATGGAAGTGACCTTCATCGTCGGACAATACATCCTCTAT GGAATCTTCCTGAAAACCCTGTACATCTGTCACCGGGAACCCTGTCCTCACCCCGTGAAC TGCTACGTCTCGCGGCCCACAGAGAAGAATGTCTTCATCATCTTCATGCTGTCCGTGGCA GCGTTGTCACTCTTCTTGAGCCTGGCCGAACTGTACCACCTGGGGTGGAAAAAAGCGAAG GAGAAATGTGCTAAATCTTACAAACCCAGCCCCACAATGGTGACTAGCAAATTGGCGCCT GCAACCCAAGTAGAGATGGCTCAAAGTTGCACTCCCCCTCCAGATTTTAATCAATGTTTG GTCAATCCCAATGGGAAATTCATCAGCCCTTTCAGCAATAAGATGGCCTCTCAGCAGAAC ACGGCCAACTTTGCCACTGAGAGGGTCCACAGCCAAGAGGATGCAGCTGGAGAAGGCCAA TTTATCCAGATCAACTATGCACAGAGTCCTGAGGCACCCAATGACTCTGCCCCCCACCAG GTCTCCAATGGCTATTTCAATGAGAAACGCAGGCTCAGCAAGACCAGCCATGCCAGCAGT AAGGCGAGGTCAGATGACCTGTCTGTGTGA

>Ps-gja8-XR_003089595-GJA8

ATGGGTGACTGGAGTTTCTTGGGGAACATTTTAGAAGAAGTGAATGAGCACTCCACAGTC

ATCGGGAGAGTCTGGCTCACAGTGCTCTTCATTTTTCGAATCCTGATTCTGGGAACAGCT GCTGAATTTGTCTGGGGAGATGAACAGTCAGACTTTGTGTGCAACACGCAGCAGCCTGGT TGTGAGAATGTCTGTTACGATGAAGCCTTCCCCATTTCCCACATCCGGCTCTGGGTCCTG CAGATCATCTTTGTCTCTACTCCTTCACTGGTGTATGTGGGGCATGCTGTGCATCATGTG CGGATGGTGGAGAAGAGGAGAGAGAGAGAGCAGGCAGAGAGGCAACAGCAAGCTGAGATG GATGAAGAGAGGTTACCGCTAACTCCAGACCAAGAGGACATCAGGAACACCAATGAAATC AATCCCAAGGGGGCCAAGAAGTTCCGACTGGAGGGAACACTCCTGGGAACCTACATCTGT CACATCATTTTTAAAGCCATTTTCGAGGTAGGGTTCATAGTAGGTCAATACTTCTTGTAC GGCTTCCGAATTCTACCCCTGTACCGCTGTGGCCGGTGGCCTTGCCCCAACCTTGTGGAC TGCTTTGTATCCAGGCCCACTGAGAAGACAGTCTTCATTATGTTTATGCTGGCTGTAGCC TCAGTATCCCTCGTCCTCAACCTTGTGGAGATCAGTTACTTGAGCTTGAAAAAGATTCGC CTGTTTTTCAGGAGGCCCACAGAGCAACAACAGGGGGAGATCCCAGAGAAGCCCCTCCAC TCCATTGCAGTCTCTTCCATTCAGAAGGTCAAGGGCTACAAGCTGCTGGAGGAAGAGAAG CCGGTGTCCCGTTACTTACCTCTGACAAAAGTGGGGGTAGAAACTGCTCCCCCTCCATCT TCTTTCAATGACTTGGATGAGAAGCTTGGTATGGGTCCCGTAGAAGACCTTTCCAGGACC TTTGATGAGAGATTACCATCTTATGCCCAAGCAAAAGAGCAGAAGGAGGAGAAGGTAGAG GCAGGGGAAGAGGAGCAGGATCAAGAAGAAGAGGAGGAGCATCAGGAACCAGAAGAAGAG CTGGAGGTGAAGAAGATGGCAGCGGAACAGGAACTGCCAGGAGAGGAACTGCCTGCAGTG CCTGCTGAAACTGACACAAGACCTCCCAGCAGGTTAAGCAAAACCAGCAGCCGGGCCAGG TCAGATGATTTGACTGTATAA

>Ps-gja9-XM_006134273-GJA9

ATGGGAGACTGGAATTTTCTTGGTGGCATCTTGGAGGAGGTCCATATTCATTCCACTATT

ATTGGGAAGGTCTGGTTAACTATCCTATTCATATTTCGAATGCTTGTCCTAGGAGTGGCA GCTGAAGATGTCTGGAATGATGAGCAATCAGAATTCATATGCAATACAGAGCAACCTGGC TGCAGAAATGTTTGCTATGACCAGGCCTTTCCTATCTCTCTCATAAGATATTGGGTCTTG CAAGTTATATTTGTATCTTCACCTTCCTTAGTCTATATGGGTCATGCTTTATACAGACTC AGAGCCTTAGAAAAAGAGAGACAAAAGAAGAAGGCACAAGTAAGAATGGAACTTGAAGGA GTTGAACTAGAAATGATTGAAGATCGGAGAAGATTGGAACGAGAACTCCGGCAACTGGAG CAAAGAAAACTCAACAAGGCACCCCTGAGAGGCTCCTTGCTCTGCACTTATGTGATACAT ATTTTTACTAGATCTGCAGTTGAAATTGCGTTTATGATTGGCCAATATCTTCTTTATGGT TTTCAGCTCGATCCTCTTTATAAATGTGAGAGAGAGCCATGTCCAAACATAGTTGACTGT TTTATATCAAGGCCAACTGAAAAAACAGTGTTCATATTATTTATGCAGTCAATAGCAATT GTATCCTTGTTTTTAAACATCTTAGAAATTGTTCATTTAGGGGTCAAAAAAATCAAAAAG GGACTTTGTGGGCCAGATAAAAGCAGGGATGACTTTGATGATTTCTATGTTAGTAAATCC AAGAAAAACTCTGTAATACCCCACTCTTATATGGGAACATGTGCAACGCCACAAAAAATT CATCCCTCTGCCCCTATTAGTTATACCATGTTAATGGAAAAACAAATTGGGACCGCAGTC CGTGATGTTCTACATTCATCTTCTGCATTCCAATCTCTTAAAAACAACCATACTGAAAAT AGCAACAATCGCATCCATGATGAACGGGAAAGTAAATTGCCAGATGAAATGCCAACTAAT ACATTGGACTGTCCTCCTCGAAATACCTGCTCAAATAATAATGATGGCTTAAACAAGGTA TTTGGGACAGAAATTAATGGTTGTCAACAACATGATGAAAAGAAACATGGCATGCATTCT AATCTAGTTGCTGCTCCGGGTCTGTGCTTGGGAAGCCTGTCTGAATTAACATCTGGAAGT TCATTGCAACCTGATATGACTTCCACTATTTCAGTTAACTGTGTTCGAAAGCTACGTGGA GTCAGTTCCCCATGGAACTGTTCTACAGTGGTTGAGAGTGAAGGGTCACCAACAGAGTCT CCTTCCACAAACAGCAACCGAGGACAAGACAGCTTCAGTGGAAGCATAGCACAAGGCCTT TCCAGCACTGACTTAAGAAAAATCAGCAGACCAGACACTCCTGACTCCCTAGGCGAACTA AGTTCAGAATCCAAACAGGGCAGAAATTGTGAAAGTCCTCAGGCTCTCTCTCCCTCACGG CGAATGTCACTGGCAAGTAATGCCAGCAGCAGGCGTGCCCCCACCGATCTTCAGATCTAA

>Ps-gja10-XM_006133351-GJA10 Underlined: Extended until stop codon

ATGGGGGATTGGAACTTGCTGGGCAGCATCCTAGAGGAAGTGCATATCCATTCCACCATC

GTGGGCAAAATCTGGCTCACTATCCTTTTTATATTCCGCATGCTGGTGCTGGGAGTGGCT GCTGAAGATGTTTGGGATGACGAGCAGTCTGAGTTCATTTGTAACACCGAGCAACCTGGC TGCAGCAATGTCTGTTATGACAAAGCCTTCCCCATCTCTCTGATCAGATACTGGGTGCTA CAGATCATCTTTGTCTCTTCCCCATCCCTAGTCTATATGGGGCATGCACTTTATAGACTT AGGGCCCTGGAGAAAGAGAGGCAAAAGAAGAGAGCTCATCTCAGAGCCCAGCTAGAAGAG CTGGAGCCCATCCTGGAAGAGCACAAGAAAATGGAAAGGGAGTTAAGGAAGTTAGAAGAA CAGAAGAAAGTGAACAAGGCACCCTTGAGAGGGTCTCTATTGCGCACCTATGTCTTGCAT ATCTTAACCCGTTCCGTGGTGGAAGTAGGTTTTATGATAGGCCAGTATGTTTTATACGGG CTTCAAATGCATCCCCTTTACAAATGCACTCGGCCTCCCTGCCCTAACACAGTGGATTGT TTTGTGTCCAGACCCACAGAGAAGACCATCTTTATGATTTTTATGCATAGCATCGCAGCT GTCTCCCTGTTCCTGAACATCCTGGAGATTATCCACCTGGGACTAAAGAAGATAAAGACA ACCCTGCACGAGAGGCAAAGAAGAGAGACAGCGATGGCAGAGGAGGAGACAAGCATTTGC AACTTGAAGAAGAACTCGGTGGTACAGCAAGTTTGCATCATGAGAAATTCCTCCCCCCAG AATAGCTATAAACTTTTGCCCACCCAGCAGGGGGGGCTGCCTGTTTATCTGCCTCCAGCA CAAGGGTACAAAGTGCTCCAGACCCCTGCTGATCATGGCCAGCGGGCAGTAGGGATGGGT GCTGAGCAGCGCCGGTGCAGCACAGACAGGCCTAAGAGTGACCAGCACCATGGACAGGTC CCTCGCCTGTACAAGCACCCAGAGCACCCCCAGGAAAGGGAGCAGCACCACAGACAGCTC CCCAACCAGAATGTGGGACAGCCACCCAGACACCCTTCCTCCAGCAGCGAGGAGACCCAC AAACAGCCCCAGCAGAGTGTTGAGAACTGCCCAGAGAGTGAACAGCACATCCCAGAGCCG CCCAGAAACCCCGTGCAGCCAGCCAAGACCCCCACTAATGCTGGTCCACTAGAGATTTCC CAAGGCCCCCGCAACATGCTCCGCAAACACAGCCGGGTGAGCAGCTGCAAAGATTACGGG GATGATCGTGTTGACTCTCCAGACAGTGGGCACTATCAGGGCAATCGCAAGGCCAGCTTC CTGTCCAGGGTGCTGTCCGAGAGCCAGTTGGCCAGTGACACAGAGAGCTCCGATTCCAGG AATGGCTCCAGCTCTGAAGCCAAATGCAGAGAGGAGAGCAGCCCTCCCATCACCCCACCA CCCCCTTCTGCAATGGGTCGCAGGATGTCCATGGTAAGTAGAGCCAATAGTAGAGCCCTC

AATAGCTGCTAG

>Ps-gjb1-XM_006119187-GJB1

ATGAACTGGGCAGGCCTCTACGCAGTGCTGAGCGGTGTGAACCGCCACTCCACTGCCTTC

GGGCGCATCTGGCTCTCCGTCATCTTCATCTTCCGCATCATGGTGCTGGTGGTGGCTGCC GAGAGCGTCTGGGGGGACGAGAAATCTGCCTTCACCTGCAACACGCAGCAGCCGGGCTGC AACAGTGTCTGCTACGACCACTTCTTCCCCATCTCCCACGTGCGCCTGTGGGCCCTGCAG CTGATCCTGGTCACCACGCCTGCCCTCTTGGTGGCCATGCACGTGGCCTACCAGCAGCAC CAGGAGAAGAAGCTGCTGGTGCTGACCGGGCACGGGGACAGCAAGCACCTGGAGGAGGTG AAGAAGCACAAGATGCGCATCTCGGGCTCCCTGTGGTGGACGTACATCTGCAGCGTGCTG TTCCGGCTGCTCTTCGAGGCCATCTTCATGTACATCTTCTACATGATCTACCCTGGCTAC CAGATGATCCGGCTGGTCAAGTGCGACGCCTACCCCTGCCCCAACACCGTCGACTGCTTC ATTTCCCGGCCCACCGAGAAGACCATCTTCACCGTCTTCATGCTGGCCACCTCGGGAGTG TGCATCATCCTCAACATGGCGGAGCTGGTGTACCTGGTGGTGCGGGCCTGTGCCCGCCGG GCGCAGCGGGACGCCAACCCCCCCTCGGGGAAGAGCTCCTTGTACGGCCACAAGCTGTCC ACCGAGTACAAGCAGAACGAGATCAACCAGCTGCTGACGGAGCAGGACGGCTCCCTCAAG

GACATGCTGCGCCGCAACCCGGGCCTGCAGGAGAAGAGCGACCGCTGCTCCGCCTGCTAG

>Ps-gjb2-XM_006138847-gjb6like

ATGGACTGGGGAACATTGCAGAAGGTTTTAGGAGGTGTAAACAAACACTCCACCAGTATT

GGGAAGATCTGGCTCACTGTCCTCTTCATTTTCCGTGTTATGATCCTTGTGGTGGCTGCA GAGAAAGTCTGGGGAGATGAACAAAATGATTTTGTTTGCAACACTCTGCAACCTGGATGC AAAAATGTTTGCTACGACCACTTCTTCCCCATCTCTCACATTAGACTGTGGGCCCTTCAG CTCATCTTTGTCTCGACTCCTGCACTTCTCGTGGCCATGCACATTGCACACAGAAGACAC GAGAAGAAAAGGCAGCTCACAAGTGGTCACAAAGTGGATATTGAAGAACTGAATAAACAA AAGGTTCATATTCAGGGCCCCTTATGGTGGACTTACACCTGCAGCATCTTCTTCAGAATG GTCTTTGAAGCTGTCTTCATGTATGTGTTTTACGTCATGTATGATGGATACCAAATGCCT CGCTTACTGAAATGTAGTGCGTGGCCTTGCCCCAACACAGTGGATTGCTTTGTTTCTCGA CCCACTGAGAAAACTATGTTTACGATTTTCATGCTTTCTGTGTCTGGGATCTGTATGCTG CTCAATATGGCTGAATTGTGTTATCTGGTGATAAAACTATGTATGAAAGCACCTAGGAAA CTAGCAACTTTAAAATAG

>Ps-gjb3-XM_006125966

ATGGACTGGAAGACGTTACAGGGCCTGCTGAGCGGGGTGAACAAGTACTCCACGGCCTTT

GGGCGCATCTGGCTCTCCGTGGTCTTCGTCTTCCGGGTGCTGGTCTACGTGGTGGCAGCA GAGCGCGTGTGGGGGGATGAGCAGAAGGACTTTGACTGCAACACCAAGCAGCCCGGCTGC ACCAACGTTTGCTATGACCACTACTTCCCCGTCTCTCACATCCGCCTCTGGGCCCTGCAG CTCATCTTCGTCACCTGCCCTTCCCTGCTGGTGATCATGCACGTGGCCTACCGGGACGAA CGGGAGCGGAAGAACCGGGAGAAGAACGGCGAGAATTGCCCCAAGCTCTATAGCAACACG GGCAAGAAACACGGCGGGCTGTGGTGGACCTACCTGCTGAGCCTCTTCTTCAAGCTGACC ATCGAGATCACCTTCCTCTATCTCCTCCACAAGATGTGGGACAGCTTTGACCTGCCACGC CTGGTCAAGTGTGCCAACCTGGACCCCTGCCCCAACATCGTGGACTGCTACATTGCCCGG CCCACCGAGAAAAAGGTTTTCACCTACTTCATGGTGGGGGCCTCCGCCATCTGCATCGTC CTCACCGTCTGCGAGATCTTCTACCTCCTCTTCAAGCAGGCCGCCCGGAGCCTGCAAAAG TGGAAGAAGAACGCCACCAAGCACGCAATCAGCTACAGCCTGGCTTCCACCTGCCGGTGC CACATCAAGCTGGAGCAGCAAGAGGCCAAAGCCAAGAACAAGACATTGGAGGCTCTCCGG GCCTCTGCCCCCAACTTGACTTTGATCTGA

>Ps-gjb4-XM_006125970-gjb4like

ATGAATTGGTCCTCGCTCCAGGAGCTCCTAAGCGGGGTGAACAAGTACTCCACGGCCTTC

GGGCGCATCTGGCTCTCCATGGTCTTTGTCTTCCGCTTCCTGGTCTACGTGGTGGCGGCA GAGCAGGTGTGGAGCGACGAGCAGAGGGACTTTGAGTGCAACACGCGCCAGCCCGGCTGC TCCAACGTCTGCTACGACCGCATCTTCCCCGTCTCCCACATCCGCCTCTGGGCCCTGCAG CTCATCCTGGTCACCTGCCCCTCCCTGCTGGTGGTCATGCACGTGGCCTACCGGGTGGCC AAGCAGAGGAAGCACCAGGAGGCGGAGGGAGGGAACAGCCGCCGGCTCTACCCCAACCCT GGCAAGAAGCGGGGAGGTCTGTGGTGGACCTACCTGCTCAGCCTGGTCTTCAAAGTCACC GTGGACGCGACTTTCCTCTACATCTTCCACCGGCTCTACGCCAGTTTCAGCCTGCCCCGC GTGGTGCGGTGCGCCGAGGCCCCCTGCCCCAACACGGTTGACTGCTTCATCGCCAGGCCC ACCGAGAAAAGACTTTTCACCTACTTCATGGTGGCCACCACTGCCTTGTGCATCCTTCTC AGTCTCTGCGAGATGGCCTACCTGGTCACCAAACGCTGCTGGGAACTTGCCACTCGGCGC CAGGGCAGGAAGCGTGGGAGCAAACCTGCAGGCTGTTATCACTGCACGCAACAGAGCCGG GTTCTTAGTGCACCATCCTCAGGGCAGGCTCCACGTGGGTCTGTCCCTTTAGATAGTGGA GAGGGTGCTGGTGAAGAGAGAGGAGGCTCACACGTTCTCTCTCTGCACTCAGGGTCTCCG GCACAGGCTCCATCCAACAGTGCAAACTGCTCACCAGCTTACAGCCTGACTGATGAAGTA

ATCAAAGGCTGA

>Ps-gjb5-XM_006125967-gjb5like

ATGAACTGGGGTGTATTTGAAGGGATCCTGAGCGGGGTGAACAAGTACTCCACGGCCTTC

GGGCGCATCTGGCTCTCCTTGGTTTTCATCTTCCGGGTGCTGGTCTACGTGGTGGCAGCG GAGCGGGTGTGGAGTGATGACCACAAGGACTTTGACTGCAACACGCTCCAGCCGGGCTGC TCCAACGTCTGCTTCGACCACTTTTTCCCCGTCTCCCACATCCGCCTCTGGGCCCTGCAG CTCATCCTGGTCACCTGCCCCTCCCTGCTGGTGGTCATGCATGTGGCCTACCGGGAAAGC AAGGAGCAGAAAAACCAGGAGGCAAAGGGGGAGCATTGCCGCCGGCTGTACCCCAACCCC GGCAAGAAGCGCGGAGGGCTATGGTGGACCTACCTGCTCAGCCTCCTCGTGAAGGCCACC GTGGACGTGGTGTTCCTCTACATCTTCTACAGGCTGTACAAGAACTACACCCTTCCCGTC GTGGTGAAGTGCGAGATCCCCCCATGCCCCAACATCGTGGACTGCTTCATCTCCCGGCCC ACGGAGAAAAACATCTTCACCCTCTTCATGGTGATCACGGCCTGCATCTGCATCCTGCTC AACCTAGTGGAGGCCTCCTACCTGGTGGGGAAAAGGTGCCGGGAGGCCCTGCTGTCCAGG AGCGAAAGGAGCAGGCGGGAGACCAGGCGGTGCAGGAGCATCGTGCATCATGCATCGTGC GTCGACGAGCAGCACAACTTCAAGTCAGGAGGGCAAGTTCTTCCTGAAGCACGTTACAAG CCAGCCACGGTTCTGGTCCCCACTGGCTCCCAGAAGGCAGAAGAGGTTCTCTCCTAA

>Ps-gjb6-XM_006138848

ATGGACTGGGGAACGCTGCAGACCATTTTAGGAGGTGTAAACAAACACTCCACAAGTATT

GGGAAGATCTGGCTCACCATTATGTTCATTTTCCGTGTTATGATCCTCGTGGTGGCTGCA GAAGAAGTCTGGGGAGACGAACAAAATGATTTTGTCTGTAACACTCTGCAACCCGGATGC AAAAATGTTTGCTACGACCACTTCTTCCCCATCTCTCACATTAGACTGTGGGCCCTTCAG CTCATTTTTGTCTCAACTCCCGCACTTCTCGTAGCCATGCATGTTGCATACAGGAGGCAT GAGAATAAAAAGCAGTTAAGAAAGGGAGGGCAGACCTGCGAATATAAAGATATTGAAGAA ATAAAATTACAAAGATTTCCTATTCAGGGTCCTTTGTGGTGGACCTACACCAGCAGCATA TTCTTCAGAATGATCTTTGAATCCCTCTTTATGTATGCATTTTATTTCATGTATGATGGC TTCCAAATGCCACGACTAGTGAAGTGCAACGTCTGGCCTTGCCCCAACACAGTGGACTGC TTTGTCTCTCGGCCCACTGAAAAGACAGTGTTCACTATTTTCATGATTGCTGTGTCTGCT ATTTGCATTTTGTTAAATGTGGCTGAATTCTGCTATCTGCTGATGAAGGTTTTCCTTAAA AAATCCAGAAGAGCAGCATCTCCAAGACATCACCCCAACCATGAGACTAAAGAGGAAAGT AAACAAAATGAAATGAATGAGCTAATGTCTGGTAGCTGTCAGAACACAGTTTTGGGATTG

CAAAACAGCTAA

>Ps-gjb7-XM_006133323-GJB7

ATGAGCTGGGTATTCCTTAGAGACCTCCTAAGTGGAGTGAATAAATATTCTACCAGCATT

GGCAGGATCTGGCTGGCCATTGTGTTTATATTCCGCTTGTTGGTCTATGTGGTGGTAGCA GAACATGTCTGGAAGGACGAACAGAAGGAATTTGAGTGCAACATCAGGCAACCGGGTTGT GAAAACGTCTGCTTTGACCACTTCTTCCCTATATCCCATATTAGACTTTGGGCCTTGCAA CTAATCATGGTCTCCACCCCTTCTCTTTTGGTTGCTCTCCATGTTGCCTATCGAGAGAAC AGGGAAAAGCGACACAAACAGAAAATCTACAAGAACCCAGGAAGCATGGACGGTGGATTG CTGTGCACTTATCTCATCAGCCTCATTTGCAAAACAGGCTTTGAAATTGGTTTCCTGGTT TTGTTTTACAAGTTATATGGTGGATTCAATGTGCCACGCCTTGTGAAATGTGACATGAAG CCTTGTCCTAACACGGTAGATTGCTACATCTCCAAGCCCACAGAGAAGAAAGTCTTCCTC TATTTTGCATTGGTCACTTCATGCTTATGTGTGGTTTTAAATGTGAGTGAACTGAGCTAT CTGATTTTTAAATACACCATAAAATGTTTCCTCAAGAGATATGACAAGGAAGTTCCAACC TTGAAAAGAGAGTGCCAGAGGCTAAATTGTGTCCACCACAATGAAACAGCAGCTACAGCT TTGTTCCACAACAATGCTTCACCAGTTCTCATCAATATTCCAGATCAACCCGAAAAAAAC TTAGGTACTGACTTGAGAGAAGGATAA

>Ps-gjc1-XM_014570845-GJC1

ATGAGTTGGAGTTTCTTGACCCGTCTGTTAGAGGAAACCCACAATCATTCAACGTTTGTT

GGCAAAATCTGGCTGACGGTTTTGATTGTCTTTCGGATTGTCCTTACTGCTGTGGGAGGG GAATCCATCTATTATGATGAACAAAGCAAGTTTGTGTGCAACACAGAGCAACCAGGATGT GAAAATGTTTGTTACGATGCTTTTGCTCCTCTTTCACATGTGAGATTTTGGGTCTTTCAG ATCATTCTTGTAGCCACTCCCTCTGTGATGTATTTAGGCTATGCCATTCATAAAATTGCC AGAATGGTGGAACATAGCGAACCTGACAAAAGAACCAGGAGCAAAACTTTTTCAGTGCGC TGGAAACAACACCGTGGCTTGGAGGAGACAGAGGATGACCATGAAGAAGATCCAATGATG TATCCAGAAATAGAGCTAGAAAGTGAACGGGAAAACAAAGAGCAGCCAGCCCCCACTAAA GCCAAGCACAATGGAAGGAGGCGAATCCGAGAGGATGGGCTCATGAAAATCTACGTGCTG CAGCTGCTGTCTAGGACTACGTTTGAAGTTGGCTTTCTTGTTGGTCAATATTTTTTGTAT GGGTTTGAAGTCAGCCCCACATTTGTATGTAGCAGGAAACCATGCCCACACAAGATAGAT TGTTTCATTTCAAGACCCACTGAAAAGACCATTTTCCTGTTAATTATGTATGGTGTAAGC TGCCTGTGTTTACTTTTGAATGTCTGGGAGATGCTCCATTTGGGATTTGGTACAATTCGA GACACTTTAAACAATAAAAGAAAACAGCTGGAAGACTCGGGTACCTATAACTACCCTTTT ACTTGGAATACTCCATCTGCTCCTCCTGGCTATAACATTGCAGTCAAGCCAGATCAAATC CAATATACTGAATTGTCCAATGCCAAAATGGCCTACAAGCAAAACAAAGCTAATATAGCT CAGGAACAGCAGTATGGGAGCAATGAAGAAAACATTCCAGCCGACCTAGAAAATCTACAG AGGGAAATTAAAGTGGCTCAGGAACGTCTGGACCTTGCAATCCAGGCATACAATAACCAA AACAATCCCAGCAGTTCCAGAGAGAAAAAATTGAAAGCAGGCTCGAACAAAAGTAGTGCT AGTAGCAAATCGGGAGATGGAAAGACCTCTGTCTGGATTTAA

>Ps-gjc2-XM_025188058-GJC2

ATGAGCTGGAGTTTTCTCACCCGCCTGCTAGAAGAAATTCACAATCACTCCACTTTTGTG

GGGAAGGTCTGGCTCACCGTGCTGATCGTCTTCCGCATCGTCCTGACGGCCGTCGGAGGG

GAGTCCATCTACTCCGATGAACAGAGCAAGTTCACGTGCAACACCAAGCAGCCGGGCTGC

GACAATGTCTGCTACGACGCCTTTGCACCGCTGTCGCACGTCCGCTTCTGGGTCTTCCAG

ATCATCACGATCTCCACCCCTTCCGTCATGTACCTGGGCTACGCCATCCATCGAATCGCC

AGGGCTTCCGAGGAGGAGAGGAGGTTTAAGGGGCTCAAGAAGAAGAAGCGGTTTGCTTTG

CACTGGCAAGCCGTCCGTCACTTGGAAGACCCTTTGGAAGCTGACGAAGAGGATCCCATG

ATCGCCGAGAACACGGGGGAAAAGGAGGAGAAAGCCAAAGCAAAGAACGGGAAGGAGCAG

CAGAAGCATGACGGCAGGAGACGCATCCAGGAAGAAGGACTGATGAAGATTTACGTCTTC

CAACTCCTGGCCCGAGCTTCCTTTGAAATTTGTTTCTTGGTGGGGCAGTACTTTCTTTAT

GGCTTTGAGGTGGAGGCGTATTTCGTCTGCAACCGACTCCCTTGCCCTCACACTGTGGAC

TGCTTTGTGTCCAGGCCCACCGAAAAGACCATCTTCCTCCTGGTGATGTATGTTGTGAGC

TGCCTGTGTCTACTCCTCAACTTGTGCGAGATGTTTCACTTGGGGTTTGGGACCATTCGA

GATGCCATTCGCAGCAGAAAGATCAATAGTTTCCGGCAGCCTCCCTACAACTATTCCTAC

GCCAAGAATATCGCCTGCCCTCCGGAGTATAACCTGGTCATGAAATCAGAGAAGCCAGCC

AAGATCCCCAATAGCCTCCTGGCCCATGAGCAGAACTTGGCAAACGTGGCTCAAGAACAA

CAGTGCACAAGCCCAGATGAGAACCTCCCGCCGGATCTGTCCAGCCTCCATAAACATTTG

AGGGTGGCCCAGGAGCAGTTGGACACAGCTTTCCAGAGCTACAACACCGCTCAGGCCAAC

ACGCAGCCTTCCAAAACTAGCAGCCCAGCGTCTGGGGGGACGGTGATGGAACAAAACCGG

ACCAATACTGCCCAGGAGAAACAAGGTGCCAAGCCCAAAGCCAGTTCAGAAAAAGGGAGC

TCTAGCAGCAAAGATGGAAAGACTTCGGTATGGATCTAG

>Ps-gjd1-XM_014572644-gjd2like

atgggcgttggggtctttgcagcccagagcgcggccgcgggcccctgggggtctgaaaac

ccggccggcggggagacgaagcggggccgggcctttgtgacttgctggctgagcgatccc

aagctgcgctgttgccagagcccagccccaagcgccaggtgccgcaggccctgcccagct

gcccccctcccccgcag

NNgatcctgctgacggtggtggtgatcttccggatcctgatcgtg

gccatcgtgggcgagacggtgtacgaggacgagcagaccatgttcatgtgcaacaccctg

cagccgggctgcaaccaggcctgctacgaccaggccttccccatctcccacatccgctac

tgggtcttccagatcatcctggtgtgcacgcccagcctgtgcttcatcacctactccgtg

caccaggcggccaagcagcggngctacccgctgctggagaaggacgcccgcaagggccgg

gcggccaacggcatcctggtgcaccaccccggggcctcggccaaggaggagcccgactgc

ctggaggtgaaggagctgcccagcacgcccctgcgcccgcccaagagcgccaaggccaag

cggcaggagggcatctcccgcttctacatcatccaggtggtgttccgcaacgcgctggag

atcggcttcctggcgggccagtacttcctgtacggcttccacgtgccggccctcttcgag

tgcgggcgctacccctgcgtcaaggaggtggagtgctacgtctcccggcccaccgagaaa

accgtcttcctggtcttcatgttcgccgtcagcgggatctgcgtgctgctcaacctggcc

gagctcaaccacctgggctggcgcaagatccgggcggccatgcggggggtgcaggctcgc

cgcaagtccgtgtgcgaggtgcgcaagaaggacctgtccgccctggcccaggcgccgccc

ctggggcgcacccagtccagcgagtcggcctacgtctga

>Ps-gjd2-XM_006123266-GJD2

ATGGGGGAATGGACCATCCTAGAGAGACTCCTGGAGGCGGCTGTGCAGCAGCACTCGACT

ATGATAGGGAGAATCCTGCTGACCGTGGTGGTGATCTTCCGGATACTCATCGTGGCCATC GTGGGAGAAACGGTGTACGATGACGAGCAAACCATGTTTGTGTGCAACACGCTGCAGCCA GGCTGCAACCAGGCGTGTTACGACCAGGCATTCCCGATTTCGCACATCAGGTACTGGGTG TTCCAGATCATCATGGTCTGCACGCCCAGCCTCTGCTTTATCACCTACTCTGTGCACCAG TCTGCTAAGCAGCGCGAGAGGAGATACTCCACTGTCTTCCTCGCCTTGGACAGGGACCAG GACTCAATGAAACGCGAGGACAGCAAGAAGATCAAGAACACCATTGTCAACGGAGTGCTG CAGAACACCGAGAACTCCACCAAGGAAGCCGAGCCGGACTGCTTGGAGGTGAAGGAAATC CCCAACCCCGCCCTCCGAACGACAAAGTCAAAGATGAGGCGGCAGGAAGGCATCTCCAGG TTTTACATCATCCAGGTAGTTTTCCGAAATGCCCTGGAGATCGGATTCCTAGTGGGACAA TATTTCCTGTATGGATTCAACGTCCCTTCCATGTATGAATGTGATAGGTACCCCTGCATT AAAGAAGTGGAGTGCTATGTCTCCAGACCCACGGAGAAGACTGTCTTCTTGGTGTTCATG TTTGCAGTGAGTGGGATTTGTGTGGTGCTCAATTTGGCAGAACTGAATCACTTGGGCTGG AGAAAGATTAAAATGGCAGTGAGAGGAGTACAAGCGAAAAGGAAATCCATATACGAAATC AGAAACAAGGACTTGCCAAGAATGAGCGTGCCGAACTTTGGCAGGACTCAGTCGAGTGAC

TCAGCCTATGTGTGA

>Ps-gjd4-XM_006133024-GJD4

ATGGAGCGCTGGGACTCACTGGGGTTTTTGATTATCACTCTCAACTACAATGTTACGATG

GCAGGAAAGATGTGGCTTACATTAGTAATCTTGCTGAGAATGTTGGTGATAGTGCTAGCA

GGATACCCCCTCTACCAAGATGAACAAGAACGATTCATCTGTAATACCTTGCAACCAGGG

TGCTCTAATGTGTGCTATGATATTTTCTCTCCTGTCTCTCACTTTCGATTCTGGCTCATT

CAGACAGTGTCTGTTCTGCTACCTTATACTGTGTTCAGCGTCTATGTTCTGCACAAGGTA

GCCATGCATGTTGTCACCGCACATTGTTCGCCATGTAGAGATGTAGGAATCAAGACCTCA

GGTGGCCATGCAGCCTTGAATGAACCCTGCAAATATACTTCAGTCACTAGCCCAGCCTGC

ACAACAGATCATCTGAGTATACCTGATTTTTCTAGGGCATATACTGTTCATCTTTTTCTG

AGAACACTGGCAGAGGCTGGTTTTGGAGCTGGGCAGTATTATCTCTTTGGATTTTTTGTT

CCTAAGCGCTTTTCCTGTTACCAGTCTCCTTGTACCAGCACAGTTGATTGCTACATTTCC

AGGCCTACTGAGAAATCCATCATGATGATCTTTATTTGGGGGGTCAGTGGCCTCTCTTTT

CTACTTAGCCTTATTGATCTAGCCTTTGCTATCCAAAGAATGGCAGTAAGAAATGGACAA

AAGAAACTGACGGAAAGTCTCCATGCAGAGGATGAGCATAATTCAGATCTACCTCTAGCT

GGGAGGCTGAAGGATTCTCCACCATCTCCAGAGGACAGAGGCCATCTAGTGATACATGAC

GGTCATACCAATGAAGTGTCCTGTTCACTTCACTCTGAAGATGAAGAAGAGCTTCAGGTG

GAAGAGAAGATTATCTCCCAGCAAACTGCCCTCTCTAACCTCAACAGTAATAGCAATAAG

CCCTGTATAACAAAAAACATTGCTGTTAATCAAGGCAGTAATGAAGCATCCCAGTGTATA

AGTGACCAGCAGGGAATTCCAAACAGGCAGACAAGAAATGGGCAACAGCAAGCCTTCACC

AAAGAGGCCCAAGTCAAGTCTCATCTTGGAGTTTACTCTTCTGTAAGTCAGAGCAAGCTT

TTAGGGCATTATTCTTCAGCTGAGCTAAGAGCTTCTGATGGGCAATCAAGCTGTAACAGT

GCTGGCTACTTGAGATCCAAAAAATCTGAATGGGTGTGA

>Ps-gje1-XM_014580361-GJE1

ATGGGGCGCCCCAACAACACAACGCCGGGGCTGCGGCTGCTCAGGCCTCCAACAGTGATT

GGCCAATTCCATACCCTTTTCTTTGGCTCAGTTCGAATGTTTTTCCTTGGAGTTTTGGGC

TTTGCGGTTTATGGAAATGAGGCCTTGCACTTCAGCTGTGAGCCAGACAAGAGGGAAGTT

AATCTCTTCTGTTACAACCAGTTCAGACCCATAACTCCACAGGTATTCTGGGCATTACAG

CTAGTGACAGTTCTGATACCTGGAGCAGTGTTTCATCTTTATGCTGCATGTAGAAACATC

GAACAGGAAGATATTCTCCAAAAGCCCACCTATACTGTTTTTTATATCCTCTCTGTTCTG

TTAAGGATTGTCCTTGAAGTTGTGGCTTTTTGGCTTCAGAGTCATCTCTTTGGCTTTCAA

GTGAATCCACTCTACCGGTGTGATGCAGGAGCCCTCGACAAAAAATTTAATATCACCAGA

TGCATGGTGCCAGAACATTTCGAAAAGACAATTTTCCTCATTGCTATGTATACTTTCACT

GTGATTACAGTAATTTTGTGTGTTGCTGAGATTTTTGAGATACTATGTAGGAGGCTCGGT

TTTTTAAATAA

# Supplementary Figure S18. Green sea turtle (*Chelonia mydas*) *gja2*.

The sequence was collected from GenBank.

.

>Cmy-gja2-XM_007056182-gja3 Chelonia mydas green sea turtle

atgggtgactggaacctgctggggaggcttctggagagtgcccaggagcactccacagtg

gtggggaaggtctggctcactgtcctgttcattttccgtatccttgtcttgggggcagct

gccgagaaagtctggggggacgaacagtcgggcttctcctgcgacaccaagcagccaggc

tgccagaacgtctgttatgacaaaaccttccccatctctcacatccgcttctgggtgctg

cagatcatctttgtctccacccccagcctcgtgtacctgggccacatcctgcatctggtg

cgcatggcggagaaggagcagcagcgagaggagacttgggtggcccagcaggaacccctg

gtgcagcataaaccttccatcaaagatgttcatggcaaaatccgcctgcagggggccatt

ctgaggacttatatttgcaacattgtttttaagatgctctttgaggtgggcttcattgtg

ggccaatacgcactgtatgggtttgaactaaagcctctctacacgtgcaaccgctggccc

tgccccaacacggttaactgctacatagcccggcccaccgagaagaccatctttgtcttc

ttcatgacgggggtggcctgcctgtctctgctcctcaatctgatagaaatataccacctg

ggctccaccaagtgcaggcaggggttggcagccaaccactgccaggcgcccgaccccaag

gccagctccaacactcccagagaaacaagcgaccaccacattccccactgtcctgtggcc

aacagccttccccatcctgctgaccccagccttccattgcagctttacgccggccaaaca

gctccctctgcgggcagggacagccgtgcagcatggagcagccgcgagacaggcaatcag

ggcctggccatggacgaaccattgcagcggggcagcaggtccagtaaatctagcgctaag

ctgagacccagtgacttggctgtttaa

# Supplementary Figure S19. Western painted turtle (*Chrysemus picta belli*) *gja2,* *gja3* and *gjd3*.

The sequences were collected from GenBank.

>Cpb-gja2-XM_005286070-gja3like Highly deviating in the first 130 nt of the cds (grey font). This part of the sequence is not included in the analyses.

atgagagaccagccggacacctctgcccccggaagaggagctgggacacgtcctgctggg

gctgcggggatctactaccccagaggcacggggcagctgagttgcctccacggccaagga

tctgcaaacgctgccgagaaagtctggggggacgaacagtcgggcttctcctgtgacacc

aagcagccaggctgccagaacgtctgttatgacgaaaccttccccatctcccacatccgc

ttctgggtgctgcagatcatcttcgtctccacccccagcctcgtctacctgggccacacc

ctgcatctggtgcgcatggcggagaaggagcagcagcgagaggagacttggggggcccag

caggaacccctggtgcagcataaaccttccatcaaagatgttcatggcaaaatccgcctg

cggggggccattctgaggacgtatatttgcaacattgtctttaagatgctctttgaggtg

agcttcattgtgggccagtacgcactgtatgggttcgaactaaagcccctctacacgtgc

aaccgctggccctgccccaacacggtcaactgctatatagcccggcccaccgagaagacc

atctttatcctcttcatgacgggggtggcctgcctgtctctgctcctcaacctgatagaa

atgtaccacctgggcttcaccaagtgcaggcagaggctggcagccaaccgctgtcgggca

cccaaccccaaggccagctccaacgctcccagagaaacaagcagccaccacattccccac

tgtcctgcaccctacagccttccccatgccgctgaccccaaccttccattgcagccttac

gccagccaagctgctccctcagcgggcggggacagccgtgcagcacggagcagccgcgag

acaggcacccagggtccggtcatggaagaactgttgcagcagggcagcaggtccagtaaa

tccagcactaagctgagacccagtgacttggctgtttaa

>Cpb-gja3-XM_005312218-GJA3

atgggtgactggagctttctggggagactattagagaatgcacaagagcactccacggtt

attggcaaagtttggctgacggtactatttatcttcaggatcctggtgctgggggctgct

gctgaggaagtctggggagatgaacaatcggactttacatgcaacacccagcaacctggt

tgcgaaaatgtctgctatgacaaagccttccccatttcccacatccgcttctgggtgctg

cagatcatctttgtctctacgccaaccctcatctacctgggccatgtgctgcacattgtg

cgcatggaggagaagaagaaagaggaagagctaaaaaagaagggaagcagcagggacagc

aaccaccaggggggagcagcagcagcaggtggccgagggagtagcaacaacagcagcacc

aataaggagccacccccaaagaaggagaagccaccaatccgtgatgagcggggtaaaata

cgcatcagtggtgccttgctccgcacctatgtcttcaatatcattttcaaaactctgttt

gagataggcttcattgtgggccagtatttcctgtatggctttgagctgaagccactctat

cgttgcagccgttggccttgcccgaacattgtggactgcttcatttcacggcccactgag

aagaccattttcatcatcttcatgctggtggtggcatgtgtctcgctgctgctcaacatg

cttgagatgtaccaccttggatggaagaagctcaagcaaggcgtgaccaaccaataccgc

cctgatccactccccattgcaccagctaccattgcaagggactccaaacctgttactctg

ccattgccatctccggcagtgttccctactgtcccgcctgctctgccggattcccgtgcc

atcactccgctgctctcttcacggactgtgccgccttactatgctgaagccactgctagg

gcacaaccagccaccacaacttccctggctggctactccggggctcctccattttctgag

gagcgacacaatactgccactcccattccaacctccatcccaatcccaactctatccccc

atcccaacacctatgcaagccatcaccccctacttcaatggcagcagccaggccttagct

gccgagcagaactggaccaatctggcagtcgagcagcagaggaagccaccagcctccagc

tcagctgcctcctctccccccagcagcatcggacaacaatcgccaaagcaagaggtggcc

agtgagcagctgctgccatcgccacctcttttgccacctgtagcagcagtcaatagcagc

agcagcaccagcctgagcagaggaagcagcagcaagtgggatgtagaaggtgaagaggag

gccacaaaggagtggccagtgtcagctgcctgcactacagtagaaatgcatgagccgcca

ctgctcatagacacacggcgcttaagcagggctagtaagtcaagcagcagtagagccagg

tcagatgacttagctgtgtaa

>Cpb-gjd3-XM_005294117-GJD3

atgggcgagtggagtttcctgagctcgctgctggacgccgtgcaggagcactcgcccatg

gtgggccggttctggctggtggtgatgctgatcttccgcatcctcatcctggccacggtg

ggcagcgacatcttcgaggacgagcaggaggagtttgtctgcaacacgctgcagccgggc

tgcaagcaggtctgctacgacaaggccttccccatctcccactaccgcttctgggtcttc

cacatcgtggtgctctcggcgcccgccctgctcttcgtcatgtacgccatgcaccagagc

ggcaagctgcgtcagggcgaggagggcggctccgcgggcccgccccgcctgcagcccagc

cagcgcacccaccacgtccgcaccttctacctggtcaacgtggccatgcgcatcctggct

gagagcagcttcctggtggggcagtggcaactgtacgggttccacgtggagccgcacttc

acctgccagcgctcgccctgcccccactacgtggactgcttcgtgtcccggcccaccgag

aaaaccatcttcatcctcttctacttcgtggtgggcgtggtctccaccctgctcagcctg

gtggagctggcccacctgctggccaaggacaggtgccaggggacagcggccaacaagccc

ccggccgcctcctacgagcagcaggaaaactggtccaaccaggcgcacgagcagtgccag

cacttcctgcccccgggcaacgggggcgccaagggggcggcctgcagagtccccaaccac

tacgagccctcagcgctgttccggccccaagccccctccatgggcagcaaggcctcccag

gccgcggccagggccgatttggtggtctag

# Supplementary Figure S20. Green anole (*Anolis carolinensis*) connexins.

The chronology of the sequences is according to the Greek nomenclature. Size nomenclature is indicated in parentheses. GenBank accession number is given for each entry.

>Ac-gja1-XM_003215563

ATGGGTGACTGGAGTGCTCTGGGAAGGCTCCTTGATAAAGTCCAAGCCTATTCCACTGCA

GGAGGAAAGGTGTGGTTATCTGTACTTTTCATTTTCCGAATCTTGCTATTGGGAACAGCC

GTGGAGTCTGCCTGGGGTGATGAGCAGTCGGCATTCCGGTGCAACACCCAACAACCTGGT

TGTGAAAATGTCTGCTATGACAAGTCCTTCCCAATCTCTCATGTGCGCTTCTGGGTTCTT

CAGTTTATTTTTGTGTCAGTGCCCACACTCATGTACTTGGCACACGTCTTCTACGTGATG

CGAAAAGAAGAGAAGCTGAACCGAAAAGAAGAAGAACTCAAGGTAGTCCAGAGTGAGGGG

GTTAATGTAGATATCCACCTCAAACAAATAGAAATAAAGAAGTTCAAGTATGGGATTGAA

GAACATGGCAAAGTAAAAATGCGTGGGGGACTGCTCCGCACTTACATTATCAGTATTATC

TTCAAATCTTTTTTTGAAGTGGCTTTTGTGCTCATCCAGTGGTACATCTATGGGTTCACC

CTGCAAGCCATTTATGAGTGTGAACGAGTGCCATGCCCACACAAAGTGGATTGCTTCCTT

TCACGTCCCACAGAGAAAACAATTTTCATTATCTTCATGCTGGTGGTATCAATAATATCT

CTCACTCTGAACGTCATTGAAATTTTTTATGTTGTCCTCAAGAGCATTAAGGATCGCATG

AAGACAAAAAATGACCCATTCTCTCCCAACAGCGGCTTGAGTCCCTCCAAGGAATGCGGG

TCCCCTAAGTATGCCTACTTCAATGGTTGTTCCTCTCCAACTGCCCCATTGTCACCCATG

TCTCCACCCGGGACAAGGCTCGTTACTGGAGACAGGAACAATTCCTCTTCATGTCGTAAC

TACAATAAGCAAGCTAGTGAACAAAACTGGGCAAATTACAGTGCAGAGCAGAACAGGATT

GGACAAGCTGGGAGCACAATCTCCAACTCACACGCCCAGAACTTCGAATTTGCAGAGGAG

CCTGAGAACACCAAAAAAATAGGCTCTGGGCATGAGCTACAACCTCTCACAGTTGTGGAC

CAGAGGCCTCCAAGCAGAGCCAGCAGTCGAGCCAGCAGTCGACCTCGACCTGATGACCTG

GAAATCTAG

>Ac-gja2-XM_003228195-gja3

atgggcgagtggcacctgctgggccggctgctggagagtgcgcaggagcactccacggag

gtgggcaaggtctggctgacggtgctcttcatcttccgcatcctggtcctgggggcggct

gccgagaaggtctggggggacgaacagtccaacttctcctgcgacaccaagcagcctggc

tgccagagcgtctgctacgaccagaccttccccatctcccacgtccgcttctgggtgctg

cagatcattttcgtctccaccccgagcctggtctacctgggccacgtcctgcacctggtg

cgcaaggaggagagggagcggggcctctctgcggctggcaccccccttcgcgacccctgg

ggccggatccgcctgcgaggggccgtgctgcagacctacctctgcaacgtggtcttcaag

gccctgctggaggtgggcttcatcctgggccagtacctgctctacggcttcggcctgaag

cctctctacacctgcagccgctggccctgccccaacgccgtcaactgctacatctcgcgg

cccacggagaagaccatcttcattctcttcatgctggccgcggcctgcctctcgctcctc

ctcaacgtggtggaggtctgccacgtgctccgagccaaatgcagcagggcaggacggggg

tcccaggccgaatcagaggtcctggctgaggcagaagtagagaccccagcccacctccct

gggggcaaagacgtcgtcgccggcatcggaaaccccaactgggatcgacaccagaagcga

ggcagcaagcccagcaagtccagtgcgaaatggaggccgagtgatttggctgtttaa

>Ac-gja3-XM_003219245

ATGGGTGACTGGAACTTTCTGGGGAGACTATTAGAGAATGCACAAGAACACTCCACAGTT

ATTGGCAAGGTTTGGCTGACAGTACTATTTATCTTCAGGATTTTGGTCTTGGGGGCCGCT

GCTGAGGAGGTCTGGGGAGATGAACAATCAGACTTTACATGCAACACACAACAACCTGGT

TGTGAAAATGTCTGCTATGATGAAGCCTTCCCAATCTCTCATATCCGTTTCTGGGTGCTG

CAGATCATTTTTGTCTCCACGCCAACCCTCATCTACCTGGGCCATGTGCTTCACATCGTG

CGCATGGAGGAAAAGAAAAAGGAGAAGGAAGAAGAAATTAGGCAAAGGGGAAATGGCAAG

GATACCAATACTAGCCACCTCAAGGGATCCAAAGGAGAAGGGGGCAGTGATGTTAGCAGC

AACAACAATAGGGATCCACCTCCACGGAAAGAAGAGAAATCACCACTCCGTGATGAGCGT

GGCAGGATTCGGATGGCAGGTGCTCTGCTGCGCACCTATGTCTTTAACATTATTTTCAAA

ACTCTCTTTGAAGTTGCCTTTATCGTGGGCCAGTATTTCCTGTATGGCTTTCAGCTGAAG

CCACTCTACCGTTGTGGCCGCTGGCCCTGCCCAAATATTGTGGATTGTTTCATTTCAAGG

CCCACAGAGAAGACTGTCTTCATCATCTTCATGTTGGCTGTGGCTTGTCTCTCTCTGCTC

CTCAATATGTTTGAGATGTACCACTTGGGATGGAAGAAGTTCAAGCAGGGCATGACTAGA

AGATACAGCCGCACAGCACCTGCTCTGACCACGGTTGCATCCATTTCAGAAGTGGAGGAC

TCCAAACCTCTCTCCTCCTCAGCCTTGTCTCCAACTGCAGAGCCTCCGGTTGTCTCAGTC

GTTCTGCCTGAAACCCGTACCATCACCCCACTGGCTTTTCCTCAAGTCCAGCTGCCACAT

TATACTGAGGCTACCGCTAGGCCTCAGCTACCCTCTGTTACAACCTCTCTGGTTGGATAT

CCAAGCGAGCCCCCGGCTGGTGAAGGGATGTGCAAGGCCACTGTGCACACAGCAGTCTCG

ACACCTATAGCAACCACGTCCTCCATCCCTACCCCTTCCCCTGTCATCAGCTCCTACTTC

AACAGCCACACCCTGGCTCATAAGCAGAATTGGGCCAACATGGCAGTCGAGCATCAGAGG

AAAATGCTGCTTTCCAGCTCGCTTGCCTCTTCCACCCCCAGCAGCCTTCAACGATCCCAT

CCAGAACAAGAGGAAAGCTGTGAACACCCTCCACCTCCGCCTTCTTTGTCACCGGTCGCT

GCCGCCAACAGCAGCAGCGTGAGCACCAGCTTGAGTGGGGGAAGCGGCAGCAAGTGGGAG

GGAGAAGGTGAAGAAGACGAGCGGCCCATGTCAGCCACCTGCACCCTGGTGGAGATGCAT

GACCCCCCTCTGCTCATAGACTCACGTCGCTTGAGCAGAGCTAGTAAGTCGAGCAGCAGC

AGGGCCAGGTCAGATGACTTGACCATCTAA

>Ac-gja4-XM_003228180

ATGGGTGACTGGGGTTTCCTGGAGAAGCTGCTGGATCAGGTCCAGGAACATTCAACGGTC

ATTGGCAAGATCTGGTTGACGGTCCTCTTCATCTTCCGCATCTTGATCCTGGGCCTCGCT

GGGGAGTCCGTCTGGGGGGACGAGCAGTCGGACTTTGTCTGTAACACCAAGCAACCCGGC

TGCACCAACGTCTGCTACGACAAGGCCTTCCCCATCTCCCACGTCCGCTACTGGGTGCTG

CAGTTCCTCTTTGTCAGCACGCCCACCTTGGTCTATCTGGGCCACGTCATCTACATCCAC

CGGCAAGAGGAGAAGCTGAAGGAACGAGAGAACGAACTCCGGGCGCTCCAGTCCAAGGAT

TACAACGTGGCGTCCACCTTGATGGCGGTCGAAAAGAAGATGGCCAAGATCTGTGTGGCG

GAGGACGGGCGGATCAAAATCCGTGGCCCGCTGATGTGGACCTACATCATCAGCGTGGTC

TTCAAGTCCACCTTTGAGGCTGGTTTCATCCTCGGACAGTGGTACCTGTACGGTTTTGTC

ATGAGGCCTCTCTATGTCTGCGAAGCCTCCCCTTGCCCACACAAGGTGGACTGCTTCATG

TCCCGCCCCACCGAGAAGACCATCTTCATCCTCTTCATGCTGGTGGTGGCCTTGATCTCC

CTCGCCCTCAACCTTCTGGAGCTCTTCCATCTCTGTTGCAAGAGCCTCCTCAACAGCGTC

AAAGAAAGCAACTGCTGCTCTCCTTCGTCCGCTCCCCCCATGCCCTGCGACGGCAACAAT

TTCCCCACCAAACTTTGCGAGATGTCGGCCGGGCCTTATCCAGACAAGTCCTACTTCTAC

CTCCCGATGACCGAGAGGCATTCCCAGCACTACCAGGCTTACAACAAGCTTTCCAATGAG

CAGAACTGGGCCAACTTCAACACCGAGGAAACCTTGGCTTTACGAGACCACAATAAGCCG

CCGGATGTCTTTGCGGCCGAAGCTCCGAAGTCCTCGCTCTCTCAGGAAAAACAGTCCAGC

CGGCCTAGCAGCAGTGCCTCCAAGAAACAATATGTCTAA

>Ac-gja5-XM_003219088

ATGGGTGACTGGAGCTTTTTGGGAGAATTCCTGGAGGAAGTCCATAAGCATTCTACTGTG

GTGGGGAAAGTCTGGCTGACTGTGCTCTTCATCTTCCGGATGCTTGTCCTGGGCACAGCT

GCCGAGTCCTCCTGGGGCGACGAACAGTCAGACTTCATGTGTGATAGCCGGCAACCTGGT

TGTGAGAATGTCTGCTATGACCAAGCCTTCCCCATCTCCCATGTCAGGTATTGGGTCCTT

CAGATTATCTTTGTCTCCACTCCTTCCCTGCTGTACATGGGACATGCAATGCACACAGTG

CGCATGGAGGAAAAGAGGAAACTTAAGGAAGCCAAGGAAGGTTCAAAGGCAATCCAGAGC

AAGAGTGACGTTTTTCATCAACAGGAGTATCCACCGCCAGAGAAGGCTGAGCTGTCCTGC

TGGGATGATTCAAATGGGAGAATCATTCTGCAAGGCACTTTGCTGAACACGTATGTCTGT

AGTATTTTAATCCGTACTGGTATGGAAGTAGCCTTCATTGTGGGGCAGTACATGCTGTAT

GGAATCTTCCTGGAAACCCTCTATGTATGCAATCGGCAACCATGCCCTCATCCAGTCAAC

TGCTACGTTTCTCGGCCAACAGAGAAGAACATCTTTATTGTTTTCATGCTCGCTGTGGCA

GCACTCTCTCTTTTCTTAAGCCTGGCTGAACTATATCATTTGGCATGGAAAAAAGTCAAG

CAGAGGTTCTCCCGCTCTTGCAAGAACAGCCCTAGCTCAAATAATGGGATACTGGAGGCA

GAATCCCAAGGGAAAAAAATGGCACAGAGCTGCACCCCTCCTCCAGATTTTAACCAGTGT

TTGGCCAACCCTGGTGGAAAGTATATCAGCCCGTTCAGCAACAAAATGGCTTCCCAGCAG

AACACTGCTAATTTTGCAACAGAGAGAGTTCAGAGCCAAGATGATATCATTGGGGAAGGC

CCATTTATCCAAGTTAACTATACACAGAACCCTGAGGTCAGGAATAATTCTGCACCCGGC

TTGCTCTCAAATGGCTACTTGCCTGACAAACGCAGGCTTAGTAAAACCAGCCATGCCAGC

AGTAAGGCCAGATCCGATGACCTATCTGTGTGA

>Ac-gja8-XM_003219087

ATGGGTGACTGGAGTTTCTTGGGAAACATTTTGGAAGAGGTGAATGAACATTCCACTGTC

CTTGGGAGAGTTTGGCTTACGGTGCTCTTCATTTTCCGCATCCTGATCTTAGGCACTGCT

GCAGAGTTTGTATGGGGAGATGAACAGTCAGACTTTGTGTGCAACACCCAGCAGCCAGGT

TGTGAGAATGTCTGCTATGATGAGGCCTTCCCCATCTCCCATATCCGGCTATGGGTCCTG

CAGATCATCTTTGTCTCCACTCCCTCCCTAATTTACTTGGGTCACACTGCACACCATGTA

AGGATGGAAGAGAAAAAGAGAGCGCGGGAAGAAGCAGAGAGAGCTCTGCAGCAGCAAGAT

GAAAGAGAAGATGAAAAATCATCCCTTGATCCAGAGCAGAAGAGTCCAGTATCTACCAAG

GGAACCAAGAGGTTCCGCCTGGAGGGAACACTTTTGAGAACCTATATCTGCCACATCTTT

TTCAAAACCCTGTTTGAAGTAGGGTTCATAGTGGGTCAGTACTTCCTTTATGGTTTCAGA

ATTCTTCCTCTGTACCGCTGTGGTAGGTGGCCTTGCCCCAACCTTGTAGACTGTTTTGTG

TCCAGACCTACAGAAAAGACAATTTTCATCATGTTTATGTTGGTTGTGGCATCAGTTTCC

CTCTTCCTCAACATTGTGGAGATCAGCCACTTGGGTCTTAAGAAGATTCTGAATGCCTTC

AGGAAAACACGTCAACAACGGTCTGTAGAAACCCCAGAGAAGCCCCTTCACTCTATTGCT

GTTTCATCCATTCAAAAAGTCAAAGGTTACAAGCTGCTGGAGGAGGAGAAGCCAGCATTC

CACTACTTCCCTTTGACAGAAGTGGGAGCAGAAACCACACCTGTTCCTCCTTCACTGAAT

GAGTTTGAGGAGAAGATGAGCATGGAACACCTGGAGGACATTCCCAGTGCCTTTGAGGAG

CAGTTGCCATCCTATGTCCAACGGGATGAACAGGAAGAGGAGAAAGTGGAACAACCAGAA

CAAGAGGTGGCTGAAGAAGTCCAAGATGAGCAAGGAGCGGAGGGATTTAAAGAGAAAGCA

GGAGTAAAAGTAGGAGAAGAGAAGCACATGGAGGATGAGCAAGAGCCTCTAGGAGGAAAT

GTGGCTCTACCATCTACAGCTGATACAAGACCTCTTAGCAGGTTAAGCAAAAGTAGCAGT

CGGGCCAGGTCAGATGATCTGACTGTATGA

>Ac-gja10-XM_008119405

ATGGGGGACTGGAACCTGCTGGGCAGCATCCTAGAAGAAGTTCACATCCATTCAACTATA

GTGGGCAAAATTTGGCTCACCATCCTTTTCATATTCCGGATGTTGGTTCTCGGTGTTGCT

GCTGAAGATGTCTGGGATGATGAGCAAGCAGAGTTCATCTGCAACACAGAGCAGCCCGGT

TGCAGCAACATCTGTTATGACAAAGCTTTCCCCATCTCCCTGATCCGCTACTGGGTGCTA

CAAGTGATATTTGTATCATCCCCTTCCCTGGTTTACATGGGACACGCACTTTACAGACTT

CGGGCCCTCGAGAAAGAGAGGCAACGGAAGAAAGCCTACCTCAAAGCCCAGCTAGAGGAG

ATTGAGCCTACAATAGAAGATCATCGGAGGATAGAAAAAGAGCTGAGAAAGTTAGAAGAA

CAAAAGAAAGTCAACAAGGCACCCTTGAGAGGAGCCTTGTTGTGTACCTATATTCTGCAT

ATCTTGACCCGATCAGTCTTAGAAGTGGGCTTTATGGTGGGTCAGTATCTATTATATGGC

TTTCAGATGTATCCACTATATAAATGTACCCGTCCCCCTTGTCCTAACACAGTGGATTGT

TTTGTATCCAGACCAACTGAAAAGACCATCTTCATGGTTTTCATGCACACCATCGCAGCT

GTTTCTCTCTTTCTGAATGTCCTGGAGATCTTCCATCTGGGGATTAAGAAGATTAAAAAT

ACTCTTTGTGGGAGGCTAAAGGAGGATCTTGTAGTTCCTGATGAGGATGCTAACTTTTCC

AACTCCAAGAAAAATTCAGTAATACAACAAGTTTGCATCATGTCAAACTCATCTCCCCAA

AGCAGTTTTAAATTTATGTCAGAGCAACAAGGAGGGCTGCCAGTTTACTTGAATCCAGAA

GATGGATACAATCCTACAGGACAAAATCAAAATGTAGAGTCACAGTGGTGTAGCAACCAA

AGAAGTAGGTCCAATCAGCACCAGGACCAAATCCACCAGGCCTACAGACATCTCGATCAT

CCCAATGAAGGTAACCAGCACCATAATAGACATCTCCCTAGGCACCCCCCAAACCTTATG

CCTCATCGACATGACCATCAACATCAAAATGCATCTTCCAGCAGTGATGACATGCAGAAG

GCACCACAGCAGGGAGCAAACAACTATCGAAGAAATGAACAACATGCAGTGGAACATTCC

AAGGCCTCTCAACATGGCACAAAAGCACCTGTTGTCTCCAGTCATACGGAGAGCCCACAA

GCTGTTCTTCGGAAGCAAAACCGGGTGGCGGCTTGCAAGGACTTGGGAGATGATCGTTGT

GATTCTCCAGATAGTGGGCCTTATCAGAACCGTAAGTCAAGTTTTTTGTCCAGGGTACTG

TCCCAGAGCCACTTGGACACCGACTCAGAGACTTCAGACTCACGGAATGGCTCTGGTTCA

GATGCTAAACGCAGAGGTGACAGCAGTCCTCCCAACACACCACCTCTTTCTTCCACTGCT

GGACGCAGAATGTCAATGGCAAGTAGACCCAAGCGGCCTTCCTACTATACCCTGCTAGTG

TGA

>Ac-gjb1-XM_008121766

ATGAAGTGGGAGGGCTTCTACGCTATCGTGATCGGAGTGAACCGCCATGCCACGGCCATC

GGGCGCATCTGGTTTTCCGTGGTCTTCATCTTCCGCATCATGGTGCTGGTGGTGGCGGCC

GAGAGCGTCTGGGGTGACGAGCAGTCCTCCTTCACCTGCAACACGCAGCAGCCCGGTTGC

AAGAACGTCTGCTATGACTACTACTTCCCCATCTCCCACATCCGCCTCTGGGCCCTGCAG

CTCATCCTGGTGACCACACCGGCCTTGCTGGTGGCCATGCACGTGGCCTACCAGCAGCAC

CAAGAGAAGAAGTTCCTGGTCATGACCGGCCATGCCGACGCCAAGCACCTGGAGGAGGTC

AAGAAGCACAAGATGCGGATCTCTGGCTCGCTGTGGTGGACCTACGTCTTCAGCGTGATC

TTCCGCATCATCTTCGAGGGCACCTTCATGTACATCTTCTACATGGTCTACCCGGGCTAC

CAGATGATCCGGCTGGTCAAGTGCGACGTCTACCCGTGCCCCAACACGGTCGACTGCTTC

ATCTCGCGGCCCACCGAGAAAACCATCTTCACTGTCTTCATGCTGGCCACCTCCGGCATC

TGCATCGTCCTCAACGTGGCCGAGGTGGTGTACCTCCTCATCCGGGCCTGCGCTCGCCGG

GCCCAGAAGCCCCACAACCCTTCCTCGGGCAAGGGCTCCTTCCTGGGCCACAAGCTCTCC

TCTGAGTACAAGCAGAACGAGATCAACCAGCTGCTGACGGAGCAGGACGGCTCCCTCAAG

GACATCCTCCGGCGAAACTCGGGGCTCCAGGAGAAGGGGGATCGCTGCTCGGCCTGCTGA

>Ac-gjb3-XM_0081217749-gjb3like

ATGGACTGGAAGACGTTGCAAGGCTTGCTGAGCGGCGTGAACAAGTATTCCACGGCTTTT

GGGCGCATCTGGCTCTCGGTGGTCTTTGTCTTCCGGGTCCTGGTTTACGTAGTGGCGGCA

GAACGCGTCTGGGGAGACGAGCAGAAGGACTTTGACTGCAACCACCGGCAACCTGGGTGC

ACCAACGTCTGCTACGACCACTACTTCCCCATCTCCCACATCCGCCTGTGGGCCTTGCAG

CTGATTTTCGTCACCTGCCCTTCATTGCTGGTCATCATGCACGTGGCTTATCGCGAGGAC

AGGGAACGCAAGAACCGAGAGAAGAACGGGGAGAACTGTCCCAAACTCTACAGCAACACG

GGCAAGAAACACGGCGGCCTTTGGTGGACTTATCTCCTCACCCTTTTCTTCAAACTGGGC

ATCGAGATCGTCTTCCTCTATCTCCTGCATCGGATATGGGACAGTTTTGACCTGCCACGC

TTGGTCAAGTGTGACTTCCACCCGTGCCCAAACACAGTAGACTGTTACATCGCTCGGCCG

ACAGAGAAGAAGATCTTCACCTACTTCATGGTTGGTGCCTCTGCCCTTTGCATCGTCCTC

ACCGTGTGCGAGATCTTCTATCTCATCTTCAAGCGAGTGGTCCGCTCGATGCAGAAGCTG

AAGAAGAAGAAGCGGTCGCTCACCTACAGCAAGGCCTCCACCTGCCCATGCCATTCAAGA

TTGGATCATGATGGGAAGTTGAAGATCTCACCTGTGGAAGCCTTGAGAGCATCTGCACCC

AACCTGACTTTAATCTGA

>Ac-gjb4-XM_003228183-gjb4like

ATGAACTGGTCCTCATACCGAGAACTCTTGAGTGGCGTGAACAAGTACTCCACCGTTGTG

GGCCGCGTGTGGCTGACGGTAGTCTTCGTCTTCCGTTTCCTTGTCTATGTGGTGGCAGCT

GAAGACGTCTGGTCTGAAGACCAACAGGACTTTGTGTGTAACACGGGTCAACTTGGCTGC

ACCAAGAGCTGCTACAACCAGTTCTTCCCTATCTCTCACATCCGTCTCTGGGCCCTCCAG

CTTATCCTTGTGACCTGCCCTTCTCTTCTCGTTCTCCTGCACGTGACCTACCGGGAAGCC

CAGCAGCAGAAGCACATTGAGGAAGCTGGAAAACGCTGCCGTTCTCTCTACTCCAACTTC

AGGAAGAGTCACGGAGGTCTTTGGTGGACCTACTTCCTCAGCATCCTTGTCAAGGTGGCC

GTAGACCTCACCTTCCTCTACATCTTCCACTGCCTCTACCCTAACTTCAAGCTGCCCACA

ATGGTGACCTGTGATGCCTTCCCCTGTCCCAATGTAGTCACCTGCTTCATTTCCAAGACA

ACAGAAAAGAATATCTTCTCATACTTCATGGCAGCTATTGCCATGCTCTGTGTCATTCTC

AACATCTGCGAGGTGGGCTACGTAGTAGTCAAGAAATGCACCAAGATTTCAGTCTCCAAG

AAGCCAGTGGACGTTTTTGAAACAGACACTCACATCTCCTTTTAG

>Ac-gjb5-XM_008121748

ATGAACTGGTCAGGCTTTGAAGATCTCCTTGGCGGGGTGAACAAATACTCTACCGCCTTT

GGGCGCATCTGGCTCTCGGTGGTCTTCATCTTCCGCCTCCTTGTCTACTTAGTGGCGGCG

GAGAAGGTTTGGGGAGATGACTCCAAGGACTTTGACTGCAACACGGAGCAGCCAGGCTGC

CCCAACGTCTGCTACGACTTCTACTTCCCCATCTCCCACATCCGCCTTTGGGCCCTGCAG

CTCATCCTGGTCACGTGTCCGTCATTGCTGGTGGTCATGCACGTGGCCTATCGGGAGGCC

AAACTGAAGAAATATTTGGAGAAAGAGGGACGGGACTCTCGGCCCCGCTACCCGCCGGGC

AAGAAGCGTGGGGGCCTGTGGTGGACCTATTTGTTCAGTCTCCTCTTCAAAGCTTTTGTG

GACATCATCTTCCTCTTTATCTTCTACCGAGTGTACCCGAGCTACAAACTTCCCCCCTTG

GTGAAATGTTCCCTTCCACCTTGTCCCAACGTGGTAGACTGCTACATCGCTAGGCCGACC

GAGAAGAACATGTTCACCTTGTTCATGATCGTCACGGCCTGCATTTGCATCCTCCTCAGC

CTCGTCGAAGCCTTTTATCTGGTTGGGAAGAGGTGCAAGGAGAGCATGGTGGCCCCACGA

GAGAGCCACGAGGGTCCCAACGGGAATGCGCTTATTCTCAAAAGGGACCCCGGTTGCCTT

TCCCCTAGTAAGCAAAATGAAGTGAGCCCACAGGTTTTCCAGGGGACAAATTACAAACCT

TTCACAACACCCCTCTTAAATATGTCACAGCATGGGGAGATAGAGCCCTCAAAACATGAA

AAACTAGGGTAA

>Ac-gjb6like-XM_008107985 Somewhat deviating, and locates outside and away from the expected position as indicated by the most similar sequences from other reptiles. Thus, sequence is excluded from the main analyses.

ATGGACTGGAAATCTCTCAAAGACGTCTTGGGAGGGGTAAATAGCCACTCCACCAGCATT

GGAAAAATCTGGCTTACTGTCCTCTTCATTTTCCGAATTATGATTCTCGTGGTTGCTGCC

GAAACAGTCTGGGGAGATGAACAGCAAGATTTTACTTGCAACACTCAACAAATAGGATGC

AAAAATGTCTGCTACGATGCATTTTTCCCTGTTTCACACATTCGACTTTGGGCTCTCCAG

CTGATCTTTGTCTCCACTCCTGCACTTTTGGTTGCAATGCATGTTGCCTACAGAAGGCAT

AAGAAGAAACGGAAATACAAACCTGGAGATAAAGTGGATTTTGAAAATCTGAAAAAGCAG

AGGTTTCATATTGAGGGTGCCCTGTGGTGGACATACATCAGCAGCATCTTCTTCCGAATC

ATCTTTGAAACTGTTTTCACATATATTTTTTATTTTATATATAGAAGCTATAAAATCCCT

CCTTTAGTCAAATGTAGTGATTATCCTTGTCCAAATACAGTGGACTGTTTTGTTTCCCGA

CCCACTGAGAAAACTGTATTTACTGTATTCATGCTGGCAGTTTCAGGCATCTGTTTGCTG

TTGAATATACTTGAATTGGCTTATCTGATACTAAAGTCTTGTGTGAGGAAATCTAAGACT

GTGAAAGCACTGCCCAGTGATTAA

>Ac-gjb6-XM_008107986-gjb6like

ATGGACTGGGGAACACTGCAGGCTGTTTTAGGAGGAGTAAACAAACATTCCACTAGCATT

GGGAAGATATGGCTTACGGTGTTATTCATCTTCCGCATTATGATCCTCGTGGTGGCTGCT

GAAGAAGTCTGGGGCGACGAACAAGATGATTTTGTCTGCAACACGCTGCAACCAGGATGC

AAAAATGTCTGCTATGATTATTTCTTCCCCATTTCTCACATCCGACTTTGGGCCCTCCAG

CTCATCTTCGTCTCTACTCCTGCGCTTTTGGTGGCAATGCACGTTGCGTACAGAAGGCAC

GAGAAGAAAAAGAAGTTCCAAAGGGGAGATAAAAGCTGTGAATATAAGGACATAGAAGAA

ATCAAGAGGCAGCGGTTTCATATTGAGGGTCCCTTATGGTGGACATACACTACCAGTATC

TTGTTCAGGCTTATCTTTGAAGCCTCTTTCATGTATGTGTTTTACTATATGTACCAGGGG

TATCATATGCCACGCCTGCTGAAATGCAGCATTTGGCCTTGTCCCAACATTGTGGACTGT

TTTGTCTCTCGACCCACTGAAAAAACCGTCTTCACCATTTTTATGATCGCGGTGTCCGGC

ATTTGCATTCTGCTCAACGTAACCGAGTTCTCTTACTTGCTGCTGAAGTTTTGTGTTCGG

AGGTCGAGAAGAGCACGAACCCCAAAGAATCACACCAACCATGAGACCAAAGAGGAAAGC

AAACAAAACGAAATGAACGAGCTGATTTCAGATAGTTGCCAGAACACAGTAACAGGATTT

CCTAGCAGCTAA

>Ac-gjc1-XM_008113220 (identical to XM_003230274)

ATGAGTTGGAGTTTCTTGACACGTCTGTTAGAAGAAATCCACAACCATTCCACATTTGTT

GGGAAGATCTGGCTGACAGTGTTGATCGTATTCCGCATTGTCCTCACCGCCGTTGGAGGG

GAATCCATCTATTATGATGAGCAGAGCAAGTTCGTGTGCAACACAGAACAGCCGGGTTGT

GAGAATGTTTGCTACGATGCTTTTGCCCCCCTCTCCCATGTGCGCTTTTGGGTTTTTCAA

ATCATCCTGGTGTCCACTCCCTCGGTGATGTACCTGGGCTATGCCATTCACAAAATTGCC

CGGATGGTGGAACACGGGGACACAGAGCGCAAATTCCGAAGCAAACCTTTCCCAATGCGC

TGGAAACAGCACCGGGGCTTGGAAGAGGCGGAGGATGATCATGAGGAAGATCCAATGATG

TACCCAGAGATTGAGGTAGAAAGTGAACGAGAGAACAAGGAGAATCCGCCCCCAACCAAA

ATGAAGCATGATGGCAGGAAGCGGATTCGAGAGGATGGGCTAATGAAGATTTACATACTA

CAGCTCCTGAGCAGAACTATGTTTGAAATTGGTTTTCTTGTTGGCCAGTATTTCCTTTAT

GGGTTTGAGGTCAGTTCCAAATTTATTTGTACCCGGCATCCCTGTCCGCACAAAATAGAC

TGCTTTATTTCCAGGCCCACCGAAAAAACCATCTTCCTGCTCATCATGTACGGTGTGAGT

TGTTTGTGTTTACTTCTGAACATCTGGGAAATGCTTCACTTGGGATTTGGCACCATCCGG

GATACGCTGAACAATAAGAGGAAAGAGCTGGAGGAGTCGGGGACCTATAATTACCCATTT

ACTTGGAATACCCCATCCGCTCCTCCAGGCTACAACATTGCAGTGAAGCCCGACCAGATA

CAGTACACAGAACTGACTAATGCCAAAATAGCCTACAAGCAGAATAAAGCCAACATAGCA

CAAGAACAGCAATATGGGAGCAACGAAGAGAACATTCCCACTGAGTTGGAGAACCTCCAG

CGAGAAATCAAAGTAGCTCAGGAACGTTTAGATCTTGCCATTCAGGCATACAATAATCAG

AACAATCCTATGTGCTCTAAGGAGAAGAAGCCCAAAGGCAGCTCAAACAAAAGCAGTGGC

AGCAGCAAATCAGGAGATGGAAAAACCTCTGTCTGGATTTGA

>Ac-gjc2-XM_008112242

ATGAGCTGGAGCTTCCTAACCCGCCTACTAGAAGAAATTCACAACCACTCC

ACGTTTGTGGGGAAGATCTGGCTGACCGTCCTGATCGTCTTCCGCATCGTCCTGACAGCC

GTCGGAGGCGAGTCCATCTATTCGGACGAGCAGAGCAAGTTCACCTGCAACACCAAGCAG

CCTGGCTGCGACAATGTCTGCTATGATGCCTTCGCGCCGCTATCCCACGTCCGGTTCTGG

GTCTTCCAGATCATCATGATCTCCACGCCGTCGGTCATCTACTTGGGCTACGCCATCCAC

AGGATCGCAAGGTCCACGGAAGAGGAGAAGAAGAGGTTCCGGGGCTTTAAGCGGAAGAAG

CAGTTTGCACTCAACTGGCAAGCTGTCCGCAACCTGGAAGACCCCTTGGGAGCTGAAGAG

GAGGAGCCCATGATCACCGATGAGATCGTGGAGATGGAAGCGAAGGCCAAAGCGAAGGAG

GAGAAGAAGGAGCAGAAGAAGTACGATGGCAGGAGACATATCCAGGAGGAGGGCCTGATG

AAGATCTACGTCTTCCAGCTCCTCACCCGAGCCTCGTTCGAAGTGGCCTTCTTGATCGGG

CAGTACATGCTCTACGGCTTCGAAGTCACGCCGTATTACCTTTGCACCCGAGAGCCTTGT

CCTCATGACGTTGACTGCTTCGTGTCCCGGCCAACTGAGAAGACCATCTTCCTCCTGGTG

ATGTACGTCGTTAGCTGCCTTTGCTTGCTCCTCAACCTGGCCGAGATGTGCCACTTGGGC

ATCGGCACCATCCGGGATGCCATCCGAGAGCGGAAGATCCACAGCTTCCGGCAGCCGCCT

TACAACTACGCCGCCTACCCCAAGAACATCTCCTGCCCGCCGGAGTACAACTTGGTGGTG

AAATCGGACAAGCAGGGCAAGGTGCCCAACAGCTTGATGGCCCATGAGCAGAACTTGGCC

AATGTCGCCCAGGAGCAGCAGTGCACCAGCCCTGACGAGAACATCCCCCCAGACTTGTCC

ACTCTCCATAAACACTTGCGGGTGGCTCAAGAGCAGCTGGACATTGCCTTCCAGAGCTAC

AACGGGACTCAAGGGAATGGCCAGCCCTCGAGGACCAGCAGTCCTGCGTCCGGCGGGACG

ATGGCGGAGCAGAACAGGGCCAACACGGCCCAGGAGAAACAAGGGGCCAAGCCTAAAGCT

GTTTCAGAGAGAGGTAGTTCCAATGGCAAAGATGGGAAAAACTCTGTCTGGATATAA

>Ac-gjc4-XM_003215060-gjc1

ATGAGTTGGAGTTTCCTGACACGCCTCCTTGAGGAGATCAACCAACACTCAACCTTTGTG

GGAAAGGTGTGGCTGTCTGTGCTGATTGTCTTCCGCATTGTGTTGACTGCCGTGGGTGGT

GAGTCCATCTACTATGATGAACAGAGCAAATTCGTCTGCAACACGCAGCAGCCTGGCTGT

GAGAATGTCTGCTATGATGACTTTGCACCCCTCTCACACGTCCGTTTCTGGATCTTCCAG

ATTATCATGATTGCCACCCCATCTGTGATGTATCTGGGCTTTGCGCTACACCGCCTCTCA

CGCCAGCCCCCTCGAAAAACCCGGGCACCTGTAGTGCACCGTGGGGCTGGCCGTGACTAT

GAAGAGGCTGAGGACAATGGTGAGGAGGACCCTATGATCTTTGAGGAGGTGGAGCAAGAA

AGAGAGGTGAAGGAGACACCGGATCAGAAACATGATGGCCGCCGGCGAATCAAAAAAGAT

GGCTTGATGACAGCGTATGTGCTGCAGTTGCTATGCCGTTTGGTGTTTGAGGTGGGGTTT

CTGCTGGGGCAATACATTCTGTACCGCTTTGAAGTCAATCCCTCATATGTATGCTCCCGT

AGTCCTTGCCCTCACACCGTTGATTGCTTTGTCTCCCGTCCCACTGAGAAAACGATTTTC

CTCTTGATCATGTATGCGGTGAGCGGGCTTTGCCTATTTCTCAATATTTGTGAGCTCTTG

CACCTGGGCTTCGGGGGAATCCGGGATGCTTTCCGTGGCCTTGACAAGGACAACGGTGTC

CCTCCCAAGTCCCAGTATGCTCGGAAGGACCCTTCTGCACCTCCTACCTATCATTCAATC

AAGAAGAATCCCTCCAAGAACCACCTTGCTAAGGACAAAATGGCTTATGGTGGAGAGAAC

TTAGCTGGCATTGGTGCTGAGCGCTATGCTGTTGCATCGGGTCTCCCAAACCATGAGTTG

GAACGACTTCGCAAGCACCTCAGGATGGCACAGGAGCATTTGGAAATGGCTTTCCATCTT

CAGCCAGAGGACACAGCCAGCCCTTCGCGTAGTAGCAGTCCAGAGTCTAATGGGTTGGCA

GCAGAACAGAACCGCCTCAACCTGGCACATGAGAAGGAAGGTGCAGCCTGTGAGCGAACA

ACTGGTATGTTGGGCCAATGTCTGGAACAAGATTGGGGAATCCTTGGCCCTCTAGAATTG

GCATCCAGTTATTTATAA

>Ac-gjd1-XM_003229683-gjd2

ATGGGAGAGTGGACCATTTTGGAAAGGCTGCTGGAGGCTGCGGTGCAACAGCATTCGACG

ATGATAGGGAGGATCCTGCTGACGGTGGTGGTGATCTTCCGCATCCTGATCGTGGCCATC

GTGGGTGAGACGGTTTATGAGGATGAGCAGACCATGTTCATGTGTAACACGCTGCAGCCC

GGCTGCAACCAGGCCTGCTACGACAAGGCCTTCCCCATCTCGCACATCCGCTACTGGGTC

TTCCAGATCATCCTGGTGTGCACCCCCAGCCTCTGCTTCATCACCTACTCGGTACACCAG

TCGGCCAAGCAGCGGGACCGGCGCTACTCCTTCCTCTACCCGCTGCTGGAAAAGGACTGT

GCCCGCGACGGTGGCAAGAAGGTCAAGAACGTCAACGGCATCTTGGTGCAGAATCCGGAT

GGCTTGTCCAAGGAGGAGCCCGACTGCCTGGAGGTGAAGGAAATCCCCAGCACCCCCCTG

CGGCCGCCCAAGAGCTCCAAGGTCAAGCGCCAAGAGGGCATCTCCCGCTTCTACATCATC

CAGGTGGTTTTCCGCAATGCCTTGGAGATTGGCTTCCTCGCCGGACAGTACTTCCTGTAC

GGGTTCAACGTGCCGGCCATCTTCGAATGCGACCGCTACCCATGCGTAAAGGAGGTGGAG

TGCTACGTCTCCCGCCCCACCGAGAAGACCGTCTTCTTGGTCTTCATGTTTGCCGTGAGC

GGGATCTGTGTGCTGCTCAACCTGGCCGAGCTCAACCACCTCGGCTGGCGCAAGATCAAG

ACGGCCATCCGGGGGGTGCAGGCCAGGCGGAAATCCATCTGCGAGGTGCGCAAGAAGGAC

CTCTCCTCGTTGGCCCAAGTGCCTACGCTTGGACGGACGCAGTCCAGTGAGTCTGCCTAT

GTGTGA

>Ac-gjd2-XM_0032144476

ATGGGGGAATGGACCATCCTAGAGAGACTCCTCGAAGCCGCCGTCCAGCAGCACTCCACT

ATGATAGGGAGGATTCTGCTGACCGTGGTGGTGATCTTCAGGATTCTTATTGTGGCCATT

GTAGGAGAAACGGTTTACGACGACGAGCAGACCATGTTTGTGTGCAACACTTTGCAGCCA

GGGTGCAACCAGGCGTGCTACGACCAAGCATTTCCCATTTCTCACATTAGGTACTGGGTG

TTTCAGATCATCATGGTGTGTACTCCCAGCCTCTGCTTCATCACATACTCGGTCCATCAG

TCTGCCAAGCAGCGGGAGCGGAGGTATTCCACCGTCTTCCTCACTCTGGACAGAGACCAG

GACTCTATGAAACGGGAAGACAGTAAAAAGATCAAAAATACCATTGTCAACGGGGTGCTG

CAAAATACCGAGAACTCCACCAAGGAGGCAGAACCAGACTGCCTGGAAGTGAAAGAGATC

CCCAACCCAGCCATCCGAACGACAAAGTCAAAGATGAGGCGGCAAGAAGGCATCTCCAGA

TTTTATATCATCCAGGTGGTCTTCAGAAATGCCCTGGAGATTGGGTTTTTGGTGGGACAA

TATTTTCTGTATGGATTCAATGTCCCATCCATGTATGAATGTGATAGGTATCCTTGCATT

AAGGAAGTGGAATGCTATGTCTCAAGACCCACGGAGAAAACTGTCTTCTTGGTCTTCATG

TTTGCCGTCAGTGGCATTTGTGTGGTGCTCAATCTGGCAGAACTGAACCATTTGGGCTGG

AGGAAGATCAAAATGGCAGTGAGGGGAGTGCAAGCCAAACGGAAATCAATATATGAAATC

AGGAACAAGGACCTTCCACGAATGAGTGTGCCTAACTTTGGCAGGACTCAGTCGAGTGAT

TCCGCTTATGTGTGA

>Ac-gjd3-XM_008113372

ATGGGTGAGTGGGGGTTCCTGAGCTCCCTTCTGGATGCGGTGCAGGAGCACTCGCCCATG

GTGGGCCGCTTCTGGCTGGTGGTGATGCTTATCTTCCGCATCCTGATCTTAGCCACCGTA

GGCAGCGACGTCTTCGACGATGAGCAGGAGGAATTTGAGTGCAACACCAAGCAGGTTGGG

TGCAAGCAGATTTGCTATGACTTGGCCTTCCCCATCTCTCACTACCGCTTCTGGGTTTTT

CACATTGTGGTGCTCTCTGCTCCAGCTGTCCTCTTTGTCATCTACTCCATGCACCAGACC

ACTAAGATAAACCGCAACCTGAAGGAGATGGAAACAAGTGAGGAGGAAGCCAATCGGGCA

GTGCAGAGGGCTCAAGCACAATATTGCCTCCAGACCAACCAACGCAGCCAAAACATCAAG

ACATTCTACATAGTCAATGTGGTTGTGAGGATCTTGGCTGAGATCGGCTTTCTTGTTGGG

CAGTGGATGCTGTATGGCTTCCAAGTGGAATATCAGTACATCTGCAAGCATCGGATGTGT

CCACACCTCATTGACTGCTTTGTCTCTCGCCCAACTGAGAAGACCATCTTCATCCAGTTC

TATTTCATGGTGGGACTGGTGTCAGCTCTCCTCAGCTTGGTGGAGCTGGCTCACCTTTTG

TTCAAGAACCGGTGCCGGAGATGCCGCACCATTGCTCCTCCTCCTACTGCGGCATCGTAC

GAACAGCAGGACAATTGGTCCAACCAGAAACAAGAGAAATCTCAGCACTTCCTGGCCCCT

GTGGATGGGGGACACAATGGGACACCTTCTCATGACCAGATACCCAACCATTGTGAGCCC

AAGGCTCCCTTCCATCATAAAAGCTCCGCCAAGTGCAGCAGGTCGTCTCGCTCCTCCACC

CGAGCAGACTTGACAGTGTAA

>Ac-gjd4-XM_008112325 Splice site

ATGGAATGTTGGGACTCACTTGGATTTTTAATCCTTGCTCTCAGCTACAACGTCACAATT

ATAGGGAAGATTTGGCTAACTCTTGTGATCTTGCTAAGATTTATGGTGATCTTCTTAGCA

GCATATCCTCTCTACCAAGACGAGCAGGAACGCTTTGTCTGCAATACGTTACAACCAGGA

TGCTCCAACGTCTGCTATGACATTTTTGCCCCAGTGTCCCACTTTCGCTTCTGGCTCATT

CAGACTGTCTCCGTCCTTCTACCCTATGCTGTGTTTGGTGTCTGTGTGTTACACAGTGTG

GTCAGGCAGGTTGTGAAGTCATCTTCTCTGCCTTATCATCGATACAAAGAGATAAAAACT

TCGGTAGGCTCCAAAGTTACAAAGAAATCATCCAAAGGTGCAGGAAGGAGCAGAATTGCT

TGCAAAGCACATGAAATGGATATTCCTGATTTTTCGGGAGCATATACAGTTCATCTTCTT

TTGCGAATATTGATAGAAGTTGGTTTTGGAGCTGGACATTATTACCTTTTTGGATTCTTT

GTTCCAAGGCGGTTCTCCTGTAACCAGCCCCCTTGTACAAGTTTTGTTGACTGCTACATC

TCCAGACCCACTGAAAAATCTATCATGATGCTTTTCATCTGGGGACTCAGTGGCTTTTCA

TTTCTCCTCAGCCTCGTTGACTTGGTCTTTGCCTTCCGGACCAACGCTGTAAGAAACCAA

AGAAATAAGTTGCTGTTGGAGAAGTTTACAGCACAAGAGAAGTGCAAATTCAGCCTGAGC

CATGGTGACCAGCCAAACCAAGATTTGCTTGGCCATGGGAGCATTGCTGTGCCAGACAGT

TGTGGCAACTGTTTGGTTGGGCGTGAGATTGCAGGAGGCTACTCTTTCCAGCCTACATTG

ACTTCTCAGCAAACAATTAACTCAAACCTCAACAGTAATAGTAACAAGTCTTGTGTTACA

TCTGCTAATCCAGAGGGCAAAGTTGTTCCTTTCTGTGGAAGTGGCCAATCAGAGATTGCA

TGTGAGCAACCAAGATGTGAACAGAAGACATGTTCTCTCAAAGGGGTCTCAACTTTTAAA

CCTCAAGATGGCTGCCATCATAGATCTCATTCTTCTGCCAGTCATCACAAGCCCTCAGTT

CATTATTCTGTCCTGGAGAGGAAAGCTTCTGATGTCCAGTCTGTCTGCAGTAGCACTGGG

TGTTCGCGATCCAAGAAATCTGAATGGGTGTGA

>Ac-gje1-XM_008122239

atggggagccccaacaacgccacgccaggactgcggctgcttaggcctccaaccgtgatt

ggacaattccacacacttttctttggctcagtgagaatgttcttccttggtgttttgggt

tttgctgtttatggaaatgaagccttacacttcagctgtgagccagacaagagggaagtt

aatctattctgttacaaccagttcaggcctataaccccacaggtattctgggctttacaa

ctagtgaccgtactggtacctggagccattttccatctttatgctgcatgtaaaagcatc

gctcaggaagatatccttcaaagacctgcctacaccgttttttatattctctctgttctg

ttaaggatcatccttgaagttgtggctttttggctccagagtcagctctttggttttcaa

gtgaacccactctacagatgtgatgcaggagcccttgataaaaagtttaatattaccaga

tgcatggtaccagaacattttgaaaagacaatctttcttattgctatgtacacttttact

gtgatcacaattatgttatgtattgcagaagtttttgaaatcttgtatagaaggctaggt

ttcttgaatacgcaataa

# Supplementary Figure S21. Some sand lizard (*Lacerta agilis*) connexins.

These sequences were obtained from GenBank.

>La-gja8-XM_033146811

atgggtgactggagtttcttggggaacattttagaagaggtgaatgaacaatccacggtc

attgggagagtctggctgactgtcctcttcatttttcggatcctaatccttgggactgct

gcagaatttgtatggggagatgaacagtcagattttgtgtgcaacacccagcagccaggt

tgtgaaaatgtgtgctacgacgaggccttccccatctcccacatccgactgtgggtcctg

cagatcatctttgtctccactccctcgctggtttatttgggccatactgcacatcatgta

aggatggaagagaaaaggaaagagcgggaagaagcagagagggctcagcaagctggggcg

gacgaagaaaattcaccccttgatccagaccggaagagccctataactacgagagaagca

agctccaagggaaccaagaggttccacctcgagggaacgctcctgagaacctatatttgc

cacatcttcttcaaaaccctttttgaggtggggttcatagtgggccagtacttcttatat

ggcttccgaattcttccgctttaccgctgtggcagatggccttgccccaacctcgtggac

tgttttgtgtccaggcctaccgaaaagactgtttttattgtgtttatgcttgctgtggca

tcagtctctctcttcctcaatcttgtggagatcagccacttaggctttaagaagatcctg

ggtgccttcaggaggccagtggagcaacaacctcaggagaccccagagaagccccttcat

tctatcgcggtttcatctatccagaaggtcaaaggctacaagttgctggaggaagaggag

aagccgacgttccactatttccctttgaccgaagtgggagtagaaaccaccccagttccc

ccctcattcaacgactttgaggagaagatcagcatggaacgcctgaaagacctttccagg

gccttgggggagcacctgccttcttacgtctatgcggatgaacaggaggaggagaaagtc

gaagcaggagaggagccggaacaggaagtggctgaggcggcggcccaggaacagagggtg

gaactggtgactgaaaaagccccagagaaaggagaagatgacaagcatatggaggaggaa

caagagccagagccgctgggagacgacttgcctttaccatccgctgatcttacagctgac

acaagacccctcagcaggctaagcaaagccagtagtcgtgccaggtcagatgatctgact

gtatga

>La-gja9-XM_033158682-GJA9

atgggagactggaatttcctgggcgggattttggaggaagtccacatccattctaccacg

attggcaagatctggctgaccatccttttcatcttccggatgctggttcttggcgtggcg

gccgaagacgtgtgggacgacgagcaagcagacttcatctgcaacacggagcagcccggc

tgcaggaatgtctgctacgaccaggcctttcccatctccctcatcaggtactgggtcctg

caggtgatctttgtctcttcgccctccttggtctacatgggccatgcactgtacaggctg

aggaccctggagaaggagcggcagaagaagaaggcccaggtgagggctgagctggagggg

acatccccagagaaggcggaggatcgacggaaactggagaaggagctgcggcagctggag

cagaagcgcctgagcaaagcgcccttgaggggctcgctgatgtgcacctacctggcccac

atcctgatgcgctctgccctggaggtggccttcatggtgggccagtacctgctctatggc

ttccagctggagccgctctaccagtgccgccggcccccgtgccccaatgtggtcgactgc

ttcgtctcccggcccaccgagaagaccgtcttcctgctcttcatgcaagccatcgccgtc

gtgtcgctcgccctcaacggcctggaagtcgcccacctggcctgcaagcgctgccgggcg

ggctcctgcggtgcttcgccctcggccctcgggggcagcccgcgctccgccctgctgggc

cagcagccaaaccccccgccctggggaccccgccctgacgacctcctcgcgcagggccca

ccactcgccgccgatgaagcccacccgccgcccggccctcgcagcccgacgcggagcagc

agcagcaccaaggagagcggccccgttgccgacgcagcccaggcgatggccggctcctct

tcctcgtcccgctcgccgccgcccgcgaacggtgcccggcgcggctcctgcagcggcggg

gactcgggcagcgccgtctccccggccacggcggctccgagcacggtcagctccccgccc

tccagcaccggcagcgagcggggacagcaggaagggagtttccagcccggccacggccgg

actcgagcctccgccgccgccgaggggagccagtcgggaggatcggcttcctgcaagggc

ggcggcggcagggcgaggaggagcggcaggagcggcggcccaggactccccccgccccgg

aaggaggcctcggccggcgggggcagccacaggcgggcgcccacggacctccgcgtctga

>La-gjb2-XM_033146294-GJB2

atgtggggaacgctccaatccattttaggaggtgtaaacaaacactcaaccagcattggg

aagatatggctcactgtcctcttcattttccgtgtcatgatccttgtggtggctgcagaa

aaagtctggggagacgaacagtcagatttcacttgtaacactctgcaacctgggtgcaaa

aatacctgctatgatgattatttcccagtttctcatattagactttgggctctccaactt

atctttgtgtctacccctgcccttctggtggcaatgcatgttgcctacagaaggcacgag

aagaaacggaaataccgacatggggagaaaattgaccttgaaagtctgaaaacccaaaag

tttcatatcgaaggacccctatggtggacatacaccagcagcctcttcttccgaattatc

tttgaagcgactttcatgtacatattttattacatgtacgatgggtaccgtatgcctcgt

gtagtgaaatgcactgattttccttgcccaaatacagtagattgctttatttctcgtccc

actgagaaaactgtatttactatttttatgctggctgtgtcaggcatttgcatgctccta

aatgtacttgagttgtcttatttgatactgaagtcttgcatgaagaagtctcagaccaag

aaaccactgcccaatcattag

>La-gjb4-XM_033158689-gjb5like

atgaactgggcttcattccaagaacttgtgagtggtgtgaacaagtattccacaggtctg

gggcgtgtgtggctcacggtggtcttcatcttccgtttccttgtctacacagtggcagct

gagcttgtctggtccgatgaccagcaggacttcatgtgcaacacacgtcagcctggctgc

accaatgtctgctataaccagtcctttcccatctctcacatacgtttctgggccttccag

cttatcctggtgacatgcccttctcttttcgtcctcatgcatgtggcctatcgggaaacc

aaggagcagaagtgcctggaaagggcaagagaacgcttccatccacagtaccccaaccct

gggaagaagtgtggaggcctgtggtggacctacctactcagcctcctcgccaaggcaact

gtagatctcaccttcctctacatcttccactgcctatatcccaactttgatcttccaaag

gtggtgaagtgtgctgtggttccctgtcccaacgtggttgattgcttcattgctcggcct

acagagaagaagctcttcacttatttcatggtagccactgccatgctatgtgtcattctc

aacctctgtgagatggcctaccttgtaagcaagagttgcaagaagtctgcagcatcangc

aaacctgagacagaagctcacatttcatttcaataa

>La-gjb5-XM_033157671-gjb5like

atgaactggggagtctttcaagggctcctgagtggggcaaacaaatattccacagccttt

ggacgtatttggctctcagtggtcttcatcttccggcttctagtctacatagtggcagct

gagaaggtttggggagatgaccagaaggactttgactgcaatactgagcaaccaggctgc

ccaaacgtctgctatgaccattacttcccaatctcccacatccgtctctgggccctacag

ctcattctggttacctgtccttccctgctggtaatcatgcatgttgcctaccgagaagcg

aaactggagaaatataggaagaaggttggagcagactatgcactgcgctatcctcctggc

aagaagcgtggaggcttgtggtggacctatttcttcagcctcatcttcaagtctgctgtg

gacatcatattcctattcatcttctaccatgtgtacccaaactacacactacccagtgtg

gtgaaatgctccctcgaaccttgtcccaacgttgtagactgcttcatagccaggcccaca

gagaagaacatcttcacccttttcatgatcatcactacctgtgtttgcatcctcctcggc

cttgttgaagccttctacctcgttgggaaacgatgtacagagtgccttcagcccagaaat

gaaagccacaggaggcagggcagtgaatggatgaggagagagaacgcttgctctctgccc

aacagcaaacatgacccaaatgaagtgggtgtgcaggtgttccatgctgcagattacaaa

ccatctacctccatcatcaaggctgcaccacctacccagagaactctaagcacataa

>La-gjb6-XM_033146295-gjb6like

atggactggggaacgctacagggcattttaggaggtgtaaaccaacattctaccagtatt

ggaaagatttggttgactgtcctgttcatcttccgtattatgatccttgttgttgcagcg

gaggaagtctggggtgatgagcaagatgattttgtgtgcaatacactgcaaccaggatgc

aaaaatgtctgctatgatcattttttccccatttcccacatcagactctgggccctccag

ctcatctttgtctccacccctgcactcttggtggcaatgcatgtcgcatacaggcggcac

gaaaggaaaaagaagttccgaaagggagataaaagctgtgaatataaggacatagaagaa

ataaagaaacagaaatttcacatcgaaggtcccttgtggtggacatacactaccagcatc

ttgttcagactgattttcgaagcttctttcatgtatgtgttttactatatgtatcaggga

tatcacatgcctcggctattgaaatgcagcgcctggccgtgtcccaatgtagtggactgc

tttgtttcacgtcccactgaaaagaccgtctttaccattttcatgatttcagtgtctggc

atttgcattctgctgaatgtggccgagctctcctatttgctgctgaaggtttgtgccaga

aggtccagaagaccagggaaaccaaaaaatcacactaaccatgagaacaaagaagaaagc

aaacaaaacgaaatgaatgagcttatatctgacagctgccagaacacagtaacaggattt

ccaagcagctaa

>La-gjc1-XM_033170019-GJC1

atgagttggagtttcctgacacgtctactagaagagatccacaaccactccacgttcgtt

gggaagatctggttgacgctgctgatcgtcttccgcatcgtcctcaccgccgtgggaggg

gagtccatctactacgatgagcagagcaagttcgtgtgcaacacggaacagccgggctgc

gagaacgtttgctacgacgctttcgccccgctctcccacgtgcgcttctgggttttccag

atcatcctcgtctccactccctcggtgatgtacctggcctacgccgtccacaaaatcgcc

cggatggtggagcacggcgagacggagaggaagttccggagcaagcctttccccatgcgc

tggaaacaacaccgaggcttggaagaagccgaggacgaccacgaggaggacccgatgatg

tacccggagatcgagctggagagcgagaacaaggaaaacccacctcctaccaaagtgaag

cacgacggcaggaggcgcatccgagaggacggcctcatgaagatttatatactgcaactc

ctgagcaggactgtttttgaaatcggcttcctcgtcggccagtatttcctctacgggttc

gaagtgagcccgaggttcctctgcagccggaagccgtgcccgcacacgatagactgcttc

atttcgaggcctacggaaaaaaccatcttcctgctcatcatgtacggcgtgagctgcctg

tgtttacttctgaacgtatgggaaatgctccacttgggattcggcaccatccgggacacg

ctgaacaataagcagaaagagctggaggactcggggacttacaactacccgttcacttgg

aacacgccgtccgcacctccgggctacaacatcgcggtgaagcccgaacagatccagtac

acggaactgtccaatgccaaaatggcctacaagcagaacaaggcgaacatcgcccaagag

cagcaatacgggagcaacgaggagaacatccccaccgagttggagaccctccagcgggaa

atcaaagtcgcccaggaacgcttagacctcgccatccaggcctacaacaaccagaacaac

ccgacctgctccaaggagaagaagccaaagggcagctcaaataaaagcagcagcagcagc

aagtcaggggatgggaaaacctcagtctggatttga

>La-gjc4-XM_033159804-gjc1like

atgagctggagtttcctcacccgcctccttgaggagatcaaccagcactcgacctttgtg

gggaaggtgtggctgactgtgctaattgtcttccgcattgtcctgacggcggtgggcggc

gagtccatctactacgatgagcagagcaagtttgtgtgcaacacgcagcagccgggctgc

gaaaacgtctgctacgatgcctttgccccgctttcgcacgtccgcttctggatcttccag

atcatcatgatagccacgccctcagtgatgtacctgggctttgcgttgcaccgcctctcg

cgccagccgcctcgaaaaagccgcgcccccgtggtgcaccgcggcgccgtccgcgactac

gaggaggccgaggacaatggcgaggaggaccccatgatctttgaggaggtggagcccgag

agggaggtgaaggagcccgagagccagaagcacgacggccgccggcgcatcaagaaggac

gggctgatgacggcgtatgtgctacagctgctgtgccgctcggcgtttgaggtgggcttt

ctgctggggcagtacgtgctgtaccgcttcgaggtcatgccctcgtacgtctgcacccgc

agcccctgccctcacacagtggattgctttgtctcccgccccacggagaaaaccatcttc

ctcctcatcatgtacgccgtgagcggcctttgtctcctgctcagcatctgtgagctgttt

cacctgggcttcggcgggattcgggatgccctgcgcggccttgatagtaaagatggtggt

gacccccccaggtcccagtatgtccagaagcatcccagtgcacccccgacctaccattct

atcaagaaggacacctccaagagacagctagccaaggacaagctgggctacggtggagag

agcttggctgggctggctgccgaccgctatgctgtcgcaccgaccgtcccgagccatgag

ttggagcgactgcgcaaacacctccggatggcacaggagcacctagaaatggccttccac

ctgcagccggaggacacggccagcccctctcgtagtagcagccccgagtccaatgggctg

gctgcagaacagaaccgcctcaactcagcacatgagaaggaaggcgcagcctgtgagcga

ccgactggcctctga

>La-gjd1-XM_033158413-gjd2like

atgggagagtggaccattttggaaaggctgctggaggctgcggtgcaacagcattccacc

atgataggcaggatcctgctgacagtggtggtgatcttccgcatcctgattgtggccatc

gtgggagagacggtttacgaggacgagcagacgatgttcatgtgcaacaccctgcagccc

gggtgcaaccaggcctgctacgacaaggccttccccatctcgcacatccgctactgggtg

ttccagatcatcctggtctgcacgcccagcctctgcttcatcacttactcggtgcaccag

tcggccaagcagagggaccgacggtactccttcctctacccgctgctggagaaggacggc

aggaaggtgaagaacgttaacggcatcctggtgcagaacccggacggctcctccaaggag

gaacccgactgcctggaggtgaaggagatccccagcgcccccatgcggccccccaagagc

tccaaggtcaagcgacaggagggcatctcccgcttctacatcatccaggtggttttccgc

aatgccttggagattggcttcctggcaggacagtacttcttgtacggcttcaacgtgccg

gccatttttgagtgcgaccgctacccgtgcgtcaaggaggtggagtgctacgtgtcgcgg

cccacggagaagaccgtcttcctcgtcttcatgttcgccgtgagcgggatctgcgtcctc

ctcaacctggccgagctcaaccacctcggctggcgcaagatcaagaccgccatccgaggg

gtgcaggccaggcggaaatccatctgcgaggtgcgaaagaaggacctctcctcgctggcc

caagtgcctacgctcggacggacgcagtcgagcgagtccgcctacgtctga

>La-gjd2-XM_033144424-GJD2

atgggggaatggaccatcctagagagactcctcgaagccgccgtccagcagcactccact

atgatagggaggattctgctgaccgtggtggtgatcttcaggattcttattgtggccatt

gtaggagaaactgtgtacgacgatgagcagactatgtttgtgtgcaacaccttgcagcca

ggctgcaaccaggcatgttatgaccaagcatttcccatttctcacattagatactgggta

tttcagatcatcatggtgtgcactcccagcctctgctttataacatattctgttcaccag

tctgccaagcagagggaaaggaggtactccactgtcttcctcactttggacagggatcag

gactccatgaagcgggacgacagcaagaagatcaagaataccattgtcaatggggtgctg

cagaacactgagaactccacgaaggaggcagaacctgactgcttggaagtaaaggaaatt

cccaacccagctatcagaaccaccaagtcaaagatgaggcggcaagaaggcatctctagg

ttttatatcattcaggtggtcttcagaaatgccctggagattggatttttagtgggacag

tatttcctgtatggattcaatgtcccatccatgtacgaatgtgacaggtatccttgcatt

aaggaagtggagtgttacgtctccaggcccactgagaagacagtcttcttggtcttcatg

tttgccgtgagcggcatttgtgtggtgctcaatttggctgaactgaaccacttgggctgg

agaaagatcaaaatggcagtgaggggagtgcaagcgaaacggaaatccatttatgaaatc

agaaacaaagacctgccacgtatgagtgtgcctaactttggcaggactcagtccagtgac

tctgcttatgtgtga

# Supplementary Figure S22. Japanese gecko (*Gekko japonicus*) *gja2*, *gja9* and *gjb7*.

The sequences were collected from GenBank.

>Gj-gja2-XM_015409011-gja3like

atgggggactggcacctgcttggaaggctgctggagagtgtgcaggagcactccacagtc

gtgggcaaagtctggctgacggtgctcttcatcttccgcattctggtcctgggtgctgct

gcagagaaagtgtggggggacgagcagtccaacttctcctgcgacaccaagcagcccggc

tgccagagcgtctgctacgaccagaccttccccatctcccacatccggttctgggtgctg

cagatcatctttgtctccacacccagcctcatctacctgggccacatcctgcacctggtg

cgcatggaacagaaggaacaagaaagcctggaagctcagggtagcagctcccttacggga

caccctaaccctaaccccaccaaggtctccattcgggatactcaaggtaggatccgcctc

cagggggccattctgaggacttacatttgcaacatcatcttcaagaccctctttgaggtg

ggcttcatcttggggcagtatgtcctgtatggttttgagctgaagcctctctacacctgc

agccgctggccctgccccaacacagtcaattgctacatctcccgccccactgagaaaacg

atcttcatcctcttcatgctagccgtggcctgcctctccctcctgctcaacatagtagag

gtgtaccacgtggccttcaccaaatgcagggtgagacagtcagccagacacaatccaggt

gctgaggaggaggctaggagtcccgatccccaaggacatgggagcactactccaccccgt

agtatcactttgcagtctagggacaaggtctctaagggggagagccctggagcagggatg

gtgaatgacagccccgcttgggttgcgagccagaagcgagacagcaagtgcagcaagact

ggcagcaaatggaagcctggcgacctagctgtttag

>Gj-gja9-XM_015409157-GJA9

atgggagactggagtttcctgggaggaattttagaagaagtccatattcactccactacc

attggcaaggtttggctaaccatcctcttcatattccggatgcttgttcttggtgtggct

gctgaagacgtctggaatgatgagcagactgaattcatctgcaacacgcagcaacctgga

tgcagaaatgtgtgttacgaccatgccttccccatttctctcataagatactgggttttg

caagtcatctttgtatcctcaccttccttggtgtacatgggccatgccttatacagacta

agaacattagaaaaagaacggcaaaagaaaaaagcaaagatgagggctgaactggaggga

gctgaatttcaaataattgaggaccgtaaaaaactagagagggaactccgacaactggag

caaagaaagctcaacaaggcaccccttcggggctcactgctctatacttatgtgatccac

attttgattagatctatccttgaaactggatttatgattggccaatacctcttgtatgga

tttcaactagaccctctgtacaagtgtcaaacagctccttgcccaaacactgtcgaatgt

tttgtctccagaccgacagaaaagaccgtctttcttgtttttatgcaatccatagcaacc

gtttccttgtgtttgaatatcctggagctcgggcacctggttttcaagaaggcccgaaag

ggattctgcgggcagtacagaaggagagacgaggcggatgaggcttacaccgctgctctg

agaaacggccttgagatgcaactacgtgtcggggcgccaccgaggcccctgccttccgct

ctgagcggattcaactggctcctgggaaaccaaacggaggcggcagcgctttgccattca

gaggaccgccaagagcaccgagggcctgcatcgccagcgccccctcagaacagcagcagc

tccaacaacaacgaggcggcgcttcagggacccaaggcgaccgccttcgaaacgcccctt

cccgcactgagggggagcccagcggccctcccagtagagcaggacaggccgtcccacacc

ccaacggctgctagcggcaagcgcgccgctccagacctctgcatctga

>Gj-gjb7-XM_015424746-GJB7

atgagctggggattcctacgagatgtcctaagtggagtgaatcagtattcaacaggaatc

ggcagaatctggctggctattgtgtttatattccgcctgcttgtgtatgtagtggctgca

gaaaacgtgtggaaagatgaactgaaggagtttgagtgcaatattaggcaacccggctgt

gaaaatgcgtgctttgatcacttcttccctgtttcccaggtgaggctttgggccttgcaa

ctgatcatggtctccaccccatcgcttctggttgtcctccatgttgcctatcgagaaaac

agagaaaaaagacacaagaaaaagttgtacaagaatacaggaagcatggatggtggattg

ctctgcacctaccttatcagcctcctttccaaaacagcatttgaaatcagctttctcttt

ttatttcacaagttgtacggtggcttcaatgttccccgacttgttaagtgtgacatgcat

ccatgtccaaacactgttgattgctacatatctaagccaacggagaaaaaagtcttcctg

tatatagtggtggtgacgtcctgcttgtgcattgttctaaatgtcactgaactcacctac

ttgatcttcaaatcctccatcaaatgctgtttcaaaaattacatcaagagagttcaggac

tccaaacatgaatgcaaaaaaacaaattctaatgatgagcacggaacaaccttggacagt

ggaaattattcagctactctcttcagcatccagagagatgaaagcaatctgccttccaat

ccacatgaaaaataa

# Supplementary Figure S23. Central bearded dragon (*Pogona vitticeps*) *gja4* and *gjb2*.

The sequences were collected from GenBank.

>Pv-gja4-XM_020800133-GJA4

Atgggtgactggggattcctggagaagttgctggaccaggtgcaggagcattcgaccatc

Atcggcaagatctggctgacggtcctcttcatcttccgcatcttgatcctgggcctcgct

Ggggagtccgtctggggagatgagcagtcggactttgtctgcaacaccaagcagcccggc

Tgcaccaacgtctgctacgacaacgctttccccatctcccacgtccgctactgggtgctg

Cagttcctctttgtcagcacgcccaccttggtctacctgggccacgtcatctacctccac

Cggagagaggagaagctgaaggagaaggagagcgagctccgagccctgcagtccaaggat

Tataaagtggcagccaccctcatggttctggagaagaagatggccaagatcggggtgacc

Gaagatgggaagataaaaatccgtggccctctgatgtgcacctacatcgctagcgtggtc

Ttcaagtccatctttgaagctggcttcctccttggccagtggtacctgtatggttttgtt

Atgaagcccctctatgtgtgtcaggggtctccttgcccacacctggtggactgcttcatg

Tctcgccccacagagaaaaccattttcatcttatttatgttggtggtgggcttgatttcc

Ctcaccctcaaccttctggagctcttctacctctgtggtaagagcctgctccgcaacgtc

Aaggagtcagactgctgctccccatcttccgccccaccactgccctgcgacgccaacgct

Ttcccttccaaactctgtgaaatgtccagcgcgccttatccggaaaagtcctacttctgc

Ctcccgatggctgaaaggcattcgccctcctaccaggcttacaacaagctgtccagcgag

Cagaactgggccaacttcaacacagaagaaaccatggctttgcaaggccagaataaaccc

Ccggatgtctttgccccagacggctccaaggtctgggtcgctcaggaaaggctcccaagc

cggccgagtagcagtgcttccaagaaacaatacgtctag

>Pv-gjb2-XM_020802033-gjb2like

atggactggaaatcactccatgccatcttaggaggtgttaataaacactctaccagcatt

ggaaagatctggcttactttcctcttcattttccgaatcatgattcttgtggttgctgcg

gaaaaggtgtggggagatgagcagcaagattttacttgtaacacactacagccaggatgc

aaaaatgtctgctatgatgaacatttcccagtttctcatattagactttgggctctccaa

attatctttgtctccactcctgcacttctggtggcaatgcatgtcgcttacagaaagcac

gaaaagaaaagggaatgcaaacctggtgataaagatgacatcgaaaatctgaaaaagcag

aaatttcacattgagggtgcactatggtggacgtacaccagcagtatcttcttccgaata

atttttgaagctaccttcatgtatatattttattttatgtacagaggatacaaaatgcct

cgtttggtgaaatgcagtgattttccttgtccaaatactgtagactgttttatttcccga

cccactgagaaaactgtatttaccattttcatgctggccgtatcaggcatctgtatgctc

ttaaatttacttgagttattgtatttgatgctaaagtcttgcatgaaaaaacctcagact

gtgaaatcactgcctagtcattaa

# Supplementary Figure S24. Tropical clawed frog (*Xenopus tropicalis*) connexins.

The chronology of the sequences is according to the Greek nomenclature. Size nomenclature is indicated in parentheses. GenBank accession number is given for each entry.

>Xt-gja1-NM_203525

ATGGGTGACTGGAGTGCCTTAGGAAGACTTCTTGACAAAGTTCAAGCCTATTCGACTGCA

GGAGGGAAGGTGTGGCTGTCTGTCCTATTTATATTTCGAATCCTTTTATTAGGGACAGCA

GTGGAGTCAGCCTGGGGAGATGAACAATCAGCATTTGTCTGCAATACTCAACAACCAGGT

TGTGAAAATGTTTGCTATGATAAGTCATTCCCGATCTCTCATGTGCGTTTCTGGGTACTT

CAAATCATATTTGTCTCAACGCCTACCCTCCTTTATTTAGCCCATGTGTTTTACTTGATG

AGAAAAGAAGAGAAGCTAAACAGGAAAGAAGAAGAACTTAAAATGGTTCAGAATGAAGGT

GGTAATGTAGACATGCACCTTAAACAAATTGAAATAAAGAAATTCAAATATGGCCTTGAA

GAGCATGGAAAAGTTAAGATGCGAGGTGGACTTCTTCGCACTTACATCATCAGCATTTTG

TTTAAATCAGTCTTTGAAGTTGGCTTTATTTTCATTCAATGGTACATGTATGGGTTTAGC

TTAAGTGCCATCTACACATGCAAAAGAGATCCTTGCCCACATCAAGTGGACTGTTTCCTT

TCTCGACCCACAGAGAAGACCATTTTTATCTGGTTCATGTTAATAGTTTCTATAGTTTCT

CTTGCCCTAAATATCATCGAGCTGTTCTATGTCACCTACAAAAGTATCAAAGATGGCATC

AAGGGGAAAAAAGACCCATATTCTGCCACAAATGATTCTGTGGTTCCAGGAAAGGAATGT

GGCTCCCCTAAATATGCTTATTTTAATGGCTGTTCTTCTCCAACTGCTCCCATGTCTCCA

CCTGGTTACAAACTTGTTACTGGAGAAAGAAATCCTTCTTCTTGTCGCAATTATAACAAG

CAAGCAAGTGAACAAAACTGGGCCAATTACAGTGCAGAACAAAACAGGATGGGGCAGGCT

GGAAGCACAATTTCTAACACTCATGCACAGCCTTTTGATTTTTCGGATGAACATCAGAAT

ACTAAGAAAATGGCACCAGGACATGAGATGCAGCCCCTGACTATATTGGACCAAAGACCA

TCTAGTAGAGCAAGCAGTCATGCAAGTAGCAGACCTCGACCTGATGATTTAGAGATCTAA

>Xt-gja2-NM_001004818

ATGGGAGACTGGAACTTGCTGGGAAAGCTTCTTGAGAGTGCCCAGGAACATTCTACGGTG

GTGGGCAAAGTCTGGCTGACTGTCCTCTTCATCTTCCGAATACTTGTCTTGGGAACAGCC

GCTGAAAAAGTCTGGGGGGACGAGCAGTCTGGTTTTACCTGTGACACTAAACAGCCCGGT

TGTCAAAACGTTTGCTACGATCACACGTTCCCCATTTCGCACATTCGATTCTGGGTGCTG

CAGATCATTTTCGTGTCCACTCCAACTCTCATCTACCTTGGCCACATATTGCATCTTGTG

CGCTTGGAAGAGAAGCGCAAGCAGAAGGAAAAGTTGCGGCAAAGCAGCAGAGACCCACAG

GATGACAAGCAGCTGCTCCTGGTGCCAAACAAGCCGGCCAAACCCCCTGTCAAGGATGAC

TTGGGCAAAGTGCGCCTGCGAGGGGCCCTGCTGCGCACATACGTCTTCAACGTCATTTTC

AAGACCATGTTTGAGGTAGGATTCATTGTGGCGCAATATTTTCTTTATGGATTCCAGCTG

CAGCCTCTCTACACCTGCAGCCGCTGGCCTTGCCCTAACACAGTTAACTGTTACATTTCT

CGACCTACTGAGAAAACCATATTTATTATTTTTATGTTGGCCGTTGCATGCTTGTCTCTA

CTGCTTAACCTGATTGAGGTTTACTATTTGGGATTCACAAAGTGCAGGCAAGGACTGTCC

CCATCCCATAATGAAACAGTAACCAAGTTACCAACTAAGACTAGCAGTACAACTCTCACC

CACATTATCCCCCACTATCCCCATTATTCTCCAACTGAGAAGGGATCTACATTCAGTCCA

ACTCTGGCATTTCCTCTGTCTCCAAGCAAGACCACGTCTAATGTAATGGACAGCAGCCAA

GCTGCGCGCAAACAAAACCGTGATAACCAGGCCGTGGAACGCAATGGGATTCATGATCTG

AACGTTGCATCTGAAAGCAGCAGTGAGTTGGACCAATTTGAGAAGCAAAGGCTCCCCAGC

AGAAACAGCAGGCACAGCAGCAACCGCTCTCGGCCTGGAGTCCTTGCAGTATAA

>Xt-gja3-NM_001114249

ATGGGCGACTGGAGTTTTCTGGGGAGACTATTGGAAAATGCTCAAGAACACTCAACAGTC

ATTGGCAAGGTTTGGTTGACAGTGCTCTTTATCTTCCGGATCCTGGTACTGGGAGCTGCA

GCAGAGGAGGTTTGGGGGGATGAACAGTCTGACTTCACTTGCAATACGCAGCAACCAGGT

TGTGAGAACGTCTGCTATGACAAAGCATTTCCTATTTCTCACATTAGGTTCTGGGTCCTC

CAGATTATTTTTGTGTCTACTCCTACCCTTATCTACCTGGGCCATGTCCTGCACATTGTG

CGAATGGAAGAAAAGAGGAAAGAGAAAGAGGAGGAGCTAAAGAAGTGCATTAAAGGTGCC

AGTGAAAAGGAACATTTAATAAAGGGTGGTGAAAAGAAGGAAAAGCCACCTATTAGAGAC

GAGAGGGGAAAAATCAGGATAGGAGGTGCCCTTCTTCGCACCTATGTCTTTAATATCATC

TTCAAGACTTTGTTTGAGATTGGCTTTATTGTAGGACAGTATTTCTTATATGGTTTTCAG

CTAAAACCCCTATACAGGTGCAGCCGTTGGCCTTGTCCTAACACCGTGGACTGTTTTATT

TCACGGCCAACGGAAAAGACCATTTTTATCATATTTATGCTTGTAGTGGCTTGTGTGTCT

CTTTTGCTGAATATGTTAGAGATATATCATCTGGGGTGGAAAAAACTCAAGCAAGGCATG

ACAAACAGATACATCCCTGATCCTTCTTGCAACAAATCAGAACCTCCAAGCCCTCAAACT

GCCCCATCTAGCATGTCCTTTCCACCATACTATACTGATGTGGCAGCTGCACTTCCAGTC

TTGCATCATGCATATGCCAGGTTCTCTGCTCCAGACTTCAAAATAACATCTGTGCCGGAG

GAAGCTCTAGAGTCTCCTTCTCTGATGAGCCATCATGAACATCTCATAGCTGCATCTGAG

CAAAACTGGACCAATCTAGCAGTGGAACGTGAGAGAATGTCTAGGTCAAGCAGTGGCAGT

TCTTGCAGTGCTACTACCTCTGCCTTAGACAGTCTGAGAAATGATGGAGGATGTGAGGAG

ATGCTAACAACAGAAGCCAGTGGAAGCAGTGCAAGGGCAGAAGACAGACCTGTGACCACC

AGGGTGGAAATGCACCAGCCGCCTGTCATGATAGACGCAAGAAGACTCAGCAGGTCTAGC

AGGTCCAGCAGTAGCAGAGCAAGGTCAGATGACTTAGCTGTCTAA

>Xt-gja4.1-NM_001130245-cx38

ATGGCAGGATGGGAGTTGTTAAAGCTCTTATTGGATGATGTTCAAGAACATTCCACACTT

ATTGGGAAAGTCTGGCTGACTGTACTTTTTATCTTCAGAATATTTATTCTGAGTGTTGCT

GGTGAGTCAGTTTGGACTGATGAGCAGTCAGATTTCATCTGTAACACTCAGCAGCCTGGC

TGTACCAATGTCTGTTATGATCAGGCCTTTCCTATTTCACATGTTCGCTACTGGGTGCTC

CAATTTCTCTTTGTTAGCACACCTACCTTGATCTACTTGGGTCATATGGTTTACCTGTCT

AAGAAGGAGGAGAAGGAAAGACAGAAGGAGAATGAGGCAAGCGAAGGACAGACAGAAGTA

CGTTCTTTTACTTCTAAGAAGATAAGGATTCAAGGGCCCTTAATGTGTACCTACACCACC

AGCGTAATCTTCAAAAGCCTTTTTGAAGCTGGGTTCCTTCTCGGCCAGTGGTACTTATAT

GGCTTTGTGATGTCTCCAATATATGTATGTGAACGGATCCCATGCAAACACCAAGTTGAG

TGTTTTGTCTCAAGGCCTATGGAAAAAACAATATTTATCATATTTATGCTGGTCGTTTCT

CTCATTTCTCTAATGTTAAATTTGATGGAGCTTATTCACTTAAGCTGTAAGTGTTTCCAC

CATAGTGTAAAAGAAGGAGCAACTTGTCCTCAAACAGGGGTTCCCTTGCATGGGTTAGGA

AACAGACTACCACCACAGGAATACATCAATCCCCCTCCTAGCAACCAAGACATTGATCTG

CCAGCTTATAACAAAATCCCTGGGGGGCAGAATTGGTCAACTATCCAGATAGAACAGCAA

GCGAATGGTCTTTTGAAACCCAAGTGTCAGTGTGACTGTTGGTCCCAAAGTGCTATTTCT

GTTGTGGTTTCTGGAGCACCAGGTATAGTCCCCAATTTGGATGCTATCAAAAGTAATCAC

CAGACCTCTTCCAAGCAGCAGTATGTTTAA

>Xt-gja4.2-NM_001079436-gja4

ATGGGAGACTGGGAGTTTCTTGAAAAACTATTGGATCAGGTGCAAGAGCATTCCACATCT

ATCGGGAAGATCTGGCTTATGGTGCTTTTCATCTTCCGAATCTTGATCTTGGGTCTGGCT

GGGGAGTCTGTTTGGGGAGATGAACAGTCTGATTTTATCTGTAATACAGAGCAGCCCGGA

TGCACCAATGTTTGCTATGACAAAGCCTTCCCCATATCTCATGTTCGCTACTGGGTACTT

CAGTTTCTATTTGTCAGTACACCCACATTGTTCTACTTGGGACATGTAATCTATCTTTCA

AGGAGGGAGGAAAAGTTGAAACAAAAGGAAAGTGAACTGAGGGCTTTGGATGACAAAGAG

CAAGTTGAACAGGCTATAGCTATTATAGAAAAGAAAAAAATGAAACTGTATATCCAAGAA

GATGGGACAGTCAAGATCAAAGGAGCCCTAATGTGCACCTATCTTACTAGTGTTATCTTC

AAAAGCCTATTTGAAGCTGGATTTCTTCTTGGCCAATGGTACCTTTATGGGTTTGTGATG

ACTCCAATTTATGTCTGTGAAAGAGTACCGTGTCCCCACAAAGTAGACTGCTTTGTATCT

CGACCCATGGAAAAAACTATTTTCATTGTTTTCATGTTAGTGGTGTCTCTCATATCTTTA

TTTCTAAATGTACTGGAACTTATTCATCTAGTCTGTAAGAGCATGATCGATACCTTGAAG

AAATATTCACCGTATGTCCCTCCAAACCGATACCCCAAGAATGAGGACACATATCCAGAA

AAGACATCAGAAACAGCCACAGCACCATTCCAAGACAAATCATATATTTACCTTCCCATG

AATGATAATATCTCATACCCACAGTACAAGATGCCAAATGAGCAGAATTGGGCAAACTTT

AATACAGAGCACCAGCTAGCTATAAGTGGTAATAACCAGTCACCACTAGGCCATTACAGC

CTCAGTGCCTTCGTGCCTGTGCCTCCAAAAACTCATTCAACAATGGAAAAGCCTAGCACC

AGAGCCAGTAGTTCTGCTTCCAAGAAGCAATATGTTTGA

>Xt-gja5-XM_004918216

ATGGGCGACTGGAGCTTCCTTGGCCAGTTCCTTGAAGAGGTGCAGAAACATTCCACGGTG

GTGGGCAAGGTCTGGCTGACCATTCTGTTCATCTTCCGGATGTTGGTGTTGGGCACGGCG

GCGGAATCCTCCTGGGGGGACGAACAGTCGGACTTTACGTGCGACACGAACACGCCCGGT

TGCGAGAACGTCTGCTACGACAAAGCGTTCCCCATCTCCCATATCCGATACTGGGTGCTC

CAGATCATCTTCGTCTCCACCCCCTCCCTGCTGTACATGGGACACGCCATCCACACCGTC

CGCGTCCAGGAGAAGAAGAAGCACCGGGAAGAGGAGAAAGCGAAGCAGAACCTGAGCGGG

GAGCCGTCCTACCATCAGAAGGAATATGCGGTGGAGAAAGAGGAGATCGTTTACACCGAC

GAGTTGAGCGGCAGGATCCGGCTTCAGGGGACGTTGCTGAACACCTACATATGCAGCATC

CTCATCCGCACCCTGATGGAAGTTGCTTTTATCGTGGGACAGTATATGCTCTATGGAATA

TTCCTGGAAACCCTGTACACGTGCCGGAGGTCGCCCTGCCCGCAGCCCGTCAACTGCTTT

GTCTCTAGGCCGACGGAGAAGAACGTTTTCATTGTATTTATGATGGCTGTGGGGGTGCTC

TCTCTCCTTCTCAGCCTCGTAGAACTCTACTACCTGGCGTGGAAAAAGATCAAGCACAAA

TTTGCCGGTGCCGGTACGCACAACCCGAGCGCCCTCAGGGTCGCAGCCGTAGAGCCGGGG

CACCGTTCCCAAAGCTGCACTCCGCCTCCGGACTTTAACCAGTGTTTAAGCAGCAATAAG

GGTAAGTTTGAGAGCCCTTTCAGCAATACGTTGGCTTCCCAGCAGAACACGGCCAACTTT

GCAACGGAAAAGGTCCAGGGCGAAGAAGACATTCGAGACGGGCAATTTATCCACATAAGC

TATGCACAAAATGCACCGGTATCCAACCACTCCACTCCGCACCATACACCGAACGGCTAC

TGCCAGAGCCCCCGAAGCCTGAGCAAGACCAGCCAAGCCAGCAGCAAAGCCCGTTCAGAT

GACTTGTCTGTGTGA

>Xt-gja8-XM_004918218

atgggagactggagtttcttgggtaatattttggaagaagtcaatgagcactcgaccgtg

attggtcgagtttggctcactgttctcttcatttttcggatactcattctgggaacggct

gctgagtttgtttggggcgatgaacaatctgattttgtgtgcaacacccagcagcccggt

tgtgaaaatgtctgctacgacgaggcctttcctctctcccacattaggctgtgggttctc

cagatcatctttgtgtccacgccatcactggtctacgtgggacacgcggtgcaccacgtt

cgcatggaggagaaacggaaggagagagaggaggctgaaatgagcaggcagcaggagatg

aacgaggaacgattaccccttgcccctgatcaagggagcatcaggaccacgaaggagaca

agtaccaaaggaaccaagaagtttcgcttggaaggaaccctcctcagaacctacatctgc

cacattatattcaaaactctttttgaagtcggctttgtggtcggccaatactttctctac

ggcttctggatcaaacccttgtatcgttgcagtagatggccctgcccaaacacggtggac

tgcttcgtttccaggccgacggagaaaacgattttcattacgttcatgttagccgtggct

gcggtctctctctttttgaacgtggtggagattagccacttgggatggaaaaagatccga

cttgcttttcgcagacccgaccaacatccccagcaactgccaggagaggtttcccagaaa

tcattgcattctatagcaatttcttctattccaaagtctaaaggctataagctgctcgac

gaagagaaagtagtctcgcgtttctacccgatgaccgaagtcggattagaagccagcccg

cttccggcgcacttcaacggctatgacaaaggcagcacagggctcctagaagacatctca

aagggttatgatgaaaccctgccatcctaccgtcaagcggaagagctagatggtgttaaa

gtgcaagcttctgatgcttttgaacaggcttcttctgaacgtgctgcctcagtgaggtcc

ccttcggtgcgtgccccatctgaccgagccccctctgaacgtgcactttctgaaagggca

cccacccgtgctgcttctgagcatgcaggttcagagcacacaccctctgtgcgaacacct

tccaacagtgcaactcccgaacgagtaccctcccgagctgcctctgaacatgcaccattg

gattgtgccccttctgagcgagcaccctcccgtgctgcctctgaacatgccccttctgat

cgtgcaccctcggagcgaggttcttcacgagcagcttcggagcgggctccatcccaaccc

gctctctttgaaccagtttcttctagaacaggctcagtccaagctctgttagagagcccc

ccttcctatgcaatatctgatctgccaccagaagcagatgccccgattgaattgataacc

ttaaaagcaccttctaaacagtctctaattgaccaagctcccactcaggtagggttagat

caatcccccaatgagcccacgccattaagagctcttccagagcaaggcaaccccaaccac

acgttccctgaccctgaagctttagaaccagatattgttgaatatgagcccctaggccca

gagatgccattactcaatctcgaattcattcccgactccagatccctgagcaggttgagc

aaagctagcagcagggcccgctcagacgacttaacggtctga

>Xt-gja9-XM_002938738

ATGGGAGACTGGAATTTCCTTGGGGTCATCCTAGAAGAAGTTCACATCCATTCCACAATT

GTTGGAAAAATTTGGCTTACAATTCTGTTTATATTCCGCATGCTTGTATTGGGAGTGGCA

GCTGAAGAGGTTTGGAATGATGAGCAGTCGCAGTTTGTATGTAACACAGACCAACCTGGA

TGCAAAAATGTTTGTTATGACCAGGCTTTTCCTATCTCACTTATCAGATATTGGGTTCTG

CAAGTAATATTTGTATCCTCACCTTCTTTGGTTTACATGGGCCATGCTATTTACAGGCTG

AGAGCATTGGAGAAAGAACGACATAGGAAAAAAGCTCAGCTTAGAGGGGAGCTTGAAGGA

GTTGAGTATGATCTGAGTGAAGACCGCAGAAGGTTGGAGCGTGAACTCAGACACTTAGAG

CAAAAAAGGCTGAATAAGGCTCCATTACGGGGTTCTCTTCTCTTCACCTATGTAATACAT

ATTTTTACAAGATCTGCTGTCGAGATTGGTTTTATGATTGGACAGCATTTGCTTTATGGA

GTTCAGCTGAATCCTCTTTACAAGTGTGAAAGAGACCCATGTCCCAATGTTGTTGATTGT

TTTGTATCAAGGCCTACAGAAAAAACTGTATTTATGCTTTTTATGCAGTGCATAGCAGCT

GTCTCTTTGTTCCTAAATATTTTGGAAATTGCACATCTTGGGGTTAAAAGAATAAAAAAA

GCATATATGATGAGGTATAAAATGAAAGAAGAGTTTGATGACTTTTATGTAATTAAACCC

AAGAAAACCTCCACAGTAAGCCACACTTGCATAGGTACGTCCTCTAGTCCACAAAAGACT

CTTCCTTCTGCACCTAGCAATTATACATTGTTGATAGAAAAACAGAGTGACCCCACTGTG

TATACCGTTTTAAATCCTCCATGTGGGTACCATCCCATAGAAGATGGTCAGAATGATATT

TGTAATACTTTCATGGAGGATGAGCAAGAAACAAAATCAGCCAATGGTATCAATGCCACA

AACACAGTTGAAAGTCACTATCATGACAACCTTGACAATAATAATGGGAGGATAAACAAA

ACATTAGATGCAGATGTTAAGACTTTGCATTGCCATGGAGAAAGAAGAACTGGTTGTATA

AGAAGTACTTATGTAGAAAATGCTCCTCTTATACAGACAACAACAACTGATGTACCATCT

GATGACACTGTGGATTACTCCTTACAAAATATATCCTGTCCAATGCCCACTAACGTACTC

CGGAAATCCAGAAGAGTCAGCGCTCCATGGAATGGTTCTAAAGCAGCAGAGAGAGGAGGC

TTTGTTCAAGATTCTGGATGCAGGGGGCGCAGTAGCTTCAGTGCAAATTTATCATGGGGA

CTCTCCAAGTCTGATCTGAAACGAGTTAGCAGGCCATCCACACCAGAAGTAATGGGAGAA

ATGAGCCCAAAATCCAAACAGGATAAAACATGTGAAGGTTCTGTGACCTTCTCTCCATCA

CGACGGATGTCACTGGCAAGTAATGCCAGCAGCCGGCGTGCCCCAACTGATCTACAGATC

TAA

>Xt-gja10-XM_018094017

ATGGGGGACTGGAATTTGCTGGGAAGCATACTGGAGGAGGTGCACATCCATTCCACCATT

GTCGGAAAGATCTGGCTGACAATTCTTTTTGTATTCCGGATGCTTGTCCTTGGGGTGGCT

GCCGAAGATGTCTGGGATGACGAACAAGATGAATTTATTTGTAATACTGAGCAACCAGGC

TGCAGGAATGTTTGTTATGACCAAGCTTTCCCAATCTCACTTATCAGGTACTGGGTACTG

CAAATTATATTTGTCTCTTCTCCATCCCTTGTTTACATGGGCCATGCACTGTACAGACTG

AGGGCTCTGGAAAAGGAGAGGCAAAAGAAGAAAGCCCAACTTAGAGCAGAGCTGGAGGAA

CTTGAAGTGGTTGTTGATGAGCACAGAAAACTGGAGAGGGAACTTAGGAAATTAGAAGAA

CAAAGGAAAGTAAACAAGGCTCCATTGAGGGGGTCCCTATTACGCACATATGTTCTGCAC

ATACTGACAAGGTCGGTAGTAGAAGTTGCTTTTATGATAGGGCAGTACTTGCTGTATGGA

TTTGACCTAGACCCCTTGTATAAATGTTCACGGTCCCCTTGCCCTAATATAGTGGACTGC

TTTGTCTCAAGACCAACAGAAAAGACTATTTTTATGGTATTTATGCACAGTATAGCAGCA

GTGTCATTGTTCCTAAATGTTTTAGAAATCTTCCATTTAGGCATTAGAAAAATAAAGCAG

GGGCTTTCTGGTAAATCCAATGCTGAGACAACAGAGGATGAAATAAGCAGCTGCAAGTCC

AAGAAGAACTCTATGCAGCCTGTTTGTATCATGAGCAATTCCTCCCCAAGTAAGATCATC

CCCTTGTCTTCAAGTGGCTACAAACTGCTGCCTGATCAACAAATGGATCCGGTAGGACTA

CCAGCTTACTTGCCCCCATCCCAGGACTTTAAGGAGGCACAAATGATGAGTGATCAAAAT

CATTATGGGAAAGTTCTTGCCCCTGACAAGCATAGGAAAACTGCAGACCATAGTTGCACT

GAGCATCAGCAGCAGGGAAGCCATTGCAAGCAAATCCTCCACTCTGCAGAGCCCCACAGG

ATACCCAATCACACTGACCACTACCATGGGCACATGACACACAGGATACCCACAATTCAG

CCTACCCCTGCCCTCCAGAAGCGCAATCTGTCCTCCAGCAGTGAAGATTCCCAAAAGAAA

CTTAAACACAACACCTGTTTGGGCTCAAAAAGTTGCCTCAAAAGCCAGCATTCCCTGGAT

CAGCCCAGGCACAGTTCTGTGCACCCACTAAGGTCATGTCTGCACTCCAGCCACATGGAG

CTTCACTCTGCACTACGAAAGTACAGCAGGGTTGGCAGCTGCAAGGACTTTGCTGAGGAT

CGCAGTGATTCTGCAGACAGTGGACATAGAAAAGTGAGTTCTACATCCAGGGGATTATCC

GAGAGCAGACTGATCAGTGACACTGAGAGTTTGGACTCTAGAAATGGCTCTGGCTCTGAA

TCTAGAAGGAGAGACGATAGTCCAAGCATCACTCCACCTCCACCATCTGGTCGCAGGATG

TCAATGGCAAGTAAAGCAGCCAGCCCTGGGCTAATACTGGCCAGTGGTTCCCACTCTCAG

TCTAGGCACCCTCATTATCATTATTACATATTACAAACAGTAAACAAAAATCCCTTAATA

AAAAGGTCATAG

>Xt-gjb1-NM_001001240

ATGAATTGGGCAGGATTATATGCCATCCTGAGTGGCGTAAACCGCCACTCTACTTCAATT

GGACGCATATGGCTCTCAGTGGTCTTCATCTTTCGCATCATGGTCCTCGTGGTGGCTGCA

GAAAGTGTATGGGGGGATGAGAAGTCTGCGTTTACGTGCAACACACAACAGCCTGGTTGC

AACAGTGTATGCTATGATCACTTCTTCCCCATCTCCCATATCCGTCTGTGGGCCCTTCAG

CTCATTATTGTGTCCACCCCTGCCCTTCTTGTGGCCATGCATGTGGCTCACCTACAGCAC

CAAGAGAAGAAGGAACTACGTATGACCGGCCATGTGAAGGACCAGGAGCTGGCAGAAGTG

AAGAAACACAAAGTCAAGATCTCCGGCACCTTGTGGTGGACCTACATCATTAGTGTTTTC

TTCAGAATCATATTTGAGGCAGCATTTATGTACCTCTTCTACCTCATCTACCCCGGTTAC

TCCATGATCCGTCTTGTTAAGTGCGATGCCTATCCCTGCCCCAACACTGTTGATTGTTTT

GTTTCTCGTCCCACGGAGAAGACCATATTCACAGTCTTTATGCTCGTTGCCTCTGGATTC

TGCATCTTTCTGAATGTTGCTGAGATAGTCTTCCTCATTGCCCAGGCCTGCTCCCGCAAA

GTCCATCGCAGACATTCTGACAGCATCAGCAAGGAGCATCAACAGAATGAAATGAACTTA

CTGATAACGGGAGGTAGTCTCATGAAACGGTCCCCTGGGGGGCAGGAGAAAGGAGATCAC

TGTTCTACTTCCTAG

>Xt-gjb2-NM_001016984

ATGGATTGGGGAACGCTCTATGAGGTTATTGGCGGAGTGAACAGACATTCTACCAGCATT

GGCAAGATCTGGCTTTCGGTTCTCTTCATCTTCCGCATCATGATCCTTGTTGTGGCTGCC

GAGAGCGTGTGGGGGGATGAGCAGTCGGATTTCACCTGCAACACCTTACAGCCCGGATGC

AAGAATGTCTGCTATGACCATCATTTTCCAGTTTCTCACATCAGGTTGTGGTGCCTGCAG

CTTATATTCGTTGCCACCCCTGCCCTTTTGGTAGCCATGCACGTGGCCTATCTAAAACAT

GAAGAGAAGAGGCAGTTGCAGAAGAAGGGAACCTTTGGGGGAAAGGACTTGGAAGAGTTG

AAGCTAAAGAAGGTCAGAATCAGGGGAACCCTATGGTGGACCTACACAACAAGCATCTTA

TTCAGAGTCCTCTTTGAAGCTGCCTTCATGTACCTATTCTACTTTCTGTATTCTGGCTTC

CAAATGCAGAGGCTCGTCCAGTGTAGCAACTGGCCGTGCCCCAACGTTGTCGACTGTTTC

ATTTCCAGGCCTACAGAAAAGACTGTGTTCACTATATTCATGATTATCGTGTCTGGTATC

TGCATGGTCCTGAATGTGATGGAGCTTTGTTACCTAATCGTGCAGGCTTCCCTAAGGAGG

TCAAAAAAACATTCACTCAGGCATTCTAACCACATGTCTGGCAAGGAGGAGAAGCAGAAC

ATCATGAATGAGCAAAAAGAGCAATAG

>Xt-gjb3-NM_001112900

ATGGATTGGAAGACATTTCAGGGTATCCTGAGTGGTGTGAATAAGTACTCAACAGCATTT

GGTCGCATTTGGTTATCGGTGGTTTTTGTGTTCCGAGTACTTGTTTATGTGGTTGCAGCA

GAGAAAGTTTGGGGCGATGAACAAAAAGATTTTGACTGCAATACTCGGCAGCCAGGGTGC

ACCAATGTCTGCTATGACCACTACTTTCCCATATCACACATCAGGCTCTGGGCCCTCCAG

CTTATATTTGTTACCTGCCCGTCTCTATTGGTTATAATGCATGTTTCCTATAGAGAGGAC

CGGGAGAAAAAGAACCGCTTAAAAAATGGAGACAACTGCTCCAAGCTCTACTCCAACACA

GGAAAAAAGCATGGTGGCCTTTGGTGGACCTACCTCCTAAGTCTTTTCTTTAAGACTGGT

ATTGAAATTGTTTTCCTTTACATATTGCATAAAATGTGGGATAGCTTTGATCTGCCTCCC

CTAGTGAAATGTTCAAACGAACCCTGCCCTAATGTAGTAGACTGCTACATAGCCCGTCCC

ACAGAGAAAAGGGTGTTCACCTATTTTATGGTTGCTGCTTCTGCTCTGTGCATCATCCTA

AACATCTGCGAAATATTCTACCTTATATTTAAAAGGATACTGAATTGCATGAACAAATAC

AAGAAGTCAAGCAAGCACTCTGTGACCTACAGCAAAGCTTCCACCTGCCAGTGCCACATC

CATCTAGACACCATTGAAAAGAAACCCATGAAC

>Xt-gjb5-XM_018091588-gjb4

ATGAACTGGGGTGTGTATGAAGCACTTATTACAGGTGTAAATAAGTACTCTACAGAGTTT

GGACGTCTTTGGCTTTCTATTGTCTTTATTTTTCGGTTTCTGGTGTACGCACTCACAGCA

GGTCGGGTTTGGGGCGATGACCAGAAAGAATTTGTCTGTAACACCCGTCAGCCAGGATGT

ACCAATGTTTGCTTCGACTACTACTTCCCTGTATCCCATGTCCGCCTCTGGGCTCTTCAG

TTGATTTTTGTAACATGTCCATCCCTCATAGTTGTTATGCATGTGGCTTACAGGGAGAAC

CGAGAACAAAAGAGAAGGGAAAAATTAGGAAAGGACTGTGAAAAAATTTACATGGATACT

ACAAAGAAGAGAGGAGGACTGTGGTGGACCTACCTCATCAGTCTTTTTTTCAAGGCCGCA

GTGGACTCAGTATTTATCTACGTTTTTTATCGGCTCTATGAAAATTTCTTTCTGTCTCGT

TTAGTGAAATGCTCTCTTAATCCTTGCCCTAACATTGTGGATTGCTATATATCTAGGCCT

TCAGAGAAAAACATCTTCACTCTCTTCATGATCATCACCTCTGCTGTTTGCATTCTCTTG

AACCTAGTTGAGGCTACCTATCTGATTGGCAAGAAGTGCAAGGAAACAATACATAAAGGG

AATAATATGACTCTTCAAGAGAGAATTGTGAAAAGTGGCCATAAAGACCAAGAAAACAAA

ACTTGCAAAGAGCAAATTAAGGTTGGAATACATTGTAAGACCTCAAATATCACTAAAATA

AAAGTAGACAATATTTCAAAGTAA

>Xt-gjb4-XM_004911610-gjb5

ATGAACTGGGCAATCTTTCAAGGTCTCCTCAGTGGTGTGAGCACTTACTCTACTGCCTTT

GGCCGCATATGGCTTTCCGTTGTATTCATTTTCAGGCTTATGGTCTATGTGGTTGCAGCT

GAAAGGGTTTGGGGTGATGAACAGGGAGAGTTTGACTGCAATACTAAACAACCAGGATGC

ACCAATGTCTGCTATGATTATTATTTCCCTGTCTCACATATTCGACTTTGGGCTCTCCAG

CTCATCCTGGTCACCTGTCCATCCCTTCTTGTCATAATGCACGTTGCTTACCGAGAGGAA

CGGGAGAGAAAACACAGAGAAAAACAGGGAGAGAATTGCGGCCGACTTTATCTAGACGTA

GGAAAAAAGAGAGGAGGCCTGTGGTGGACATATCTATTAAGCCTTCTAGCTAAAGCATCA

GTGGATTCAGTTTTCCTCTATGTCTTTCATCGGATCTACAACAACTATTCTCTCCCAAAT

TTGGTTAAATGCTCCATAAAGCCTTGTCCAAATACTGTTGACTGTTTTATATCAAGGCCC

ACAGAAAAAAACCTATTTACCCTATTTATGGTTGCCACTACAATACTCTGCATTTTTTTA

AATCTTTGTGAGATGATATATCTAATTGCCAAAAGGTGTCGAGAGTGCCTGGGACACAGG

AACATAAAATCCAAAACTCGCTCCAACAATGTCTATGCATACATTGAGAAGCAAGAGATT

TTCCATGCCAACAACAAGATAAATGAAAAAATGTCTCGGCTAAATCCATCCATTAGATTG

ACACCGCCACAGACTGAATTAAAATAA

>Xt-gjb7-XM_004914539

ATGAGTTGGTCATTTTTGCGTGATATCTTGAGCGGAGTGAATAAATATTCCACAGGCATT

GGAAGAATCTGGCTTTCGGTTGTGTTCATTTTCCGACTTTTGGTCTATGTCGTTGCTGCA

GAAGCTGTGTGGAAAGATGAACAGAAAGAATTTGAGTGCAACATCAGACAGCCCGGGTGC

GAAAATGTCTGCTTTGATCAGTTCTTCCCCATCTCCCAGGTGCGCCTTTGGGCTCTACAG

CTGATTATGGTGTCAACACCCTCGCTTCTTGTTGTTCTTCATGTGGCCTATCGGGAAAGC

AGGGAGAAGAGGCACAACAAGAAGCTGTATGCCAACACTGCAGACATGGATGGGGGATTA

TGGTGTACATATGTCATAAGTCTTCTCTTTAAGACTGTGTTTGAATTTGGATTTCTTGTA

CTTTTCTACAAACTGTATGGTGGTTTCAGTGTGCCACGACTGGTGAAATGTGATATGGAT

CCCTGCCCCAATGTTGTGGACTGCTATATCTCCAAACCTACAGAGAAGATGATTTTTCTC

TATTTTGTGGTCACAACCTCAGGACTTTGCATTTTGCTTAATGTTAGCGAGTTATTTTAC

CTTATTTTCAGATACTGTACAAAATTCTACCTGAAAAAGCAGGCAAACAAAATGGCACAA

GAAACATGTGATTGCAAGAATAAGCAACATAACCTAATACCCATTACATCTGAGCAGGCG

GGCACTGATGTGCCTGATGGTTAA

>Xt-gjc1-XM_002935563

ATGAGCTGGAGCTTCTTGACGCGCCTCCTAGAAGAAATCCATAACCACTCAACTTTTGTG

GGCAAGATTTGGTTGACTGTTCTGATTGTCTTCCGCATTGTGTTGACTGTTGTGGGAGGA

GAGTCCATCTACTACGATGAGCAAGCCAAGTTTGTGTGCAACACTGGGCAACCGGGCTGC

GAGAACGTGTGTTATAATGCGTTTGCTCCCCTCTCCCACGTTCGCTTCTGGGTTTTCCAA

ATCATCTTAGTGTCTACACCATCCCTCTTTTACCTCGGATACGCCATCCACAAAATAGCA

AGGACGGAGGACCACATGGAGCAAGAAAGAAGGACAAAGCACAGAATGTTCCCGATGCGC

TGGAAGCAGCAACAAGGCATAGGGAATGCTGACGATGACAATGAAGAGGACCCCATGATG

TACCCCGAAACTGACATTGTAAGCGAGAGGAGCAAGCGTGAATGTATGCACAGCAGGGTA

AAACACGACGGGCGACGGCGCATCCATGCTGATGGACTGATGAGGATTTACGTTCTTCAG

CTACTTTTCAGAACATTTTTTGAAGTGGGATTTCTGGCTGGACAGTTCTTCCTGTATGGG

TTTGAAGTCACCCCAATATATGTGTGTGATACAAAGCCTTGCCCTCACAATGTGGATTGC

TTCATATCTCGACCCACTGAAAAGACCATATTCTTGCTTATTATGTATGGAGTAAGCGGC

CTTTGCTTGCTCCTCAATGTCTGGGAAATGCTCCACCTCGGGTGCGGGGCGATACTTGAC

GCGATTAACAGTAGGAAGAAGGAGTCTGATGATACCTCTGCTTACAATTACCCTTTCACG

TGGAACACCCCATCAGCTCCCCCCGGGTACAATATTGTTGTGAAACCTGATCAAATACAG

TTCACAGATCTCTCAAATGCCAAAATGGCTTACAAACAGAACAGGGCCAACATCGCTCAG

GAGCAACAATATGGCAGCAGTGAGGACAATATCCCCGCCAGTCTGGAGCACTTGCAACGG

GAAATCAGGGTTGCTCAGGAGAGGCTACAAATGGCCATCCAAGCCTACAACAGCCAAAGC

CATCTGCCCTCCTCCAAAGAAAAGAAGATCAAGAAGGGCTCCAACAAGAGCAGCGTCAGC

AGCCGCTCTGGGGATGGAAAGATGTCTGTGTGGATTTAA

>Xt-gjc2-XM_031903438

ATGAGCTGGAGCTTTTTAACCCGTCTTTTAGAAGAAATTCACAATCACTCCACCTTTGTG

GGGAAGGTGTGGCTCACGGTTCTGATCATCTTCAGGATAGTCCTGACAGCAGTGGGAGGG

GAGTCTATCTATTCGGATGAACAGAGCAAGTTCATGTGCAACACCAAGCAGCCGGGCTGC

GACAATGTCTGCTACGATGCCTTTGCCCCTCTTTCCCACGTCCGCTTTTGGGTCTTCCAG

ATCATCATGATATCTGCCCCTTCTGTAATGTATCTCGGCTACGCTATACACAGGATCGCC

AGGTCGGCAGAAGAAGATCGCCGGCACCATTCATGTAGGAAGAAAAAGTCGTTTTCCAAA

TGGCGGCCAGGCCAAAATGCTGAGGACCCGGAAGCCGAAGAAGAAGAACCTATGATATAC

GATGGCCCATCTGAGCCAGAGAAGGTGGAAAGCAAAGAAAAGGAAACGAAAAAGCACGAC

GGTCGGAGGCGCATCAAAGAGGAGGGGTTGATGAAAATTTATGTTTTCCAGTTGGTATCA

AGGGCCATTTTTGAAGTGAGTTTTTTGGTAGGCCAATACTTCTTATATGGGTTTCGAGTC

AAGGCCTATTTCGAGTGCAGCACTAACCCGTGCCCCCATAAGGTCGACTGCTTTGTTTCA

CGACCAACGGAGAAGACGGTTTTCCTTCTAATAATGTATGTGGTGAGTTGCCTTTGCTTG

TTCCTTAATGTCTGTGAGATGTTGCATCTCGGCGTTGGAACCCTGCGGGACGCTATCCGC

AACAGGAGAACTCCTAGAAACAAGCAGGCCCCGTACAATTATTCTTATCCGAAGAACATC

ACTTGTCCTCCAGAATACAACATGGTGGTCAAGTCGGACAAATCTACCAAAATCCCAGTA

GGCCTAATGGCTCATGAGCAGAACTTGGCCAACGTTGCTCAGGAACAAAACTGCCCCAGT

GACAACGCCCCTTCTGACCTGTCTGCTTTGCATAAACATTTGAGCATTGCTCAGGAACAA

TTAGATATAGCCTTTCAGAACTATAATACTCACGTCAACGGCCAGCCGTCCAGGACGAGC

AGTCCGGCTTCTGGTGGGACGGTGGTGGAGCAGAACCGAGCCAACACCGCTCACGAAAAG

CAAGGAGCCAAGCCCAAATGCGGCTCAGACAAGGGTAGTACCAGCAGTAAAGAAGGAAAA

ACCTCCGTATGGATATAA

>Xt-gjc4-NM_205831-gja7-cx45 This entry sequence suggests an intron of 3200 nt in the 3'-tail. We have cut the sequence at the end of exon 1.

ATGAGCTGGAGTTTCTTAACGCGTCTCCTCGAGGAGATCAACAACCACTCGACGTTCGTC

GGGAAGGTCTGGCTGACGGTGCTCATCATATTCCGGATCGTCCTGACCGCGGTCGGCGGG

GAGTCCATATACTACGATGAACAGAGTAAATTCACGTGCAACACGCAGCAACCCGGTTGC

GAGAACGTGTGCTACGACGCCTTTGCGCCCCTGTCGCACGTTCGGTTCTGGGTCTTCCAG

ATCATCCTCATCACCACCCCGTCCATTATGTACCTGGGGTTCGCCATGCACCGGATTGCG

CGCCAGCCCGAGATGCAGATAAGGCGCTCGGAGAAAACCAAAAGCAAAAAGCGCGCTCCC

ATAATTCATCGGGGGGCCATGAGAGACTACGAAGAGGCTGAAGATAACCAAGAGGAAGAT

CCCATGATTTGTGAAGAAGAGGAACCCGAGAAAGACAGCGAGAAGGGAGACAAAAAGAAG

CACGATGGGCGCCGGCGGATCAAACAAGATGGCCTGATGAAGGTCTACGTGTTGCAGTTG

CTCTTCCGTTCGGTGTTTGAAGTCGGCTTCCTGATGGGCCAGTACATACTGTATGGGTTC

GAGGTCATTCCCTTTTTCGTGTGCTCCCGGAAACCGTGCCCTCACACCGTCGACTGCTTC

GTGTCACGCCCCACCGAGAAGACCATCTTCCTGCTGATCATGTACGCCGTCAGTGCGCTC

TGCCTCTTCCTCAACCTGTGCGAGCTCTTCCACTTGGGCATAGGGGGCATCCGAGATGCC

CTCCGGCAGAAGAAGAAAGAACTTCAGGAATCCCGTAAAAAGACCCCCAGTGCCCCACCA

AATTACCACTCTGTCCTGAAGAAAGGCAGGCTGCCCAACGGGAAACCCGTTTTCCCAGGC

AACGGAGTGTCCGAAGGCTTCGAGTTGCCGACACACGAGCTGGACAGGTTACGGCAGCAC

CTCAAGTTGGCTCAGGAGCATCTCGACTTGGCTTTCCACCTCAACCCAACGGGCGATAAT

ACCCACGCGTCCCGGAGCAGCAGCCCCGAGTCCAACAGCATTGCCGCCGAACAGAATCGC

CTAAACTTGGCACAAGAGAAGGGAGTGGGAAACCGGGAAAAATCTG

>Xt-gjd1-XM_002939244-gjd2 Splice site

ATGGGTGAGTGGACCATCTTGGAGCGCCTGCTGGAGGCTGCCGTGCAGCAGCACTCTACA

ATGATAGGAAGGATCCTATTGACCGTAGTGGTTATATTCCGGATTCTAATTGTGGCTATC

GTTGGAGAGACAGTCTATGAAGATGAGCAGACAATGTTCATGTGCAATACACTTCAACCT

GGTTGTAACCAAGCGTGCTATGATAGAGCATTTCCCATTTCTCACATTCGCTACTGGGTA

TTTCAGATCATTTTGGTTTGCACACCCAGTCTCTGCTTCATTACCTACTCTGTGCACCAG

TCAGCCAAGCAGAAAGACCGAAGATATTCCTTTCTATATCCCTTATTGGAGAAGGAACTC

CCAAGAGAGAACAAAAGAATTAAAAATATTAACGGTATTCTAGTGCAAAATCAGGACATT

TCCTCCAAAGATGAGCCGGACTGCTTGGAGGTCAAGGAAATTCCAAACACTCCTAAGAAA

GCCGTCAAGAGTACAAAAGTCAAGCGTCAGGAGGGAATTTCTCGATTCTACATCATTCAG

GTCTTTTTCAGGAATGCTCTAGAGATTGGGTTTTTGGCTGGACAATATTTTCTGTATGGC

TTCAGTGTGCCTCCAATCTTTGAGTGTAACCGATACCCTTGTGTGAAGGAGGTAGAGTGC

TATGTTTCAAGGCCCACTGAAAAGACTGTCTTCCTAGTCTTTATGTTTGCTGTCAGTGGC

ATCTGTGTTCTCTTAAACTTAGCAGAACTAAACCACCTTGGGTGGAGAAAGATCAAGACA

GCTATGAGAGGGGTTCAAGCACGAAGAAAGTCAGTCTCCGATGTACGCAAAAAGGAGCCA

TCAACACTGGCCCAGGTTCCTACATTAGGTAGGACACAGTCCAGCGAGTCGGCTTATGTT

TGA

>Xt-gjd2-XM_002932834

ATGGGAGAATGGACTATCTTAGAAAGACTTTTGGAGGCTGCTGTGCAGCAGCACAGTACT

ATGATAGGAAGGATTCTGCTGACAGTGGTTGTCATCTTCCGGATACTTATTGTGGCCATT

GTTGGAGAGACTGTTTATGATGATGAGCAAACTATGTTTGTGTGCAACACTCTTCAGCCA

GGATGTAATCAGGCATGCTACGACAGGGCTTTTCCAATCTCACATATAAGATACTGGGTG

TTCCAAATCATCATGGTCTGTACACCAAGTCTCTGCTTTATTACTTACTCCGTCCACCAG

TCTGCCAAGCAGAGGGAGAGGAGGTACTCTACAGTCTTCTTAACGGTAGACAGAGACCCA

GACACTGTAAAACGAGAGGACAGCAAGAAGATTAAGAACACCTTGGTTAATGGTGTCCTG

CAAAACACAGAAAACTCCACCAAGGAGGCCGAGCCTGATTGCTTAGAGATCAAGGAAATC

CCAAACCCATCCATTCGAACCACCAAATCCAAGATGAGGCGCCAAGAAGGAATCTCAAGA

TTTTATATCATCCAGGTGGTCTTCAGAAATGCTCTGGAGATTGGTTTCCTAGTGGGACAA

TATTTCCTGTATGGGTTTAATGTCCCGTCCATGTACGAATGTGACAGATATCCTTGCATC

AAAGAGGTGGAGTGTTATGTGTCTAGGCCAACTGAGAAGACGGTATTCCTGGTTTTTATG

TTTGCGGTTAGTGGCCTCTGTGTTGTGCTCAATTTGGCAGAGCTGAACCACTTGGGCTGG

CGAAAGATCAAGATGGCAGTCAGGGGAGTACAAGCAAAAAGGAAATCCATATATGAGATC

AGGAATAAGGATTTGCCCAGAATGGGGGTGCCCAACTTTGGCAGGACTCAGTCCAGTGAC

TCAGCCTATGTGTGA

>Xt-gjd3-XM_002940217

ATGGGGGAGTGGGGCTTTCTGAGCTCATTGCTGGACGCAGTGCAGGAGCATTCTCCCATG

GTGGGACGTTTCTGGCTGGTGGTTATGCTCATATTCCGCATCCTTATCCTGGCCACGGTC

GGAAGCGACATGTTCGAAGACGAGCAAGAGGAGTTTGTGTGCAATACAATGCAGCCGGGT

TGCCGACAGGTTTGCTATGACCGTGCCTTCCCCATCTCCCACTATCGATTCTGGGTTTTT

CACATTGTGCTGTTATCAGCCCCGGCTGTACTCTTTGTCATTTACTCCATGCATCAAAAT

TCCAAGATGGGGAGAGACGCCGAAGAGGAGGAGGCTGCGAGAGCCGGACAGCAGGCCATC

GTCAGGTCAAAAACAAGGCCAGACCAGCGTGGGCGCCACATACGCACCTTTTATATCGTC

AATGTGGTTATGAGGATTTTGGCCGAGGTTGGATTTCTAGTCGGACAGTGGCTTCTCTAT

GGGTTTCGGGTGGACCCCCAATATCTGTGCCAACGTGAACCCTGCACGCATACTGTGGAT

TGCTTTGTATCAAGGCCCACGGAAAAGACAATCTTCCTCCAGTTTTACTTTGTGGTGGGC

GTCATCTCCGCTTTTCTTAGCGTGTGTGAACTTCTGCACATTCTGCTAAAGGGAAAGTGT

AGGGCAGCGGGTGAGGGGGACGGACCTAGCCCCCCGCCAGCCTATGAAAGGGACAACTGG

TCCAACAGGGAGCACGAGAGGACAAATCATTTCTTGCTCCAAAATCAAACACGGGACAAC

AGGGAAATCAGAAGACCCGACGGGCACCGCCTCTCCATCCCGTACTCCCAAAGCACAACC

TACAAGCTCAAGGTGGTTCCCAGCTCATCCAATAGTAGCAAATCTTCCAGGTTACTGGTC

AAAGGAGATTTGACTGTGTAG

>Xt-gjd4-NM_203753

ATGGAGTATTTAGACTCAGTGGGATATTTAATCATCACTTTTAACTACAATGTTACTGCA

ATTGGGAAGGTCTGGCTCACATTAATGCTTTTGCTGAGGATGGCTATGGTGTTATTTGCC

GGATACCCTCTTTATCAAGACGAGCAGGAGCGTTTTATCTGCAACACCTTACAGCCCGGC

TGCTCCAATGTCTGCTACGACATCTTCTCCCCTGTGTCCCACATGAGATTTTGGCTTTTC

CAGACCATGATAGTCTTTCTACCCTACGCTATGTTCGCTGTTCACATTTTGCACCGAGTG

GTTGGCTACATGGCACCAACAAATGATCACTTCCGGAAAAGCAACACCCCGACTTATCAT

GGCGTGGGCTTACAGATGGAGATACCTGATTTTTCCTTGGCGTATATAGTCCAATTACTT

CTGAGAACTCTTCTAGAGGCTGGTTTCTGCACGGGGCAGTATTTTCTCTTTGGGATATTA

GTTCCAAAGAGATTCAGCTGTTCTCAGGCCCCCTGCACCAGCAGTGTTGATTGTTACATC

TCTAGACCCACTGAAAAGTCTCTCATGATGATCTTTATCTGGGGGACAGGAGTGATATCT

CTCATACTTGGGCTGGTGGACCTCATTTACGTAATCCATCGGCGAAGGCGCTCCGAGCGC

CTAAAGAGAGAACTGCTTCTGCTGGAAAACGACTCCCTAAAAGGTGGCTGCTGTTCTGAT

TCACCTCCAAATTCAAACTTGCACGGCGCTCCGGAGCCAATGGCCTTGTACGCTGATTCT

GCTTCCAATGATCTTGAAAAAATAGAGGAGAAAATTAAGGGCGACTCCCATTCTCTGCCA

TCGTATGAAGGGGAAACGGCTTCTTTTCAGACAGAAAGTTCTCACCATGATGTGGGAAAG

TCCAACATGAACACCAACAGCAATAAATCATGTTCTTGGCAAGTCAGCCTGACTTCTGCC

GACGGCAAACCCACCAAGAGTGATCACCACTTGGTCACCCCAAGGCAGCTCAAACCAAAG

CAGGAGAATTGCAGCAGCTCTAATCTGGGAATACTCAGCCCCCCTGAAAAATCCCCAGCG

TCCAAATCCAAGAAATCTGAGTGGGTTTAA

>Xt-gjd5-NM_001097411-gjd2prov

ATGGGGGACTGGTCTATCTTGGGGCGTCTCTTGACAGAAGTCCAGAACCATTCCACTGTG

ATTGGAAAGATATGGCTGACAATGCTTCTTATTTTTCGCATTCTTTTGGTAACGTTGGTG

GGAGATGCAATGTATGGAGACGAACAGTCCAAATTTACCTGCAACACTCTTCAGCCTGGT

TGCAATAATGTGTGCTATGACAGTTTTGCTCCTGTTTCACACCTCAGGTTTTGGATATTT

CAGATTGTCCTGGTTTCAACACCTTCCATTTTTTATATCATCTATGTCCTGCATAAAATT

GCCAAGGATGAGAAGACTGAACTGGAAAAGGCCTATGTCATGGAGCTGATTCAGAGCCTA

TCTGCTGAAAAACACCTGTCTGAGAGTAATGTAAACCCAGATACTGAACAACAAATAGAT

GGTAAAGATGCTTCATGGAAAGAAACCGGATTTAAGCCAAAGTTAAGAAAAGATTTCAAA

GGGTCCATCCCAGTCCTTGACAAAATGCACATCATCTATATTGTCCACGTGGTACTCAGG

TCTCTGATGGAGGCTGGGTTCCTGGTGGGCCAGTATTATCTTTTTGGTTTTGAAGTGCCT

CACCTTTTCCGATGTCATACCTACCCTTGTCCAACTACAACTGACTGCTTTGTCTCAAGG

GCCACAGAAAAGACTGTTTTCCTAAACTTCATGTTTGGAGTTGGACTTGGCTGCTTCATT

CTGAACCTTGTGGAACTGCATTACTTAGGTTGGTTCTATGTGTTTCGCATTTTATCCTTA

GCCTGCTCTGCCTGCTGTGATTTTAAGAGTATCAAGAAGCCAAAGGTTCTACACCCAGAG

CAGAACAGCTTACTCATACACTTAAAGGACACCATACAAGAAAGGTTTGCGCTAAAGAGT

TCTTCCAGTTTATCCCAGGAGAAGATCAGTTGTCTCCCCTCTCATTCAGCTGCCATTTCC

TTTACACAGGATGACAACAGTGATACTACCTCCAGGAAAGGCCCAGACAGTATGCATAGT

AGCCTCGATAAAATATATAAAATCAAAAAATCTTGGCTTTAA

>Xt-gje1-XM_002933872

atgtctttaaattatatcaagaacttctatgaaggatgtctcaggcctccaacagtgatt

ggtcagttccatactctcttctttggttctgtgcgaatgttctttcttggtgtattgggc

tttgctgtatatggaaacgaagctttacattttagctgtgaacctgacaagagggaggtg

aacctgttttgttacaatcaatttcgaccgataactcctcaggtattttgggccctacag

ctagtgacagtattggtcccgggagctgtattccatctttatgcagcctgtaagagtatt

gatcaagaagacattctccagaagcctgtctatactgtatcttacattatatctgtgctg

ctaaggattatcctggaggtggtagcattttggcttcagagtcatctttttggttttcag

gtgaatccgatctttatgtgtgatgcgggaagtcttgataagaaatatgatctcaccagg

tgcatggtaccagaacactttgaaaaaacaatatttctcattgctatgtacacctttact

gtcattacaataatattgtgtgttgctgaaatctttgagattctgtgcagacggctaggt

ttcctaaacaatgagtaa

# Supplementary Figure S25. Tibetan frog (*Nanorana parkeri*) connexins.

The chronology of the sequences is according to the Greek nomenclature. Size nomenclature is indicated in parentheses. GenBank accession number is given for each entry.

>Np-gja1-XM_018563743

atgggtgactggagtgccttaggaagactccttgataaggtgcaggcctattccactgcc

ggagggaaagtgtggttgtctgttctgttcattttccggatcctgttattaggcactgca

gtagagtctgcttggggtgatgaacaatctgcattccgatgcaacactcaacaacctggt

tgcgaaaatgtctgctatgacaaatcatttccaatctctcatgtacgattctgggttctc

caaattatatttgtctctacacctacacttctatatttagcacatgtgttttatttgatg

cgcaaagaggagaaactgaaccgaaaagaagaagaactcaaaattgtccagaatgatgga

gggaatgtggacatgcatcttaagcaaattgaaataaagaaattcaaatatggcttggaa

gaacatggaaaggtcaagatgcgtggtggtctacttcgtacttatgtgataaccattctg

ttcaaatcattttttgaggttggtttccttctcatccaatggtacctctatgggttcagt

ttaaatgctatctatacctgcaaaagagatccctgcccacatcaagttgactgtttcctt

tcccgtcccactgagaaaaccatttttatctggtttatgctagtggtgtctttagtatca

cttgctttaaacatcattgagttgttctatgttggttttaaaagtgtcaaggatggcatt

aaaggaaagaaggacccctatgccaccacaaacgatgcagtgaatcctgccaaggattgt

ggttcccctaagtatgcatatttcaatggctgttcttctccaaccgcgcctatgtcacca

ccaggttacaagcttgttactgaagacaggaacccttcttcctgtcgtaattataacaaa

caagcaagcgaacaaaactgggctaattacagtgcagaacaaaccaggatgggccaagct

ggaagcactatttcaaacactcacgctcagccatttgactttccagatgaacaccagaat

gtaaaaaaaatgccaccaggacatgaaatgcagcctctcagtacacttgaccacaggcct

tcaagcagagcaagtagccatgcaagcagcaggcccagacctgatgacctggagatttaa

>Np-gja2-XM_018574717-gja3like The predicted cds contains some in-frame 5´ repeats (each of 33 nt, grey font) to our predicted start codon, and the repeats that have been excluded here

atgctcggcgagttggcgattctgcccggcact

atgcccggcgagttggcgattctgcccggcact

atgcccggcaagttggtctggctgacggtcctcttcatcttccggatcctggtcctgggg

tcggcggcggagaaggtctggggggacgagcagtccggcttcacctgcgacaccaagcag

cccggctgccagaacgtatgctacgaccggacgttccccatctcgcacatacgcttctgg

gtcctgcagatcatcttcgtgtccacgccgacgctcatctacctggggcacatcctgcac

ctggtgcgcatggaggagaagcagaagcagaaggagaagttgcgtcagagcaagggggac

ccccagggggaccggcagccgctcctggagccgccgaggcccgccaagccgtctgtgaaa

gatgacatgggcaaggtgcggctgcggggggcgctcctgcggacttacgtcttcaacgtc

ctcttcaagacgttgttcgaggtggggttcgtcgtggcgcagtacttactgtacgggttc

cagctgcagcccttgtacacgtgcagccggtggccttgccccaacacggtgaactgttac

atctcccggcccaccgaaaagaccatcttcattttattcatgttggccgttgcgtgcttg

tcgcttctgctcaacctcatcgagatctactacctggggttcaccaagtgcaggcagggg

ctgaccgggtcccgccgggaagccgtgccgagaccaccggccaaggcgggaagtatcacg

cacacgcacatcatccctcactacacccacttcagtcctcccgagaaagggtccagcttc

aacagccccctgcccttccccctctcccccaaaaaaaacacgacggccgccattgacagc

agccaggccgcccggatgcagaacagggagaaccaagcggcggaacgcaacggcatcggt

gacctgaacgtggcgtccgagagcggcagcgacttagaccaggccgagcgccagaggcta

ccgagccggaacagcaggcagagtagcaccagatcacggcccggggctctcgccgtgtga

>Np-gja3-XM_018554688

atgggtgactggagttttctgggcagactattagaaaatgcacaagaacactcaactgtc

attggaaaggtctggttgaccgttttatttattttccgaatattggttttaggagcagca

gcagaagaagtgtggggggatgaacagtctgactttacttgcaacacccagcaaccaggt

tgtgaaaatgtctgctatgacaaagcctttcctatttctcacattaggttctgggtactc

caaattattttcgtctctacacccaccctcatctacctgggccatgtcctgcacattgtg

cggatggaagaaaagaaaaaagagaaggaggaggagctaaagaagtgcgttaaagataat

gatgagaaggaactgctcctcaagaagagtgtcgagaagaaggaaaaacccccaatcaga

gatgaaaggggtaagataagaataggaggtgcactcctccgcacttatgtcttcaacatt

attttcaagactctgtttgagattggctttattgtagggcagtatttcctgtatggtttt

gagctgaaacccctctaccgttgcaaccgttggccttgccccaacactgtggactgcttc

atttccaggccgacagaaaagacaattttcattatatttatgcttgtagtggcttgcgtg

tctcttttgctgaatatgttagagatatatcatctggggtggaagaaactaaagcaaggt

atgaccaacagatatattccagatcctgcttgtaataaagcaaatcctcctagtctacta

ccacgaactgttccgccaagtatggcttttccgccatattatactgacgcagctgctgcc

cctccaaccatgcaacatgtatatgctaggtcacccatctctgattttaagatgacatca

ctaacagaagagcctcttgatccttcctacttcagcggtcattttgatcatcgtgcctta

gctagtgaccagaactgggccaacctggcggtggagagagaaaggaagcctggatccagc

attgccagtgtctgcagtgcaactacttctgctccaagcagtgtaagaaatgaggaagca

tgtgagcccccagtggggtcaggaagtggaaacaattcaagagcagaagcagatgacata

ccagccaccaccactgtagagatgcaccagcctcccgtgttgatagacacaaggaggctc

agcagggcaagcaaatcaagcagtagcagagctaggtcagatgacttagctgtgtag

>Np-gja4.1-XM_018558032-gja2like Np-gja4.1/2/3 are all on same scaffold (NW_017306584), located 10-20 K nt apart

atggcaggatgggatctacttaaaatattgcttgatgaagtccaagaacattccaccctt

atcggaaaggtctggttgactgtgcttttcatctttagaattctaattctcagcttggca

ggagactcagtttggggagatgaacagtctgatttcatctgcaatacagaacaacctggt

tgcaccaatgtctgctatgataaggcttttccaatctcgcatataaggtattgggttctg

cagttcctttttgtcagcactccaactttgatttaccttggacatgtaatttacctgtct

aggaaagaggagaaacggaaactcaggaataatgaaatgaaggccattgaagcgacagtg

tcaaaagtaaagagtgacagtaaaaatgaatctcctactaagaagtttaagattcaagga

gcattaatgtgtacctacacaattagtgttgtcttcaaaagtgtcttcgaagctggattt

gtttttggtcagtggtacctttatggctttgtgatgcctccagtctttgtgtgttctagg

atgccttgtccccacaaggtggactgttttgtctcccgtcctatggaaaaaactattttc

atcatctttatgctagtggtgtccctcatctctttgcttttgaacttgctggaacttgtc

cacctgggctgtaaacagtttcatcaaactgttaatactggagtacctttccatcctacc

aggtgctacacaaacacacagtcaatcaatcttccttatctccatcaatcttaccatcat

cctacagcaagcaaccaagaaacagagctgccagtttacaagaaccctgaaagtaccaat

tggcctagtattaacactgaagagcaggtgaactgtctagagaaagacaaggcatcctct

gagtgctggtcccaaagtggtttatctgtggtcgctactaatgctcctagtttaaaggcc

aacctggcttccaattcaagcagttgccagacatcaagcaaacaacagtacgtctaa

>Np-gja4.2-XM_018558045-gja4like

atgggagactgggagtttttagagaagttgttggatcaggtgcaggagcactccacaagt

attggaaaagtctggctaatggtgctttttgtgtttcgcatcctaattcttggtctggcg

ggagaatccgtttggggcgatgaacaatcagattttgtctgcaatacggatcagcctgga

tgtccaaatgtctgttatgacaaagccttccccatctctcatgtgcgcttctgggtgctt

cagtttctctttgttagcacccccacgttattttacttggcacacgttatttatctgtcc

agaaaagaagaaaagctgaagcaaaaagaggaagagctaagggctatgagtgacaaggac

cagcaagtggaccgtgcaatagctgtcattgagaagaaacgtctaaaaatctgtattcaa

gaggatggtaggatcaaaatccgaggagcgctaatgttgacatacacaatcagtgttatt

ttcaagagtatttttgaagctggtttcctgcttggccagtggtacttgtatggctttgtg

atgcccgcattatatgtgtgtgagagaaccccatgtcctcataaagtagactgttttgta

tctagacccatggagaaaactatcttcatccttttcatgctagtagtgtcactgatctct

ctagtactaaacctcctagaactgattcatctaatgtgtaagagtatggttcatgcctta

aaaaaatattccccctatgtgcctaatacaaaatacacaaaggaagaggatatttaccca

gggaaatgctctgaggcagccagtgcaccttttcaggacaagtcttacctgtacctcccc

atgactgagaacatttcctacccagcatacaaagtacagaatgaggacaactgggcaaac

ttcaatgctgagaaacatttatgcctcagtgcagacaaacaggcactagatcattacagc

aacagtccctttaaacctgtttctcccaactcccatgctgttgtcgagaagcagccaagc

agagccagcagttcggcctctaaaacacagtatgtctga

>Np-gja4.3-XM_018558047-gja4like

atgggggactggagctttcttgaaaagttactagaccaagtacaggagcattctacaaca

gttggaaaaatctggcttgtggtgctgtttatattccgtcttctgattttaagccttgcc

ggggaatcagtttggggagatgagcaatcagacttcatttgcaacactaaacaacctggt

tgtccaaatgtttgctatgataaagcttttcctatttctcatatccggtattgggtgcta

cagtttcttttcgtgagtacacctactctgttctacttggctcatgttgcctacctgtct

aggaaacaggaaaagctaaggcagaaagaaagccaatacatgattgtagaagacaaaatt

caacaagaggataccggcttgtcgcttgatgaaaagaaacacaggaaattattatttcaa

gaggatggaagagtcaagatcagaggagcactgttgtgcacatacatagcaagcattatc

tttaagagtctatttgaggctggattcataattggccaatggtatctctatggttttgtg

atgcaggcaaaatatgagtgtgaaagaccgccgtgccctcataaggtggactgctttgtg

tcaagacccatggaaaaaactatattcatccttttcatgcttgccgtctccctcatctct

ttgtttctcaacatagtagaattcgttgttttgatttctcacagtatatgtcgaaaagta

aaaaagtatgcaagagtcgcatataaaagtgatgcaggaatattccaagacaagacttac

aacttacctagtgcaccagagtctgacaaatcgtgtctgtattttcccacaatcggggat

aaatgccctccctataaaatttctgatgaggaaaattgggtcaacttcaatacagagaag

gagatggccctgaatggtgtagttcactcaaatctggacagtaatgtttacagccgtgac

actaaccaagtttctaaccgctctggcagttctatgtcaaagaagcagtatgtctaa

>Np-gja5-XM_018557860

atgggtgactggaccttcctgggggaattcttggaggaagtacagaagcattccacagtg

gtggggaaagtatggctgactatactgttcatcttccgaatgttggtcctcggcactgcg

gcagagtcctcctggggagatgagaaatcggacttcatgtgtgacaccacccaacccggt

tgtgagaacgtctgctatgacaaagcattccccatctcccacattagattctgggttctc

cagatcatttttgtgtccactccatccttactatacatggggcacgccatgcacaccatt

agggtggaggaaaagaaaaaattcagggaagaggaaatgaaagccaagcaggacatgagt

ggagactcaacctaccaccagaaagtgtacccaccagaaaaagtagaacttccctataag

gatgagattagtgggaaaattaggcttcaaggaagcttgttaaatacatacgtgtgtagc

attctcatccgcacagtgatggaggtagctttcatcgttggacaatatatgctgtatgga

atctttttggagaccctctatatatgcaagaggaaaccttgcccccatcccgtaaactgc

tatgtatccaggccaactgagaaaaacgttttcattgttttcatgttggctgtggcctca

ttctcacttctactcagcctggttgaactctactatttgtcatggaaggggctcaagcag

aaattgactaggccaccgccaagtggccaaaacgccttgagggtggccactatagagcca

gagcaccgctcccagagctgtacacctccgcccgattttaatcaatgtctaaacactagc

aaaggcaagtttgccaccccattcagcaatgctcttgcatctcaacaaaacacagctaac

tttgccaccgagagggtccagactgaagatgacatcggacaaggaccgttcgtccacatc

aactattcacaaaatgctccggtgaccaaccactctgcgccacaccacatgcccaatggc

tactgccacagcggccggcgcttgagcaagacaagccgcactagcagcaaagcccgctca

gatgatttggcagtgtga

>Np-gja8-XM_018557858

atgggagactggagcttcttggggaatattttggaggaggtgaatgagcactccactgtg

atcggtagagtttggctcactgtcctgtttattttccggatcctcattttgggaacagcg

gcagagtttgtgtggggggatgaacagtctgattttgtatgcaacacccagcagcctggt

tgcgagaatgtctgctacgatgaggcctttcctctgtcccacatcagattgtgggtcctg

cagatcatctttgtctccacaccatccctggtgtatgtggggcatgctgtgcaccacgtg

cggatggaggagaaaagaaaggagcgagaagaggcagaaatgagtaggcaacaggaaatc

aatgaagagaggctgccgctggctcctgatcaagggagcatccgaaccaccaaggaaaca

agtaccaagagtactaagaagtttcggctagagggaaccctcctaagaacctacatctgc

catatcatctttaagacactttttgaggtgggctttgtggtgggccagtattttctgtat

ggtttccggatcctccccctgtaccgttgcagccgttggccttgcccaaacacggtagac

tgttttgtctccagacctacagaaaagactgtgttcataatgttcatgctggctgtggcg

gcagtgtcactttttctaaatgtgatagagatcagccacctgggctggaaaaagatccgt

cttgcatttcgaaggtcggcggatcatcaaactcaacagctgctgggagaagtttcacag

aagcccctgcactccatagccatctcttccattccaaagtctaagggctacaagttgctg

gaggaagaaaaggctgtctcccatttctatccaatgactgaagttggattagaagctagc

cccctacctactcagtttaacaactatgacaaaagcagcacgggactcctggaagacctc

tccaaggcttatgatgaaactctgccctcctaccgccaagctgaagagcaagaggtggtg

aaagtgcaagtgactgagaccttagaacaaactccatctgagcgagccccatcagttagg

gctccttcggtgcgtgccctctctgaccgcgttccctctgagcgtgccctttctgaaaga

gcaccatcacgtgcagtttctgaccatgcagcttcagagcatgctccctctgaacatgca

tccatttcaagaatacaatccaaccatgcactctctgaaagagcaccctcagagcaagcc

atctcccgtgctggttctgagcatgccccttcggagcgagctcactctcgggcagtatgt

gagcttgcaccctcagagcgagctccttctcggaccatttctgaacacgccccttctgag

ggagctccatctcgagctacttccgagcacgcactctcaaaccgagctccctcacgggca

gcatcggagcatgctccatttgaacatgcgcctgagcaagtgccttctcaagcagcttct

aatgtccagttatatgatgctcctgtagaacagccccaagaaattgtcacgtcaccttta

gaagacgatcgtacaccctcaagagctctctcagaccacattggtcctgatccttcccca

tcagatcgtgaggttcctcaacctgtgcccccagaaccagacgtttgtgaaccagagcct

acaggcctggagttggctctattgagttttgaattagttccagattcccggtccctcagt

agattaagtaaagcaagcagcagggcccgatcagatgatttaatggtatga

>Np-gja9-XM_018560310

atgggagactggaatttccttggggtaatcctggaggaagtccacattcattctacaata

gtggggaagatctggcttaccatcctgtttatatttcgaatgctggtcttgggggtagca

gctgaagaagtctggaatgatgagcagtcacaatttgtttgcaacacagagcaacctgga

tgcaaaaatgtctgttatgatcaagcatttcctatatctttgatcagatattgggtcctg

caggtcatctttgtgtcttcaccttcattagtctacatgggccatgctatttatagactt

agagctcttgaaaaagaaagacacaagaagaaagcacagctaaaaggggaacttgaaaga

gtggagtatgagttgactgaggatcgtaagagactggaacgtgaactccggcagctagag

caaagaaaactcaacaaggctcctcttcgtgggtctctcctttgtacctatgttatacat

atattcactagatccgctgttgaggttggttttatggtcggtcagcatttgctttatggt

gttcaattagatccactttacaaatgtcagagggacccttgccctaatgtggttgactgc

tttgtatcaagacccacagaaaagacagtatttatgttatttatgcagagcattgcaggt

gtttctctttttcttaatattctggaaataggacatctgggtgtgaaaaagattaggaaa

ggaggtttagtgagatataaatttaaagatgaatttgatgacttttatgtaatgaaacct

aagaagcactccactgtaactcatacaggcatcgggacatcttctagtccacataagacc

cttccttcagcccctagtaactacacattgttaatggataaaccaaatgatccatctgtg

tatccaatattgaatccaccttgtggatttcatcctatagtagatatgcgtacagaaaac

aatagtacctgtatccttgatgaacaagaaaccaaatcagctaatgaagtcaaccctgca

aacatggcagacaaccacttccacaacagcagctccaacaataatgaaaagttcaccaag

acttatatttttgataacaagactccaaaaagccaaccgcaaaggaaaagtggctgtata

agaagctctcgatccagtgttgaaagtgcactgggtatttcaatgggaacaatgactgaa

ataccttcagaggacaccgtggattgctctgtacagaatgttatttgctctacaaccagt

aatgcagtccataagaccaggagaattagtgccccttggcacaattctaggatgggtgac

agatgtggttttgttcaagattctgtaagcagcagcaggggacgctacagtttcagcgca

aatagatcatgggcactctccaagtctgacctcaagcgcatcagtcggcctgctacacca

gaagccctaggggagcagagtccagagtacaagcaggataaggagtgtgatggttccgtg

accttctctccatctcgacggatgtcactggcaagtaacgccagtagccggcgtgtcccc

tccgatctacagatctaa

>Np-gja10-XM_018573519

atgggtgactggaacctgctgggaagcattctggaagaagttcatattcactccaccata

gtggggaagatctggctcaccatcctcttcatatttcggatgctggtgcttggagtagct

gctgaagatgtttgggatgatgaacaagaagaatttatttgcaacacagaacagcctggt

tgcaggaatgtctgctatgaccaagcattccctatctctcttattaggtattgggtgttg

cagataatttttgtatcttctccatccctagtttatatgggacatgcactttacagacta

agggccctggagaaagagagacagaggaaaaaggcccaaatcagggcggagctggaagag

ctagatacagttattgatgagcacaggagactggagaaggagctgagaaagttagaggaa

caaaagaaagttaacaaggctccccttagaggttcccttctacggacttatgtgctgcac

atcctaacgagatccattgtagaagttggatttatggtgggacagtactttctgtacgga

tttgaccttgatccactttacaaatgttcacgctttccatgccccaacatagtagactgt

tttgtatccaggccaaccgaaaaaaacatcttcatggttttcatgcacagcattgctgct

gtgtcattatttctaaatattttagaaatttttcatctaggtattaggaagatcaaacaa

gggctttatggaaaacatcttttagaggccacagaagatgaaataagcatttgcaagtcc

aaaaagagctctgtgcagcaggtttgcatgttgagtaactcttccccgaacaagatgata

cctttgtcttcaagtagttataaactgattcctgaccagcaattggactccattggatta

cccgcctatcttccaccagccccgggatttaaggaagtgcaaatgattaatgatcaaggt

cagtacggtaaggttatagctcctgaaaagcataagaggagtgtggatcatcttaacaca

gatcagcacagtggacagatcaacatgcacagtaaacaagaatctagatatgtggactgt

cagagaatgaacaatcacattgatcaataccctggccaaattctgaacaggactccgcct

aatcagcatcttgcagagcgcagggaagaacagacattgcagaagagaaatctctcatca

agcagtgaggactcgcataagaaatctcaacgtaatagctgtctggggtccaagagctgc

cttaaaagccatcagtctttagatcaagcaaggcacagtacagtacaccctcttagatca

tgcctccattccagccatatggagttaccatctgctttgcgcaaatacagcagagtcggt

agctgcaaagactttgcagaagatcgaagtgattctgcagacagtgggcaccggaaggtc

agctccacttccagaggattatcagaaagcagactcgctagtgatccagatagttctgac

tcaaggaatggctcaggatctgaatcaaggagacgagaggaaagctcgagcataacccct

cctcccccatctgggcgcagaatgtcaatggcaagtagaggcagacagtcagtggtttat

ccactctacagattattggtctag

>Np-gjb1-XM_018574721

atgaactgggccggattttacgccatcctgagcggcgtgaaccgccattccacgggcatc

ggtcgcatatggctgtccgtggtcttcatcttccgcatcatggtcctggtggtggcggcg

gagagcgtgtggggggacgagaagtcggcgttcgcctgcaacacccaacagccgggatgt

aacagcgtctgctacgaccacttcttccccatctcccacatccggctgtgggccctgcag

ctcatcatcgtgtccacgccggccctcctcgtggccatgcacgtggcccacctgcagcac

caggagaagaaaatcctgaagctgtccggccacgacgaccccaaggagctggcgcaggtg

aagcggcagaagatccgcatctcgggcacgctgtggtggacctacacgctgagcgtggtc

ttccgcatcatcttcgaggtggccttcatgtatctcttctaccacatctaccccggctac

gccatgatccggctggtcaagtgcggcgaatacccctgccccaacaccgtggactgcttc

gtgtcccgccccaccgagaaaaccatcttcaccgtcttcatgctggccacgtccggcatc

tgcatcgtcctcaacgtgtccgaactcttcttcctgatcgggaaggcgtgcgcccgccgc

aggctccgccacgaagactcctaccggggccacgagcatcggcagaacgagatgaacacc

ttgctgaccgtggacagcctcaaaaaggcgcccgggggccaagagaaaggagagcactgc

tccacgtcctag

>Np-gjb3-XM_018558031-gjb3like

atggattggaagacattccagggtctcttaagtggcgtgaataagtactcaacagctttt

ggacgtatttggctatcagttgtctttgtattccgagttcttgtgtatgtggttgcagca

gagaaagtatggggagatgagcaaaaagattttgattgcaacacccggcaaccaggctgc

actaatgtctgctatgaccactacttccccatctctcatattcgactttgggccctccag

ctgatatttgtgacttgcccttctcttctggtaataatgcatgttgcttatagagaagag

cgagaaaaaaagaaccgcgtgaaaaatggtgagaattgttctaagctgtactccaatact

ggcaagaaacatggaggtctctggtggacttatcttctgagtcttttcttcaaaactggc

atagagatcacctttctttacatcctgcataaaatgtgggacagctttgacatgcctcgg

ctcgtcaagtgtaccaatgtcgacccgtgccccaacattgtggattgttatatcgccagg

ccaactgagaagaaagttttcacatacttcatggtagcagcctctgcaatgtgcattgtc

atcagcatttgtgagatattttacctgatctttaaaagatttttaggctgttttaacaag

ttcaaaaagtccagtgatcactcggtaacctacagcaaagcttctacgtgccagtgccat

attcacttagataatacagagaagaagccacttaataagacagttgcatcgataagagct

tcagcaccaaatctaacagatttgtaa

>Np-gjb4-XM_018558030-gjb5like

atgaattggggtgtctttcaagccctcctgagtggagcaagtcatttctcaacaagcttt

ggacgcatttggctatccatagtctttatctttcgcttgctggtttatgtggtggcagct

gaaagagtttggggtgatgaacaaggagaatttgactgtaacacaaaacagcctggatgc

cacaatgtctgttatgatcattactttccagtgtcccacatcagactctgggccctccag

ctcattctggtcacctgtccatctcttcttgttatcatgcatgtggcctacagggaagat

cgagaaagaaaacacaaagaaaagcaaggagaggacaatggacggctgtatgcagacaca

ggaaagaagcgaggaggtttatggtggacatatgttatcagccttatagtcaaggcttcg

gtagatgtagtgtttctctacatctttcaccatatatatcataactattcattgccaaga

cttgtcaagtgtacattgaatccatgtcctaacactgtagactgctttatatctagacct

actgaaaaaaatattttcacattatttatggtggtcacaacaataatctgtattttgttg

aacttgttggagataggatacctagtgaccaagaggtgtcatgaatgcctcattcaaaag

catctcaagaatcagtcgactaacgtcattgcatatcttcagaagcaacagatgtttcat

gttaacaacaagctaaatgaagaacttgaccagctaaagccttgtattcatataatgcca

ccagaaactgaactgaaataa

>Np-gjb5-XM_018558022-gjb5like

atgaactggggtgtgtatgaagctctgctgacaggtgtcaataaattctccactgaattt

ggtagactttggctttccattgtttttatatttcgcatcttggtatatggactaactgca

aaccgcgtctggggagatgaccaaaaagactttgactgtaacactcggcagccagggtgt

tctaatgtctgctatgaccattactttcctgtgtcccacattcggctgtgggctttacag

ctcatcctggtcacctgcccttctcttcttgttgtgatgcatgttgcttatcgagagaac

cgagagaggaaatacagtgagagattgggggagaaatatgagaagctctacaaagacatc

agcaaaaagagagggggcctatggtggacctatctcttgagtcttctagtcaaagctgct

gtggacataacttttatttttatttttcatcgactctatgaaaatttttttcttcctcga

gtagtaaaatgcacacttagtccgtgtcccaacacagtagactgcttcatctccagacca

tctgaaaagaacatcttcactctatttatgatcattacttctgctgtgtgtgtccttctg

aactttgtggaagcttcatatcttgtagggaaaaaatgtagagagaccatgcaagccaga

aaaaagaaagttttgggagagacatgctctgatcactgtgtcagtgttcgtaatgaacaa

ccatgcacagataaaaattgtaagaccaagagtataatttcctaa

>Np-gjb7-XM_018554351

atgagctggtcttttttaagagacattttaagcggtgtcaacaaatactcaactggcatt

ggaaggatctggctgtcagttgtcttcatattccgacttctggtgtatgtggttgctgcg

gaaaacgtttggaaagatgagcaaaaggagtttgaatgtaacgtcagacagccggggtgt

gaaaatgtctgcttcgatcacttcttccccatctcacaagtcagactgtgggctctccag

ctaataatggtgtctacaccatcacttctcgtggtgctccacgtggcatatcgggaaagc

agagagaagaggcacaacaagaagctttacgaggacacaggaagcatggacggtggctta

tggtgcacatatgtcatcagcctcctatttaaaaccacgttcgagtttggtttccttatt

cttttctataagatatatggcgggtttagtgttccccggctggtgaagtgcgatatggag

ccttgccccaatcttgttgattgttacatctccaaacctacagagaaaatgattttcctg

tattttgtggtacttacttcaggactctgtattatactcaatatctctgagctggggttg

ctcatcttcaaatatttttcaaaatgctgtcggaaaagatatgagcaaaaactgacctca

ccacagtgtgactgtaggaatcgtcaccatatcaatgatcaggaaagtgcatctcactta

cagccaaagtcaaatgattctgagcaatag

>Np-gjb8-XM_08554680-gjb2

atggattggggaactctttatgaagtcattggaggggtcaacaaacattctacaagcatt

ggcaaaatctggctctctgtcctgttcatattccgtattatgattttggtggtggctgcg

gagagtgtctggggtgacgagcaatcagatttcacctgcaacacattacaacctgggtgc

aaaaatgtgtgctatgaccaccactttccagtgtctcacatcaggctctggtgtctccag

ctcatctttgtggctacaccagcactgttggttgccatgcacgtagcttacttgagacac

ggaaaaaaaagaaaacttcagaaaaatcgccagtcctccaagtttgatgacacggactta

gaggagttaagacagtatagatttcagatagtaggtacactctggtggacctacaccatc

agcatcctgttcagggtcctgttcgaggctactttcatgtatctcttctacttcctctac

actggtttccatatgcaacgtcttgtcaagtgcgacaactggccttgcccgaatgtggtt

gattgttttatttcaaggcctactgaaaagactgtatttaccatcttcatgattatcgtg

tctggcatttgcatggtgctaaatgtggcagagttgtgctatctcatcattcaggcatct

atgagaagagccaaaaagaatgccaagagatacaccaaccacataagtggcaaagaggag

tcacagaataagctaaatgagcagaaggaacaataa

>Np-gjc1-XM_018555183

atgagctggagcttcttgacacgactgttagaagaaatccacaatcactccacatttgtt

ggaaaaatctggctgacggttctcatagtcttccgaattgttctgactgtcgtcggaggg

gaatccatttattacgatgaacagtcaaagttcgagtgcaacacagatcagccaggctgc

gagaatgtgtgctacgatgccttcgcccccctttcccatgtacgtttttgggtatttcag

attatcttggtggccaccccaccacttttgtatttgggttatgcaattcacaaaatcgcc

aaaatggaggaacgctttgagtacgataagaaggcaaggttcagagccatgcccaagcgc

tggaagcagaatcgggctgtagaggaggtagaagaagaacatgaagaagaccccatgatg

tacccagggactgagcagggtagtgacagagggaaccacggccatgtcaagattaagcat

gatggtcgaaggcggattcgggctgatggattgttgaggatttatgtcctgcagctgatt

gtcaggacggtttttgaggtagggtttttggctggtcagtatttcctttatggctttgaa

gttatcaggagatacgagtgtgagaaagacccttgtccacacacagtggactgctttata

tcccgacctactgaaaagaccatcttcctgttaataatgtatggcgtcagctgcttgtgc

cttctcctgaacatatgggagatgcttcaccttggctttgggactatacgggacgcactt

aacaacagggggaagcaagtggacgattcttcttcctctacctatcctttctcatggaac

actccttcagctccacctggatacaacattgctgtgaaaccagaccaaatccattataca

gaactgaccaatgccaaaatggcttacaagcagaacagggccaacatagctcaggagcaa

caatacaggacaagtgaggacactctccccgctgacctagaacaccttcatcaggaagtg

cgcatggcacaagaacgcttgcaaactgccattcacgcttactcaactcagagcaaccca

ccttccaccaaagagaagcgatccaaaaaagggtctaacaagagcagcgtcagcagccgg

tccggagacggaaagacctctgtgtggatttaa

>Np-gjc2-XM_018558641

atgagctggagttttttaacccgtcttctagaagaaattcacaatcactccacctttgtg

gggaaggtctggctcacggtcctaatcatcttccgaatagtcctaaccgcagttggcggg

gagtcgatctactccgatgaacagagtaaattcatgtgcaacacgaagcagccgggctgc

gataacgtctgctatgacgcctttgcccctctgtcacacgtgcggttctgggtcttccag

atcatcatgatatcggctccgtcggtcatgtacctcggctacgccatccacaggattgct

cgggccgccgaggacgacaggcggaaccatatgcgaaagaagaagaaaaaggctttttcg

agatggcggacgggtgaaaacgttgaagacccggaggacgaggaggaggagcccatgatc

tacgagactccaacagaaccagagaaaacagaaagcaaaggcaaggaaaacaaaaaacac

gacggccggagacggatcaaagaagagggactgatgaaaatttatgtctttcagcttttg

tcgagggctgtttttgaagttggctttttgataggtcaatatttcttgtacggcttcgaa

gtcttgccttccttccagtgcaacacacagccgtgcccacacacggtagactgttttgtg

tctaggccgacagagaagacggtatttttgctgatcatgtacgtggtgagctgcctgtgc

ttgttgctgaacatctgcgagatgttccacctcggagttgggaccctaagggacgtgatt

cgcagccggaggactaatgccgccaggcagcccccttacaattactcctacccaaagaac

accgcttgccccccggaatacaatatggtggtgaagtcagacaagtcctccaaagtcccc

aacagcggcatggcccacgaacaaaacttggccaacgtcgctcaagaacagaactgcacg

agccccgacgacaacgttccgtcagacctcacctctttacataaacatttgagggttgcc

caggaacagttggacattgcattccagacctataactctcaggtcaacgcccaggcatcg

aggaccagtagtcctgcttctggggggacggtggtggagcagaatcgggtaaacactgca

cacgaaaagcaaggagcaaagcctaaggctggctcggacaaaggcagtaccagcagcaag

gatgggaaaacttccgtatggatttaa

>Np-gjc4-XM_018563038-gjc1like

atgagctggagctttctaacccgccttctcgaagagatcaacaaccactccacctttgtg

gggaaggtatggctcacgatcctgatcatttttcggatcgtgctgaccgctgtgggtggc

gagtccatctactatgatgagcagagcaagtttgtgtgcaacacgcatcagcccggttgc

gagaatgtgtgctacgatgcttttgcgcccttgtcacatgtccgattctgggtcttccag

atcatcctcatcacaaccccatccatcatgtacttgggcttcgccatgcaccgcatcgcc

cgccagccggaggattaccggaggcagctggaaagggctaaaagccagaagaaaagggca

ccggtggttcatcgtggggccaaacgggattatgaagaagcggaagatgacaacgaagag

gaccccatgatcagcgaggagctggtaaccgaaaaggacgttgaggataaggacaaaaag

aagcacgatgggcgaaagcgcattaagcaggacgggctgatgaaggtgtatgtgctgcag

ctgcttttccgctcagttcttgaagtcgggttcctctttgggcagtatattttgtacggc

tttgaagtcatgccatcgtacgtgtgcacaaggagcccttgtcctcatactgtggactgc

tttgtgtctcgtcccaccgagaagaccatcttccttctcatcatgtatgccgtcagctcc

ctgtgccttttgcttaacctgtgtgagctcttccacctgggcgttggtggcattcgcgat

tcactgcgacggagaagccgggatgccgatgagggccagtactctaaaaagacccccagt

gccccacctaactaccaatctgtgctcaaaaaagggaaggtgcccaacggtaagctgtat

ccagggaatggtgggtctgaagcctttgagatgccagtgacccatgagctggaccaactg

cgtcggcatctcaagctggcccaagaacaactggacttggcctttcaattgaacagcatt

gccggaaccgcccaacagtcacggagtagcagtccagaggctaacagcatggcagcagag

cagaacagactcaactttgcacaggagaagggtggaggagtctgtgaaaaatcaggtctg

tga

>Np-gjd1-XM_018555998-gjd2like The sequence in grey font is considered less likely to be a part of the proper RNA. The more likely correct exon 1 with 71 coding nucleotides is missing.

atggtacatacagatgctgtggagagcaaagagtgcttgatttgtggaacttcagtgttc

cgtacctccagttccagttttcctcacctaccgttccagtgttccttacctctggttcta

gtgttcctcacctccagttccagtattgctcacctccagttccagtattgctcacctcca

gttcaagtgttcctcacctccaattccagtgttcctcacctccgattccagtgttcctca

cctccaattccaatgttcctcacctctggaactggaggtgatagagttggcaagctcttg

ccttcaaacccagaccagcaactgactagaggtctagaaaaggtgaccatccgtgataat

agagatgaagctgtaatagtccactcgaaggaatactcattatgtgcctttctccttggc

ag

NNgattctgctgactgtggttgtcatttttcgaatattaattgtggctattgtcggagag

acagtctatgaagatgaacagaccatgttcatgtgcaacactcttcagcctggctgtaac

caagcctgctacgatcgagcattccctatctcccacatccggtactgggtcttccagatc

atcttagtgtgtaccccaagcctctgttttatcacatactctgtccaccagtcggccaag

cagagggaccggagatattccttcctctatccccttttagagaaggaactgccacgagag

aaccgaaggatgaaaaatatcaatgggattttgatgcagaaccaggacctttcgtccaag

gatgagccagactgtctggaggtaaaagagatcccaagcacgcctaaaaaagcagcaaag

agccccaaggtcaagcgacaagagggcatctcacgcttttacatcattcaggtcttcttc

aggaatgctttagagattggtttcctggcaggacagtacttcctttatggttttagcgtt

ccacccatctttgagtgtgaccgctacccttgcgtaaaggaagtcgagtgctacgtatct

cggcccactgagaagacagtcttcctggttttcatgtttgctgtaagtggcatctgtgtt

cttctcaacttagccgagcttaaccaccttggttggaggaaaatcaagacagccatgagg

ggagttcaggctcgcagaaagtcagtttgcgaggtgcgtaaaaaggagccatcaactctg

gctcaagtgccaacattggggaggacacaatccagtgagtcagcttatgtttga

>Np-gjd2-XM_018572752

atgggagaatggactatattagaaagacttctggaggctgctgtgcaacagcacagtact

atgataggaaggattctgctgaccgtggttgtcatctttagaatactcattgttgccatc

gtaggagagactgtttatgatgatgagcagaccatgtttgtctgcaatacactccagccg

gggtgcaaccaggcgtgctatgacagggcttttccaatctctcacatcagatactgggtg

ttccagatcattatggtctgtactccaagcctttgctttatcacctattccgtgcaccag

tctgctaaacagaaggaaaggaggtattccactgtgtttctcaccttggacagggaccaa

gacagtatgaaaagagaggacagcaaaaaaatcaagaacacactggttaatggtgtcctt

caaaacaccgaaaactctaccaaggaggctgaaccggactgcttggagatcaaagaaatt

cccaacccctccataagaaccactaaatctaaaatgaggcgccaggaaggaatctctagg

ttttatattattcaagtggtcttcagaaatgccctggagattgggtttcttgttggacaa

tactttctgtatgggttcaatgtaccctctcagtatgagtgtgacaggtatccgtgcatc

aaagaggtggagtgttatgtatccagacccacagaaaagacagtgtttcttgtttttatg

tttgcagtcagtggtctctgtgttgttcttaacttggctgagctaaaccacttaggctgg

aggaaaatcaagacggcagttagaggtgtacaggccaagaggaaatccatttatgaaatc

agaaataaagacttgcccaggatgggagtacccaactttggaaggactcaatctagtgac

tctgcctatgtgtga

>Np-gjd3-XM_018566425

atgggggaatggggctttctgagctcgttgttagatgcggtccaggagcactctcccatg

gtgggaaggttctggctggtggtgatgctgatctttcgcatactcattctggccaccgtg

ggcagtgacatgtttgaagatgaacaggaggaatttgtatgtaacactatgcaaccaggc

tgccgtcaggtttgctatgaccaggccttcccaatatcccactaccgcttctgggtcttt

catattgtgctgctctctgccccagcagtattgtttgtcatttattccatgcaccagaat

gccaagatgggaagagatgcagatgaagaggaggcagcacaacatgggcagccgggcagg

accaggtccaagttaagggctgagcagcgtggacgtcacatacgcaccttctacatcgtc

aatgtggtgatgaggataatggcggaggtgggttttttagtgggtcagtggctcctctat

gggttcaaagtagctccagactacctctgtgtacgtgaaccctgcccacataaagtggac

tgctttgtgtccaggccaacagaaaagaccattttcctgcagttttactttgtggttggg

atcatctctgcgattctcagcctgtctgaactgctacacattctggtgaagggaaagtgc

cggtctagggaaggactgggatcaagtccacctccagcctacgagagggataattggtcc

aacagagaacatgaaaggaccaataactttcttcttaaaagacaaagtcctgatgatcgg

agagctagcggggctggtggtcatcgactttctattgcatatcctcctggcacggcctat

aaactaaaggtagcccctagctcagctatcagtagcaagtcatctagactgatcaaagct

gacttaactgtataa

>Np-gjd4-XM_018552320

atggaaatttttgactcgctgggatatgtgatcatcactttcagctataatgttactgta

accgggaagatctggttgacgttaatgatcctcctcagactagctatggttgtccttgct

ggttacccgctctaccaggatgaacaggaacgctttatctgcaatacattgcagcctggg

tgctccaatgtgtgttatgatatcttctccccaatgtcccacatgcgatactggctgatt

cagactctgatgatctttataccctacgccttgtatagcgtccatgtcatacacaaagtt

ctggcttatatggccaatgcaaatgaccattgcagaaaaagtacctcctccacttatcat

ggagtgggcttacaaatggaaatacttgatttttcttgggcttatcttgttcatgtcctg

ctaaggatttgtctagaggttggctttggtgttggtcagtattttctttatggaattttt

gttccaagcagattcagctgttcgcagtctccctgcaccagcaacgttgactgctacatc

tcaagacccactgaaaagtcgcttgtgatgatctttgtctggggaactggaatcatctct

ctgattctgagcttgatggatctaactggagtctgtcaccgaagacgaagatcagagaag

cttaaacgacagcttcttctcctggaaaatgattctctaaaaggggcttgttgctcagac

actcctccacatacaaagttagctgtcacaccagatcagatgactatgtatcctgactgt

ccttcacacagcctagagaaaaaggaggaacaaattaaaggtgattctcattctctgcca

tcatttgaaggtgaaacagcttctttccaaactgacaattctcaccaggacatgggaaag

tccaatgtaaattccaacagcaacaaaacctgttcgtggcaagtaactatgtctggaaat

aacacactagggaatgaacatcaactgctcacccaccggcaaccaaagtccaaaaaggag

atttgtactgcagctagtatggcggattcacctcaactagaaaaaaatgttagttcaaaa

aacaaaaaatcggagtgggtctaa

>Np-gjd5-XM_018563428-gjd2like

atgggagactggtctatcttgggacgtctactaacagaggtccaaaaccattcaactgta

attggaaaaatatggctgacaatgctcctgatattccgaatcctcttggtaacactggtg

ggagatgccatgtatggtgatgaacagtctaaattcacttgcaatacccttcaaccgggc

tgcaataatgtctgttatgatagttttgcaccggtctcacatcttagattttggatcttt

cagattgtcctagtatcaaccccttccattttttacatcatctatgtcctgcacaagatt

gctaaagatgagaagactgaactagaaagggcctacatcatggaacttcttcaaagttta

tccattggcgaacatttcctgaaaaagagcaaaagtccagatgctgatgagcaggtggag

gttaaagagataaccaaaaaaggagagaggttcaagccaaagtttgcgaatgatcctgtc

cggggtcccattccggtgtttgacaagatgcacgtaatttacattgtccacgtggtgctc

aggtcgatcatggaagtggtgttcctagttggtcaatactacctttacggctttgaagta

cctcatcttttccagtgtcacacctacccttgtcccaccacaacagactgttttgtctcc

agagctactgagaagacagtctttcttaactttatgtttggagttggtcttggttgcttt

attttgaacattgtggagctgcattacctggggtggttctatgtatttagaattttagca

gttgcttgcaatacttgttgtgaattcaagagcagaaagaaaaagaggcccatggtattg

catcctgagcagaatagtctacttatacatctgaaggacaccatacaagaacgatttgtc

ctgaagacctcagcaagcctttccacagagaaaattggctgtttaccttccccaccagta

aacacgatttccttcacaaatgatgacactgatactaccgcaaggaggagcccagatatt

aaacacagtaatataggcaaaatagccaagggcaagaaatcatggctttag

>Np-gje1-XM_018565608-gje1like

atgtctttaaattatatcaagaacttctatgaaggatgtctcagacctccaacggtgatt

ggtcagttccatacccttttctttggttctgtacgaatgtttttccttggtgtcctgggt

tttgctgtttacggaaatgaggccttacatttcagctgtgaacctgacaaaagggaggtg

aacctcttctgttataaccagtttcgaccgataactcctcaggtattttgggcacttcag

ctggtgacagtactagttccaggagcggttttccatctctacgcagcctgtaagagtatt

gaccaagaagacattctccagaagcccgtttatacagtcttctacattatctcagtactg

ttgcgaattacattagaagtggtggccttttggctacaatctcatctttttggcttccaa

gtgaatccaatctttaaatgtgatgcaggaagcctagataaaaaatacaatgtcaccaga

tgcatggttcctgaacactttgagaaaacaatcttcctcattgctatgtatacttttact

gtgattacgatagtactatgtgtagctgaaatctttgagattctatgcaggcgtttagga

tttttaaacactcagtga

# Supplementary Figure S26. Coelacanth (*Latimeria chalumnae*) connexins.

The chronology of the sequences is according to the Greek nomenclature. Size nomenclature is indicated in parentheses. GenBank accession number is given for each entry.

>Lc-gja1-XM_006005808-GJA1-43

ATGGGTGATTGGAGTGCACTAGGAAAACTTCTTGATAAAGTTCAGGCATATTCTACTGCA GGAGGCAAGGTATGGTTGTCTGTCCTCTTCATCTTCCGAATCCTCATTTTGGGAACAGCA GTTGAGTCAGCCTGGGGGGATGAACAGTCTGCATTCACTTGTAATACCCAACAACCTGGT TGTGAGAATGTCTGTTATGACAAGTCCTTCCCCATTTCCCATGTTCGCTTTTGGGTTCTG CAGATCATATTTGTCTCAACACCCACCCTCCTGTACCTGGCCCATGTGTTCTACATAATG AGGAAAGAAGAAAAACTCAGCAAGAAAGAAGAAGAGCTTAAAATAATTCAAAAGGACCAT GGTGATGTGGACCTTCACCTGAAGCAAATTGAGTTAAAGAAGTTTAAATATGGAATTGAA GAACATGGAAAGGTTAAAATGAGAGGAGGGCTGCTTCGTACTTATATCACAACCATCATT TTCAAAACTGTTTTTGAGATTGCATTTTTGCTGATTCAGTACTATATTTATGGTTTCAAA CTGGAGGCTGCCTATGTTTGTGAGAGACCCCCATGTCCACACAAAATCGACTGCTTCCTC TCTCGTCCCACAGAAAAAACAATTTTTATCATCTTCATGTTGGTGGTGTCTGTAGTATCA CTTTTGTTGAATATTATAGAGTTGTTCTATGTCTTTCTTAAAAGTGTAAAGGATCGTATG AAAGGGAAAAATGATCCATTCTCTACCATAGGTGGTGGCTTGAGCCCAACTCCAAAAGAC TGTGCCTCTCCAAAATATGCATTTTATAATGGCTGTTCCTCTCCCACTGCCCCACTGTCC CCCATGTCCCCTCCAGGCTACAAGCTTGCTACTGGAGATAGGAACAACTCTTCTTGTCGT AACTATAACAAGCAAGCCAATGAGCAAAATTGGGCAAATTATAGTGCTGAACAAAACCGA ATGGGGCAGGCTGGTAGCACCATATCCAACTCTCATGCTCAAGCATTTGACTACCCTGAT GACCACCAGGACACAAAAAAGGTGGCATCTGGACTTGAATTGCAACCTCTCACCCTCATG GACCAAAGGCCACCCAGCAGAGCCAGTAGTATAGCCAGCAGTAGACATAGACAGGATGAC

CTTGAAATCTAG

>Lc-gja2-XM_006005447-gja3like-39.9

ATGGGAGACTGGACTTCATTGGGGAAGCTACTAGAGAACGCACAGGAGCATTCCACAGTT GTAGGCAAGGTGTGGCTCACCGTCCTCTTCATCTTCCGGATCCTGGTCCTGGGCACGGCT GCCGAGAAGGTGTGGGGTGATGAGCAGTCTGGATTCACTTGTGACACCAAGCAGCCCGGT TGCCAGAATGTTTGTTATGATTGGACCTTCCCCATCTCCCACATCCGCTTTTGGGTTCTT CAGATCATCTTTGTCTCCACACCCACCCTCATCTACCTGGGCCATATTCTCCATATCATC AGGAAAGAGGAGAAAGAGAAGCAGAGAGAAAAAGAGGGGCAGGAGAAAAGTGGGCACCAC GGGGACAAACAGCCCCTCCTAGGAGAACCCAAGAACCCTAAGGTCTCCATCCGAGATGAT CAGGGCAAAATCCGGATGAGGGGGGCTATCTTGAGGACTTATGTCTTCAACATTATCTTC AAGACTCTGTTTGAAGTGGGCTTCATTGTGGCCCAGTACTTCTTGTATGGTTTCGAGCTA AAGCCCCTCTATACTTGTAGTCGATGGCCATGCCCCAACACGGTCAACTGCTACATCTCC CGTCCAACAGAGAAGACTATCTTCATCTTGTTCATGTTGGCAGTGGCCTGCCTCTCCTTG CTCCTAAACCTCATTGAGATGTATTATCTGGGCTTTACCAGGTGTTGTCAGGGACTCACC AACCGGTACGGCTCAGAGACGGGTTCCAAGCCCTGTTCAGAAGCTCCCAGTGAAAACGCT GTGCCTTTTGTCCAGCATTACCCTTTCTTCCCTAGTCATGGCAGGCCGCAGCCATATAAC CCCAGCCTAGCGGCATACCCCTTGCCTTCCATGAATGAGAACAATTCCTCCTTTCACCCT TACAACAGCAAGGCCGCCCACAAACAGAACCGGGACAACCTGGCTGTGGAGAGGAACGGG CGCTCATCTGACAGCAATCACAGCCCAAAGGAGGGCAAAGCTGCGCCATCATCGCCCTCA GACAACCAACGGAGCAGCAGCCGGTCCACCAAACACGGGAACAAAGCCAAACCGGAGGAT

CTGGCAATTTAA

>Lc-gja3-XM_006004018-GJA3-46

ATGGGTGACTGGAGTTTTCTGGGGAGGCTTTTAGAAAATGCACAAGAGCACTCCACAGTA ATAGGCAAAGTCTGGCTGACAGTGCTATTCATCTTCAGGATTCTGGTATTAGGGGCAGCA GCAGAAGAAGTGTGGGGAGATGAGCAGTCTGACTTTACCTGCAATACCCAGCAGCCCGGT TGTGAAAACGTATGCTATGATAAGGCTTTTCCCATCTCCCATATTCGCTTCTGGGTGCTT CAGATCATTTTTGTCTCCACACCCACCCTTATCTATCTGGGTCATGTGCTGCATATAGTG CGAATGGAAGAAAAGAGAAAAGAGAAAGAAGAACTAGCGAAATGCAATAAAAGCCAAGAG GACAAAGAACTCCTATTGAAAAAAGGTGGTGGGAAAAAACAGAAACTACCAATAAGAGAT GAGCGTGGTAAAATAAGAATTAGCGGTGCACTGCTGCGCACGTATGTCTTCAATATTATT TTCAAGACATTGTTTGAAGTTGGATTTATTGTAGGTCAATACTTTCTCTATGGTTTTGAG CTGAAGCCTCTTTACAGGTGCAACAGGTGGCCTTGCCCGAACTCTGTGGACTGTTTTATC TCAAGGCCCACCGAAAAGACTATTTTCATTATATTTATGCTTGTGGTGGCTTGCGTGTCT CTTTTGCTTAATATGTTAGAGATTTACCACTTGGGATGGAAGAAGCTCAAGCAGGGGGTG ACCAATGAATTCACCCCAGACCCTGTACTTGCTTGTAACAAAATGGAGTCACAGTCCTCA ACCCCAGCTCCCAGGACTGCTCCTCCTAGCCTTCACTATCCCCCCTTTTATGCCGAGGCT ACTTCAGGAGGGCACCATGGCTACCCCGGGGCCCCTGCTGCTGACTTTAAGGCTATGCCT CCCCCAGCAGAGGAGCCTCACAAGCCGTCGTATTTCATCAATAGCAACAACCACAGGCTT ACCACGGACCAGAACTGGGCCAACCTAGCGGCTGAGCAGCAGACGCAGGAGAGGAAGCCG GCCTCGAGCTCCTCCACGAACAGCAGCGTCGGGAATGAAGGTGCACAGGAAGCGCCAACT CCCGGCAGCAGCGGCAGTCTAGGCGGCGGGAAGCAGGGACAGGAAGACGGGAACGTGACC ACAGTGGAGATGCACGAGCCTCCAGTTCCCACAGACACGCGACGATTGAGCAGGGCCAGT AAGGCCAGCAGTAGTAGAGCCAGGCCGGATGACCTGGCAGTCTAG

>Lc-gja4-XM_005988525-GJA4-37

ATGGGAGACTGGGGATTTCTAGAGAAGCTGCTGGATAAGGTCCAAGAGCACTCCACCCCC ATTGGTAAGGTTTGGTTGACAATCTTGTTCATCTTTCGAATTCTGATCTTGGGAGTAGCA GCAGAGTCTGTGTGGGGAGATGAGCAGTCAGATTTCACCTGTAACACTGAGCAACCTGGC TGTGAAAATGTATGCTATGATAAAGCTTTCCCCATCTCTCACATTCGCTACTGGATGCTG CAAGTCCTCTTTGTTTCCACACCAACCATTGCCTTCCTTGGCCATGTAGCACACATCACA AAAAAGGAAGAAAAACGAAAGCAGGAGGAGAAAGAACTTATGGAACTAGAATCAAAAGAC TTTCAAGTGGAGCAGTATCTGCAAAAATTAAAACACAAAATCTCTAAAAATGTTATTCAA GAAGATGGTAAACTAAAAATTAAAGGCTCCCTGATGTTCTCTTATATAATAATTACTTTT CTGAAGGCAATCATAGAAGCTGCCAGTTTGTTTGGTCAGTGGTGTTTATATGGATTCTAT ATGCATCCTATTTATGTATGTAAGAGAGTCCCATGCCCACACCAAGTGGACTGCTTTATA TCTCGTCCCACGGAAAAGACCATCTTCATCATTTTTATGCTAGTTGTAACACTGGTGTCC TTCTTTCTGAACCTGGTAGAACTGTGTTACCTATTGTGTCAGTCTGTTATCAGAAAGGTT AAAAAATCCTCCTTGGCTTTGAAACCAAGAGATTTGCTGAAGAAGCCATGGGAAGGCCCA AATGAGCCTTTTGACAAGCCATATTTCTACCTACCCGTGGAAGGCAACCATCAGTCATAT CAATGCTTCAAAATGTCAAATGAGCAAAATATGACCAACTTTAGCATGCAACGCAAACTA GCAGAACAAGGTGGTAATGGCCCAGACACAGACCACATTTCACAAGTAGCCTCAAGAAGT GATATGGATGCTACTTCTCAGTTAGTGCCTGACCAAAGGCCTCCTAGTAGAGTAGATAGT GAGGGCTCAAAAAAACAATACGTGTAA

>Lc-gja5-XM_006011592

ATGGGTGACTGGAGTCTGTTGGGTAATATCCTAGAAGAAGTGCAGGAACACTCCACTGTT GTTGGAAAGGTTTGGCTCACAGTCCTCTTCGTCTTCCGAATTCTGGTCTTGGGTACTGCA GCAGAATCTTCTTGGGGGGATGAACAGTCAGACTTTCTTTGTGACTCTCTGACACCTGGT TGTGGCAACGTTTGTTATGACGATGCCTTCCCTATCTCTCATATCAGGTACTGGGTGCTT CAGATTATTTTTGTGTCAACCCCTTCCCTTATTTACATAGCACATGTCATGCATACAGTC CATATGGAGGAGAAAAGAAGTTTGAAGGAGCAAGAAGAGAGAGCCCGAAGTGAAGGCAAC AGGGAAGCTCCCTTCCTCGAAAAACAGCCTTTGGAAGAGAAAGTGGATATTCTTCCTGTG AAAGATGAAGTAAGTGGCAAGATTAGACTCCATGGATCCTTGCTTCACACCTACATGCTG AGCATCCTCATTAGAACTCTCTTTGAAGTGGGCTTCATAGTAGGCCAGTATATGATATAC GGGATACTCCTTCATCCCTTGTACGTCTGTGGTATGCCACCTTGCACTAACAAAGTCAAT TGCTACATGTCAAGGCCAACCGAGAAGAATGTGTTCATTGTCTTCATGCTTGCAGTTTCT GCACTGTCGCTCCTTCTCAGTTTCATTGAACTCAACCATCTGATATTGAAGAAAATGAAG GAAGAGTTGTTCAACAGGTATAGATCCCAATCCAGTGCAATCACTGAAGGCCTACTGTCC AAAGAGCCCAAACCCCTGCAGGGACCCTCTGGGAGCTACACCCCTCCCCCTGATTTCAAT CATTGTGTAAACAGCCCCAATGGAAAGTATAACCCATTCAACAACAAGCTTGCATCCCAG CAGAACTCTACCAACTTTGCCACAGAACAGGACCAGAGTCAAGAGGAGCTAGGAAGTGGA CAATTCATTCATGTGAACTATGAACATAACAAAGAGGCTTCCAACAGTTCTGTTCCCCCT CTTCATCTGTCACATGGGTTTAACAAAGACAACCGTCGTCTGAGCAAGGCTAGCAAAAGC AGCAGTAAAGGAAGGTCTGATGACCTGGCGATATAA

>Lc-gja8-XM_006011594

ATGGGTGATTGGAGCTTTCTTGGGAATATCTTGGAGGAGGTGAATGAACACTCCACTGTG ATTGGACGAGTTTGGCTGACTGTGCTCTTCATATTCCGGATTCTGATTCTGGGCACCGCT GCCGAGTTTGTGTGGGGTGATGAGCAGTCCGACTTTGTTTGTAACACCCAGCAGCCCGGT TGTGAGAATGTCTGCTATGATGAGGCTTTCCCCCTCTCCCACATCAGGCTGTGGGTCCTG CAAATCATTTTCGTGTCCACTCCGTCACTGGTGTATGTGGGCCATGCTGTCCACCATGTC AGGATGGAGGAAAAGAGGAAGGAGAGAGAAGAGGCTGAGATAAGCAGGCAGCAGGAACTG AATGAGGAAAGGCTGCCTCTAGCTCCTGATCAGGGAAGCGTGAGGACAACCAAGGACACC AGCACCAAAGCCACCAAGAAGTTCAGGCTTGAAGGAACCTTGCTGAGAACTTACATCTGC CACATCGTCTTCAAGACCCTCTTTGAGGTGGGCTTCGTGGTGGGTCAGTACTTCCTGTAT GGCTTCCACATCCTCCCACTCTACAGGTGTGGCCGGTGGCCCTGCCCCAACATTGTGGAC TGCTTTGTCTCCCGGCCCACTGAGAAGACTGTCTTCATCATGTTTATGTTGGCCGTGGCC TGTGTATCTCTGTTCTTGAATTTTGTGGAGATCAGTCATCTAGGCTGGAAGAAGATCCGC TTTGCCTTCAAGACACCAATGCAGCAGCAGCAAGGAGAGCTGTCCACGGAGAAGCCCTTA CATTCCATTGCTGTGTCCTCCATACAGAAGGCCAAAGGCTACAAGCTCTTGGAAGAAGAC AAACCTGTCTCCCATTTCTACCCTCTCACTGAAGTCGGGGTAGAAACCACCCAGCTCCCA GCTCCCTTTGCAGGTTTTGAAGAGAAGGAGAATGCCACAGGGCCTCTTGAAGACATCTCC AAGGTTTATGATGAAACACTGCCATCTTACGGGCAGATGACAGAGCAAGAGGAAGAGATT CCAGACCCTCAGGCTGAGCCCACAGAGATTGCCGCCGCTGATACCAGACCCCTCAGCAGG TTAAGTAAAGCCAGCAGCAGGGCTAGGTCAGATGATTTAACTGTATGA

>Lc-gja9-XM_005988776-GJA9-59

ATGGGAGACTGGAACTTCCTTGGAGGTGTCTTGGAGGAGGTCCATATCCACTCCACCATT GTTGGGAAGATCTGGCTGACTATCCTGTTTATATTCCGCATGCTGGTCCTTGGAGTGGCA GCTGAAGATGTCTGGAATGATGAGCAGTCAGAATTCATCTGCAACACAGAGCAGCCAGGC TGCAGGAACGTCTGCTATGACCAAGCCTTCCCCATCTCTCTTATAAGATACTGGGTCTTG CAGGTCATCTTTGTGTCCTCTCCATCATTGGTCTACATGGGCCATGCCTTGTACAGGCTG AGGGCTCTGGAGAAAGAGAGACAGAGGAAGAAGGCACAGTTAAGAGGGGAGCTTGAAGTG GTAGAAATTGAGATGACTGAAGACCGAAGAAGGCTGGAAAGAGAACTCAGACAACTGGAG CAGAGGAAGCTGAACAAAGCACCACTAAGAGGCTCCCTTCTCTGCACCTATGTACTCCAC ATTCTTACCAGATCTGCTGTGGAAGTTGGGTTTATGGTTGGCCAATATCTCCTTTATGGA TTTCAGCTGGATCCTCTTTATAAGTGCGAGAGGGAACCATGCCCTAATGTAGTTGACTGC TTTGTATCAAGGCCAACTGAAAAGACTGTGTTCATACTTTTTATGCAGTGCATAGCAGCA GTCTCTTTATTTCTAAACATTCTGGAGATCATCCACTTGGGCTTCAAGAAGATTGAAAAG GGACTTTTCCAACAGAATCCTAAGATAAAAGATGAATTTGATGATGTTTATGTCAGCAAG TCAAAGAAGAACTCAGTGGTTCAGCAAGCTTGCACAGGCACAGTCACAACCCCACAGAAA TCTATTCCCTCTGCACCAAGTGGCTACACTTTGCTGATGGAGAAGCAAGGAGATTCAGGG GTTTACCCTGTTTTGAATCCCGTGTCTGCATTCAAACCAGTCCCAGATAGTTCTAAAGAA ACTGGCAAAAGTTGCACTGAGGGACAACAGGAAAGCAAAGCGTTTAATCCAGTCCAAACT AAACCCACAGGGGAAAATGGCTTTCAGAACACCAGCTCTAGTGTTAATGAAGAGACCAAC AGACAGACTGGCGCAGAGATGAATGGTGTACAGAAGCGTGAAAACCATCTTGAGCGTTCC CAGTTTGCAAGACGCTCTGGGATACTTACTACTACTGCACAGAATTCAGGAAGCCAAGCA GAAATATTTTCTGGGACACCATTATACCCTTTATTACAAGCAGATATGAGTTCATCCTCC ATGCCACAGCAAGCCAGCATGGGTCGAAAACCACGCCGAATTAGTGCTCCATGGAACTGC TCTACGGTGGTTGAAAGTGGGGGATCTCCAGTACGATCTCCTGCTGCAGGCAGTGCCAAG GGCCGATGCAGCTTCAGCGCTAGCCGGGTCAGAGCAGTTTCCAAGTCTGACCTCAAACAC CCCAGCAGGCCAGACACCCCTGACTCAGCGGATGGGTCCAGCTCAGAGTCAAAGCACAGC AAGAATTCTGACACTCCACAGACTGTGTCTCCCTCTCGGCGAGTCTCATTGGCAAGTAAT GCCAGCAGCAGGCGTGCCCCCACTGATCTTCAGATCTGA

>Lc-gja10-XM_014484226-gja10like

ATGGGAGATTGGAACTTGTTGGGAAGCATCTTAGAAGAAGTCCACATCCATTCTACCATA GTAGGAAAGATCTGGCTAACCATACTGTTTATATTTCGCATGCTTGTGCTTGGAGTGGCT ACTGAAGATGTTTGGGATGATGAACAAGCAGAGTTCATCTGCAACACAGAACAGCCTGGT TGCAGGAATGTATGTTACGATAAAGCTTTCCCTATTTCTTTAATCAGATACTGGGTCCTG CAAACTATCTTTGTATCTTCACCGTCCCTGGTTTACATGGGCCATGCACTTTACAGGCTC AGGGCACTAGAGAAAGAAAGACAGAAGAAGAAGTCCCAGTTAAGGGCTGAACTGGAAGAA ATGGAGCCCTTGCTGGAGGAACACAAAAAACTGGAGAAAGAACTGAGAAAGTTGGAAGAG CAAAAGAAAGTCAACAAAGCTCCCCTAAGAGGATCTCTTTTACGCACTTATGTTGTGCAT ATCTTAACCAGATCTGTGGTGGAAGTAGGTTTCATGGTAGGCCAATATGTTTTGTACGGA TTTCAGCTGAGTCCTCTTTATAAATGCTCACGGTTACCCTGTCCAAATATAGTGGACTGC TTCGTCTCCAGACCCACAGAAAAGACTATTTTCATGGTCTTTATGCTGATTATAGCAGCC TTTTCCTTGTTTTTGAACATCTTGGAAATTTTTCATTTGGGAATCAAGAAGATAAGGCAA GGTCTTTATGGAAGACCAAGAAGAAGCGCCACTCAAGAAGATATAAGCATTTGTAAAGTT AAGAAACACTCAGTGCAGCAAGTATGCATTATGAACAGCTCCTCTCCCCACAAGCTTGTC CCCTTAACACAAAGTGGTTACAAAATTCTGCCCGATCAGCAAGCGGACTCTTTTAATCTT CCCGCTTACATGCCTCCTTCAGCCGAGTTCAAGGTGATCCAGGAGCAAAGCAGCCCAAAG GACAAGGCTAAAGAGCCGAGTGGCAACCAGGACCAAGGGCTGAGCCACGAAGATTACCAC CGAAGTGGCCCTCTGCAGAGCCGCGAGGATCATAGACCCAGCGGCCAGAACCACGGCCAG CTCCGCGACCGCCCCTTCCACCAGAGGCGAGCGCCGCCGCCGTGCGAGCTGCTGCTCAGG GATCAGCACCACCGACAGCATCCTGATCGCGGCCAGGGTCAGGACGCTGGACAGCGCCGC GGGCCGTCTAGCCACAGCCAGGACACCCGGGAGCACTCCTTCAGCAGCGACGACTTGCAC AGCAAAGCCCCTCCCAACGGGCTCGGGCCCAAAGGCTGCCAGAAGAGCGAAAACGCCCTG GACCAGCACAGGCACGCCGGCTACCCGGCGAAGGCTCCCCTCCACGCCAGCCAGCCCGAG ATCCCCGCCGCCCTGCGCAACGCACTCCGCAAGCAGAGCCGGGTGAGCGGCGGCAGGGAC TACCCCGAGGACCGCAGCGAGTCCCCGGACAGCTCGCACTACCAGGGCACCCGGAAGTCC AGCTTCATGTCGAGGGTGATGACGGAAAGCAGCCGATCGCCCAGCCCGGACTCGCGGAAC GGCTCTGGCTCCGAGTCCAAGCGGAAAGTGGAGAGTCCTCCGATCACCCCTCCTCCGCTG TCAGGGCGCAGGATGTCCATGGCAAGTAGCATTATAAGAGAAGCCCACTCTCCTCTGGGA

AAACAGATCAGATAG

>Lc-gja11-XM_014494418-32.2like

ATGGGAGCCTGGATATTTTGGGGAAGACTGTTGGATAAAATAGAGTCCCATTCCACAGTG GTTGGAAACATATGGTTGACGGTGCTGTTTGTGTTCAGGATCTTAGTGCTGGGAGCTGTT GAAAGAGTTTGGGGAGATGAGCAATCAGTCTTCATTTGCAATACTCACCAGCCTGGCTGT GAAAATGTCTGCTATAACAAAGCTTTCTCCATCTCCCACATGCGCTTCTGGGTGGTGCAG ACCATCTTTGTCTACACACCAGCTCTATTGTATCTAGGGCATGTTGTGTATGTGATTCAC CAGGAGGTGAAACTCCAAGAACTGCAGAACCAGGAACATGAAGAACCATTTAAAAGATCT CCAAAACACACAACAGAAAGTGGAAAAGCCAAAATTCAAGGAAGTCAGCTAAGGATCTAC TACTTGTTGAATGTTGCATGTAAAATTCTTTTCGAGACTGCATTCATTGTCGGTCAGTGG TATCTGTATGGCTTCACATTAGAACCACAGTATATATGTTATCAACATCCATGTCCTCAC AGTGTAGATTGTTATGTTTCTCGTTCAACTGAAAAGACCATTTTTATCCTCTTTATGCTA GTGGTGGCATGCGTTTCACTGGCTCTAAATCTAATTGAATTTTGCATGTGTCTTTGCTGT AAAGGTATTAAACAGACCACAGGAAAGAACTACCAACATTATCTTAGGGATTCAGTGAAT ACCATTGATGCGTGCAATAAAAAAGACCCAAGTCATTACAATACAGCACGTCCTAGTACA GAACAGAATGAGACCAGTGGAGCTCACCTTCAACATAACAGGGAACAGAGAAAACAGTAA

>Lc-gja14-XM_006009865-gja5like

ATGGGTGATTGGTCATTTCTGTCCAGGATACTGGAGCAGGTCCATGAGCACTCTACTTTG GTGGGCAAAGTCTGGTTGACAGTCCTTTTCATTTTCCGGATGCTGGTGCTGGGGACCGCA GTGGAGTCGGTCTGGGGGGATGAGCAGTCGGGCTTCACCTGCAACACCCAGCAACCAGGC TGCGAGAGCATGTGCTATGATCAGACCTTCCCCATCTCCCACATCCGTTTCTGGGTCCTT CAGATCATTTTTGTCTCCACTCCTTCTCTGGTCTACATGGGCCATGCTGTTTACATCATG AAGCTGGAGGAGAAGAGAAAGCTTATGGAGAAAGAACTCAAGGTTTTGACCCACAAGGGG GTGATGCCACTTCTTGAAGACAAGGAGAGCAGACCCAACTCAGAGCAAAGTTCTACCAAG GAGGAGCATAGAGGACAGTTTAAGATCAAAGGGTCTTTACTTCAGACCTATGTGTGCAGC ATCATCATCAGAACACTCTTTGAGGTTGGCTTCATTATTGGCCAATATTTTATCTACGGT ATTTTCCTCAAGAGGATGTACAAGTGCGAACGGTGGCCCTGCCCCAACATCATTGATTGC TTTGTCTCCCGGCCAACAGAAAAGAATATTTTTATTGTCTTCATGCTTACTGTGGCCAGC ATCTCCTTGTTTTTGAACCTAGTGGAGATGTACCACTTGGGTTGGAAGAAGTTCAGGGAA GGGTTGACCAATACCTTCTACCAGGAAGGCGATGCTGTTCTCCAGGACACACCCAAATCA TTGACAAAGATTGAGTATGTGGCCCTGGCTGGCTGCAAGACCCCCAGTGTGGGTTATGTT CCCTCACCCACCTACCAGCCTTCACCCAAAATACAGATGAGTGATGCCAAGGTGTTCAGC AACAGGCTTGCCAATGAGCAAAACTGGGTGAACATGGCCACCGAGCAAGGTAGGAGCAAC AAAGGGAATGGGCAAGTCCGCCCACTGGTTGGCTCCCTTGAAGGAACTAAAGAGGATGTA TTGGAAACTTCAGATTCACCCAAAGTTGGCAAGACTCGCCCTCTCAAAAGTAGTAGGGCA AATAGCAAGGAGAAATCCATTCACCTTACAGTTTAG

>Lc-gjb1-XM_006005446-GJB1-32

ATGAATTGGGCAGGTCTGTACGCCATCCTCAGTGGGGTGAACAAACACTCCACGGGAATT GGACGGATATGGCTTTCTGTGATCTTCATCTTCCGGATCATGGTCCTGGTGGTGGCGGCC GAGAGCGTCTGGGGCGACGAGAAGACGGCCTTCATCTGCAACACCCTGCAGCCCGGTTGC AACAGCGTCTGCTACGACCATTTCTTCCCCATCTCCCACATCAGGCTGTGGGCCCTTCAG CTCATTCTGGTGTCCACACCGGCCCTCCTGGTGATCATGCACGTGGCCCACCAGCGACAC AATGAGAAGAAGTTGCTAATTCTGACCGGCCACGCAAGCCCTAAGGAGATGGAACAGGTC AAGAAACACAGGTTCAGGATCTCTGGTACCTTGTGGTGGACGTACACTGTCAGTGTAATC TTCAGAATCATCTTTGAGGTCACCTTCATGTATATCTTCTATCTGATCTACCCAGGATAC CAGATGATCAGACTGGTGAAGTGCGAGGCCTACCCCTGCCCCAACACGGTGGACTGCTTC GTCTCCAGGCCCACCGAGAAGACCATTTTCACTGTCTTCATGCTGTCCGCCTCGGGGATC TGCATTATCCTCAACATCGCAGAATTGGTCTACCTGATCATCAAGGCTTGTACCCGGCAC TTTGGGAATCCAGACAGCAAGTCCTATAGAACCAAACTCTCAGAGTACAAACAGAATGAA ATTAATCAGTTGCTCAGGGAGCAGGATGGCCTCACCTTGAGGGACTCCAAAATGAATGAT ATGAAGAGGAACCCACCATCCGAGAAGGGTGACCGCTGCTCAGCTTGCTAA

>Lc-gjb3.1-XM_005988488

ATGGATTGGAAGACTTTTCAAGCTCTTTTGAGTGGGGTTAACAAATACTCCACAGCTTTT GGTCGGGTTTGGCTTTCAGTGGTGTTTGTCTTCCGTGTGCTTGTCTACGTGGTGGCGGCA GAAAGGGTGTGGGGAGATGAACAAAGGGATTTTGACTGTAATACTAAACAGCCTGGCTGC ACCAATGTGTGTTATGACGATTTCTTCCCTATTTCTCACATCCGGATATGGGCCCTACAG CTCATCTTTGTCACCTGCCCCTCCCTTTTGGTAGTCATGCATGTGGCTTATAGAGAAGAC AGAGAGAGGAAGAACAAACAGCAACATGGTGAAAACTGTCCCAACATCTACAAAAATACA GGAAAAAAACATGGTGGCCTCTGGTGGACCTACGTGATAAGCTTGTTTTTCAAGACGGGA ATTGAAATTGCCTTCTTGTACATCCTCCACCACATTTATGACAGCTTCTACCTGCCAACA CTCGTCAAGTGCACCCAGGATCCATGCCCCAACATTGTTGACTGTTACATTGCTAGGCCC ACTGAAAAAAGGGTCTTCACTTACTTCATGGTGGGGGCCTCTGCTTTTTGTGTAGTATTA AGCGTCCTTGAAATAATCTATCTCATCAGCAAACGGATATTCCACTGCATCCGAAAGCAA AGGAAAAGATCCAGCAAGAATTCAATGACCTCTAGCAAGGCATCGACTGCCCTCTATCAT AATAGACATTATCATTTCAACATGAAAACAATGAATGATATCAAACGTATGGACAGCCTG CATGCCTCTGCCCCCAATTTATCTTTGTTGCATTAA

>Lc-gjb3.2-XM_005988487-gjb3like

ATGGACTGGGAGGAGTTTCAAGATGTCCTGAGTGGGGTGAATAAGTTCTCCACATCATTT

GGTAGGATTTGGCTCTCAGTGGTCTTCATCTTTAGGGTCATGGTCTACCTGGTGGCTGTA

GAGAGTGTTTGGGAAGATGATCAGGAAGACTTTGACTGCAACACTGAACAGCCTGGGTGC

ACCAACGTCTGTTATGACATGATTTTTCCCATTTCCCATATTCGGTTATGGGCCCTTCAG

CTCATCTTTGTCACCTGCCCCTCCCTACTGGTAGTCATGCATGTGGCCTACCGAAATGTG

CGGGAGAGGAACCATAGGCAGAAATATGGGGAAAACAGTGGCAAACTTTATGAGGATGTA

AGCAAAAAGGAAGGAGGTCTATTTTGGACTTATCTAATCAGTCTTCTCTTCAAAAATGGA

TTTGAGATTGGCTTTCTGGTGCTTATTTACAGGATCTACAACAGCTTTGCCTTGCCCCGT

CTTCTCAAATGCAGCGAAGCCCCCTGTCCCAACACAGTTGACTGTTACATTGCTAAGCCA

AAGGAGAAAACAATTTTAACTTACTTTATGGTGGGTGGTTCTGTCCTTTGCTTTGTTCTG

AATATCATAGAGATGTTCTACCTCATTTTCAGCCATATCTATAGTCGCTGGCAGGTCCAG

CAATCCAGAAGAGAGGAGTTTTCCTTTCATGAAGAAGCCAGTAGGCGTCATTTGAGAAGG

AAGTCAATGAAGACACAGCAATTGTAG

>Lc-gjb7-XM_006009197-GJB7-25

ATGAGCTGGAATTTTTTCCTTGATGTCCTGAGCGGGGTGAATAAATATTCCACAGGGATT GGAAGAATCTGGCTGTCTGTGGTATTTATATTCCGGCTGTTCATTTATGTAGCTGCAGCA GAACAGGTGTGGAAGGATGAGCAGGCAGACTTTGAGTGCAACATCAGGCAACCTGGCTGT GAAAATGTGTGCTTCGACTATTTCTTCCCCATCTCTCAGATCAGGCTTTGGGCCTTGCAG CTCATACTGGTGTCCACACCTTCTCTGCTGGTGGTTCTTCATGTGGGGTACAGAAAGCAC AGGGAGAAGAGGTACAACAAAAAACTGTATGAAGACACAGGGAGCATGGATGGTGGACTG TGGTGCACCTATATCATTAGCCTCATCTTCAAAACAGGATTCGAGATTGGTTTTCTCTTC ACTTTTTTCTGGTTATATGGTGGGCTTAATGTTCCCCGACTTGTGAAATGTGACATGCGT CCCTGTCCCAACACAGTTGACTGCTACATTGCCAGGCCAACAGAGAAGAAGATATTTCTC TATTTCATGACTGCTGCGTCTGGGTTGTGCATTGTGCTGAATGTGTGTGAGATGAGCTAC CTGATCATCAAACAATGTTGGAAGTATGGTGTAAAGAGGTACAATTTGAATATTAAGACA TACCAGCCTCAGAAGTTTCAACAGTGCGACCACAAGGAGAATCCAACTTATGTTCCAAAC AACAGCTCCAGTCCCATTCGTAATGTTGACAAAAGCCAGCAAGAAGACACACTGCACCAA

AAGGCTTGA

>Lc-gjb8.1-XM_014493276-gjb2 Locates on scaffold JH127554, and with XM_006004019 and XM_014493277 as close neighbors.

ATGAACTGGGATGCACTTCGTTCTGTTTTAGGAGGAGTTAACAGACATTCTACTAGTATT GGCAAAGTATGGCTCTCTGTCCTCTTTATTTTCCGAATTATGATCCTTGTTGTGGCTGCT GAAAAAGTCTGGGGAGATGAACAATCACAATTTGTCTGCAACACCAAGCAACCTGGTTGT GAAAATGTCTGTTATGATTACTTCTTTCCAGTCTCCCACATAAGACTATGGTGTTTACAA CTCATTTTTGTGTCCACTCCTGCCCTTTTAGTTGCCATGCATGTTGCCCACAAGAAACAT GAAGAGAAAAAGTTCATACTCCAAAGAAATGAAGAAGCAAAAGAGAAAGATATTGAAAAA CTTAAGCTACAGAAGGTTCGGATTTCTGGAGCACTGTGGTGGACGTATACCAGTAGTATT TTCTTTAGACTTCTCTTTGAAGCAGGCTTCATGTATATGTTCTACTTTCTTTATGGTGGG TACCAGATGCCTCGGCTGGTAAAATGTGATACTTCACCCTGCCCCAATACAGTGGACTGT TTTATTTCCAGGCCCACTGAAAAGACAGTGTTTACCATTTTCATGATTGGCGTGTCTCTT GTTTGCATACTGCTAAATATGACTGAACTGCTGTACTTGATAATAAAGGCATGCATAAGA

CACTTTACAAGATAA

>Lc-gjb8.2-XM_014493277-gjb2like

ATGAACTGGAGCACGCTGTACACTGTGTTAGGAGGAGTGAACAAACATTCTACTAGTATT GGGAAAATATGGCTCTCTGTCCTATTTATTTTCCGTATTATGATCCTTGTTGTGGCTGCT GAAAGTGTCTGGGGAGATGAACAATCACAATTTGTCTGCAACACCTTACAACCTGGGTGT GAAAATGTCTGCTATGATCACTTTTTTCCAGTCTCCCATATAAGACTGTGGTGTTTACAA CTCATTTTTGTGTCCACTCCTGCCCTTTTAGTTGCCATGCATATTGCCCACAAAAGACAT AAAGAGAAAAGGTCCATACTCCAAAGAAATGAGGAAACAAAAGAGAAAGATATTGCAAAT CTTAAGCAACAAAAGCTTCGGATTGTTGGAAATCTTTGGTGGACATACACCAGCAGCATT TTCTTCAGAATAATCTTTGAAGCTGGCTTCATGTATGTGTTCTACTTTCTTTACAATGGG TACCAAATGCCCCGCTTGGTAAGATGTGATGCATCACCCTGCCCCAACACAGTGGACTGT TTTATTTCCAGGCCCACTGAAAAGACAATATTTACCATTTTTATGATTGGTGTGTCTGTC CTTTGTATGTTGCTAAATGTGGCAGAACTACTTTACTTAATGATAAAGGCATGCATGAGA CAATCTAAAACACATAATTCAAAACATTACCACCCACACCATGTATCTATAGGGAAAGAA ATTGAACAAAATGAAATGAATAAGCTTCGGTCTAATTCTACCAGCTGTTAG

>Lc-gjb8.3-XM_006004019-gjb2like

ATGAACTGGGGCACCCTATACTCTGTTTTAGGAGGTGTGAACAAACATTCTACTAGTATT GGAAAAATATGGCTCTCTGTTCTCTTCATTTTTCGTATTATGATCCTCATTGTGGCTGCT GAAAGTGTCTGGGGAGACGAGCAATCGGACTTTGTCTGTAACACCCTGCAGCCAGGGTGC AAAAATGTCTGCTATGATCAGTTCTTTCCAGTATCACACATCAGACTGTGGTGCTTACAG CTCATTTTTGTGTCCACTCCTGCCCTTTTAGTTGTTATGCACGTTGCCCACAGAAGACAT AAAGAGAAGAAGTCCATACTCCAAAAAAATGAGGAAACGAAAGAGAGAGCAATAGAAAAT CTTAAACAGCAAAAGCTTCGAATTGTTGGAGCTCTGTGGTGGACATACACCAGCAGCATT TTCTTTAGAATGCTCTTTGAAGCTGGCTTTATGTATATTTTCTACTTCCTTTACAATGGA TTCCGAATGCCTCGGCTGGTTAAATGTGATGCTTGGCCCTGCCCCAACATAGTGGATTGT TTTATTTCCAGGCCCACTGAAAAGACAATATTTACTATTTTTATGATTGTGGTGTCTGCT GTTTGCATGGTGTTGAATGTGGCTGAATTAGTTTACTTAATAATAAAAGCATGTATTAGA AACTTCAGAAGACACAACCCAAGGAATTACCACTCCAAAGGGAAAGAAATCCAACAAAAT GAAATGAATGAACTTCTGTCTGGTTCTTCCAGCTGCCAGACCACTGTTACAAGTGTTATC

AAAATAGCTTAA

>Lc-gjb10-XM_005988489-gjb4

ATGAGTTGGGCCTTCCTCCACGGCATAGTGAGTGGAGTTAATAAGTACTCCACAGCTTTC GGTCGAGTATGGCTGTCAGTGGTCTTCATCTTCCGTCTGTTAGTCTATGTGGTGGCTGCT GAACGTGTCTGGGGTGATGAACAGAAGGACTTTGACTGTAACACCAGACAGCCTGGTTGC ACCAATGTCTGTATGACTACTACTTCCCCGTCTCTCACATCCGGCTGTGGGCTCTTCAG CTTATCTTTGTCACCTGCCCATCACTGCTGGTGGTCATGCATGTGGCCTACAGGGAAGAC AGAGAGAAAAAGCACAGGGAACACGAAGGTGAAAACTACAGAAAATTGTATGTTAATACA GGTAAGAAAAGAGGAGGACTATGGTGGACCTATCTCTTCAGTCTCGTCTTCAAAGCAGGT GTAGATGCACTTTTCTTGTATATCTTTCATAAACTGTATGAAAATTACGACATGCCCAGG CTGGTGAAATGTACCATCTCACCTTGCCCCAACATCGTTGACTGCTTCATTGCCCGGCCA ACTGAGAAAAAGATCTTCACAATTTTTATGGTGGTGACATCGGCCCTATGCATCCTCTTG AGCATATGTGAAATTATTTACCTGGTGGGCAAGAGGTGTCATAAGCTCATCAGATCCCCC AAAAGTGAAAGTAACAGGAGCCTCACTACTACTTCCCTTTTAACTGACAATTACTCTGGA GAACCTCCAACCTATGGTTTTAAAAGTCATGCTGTAAATGGAAAAGAAAGCAAGCCCCCT CAGATTCAAGTTACTGGACATGATCACTAA

>Lc-gjc1-XM_006000156-GJC1-45

ATGAGCTGGAGCTTCTTGACCCGCCTTCTAGAGGAAATCCATAACCATTCAACTTTTGTT GGCAAGATATGGATGACGGTCCTGATTGTGTTTCGTATTGTCCTTACAGCTGTTGGAGGA GAGTCCATATACTATGATGAACAGAGCAAATTTGTCTGCAATACACAACAACCAGGTTGT GAGAATGTCTGCTATGATGCTTTTGCTCCCCTTTCCCATGTAAGATTTTGGGTTTTTCAG ATCATTCTAATAGCCACTCCATCTGTGATGTACCTGGGCTATGCAGTTCATAAAATTGCC CGAATAGACCAGCAGGACATTAAGGAGAGGAAGGTGAAGTGCAAACCCTTTGCCATGCGT TGGAAACAGCATCGTGGTTTAGAGGATGCAGAGGAGGATAATGAGGAAGATCCTATGATG TACCCAGAGCTAGAAGTAGAAAACGAAAAAAGCAAGGAAAGCAGCACCAAAATTAAACAT GATGGGCGGCGACATATTCGAGATTATGGACTCATGAAAATTTATGTTCTGCAGCTGTTG ATTAGGACTGCTTTTGAGGTTGGCTTTCTTGTTGGCCAGTACTACTTGTATGGATTTGAG GTTACTCCATCCTATATTTGCAGTAGGAGTCCATGCCCTTATAAAGTGGATTGCTTTGTT TCCAGACCTACTGAAAAAACTATCTTTCTCTTGATAATGTATGGTGTCAGCTGTTTGTGT TTGGTTCTGAATATCTGGGAGATGATGCATTTAGGTTTTGGAATTATTCGAGACCTGCTG ATCACCAAGAGGAAAGCTTTGGAAGAATCAGTGGTGTGTAACTATCCATATTCCTGGAAT GCTCCATCAGCACCACCTGGTTACAACATTGCAGTTAAGCCAGACCACATCCAATATACT GAGTTATCTAATGCCAAAATGGCCTATAAGCAAAACAAAGCAAACATAGCTCAGGAGCAG CATTATGGGAGCAATGATGAGAACATTCCTGCTGATTTAGAAAACCTGCAGAAACAAATT AAAATGGCTCAGGAGCATCTTGACATGGCCATCCAGGCTTATAATAACCAAAACATTCCA AATGGTTCTCGAAATAAGAAATGCAAAGGGGGTTCAAACAAAAGCAGTGCCAGCAGTAAA TCTGGTGATGGGAAGACCTCTGTATGGATTTGA

>Lc-gjc2-XM_006006316-GJC2-47

ATGAGCTGGAGTTTTCTCACCCGCCTTCTGGAGGAGATCCACAACCACTCCACCTTTGTG GGGAAGGTCTGGCTCTCGATGCTCATCATCTTCCGCATCGTGCTGACGGCTGTTGGCGGG GAGTCCATCTACGCGGACGAGCAGAGCAAGTTCACCTGCAACACCAAGCAACCGGGCTGC GACAACGTCTGCTATGACGCCTTTGCCCCGCTGTCCCACATCCGCTTCTGGGTGTTCCAG ATCATCATGATCTCCACCCCTTCCATCATGTACTTGGGCTATGCCATCCACAAGATTGCT AAGACATCTGAGGAAGAGAAGAAGTTTAGAGCCTTCAAGAGGAAGCATCCTACCATCAGG TGGCGTTCTGTTCGCAATGTGGAGGAATCCTTGGCTGAGGAAGAGGAAGAAGAGGAGCCC ATGATCTACCAGGACATCATGGAAATCCAAGACAAGACGGAGAGTAAAGAGAACCAGAAG CATGATGGGAGGAGGCGCATCATGCAAGAAGGGCTGATGAAGATTTATGTCCTGCAGTTG CTAGCTAGAGCAATGTTCGAAGTTGGCTTCTTGGTGGGGCAGTATTTCTTATATGGGTTT GAGGTCCACCCAACCTTCCTGTGCGATCGCAACCCTTGCCCTCACAATGTGGACTGCTTC GTTTCCCGTCCAACAGAGAAGACCATTTTTCTCCTGGTCATGTATGTGGTGAGCTGCTTG TGCTTGCTACTGAACCTCTGTGAGATGTTCCACTTGGGATTCGGTACCATCAGGGATGTG ATTCGCACCAGGCGAAATGACAGAGGCAACCATTTGCTCCACAGCTACCCTTACCCAAAG AATATCCCCACTTCCCCTCCCAAGTACAACCTTGTGGTGAAGACTGACAAACCTGCCAAG GTCCCCAACAGCCTGATGGCTCATGAGCAGAACTTGGCCAATGTTGCTCAGGAGAGGCAG TGCACCAGCCCAGATGAGAACCTCCCACCAGACTTGGCTACATTACACAGGCAACTCAGG GTGGCCCAAGAACAGCTGGAGGTTGCCTTCCAGAGTTACAATGGGCAAATGAATGCACAT CCTTCCAGGACCAGCAGCCCAGTGTCAGGGGGGACCGTGGCTGAGCAGAACAGGGTCAAC TTGGCTCAGGAAAAACAAGCTCCCAAAGAAAAAACTACCGTCGAAAAAGCCGGCACCAGC AGCAAAGAGGCTGGGAAGATCTCGGTTTGGATCTAA

>Lc-gjc4-XM_005991770-gjc1like

ATGAGTTGGGCCTTCCTGACCCGTCTTCTGGAGGAGATCAATAATCACTCCACCTTTGTA GGCAAGGTATGGCTTACGGTACTCATAATCTTCCGCATTGTGCTAACAGCAGTGGGAGGG GAGACCATCTACTATGATGAACAGAGCAAATTTGTCTGCAACACACAACAGCCTGGTTGT GATAATGTGTGCTATGATGCCTTTGCACCCCTCTCGCATGTCCGCTTCTGGATCTTCCAG GTCATCATGGTGAGCACACCATCCATCATGTACTTAGGCTTTGCCATGCACAAGATTGCC CGGGTGCCTGATGGTGACGAGAAGAAGAAGAAGAAGAAACCACGGCGCATTCCCATTATC CAACGTGGTCCCAACCGGGATTATGAGGAAGCAGAGGACAATGGTGAGGAAGACCCCATG ATTGTTGAAGAGATCGAAAATGAGAGTGGTGCAAAGGAAGAGAAAGAAGACAAAGACAAA CATGACGGAAGGAAACGAATCAAACAAGATGGACTGATGAAGATCTACGTCGTGCAGCTT CTGTTTAGGTGCATCTTTGAAGTGGGCTTCTTGATTGGGCAATACATCCTCTATGGCTTG GAGGTGGCCCCATCCTATGTGTGTACCAGAGGGCCCTGCCCGCATACAGTAGACTGCTTT GTCTCCAGGCCAACTGAGAAGACTATCTTTCTGCTGATCATGTATGCTGTCTCTGCTTTG TGCCTTTTCCTCAACATCTGCGAGCTCTTCCATCTTGGTGTGGGAGGTATCCGGGATGCA CTAAAGAACAAGAGTGTGCACAGGTTTGACTCCATGGGGCATTCTTATTCAAAGCAGACA CCCAGTGCTCCCCCAAGGTACTATTCCTTGATTAAGAAAGAACGACAAAAGATGGGCAAT GGGAAGATGATGTACAGCAATGACATCTGCACTAGCTCAGCGGAGGAGCCTTGTGAGAGA GGGGAGGACCCAGTCCCTACTGACCTTGAGAACCTGAGAAGGCATCTGAAGGCAATTCAA GATCAGCTGGACATGACTTTTAGCACCGGAGAGAAGGAGCATGCTTCCCGAAGTGGTAGT CCAGAGTCCAACGGCACTGCTGCTGAAGAGAACCGCCTGAACTTTGCACAGGAAAAAGGG GCTGAAGGTAAAACAAAAGGTAATGGAAATGTTTCTGTATGTGTATGTATTTAA

>Lc-gjd1-XM_005987869-gjd2like

ATGGGGGAATGGACTATTTTGGAGAGGCTGTTGGAAGCAGCCGTCCAGCAGCATTCTACA

ATGATAGGAAGGATCCTTCTCACGGTGGTGGTGATTTTCCGGATTCTAATCGTGGCTATC

GTTGGCGAGACGGTATACGAAGATGAGCAGATCATGTTTATATGCAACACCCTGCAGCCC

GGGTGCAACCAGGCCTGCTACGACAAAGCCTTTCCCATCTCGCACATCCGCTACTGGGTC

TTCCAGATCATCTTGGTCTGCACCCCTAGCCTCTGCTTCATCACCTACTCCGTCCACCAG

TCTGCCAAGCAAAGGGAAAGACACTACTCCATCCTCTACCCGGTTCTGGACAAGGACTTT

GGCCGGGAGAGCAAAAAGGCCAAGAATGTCAACGGCATCCTGGTCCAGAACCCGGAGGGC

TCTTCCAAAGAGGAGCCAGACTGCTTAGAAGTGAAGGACATTCCCAATACACCCAGGAGG

ACCTCTAAGAGCTACAAGATCAAGCGGCAAGAAGGAATCTCCAGGTTCTACATCATCCAG

GTGGTCTTCCGAAACGCTTTAGAGATTGGCTTTTTGGCAGGGCAGTACTTCTTGTACGGG

TTTAACGTTCCAGCCATCTTCAAGTGCGACAGGTACCCTTGCATCAACAGTGTGGAGTGC

TACGTCTCCAGGCCCACAGAAAAGACCGTCTTCTTGGTGTTCATGTTTGCTGTCAGCGGG

ATCTGCGTTGCTCTCAACCTGGCTGAGTTGAACCACTTGGGCTGGAGGAAAATCAAAACG

GCCATCAGAGGGGTGCAGGCCAGGCGGAAATCCATCTCTGACATCCGGAAGAAAGAACTA

CCCCCCATAGCTCAAATGCCTAACTTGGCGAGGACTCAGTCCAGTGAATCTGCATACGTC

TGA

>Lc-gjd2-XM_006004060-GJD2-36

ATGGGAGAATGGACCATCTTAGAGAGACTCCTGGAGGCTGCTGTTCAGCAGCACTCCACT ATGATAGGACGGATTCTGTTAACAGTCGTGGTGATCTTTAGGATCCTGATTGTGGCTATA GTTGGGGAGACAGTGTATGATGATGAGCAAACCATGTTCGTTTGTAATATCCTCCAACCA GGCTGTAATCAAGCTTGCTATGACAAGGCATTTCCAATCTCCCACATCAGATACTGGGTA TTTCAGATCATCATGGTATGCACCCCGAGTCTTTGCTTCATTACTTATTCTGTGCACCAA TCTGCCAAACAAAGAGAAAGGAGATATTCCACAGTCTTTCTCACCTTAGACCGAGAACAG GAGAGTCTGAAACGAGAAGATAGTAAGAAGATTAAGAACACCATTGTCAATGGAGTCCTT CAAAACACAGAAAACTCTACAAAAGAAGCTGAACCAGACTGTTTAGAAGTCAAAGAAATT CCAAATTCAGTGATGAGAACTACAAAGTCCAAGATGCGACGTCAAGAAGGGATCTCCAGA TTTTATATCATTCAAGTAGTATTTAGGAATGCATTAGAGATTGGATTTTTGGTGGGACAA TACTTCCTTTATGGATTTAACGTTCCATCAACGTATAAATGTGATAGATACCCATGCATT AACGAAGTCGAGTGTTATGTCTCAAGGCCAACAGAGAAGACTGTCTTCTTGGTTTTTATG TTTGCAGTCAGTGGAATATGTGTAGTTCTCAATTTAGCAGAACTGAACCATCTGGGCTGG AGGAAGATTAAAATGGCAGTGAGAGGAGTACAGGCTAGAAGAAAATCTATTTATGAAATC AGAAATAAGGACCTACCAAGAATGAGTGTGCCTAACTTTGGCAGAACTCAGTCAAGTGAC

TCAGCCTATGTGTAA

>Lc-gjd3-XM_006000524-GJD3-31.9

ATGGGAGAATGGAGCTTCCTCAGCAGTTTTCTGGAATATCTCCAGAACCACTCACCCATG GTGGGCCGTATCTGGCTGGTAGTCATGTTGATCTTCCGCATCCTGATTCTAGCCACGGTG GCCAGCGACATGTTCGAGGATGAGCAGGAGGAGTTTGCATGCAACACCTTGCAGCCGGGG TGTAAGCAGGTCTGCTACGACAAAGCCTTCCCCATTTCTCACTACCGCTTCTGGGTCTTC CACATCGTGCTGCTGTCGGGCCCTTCCATCTGCTTCGTTATCTATGCCATGCACCATGCT TCCAAAATAGAAAAGGAAAAAGAAGCACTGGAAAGTGGGGTGAGCAGCAACAAGTCAAAA CGGCAAACATACAACCAGGAAGATAACTTACGCAAAATCTACATCGGGAATGTAGCCTTC AGGCTTTTGCTGGAGGTTGGCTTCACCGTTGGCCAATGGGCTCTCTATGGCTTCAAAGTG GAGGCCCAGTTCCCCTGCGAACGATTTCCATGCCCTTATACTGTAGACTGTTTTGTCTCA CGTCCAATGGAGAAGACAATATTTCTACTCTTCTACTTTGCAGTCGGGGTGCTGTCTGCT GTCCTGAGCTTGGTCGAGCTGATCACAATCATACTTAAACGGAAGAGTCGTGGCAAAACC TCTTCCTACGAGAAGGACAATCGGTCCAATAGCAAGCAGGAGAGATCAGAGACTTTACTG CTGGCAAAATTCCCGCCTTACCCTGTAGAAAAAAGCCAAGACATTCCAAATCATGTGGGC AAAAACTCTACCTACAAGGGCAGCTCAGCAGACAGCAATACCAACAGCACCAAATCTTTC AGATCAGTTGACAAGAAAGGCTTGAAAGTATAG

**>**Lc-gjd4-XM_005994831-GJD4-40.1

NGAAAAATCTGGCTAACGTTGATAATTTTGCTTAGACTTCTTGTGACTACCCTGGCAGGT

TATCCACTCTATCAGGATGAGCAGGAAAGATTTGTTTGCAACACCATACAGCCAGGATGC

TCAAATGTTTGCTATGATCTGTTTTCTCCCATGTCCCACTTTAGATTTTGGCTCATCCAG

ATTGTTACCTTGTGCCTACCATACGCAATATTCTGTGTTTATGTAATTCACAAGGTTACA

AAACGAATTGGGTCAGATACAACATTTAAACGGGCTTCATTAACGAGGACAACCTATGAA

GTGGAAAAGCAAAAAATACCTGAATTCCTGGGGGCTTATGTATTTCAGCTGCTTTTGAGG

ACTATAGCAGAGGCTGGATTTGGTGCTGGGCAGTACTACCTATATGGATTCTTTGTCCCA

CAGAGATTTGTTTGTCACCAGCCTCCTTGCACAAGCATGGTAGACTGCTACATTTCAAGA

CCAACAGAGAAAACAATTATGATAAATTTTATGTTTGGAATTAATGCCATGTCCTTTCTC

CTTAATGTTGTGGACCTGATCTATGTGGTAAAGAGAACTATGAGACAAAATCACAAAAAT

AAACTGCTTATGGAAAATTTCTATCAAGAGGAAGAGTATTGCATTGCTCCACATAGCAAG

CACTCAGAAGTTCAGTCAGCTCAAGAGTACAGTGGCAATAAAAGAAGAGGAAGCCATATA

AGTGAAGAAAGTGGAGCTTCTGTTAGATCAGAAGAGGAAGAAATTAGTTCTGTTCACTCT

GAAATTCTGTCACAAAATACTGCCAGATCTAATAACAATAGCAACAACACATATATGCAG

AACAGCCTTGCTGCAGAAGGATATCCTGAGCAAGATGGTAGCGAAGTGGCCTTGTGTCTG

AATGAGTGCAAGGTGCCTGCTGCCACACAAGGGAAATATGGAAAATGGGGACAGGGAAAA

AGTTCCAGCACAAAATCTCGCCAACTAGGTTTTGGAGATCCTTCAACTGCAATCCACACA

AGGTTTATAGGACATTACTCACTAGTAGAAATGAACCAAGTGGATTTGCAGTCCAACTGT

AGCAGTCCTGGTCATAGTTCACAAACCAAATCTGAATGGGTGTGA

>Lc-gjd5-XM_005996992-gjd2like

ATGGGCGACTGGTCAATCCTTGGTCGTCTCCTCACTGAAGTCCAGAATCATTCTACTGTG ATTGGCAAGATCTGGCTGACTATGCTGCTCATTTTCCGGATCCTCCTGGTCACCTTGGTG GGAGACGCAATCTACAGCGATGAACAGTCCAAGTTCACTTGCAACACCCTCCAGCCTGGC TGTAACAACGTTTGCTACGACACTTTTGCCCCCGTGTCCCACCTCAGGTTCTGGGTGTTC CAGATCGTCTTAGTCTCTACCCCCTCCATCTTTTATATTATCTACATCCTCCATAAAATA GCAAAAGATGAGAAAAGGGAAGTGGAAAAGGTCTACATGCAAAGACTGCCAACTGATCAG CGGCTACAAGAGGAGTTGGGCTTAAGTCAAAGGCTTGATAGTTTTGAAACTCAAATGGTT ACCAGCAATGGAGAAGATGAACAACAAATTCTTGGAAATGACATTAAAGATCCAACACAG CTGTCCGACAAGGTGTTGATAATATACATTGTCCATGTCATCCTGAGGTCTGTCATGGAG GTGGTTTTTTTAGTAGGACAATTCTATCTCTATGGCTTTGAGGTTCCTCACCTGTTCCGT TGTCAAACGTACCCCTGCCCCACCAGAACTGACTGTTTTGTATCCAGGGCCACAGAGAAG ACAATCTTCCTCAATTTCATGTTTGGAGTAAGCCTTGGGTGCTTTCTCCTGAACATTGTG GAGCTCCATTACTTGGGATGGGTTTATGTGTTCCGGATTCTGTGTTCCACTTGCTCGGCC TGCTGTGTGTCCAAGAGGAAAAAGAAAGAAAAAATGAAGATTTATTCAGAGCACAACCCT CTTCTAATGCAACTGAAAAACTCCATCCAAGAAAAGCTAGTCATGAAGACCTCCTCCAAG TTGTCTCAAGAGAAACTGGGGTACCTCCCTTCCAACGCAGCTGCAATATCATTTGCAACA GATTCAACACAATGTTCTCCCAAGAAAAGCCCAGATATCAAATCAAAACATGGAAGTGCG ACTAAAGCCGGGAAAGCAAAAAAATCCTGGCTTTAA

>Lc-gje1-XM_014483873-uncharacterized Splice site

ctcagacctccaactgtgattggtcagttccatacccttttctttggttctgttcgcatg

ttcttccttggggttttgggctttgctgtttatggcaatgaagctttgcacttcagctgt

gatccagacaagcgagaggtaaacctcttctgttacaaccaattcaggcctataacacct

caggtattctgggcgttgcagttggtgactgtattagttcctggcgctgtgttccacctg

tatgctgcctgtaaaaatatcgatcaggaagaaattcttcaaaaacctgtttacactgtc

ttctacatcatttctgttctgttgagaatcatccttgaagttatagccttttggcttcaa

agtcatctgtttggttttcaagttcatccactcttcacatgtgatgctagtgctctagat

aagaaaataaatattaccaagtgcatggtgccagaacattttgaaaagaccattttcctc

agtgctatgtatacttttactgtcatcacggtggtgttgtgtgttgcagagatttttgaa

atcttatgcagaagattgggctttttgaacagtcaataa

# Supplementary Figure S27. Reedfish (*Erpetoichthys calabricus*) connexins.

The chronology of the sequences is according to the Greek nomenclature. Size nomenclature is indicated in parentheses. GenBank accession number is given for each entry.

>Ec-gja1-XM_028797787

atgggtgactggagtgctttaggaagacttcttgacaaagtccaggcttattccactgct

ggaggaaaggtctggctctctgttctcttcatttttcgaatcctagtcttggggacagca

gtcgagtcagcctggggagatgaacagtcagcttttaaatgtaacacccagcaaccaggt

tgtgagaatgtatgctatgacaagtcttttcctatctcccatgtccgtttctgggtgcta

caaattatatttgtgtcaactcccacgctactttatctagcgcatgttttctacctaatg

cgcaaagaagagaaattgaacaaaaaagaagaagaactgaaaattgtccagaatgacggt

ggtgatgtagatttgccactcaagcaaattgaaatcaagaaatttaagtatgggctggaa

gagcatggaaaagtaaaaatgaaaggagccttgatgcgtacctacattgtgagcatactt

ttcaaatctgtctttgaagtcggctttctgttgattcaatggtacatttatggttttaag

ctggaggccatttacagatgtgaaagggatccttgccctcataaagtggactgtttcttg

tcccgccctactgagaaaaccatcttcatcatctttatgttagtagtgtctttggtgtca

cttctactaaatgtgattgagctattctatgtcctcttcaaaggcatcaaggatcgggtg

aaaggaaaaagagatccctacacaaacaatatggtcatgagtccagtagctaaagactgc

acatctccaaaatatgcctattacaatggctgctcctccccaacagcacccatgtctcct

atgtctccgccaggctacaaacttgccactggagacaggaccaactcttgtcgcaattac

aataagcaagccaatgagcaaaactgggcaaactacagcactgagcagaaccgtctgggc

caaacaggcagcaccatctcaaactcccatgcgcaagcatttgactacccggatgatgtg

ccagacaataagaagctaccatccggacatgagctacagcctctagctgttgtggatcaa

aggccctcaagcagagccagtagtcgagtcagcagtagagctcgacctgatgatcttgat

gtgtag

>Ec-gja2-XM_028817004-gja3like

atgggtgattggagtttgctgggtaaattgctggagagtgctcaggagcactccacagtt

gttggaaaggtctggctgacggtgctcttcatctttcgcatcttagtgctgggcactgct

gcagagaaagtgtggggagatgaacagtcaggcttcacttgtgacacaaagcaacctggt

tgccagaatgtctgctatgacaagaccttccctatatcacatattcgcttttgggttcta

cagatcatttttgtgtccacacccaccctcatctatctggggcacatcctacacttagta

cgtatggaggagaagcagaaggagaaagagaaaaacaaggtatctcagcatcacggtgat

aagcaaccactcctggggaatagtaaatgtaaaaagccacctgtgaaggatgaacagggc

cggatacggatgcaaggtgccatcttgcggacctacatcttcaacattattttcaaaact

ctgtttgaagtgggctttatagttgctcagtatttcctgtatggatttgagctaaagcca

ctgtacacatgtaatcgtctgccgtgccccaatactgtaaactgctacatctctcgacct

actgagaagacgatcttcattttatttatgcttgctgtagcttgcctgtctttgttcctc

aacctggtggaaatgtaccacttgggcttcacaaagtgccgacagggcctgagatacaga

acagaaattgcatcagaagctggctcaaaggctgctagcgaagctgtagcaccttatgtc

ccaagttacccttactttggagcacaaccaccagctccagttcattaccaagctgggact

agatatagtccatctcccttgactgaaacggattcagtctgtcatccatacaacagcaaa

gtagcttacaagcaaaacagggataaccttgcagtagaaaggaatggaaagcctgaggag

agcaactcaaaactcaaaagagaacctcagtctcccaatgacaaccaacgtaaggctggc

cgtgtcagtagtaagccgaataatggcaagagccggctcgatgacctaaaaatctag

>Ec-gja3-XM_028800566

atgggtgactggagctttttgggcagacttttggaaaatgcacaagaacactcgacggtg

ataggcaaagtctggctgaccgtgctctttatcttcaggatcctggttctgggggctgct

gctgaggaagtttggggagatgagcaatctgacttcacgtgcaacatccagcagcctggt

tgtgagaacgtctgctacgacaaggccttccctatctcccacattcgcttctgggtgctt

cagatcatttttgtgtcaacgccgaccctcatttaccttggtcatgtcctgcatattgtg

cgaatggaggagaagaagaaggaaaaagaagcggagctgcgcaagtcacaaagacatctg

gaggaaaaggagctcctctttaaaaacgacggcgcagtgacaggtaaaaaagagcggctt

cccatccgagacgagcatggtaaaatccgcattaggggagcgcttttgcgtacctacgtc

ttcaatatcatctttaagaccatatttgaaattggatttattgtaggtcagtattttctt

tatggttttgaactaaaacccctgtaccggtgtaaccggtggccgtgccccaacattgtg

gactgctttatttctcgtcccacagaaaagaccatcttcatcatatttatgcttgtggtg

gcttgcatatcacttttgctgaatttgttagagatttatcaccttggctggaagaagata

aaacagggaatgacaaacgggttatccccggaccaggaactcccttgccacaaggttgac

ctggaacccaataccacaagtcccagaactgcccctgccagtcttagctatccaccttat

tacactgaggtcgttgcggggggtccacaagattaccctgaggcccccactgcagagttc

aagatggcaccgctctccgtggaccagcaccaagcacctcccttctatattaacaacaac

aaccacaggctggccacggagcagaactgggcaaatctcgctaccgagcagcagactcgt

gagagaaagcctgcctccctttctgcctccaccatcagcagtgtccacaacgagcaaaac

caggacacgacggcagacgccagcagcggaagtgcgagtggccggaaaagcgaggaggac

gagtgccacgtgaccaccactactgtggagatgcacgagcctcccgcgctcagcgacgtc

cggagattcagcagggcgagtaaaggcagtggcagcaaggccaggtccgatgacctcgct

gttta

>Ec-gja4-XM028819478-gja4like

atgacagattggtctttcttggagcgtgtgctcgggcaggtcaatgagcactcgactggt

gttgggaaagtctggttgacagtgctcttcatattccgggtgatggtactgtgctcagct

gcagagacagcctgggatgatgagcagtctgaatttctctgcaacacgctacaacctggt

tgccaacttgtgtgctatgatcttagcttccccatctcccattaccggtactttgtgctg

cagatcatttttgtctccacacccacactcatctatcttgggcaccttgcagtacgggca

agccgaaagaagaaagagcagcaagagaatggcgtggaaggaaatgaggaggcagtggga

tatggagagtcaaaaagggatatgcaggaacatgaatttcagaatgttcccaaactgaaa

ggagctttaatgatcacctacactataagtattgtctgcaaagtaatcttggaagctggc

ttcatgctggggatgtggttcctgtatggtttctttattcagcctcgctatgagtgccaa

ggatttccgtgcccttacaaagtggactgctttgtttctcgaccaactgagaaaaccatt

ttcaccatctacatgcaagtacttgcactgatctctcttgtgcttaatgtcatagagctc

tttcatttacttggcaagacaatggtaaggagagcgattaaatactatgaaaatcagagt

gcccttcaggtttacaagagaccagtagtttatgagcccactctgaattatatggagaat

gcccagctctgcttaccaatggctagtgccagtaaccatagccattttagtgatccaaat

attaatttcaaaccacaagaaagtggtacggattcagaaagaaacgacagacaacagagg

aatattgagtccaaatctgaccagctcccaggatatccagggtatctagaagagcacaat

gaaaaagaaatggcatcggggagactaacagatagctctgaaggacagagaaggcactat

gtatga

>Ec-gja5-XM_028800789-gja5like

atgggtgactggaccttcttaggaaatgttctcgataaagttcaacagtactcaacaatg

gtgggaaaggtctggctgactgttttattcattttccgtatcctagtcttaggcacagca

gctgagtcttcctggggcgatgagcggactgactttctatgcgacacccttcagcctggt

tgcacaaatgtttgctatgataagatgttcccaatctcccacatccgttattgggttctc

cagtttatatttgtttccaccccctcactaatctacatggtacatgctttacatttggtt

catatggagcaaaagaggatgcagaatgaagacagtgaaagatcacctccaaggcataag

gagtatcccgaagaaacagaagaacctattgtgaagattccattgaagggagctttactc

aaaacatatgtgtttagtatcatcataagaaccttcattgaagtggctttcattttgggc

cagtattatttgtacggcatattacttaaagtgctttatgtctgccactcctcaccttgc

ccaaatgctgtcaactgctatgtatccaggccaactgagaagaatgtattcattgtattt

atgcttgttgttgctgcactgtcacttttgttgagtttactcgaactttaccatcttggc

tggaagaaagtgaaagaatgtttacagagctataaagtgccgccctcacaagatgatgca

atccctgccaccaatgaaggccaaggcaaaattgcacaggcctgcacaccaccgccagac

tttgatcaatgccttgtgcaaccaacttcccattgccagacattcagtgacaagatggct

caccagcaaaactcagtcaatctggcaacagagcttcatagaagcagtgataacctgggt

caggaagattttctgcacatgaattatatgcaaggctgtaacgaatctaatgcaacaccc

actgtatatattgacagctttttaaagggaaagtgcagactcagtaaagcaagtggatcc

agtagtagagtgagaacagatgacttaactgtatga

>Ec-gja8-XM_028801326

atgggtgactggagttttttaggaaacatactagaagaagtgaatgaacactccactgtg

ataggaagagtatggctgacggtgctcttcattttcaggatattaatccttggcactgca

gctgaatttgtgtggggagatgaacagtcagattttgtgtgcaacacccagaccccaggt

tgcgaaaatgtctgctacgatgaggcttttcccttgtcacacatcagactgtgggtcctc

cagatcatttttgtctccactccatcgctggtgtatgtcggccatgctgttcactatctg

aggatggaagaaaaaaggaaggaaagagaagaagcagagatgaacagacaacaggagatg

aatgaggagaggctaccccttgctccagaccaaggaagtgtaagaacaaccaaagaaaca

agtaccaaaggcagcaagaagttcaggctggaaggcacaattctgaggacttacatctgt

cacatcatcttcaaaactatttttgaagtggggtttgttgtaggccaatatttcctctat

ggcttctacattcgccctctctacacgtgtagccgttggccttgtccaaacacagtggac

tgctttgtgtccagacctacagagaagaccatttttgttatatttatgctggcagtggct

tctgtatctctctttctcaattttgtggagattggtcatttgggtctaaagaaaataagg

tttgcatttagaaaaccacctcaaacccaagcagagatggttatggagaagcatttgcat

tccatagctgtttcttcactccagaaagcaaaaggttataagctccttgaggaagacaag

ccagtgtcacacttctaccctctaacagaagttggaatggaagccagcaatttgccagct

ccgtttgctccttttgaagggaaagataatgctgccagtcctctgcaagatgtttcaaag

gtctacgatgaaacattaccgtcgtatggtcagagcacagagcaggaagaagaaatttca

gaggaacctcccgctgaaactgcagacacattagaagatacaagacctctaagcaggtta

agtaaagcaagcagtagagccagatcagatgatttaactgtatga

>Ec-gja9-XM_028819167

atgggtgactggaacttccttggaggaatcttggaagaagtgcatattcactccaccatt

gttggaaagatctggctgacaattctctttattttccgtatgctggtccttggggttgca

gcggaagacgtgtggaatgatgaacaatcagacttcatttgtaacactaaacagccaggc

tgtagaaatgtctgctatgacagggctttccccatctctctcatacgctattgggtcttg

caagttatctttgtgtcttcaccctctttggtatacatgggtcatgctttatacagactg

agagctttagaaaaagaaaggcaaaggaaaaagacccacctcagaaaagagcttgattca

atggatgcagactgctccgaagttcgaaggaggctagagagagaacttaggcatctagaa

caaagaaagctgaacaaggcaccactcagaggatcacttctacgaacatacgtaatacac

attgttacaagatctgcagttgaagtagggttcatggtgggccagtacctgctctatggg

ttcaacctagagccattgttcaaatgcgaaaaagatccatgtccaaacctagttgattgc

tttgtgtcacggccaatggagaagactgtttttatggtttttatgcagtgcatagcagca

gtttctttgtttcttaatattctggaaattatccatttaggttataagaaatttaaaaat

ggactacttgattattattcaaatcttaaagatgacttagatgattattatatgagcaaa

tctaagaagaactctgtggtacatcaagcatgtactgtttcatcattgactcgtaaatct

accattcctactgcgccaagtggctataccttactgacagagaagcccagtgaggtttcc

gtttacccagtgcaaaatccactctcagccttcatgccatttcaatgtaatccatgtaca

gaaggtagtaaagagttcatcaatcataaccttagtgactacaatgcctccaaagggacc

gagggactcggacttcctgggactgatacaaccactgtacacactacacagaagattgag

gatcagaggcaagatgaaaccaaaaagtttccagatgaacatgcaggctctgtacaacac

tcctcacagcaaccagagacatcatcctgtcatgtgccacgtagtggggcaggtcggaag

ccacgcagggtcagtgcaccatggaactgctccacagtggtggaaagtaacggatcaaat

ggggacacaagtgtaagcagttacaaaggtagatgtacttacagttctctcaatgttcag

gcagtttcaaagtccgtcctaaaacagtcaaaccgtccacagactccagactcatttggc

gagtcaagttcggaatcaaagcacagtcgaaacagcaatagccctcgtgcatcatctcca

accagaagaatttctttggcaagtagtagcaacagcagcaggcatgctcctggtgacctt

caaatttaa

>Ec-gja10-XM-028791089

atgggggactggaacttgcttgggagtatcttggaggaagttcacattcactccactatc

gtagggaagatctggctaacaatcctctttgttttccgtatgttggtcttgggggtggca

acagaggatgtctgggatgatgagcagtccaagttcatctgtaacacagagcagccaggt

tgcaggaatgtctgttatgataaagccttccccatctccctcatccgatattgggttttg

cagatcatctttgtgtcgtcgccttccttggtgtacatgggacatgcactttacagatta

agagctttggaaaaagagcgacagaagaagaaggcacaactgcgagcagaactggaagaa

cttgagcctctactggatgaatacaggaagcttgagcgggagctcagaaagctcgaagaa

cagaagaaggttaataaagcacctctgaggggatccctgctgcgtacctatatctttcat

atcctaactagatctgttcttgaagttggttttatggtgggccagtacattttatatgga

tttcagctggattctgtctataaatgcaccaagatgccatgtccaaatactgtggattgt

tttgtgtcaagacctacagaaaaaaccatattcatggtatttatgcacagcattgctgct

gtttcactatttttaaacctcttggaaatatctcacctgggtgttcggaaaattaaacag

agtctgtatgggacctcccgatgccagggcgcagaggatgacctaatcatcagtaagtca

aagaaaaattccatggtccagcaggtctgtatcttgagcaactcttcacctcacaaaatt

atccccttaactcagaccagctacaaaatattacccgaagggcaggctgatgcccttcca

atttatctgcctcctgcaggctttcaaacaattcatgagcaaactgatgaaaaggatgag

gtgcctagacaaaccccacctgagcgcattcattgtcaaggtgatgaacacccaaggaag

ctggcagaatgtagccagagtcagcatcacatcttgcaccctgagaagggatctgagagg

cgcaataccctggagaatcgtgatcattcctgcagcagtgaggactccaacaataacaga

acacatgtcaatgggattggctccaagaacatacctcacccagccaaggcagccatccat

tccagccacatggagatcccagcggcagtacgcaatgcattacgcaagcacagtcgtgtc

agtgtttgcaaggactatggggaagaccgcagcgactccccagacagcggccactaccct

tccacaaggaaggcaagcttcatgtcgagagtagtttctgagagcaagctgcccagtcga

tcaggaagcccggagtcaaggagtggttccagctcagaatccaagcgccttgggaaagga

gaaagtcctccgatcacaccccctccagcaggccggaggatgtcaatggcaagtagagcc

aaaagacgagccacttcagatctcactatatag

>Ec-gjb1-XM_028815444

atgaactgggcgtctttttatgccgttcttggcggcgtcaacaggcactccactgggatc

ggccgtatctggctttctgtcctcttcatcttccggatcatgatcttggttgtggcagca

gagactgtgtggggcgatgagaagtcaagctttacctgcaacacccagcagcctggctgt

aacagtgtctgttatgaccagttttttcccatctctcatgtgcgtctgtggtccctgcaa

cttatcctggtttccaccccagcgcttcttgttgctatgcacgtagctcaccggcgacac

atggataagaagattctccgactttcagggcgctccagcagcaaagacctggagcaaatc

aagaacaaaaaaatgaaaattactggagccttgtggtggacctacatgatcagtgtcatt

ttccgtgtgctctttgaagctgctttcatgtatatcttctacatgatctacccgggctac

aagatgtttcgactggtcaagtgcgattcttacccttgccccaacactgtcgactgcttt

gtgtcgcgacccactgaaaaaaccattttcacaatcttcatgctggtagtgtccggtgtc

tgcatcctcctcaatattgcagagattgtctacttgatttccaaggcttgtgcgagaagt

gtgagggatgcggagtccggccctttctatggtcagaagttctctggatacaaagaaggc

cagggtgaccagctcctctcggaccacgctgctctgagcaagatgaggaggaaccccggg

tccgataagtgggaaagatgctctccatcttag

>Ec-gjb3-XM_028819929-gjb3like

atggactggaagactttccaggccctccttagtggagtgaacaagtattcaacagctttt

ggacgcatctggttgtcagtcgtatttgtgttcagggttatggtgtacgttgttgccgct

gaacgagtgtggggagatgaacagaaagactttgattgcaacacccgacaacctgggtgc

agtaatgtttgctatgattttttctttcctatctcccacatccggttatgggcccttcag

cttatctttgtcacatgtccttcattgatggtagtcatgcatgtggcttacagggaagac

cgtgagaaaaagcacaagaaacttcatggtgaaaactgtggcaaactctacaaggacact

ggaaagaagcatggtggcctatggtggacctacttgatcagcttgtttgttaagctgggc

attgaaattgctttcctcttcatcttacactatatctacaacagctttgatctgccacga

ttggtcaaatgtgaggttaacccttgccccaaccaagttgattgctttattggtcgtcct

acagagaaaagagttttcacttactttatggtgggtgcctctgcattatgcattgtgcta

agcatttgtgaaatcatctatctgattacaaagagattcgttcgttgtgcccaaaagatg

aaaaaacgatcaaaagcgcactctgtgacctacagcaaagcttctacttgccagtgcaac

actggcatgaaccaaatggaactgaagcctttcaacaaaaacgataatttacgggcttca

gcacccaatctctctactctttag

>Ec-gjb7-XM_028797469

atgaactggggattcctcagagatctcctgagtggggtaaacaaatactccacagtgata

gggaggatctggctttctgtcgtgttcatttttcgcattctgatttatgtagctgcagca

gaacaagtctggaaggatgaacagaaggacttcgtgtgcaatacaaaccagccaggctgt

gagaatgtgtgctttgatcacttctacccaatttctcaagttagactctgggccttgcag

ctgataatggtgtccacaccgtccttgttagtggtgctgcatgttgcataccgacaacac

agagaaagtcggtataaaaagaagctatatacaaacacagggaagatggatggaggatta

tggtggacatatcttattagtttactattcaaaacaacctttgaagtgggctttctgtta

gcattttactatctatatagtggatttgaagtcccccagttgctccagtgtaaaattagt

ccttgtccaaacactgtagattgctacatttcacgagctacagaaaaaaaagtgtttctt

tacatcatgggtgtgacttccatcttatgcatcgtgctgaacatttctgaaatgatttat

ctttttcttaagcagtgctggaaatgctgtatcaaggcatatgattcagttgatgcaaag

agacattgtgattgtcagcagcacacaaagctaaacagtatgctttctggggatcatata

atgttgaaagaagactcaacaaatacaatctgtcagactcaagaaatatga

>Ec-gjb8.1-XM_028801319-gjb2like

atgagttggggacaactgtactctttgcttgggggtgtaaacaaacactcaaccagcttg

ggaaagatatggctctcagtcctctttatttttcgcatcatgatcctggcagtggcagct

gaaagtgtttggggggatgaacaaatggacttcatgtgcaacactttacaaccagggtgc

aagaatgtctgctatgatcattactttcccatctctcatattcgtctgtggtgcctccag

cttatctttgtgtctactccggctctgctagtagctatgcatgtggcccacagcaaaaag

gaacagaagaaggacatcattaagactatgggagataaggctgatgaggacctagagatg

ctcaagaaaaaaaggcttccaattacaggctcattatggtggacttacacagtaagcctc

tttttcaggctggcatttgaaggggctttcatttatgttttttacttgttgtacagtgga

ttccagatgccccgcctggtaaagtgtgatgcatggccatgtcctaatgtggtggattgc

ttcatttcacgtcccacagagaaaaccgtgtttaccattttcatgattagtgcctctgca

ctctgtatggtcctcaatgtggcagaaatgtgctatttggtggtcaaagccctagtacgc

tattgcaagtgctcccagcagggggtcagtgctcaccagctccccaatggtatggagtac

ctgcagaacaaggagaaggggcagctgctttctgaggcctcttctagcaaagacagttcc

atggacaagcttgcatag

>Ec-gjb8.2-XM_028800567-gjb2like 95% identical to XM_028801319. Although located on different contigs, it is considered likely that the two sequences are generated by gene duplication

atgagttggggacaactgtactctctacttgggggtgtaaacaaacactcaaccagcttg

ggaaagatatggctctctgtcctctttatttttcgtatcatgatcctggtggtagcagct

gaaagtgtttggggggatgagcagtcggacttcacgtgcaacactttacaaccagggtgc

aagaatgtctgctatgatcattactttcccatctctcatattcgtctgtggtgcctccag

cttatctttgtgtctactccggctctcctagtagctatgcatgtggcccacagcaaaaag

gaacagaagaaggacatcattaagactatgggagataaggctgatgaggacctagagatg

ctcaagaaaaaaaggcttccaattacaggctccttatggtggacttacactgtaagcctc

tttttcaggctggcatttgaaggggctttcatttatgttttttacttgttgtacagtgga

ttccagatgccccgcctggtaaagtgtgatgcatggccatgtcctaatgtggtggattgc

ttcatttcacgacccacagagaaaaccgtgtttaccattttcatgattagtgcctctgca

ctctgtatggtgctcaatgtggcagaaatgtgctatttggtggtcaaagccctagtacgc

tgttgcaggcgttcacagcaaaggtcttatcaccccagccatctgcccaaagggaaggag

cacctgcagaacaagaagaatgagcagctgctttccgaggcctcttctagcaaagacagc

tccatggacaagcttgcatag

>Ec-gjb9-XM_028819741-gjb4like

atgaattgggcatttctggaaggactaataagtggtgttaataagtactcaactggcttt

ggccgggtgtggcttacaatggtcttcatttttagagtcttggtgtttgtggtagcagct

caaaaagtatggggtgatgaaacaagtgattttatttgcaacaccaggcagccaggctgt

gagaatgtctgctatgatagtgtttttccgatctctcatatacgtatttgggctttgcaa

ctgatatttgtgacctgcccttctctgcttgttgtagcccatgtgaaatttcgggaagag

aaggacaaaaagtatgcagctgggcacaaaggctcccatctttacaacaaccctggaaaa

aagcatggtggactgtggtggacatatctcctgagtcttgtttttaaggcttgcttggat

gctatattcttgtacatcttatactacatctatgaaggctatgatctacctcggctcacc

aagtgttctcttccaccatgtcccaacactgtagactgcttcatttctcgccccacagag

aaaaggattttcactatgttcatggtggtcacatcggcaatttgtattgctatgtgcctt

gtagagattttctacttggtcagcagacgctgtgtgaagctttacaatcggcggcagatg

atgtacagcaagaccatagagtgcaaaaccagtcttcaaagctacaagctggagaatagc

tcagaaaatactaactcagagtgcctaagtgatactcctgtgtcaagacacaagaaaacc

tcagatcctaatacaactatataa

>Ec-gjb10-XM_028820016-gjb5like

atgaactgggctttcctgcaaggcctgctgagtggggtgaacaaatattcaaccgctttt

gggcgcatctggctctcggtggtattcatcttccggcttttggtgtatgtggtggcagca

gagcgtgtctggggagatgatcagaaggacttcacctgcaacacagctcagcccggctgc

cacaatgtctgctatgactacttcttccccgtctcccacatccgcctgtgggcattgcag

ctgatttttgtgacctgcccttcatttcttgttgtcatgcatgtggcctacagggaagaa

cgtgagaggagaaatcgggaaaaacatggagagaactgtggacgtctttacctggacaca

ggcaagaagagaggaggcctctggtggacctacttcttgagcctagtcttcaaggctgtg

gtagatgcagcctttctctacgtgctctattatatatatgaagcctacactttccctcgt

atggtgaagtgctctcagtttccatgcccaaatattgtggactgcttcatttccaggccc

acagaaaaacgcattttcacactgttcatggtgggcacctccattctctgcatcatcctg

agcattattgagatgttctacctgcttggcaagcgctgccgggactgtatagttgaacga

aagcggcggaatcaaatgaatgttgtgtctctgatgagcaacaaggaaaatggtaacatt

ttccaaagtcatagcttgttggtcccagcctataatcctaacatgcataagtag

>Ec-gjc1-XM_028818617

atgagctggagtttcctgacgcggctacttgaggagatccataaccactcgacctttgtg

ggcaagctgtggttcaccgtcctcatcatcttccgcatcgttctgacggtcgtcggcggc

gagtccatttactacgacgagcagagcaagtttgtgtgcaacacggcccagccgggctgc

gagaacgtctgctatgacgcctttgcccccctgtcgcatgtgcgcttctgggttttccag

atcatcctcatctcggcgccgtccgtcatgtacctgggctacgccgcccacaagattgcc

cgcatggaagaagaggcgggcggaaaggtgggcaccggctttggggcgggcagaaaacac

aagggcaagttcttcagccggaggaatcagcaccgaggcatccaggaggcagaagatgag

catgaggaagaccccatgatctaccaggaggcggaggtggagagccgcagtgacacaaac

gccgtgacggccgagagtccggcgccgggaaggccgcacgacaaggtccggcacgacggc

cggcggcgcatcaagaaggacggcctgatgaagatctacgtggcccagctgctgagccgt

gccaccttcgagctgggcttcctggtgggccagtatgccctgtacggctttagggtgccg

ccctcgtacatgtgtgagcgtgtgccctgccctcacacagtggactgcttcgtgtccagg

cccaccgagaagaccatcttccttctgatcatgtacgccgtcagcaccctctgcctcgtg

ctgaacgtctgtgagatgcttcatctgggcttcgggaccattggggacgtgctgcggagc

aggcagtgccccaccgaagagcagtacctgcaccctcctgcgggggacactgccacctac

acctaccccttcacatggaatgccccttcggcgccgccaggctacaacatcgtggtcaag

cccgagcaaatcccgtataccgacctgagcaacgccaagatggcctgcaagcagaacaag

gccaacatcgcccaggaagagcagcagcagcagcagtacagcagcaacgaggacaacttc

ccgatggcggacatgcgcgacgccctgcagaaggacatccgtgtggctcaggagcggctg

gaggctgccatccaggcctatggtcaccagaacctgcagccacgcggccaaagagatggc

aagaaggagcggggtggcacacgcccggtgccaggtcacacgggtgggggcagcagcagc

agtagcagccggtcagtgtcgggcaagccctcggtctggatttag

>Ec-gjc2-XM_028803887

atgagttggagctttctcactcgactactagaagaaatacacaaccattcaacatttgtg

ggaaaagtatggttgactgtactgataatatttcgtattgtactgacagctgttggagga

gaatctatctattctgatgaacaaaccaagtttatctgtaactctaaacaaccaggatgt

gacaatgtgtgctatgatgcatttgcaccactgtcccatgtcagattttgggtcttccaa

attattatgatatccacaccttctatcatgtatctgggttatgccattcacaagatttca

cgttcctctgaagaagagcgaacaaagttcaaaaattgtaacaaaaaacaacattctagc

caatggaggaatacacataatttggatgaattactagtagaagaagaagaacccatgatt

tatgaagatacactcgatgttcaggatgctaaacaagagagtaaagtccagattaaagaa

cagcagaaacatgatggacgacgtagaatcatgcaagaaggtcttatgagaatatatgta

atacaacttttagctcgagccatttttgaactaggtttccttggtggtcagtacctacta

tatggatttagagtaagcacttcctatgtctgctctaaaactccatgccctcatcaggta

gactgctttgtttcaaggcccacagagaagacaatctttcttttaattatgtatgtagtt

agctgtctttgtctcctgctaaacatttgtgagatgttccatttaggtattggcaccatc

agggatataatccacaatcgacaaaacaagtccaaacattactcttgcagctattcttat

cctagaaatattcctgcatctcctccaggatataacttagttgtgaagtcagataaaaac

agcaaaattcctaacagtttaataactcacgaacagaacatggccaatgttgctcaagag

cagtgttctaacagtcctgatgaaaatatcccatctgacctggtaagcctacataggcac

ctaaaggttgcccaagagcaacttgacatggccttccaaacctacaacagcaagagcaat

gcacagccttcaaggacaagcagtccagcatcaggagggactttagtggaacagaacaga

gttaatgcagcacatgaaaaacaaggtgctaggccgaaatcaagcatagaaaagccagaa

gctgttaaaaaagatggaaaaacatctgtgtggatttaa

>Ec-gjc4-XM_028807565-gjc1like

atgagctggagcttcttaacccgcttattggaagaaattcataaccattccacttttgtg

gggaagctatggctaaccatacttattgtttttcgcattgtattgacagccgttggagga

gagtctatctactatgatgaacagagcaagtttgtttgtaacacgcatcaacctggttgc

gagaatgtctgttatgatgcctttgcacccctttctcatgtgcgtttttgggtattccaa

ataattatggtatccacaccatcaatcatgtaccttggctttgcaatgcacaaaattgcc

agaacaggagatgtagaacagagcaaagtcaagaagcggatgccagtgatccagcgagga

gcaagtagagattatgaggaagcagaggacaatgatgaagaagatccaatgatatttgaa

gaaattgagcctgaaaaagatgataaaacttcaaaagcaactgaaaaaaagcatgatggg

cgtcgcaggataaagcaagatgggctaatgaaaatttatgtcctacagcttttgtctcgt

tcagtatttgaggtggctttcctttttggccaatatatattgtatggatttgaagtacct

ccatcttatatctgtagccgcagcccctgtcctcatattattgattgctttgtctcaagg

ccaacagagaaaactatatttcttcttattatgtatgttgtgagtggactttgtctactg

atggatatctgtgaaatgattcacctaggaatcggtggcattcgtgatgcacttcatagc

aaatctagaagacctccacctgggactaatatgcgatcatctgtgtctagacagctacca

tctgcacctccaggttaccacacaactctcaaaaagatgcccaaggctaaattatcaggt

ggacgcaagtccgagtttgctgataatcttggggattctggtcgtgaatcatttgcggat

gaaactgcttcaagagagctggacaggttacgcaaacacctaaaaatggcccaggaacac

ttggatatggctttccagctggaaattagcagtccatctcgtagcagcagtccagagtca

aatggtactgcagtagaacagaatcgtttaaactttgctcaggagaaagagggagcatct

tgtgaaaaaggaatcagagcataa

>Ec-gjd1-XM_028794147-gjd2like

atgggggaatggacaatattggagaggctgctggaggctgctgtacagcagcattcgacc

atgatcggccgaatcctgctgacagtggtggtaatcttccggatcctcattgtggccatt

gtaggagagacggtatatgaagatgagcagaccatgtttatgtgcaacaccttgcagccc

ggttgtaatcaggcctgctatgacaaagccttccccatctcccacatccgctactgggtc

ttccagatcatcctggtgtgcacccccagcctgtgcttcatcacctactcagtccatcag

tccgccaagcagcgtgatcgcagatattccttcctctatcctcttctcgaaaaggacttc

agtaggccagaaagcagcaaaaagctaaagaatgtaaatggcatcctggtgcaaaaccca

gatgggacagccaaggaggaacctgactgtttggaggtaaaagagatacccaacaccccc

atgaggaccagcaagagtaccaagttccatcgtcaggagggtatttccaggttctatatc

atccaggtggttttcagaaatgctctagagattggctttctggctggccagtactttctt

tatggattcaatgtacctgcaatctttgagtgtgaacgttatccttgcatgaaggaagta

gagtgctatgtgtcacggcccactgagaagacagtgtttctggtcttcatgtttgccgtc

agtggtatatgtgtggtcctcaatttggcagaactcaaccacctgggttggaggaagatc

aaaacagctatccgaggggtacaggcacggaggaaatctatctgtgaggtgcggaagaaa

gatgtctctcacttgacgcaggtccccaacttgggcagaactcagtccagtgagtcagca

tacgtttga

>Ec-gjd2-XM_028821539

atgggggaatggaccatcctggagcgactcttggaggctgcggtccagcagcactctacg

atgataggaaggatcctgttaacggtggtggtgatcttccggatcctaatcgtggcaata

gttggagagacggtctacgacgacgagcagaccatgttcgtgtgcaacaccctccagccg

ggctgtaaccaggcctgctacgacaaggctttccccatctcccacatgaggtactgggtc

ttccagatcatcatggtgtgcaccccgagcctttgcttcatcacgtactcggtgcaccag

tccgccaagcagaaggagcggaggtactccaccgtgttcctcactctggacaaggagcag

gactcgatgaagcgcgaagacagcaagaagatcaagaacaccatcgtcaacggcgtgctc

cagaacaccgaaaacctaaccaaagaaacggagcccgactgcctggaggtcaaggagatc

cccaactcagccctccgaacgaccaaatccaagatgcggcggcaggagggcatctcccgc

ttctacatcatccaggtggtgttccgaaacgccctggagatcggctttttggtcggccag

tacttcctctatggatttaacgtgccctcggtgtacgagtgtgtccggtacccttgcata

aaagacgtcgagtgctacgtctccaggcccacggagaagacggtctttctggtgttcatg

ttcgccgtgagcgggctctgcgtggtccttaatttggcagaactgaatcatttgggatgg

agaaagatcaaaatggcagtcaggggggtgcaggccagaaggaaatccatttacgaaatt

cgaaacaaggacctcccgagaatgagcgtgcctaattttggtcggactcagtccagcgat

tctgcctacgtctga

>Ec-gjd3-XM_028818825

atggcggagtggaactttctacaggagctcttcaggagcctccacaatgacatgacggct

ctgggccgcctttggctgctgcacgttatggtcttccgcctcctcctcgtgggcacggtg

actcccagtctctttgaaaacgaatacgcggagttcacctgcaacacccagatgactgga

tgtagagaggtgtgctttaacagggccaccaccatctcgcactatcgcttctgtgtcttc

catgttgtgctcctgtccactcccgtcatcctcttcgtcacgtatgctttgactaagagc

gcagtgcagcacggcgctgaggtggaagccttagctgggcaagagacgcagcgttcaagg

aggagactcgtctaccttatcaacgtcatcgtccgtctgctggtggaggttggcttcctc

gttggccagtggtacttatatgacttcagggtgaaatccatatacatctgcacggcacac

ccctgtccccacaatgtggactgtttcttggcacgctcctatgagaaaactgcatttctg

tgcttctactttggtgtcggggtgttctcttcggtgatcagtttcctcgaggtcttgtac

atcatccgcaagtggaaagcatccggcagggaacaagtggggcacaaccagtccaccctg

acccacagcacgccaggcgcagacgagctgcttaccatggagagccagacaaagacccct

caagaccagtga

>Ec-gjd4-XM_028803298 The first part of teh predicted sequebnce is here considered as a part of an intron.

atgacgtttagtgcaactcctaagagtaaccctgagccactgacaaatacaggcctagat

cttcatctaagtaacaaatttacttcagtgtttatggtaattgtcttggcgcatcaccca

acttctactgagcttcagtttgacgatactctgcccttgatgaacttgagaattcatttg

ccccttgaggatggtaagctgtggttaacgctgatggtgcttctgcggttcctcgttctc

cttctggctggctatcctttgtactgggatgagcaggaacgctttgtctgtaacacgatc

cagcccggctgtgccaatgtgtgttacgatgtgttctctcctgtgtcccacttcaggctt

tggttagttcaggtggtcatcctcagctttccatttgctcttttcactgtctatgtaatt

cacaaggcatctatgggtgcctcaatggaaattttcacatcgagcattgagaggtctcca

ttccaaaaagtccatcaggacattttccacaaggatttgtcacagccaggtgcagagatg

accgttcccagtttcttgggggcgtatgttttgcagctcctactacggattttcctagag

gtggcattcggagctggacattattacctctttggattctttattcccaggaggtacatc

tgctcagaagctccttgtaccagcacagtggactgctacatctctaggcccacagagaag

agcatcatggcaacctttatgctcggcgtcagctgcatttcttttctggtcagtattgta

gatctcctgtgtgtgattaaaagggcagtgaggcagaagcataaagagcggctgctttta

gaaaagctgtgcaaagaagagcagtattacctgactcctccgggccgagatgtggatgcc

catctacagctggctcaggagcacagtcacgtatgtcaagcagttgatgcaatgtcagtg

cagctgggagagttggaccaaatgtcaatccactcagcagctgggcctcacaggactgcc

acttccaacacaaatggcaacaatgcttatacacaggagcccgaggagcatgtggagcaa

gatgggagtgaagtagctttctgcatgaccgaggcagaggtggcattgccgccccagacg

ccaggccgacacggccgacgtggccgccacagggcaagtcgttcaagccgttgggagaaa

attcaaaacagccaagcagattcctctcctgatttgtcaagaccaaggcggatgggtcaa

tatgtgatggtggaaatgatgacctcagacgcacagtccatctctagcgaaaggagatcg

gagtgggtatga

>Ec-gjd5-XM_028808333-gjd2like

atgggtgattggtccattcttggccgcttcttaactgaggtgcagaaccattcaactgta

attgggaagatatggttaaccatgctgcttattttccgaattcttttggtcacattggtg

ggagatgcagtgtacagtgatgagcagtccaaattcacctgcaacacattacagcctggc

tgcaacaacgtgtgctatgatgcatttgcccccgtatcccacctgcgtttctgggtcttc

cagattgtcctggtctccacaccatccatcttctacattgtgtatgtcttgcataagatt

gcaaaagatgagaaaatggagatggagaaggcacgagttcatgagttgcttaatcatccc

tctcttgtggtggagccccatgggagtgaaagctttagccctcattctacctatgagact

gaatggagctgtgtagaacagactctgactggagatgaaattaagaaggtcaataaagat

cccacaaagttgcccagtacagttttggtaatatacatcatccatgtggtgttaaggtcg

ctgatggaagttgtttttttaataggccagtattatctgtttgggtttgcagtgccccac

ttgttccgatgtgagacttacccctgccccaccactactgactgctttgtttctagagcc

actgagaaaaccattttcctcaacttcatgtttgctgttagtttgggttgcttaattctc

aatgtagcagagttacactacctggggtggatttatattttccaaatgctctgttcggcc

tgctcgaagtgcttaaaaaatgagccaagtcaatattcacaggagaatccacttcttcaa

caggtcagagaaactctcctggaaaagctagacctcaagacctcttgtggcctatctcca

gagaagcctaactatgcactctcactgcctaccaccatctcctttgagagtgattccaca

gccgagtgtacctccaagaagagcccagatgaaaaggaacgggacaaatcaaagcttgtg

aatgtggccaagaaaattagaaaatcctggctgtag

>Ec-gjd6-XM_028823223-gjd2like

atgaccgagtggacggtgctcaaacggctccttgacgcggttcaccagcactcgacgatg

atcggtaggatctggctgaccgttatggtcatttttcgcttgttgatagtcgccgtggcc

acagaggacgtgtataccgacgagcaggacatgttcgtgtgcaatacgctccagccaggc

tgcgccaacctctgctacgactcgttcgcccccatatcccacgcgcgcttctgggtcttt

cagatcatcttggtgtccacgccatcgctgtgcttcatcgtctacacctggcacaacctg

tcaaaaataaacggggagtccgcgaaggaggagatctgcgcggtgcagcacggaggctgc

gggtccagccagggctcttgcaagtcccacggatatctggacgaggccggccggagtgga

cctggaggtgtccaaaggggccgccggaccacgtcgaggaggctcgtccggtgctatgtg

gtccacgtgtgctgccgcgccgccctggaggtcggcttcgtggccgcgcagtgcgcgctc

ttcggcttccaggtgcctcctcgcttcttgtgctcggcgccgccctgcgcgcaggcggtc

gactgctacgtgtcaagacccaccgagaagaccatcttcttaatgttcaccttctctgtc

ggcgtcctctgcagttgcctcaattttctcgaactcaatcatttaggatggaagaagatc

aaacggtcgctgctcacgcacgatttttctcaggggggctaccaggagatcaggagcccc

gacacgaactccgtggactccctcacactgaggggacatgcaaacgagtcgtcactcgca

tcttataacctggaggcggaacacaaccgggagtggacatttacgggggtttgttctcct

ggtggccagaagatggtcgactcgggcagaggggaggacagaagtcaggggggcgcctcg

tcattaaaggtgaagcctggaaagagcaaaggggggcgacagaagagcggtgaggtctgg

atctga

>Ec-gje1-XM_028820988 Splice sites

atgtctttaaactacattaagaatttttatgaaggatgtctcaggcctccaacagtgatt

ggtcagtttcataccctcttctttggctctgtgcgcatgttctttcttggggttctggga

tttgcagtttatggcaatgaggctttacacttcagctgtgatcctgataagagagagtta

aacctcttctgttacaaccaattcaggccgataacacctcaggtattttgggcactacag

ctggtcactgtgttggttcccggagctgtgtttcatctctatgctgcttgtaagaatatc

gatcaagaagaaatcctccaacgacccatctacactgtgttttatatcatttctgtactg

ttaagaatcattctggaagtcatagcgttttggcttcagagtcatctttttggctttcag

gttcacccaatttacatgtgcgacgccagcgccctggagaaaaagttcaacgtcaccaag

tgcatggtgcccgaacatttcgagaagaccatcttcctcagcgccatgtacacgttcact

gtcatcacggtggtgctgtgtgtcgccgagatatttgagattctttgcagaagactggga

tatttgacaagtcaatga

# Supplementary Figure S28. Sterlet sturgeon (*Acipenser ruthenus*) connexins.

The sequences were initially found by blasting GenBank wgs (organism: *Acipenser ruthenus*) with sequences from the other cartilaginous fishes. The found sequences became later supported by published predicted sequences in GenBank (XM numbers). The genomic positions for the sequences are found in Supplementary Table S5. The A/B ohnologs are selected from the following criteria: (i) All sequences on a specific chromosome are selected to be either A or B. (ii) The lowest chromosome number is selected to contain the A ohnolog, and the higher chromosome number is selected to contain the B ohnolog. The A ohnologs are used in the phylogenetic analyses, except for *gje1*, as for this ohnologous pair, it seems like the A ohnolog might be a pseudogene. To distinguish between the genome duplications that generated sturgeon ohnologs and the teleost ohnologs, the sturgeon ohnologs are indicated by upper-case "A/B" (in contrast to the lower-case "a/b" for teleosts).

>Ar-gja1A-XM_034004250-gja1

ATGGGTGACTGGAGTGCTCTAGGAAGACTTCTTGACAAAGTCCAGGCCTACTCAACAGCA

GGAGGAAAGGTGTGGCTTTCGGTTCTTTTCATCTTTCGAATCCTGGTCCTGGGGACGGCA

GTCGAGTCAGCCTGGGGGGATGAACAATCTGCTTTCAAGTGCAACACTCAGCAACCAGGT

TGTGAGAATGTCTGCTATGACAAATCATTCCCCATTTCCCATGTCCGCTTTTGGGTCATG

CAGATTATATTTGTCTCAACCCCAACACTACTTTACCTTGCTCACATTTTCTACCTTATG

CGAAAAGAGGAAAGATTGAATAAGAAAGAGGTGGAACTGAAAGCGGTCCAGAATGATGGT

GGCGACGTGGACCTGCCCCTCAAGCAGATTGAAATCAAGAAGTTTAAGTATGGTCTGCAG

GAGCATGGGAAGGTAAAGATGAAGGGTGCCCTCTTGCGCACCTACGTTGTGAGCATTATC

TTTAAATCATTCTTTGAAGTTGGTTTCCTGTTGATACAGTGGTACATTTATGGGTTTAGT

CTGGAGGCTGTATATACCTGCAAGAGGGATCCATGCCCACACCAAGTAGACTGTTTCCTC

TCTCGTCCTACTGAGAAGACCATCTTTATCATCTTCATGTTAGTGGTGTCTTTAGTGTCC

TTAGTGTTGAATGTGATTGAGTTATTCTATGTCTTTTTCAAAAGCATAAAGGATCGTGTG

AAGGGAAAAAGAGATCCTTACTCATCTAGTGGTGCAATGAGCCCGACATCTAAGGACTGT

ACCTCTCCAAAGTATGCCTTCTACAATGGCTGCTCCTCTCCCACCGCCCCCTTGTCTCCC

ATGTCTCCTCCTGGGTACAAGCTTGCCACTGGGGACAGGACCAACTCATGCCGCAACTAT

AACAAGCAAGCCACTGAGCAGAATTGGGCCAATTACAGCACCGAACAGAACCGCCTAGGA

CAAACTGGCAGCACCATCTCCAACTCTCACGCACAAGCGTTTGACTTCCCTGATGACACC

CATGATAATAAGAAACTAGCACCTGGACATGAGCTCCATCCTCTAGCTCTCATGGACCCC

AGGCCCTTGAGTAGAGCCAGCAGCCAAGTCAGCAGCAGAGCTAGACCAGATGACCTTGAG

GTCTAG

>Ar-gja1B-XM_034017373-cx43

ATGGGTGACTGGAATGCTCTAGGAAGACTTCTTGACAAAGTCCAGGCCTACTCAACTGCA

GGAGGAAAGGTGTGGCTTTCGGTTCTTTTCATCTTTCGAATCCTGGTCCTGGGGACGGCA

GTCGAGTCAGCCTGGGGGGATGAACAGTCTGCTTTCAAGTGCAACACTCAGCAACCAGGT

TGTGAGAATGTCTGCTATGACAAATCATTCCCCATTTCCCATGTCCGCTTCTGGGTCTTG

CAGATTATATTTGTCTCAACCCCAACACTACTTTACCTTGCTCACATTTTCTACCTTATG

CGGAAAGAGGAAAAATTGAATAAGAAAGAGGTGGAACTGAAAGCGGTCCAGAATGATGGT

GGCGACGTGGACCTGCCTCTCAAGCAGATTGAAATCAAGAAGTTTAAGTATGGTGTGCAG

GAGCATGGGAAGGTAAAGATGAAGGGTGCCCTCTTGCGCACCTACGTCGTGAGCATTATC

TTTAAATCATTCTTTGAAGTTGGTTTCCTGTTGATACAGTGGTACATTTATGGGTTTAGT

CTGGAGGCTGTATATACCTGCAGGAGGGATCCATGCCCACACCAAGTAGACTGTTTCCTC

TCTCGTCCTACTGAGAAGACCATCTTTATTATCTTCATGCTGGTGGTGTCCATAGTGTCA

TTAGTGTTGAATGTGATTGAGTTAGTCTATGTCTTTTTCAAAAGCATTAAGGATCGTATG

AAGGGAAAAGGAGATCCTTTCTCATCTAGTGGTGCAATGAGCCCAACATCTAAGGACTGT

GCCTCTTCAAAGTATGCCTTCTACAATGGCTGCACCTCTCCCACAGCCCCCTTGTCTCCC

ATGTCTCCTCCTGGGTACAAGCTTGCCACTGGGGACAGGACCAACTCATGCCGCAACTAC

AACAAGCAAGCCACTGAGCAGAATTGGGCCAATTACAGCACCGAACAGAACCGGCTTGGA

CAAACTGGCAGCACCATCTCCAACTCTCACGCGCAACCGTTTGACTTCCCTGATGATGCC

CATGATAATAAGAAACTAGCACCTGGACATGAGCTCCATCCTCTAGCTCTCATGGACCCC

AGGCCCTTGAGTAGAGCCAGCAGCCAAGTCAGCAGCAGAGTTCGACCGGACGACCTTGAG

GTGTAG

>Ar-gja2A-XM_034020768-gja3like

ATGGGAGACTGGAGCTTGCTGGGAAAGTTGCTGGAGAGCGCACAGGAGCACTCGACGGTG

GTGGGGAAGGTGTGGCTGACGGTGCTCTTCATCTTTCGCATCCTGGTTCTGGGAGCCGCT

GCCGAGAAGGTGTGGGGGGACGAGCAGTCGGGCTTCACCTGCGACACCAAGCAGCCCGGC

TGCCAGAACGTCTGCTACGACAAGACCTTCCCCATCTCGCACATCCGCTTCTGGGTGCTG

CAGATCATCTTCGTGTCCACCCCTACCCTCATCTACCTGGGGCACATCCTGCACCTGGTG

CGCATGGAGGAGAAGCAGAAGCAGAAGGAGAAGGAGAGGGCCGGCCGTGCCGCCCACCAC

GGGGACAAGCAGCCCCTGCTGGCAGAGCCCAAAGACAAGAAGCCGCCCGTGAGGGACGAG

CAGGGCAGGATCCGGATGCAAGGCGTCATCCTGCGGACCTACATTTTCAATATCATTTTC

AAAACCCTTTTTGAGGTGGGGTTTATAGTGGCTCAGTACTTCTTGTACGGGTTCCAGCTC

AAGCCCCTCTACACTTGTGACCGCTGGCCATGCCCAAACACCGTGAACTGCTACATCTCG

CGGCCCACCGAAAAGAGCATCTTTATTATTTTCATGCTCGCCGTGGCTTGCCTCTCGTTG

CTCCTCAACCTGGTGGAAATCTGCCACTTGGGTTTCACAAAATGCAGGCAAGGGTTGAAA

AACAGGACTGAGCTGGCTTGTGAAGCGGGGTCTAAAGCACCAAGCGAAGCAGCGGTGCCT

TTTGTGCCAAATTACCCCTACTTCTCCGGCCATCAGCCACCTCCCGGACCCTACCAGTCT

GGATCCCGATACAGCCCATCTCCCTTGACCGAAATGGACTCCACCTATCACCCGTACATC

AGCAAGGCTGCCTACAAGCAAAACAGAGACAACCTAGCCGTGGAGAGGAACGGCAAGCCA

GAGGAAAGAGACCTAAAAGTGAAGAAAGGGACTCAGCCACCGGTGGAGAAACAGCGCCGG

CCCAGTCGATCCAGCAAACACAGCAACAGCAAGACCCGGTTGGATGATCTCAAAATCTAA

>Ar-gja2B-XM_034051396-gja3like

ATGGGAGACTGGAGCTTGCTGGGAAAGTTGCTGGAGAGCGCACAGGAGCACTCGACGGTG

GTGGGGAAGGTGTGGCTGACGGTGCTCTTCATCTTTCGCATCCTGGTTCTGGGTGCCGCT

GCCGAGAAGGTGTGGGGGGACGAGCAGTCGGGCTTCACCTGCGACACCAAGCAGCCCGGC

TGCCAGAACGTCTGCTACGACAAGACCTTCCCCATCTCGCACATCCGCTTCTGGGTGCTG

CAGATCATCTTCGTGTCCACCCCTACCCTCATCTACCTGGGGCACATCCTGCACCTGGTG

CGCATGGAGGAGAAGCAGAAGCAGAAGGAGAAGGAGAGGGCCAGCCGTGCCGCCCACCAC

GGGGACAAGCAGCCCCTGCTGGCAGAGCCCAAAGACAAGAAGCCGCCCGTGAGGGACGAG

CAGGGCAGGATCCGGATGCAAGGCGTCATCCTGCGGACCTACATTTTCAATATCGTTTTC

AAAACCCTTTTTGAGGTGGGGTTTATAGTGGCTCAGTACTTCTTGTACGGGTTCCAGCTC

AAGCCCCTCTACACTTGCGACCGCTGGCCGTGCCCCAACACCGTGAACTGCTACATCTCG

CGGCCCACTGAAAAGAGCATCTTTATTATCTTCATGCTCGCCGTGGCTTGCCTCTCGTTG

CTCCTCAACCTGGTGGAAATCTGCCACTTGGGTTTCACAAAATGCAGGCAAGGGTTGAAA

AACAGGACTGAGCTGGCTTGTGAAGCGGGGTCTAAAGCACCAAGCGAAGCAGCGGTGCCT

TTTGTGCCAAATTACCCCTACTTCTCCGGCCATCAGCCACCTCCCGGACCCTACCAGTCT

GGATCCCGATACAGCCCGTCTCCCTTGACCAAAATGGACTCCACCTATCACCCGTACATC

AGCAAGGCTGCCTACAAGCAAAACAGAGACAACCTAGCCGTGGAGAGGAACGGCAAGCCA

GAGGAAAGAGACCTAAAAGTGAAGAAAGGGATTCAGCCACCGGTGGAGAAACAGCGCCGG

CCCAGTCGATCCAGCAAACACAGCAACAGCAAGACCCGGTTGGATGATCTCAAAATCTAA

>Ar-gja3A-XM_034012191-gja3

ATGGGTGACTGGAGCTTCCTGGGGAGACTTTTGGAAAATGCACAAGAACACTCCACGGTG

ATAGGCAAGGTCTGGCTGACAGTGCTGTTTATCTTCAGGATCTTAGTGCTGGGAGCAGCC

GCCGAAGAAGTCTGGGGAGACGAACAGTCTGATTTCACATGCAACACCCAGCAGCCTGGT

TGTGAAAACGTCTGCTATGACAAGGCCTTCCCAATCTCCCACATCCGCTTCTGGGTGCTT

CAGATCATCTTTGTGTCGACGCCCACCCTCATCTACCTGGGGCATGTCCTGCACATCGTG

CGGATGGAGGAGAAGAGGAAAGAGAAGGAGGAAGCGCTGCGGAAGTCTAGCAGACATCAA

GAGGAGAAGGACCTCTTCAAAAATGGCAGCGGTGGGAAAAAAGAGAAACCACCTATCAGA

GATGAGCATGGCAAAATACAAATCAGGGGCACCCTCCTGCGCACCTACGTTTTCAACATC

ATTTTCAAGACCTTGTTTGAAGTTGGATTTATTGTAGGTCAATATTTTCTTTATGGTTTT

CAACTGAAGCCCCTATACAGGTGTGGCAGGTGGCCGTGCCCCAATACAGTGGACTGCTTT

ATCTCTCGACCCACAGAAAAGACAATTTTCATCATATTTATGCTTGTGGTGGCTTGCGTG

TCTCTTTTGCTGAATTTGTTAGAGATTTATCACCTAGGATGGAAGAAGCTCAAACAGGGA

ATGATCAAGGAGTACTCCCCAAGTCACGTACCACCATGCAACAAAACAGAGCCTGAACCC

ATGACCCCCACCCCTAGAACTGCCCCACCCAGTCTTAGCTACCCACTTTATTACACAGAC

ACCACTGCAAGGGGTCCCCATGCTTACCCCGCAGCTCCCCCGGCAGAGTTCAAGATGTCA

CCTCTTACTGAGGAACATCACCCATCCCCTCCCTTCTACATCAATAGCAACCATAGGCTG

GCCACTGAGCAGAACTGGGCAAACCTCGCCACAGAGCAGCAGACTCGTGAGAGGAAACCT

GCCTCCCCTTCTGCCTCCACCACTAGCAGCGTCCGCAACGAGCACAGCAGCAAGGACGCA

CTGGCCAGCAGCAAGAGCTTGAGCGCTGGGAAAAGTGAGCAGGAAGAGGGGCATGTGACC

ACCACAGTAGAAATGCATGAGCCCCCTGCTGTCAGTGACACGCGACGATTGAGCAGGGCG

AGTAAGTCTAGCAGCAGTAGAACAAGATCAGATGACCTAGCGGTCTAG

>Ar-gja3B-XM_034923915-gja3like

ATGGGTGACTGGAGCTTCTTGGGGAGACTTTTGGAAAATGCACAGGAACACTCCACGGTG

ATAGGCAAGGTCTGGCTGACAGTGCTCTTTATCTTCAGGATCTTACTGCTGGGAGCAGCC

GCCGAAGAAGTCTGGGGAGATGAACAGTCTGATTTCACATGCAACACCCAGCAACCTGGT

TGTGAAAACGTCTGCTATGACAAGGCCTTCCCAATCTCCCACATCCGCTTCTGGGTGCTT

CAGATCATCTTTGTGTCGACGCCCACCCTCATCTACCTGGGGCATGTCCTGCACATCGTG

CGGATGGAGGAGAAGAGGAAAGAGAAGGAGGAAGAGCTGCGGAAGGAGCTCTTCAAAGAT

GGCGGCGGTGGGAAAAAAGAGAAAGCACCTATCAGAGATGAGCATGGCAAAATACGAATC

AGGGGCGCCCTCCTGCGCACCTACGTTTTCAACATCATTTTCAAGACCTTGTTTGAAGTT

GGATTTATTGTAGGTCAGTATTTTCTTTATGGTTTTCAACTGAAGCCCCTATACAGGTGT

GACAGATGGCCGTGCCCCAACACAGTGGACTGTTTTATCTCTCGACCCACAGAAAAGACA

ATTTTCATCATATTTATGCTTGTGGTGGCTTGCGTGTCTCTTTTGCTGAATTTGTTAGAG

ATTTATCACCTAGGATGGAAGAAGCTCAAACAGGGAATGATCAAGGAGTGCTCCCCAAGC

CACGTACCACCGTGCAACAAAACAGAGCCTGAACCCATGACTCCCACCCCTAGAACTGCC

CCACCCAGTCTTAGCTACCCACCTTATTACACAGACACCACTGCGGGGGGTCCCCATGCT

TACCCCGCAGCTCCCCCGGCAGAGTTCAAGATGTCACCTCTTACTGAGGATCAGCACCCA

TCCCCTCCCTTCTACATCAACAGCAACCATAGGCTGGCCACTGAGCAGAACTGGGCAAAC

CTCGCCACAGAGCAGCAGACTTGTGAGAGGAAACCCGCCTCCTCTTCTGCCTCCACCAAT

AGCAGCGTCCGCAACGAACGCAGCAGCAAGGACGCACTGGCCAGCAGCAGTGCTTGGAAA

AGTGAGCAGGAAGAGGGTCATGTGACCACCACAGTAGAAATGCATGAGCCCCCTGCTGTC

AGTGACACACAACGATTGAGCAGGGCGAGTAAGTCTAGCAGCAGTAGAACAAGATCAGAT

GACCTAGCGGTCTAG

>Ar-gja3C-Not predicted

ATGGGTGACTGGAGCTTCTTGGGGAGACTTTTGGAAAATGCACAGGAACACTCCACGGTG

ATAGGCAAGGTCTGGCTGACAGTGCTCTTTATCTTCAGGATCTTACTGCTGGGAGCAGCC

GCCGAAGAAGTCTGGGGAGATGAACAGTCTGATTTCACATGCAACACCCAGCAACCTGGT

TGTGAAAACGTCTGCTATGACAAGGCCTTCCCAATCTCCCACATCCGCTTCTGGGTGCTT

CAGATCATCTTTGTGTCGACGCCCACCCTCATCTACCTGGGGCATGTCCTGCACATCGTG

CGGATGGAGGAGGAGAGGAGGAAAGAGAAGGAGGAAGAGCTGCGGAAGGAGCTCTTCAAA

GATGGCGGCGGTGGGAAAAAAGAGAAAGCACCTATCAGAGATGAGCATGGCAAAATACGA

ATCAGGGGCGCCCTCCTGCGCACCTACGTTTTCAACATCATTTTCAAGACCTTGTTTGAA

GTTGGATTTATTGTAGGTCAGTATTTTCTTTATGGTTTTCAACTGAAGCCCCTATACAGG

TGTGACAGATGGCCGTGCCCCAACACAGTGGACTGTTTTATCTCTCTCGACCCACAGAAA

AGACAATTTTCATCATATTTATGCTTGTGGTGGCTTGCGTGTCTCTTTTGCTGAATTTGT

TAGAGATTTATCACCTAGGATGGAAGAAGCTCAAACAGGGAATGATCAAGGAGTGCTCCC

CAAGCCACGTACCACCGTGCAACAAAACAGAGCCTGAACCCATGACTCCCACCCCTAGAA

CTGCCCCACCCAGTCTTAGCTACCCACCTTATTACACAGACACCACTGCGGGGGGTCCCC

ATGCTTACCCCGCAGCTCCCCCGGCAGAGTTCAAGATGTCACCTCTTACTGAGGATCAGC

ACCCATCCCCTCCCTTCTACATCAACAGCAACCATAGGCTGGCCACTGAGCAGAACTGGG

CAAACCTCGCCACAGAGCAGCAGACTTGTGAGAGGAAACCCGCCTCCTCTTCTGCCTCCA

CCAATAGCAGCGTCCGCAACGAACGCAGCAGCAAGGACGCACTGGCCAGCAGCAGTGCTT

GGAAAAGTGAGCAGGAAGAGGGTCATGTGACCACCACAGTAGAAATGCATGAGCCCCCTG

CTGTCAGTGACACACAACGATTGAGCAGGGCGAGTAAGTCTAGCAGCAGTAGAACAAGAT

CGGATGACCTAGCGGTCTAG

>Ar-gja4A-XM_034023067-gja4like

ATGGCGGACTGGAGCTTGCTGGAGAAGCTGCTGGGGGAGGTGCAGGAGCACTCCACGGGG

CTGGGGCAGGCCTGGCTCACCGTCCTGTTTGTGTTCCGCGTGCTGGTGCTGTGCACGGCC

GCCGAGTCTGCCTGGGACGACGAGCAGGCTGACTTCACCTGCAACACCCTGCAGCCAGGC

TGCCAGCCAGTCTGCTACGACCGCTGCTTCCCCATCTCCCACTTCCGCTACTTCGTGCTG

CAGATCATCTTCGTGTCCACTCCCACCCTGCTCTACCTGGGGCACGTGGCCTGGAGAGCG

GGCCGGGCGAGGAAGAGGGGGGAGGAGGAGGAGGAGGGGGGAGCCGAGCTCAAAGGGGTG

CTGATGTTCCCCTACACCCTGAGCGTCGTCTGCAAGGTGATCTTGGAAACAGGCTTCCTC

CTGGGGCTCTGGTTCCTCTACGGCTTCACCATCCAGCCCCTCTACGTGTGCCAGGGCTTC

CCCTGCCCTCACCAGGTGGACTGCTATGTGTCGCGCCCCACCGAGAAGACGGTCTTCACC

ATCTACATGCAGGGCATTGCCGTGGTGTCCCTCCTCCTCAACCTGATCGAGCTCCTCCAC

CTCCTGGCTGGGGGGCTCATGAGGAAGGCTCGCAGGCTCTACTGGGGGGGCAGGCGGGCC

GAGGAGCCAACGCTCAAGCCAGGGCTCCCCGAGAACGGATTCGTCTGCCTGCCCGCCAGC

GGACCCCCCGCCGGTCCCCCACAGACCTGGGCTTGCCAGGCAGAGCAAAGGACCGCAGCC

CCCCAGTTTGACCAGCTGCCCCAGTACGGGGCCTGCGTCGGTCCAGTGAAGGCAGCTACC

TCTAATAAGCCTGCAGATCGAAGGTCAGCAAGCAGGGGCTCCGAGGCAGAGAAGAAGCAG

TATGTTTAA

>Ar-gja4B-XM_034022433-gja4like

ATGGCGGACTGGAGCTTGCTGGAGAAGCTGCTGGGGGAGGTGCAGGAGCACTCCACGGGG

CTGGGGCAGGCCTGGCTCATCGTCCTGTTTGTGTTCCGCGTGCTGGTGCTGTGCACGGCC

GCCGAGTCTGCCTGGGACGACGAGCAGGCTGACTTCACCTGCAACACCCTGCAGCCAGGC

TGCCAGCCAGTCTGCTACGACCGCTGCTTCCCCATCTCCCACTTCCGCTACTTCGTGCTG

CAGATCATCTTCGTGTCCACTCCCACCCTGCTCTACCTGGGGCACGTGGCCTGGAGAGCG

GGCCGGGCGAGGAAGAGGGGGGAGGAGGAGGAGGAGGGGGGAGCCGAGCTCAAAGGGGTG

CTGATGTTCCCCTACACCCTGAGCGTCGTCTGCAAGGTGATCTTGGAAACAGGCTTCCTC

CTGGGGCTCTGGTTCCTCTACGGCTTCACCATCCAGCCCCTCTACGTGTGCCAGGGCTTC

CCCTGCCCTCACCAGGTGGACTGCTACGTGTCGCGCCCCACCGAGAAGACGGTCTTCACC

ATCTACATGCAGGGCATCGCCGTGGTGTCCCTCCTCCTCAACCTGATCGAGCTGCTCCAC

CTCCTGGCCGGGGGGCTCATGAGGAAGGCTCGCAGGCTCTACTGGGGGGGCAGGCGGGCC

GAGGAGCCAACGCTCAAGCCAGGGCTCCCCAAGAACAGATTCGTCTGCCTGCCCGCCAGC

GGACCCCCCGCCGGTCCCCCGCAGACCTGGGCTTGCCAGGCAGAGCAAAGGACTGCAGCC

CCCCAGTTTGACCAGCTGCCCCAGTACGGGGCCTGCGTCAGTCCAGTGAAGGCAGCTACC

TCTAATAAGCCTGCAGATCGAAGATCAGCAAGCAGGGGCTCCGAGGCAGAGAAGAAGCAG

TATGTTTAA

>Ar-gja5A-XM_034011119-gja5like

ATGGGTGACTGGAGTCTTTTAGGAAACATCCTTGAAGAAGTACAGGAGCACTCAACGATA

GTGGGAAAGGTCTGGCTCACTGTGGTCTTTATTTTCCGGATCCTTGTCCTGGGCACTGCA

GCTGAGTCCTCCTGGGGAGATGAGCACAGCGACTTTGAATGCGATACCCTGCAGCCTGGT

TGCACGAATGTGTGCTACGACAAGGCTTTCCCCATCTCACACATCCGCTATTGGGTTCTT

CAGATTATATTTGTTTCAACCCCCTCCCTCATCTACATGGGACATGCTATGCATACAGTC

CGCATGGAGGAGAAAAAGAAACTCAGAGAGCAAGAAGACAGGGCCCAAGGTGGCAAAGAG

TATCTAGAAGACAAGGTGGACGTATCAATGAAAGAAGAAGCCACTGTCAAGATCAAGCTC

AAGGGGACTTTACTCCAAACATACGTCTTCAGTATCCTCATCAGGACGGTTATTGAGGTG

GCTTTCATTGTGGGTCAGTATCTTATTTACGGGATCTTCCTGAAACCCCTCTACCACTGC

GATAATTGGCCTTGCCCTAATCCTGTGAACTGCTACATGTCCAGGCCAACTGAGAAGAAC

GTCTTCATAGTGTTCATGCTTACTGTGGCGGTGTTCTCACTTTTTCTGAGTGTGGTGGAG

CTTTACCATCTTGGATGGAAGAAGTTCAAGCAGTGTTTGAGAACCTACAACTCCACCCCT

AACCCTGTTGCAGCTGCCCCCATCTCCAAGGAACCTGACGCACCAGACAGACCCTCCCAG

GCTTGCACACCTACACCAGATTTCAACCGGTGTTTGGTGGCGACCGATGGCCGATGCAAC

CCTTTCAATAACAAGCTGGCCCACCAGCAGAACTCTGCCAATATGGCCACAGAGCGTCAC

CATAGTCGTGACAACTTGGAGGGTGAAGATTTTCTCCAAATGCACTACCTGCAAGGAAGT

GAGACCTCCAATAGTTCAGCTGCCCCTCCTGGGCATGAGAACCTTTTTAAGGATAAACGT

AGACTCAGCAAGGCCAGCGGGTCCAGTAGTAGAGTGAGATCTGATGACCTAGCAGTATAA

>Ar-gja5B-gja5like

ATGGGTGACTGGAGTCTTTTGGGAAACATCCTTGAAGAAGTACAGGAGCACTCAACGATA

GTGGGAAAGGTCTGGCTCACTGTGGTCTTTATTTTCCGTATCCTTGTCCTGGGCACTGCA

GCCGAGTCCTCCTGGGGAGATGAGCACAGCGACTTTGAATGCGATACCCTGCAGCCTGGT

TGCACCAATGTGTGCTACGACAAGGCTTTCCCCATCTCACACATCCGCTATTGGGTTCTT

CAGATTATATTTGTTTCAACCCCCTCCCTCATCTACATGGGACATGCTATGCATACAGTC

CGCATGGAGGAGAAAAAGAGACTCAGAGAGCATGAAGACAGGGCCCAAGGTGGCAAAGAG

TATCTAGAAGACAAGGTGGACGTATCAATGAAAGAAGAAGCCACTGTCAAGATCAAGCTC

AAGGGGACTTTACTCCAGACATATGTCTTCAGTATCCTCATCAGGACTGTTATTGAGGTG

GCTTTCATTGTGGGTCAGTATCTTATTTACGGGATCTTCCTGAAACCCCTCTACCACTGC

CGTAATTGGCCTTGTCCTAATCCTGTGAACTGCTTCATGTCCAGGCCAACTGAGAAGAAC

GTCTTCATAGTGTTCATGCTTACTGTGGCGGTCTTCTCACTGTTTCTGAGTGTGGTGGAG

CTTTACCATCTTGGATGGAAGAAGTTCAAGCAGTGTTTGAGAACCTACAACTCCTCCCCT

AACCCTGTTGCAACTGCCCCCATCTCCAAGGAACCTGACGCACCAGCCAGACCCTCTCAG

GCTTGCACACCTACACCAGATTTCAACCAGTGTTTGGTGGCGACCAATGGCCATTGCAAC

CCTTTCAATAACAAGCTGGCCCACCAGCAGAACTCTGCCAATATGGCCACAGAGCGTCAC

CATAGTCGTGACAACTTGGAAGGCAAAGATTTTCTCCAAATGCACTACCTGCAAGGAAGT

GAGACCTCCAATAGTTCAGCTGCCCCTCCTGGGCATGAGAACCTTTTTAAGGATAAACGT

AGACTCAGCAAGGCCAGCGGGTCCAGTAGTAGGGTGAGATCTGATGACCTAGCAGTATAA

>Ar-gja8A-XM_034010978-gja8like

ATGGGTGACTGGAGTTTTTTGGGAAACATCTTAGAAGAAGTAAATGAACATTCCACTGTG

ATAGGAAGAGTATGGCTGACAGTGCTCTTCATTTTTAGGATTTTAATCCTTGGCACCGCT

GCCGAGTTTGTCTGGGGAGATGAACAGTCAGACTTTGTTTGCAACACCCAGCAGCCTGGT

TGCGAGAATGTCTGCTATGACGAGGCTTTCCCCCTTTCTCATATCAGACTTTGGGTTCTC

CAAATCATATTTGTCTCCACACCATCACTGGTTTATGTGGGCCATGCTGTCCATTATGTG

AGGATGGAAGAAAAGAGGAAGGAGAGGGAGGAAGCTGAAATGAACAGGCAGCAGGAGATG

AACGAGGAGCGGTTGCCTCTGGCTCCAGATCAAGGGAGTGTGAGAACCACCAAGGAAACC

AGCATGAAAGGCAACAAGAAGTTCAGGTTGGAAGGAACATTGCTGAGGACCTACATATGC

CATATCATCTTCAAAACCATCTTCGAGGTTGGCTTTGTGGTAGGACAGTACTTTCTCTAT

GGCTTCCACATTTTCCCTCTCTACAGATGCAGCCGGTGGCCCTGTCCCAATACAGTCGAC

TGCTTTGTTTCCAGGCCGACGGAGAAGACGGTGTTCGTCATCTTCATGTTGGCAGTGGCT

TCGGTGTCACTCTTTCTCAACTTTGTGGAGATCAGTCACCTAGGAATGAAGAAAATTAGG

TTTGCCTTTAGGAAGCACCCCCAGCAGCAAGGGGAGTTGGTTTCTGAGAAGCCTTTGCAC

TCCATTGCTATTTCTTCTATCCAGAAAGCCAAGGGTTACAAGCTCCTCGAGGAAGACAAG

GCTGTGTCTCATTTCTATCCTTTGACAGAAGTCGGGATGGAAGCCAGTAGCCTGCCAGCA

CCATTTACAACTTTTGAAGAGAAGAGTAATGATGCAGGTCTTGTGCAAGATGTTTCCAAG

GTCTATGATGAAACATTACCATCCTATGTTCAGACTACCGAGCAAGAAGAAATTCTAGAA

GAACCACCCGCAGAAGCCACTGAGACGATAGAAGATACAAGACCTTTGAGCAGTTTAAGC

AAAGCAAGCAGCAGGGCCAGGTCAGATGATTTAACAGTATGA

>Ar-gja8B-XM_034009780-gja8like

ATGGGTGACTGGAGTTTTTTGGGAAACATCTTAGAAGAAGTAAATGAACATTCCACTGTG

ATAGGAAGAGTATGGCTGACAGTGCTCTTCATTTTTAGGATTTTAATCCTTGGCACCGCT

GCCGAGTTTGTCTGGGGAGATGAACAGTCAGACTTTGTTTGCAACACCCAGCAGCCTGGT

TGCGAGAATGTCTGCTATGACGAGGCTTTCCCCCTTTCTCATATCAGACTTTGGGTTCTC

CAAATCATATTTGTCTCCACACCATCACTGGTTTATGTGGGCCATGCTGTCCATTATGTG

AGGATGGAAGAAAAGAGGAAGGAGAGGGAGGAAGTTGAGATGACCAGGCAGCAGGAGATG

AACGAGGAGCGGTTGCCTCTGGCTCCAGATCAAGGGAGTGTGAGAACCACCAAGGAAACC

AGCATGAAAGGCAACAAGAAGTTCAGGTTGGAAGGAACATTGCTGAGGACCTACATATGC

CACATCATCTTCAAAACCATCTTCGAGGTTGGCTTTGTGGTGGGACAGTACTTTCTCTAT

GGCTTCCGCATCTTCCCTCTGTACAGATGCAGCCGGTGGCCCTGTCCCAATACAGTCGAT

TGCTTTGTTTCCAGGCCGACGGAGAAGACGGTGTTTGTCATCTTCATGTTGGCAGTGGCT

TCGGTGTCACTCTTTCTCAACTTTGTGGAGATCAGTCACCTAGGAATGAAGAAAATTAGG

TTTGCCTTTAGGAAGCACCCCCAGCAGCAAGGGGAGCTGGTTTCTGAGAAGCCTTTGCAC

TCCATTGCTATTTCTTCTATCCAGAAAGCCAAGGGTTACAAGCTCCTCGAGGAAGACAAG

GCTGTGTCTCATTTCTATCCTTTGACAGAAGTCGGGATGGAAACCAGTAACCTGCCAGCA

CCATTTGCAACTTTTGAAGAGAAGAGTAATGACGCAGGTCTTGTGCAAGATGTTTCCAAG

GTCTATGATGAAACATTACCATCCTATGTTCAGACTACCGAGCAAGAAGAAGAAATTCAA

GAGGAACCACCCGCAGAAGCCACTGAGACGATAGAAGATACAAGACCTTTGAGCAGTTTA

AGCAAAGCAAGCAGCAGGGCCAGGTCAGATGATTTAACAGTATGA

>Ar-gja9A-XM_034023019-gja9like

ATGGGTGATTGGAACTTCCTGGGAGGGATTTTGGAGGAGGTGCACATCCACTCTACCATC

GTCGGGAAGATCTGGCTGACAATCCTCTTCATCTTCCGCATGCTGGTCCTCGGGGTGGCA

GCTGAAGATGTATGGAACGACGAGCAGGCCGAGTTTATCTGCAACACGGCTCAGCCTGGG

TGCAGGAACGTCTGCTACGACAAGGCTTTCCCCATCTCACTCATACGCTACTGGGTCCTG

CAAGTCATCTTTGTCTCCTCACCCTCCTTGGTGTACATGGGCCATGCTTTGTACAGACTG

AGGGCCCTGGAGAAGGAAAGACAAAAAAAGAAGGCACAACTACGAAGAGAGCTGGCTGTG

GACATAGACCTGGTCGAAGTTCGAAAGAAACTGGAGCGAGAACTTAGACAACTGGAGCAA

AGGAAGCTGAACAAAGCCCCGCTCAGAGGATCTCTACTCCGCACGTACGTGATGCACATC

GTTACGAGGTCTGGGGTCGAAGTTGGATTCATGGTCGGGCAATACTTCCTCTATGGCTTC

CAGCTTGACCCACTCTTTAAATGTGAGAGGGAACCGTGTCCAGAAGTAGTCGACTGTTTT

GTGTCACGGCCAACTGAAAAGACAGTGTTCATGATGTTTATGCAGTGCATAGCAGCACTC

TCGTTATTCCTCAACATTCTTGAAGTCATCCATTTAGGATACAAGAAGCTAAAAAATGGA

ATTTTAGACTATTATCCAAACCTTAGAGATGATCTGGATGATTACGACATGAGCAAATCT

AAGAAGAACTCTGTGATCCAACAGGTTTGCATCGGGATGTCTACGGGCCATAAAGCCACC

ATCCCCACGGCACCCAGTGGGTACACCCTACTGATGGAGAAGCAAGGAGATGCACCGCTT

TACCCCATCCTGAACCCGTCTTCTGCTTTCATGCCCATTCAGGGTGATCCAAATGGGAAC

ACAAAGAGCTGTCCAGAAAGCCACAAGGAGAGCTCAAATCAGAGCGCTGGGGAACAGAAC

AGCAGCTCAAACAATACAGTGCTAAACAAGTCCCCCAAGGCCAACAGTTTGCCAAAGCAG

GGGGAGCAAGATGATCATTCCCACAGCACAGCCCGCCATTCTGGTGGGGATCGTCTTTCT

GAAGGTCCTGGGGAATCTGGATCCTCCTTGTACCCTGCGCTCCAGGGGGAGTTGTCATCC

TGCCCGACAATGCAGGTCAGTTCCAGCCGGAAGCCGCGACGCGTCAGTGCTCCGTGGAAC

TGCACAACGGTGGTGGAAAGCAACGGATCCAACGGTGACGGGGCTGTCAGCAGCAGCCAC

CAGGGAAGATGCAGCTTCAGTGCCACTAAGGCTAGGGCGGCCTCTAAGTCTGACCTCAAA

CGACTCAGCCGGCCAGATACCCCGGACTCAATTGGGGAATCAAGCTCAGAGTCGAAGCAG

AGTCGTAATTGTGACAGCCCACAGGCCCTCTCCCCCGCTCGGCGAATGTCATTGGCAAGT

AACGCCAGCAGCAGGCGCGCCCCCACCGATCTTCAGATCTAA

>Ar-gja9B-XM_034022526-gja9like

ATGGGTGATTGGAACTTCCTGGGAGGGATTTTGGAGGAGGTGCACATCCACTCCACCATC

GTCGGGAAGATCTGGCTGACGATCCTCTTCATCTTCCGCATGCTGGTCCTCGGGGTGGCA

GCTGAAGACGTGTGGAACGACGAGCAGGCCGAGTTTATCTGCAACACGGCTCAGCCTGGG

TGCAGGAACGTCTGCTACGACAAGGCTTTCCCTATCTCACTCATACGCTACTGGGTCCTG

CAGGTCATCTTTGTCTCCTCACCCTCCCTAGTGTACATGGGCCATGCTTTGTACAGACTG

AGGGCCCTGGAGAAGCAAAGACAAAAAAAGAAGGCACAACTACGAAGAGAGCTGGCTGTG

GACATAGACCTGGTAGAAGTTCGAAAGAAAATGGAGCGAGAACTTAGACAACTGGAGCAA

AGGAAGCTGAACAAAGCCCCGCTCAGAGGATCTCTACTCCGCACGTACGTGATGCACATC

GTTACGAGGTCTGGGGTCGAAGTTGGATTCATGGTCGGGCAATACTTCCTCTATGGCTTC

CAGCTTGACCCACTCTTTAAATGTGAGAGGGAACCGTGTCCAGAAGTAGTCGACTGTTTT

GTGTCACGGCCAACTGAAAAGACAGTGTTCATGATGTTTATGCAGTGCATAGCAGCGCTC

TCGTTATTCCTCAACATTCTTGAAGTCATCCATTTAGGATACAAGAAGCTAAAAAATGGA

ATTTTAGACTATTATCCAAACCTTAGAGATGATCTGGATGATTACGACGTGAGCAAATCT

AAGAAGAACTCTGTGATCCAACAGGTTTGCATCGGGATGTCTACGGGCCGTAAAGCCACC

ATCCCCACGGCACCCAGTGGGTACACCCTACTGATGGAGAAGCAAGGAGATGCACCGCTT

TACCCCATCCTGAACCTGTCTTCTGCTTTCATGCCCATTCAGGGTGATCCAAATGGGAAC

ACAAAGAGCTGTCCAGAAAGCCACAAGGAGAGCTCAAATCAGAGCGCTGTGGAACAGAAC

AGCAACTCAAACAATACAGTGCTAAACAAGTCCCCCAAGGCCAACAGTCTGCCAAAGCAG

GGGGAGCAAGATGATCATTCCCACAGCACAGCCCGCCATTCTGGTGGGGATCGTCTTTCT

GAAGGTCCTGGGGAATCTGGATCCTCCTTGTACCCTGCGCTCCAGGGGGAGTTGTCATCC

TGCCCGACAATGCAGGTCAGTTCCAGCCGGAAGCCGCGACGCGTCAGTGCTCCGTGGAAC

TGCACAATGGTGGTGGAAAGCAACGGATCCAACGGTGACGGGGCTGTCAGCAGCAGCCAC

CAGGGAAGATGCAGCTTCAGTGCCACTAAGGCTAGGGCGGCCTCTAAGTCTGACCTCAAA

CGACTCAGCCGGCCAGATACCCCGGACTCAATTGGGGAATCAAGCTCAGAGTCGAAGCAG

AGCCGTAATTGTGACAGCCCACAGGCCCTCTCCCCCGCTCGGCGAATGTCATTGGCAAGT

AACGCCAGCAGCAGGCGCGCCCCCACCGATCTTCAGATCTAA

>Ar-gja10-XM_034004988-cx52.6 An intron is probably starting before the indicated repetitive sequence

ATGGGTGACTGGAACTTGCTTGGAAGCATCTTGGAAGAGGTCCACATTCACTCCACCATA

GTGGGGAAAATTTGGCTCACAATCCTCTTCATTTTCCGAATGCTGATACTGGGAGTTGCT

GCCGAAGATGTCTGGGATGACGAGCAGGTTGAGTTTATTTGTAACACAGAGCAGCCCGGC

TGCAGGAATGTTTGCTATGACAAAGCCTTCCCAATCTCCCTGATCCGATACTGGGTCTTA

CAAATCATTTTTGTCTCTTCGCCTTCCTTGGTGTACATGGGCCATGCACTTTACAGGTTA

AGGGCTTTAGAGAAGGAAAGACAGAAAAAGAAGGCCCAACTGAGAGCGGAACTAGAAGGG

ATTGAGCTGATACTCGAAGAATACAAGAGGATGGAGAGGGAGCTGAGAAAGCTGGAGGAG

CAGAAGAAAGTGAACAAGGCTCCGCTCAGAGGCTCTCTGTTGCGCACCTACGTCCTTCAT

ATCCTAACCAGATCTGTGGTCGAAGTTGGCTTCATGGTGGGCCAGTATGTCCTGTATGGA

TTTCAGTTTGACCCTTTATATAAATGCACGAGAATGCCTTGCCCAAACAGCGTGGATTGT

TTTGTGTCAAGGCCAACTGAAAAGACCATTTTCATGTTGTTCATGTTTTGTATTGCAGCT

GTTTCGGTGTTTTTGAACATCCTCGAAATCTCTCACTTGGGCGTCAAAAAGATCAAGCAA

ACTCTCTACGGGAGAACCGTCAGACATGGCACAGAGGAAGATTTGAGCGTTTACAAATCC

AAGAAAAACTCCATGGTCCAGCAAGTCTGCATACTGACCAACTCTTCACCGCACAAGATG

GTTCCTTTAACTCAGACAAGTTACAAAATATTGCAGGAGGGCCAGCTGGACCCTGTGCCG

ATTTACGTGCCCTCGGCGGGGTTCAAGATGATACATGGAGGGAATGATGCGAAGGATGAG

ATCCTAAGGCAAAACCAAAATGACCACAGTCATGATGCCTTTAGCGATCAGCACCCTAGA

CAACTCCGCGAGCGCAGTCAGAGCCAGCTCCACGCGCAACGCCTGGATAAAGCCTCCGAG

CGACGCAATACATTAGAAGCCCGGGATCGTTCTTGCAGTAGCGACGACTCCAACAGGTCC

CGGCAGAACGGCATCGGAGAGAAGAGCTTCAATCAACTGCAGCAAGCTGCCCTGCCTCCT

AAAGCTTCGTTCCAGTCAAGCCAGCTGGAGATCCCGGCTGCTGTGAGGAATGCTCTTTGC

AAACAAAGCCGGGTCAGCTGCTGCAAGGACTACGCAGAAGACCGCAGCGACTCCCCAGAC

AGCGGGCACTATCCTTCCACGAGGAAGGCCAGTGTTATGTCAAAGGTGGTATCTGAGAGC

AAGCTGGCAAGTCGATCGGGTAGCCCTGACTCCAGGAACGGCTCCGGGTCAGAATCCAAG

CACCTGGGAAAAGGAGAAAGCCCCCCCATGACGCCGCCTCCGTCCGGGCGAAGAATGTCC

ATGGCAAGTAGATCACTCACT

GAGATCACCAGACAGGACATGCGTTTCAGATAC

GAGATCACCAGACAGGACGTGCGTTTCAGACAC

GAGATCACCAGACAGGATGTGCGTTTCAGACAC

GAGATCACCAGACAGGACGTGCGTTTCAGATAC

GAGATCACCAGACAGGACGTGCGTTTCAGACAC

GAGATCACCAGACAGGACGTGCGTTTCAGATAC

GAGATCACCAGACAGGACGTGCGTTTCAGACAC

GAGATCACCAGACAGGACGTGCGTTTCAGATAC

GAGATCACCAGACAGGACGTGCGTTTCAGATAC

GAGATCACCAGACAGGACGTGCGTTTCAGACAC

GAGATCACCAGACAGGACGTGCGTTTCAGATAC

GAGATCACCAGACAGCATGTGCGTTTCAGATAC

GAGATCACCAGACAGGACGTGCGTTTCAGACAC

....

>Ar-gja11-XM_034004251-cx32.2like

ATGGGTGACTGGACATTTCTGAGCAGATTACTGGAGAAAGTGCAGTCCCATTCCACAGCG

GTTGGAAGGATTTGGCTGACTGTACTGTTTGTCTTCAGGATTTTAGTACTGGGAGCTGGG

GCCGAGAAGGTCTGGGGTGATGAACAGTCTGGGTTTATCTGCAACACGCAACAACCTGGT

TGCAAGACTGTCTGTTACGACAGTGCTTTCCCCATCTCCCACATCCGCTACTGGGTCCTT

CAGATCATCTTTGTCTCAACTCCAACATTAGTCTATCTCGGGCACGTTTTGCATGTGATT

GGAAAGGAAGCAAAACATAGGCAGAATAAGCAGAGTGAAAGTGAATATGAGGGACTGATC

TTCATGAAAGCCAAGAAGTTACCAAAATATACAGATGAACAGGGAAAAGTCAGAATTCGA

GGATGTTTGCTGGGCAGTTACCTGACTAATGTCACTTGCAAAATATTGTTGGAGGTGGCA

TTCATTGTAGGCCAGTGGTATCTCTATGGCTTCACACTTGAACCCATGTTTTTGTGTAAC

AGGGAGCCCTGTCCTCACTATGTAGAATGCTTCATTTCACGTCCCACTGAGAAGTCCATT

TTTATTGTGTTTATGTTTGTGGTAGCCTGTGTGTCCCTGGCTTTAAACCTCATTGAAATA

TTGTTCCTCTGTGGGAGTAGGATTAGAGATAGCACAAAAAAATGGAATCAGCACCAGATT

GATGACCTAAAACACACTTCTGAAAGGATTTCAGATTTTAGAGCAGCTCCCCTTTCTGAC

ATTGACTTTTCCAGTAAAGAGGACCTCATGTCTCAGAACAGGATGAACTATAAGTTTGAG

CAGAATGGGGAGAAGTTGGAGAGCGAGTTCAGTAAAAGTGTTGGTGGAGAGGAGCAGGAG

AAGGAGCCAGGAGCTGAACAAGAACAACCCATTACAGGGGAACCTCAGATAGTCATCCAC

AGCTCTGAAGGAAGCATGGAAAGTGTACTAGTAGAGAATTGTTTAAGGAAAGATAGATAT

ATTTAA

>Ar-gja14A-XM_034910414-gja3like

ATGGGTGACTGGACTTTCCTGCTGAAGATCCTGGAGCAAGTCCACGAGCACTCGACTGTG

GTGGGCAAGGTCTGGCTGACCGTGCTGTTCATCTTCCGTATGATGGTCCTGGGGGCGGCC

GTGGAGAACGTCTGGGGGGACGAGCAATCGGGCTTCACCTGCAACACCCAGCAGCCAGGC

TGCCAGAACCTCTGCTACGATGATGCTTTCCCCATCTCTCACGTGCGCTACTGGGTCCTG

CAGATCATATTTGTCTCCACTCCATCCCTGGTCTACATGGGCCATGCTCTGCACAAGGTC

CGAATGGAGGAGAAACGCAAGGACCTGGGAAGAGTCGAGGGGAAGGTTGTCCAAAGCGAG

AGTGAACTGTTACTGTCTGAGAAAGATCTGGTTCCCTGCTTGAAAGAGACCCGCTTTCAG

CTGAAAGGGGCCTTACTGCAGACTTATGTCTGCAGCATCCTCTTCAGGACGGTCTTCGAA

GTGGGGTTCATCGTCGGGCAGTACTTCCTCTACGGGATCTTCTTGAGGGGACTGTACAAA

TGTAGCAGGTGGCCTTGTCCCAATACTGTGGACTGCTTTGTGTCGAGACCCACTGAGAAG

AACGTTTTCATCACCTTCATGCTGACTGTAGCCAGCATCTCCTTGCTGCTGAACCTCATG

GAGATGTATCATCTTGGGTGGAAGAAGTTCAAAGAGGGTTTGACGAATACCTTTTATAGG

AGGGCTGGTGCTTCCAAAGCAGGACCAGACAAGCCTGCTGTGCCCAAAATGGGGTACGTA

GCTCTCGCTGGCTGGAAAACCTCTGCTAGGAATTCTCCACCCAGCTGTCAACATTCTCCC

AAGCTGAAACATTTCAGTAACAAGCTGGCGAACGAGCAGAATTGGGTGAATCTTGCTACG

GAACAAGGGCAAGCTGAAAAGGGCAGTGAGATGATGCCCCAGCCAGGGTTACATAAAACT

GGCACAGCTTTGGATATGTTGGGTTTGCCCAGAGCTGGGAAAGGCAAGAGTCAGAGAAGC

AGTAGACCAAGCAGTCGGGAGAGGACTCTAGTCTGA

>Ar-gja14B-XM_034913590-gja3like

ATGGGTGACTGGACTTTCCTGCTGAAGATCCTGGAGCAAGTCCACGAGCACTCGACTGTG

GTGGGCAAGGTCTGGCTGACCGTGCTGTTCATCTTCCGTATGATGGTCCTGGGGGCGGCC

GTGGAGAACGTCTGGGGGGACGAGCAATCGGGCTTCACCTGCAACACCCAGCAGCCAGGC

TGCCAGAACCTCTGCTACGATGACGCTTTCCCCATCTCTCACGTGCGCTACTGGGTCCTG

CAGATCATATTTGTCTCCACTCCATCCCTGGTCTACATGGGCCATGCTCTGCACAAGGTC

CGAATGGAGGAGAAACGCAAGGACCTGGGAAGAGTCGAGGGGAAGGTTGTCCGAAGCGAG

AGTGAACTGTTACTGTCTGAGAAAGATCTGGTTCCCTGCTTGAAAGAGACCCGCTTTCAG

CTGAAAGGGGCCTTACTGCAGACTTATGTCTGCAGCATCCTCTTCAGGACGATCTTCGAA

GTGGGGTTCATCGTCGGGCAGTACTTCCTCTACGGGATCTTCTTGAGGGGACTGTACAAG

TGCAGCAGGTGGCCTTGTCCCAATACTGTGGACTGCTTTGTGTCGAGACCCACTGAGAAG

AACGTTTTCATCACCTTCATGCTGACTGTAGCCAGCATCTCCTTGCTGCTGAACCTCATG

GAGATGTATCATCTTGGGTGGAAGAAGTTTAAAGAGGGTTTGACGAATACCTTTTATAGG

AGGGCTGGTGCTTCCAAAGCAGGACCAGACAAGCCTGCTGGGGCCAAAATGGGGTACGTA

GCTCTCGCTGGCTGGAAAACCTCTGCTAGGAATTCTCCACCCAGCTGTCAACATTCTCCC

AAGCTGAAACATTTCAGTAACAAGCTGGCGAACGAGCAGAATTGGGTGAATCTTGCTACG

GAAGAAGGGCAAGCTGAAAAGGACAGTGAGATGATGCCCCAGCCAGGGTTACATGAAACT

GACACAGCTTTGGATATGTTGGGTTTGCCCAGAGCTGGGAAAGGCAAGAGTCAGAGAAGC

AGTAGACCAAGCAGTCGGGAGAGGACTCTAGTCTGA

>Ar-gjb1A-XM_034903629-gjb1like

ATGAATTGGGCGTCTTTTTATGCCGTTCTCAGCGGTGTAAACAGGAACTCCACTGGAATC

GGTCGCATCTGGCTGTCGGTCCTCTTCATCTTTCGAATCATGGTCCTGGTGGTCGCAGCG

GAGAGTGTCTGGGGTGACGAGAAGTCAGGCTTCATCTGCAACACGCAGCAGCCTGGTTGC

AACAGTGTCTGCTATGACCAGTTTTTCCCCATTTCCCACATCCGCCTGTGGGCACTGCAG

CTCATCCTGGTGTCCACTCCAGCCCTCCTCGTGGCCATGCACGTGGCTCACCGCCGGCAC

ATCGAGAAGAAGATCCTAAAGCTGTCCGGCCACAGCAGCAGCAAAGACCTGGAGCAGATA

AAGATGCAGAGGATGAAGATCACGGGGGCCCTGTGGTGGACCTACATGATTAGCGTGATC

TTCCGGATCCTCTTCGAAGCCGCTTTCATGTACATCTTCTACCTGATCTACCCGGGCTTC

AAGATGATTCGGTTAGTCAAGTGCGACGCCTACCCCTGCCCCAACACTGTGGACTGCTTT

GTATCCAGACCCACGGAGAAAACCATCTTCACCATCTTCATGCTGGTGGTGTCTGGGGTC

TGTATCCTCCTCAACATCGCAGAGGTTGTCTACTTGATCACCAAGACCTGTTTCAAGAGC

TTGCCAGACTCGGAGTCCGGCTCCAAAGGTACTTTTTATGGGCACAGGTTCTCGTCGTAC

AAACAGAACGAGATCAATCAGCTGATCTTGGACTCGAAAATCAACGGGTCGAAGAGAAAT

CCCGGTTCTGATAAGGGGGACAGGTGTTCGGCTTGTTAG

>Ar-gjb1B-XM_034019835-gjb1like

ATGAATTGGGCGTCTTTTTATGCCGTTCTCAGCGGTGTAAACAGGAACTCCACTGGAATC

GGCCGCATCTGGCTGTCGGTCCTCTTCATCTTTCGAATCATGGTCCTGGTGGTCGCAGCG

GAGAGTGTCTGGGGCGACGAGAAGTCAGGCTTCATCTGCAACACGCAGCAGCCTGGTTGC

AACAGTGTCTGCTATGACCAGTTTTTCCCCATTTCCCACATCCGCCTGTGGGCACTGCAG

CTCATCCTGGTGTCCACGCCAGCCCTCCTCGTGGCCATGCACGTGGCTCACCGCCGGCAC

ATCGAGAAGAAGATCCTAAAGCTGTCCGGCCACAGCAGCAGCAAAGACCTGGAGCAGATC

AAGATGCAGAGGATGAAGATCACGGGGGCCCTGTGGTGGACCTACATGATTAGCGTGATC

TTCCGGATCCTCTTCGAAGCCGCTTTCATGTACATCTTCTACCTGATCTACCCGGGCTTC

AAGATGATTCGGTTAGTCAAGTGCGACGCCTACCCCTGCCCCAACACTGTGGACTGCTTT

GTATCCAGACCCACGGAGAAAACCATCTTCACCATCTTCATGCTGGTGGTGTCTGGGGTC

TGTATCCTCCTCAACATCGCAGAGGTTGTCTACTTGATCACCAAGACCTGTTTCAAGAGC

TTGCCAGACTCGGAGTCCGGCTCCAAAGGTACTTTTTATGGGCACAGGTTCTCGTCGTAC

AAACAGAACGAGATCAATCAGCTGATCTTGGATTCGAAAAGCAACGGGACGAAGAGAAAT

CCCGGTTCTGATAAGGGGGACAGGTGTTCGGCTTGTTAG

>Ar-gjb3A-XM_034906208-gjb3like

ATGGACTGGAAGACACTCCAAGGCCTCCTGAGTGGGGTCAACAAGTACTCCACGGCGTTC

GGCCGCATCTGGCTGTCCGTGGTCTTTGTGTTCCGAGTCATGGTGTACGTGGTGGCGGCC

GAGAAGGTGTGGGGCGACGAGCAGAAGGATTTTGACTGCAACACCAAGCAGCCCGGCTGC

ACCAACGTCTGCTACGACTATTTTTTCCCCATCTCCCACATCCGCCTGTGGGCCCTGCAG

CTCATCTTCGTCACCTGCCCCTCACTGCTCGTGGTCATGCACGTGGCCTACAGGGAGGTC

CGCGAGAAGAAGCACAAGCAGGAGCATGGAGAGAACTGCTCCAACCTGTACCAGAACACA

GGGAAGAAGCACGGCGGGCTGTGGTGGACCTACTTGCTCAGCCTCTTCGCCAAGTTGGGC

ATCGAAATAGGCTTCCTCTACATCCTCCACCACATTTACGACAGCTTCTACCTGCCACGC

CTGGTGAAGTGCGAGGTCCTACCCTGCCCCAACGTGGTGGATTGCTACATCGGCCGGCCG

ACGGAGAAGAAGGTCTTCACCTACTTCATGGTGGGTGCCTCTGCCCTCTGCATCCTGCTG

AGCGTTTGTGAGATCATCTATCTCATCACCAAGCGAGTCCTGCGCTGCAGCAGGAAGCTG

AAGAACAAAAGCAAGAGGAACTCAGTGGCCTACAGCAAAGCCTCCACCTGCACGTGTCAC

CACAAGATGACACCCCTGGACTTGAAGCCTGCCCTCAAAGGCACGGAGCTGCGGGCGTCC

GCTCCCAACCTCTTTTTCGGTCAATGA

>Ar-gjb3B-XM_034908573-gjb3like

ATGGACTGGAAGACACTCCAAGGCCTCCTGAGTGGGGTCAACAAGTACTCCACGGCGTTC

GGCCGCATCTGGCTGTCCGTGGTCTTTGTGTTCCGAGTCATGGTGTACGTGGTGGCGGCC

GAGAAAGTGTGGGGCGACGAGCAGAAGGACTTTGACTGCAACACCAAGCAGCCCGGCTGC

ACCAACGTCTGCTACGACTATTTCTTTCCAATCTCCCACATCCGCCTGTGGGCCCTGCAG

CTCATCTTCGTCACCTGCCCCTCGCTGTTGGTGGTCATGCACGTGGCCTACAGGGAGGAC

CGTGAGAAGAAGCACAAGCAGAAGAATGGAGAGAACAGCTCCAACCTGTACCAGAACACC

GGGAAGAAGCACGGCGGGCTGTGGTGGACCTACTTGCTCAGCCTCTTCGTCAAGTTGGGC

ATCGAAATAGGCTTCCTCTACATCCTCCACCACATTTACGACAGCTTCTACCTGCCACGC

CTGGTGAAGTGCGAGGTCCTACCCTGCCCCAACGTGGTGGATTGCTACATCGGCCGGCCG

ACAGAGAAGAAGGTCTTCACCTACTTCATGGTGGGCGCCTCTGCCCTCTGCATCGTGCTG

AGCATTTGTGAGATCATCTATCTCATCGCCAAGCGAGTCCTGCGCTGCAGCAGGAAGCTG

AAGAACAAAAGCAAGAGGAACTCAGTGGCCTACAGCAAAGCCTCCACCTGCACGTGTCAC

CACAAGATGACACCCCTGGACTTCAAGCCTGCCCTCAAAGGCACTGAGATGCGGGCGTCC

GCTCCCAACCTCTTATTTAGTCAATGA

>Ar-gjb7A-XM_034005445-gjb7like

ATGAACTGGAGCTTTCTCCGAGATATCCTGAGTGGAGTGAACAAGTACTCCACGGTGATC

GGGAGGATCTGGCTGTCCGTGGTCTTCATATTCCGCATCCTGATCTACGTGGCAGCGGCG

GAGCACGTGTGGAAGGACGAGCAGAGTGGCTTTGTGTGCAACATCAAACAGCCAGGCTGC

GAGAACGTGTGCTTTGACTACTTCTTTCCCATCTCCCAAATCAGGCTGTGGGCCTTGCAG

CTCATCATGGTTTCCACCCCGTCTCTTTTGGTTGTGCTTCATGTGGCGTACCGTCAACAC

AGGGAGCACACGCACAGGAAGAAGCTGTATGTGGATACAGGGACGATGGATGGGGGTCTT

TGGTGTACCTATCTCGTCAGCCTCCTGTTCAAGACGTTCTTTGAGGTGGGCTCCCTGCTG

GCATTTTACTATCTCTACAGTGGCTTTACTGTGCCTCAGCTGCTCCAGTGTACAATGAGC

CCTTGCCCCAATACAGTCGACTGCTACGTTTCCCGGGCAACCGAGAAAATGATCTTCCTT

TACATCATGGGCTCCACTTCCATCTTGTGCATCGCACTAAATGTGTGCGAGATGGTCTAC

CTTCTACTCCAGCAGTGCTGGAAATGTTATTTTAAGAGGTACGTCCCGGTCATGGAGGAA

CACCAGTGCCACTGTGCAAAGCCCAGGCCTTTAAACAGCAGTGCAGCTGCAATCCCTGTG

CCCCTGAAGGTAGACTCCACAAACTCAAGCTTTCATACATTATAG

>Ar-gjb7B-XM_034017448-cx28.8

ATGAACTGGGGCTTTCTCCAAGGTATCCTGAGCGGAGTGAACAAGTACTCCACGGTGATC

GGGAGGATCTGGCTGTCCGTGGTCTTCATATTCCGCATCCTGATCTACGTGGCCGCGGCG

GAGCATGTGTGGAAGGACGAGCAGAAGGACTTTGTGTGCAACATCAAACAGCCGGGCTGC

GAGAACGTGTGCTTTGACTACTACTTTCCCATCTCCCAAATCAGGCTGTGGGCCTTGCAG

CTCATCATGGTTTCCACCCCGTCTCTTTTGGTTGTGCTTCACGTGGCGTACCGTCAACAC

AGGGAGCACACGCACAGGAAGAAGCTGTATGTGGATACAGGGACGATGGATGGGGGTCTT

TGGTGTACCTATCTCATCAGCCTCCTGTTCAAGACGTTCTTTGAGGTGGGCTCCCTGCTG

GCATTTTACTATCTCTACAGTGGCTTTACTGTGCCTCAGCTATTCCAGTGTACAATGAGC

CCATGCCCCAACACAGTCGACTGCTACATTTCCCGGGCAACCGAGAAAAAGATCTTCCTT

TACATCATGGGCTCCACTTCCATCTTGTGCATCGCGCTGAATGTGTGTGAGATGGTCTAC

CTTCTACTCCAGCAGTGCTGGAAATGTTATTTTAAGAGGTACGTCCCGGTCATGGAGGAA

CACCAGTGCCACTGTGCAAAGCCAAGGCCTTTAAACAGCACTGCAGCTGCAATCCCTGTG

CCCCTGAAGGTAGACTCCACAAACTCAAGCTTTCACACAATTTAG

>Ar-gjb7C-XM_034017449-cx28.8

ATGAACTGGGGCTTTCTCCAAGGTATCCTGAGCGGAGTGAACAAGTACTCCACGGTGATC

GGGAGGATCTGGCTGTCCGTGGTCTTCATATTCCGCATCCTGATCTACGTGGCCGCGGCG

GAGCATGTGTGGAAGGACGAGCAGAAGGACTTTGTGTGCAACATCAAACAGCCGGGCTGC

GAGAACGTGTGCTTTGACTACTTCTTTCCCATCTCCCAAATCAGGCTGTGGGCCTTGCAG

CTCATCATGGTTTCCACCCCGTCTCTTTTGGTTGTGCTTCACGTGGCGTACCGTCAACAC

AGGGAGCACACGCACAGGAAGAAGCTGTATGTGGATACAGGGACGATGGATGGGGGTCTT

TGGTGTACCTATCTCGTCAGCCTCCTGTTCAAGACGTTCTTTGAGGTGGGCTCCCTGCTG

GCATTTTACTATCTCTACAGTGGCTTTACTGTGCCTCAGCTATTCCAGTGTACAATGAGC

CCATGCCCCAACACAGTCGACTGCTACATTTCCCGGGCAACCGAGAAAAAGATCTTCCTT

TACATCATGGGCTCCACTTCCATCTTGTGCATCGCGCTGAATGTGTGTGAGATGGTCTAC

CTTCTACTCCAGCAGTGCTGGAAATGTTATTTTAAGAGGTACGTCCCGGTCATGGAGGAA

CACCAGTGCCACTGTGCAAAGCCAAGGCCTTTAAACAGCACTGCAGCTGCAATCCCTGTG

CCCCTGAAGGTAGACTCCACA

>Ar-gjb8A-XM_034011049-gjb2

ATGAACTGGGCGCAGCTGTATTCTCTGTTAGGAGGCGTGAACAAGCACTCCACCAGCCTT

GGGAAGGTCTGGCTGTCTGTCCTCTTCATCTTCCGCATTATGATCCTCATCGTGGCAGTG

GAGAGTGTCTGGGGAGATGAACAATCGGACTTCACCTGCAACACCCTACAGCCCGGCTGC

AAGAATGTCTGCTACGACCACTACTTTCCCATCTCCCACATTCGGCTCTGGAGCCTTCAG

CTTATTTTTGTTTCCACTCCCGCTCTCCTGGTAGCCATGCACGTGGCTCACAGCATAAAA

GAGGAAAAGAAGGATCTCATTAGAACTATGGGTGACAAAGCAGATGTGGACATAGAAATG

CTTAAGAAGAAGAAGTTCCATATTGTGGGGCCGCTGTGGTGGACCTACACTGTTAGTCTG

TTTTTCAGGTTGCTGTTTGAAGCTGCCTTCATGTACGTGTTTTATTATCTTTACAATGGC

TTCCATATGCCACGCCTGGTGAAATGTGATGCTTGGCCCTGCCCAAACACAGTTGATTGT

TTCATTTCTCGACCCACCGAGAAGACCGTGTTCACCATTTTCATGATTGCTGCCTCGTCC

CTGTGTATGGTACTCAATGTGGCTGAACTGTCCTACCTGATCGTAAAGGCTTTGCTGGGG

TATTGCAGAAAACAAAGATGTCACCCTAGTCATGTTTCCAAAAATAAGGAATGCCACCAA

AATAAAAGGAATGAAATCCTGTTATTGGAATCCAGAAGAGCCAGTGCTCCACAGAAATAG

>Ar-gjb8B-XM_034919050-gjb2like

ATGAACTGGGGCCAGCTGTATTCTCTGTTAGGAGGCGTGAACAAGCACTCCACCAGCCTT

GGGAAGGTCTGGCTGTCTGTCCTCTTCATTTTCCGCATTATGATCCTCATCGTGGCAGTG

GAGAGTGTCTGGGGAGATGAACAGTCGGACTTCACCTGCAACACCCTACAGCCCGGCTGC

AAGAACGTCTGCTACGACCACTACTTCCCCATCTCCCACATTCGGCTCTGGAGCCTTCAG

CTCATTTTTGTTTCCACTCCTGCTCTCCTGGTAGCCATGCACGTGGCTCACAGCATAAGA

GAGGAAAAGAAGGATCTCATTAGAACTATGGGTGACAAAGCAGATGTGGACATAGAAATG

CTTAAGAAGAAGAAGTTCCATATTGTGGGGCCGCTGTGGTGGACCTACACTGTTAGTCTG

TTTTTCAGGTTGCTGTTTGAAGCTGCCTTCATGTATGCTTTTTATTATCTTTACAGTGGC

TTCCATATGCCACGCCTGGTGAAATGTGACGCTTGGCCCTGCCCAAACACAGTTGATTGT

TTCATTTCTCGACCCACCGAGAAGACCGTGTTCACCATTTTCATGATTGCTGCCTCATCC

CTGTGCATGGTACTCAATGTGGCTGAACTGTCCTACCTGATCATAAAAGCTTTGCTGGGG

TGTTGCAGAAAACAAAGGTATCACTCTGGTCATGTTTCCAAAAGTAAGGAACGCCACCAA

AATAAAAGGAATGAAATCCTGCTATTGGAATCCAGCAAGGCTAGTGCTCCACATAAATAG

>Ar-gjb8C-XM_034009766-gjb2like

ATGAACTGGGGCCAGCTGTATTCTCTGTTAGGAGGCGTGAACAAGCACTCCACCAGCCTT

GGGAAGGTCTGGCTGTCTGTCCTCTTCATTTTCCGCATTATGATCCTCATCGTGGCAGTG

GAGAGTGTCTGGGGAGATGAACAGTCGGACTTCACCTGCAACACCCTACAGCCCGGCTGC

AAGAACGTCTGCTACGACCACTACTTCCCCATCTCCCACATTCGGCTCTGGAGCCTTCAG

CTCATTTTTGTTTCCACTCCTGCTCTCCTGGTAGCCATGCACGTGGCTCACAGCATAAGA

GAGGAAAAGAAGGATCTCATTAGAACTATGGGTGACAAAGCAGATGTGGACATAGAAATG

CTTAAGAAGAAGAAGTTCCATATTGTGGGGCCGCTGTGGTGGACCTACACTGTTAGTCTG

TTTTCAGGTTGCTGTTTGAAGCTGCCTTCATGTATGCTTTTTATTATCTTTACAGTGGCT

TCCATATGCCACGCCTGGTGAAATGTGACGCTTGGCCCTGCCCAAACACAGTTGATTGTT

TCATTTCTCGACCCACCGAGAAGACCGTGTTCACCATTTTCATGATTGCTGCCTCATCCC

TGTGCATGGTACTCAATGTGGCTGAAACTGTCCTACCTGATCATAAAAGCTTTGCTGGGG

TGTTGCAGAAAACAAAGGTATCACTCTGGTCATGTTTCCAAAAGTAAGGAACGCCACCAA

AATAAAAGGAATGAAATCCTGCTATTGGAATCCAGCAAGGCTAGTGCTCCACATAAATAG

>Ar-gjb8D-XM_034008147-cx30.3

ATGAACTGGGGCCAGCTGTATTCTCTGTTAGGAGGCGTGAACAAGCACTCCACCAGCCTT

GGGAAGGTCTGGCTGTCTGTCCTCTTCATTTTCCGCATTATGATCCTCATCGTGGCAGTG

GAGAGCGTCTGGGGAGATGAACAGTCGGACTTCACCTGCAACACCCTACAGCCCGGCTGC

AAGAACGTCTGCTACGACCACTACTTCCCCATCTCCCACATTCGGCTCTGGAGCCTTCAG

CTCATTTTTGTTTCCACTCCTGCTCTCCTGGTAGCCATGCACGTGGCTCACAGCATAAGA

GAGGAAAAGAAGGATCTCATTAGAACTATGGGTGACAAAGCAGATGTGGACATAGAAATG

CTTAAGAAGAAGAAGTTCCATATTGTGGGGCCGCTGTGGTGGACCTACACTGTTAGTCTG

TTTTCAGGTTGCTGTTTGAAGCTGCCTTCATGTATGCTTTTTATTATCTTTACAGTGGCT

TCCATATGCCACGCCTGGTGAAATGTGACGCTTGGCCCTGCCCCCAAACACAGTTGATTG

TTTCATTTCTCGACCCACCGAGAAGACCGTGTTCACCATTTTCATGATTGCTGCCTCATC

CCTGTGCATGGTACTCAATGTGGCTGAACTGTCCTACCTGATCATAAAAGCTTTGCTGGG

GTGTTGCAGAAAACAAAGGTATCACTCTGGTCATGTTTCCAAAAGTAAGGAACGCCACCA

AAATAAAAGGAATGAAATCCTGCTATTGGAATCCAGCAAGGCTAGTGCTCCACATAAATAG

>Ar-gjb9A-XM_034024040-gjb4like

ATGAACTGGACGGCGCTGGAAGGTCTGCTCAGCGGGGTCAACAAGTACTCCACGGTGTTC

GGCCGCGTGTGGCTCTCGGTGGTCTTCGTCTTCCGAGTGCTGGTGTTCGTGGTGGCGGCG

CAGAAGGTGTGGGGCGATGAGTCCAAGGACTTCGTCTGCAACACGAAGCAGCCCGGCTGC

ACCAACGTCTGTTACGACAGCATCTTCCCCATCTCCCACGTGCGCCTCTGGGCCCTGCAG

CTCATCTTCATCACCTGCCCCTCCTTGCTGGTGGTGGCTCACGTCAAGTACCGCGAGATC

AAGGAGCTGAAGTACGCCAAAGCCCACAAGGGCTCCCACCTCTACGCCAACCCCGGGAAG

AAGCGTGGCGGGCTCTGGTGGACCTACTTCTTCAGCCTGGTCTTCAAGGCCGGCTTCGAC

GCAGCCTTCCTCTACATCCTCTTCTACATCTACCACGGCTATGACATGCCCCGGCTCTCC

AAGTGTGAGCTGGAGCCCTGCCCCAACATGGTGGATTGCTTCATCTCCCGCCCCACCGAG

AAGAAGATCTTCACCATCTTCATGGTGGTCACCTCGGCACTGTGCATCTTCATGTGCGTC

ATCGAGATGCTCTACCTGGTAGGCAAGCGCTGCCAGAAGATCATGAGGATGAGTAAGCAG

AACGAGAGGTATCTCCTCTCAGAGTCCAGCCAGCCTGGATTCATCCAGCTCTACCAGCCC

ATTCCGCTCAACAACAGCAGCGCCAAGGAAGGCAAGAAGGTGGTGAAGGTGCCAGCGCAG

GTCAACAATTCCACCCTTGAAACTGGGACCGGTTTAAGTGTGCCACACGGTGCTTCCCAG

TCCACTTCATCTTGCACTGCAGGACCTGCTTCACCAAACTGA

>Ar-gjb9B-XM034022435-gjb4like

ATGAACTGGACGGCGCTGGAAGGTCTGCTCAGCGGGGTCAACAAGTACTCCACGGTGTTC

GGCCGCGTGTGGCTCTCGGTGGTCTTCGTCTTCCGAGTGCTGGTGTTCGTGGTGGCGGCG

CAGAATGTGTGGGGCGATGAGTCCAAGGACTTCGTCTGCAACACGAAGCAGCCCGGCTGC

ACCAACGTCTGTTACGACAGCATCTTCCCCATCTCCCACGTGCGCCTCTGGGCCCTGCAG

CTCATCTTCGTCACCTGCCCCTCCTTGCTGGTGGTGGCTCACGTCAAGTACCGCGAGATC

AAGGAGCTGAAGTACGCCAAAGCCCACAAGGGCTCCCACTTCTACGCCAACCCTGGGAAG

AAGCGTGGCGGGCTCTGGTGGACCTACTTCTTCAGCCTGGTCTTCAAGGCCGGCTTCGAC

GCAGCCTTCCTCTATATCCTCTTCTACATCTACCACGGCTATGACATGCCTCGGCTCTCC

AAGTGTGAGCTGGAGCCCTGCCCCAACGTGGTGGATTGCTTCATCTCCCGCCCCACCGAG

AAGAAGATCTTCACCATCTTCATGGTGGTCACCTCGGCACTGTGCATCTTCATGTGCGTC

ATCGAGATGCTCTACCTGGTAGGCAAGCGCTGCCAGAAGATCATGAGGATGAGGAAGCAG

AACGAGAGGTATCTCATCTCAGAGTCCAGCCAGCCTAGATTCATCCAGCTCTACCAGCCC

GTTCCGCTTAACAACAGCAGCACCAAGGAAGGCAAGAAGGTGGTGAAGGTGCCAGCGCAG

GTCAACAATTCCACCCTTTAA

>Ar-gjb10A-XM_034906258-gjb4like

ATGAACTGGGCGTTCCTCCAGGGCTTGTTGAGCGGGGTCAACCAGTACTCCACGGCGTTC

GGCCGCGTCTGGCTCTCCATCGTCTTCCTCTTCCGGGTCATGGTGTACGTGGTGGCGGCC

GAGCGGGTCTGGGGAGATGACCAGAAGGACTTCCAGTGCAACACGGCCCAGCCCGGCTGC

CACAACGTCTGCTACGACCACTTCTTCCCTGTCTCCCACATCCGCCTGTGGGCCCTGCAG

CTCATCTTCGTCACCTGCCCCTCACTGCTCGTGGTCATGCACGTGGCCTATAGGGAGGAA

CGCGAGCGCAGGCACCGCGAGCGCAACGGGGAGGACTGTGGCCGCCTGTACAAGGACACG

GGCAAGAAGAGGGGTGGCCTCTGGTGGACCTACGTCTTAACCTTGGTCTTCAAGGCCGGG

GTGGACGCCACCTTCCTCTACGTCCTCTACTACATTTATGAAGCTTACGACTTCCCGCGC

CTCATCAAGTGCAGCCAGAAGCCCTGCCCCAACGTGGTGGACTGCTTCATTTCTAGGCCC

ACGGAGAAGAAGATCTTCACCATCTTCATGGTGGTCACATCCATTCTCTGCATCTTCCTA

AACCTCTGCGAGCTCATCTACCTCCTGGGCAAGCGCTGTCGGGAGTGCGTGGTCTCCAAC

AACCGCCGGCGACAGCTGTTCATGGCCTCCACCGTGATCAACAACAAGGAGAGCAACAGC

ATTATGGAAAACTCTCTGAAGGTGCCGCCCTATGCCCCTCCCAGCATTAAGGTTTCCAAG

TAG

>Ar-gjb10B-XM_034021714-gjb4

ATGAACTGGGCGTTCCTCCAGGGCTTGTTGAGCGGGGTCAACCAGTACTCCACGGCATTC

GGCCGCGTCTGGCTCTCCATCGTCTTCCTCTTCCGGGTCATGGTGTACGTGGTGGCGGCC

GAGCGGGTCTGGGGAGATGACCAGAAGGACTTCCAGTGCAACACGGCCCAGCCCGGCTGC

CACAACGTCTGCTACGACCACTTCTTCCCCGTCTCCCACATCCGCCTGTGGGCCCTGCAG

CTCATCTTCGTCACCTGCCCCTCACTGCTCGTGGTCATGCACGTGGCCTATAGGGAGGAG

CGCGAGCGCAGGCACCGCGAGCGCAATGGGGAGGACTGCGGCCGCCTGTACAAGGACACG

GGCAAGAAGAGGGGTGGCCTCTGGTGGACCTATGTCTTAACCTTGGTCTTCAAGGCCGGG

GTGGACGCAACCTTCCTCTACGTCCTCTACTACATTTACGAGGCTTACGACTTCCCGCGC

CTCATCAAGTGCAGCCAGAAGCCCTGCCCCAACGTGGTGGACTGCTTCATATCTAGGCCC

ACGGAGAAGAAGATCTTCACCATCTTCATGGTGGTCACGTCCATTCTCTGCATCTTCCTA

AACCTCTGCGAGCTCATCTACCTCCTGGGCAAGCGCTGTCGGGAGTGCGTGGTCTCCAAC

AACCCCCGGCGTCAGCTGTTCATGGCCTCCACCATGATCAACAACAAGGAGAGCAACAGC

ATAATGGAAAACTCTCTGAAGGTGCCGCCCTATGCCCCTCCCAGCATTAAGGTTTCCAAG

TAG

>Ar-gjc1A-XM_034057391-gjc1

ATGAGCTGGGCCTTCCTGACACGGCTGCTTGAGGAGATCCACAACCACTCCACCTTCGTG

GGGAAGCTGTGGCTGACCGTGCTCATCGTGTTCCGCATCGTGCTGACGGTCGTGGGCGGC

GAGTCCATCTACTACGACGAGCAGAGCAAGTTCGTGTGCAACACGGCCCAGCCGGGCTGC

GAGAACGTCTGCTATGATGCCTTCGCCCCGCTGTCCCACGTGCGTTTCTGGGTCTTCCAG

ATCATCCTGGTGGCCACGCCCTCCGTCATGTACCTGGGCTACGCCGTGCACAAGATCGCC

CGCAGCGAGGAGGGGCGCGGCTGCCGGCAGGGGGGCTTCGGCTGCGACAGGAAGCAGCGA

CGCAAGCTGTTCAGGGGGGACAGGAAGCAGCACCGTGGCATCGAGGAGACCGAAGAGGAC

AACGAGGAGGAGGACCCCATGATCTACGAGGAGCCCGAGCTGGAGAGCCGCAGCGAGGGG

AACGGCGCGGGCGCTGCGACGCAGGCCAGGACCGCACCGGGGAAGGCAGGGCGGCACGAT

GGGCGCCGGCGCATCAAAGAGGACGGGCTGATGAAGATCTACGTGATGCAGCTGCTGGCA

CGCACCTCCCTGGAGGTGGGCTTCCTCGGGGGGCAGTACGCCCTGTACGGCTTCGAGGTG

TCCCCCGCCTACGTGTGCGAGCGCAGCCCCTGCCTCCACAAGGTGGACTGCTTCATCTCG

CGCCCCACCGAGAAGACCATCTTCCTGCTCATCATGTACGCCGTCTCCCTGCTCTGCCTA

GTCATCAACCTGTGGGAGATGCTGCACCTGGGCTTGGGCACCATTCACGATGTCCTGCGC

AGCAAACACCAGCCCCCTGAGGAGGGGCACCACCTCCCCCAGCCCCCCGGGGGCCCCACC

AGCGCCCCCTACTCCTACCCCTTCACCTGGAATGCCCCCTCCGCGCCTCCCGGCTACAAC

ATCGTGGTTAAGCCGGAGCAGATCCCCTACACTGACCTCAGCAACGCCAAGATGGCCTGC

AAGCAGAACAAAGCCAACATTGCGCAGGAGGAGCAGCAGCAGTACGGCAGCAACGAGGAC

AACTTCCCTGGCACCGGCCCGGCCGCGGAGCAGAGAGAGATCCGCGCCGCTCAACACAGG

TTGGAGGCTACCATCCAGACCTACAACCAGAACCACCACAGCCCACAACACCAGCACCAG

AACATCTCCAGCGCAGCCAAGGACAACAAGAAGCAGCACAAGCCTGGCTCCAGCAGGAGC

GTGAGGGGAGGAGGGGGAGCCAGCAGCTGCAGCAGGTCCCTGGGAGGGAAGTCCTCCGTT

TGGATTTAG

>Ar-gjc1B-Not predicted

CTTCCTGACACAGCTGCTTGAGGAGATCCACAACCACTCCACCTTCGTGGGGAAGCTGTG

GCTGACCGTGCTCATCGTGTTCCGCATCGTGCTGACGGTCGTGGGCAGCGAGTCCATCTA

CTACGACGAGCAGAGCAAGTTCGTGTGCAACACGGCCCAGCCGGGCTGCGAGAACGTCTG

CTACGACGCCTTCGCCCCGCTCTCCCACGTGCGCTTCTGGGTCTTCCAGATCATCCTGGT

GGCCACCCCCTCCGTCATGTACCTGGGCTACGCCGTGCACAAGATCGCCCGCAGCGAGGA

GGGGCGCGGCTGCGACGGGAAGCAGAAACGCAAGCTGTTCGGGTCGGGCAGGAAGCAGCA

CCGCGGCATCGAGGAGACCGAAGAGGACAACGAGGAGGAGGACCCCATGATCTACGAGGA

GCCCGAGCTGGAGAGCCGCAGCGAGGGGAACGGCGCCGCGGCGCTGACCAGGACCACGCC

GGAGAAGGCAGGGCGGCACGACGGGCGGCGGCGCATCAAAGAGGACGGGCTGATGAAGAT

CTACGTGATGCAGCTGCTGGCACGCACCTCCCTGGAAGTGGGCTTCCTCGGGGGGCAGTA

CGCCCTGTACGGCTTCGAGGTGTCCCCCGCCTACGTGTGCGAGCGCAGCCCCTGCCCCTA

CAAGGTGGACTGCTTCATCTCACGCCCCACCGAGAAGACCATCTTCCTGCTCATCATGTA

CGCAGTCACCGTGCTCTGCCTGGTCCTCAACCTGTGGGAGATGCTGCACCTGGGCTTGGG

CACCATTCACGATGTCCTGCGCAGCAAACGCCAGCCCCCCGAGGAGGGGTACCATCTCCC

CCAGCCCCCCGGGGCCCCCGCCCGCGCCCCCGACTCCTACCCCTTCACCTGGAACACCCC

CTCCGGGCCTCCCGGCTGCAGATCCCCTACACCGACCTCAGCAACACCAAGATGGCCTGC

AAGCAGAACAAAGCCAACATCATGCAGGAGGAGCAGCGAGAGAAACCCACCGCTCAGCAC

AGACTGGAGGCTGCCATCCAGACCTACAACCAGAACCACCACAGCCCACACCACCAGCAC

CAGAACATCTCCAGCACAGCCAAGGACAAGAAGCAGCAGCACAAGCCTGGCTCCAGCAGGAG

>Ar-gjc1C-Not predicted

CTTCCTGACACAGCTGCTTGAGGAGATCCACAACCACTCCACCTTCGTGGGGAAGCTGTG

GCTGACCGTGCTCATCGTGTTCCGCATCGTGCTGACGGTCGTGGGCGGCGAGTCCATCTA

CTACGACGAGCAGAGCAATTTCGTGTGCAACACGGCCCAGCCGGGCTGCGAGAACGTCTG

CTACGATGCCTTCGCCTCGCTCTCCCACGTGCGCTTCTGGGTCTTCCAGATCATCCTGGT

GGCCACGCCCTCCGTCATGTACCTGGGCTATGCCGTGCACAAGATCGCCCGCAGCGAGGA

GGGGCGCGGCTGCGACGGGAAGCAGAAACGCAAGCTGTTCGGGGCGGGCAGGAAGCAGCA

CCGCGGCATCGAGGAGACCGAAGAGGACAACGAGGAGGAGGACCCCATGATCTACGAGGA

GCCCGAGCTGGAGAGCCGCAGCGAGGGGAACGGCGCCGCGGCGCTGACCAGGACCACGCC

GGAGAAGGCAGGGCGGCACGACGGGCGGCGGCGCATCAAAGAGGACGGGCTGATGAAGAT

CTACGTGATGCAGCTGCTGGCACTCACCTCCCTGGAGGCAGGCTTCCTCGGGGGGCAGTA

CACCCTGTACGGCTTCGAGGTGTCCCCCGCCTACGTGTGCGAGCGCAGCCCCTGCCCCTA

CAAGGTGGACTGCTTCATCTCGCGCCCCACCGAGAAGACCATCTTCCTGCTCATCATGTA

CGCCGTCACCCTGCTCTGCCTGGTCCTCAACCTGTGGGAGATGCTGCACCTGGGCTTGGG

CACCATTCACAATGTCCTGCGCAGCAAACGCCAGCCCCCCGAGGAGGTGTACCACCTCCC

CCAGCCCCCCGGGGCCCCCGCCAGCGCCCCCTACTCCTACCCCTTCACCTGGAACACCCC

CTCCGGGCCTCCCGGCTGCAGATCCCCTACACCGACCTCAGCAACACCAAGATGGCCTGC

AAGCAGAACAAAGCCAACATCATGCAGGAGGAGCAGC

>Ar-gjc2A-XM_034000763-gjc1

ATGAGCTGGAGCTTTCTCACCCGTCTTCTGGAAGAAATCCACAACCACTCCACCTTTGTG

GGGAAAGTGTGGCTAACCGTTCTTATCATCTTTCGCATTGTGCTGACAGCAGTCGGAGGG

GAGTCCATCTACTCGGATGAACAGACCAAATTCACCTGCAATACCAAGCAACCTGGATGT

GACAATGTCTGCTACGATTCCTTCGCCCCCCTGTCCCACGTCCGTTTCTGGGTCTTCCAG

ATCATCATGATCTCAACACCTTCCATCATGTATCTGGGCTACGCCATCCACAAAATTGCC

CGCTCATCTGAGGAGGAGAGGAGGAGGTTCAAGAAGTTCCGGAAGAAGCCCCACGCCATC

AAGTGGAAGGCCACCCACACTCTGGACGAGGTGCTGGAAGAGGAAGAGGAGGAGCCCATG

ATCTATGAGGACACACTGGAGGTGCATGAGATGAAGCCAGAGAGCAAAGTGCAGGGCAGG

GAACAGCAGAAGCACGACGGCCGGCGTAGAATCATGCAGGAAGGACTAATGAAGATTTAT

GTCCTCCAGCTTCTGGCCCGAGCTATTTTTGAGGTGGCTTTTCTCGGGGGCCAGTATCTT

CTATATGGCTTCAAGGTCAGCCCATCGTACATCTGCGCCAAGAACCCCTGCCCTCACCAG

GTAGACTGCTTTGTGTCTAGGCCCACAGAAAAGACCATCTTCCTCCTGATCATGTACGTG

GTCAGCTGCTTGTGCCTGTTGCTGAACATCTGTGAGATGTTCCACTTGGGTATCGGCAGC

ATCCGGGACATCATCCGCAACAGGAGGAACAAGGGCAAGCGCCCGTCCTACAGCTACCCC

TACCCCAGGAACATTCCTTCATCGCCCCCTGGGTATAACTTGGTGGTCAAGTCGGATAAA

CCCAATAAGATCCCCAACAGTCTGATCACACATGAGCAGAATTTGGCTAACGTGGCACAA

GAGCAGCAGTGCACCAGCCCCGATGAAAATATTCCCTCTGATCTGGCTAGCCTGCACAGG

CACCTCAGGGTCGCCCAAGAGCAGTTGGACCTGGCCTTTCAGACTTACAACAGCAAAAGC

AATGCACAGCCTTCAAGAACCAGCAGCCCGGCCTCTGGCGGGACTGTGGTGGAGCAGAAC

CGAGTCAATACAGCTCATGAGAAACAAGGGGCGCGGCCAAAATGCAGCACAGAGAAAGCA

GGGTCTATCATTAAAGATGGAAAGACATCTGTGTGGATTTAA

>Ar-gjc2B-XM_033993461-gjc1like

ATGAGCTGGAGCTTTCTCACCCGTCTTCTGGAAGAAATCCACAACCACTCCACCTTTGTG

GGGAAAGTATGGCTGACTGTTCTTATCGTCTTTCGCATTGTGCTGACAGCAGTCGGAGGG

GAGTCCATCTACTCGGACGAACAGACCAAGTTCACATGCAACACCAAGCAACCTGGATGT

GACAATGTCTGCTACGATTCCTTCGCCCCCCTGTCCCACGTCCGTTTCTGGGTCTTTCAG

ATCATCATGATCTCAACACCTTCCATCATGTATCTGGGCTACGCCATCCACAAAATCGCC

CGCTCATCTGAGGAGGAGAGGAGGAGGTTCAAGAAGTTCCGGAAGAAGCCCCACACCATT

AAGTGGAAAGCCACCCAAACTCTGGATGAGGTGCTGGAAGAGGAAGAGGAGGAGCCCATG

GTCTATGAGGACACACTGGAGGTGCATGAGATGAAGCCAGAGAGCAAAGTGCAGGGCAGG

GAACAGGTGAAGCACGACGGCCGGCGTAGAATCATGCAGGAAGGACTGATGAAGATTTAT

GTCCTCCAGCTTCTGGCCCGAGCTATTTTTGAGGTGGCTTTTCTCGGGGGCCAGTATCTT

CTATATGGCTTCAAGGTCAGCCCATCGTACATCTGCGCCAAGAACCCCTGCCCTCACCAG

GTGGACTGCTTTGTGTCCAGGCCCACGGAAAAGACCATCTTCCTCCTGATCATGTACGTG

GTCAGCTGCTTGTGCCTGTTGCTGAACATCTGTGAGATGTTCCACTTGGGTATCGGCAGC

ATCCGGGACATCATCCGCAACAGGAGGAACAAGGGCAAGCGCCCGTCCTACAGCTACCCC

TACCCCAGGAACATTCCTTCATCGCCCCCTGGGTATAACCTGGTGGTCAAGTCGGATAAA
[truncated: 374,406 more chars]
