# Supplementary material for: Connexins during 500 Million Years—From Cyclostomes to Mammals
Source: Int J Mol Sci. 2021 Feb 4;22(4):1584. doi: 10.3390/ijms22041584 (PMC7914757; doi:10.3390/ijms22041584)
Supplement: Supplementary file 1 [file ijms-22-01584-s001.zip › Additional File 2 Supplementary Figures S44-S71.pdf]

Supplementary Materials

Additional File 2: Phylogenetic trees and their branches

Supplementary Figures S44 - S71

Mikalsen, SO; í Kongsstovu, S; Tausen, M.
Connexins during 500 million years – from cyclostomes to mammals

Contents

Supplementary Figure S44. Expanded view of *GJA1*. 3
Supplementary Figure S45. Expanded view of *gja2* and *GJA3*. 4
Supplementary Figure S46. Expanded view of *GJA4*. 5
Supplementary Figure S47. Expanded view of *GJA5* and *gja14*. 6
Supplementary Figure S48. Expanded view of *GJA8*. 7
Supplementary Figure S49. Expanded view of *GJA9*. 8
Supplementary Figure S50. Expanded view of *GJA10*. 9
Supplementary Figure S51. Expanded view of *gja11*, *gja12* and *gja13*. 10
Supplementary Figure S52. Expanded view of *GJB1*. 11
Supplementary Figure S53. Expanded view of *GJB2* and *GJB6*. 12
Supplementary Figure S54. Expanded view of *GJB3*. 13
Supplementary Figure S55. Expanded view of *GJB4* and *GJB5*. 14
Supplementary Figure S56. Expanded view of *GJB7*. 15
Supplementary Figure S57. Expanded view of *gjb8*. 16
Supplementary Figure S58. Expanded view of *gjb9*. 16
Supplementary Figure S59. Expanded view of *gjb10*. 17
Supplementary Figure S60. Expanded view of *GJD1* and *GJD2*. 18
Supplementary Figure S61. Expanded view of *GJD3*. 19
Supplementary Figure S62. Expanded view of *GJD4*. 20
Supplementary Figure S63. Expanded view of *GJD5*. 20
Supplementary Figure S64. Expanded view of *gjd6*. 21
Supplementary Figure S65. Expanded view of *GJC1*. 21
Supplementary Figure S66. Expanded view of *GJC2*. 22
Supplementary Figure S67. Expanded view of *GJC3*. 23
Supplementary Figure S68. Expanded view of *gjc4*. 23
Supplementary Figure S69. Compressed phylogenetic tree including *GJE1* for all vertebrate groups except cyclostomes. 24
Supplementary Figure S70. Expanded view of *GJE1*. 25
Supplementary Figure S71. Compressed phylogenetic tree (including *GJE1*) for all vertebrate groups including cyclostomes. 26

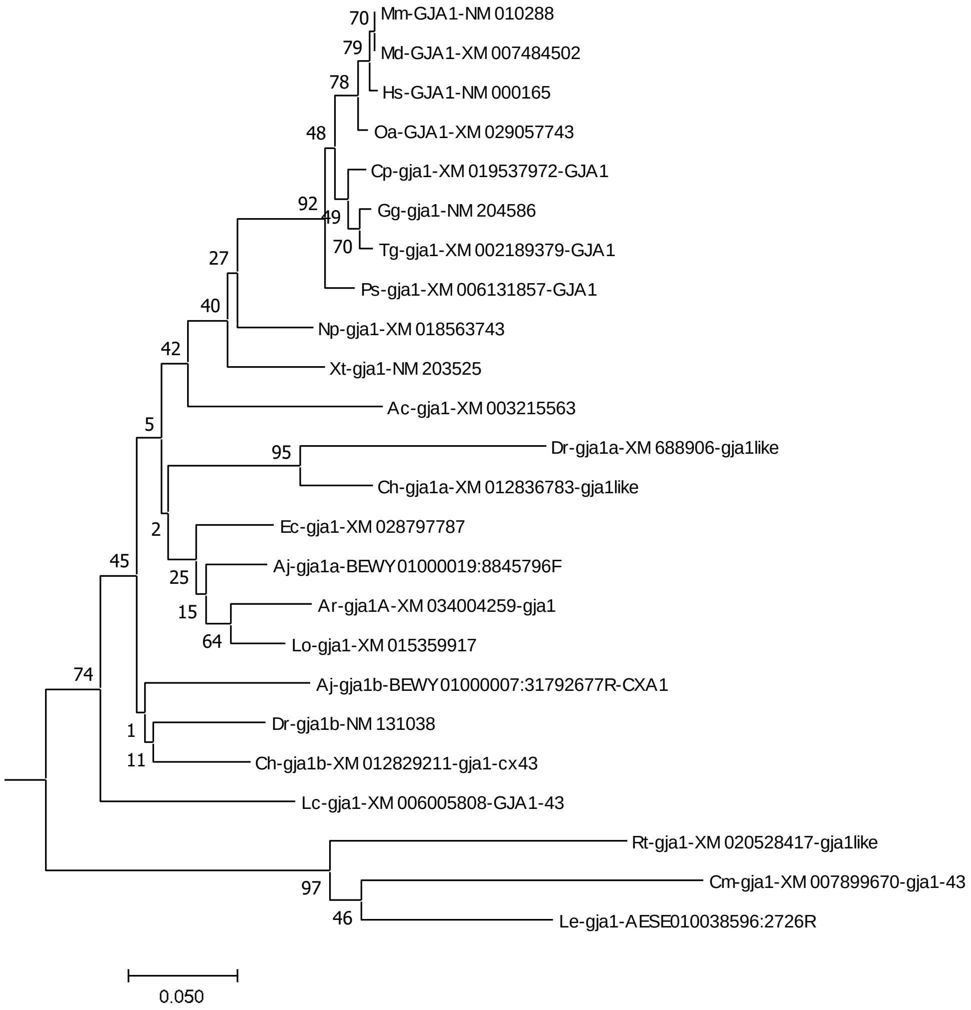

Supplementary Figure S44. Expanded view of *GJA1*.

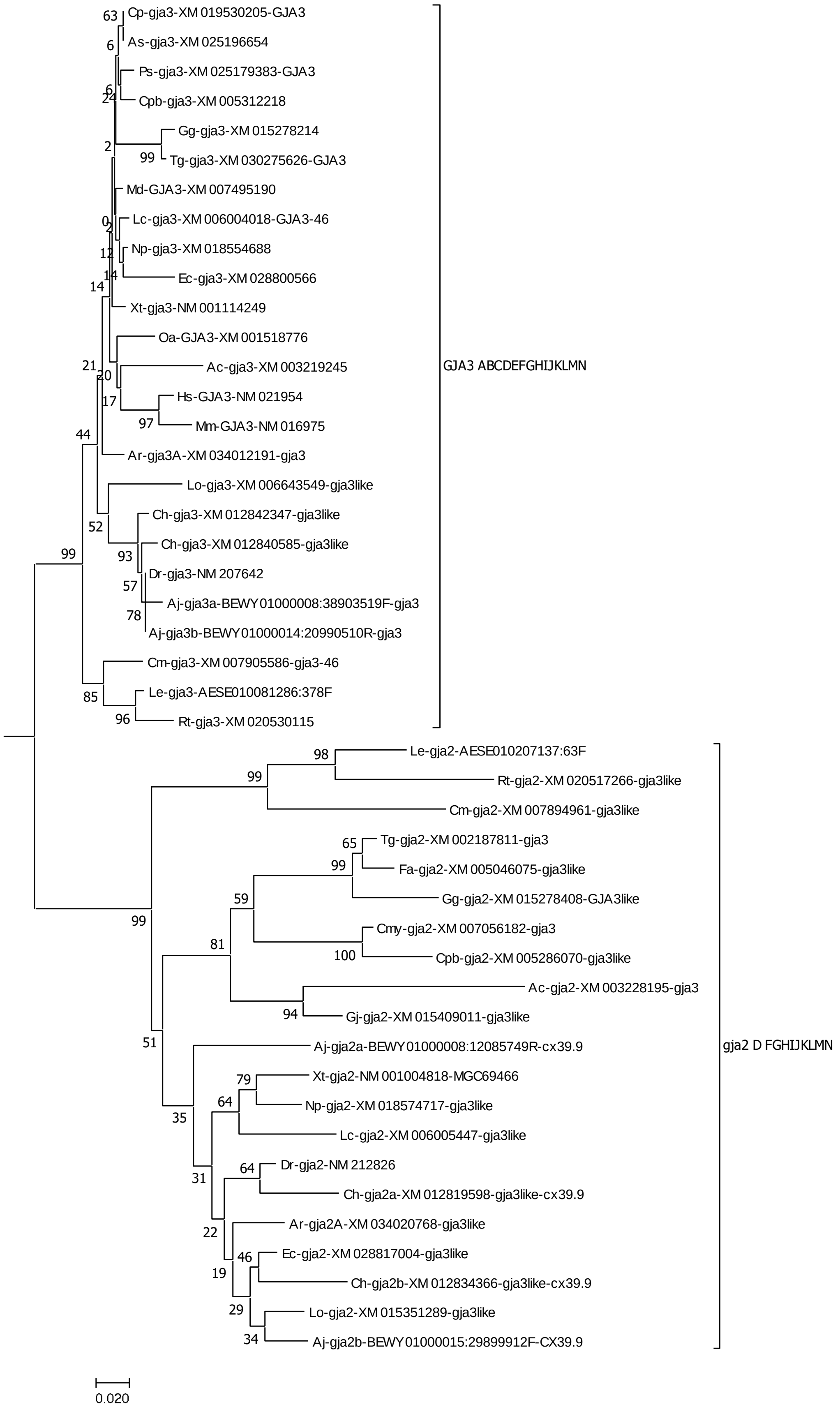

Supplementary Figure S45. Expanded view of *gja2* and *GJA3*.

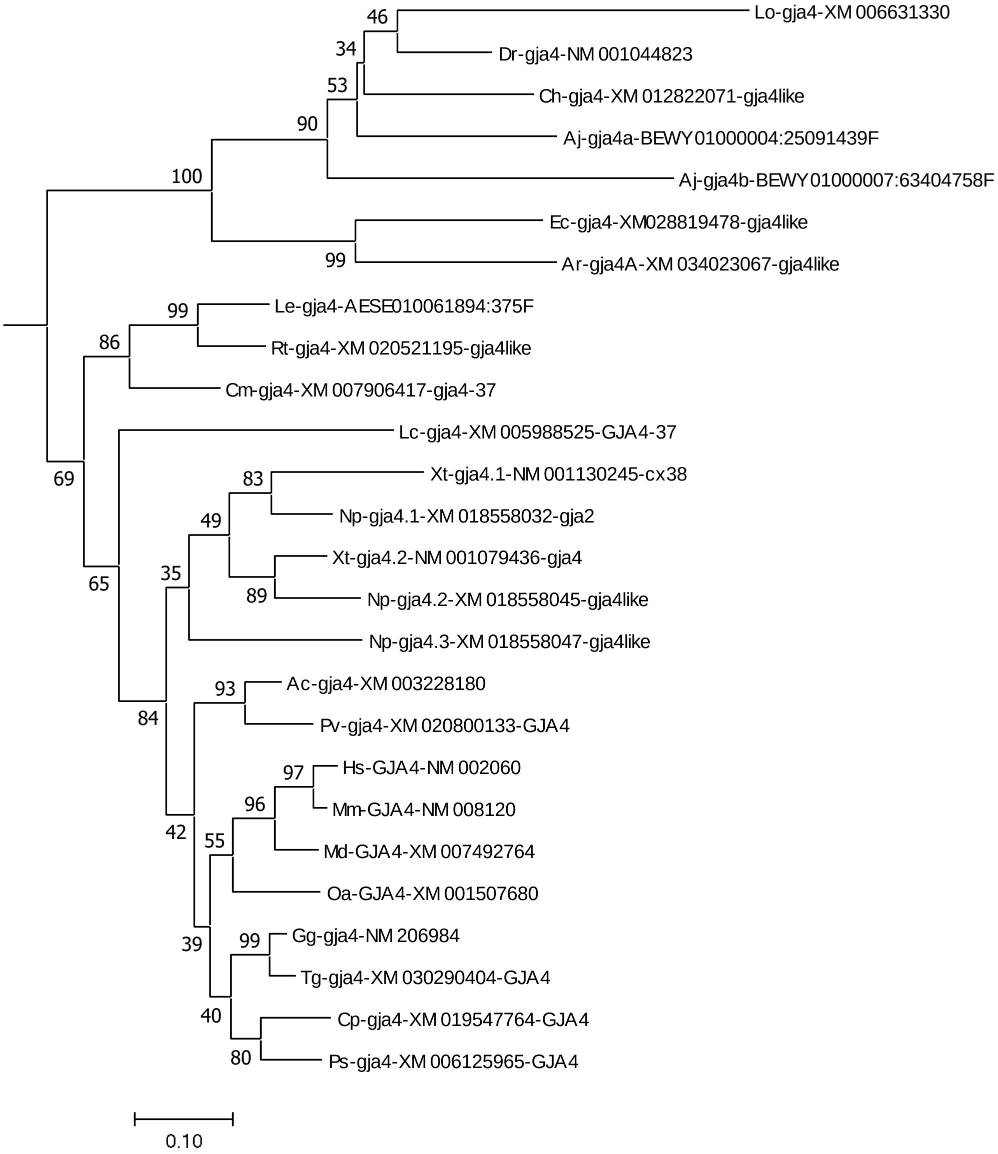

Supplementary Figure S46. Expanded view of *GJA4*.

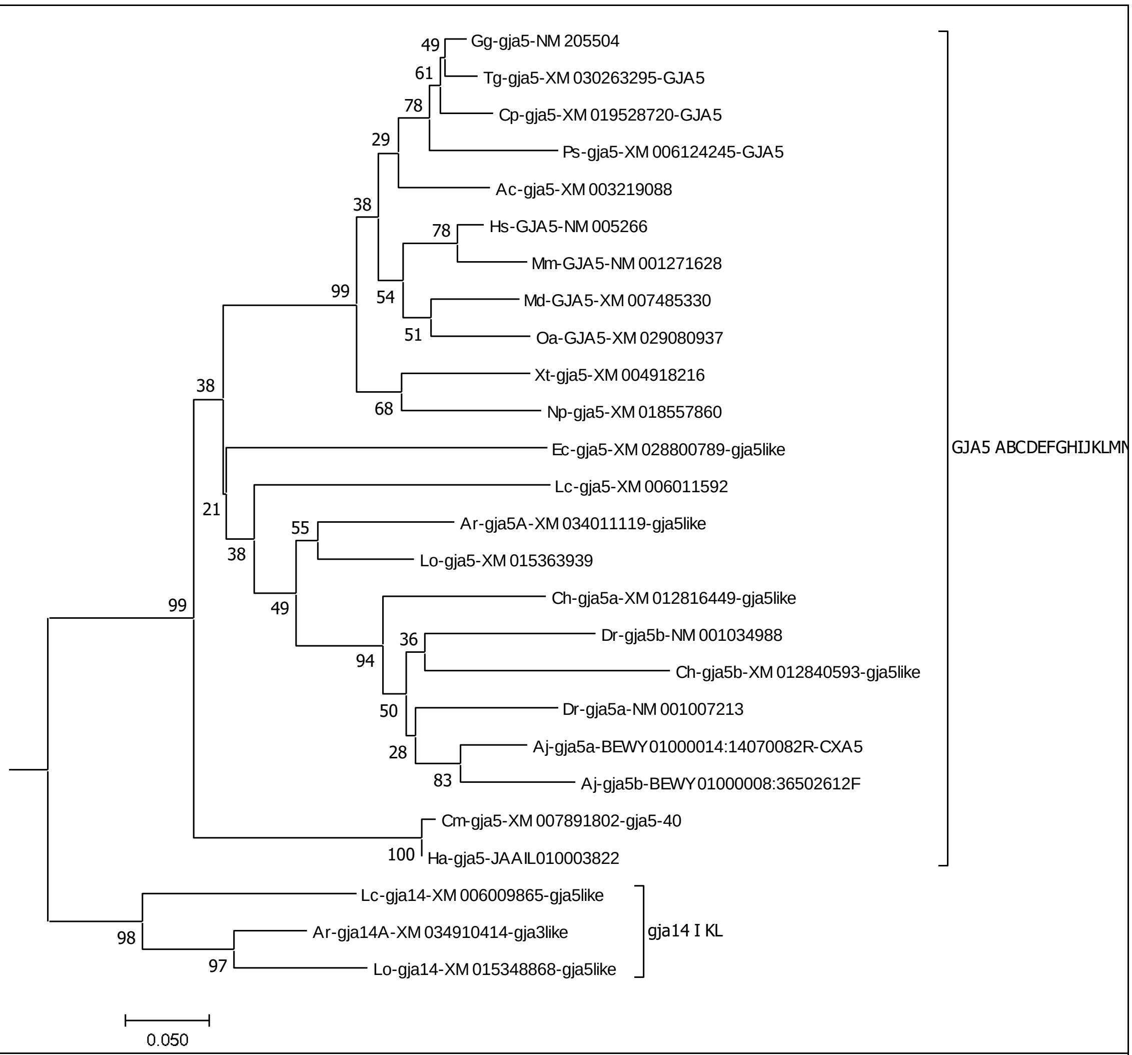

Supplementary Figure S47. Expanded view of *GJA5* and *gja14*.

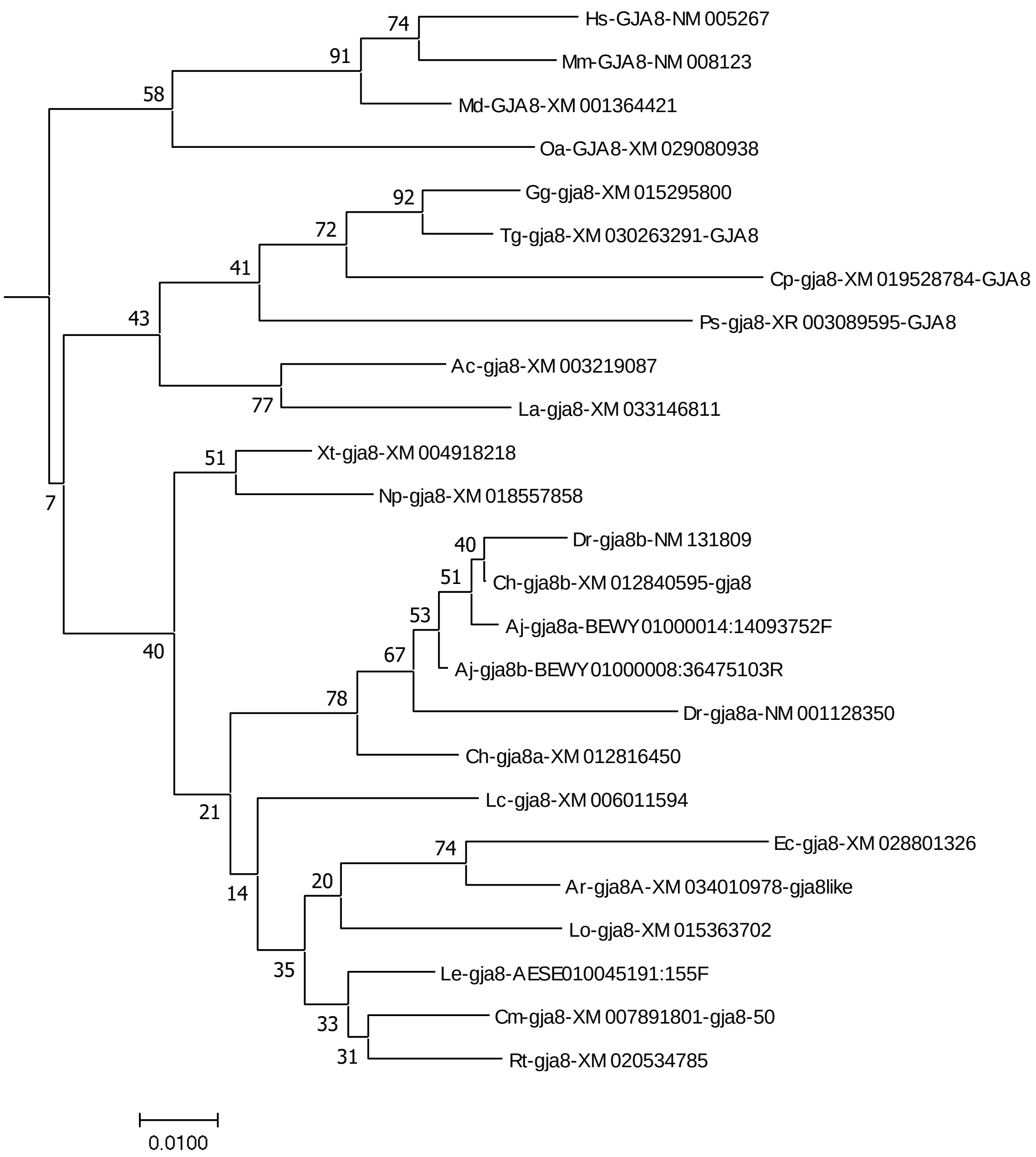

Supplementary Figure S48. Expanded view of *GJA8*.

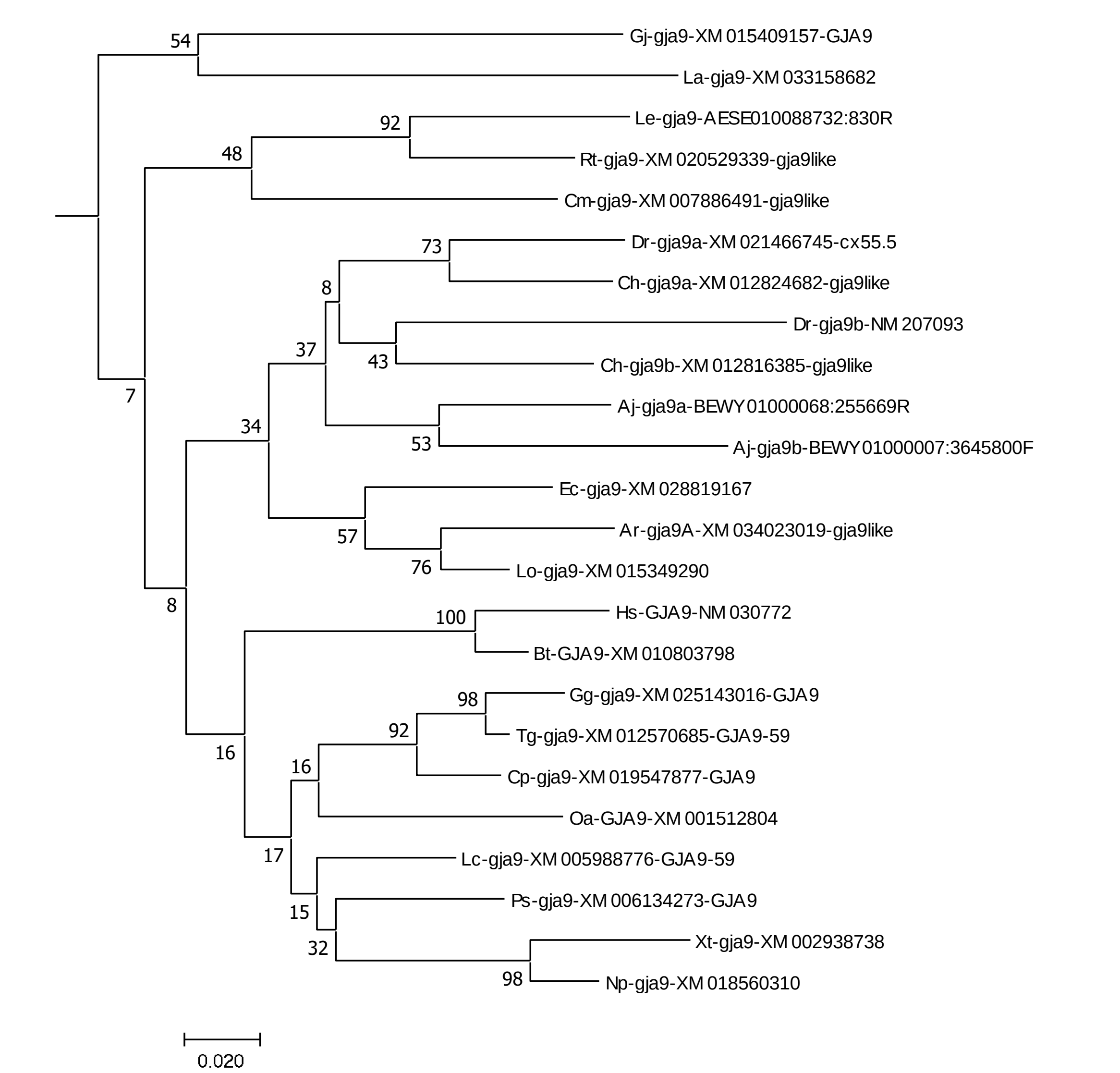

Supplementary Figure S49. Expanded view of *GJA9*.

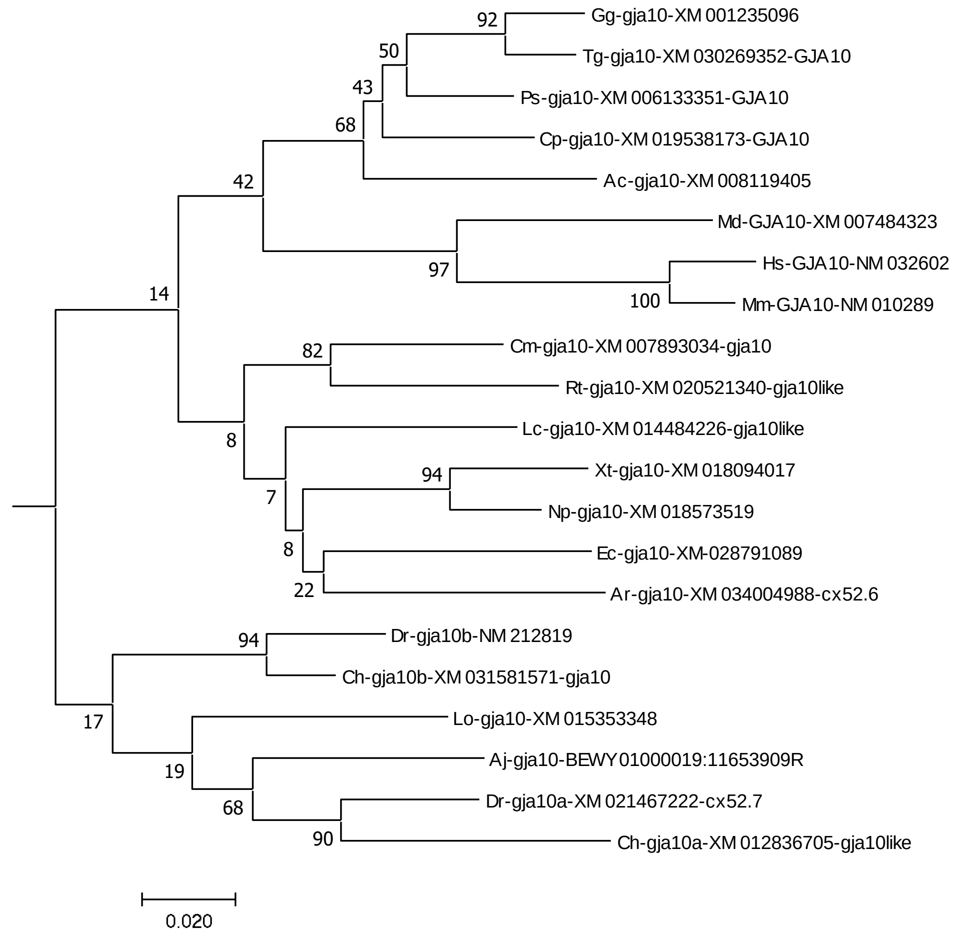

Supplementary Figure S50. Expanded view of *GJA10*.

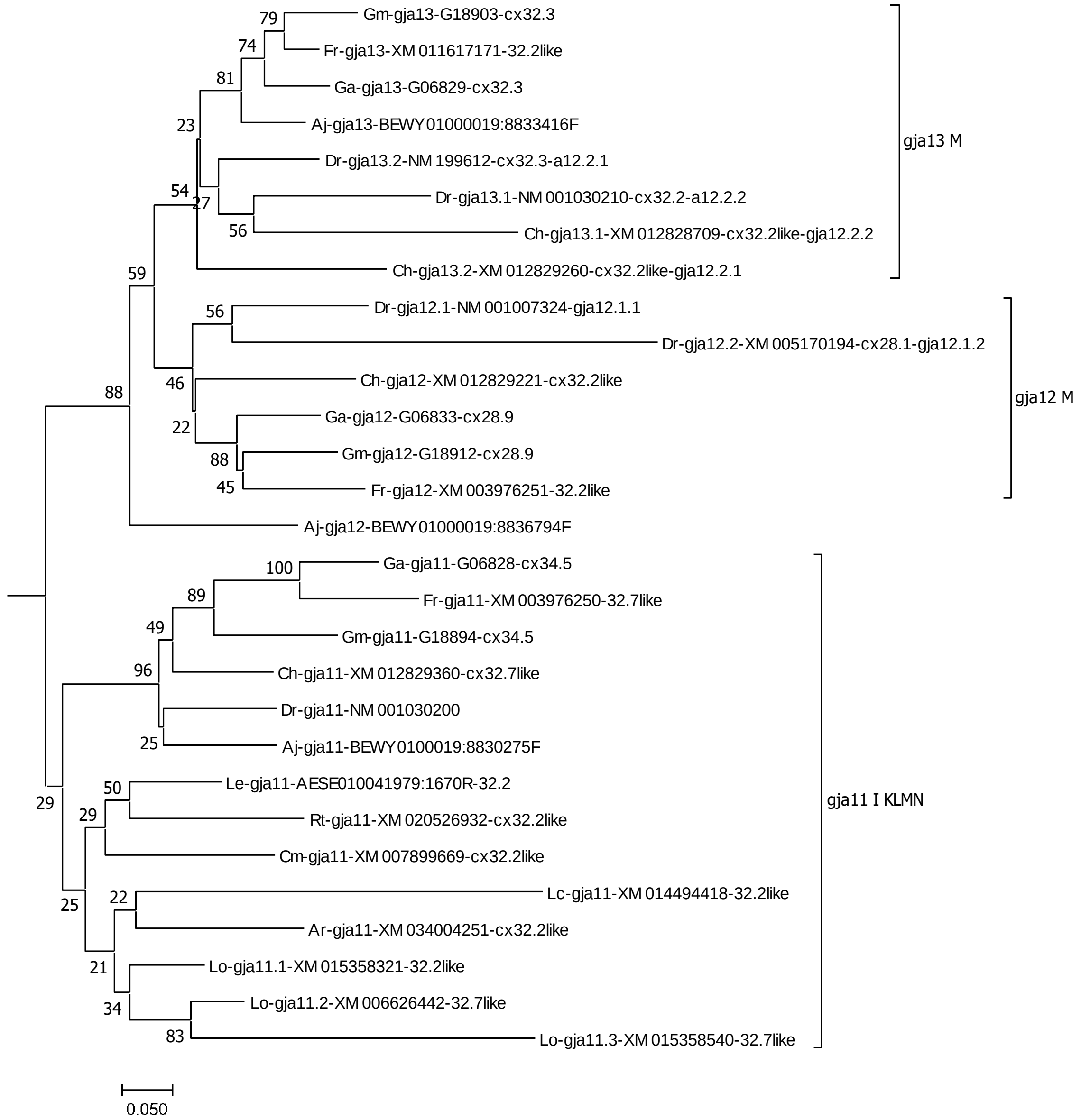

Supplementary Figure S51. Expanded view of *gja11*, *gja12* and *gja13*.

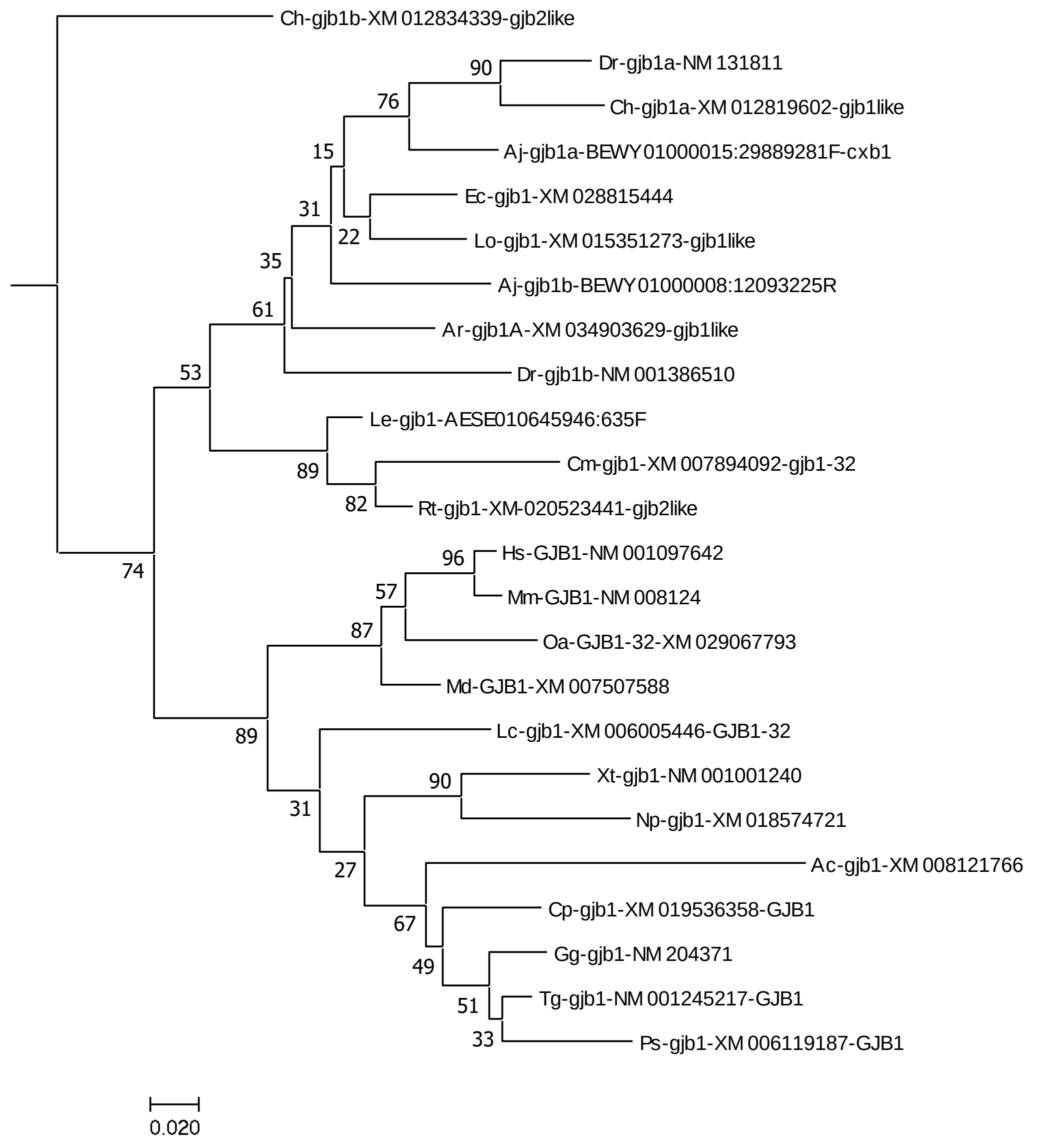

Supplementary Figure S52. Expanded view of *GJB1*.

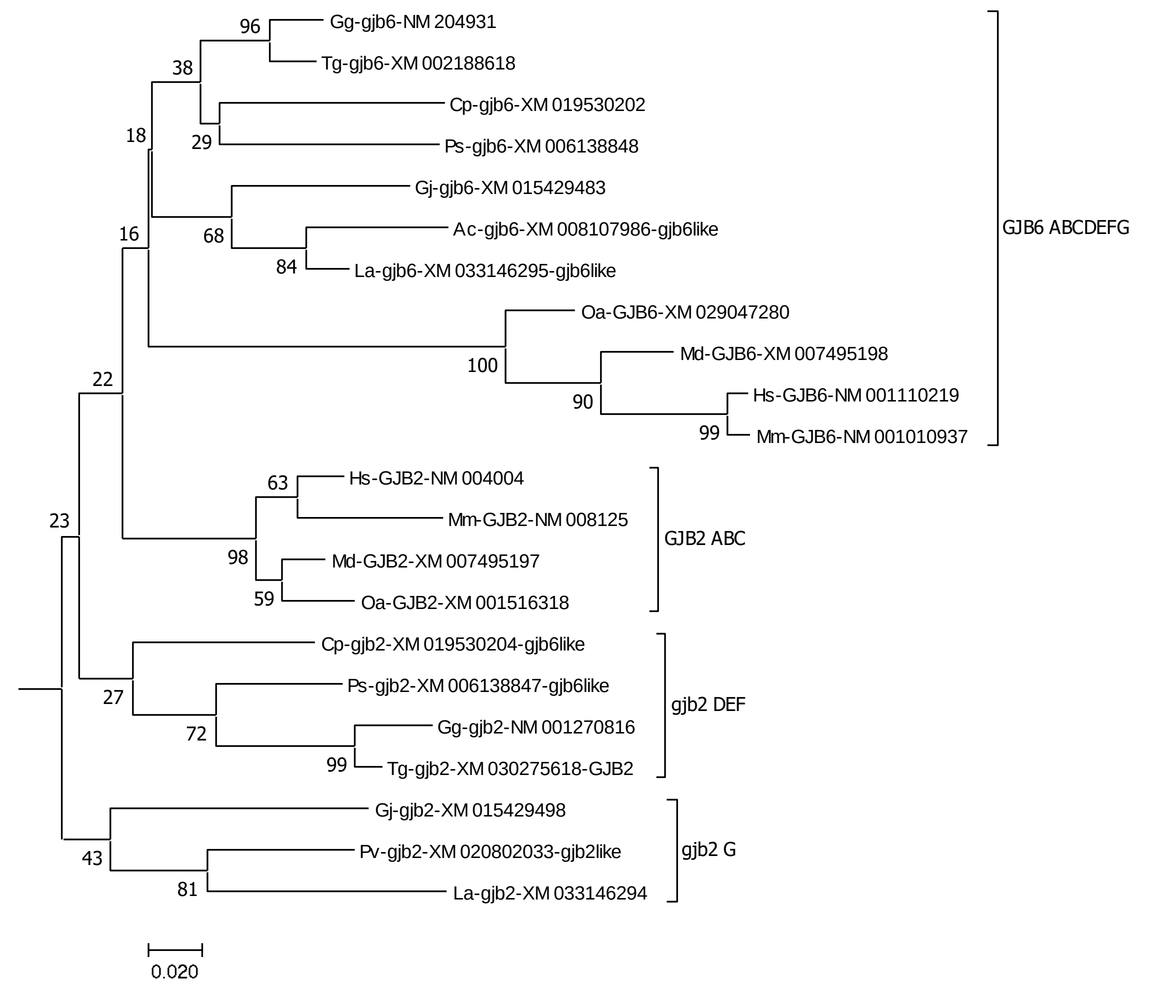

Supplementary Figure S53. Expanded view of *GJB2* and *GJB6*.

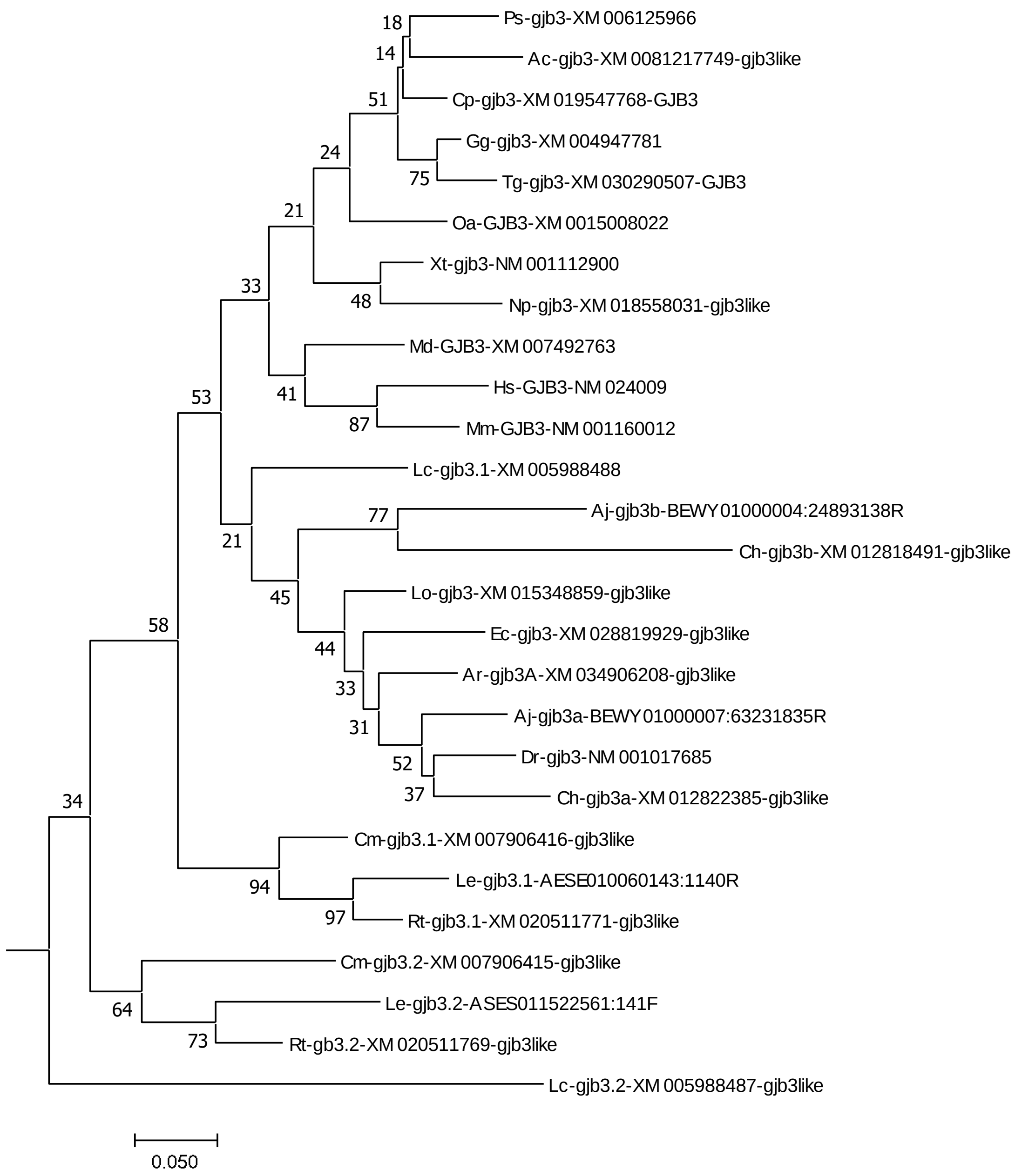

Supplementary Figure S54. Expanded view of *GJB3*.

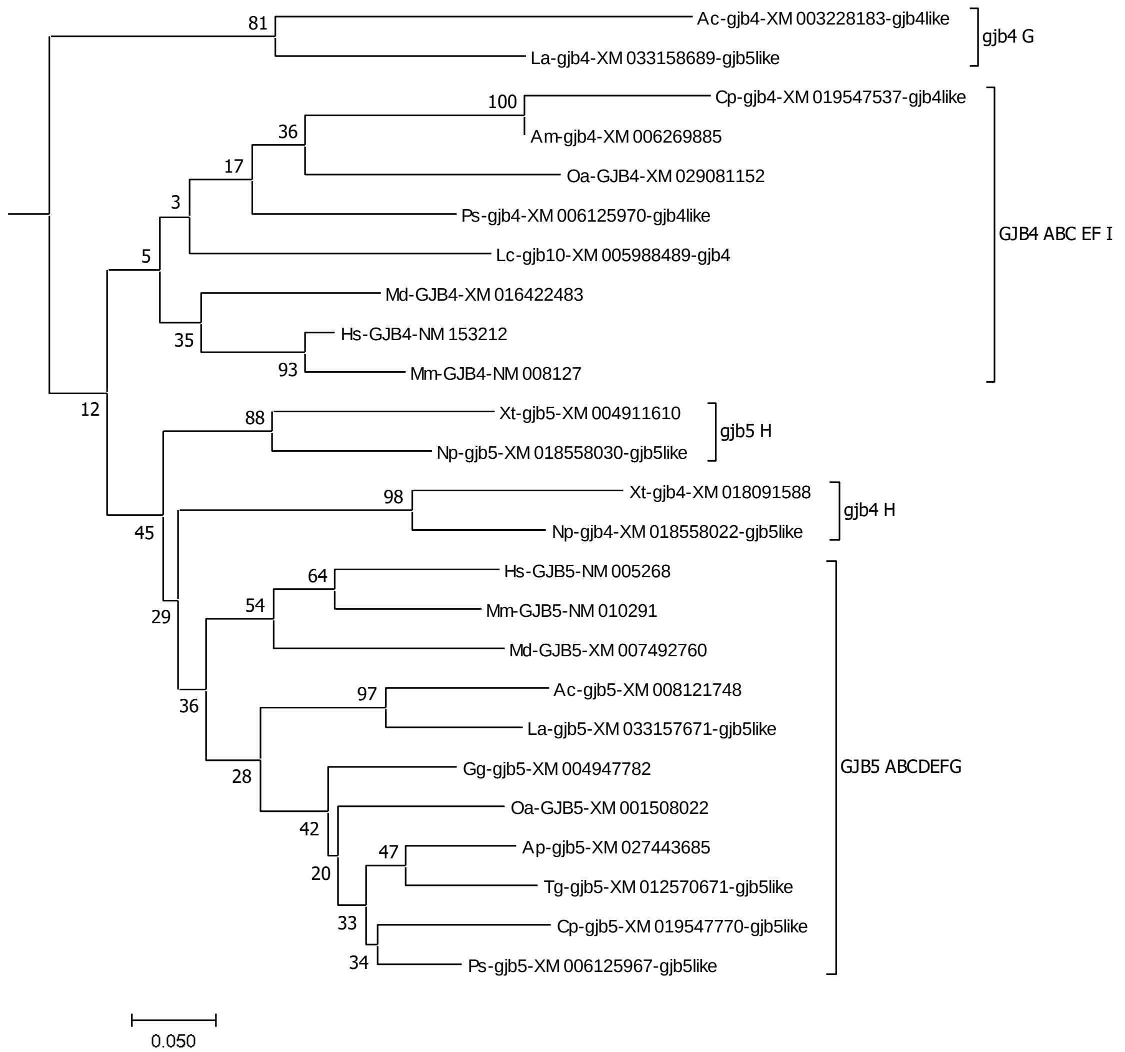

Supplementary Figure S55. Expanded view of *GJB4* and *GJB5*.

Note that Lc-gjb10-XM_005988489 in some models and when lower gamma value was used, either located together with chondrichthyan *gjb10*, with the larger *GJB3* orthogroup, or as a singleton. Further note that the amphibian sequences indicated by gjb5 H and gjb4 H tended to inverse their location depending on whether the model was run with amino acids or nucleotides. We have here used the same names on the *Xenopus* sequences as in GenBank, but synteny considerations suggests that the names should be reversed, *i.e.*, *Xenopus* XM_004911610 should be named *gjb4* and XM_018091588 should be named *gjb5* (see text), and similarly for the *Nanorana* sequences.

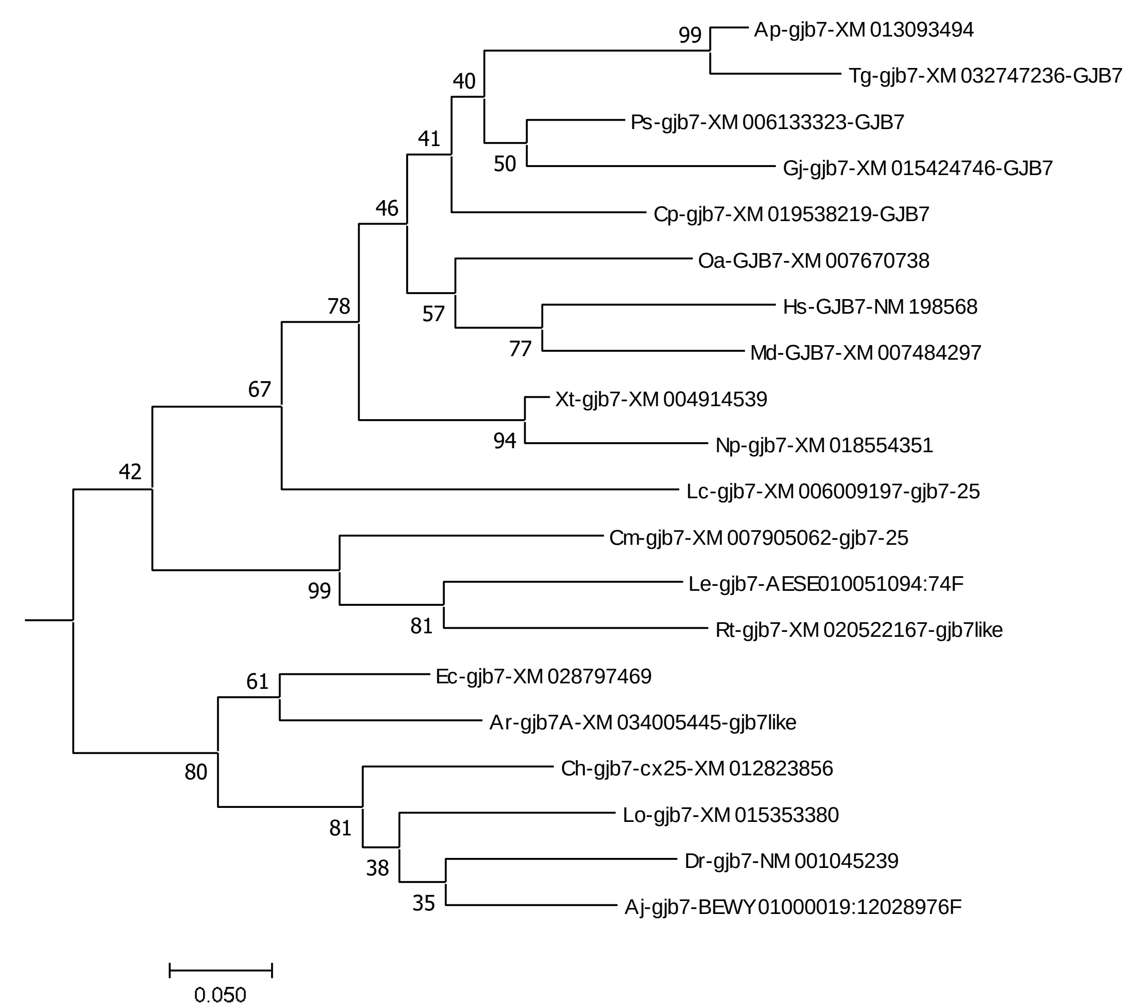

Supplementary Figure S56. Expanded view of *GJB7*.

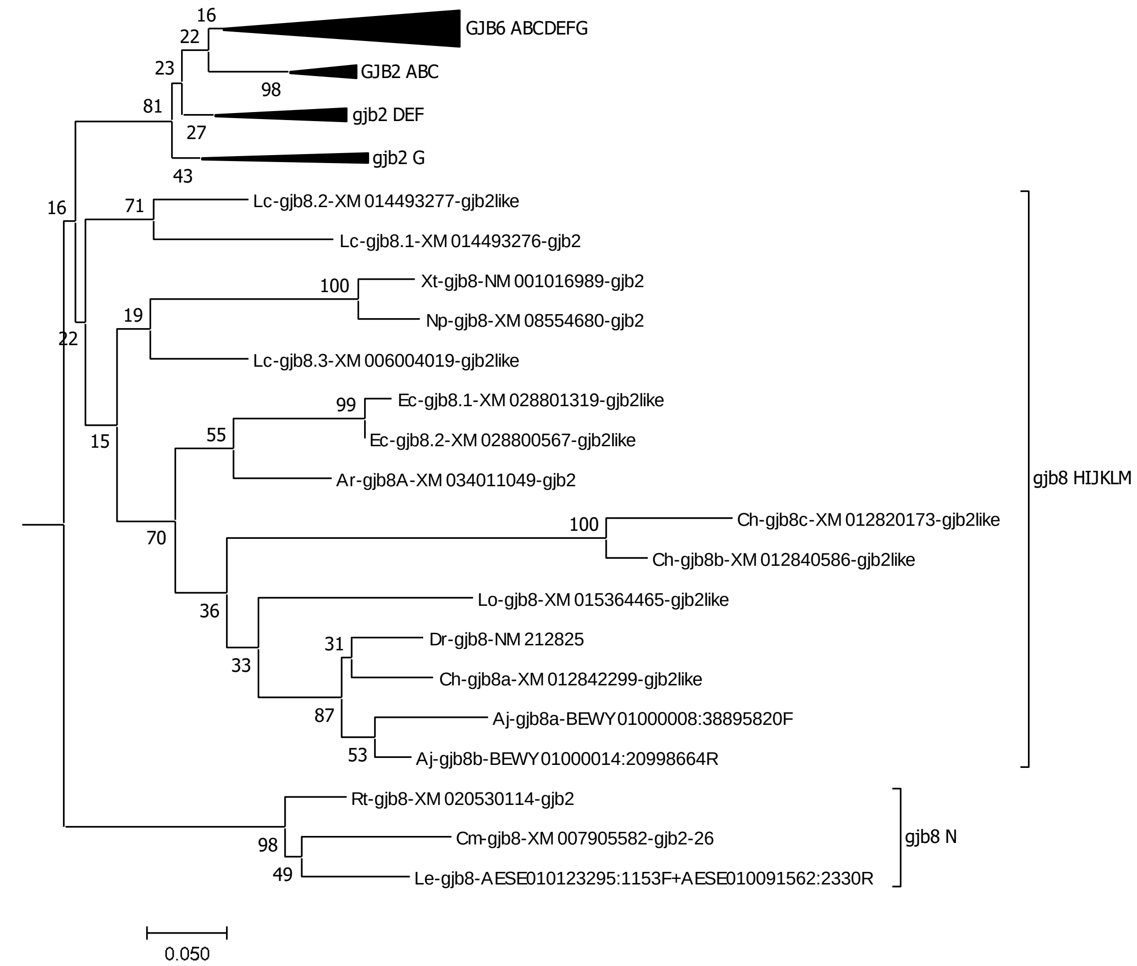

Supplementary Figure S57. Expanded view of *gjb8*.

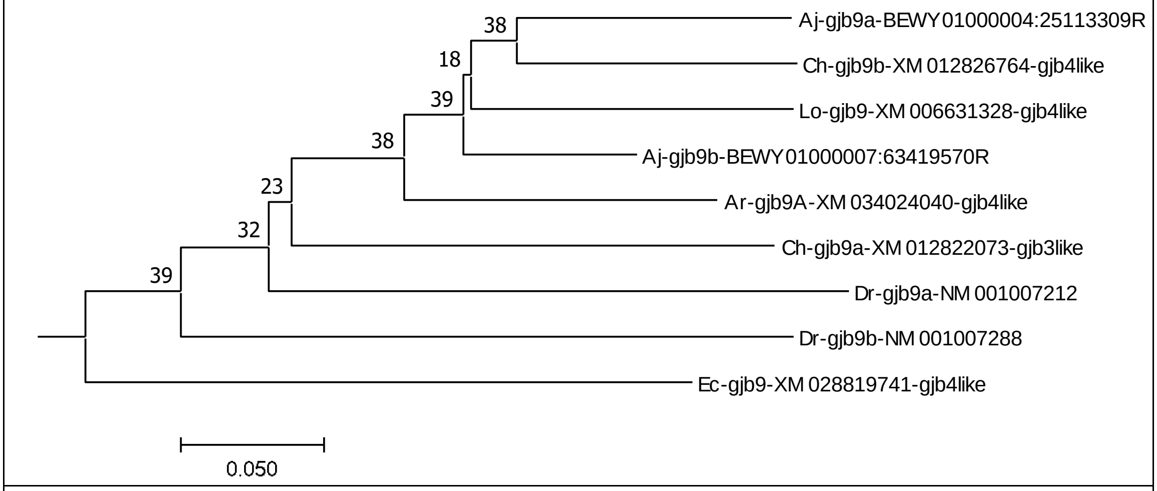

Supplementary Figure S58. Expanded view of *gjb9*.

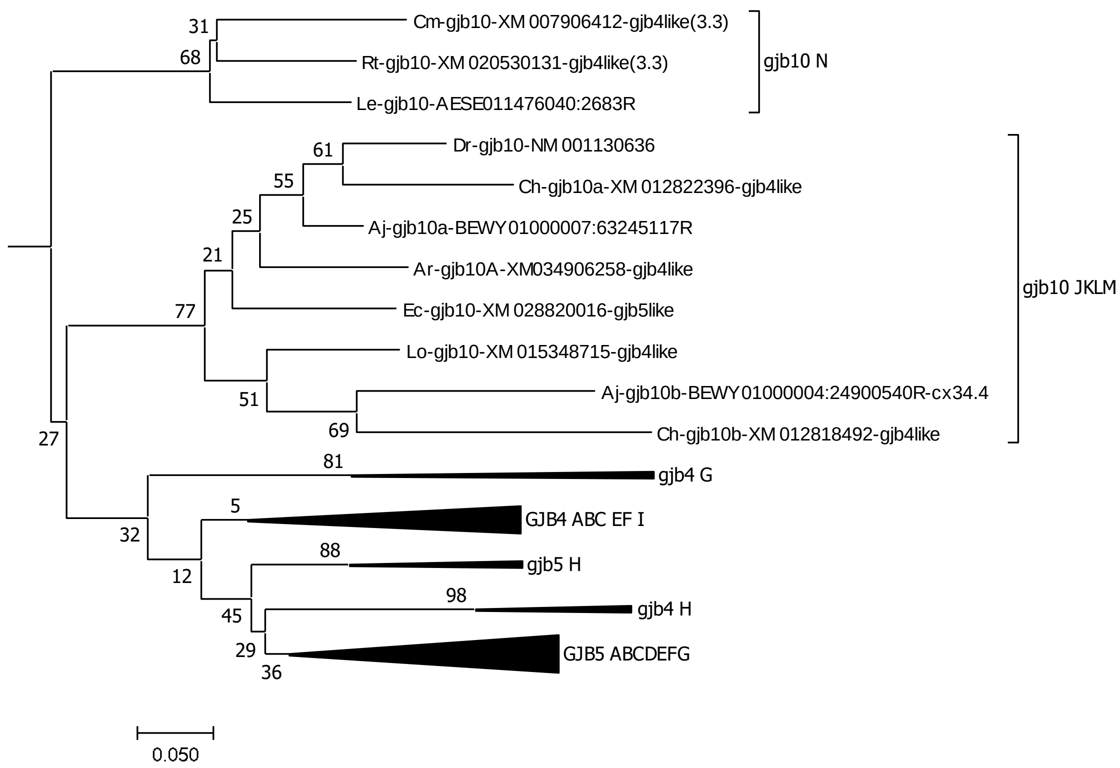

Supplementary Figure S59. Expanded view of *gjb10*.

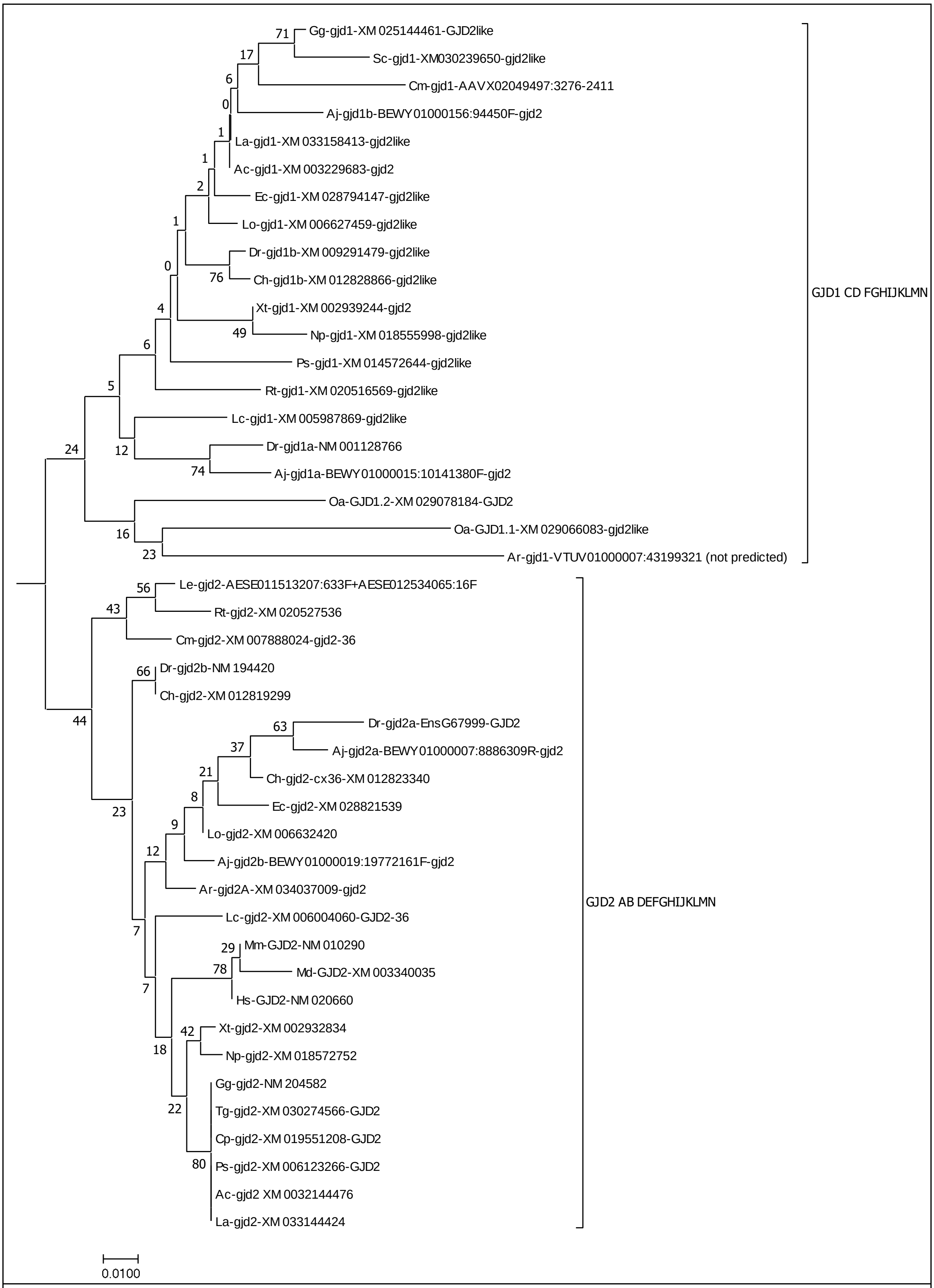

Supplementary Figure S60. Expanded view of *GJD1* and *GJD2*.

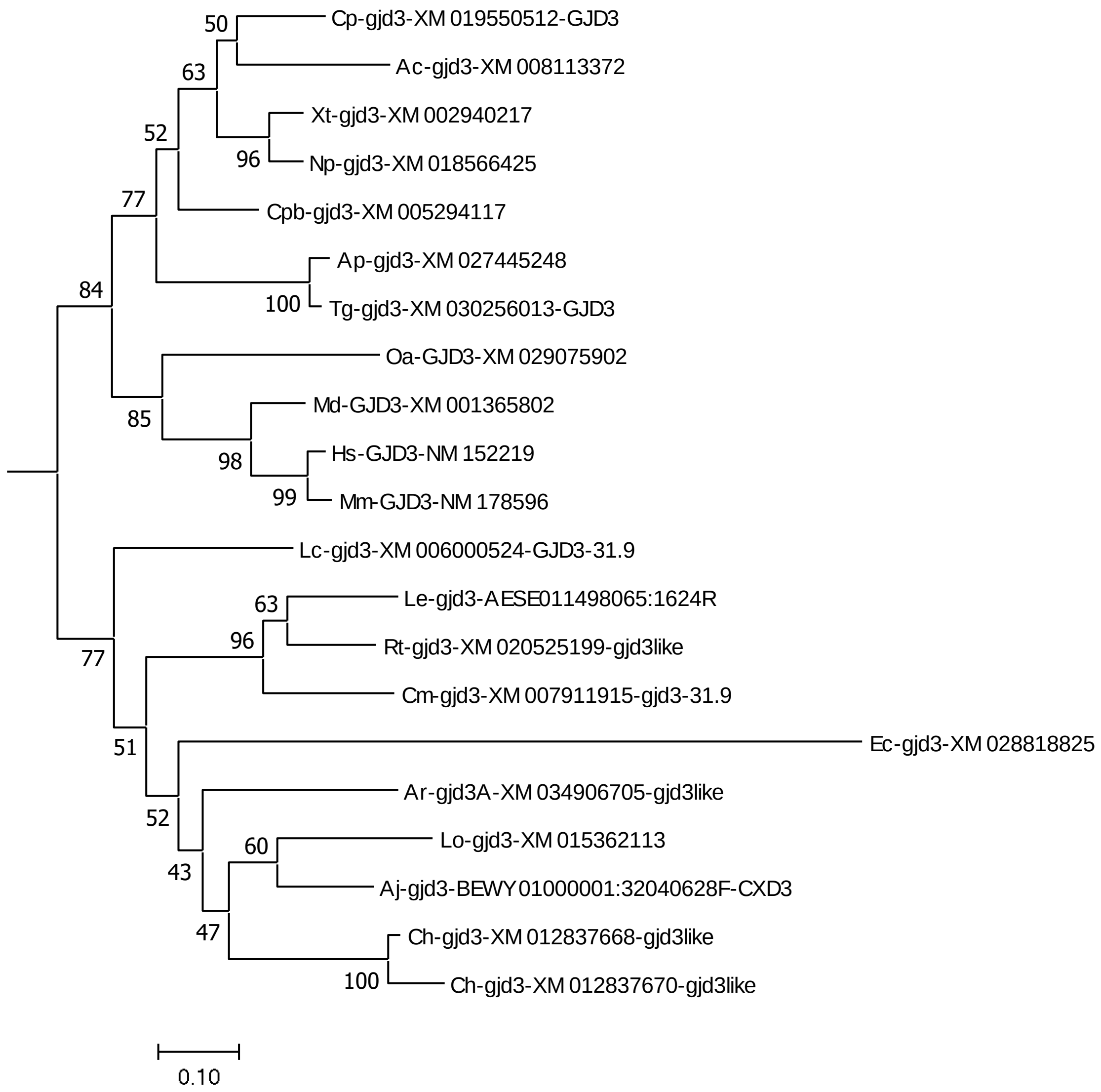

Supplementary Figure S61. Expanded view of *GJD3*.

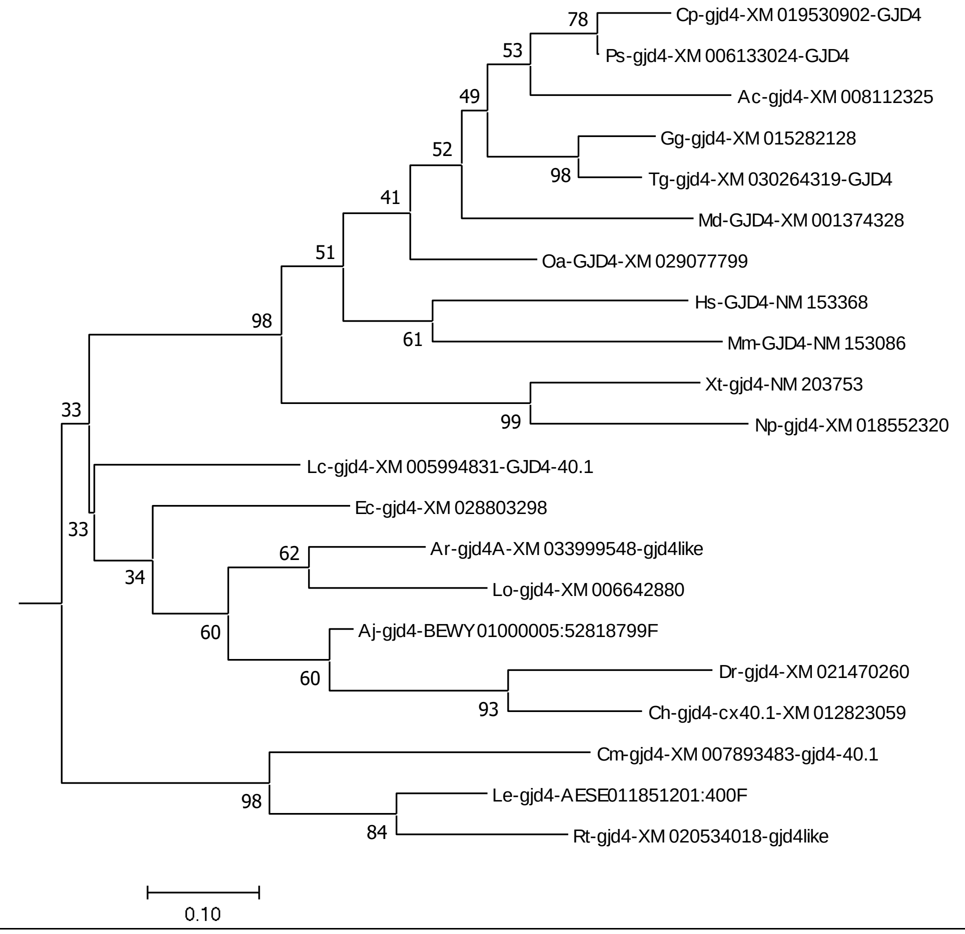

Supplementary Figure S62. Expanded view of *GJD4*.

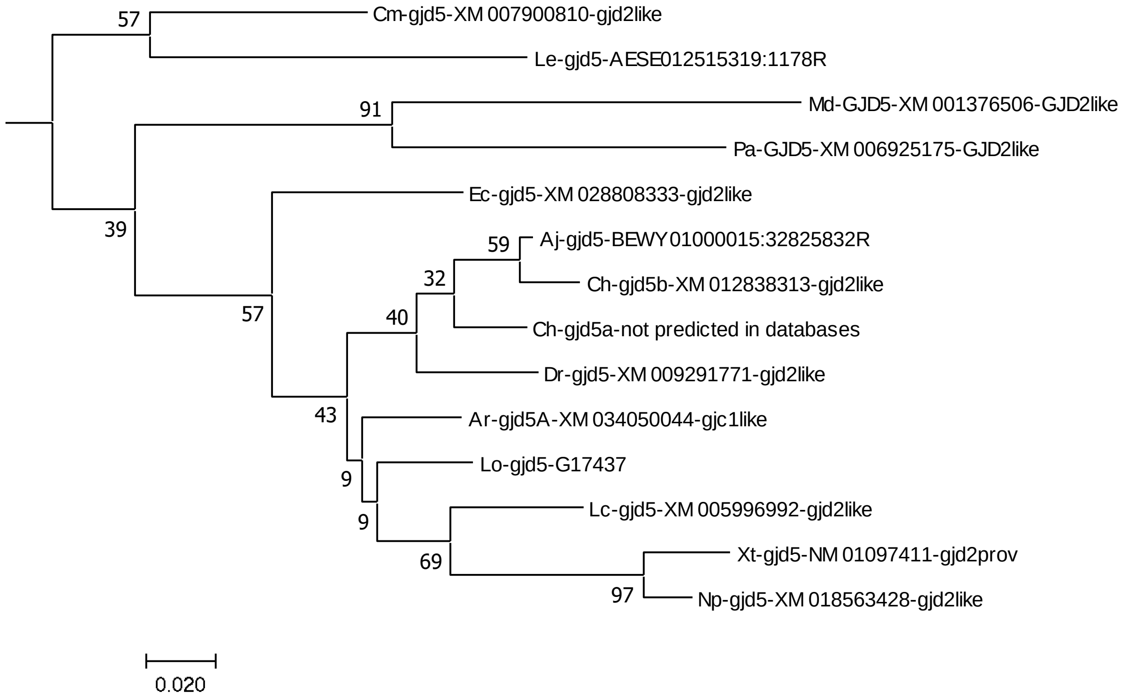

Supplementary Figure S63. Expanded view of *GJD5*.

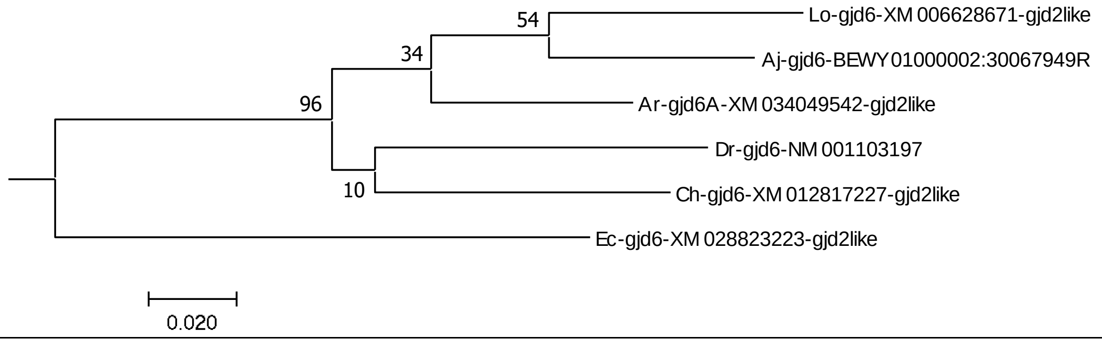

Supplementary Figure S64. Expanded view of *gjd6*.

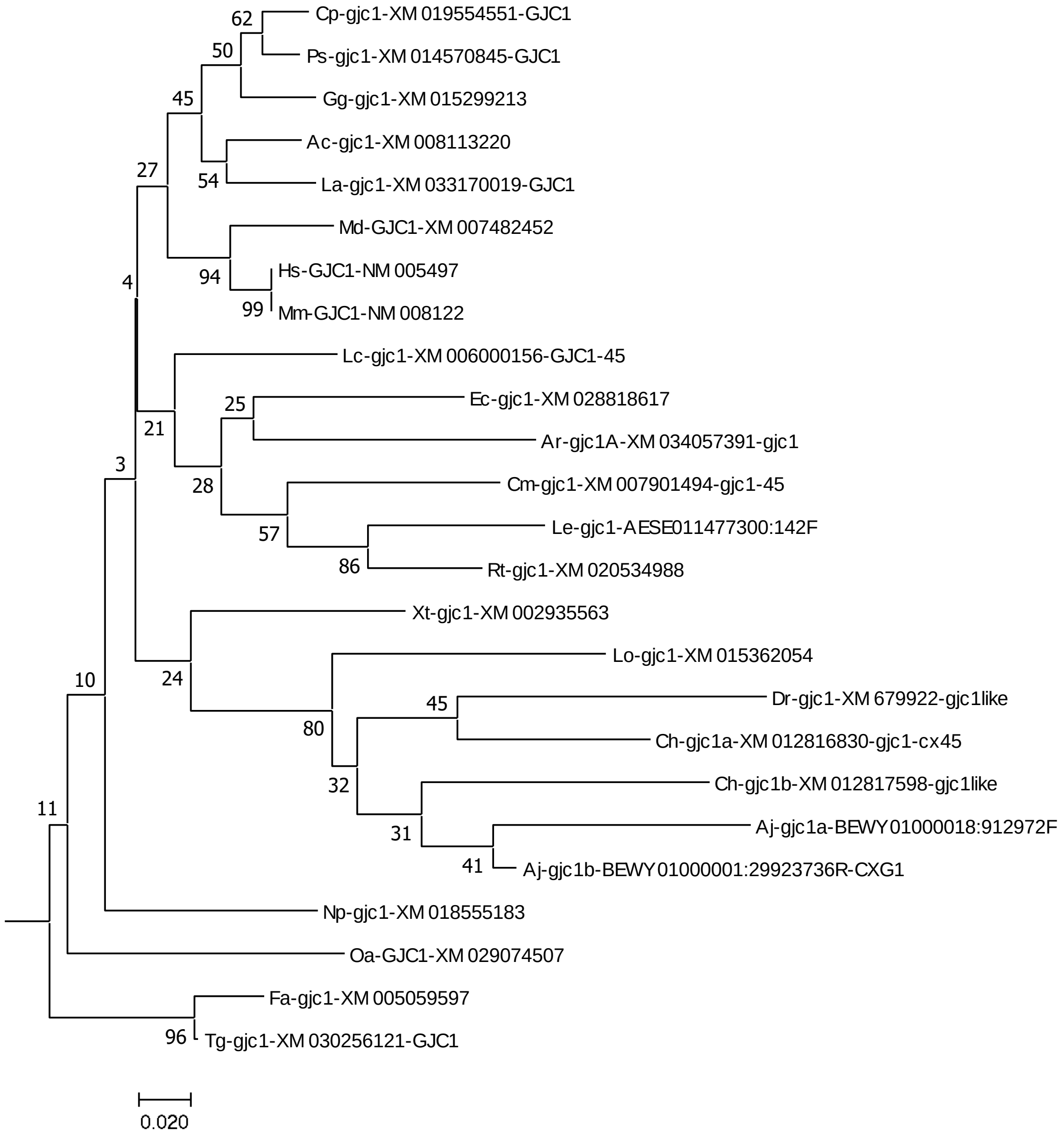

Supplementary Figure S65. Expanded view of *GJC1*.

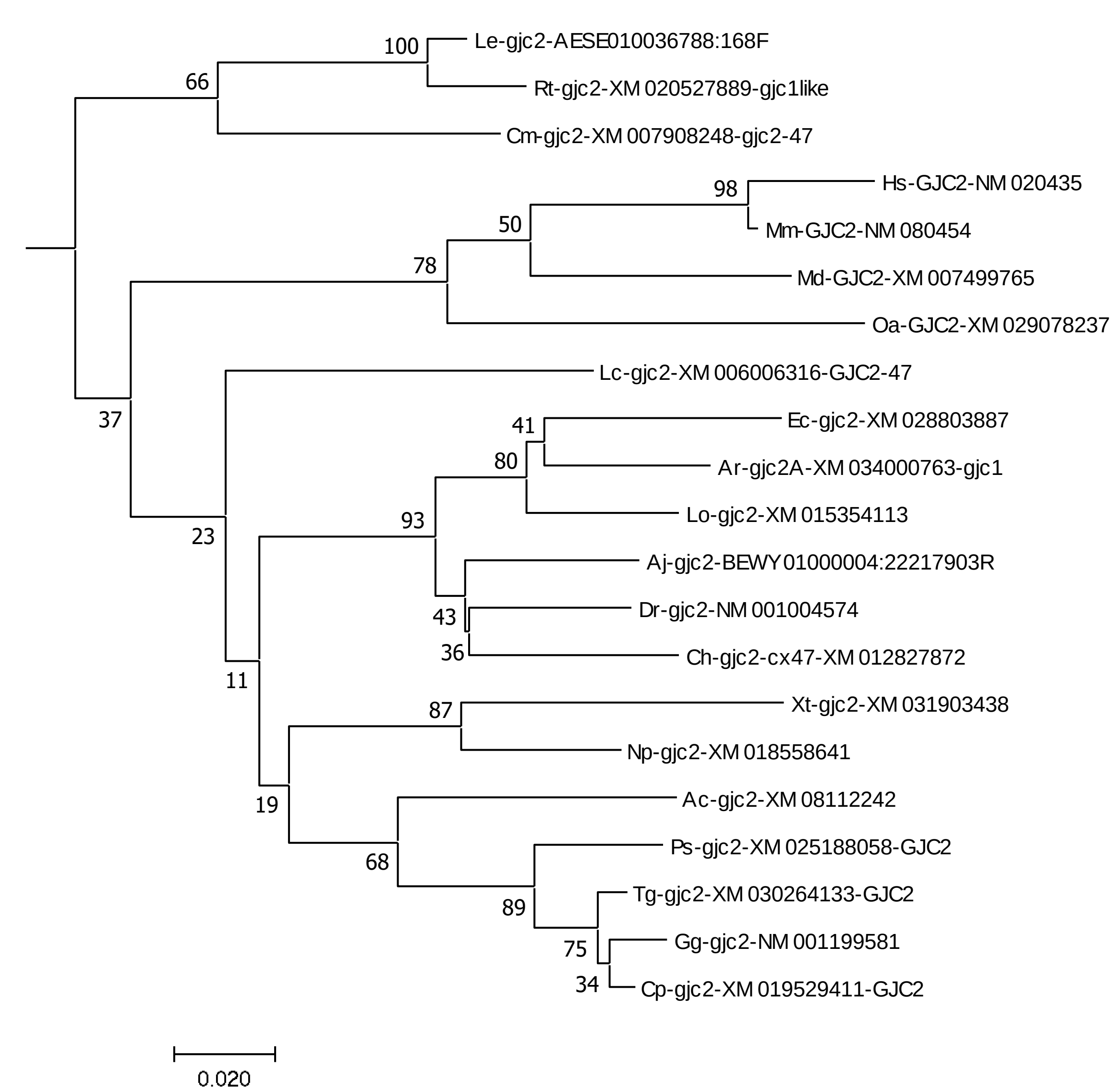

Supplementary Figure S66. Expanded view of *GJC2*.

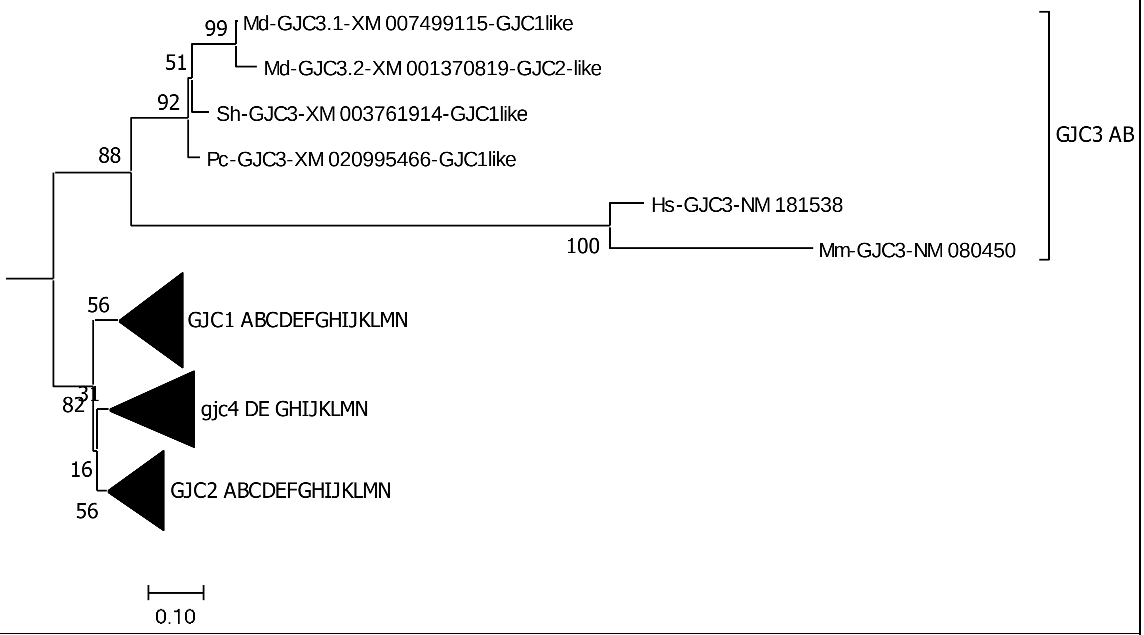

Supplementary Figure S67. Expanded view of *GJC3*.

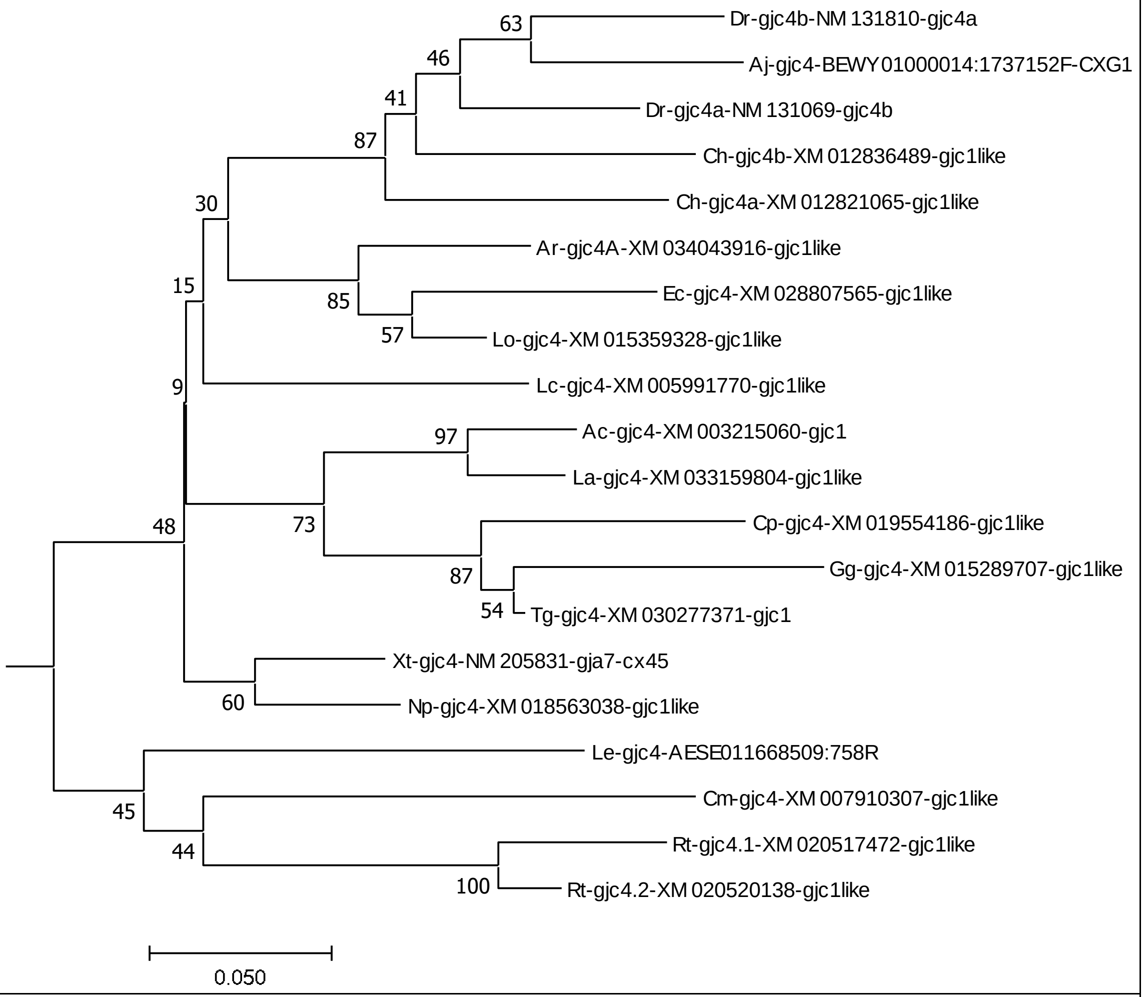

Supplementary Figure S68. Expanded view of *gjc4*.

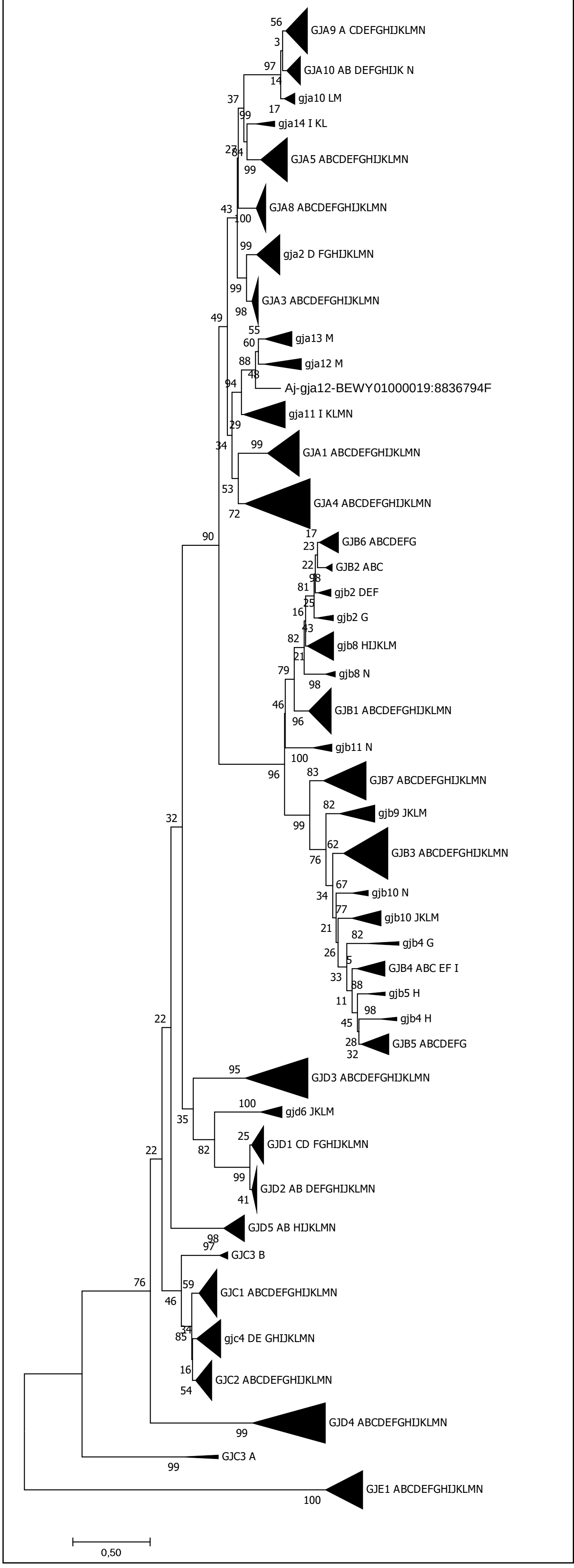

Supplementary Figure S69. Compressed phylogenetic tree including *GJE1* for all vertebrate groups except cyclostomes. The orthologous gene groups are indicated by their Greek nomenclature, using upper case letters if mammalian sequences are included in the group, and lower case letters if mammalian sequences are not included in the group. The letters at each branch signify the vertebrate groups as follows: A, Placental mammals; B, Marsupials; C, Monotremata (platypus); D, Aves (birds); E, Crocodylians; F, Testudines (turtles); G, Squamata (lizards and snakes); H, Amphibia; I, Lobe-finned fish (*Latimeria*); J, Chondrostei - Polypteriformes (reedfish); K, Chondrostei - Acipenseriformes (sturgeons); L, Holostei (spotted gar); M, Teleostei; N, Chondrichthyes (cartilaginous fishes). A space is left in the sequence of the letters to make it more evident that one or more groups are lacking that particular gene. The tree was constructed on basis of the amino acid sequence (205 positions, 579 sequences) of the conserved domains by the Neighbor-Joining method using JTT substitution matrix and with rate variation among sites corresponding to a gamma shape parameter = 1. Five hundred bootstrap iterations were run.

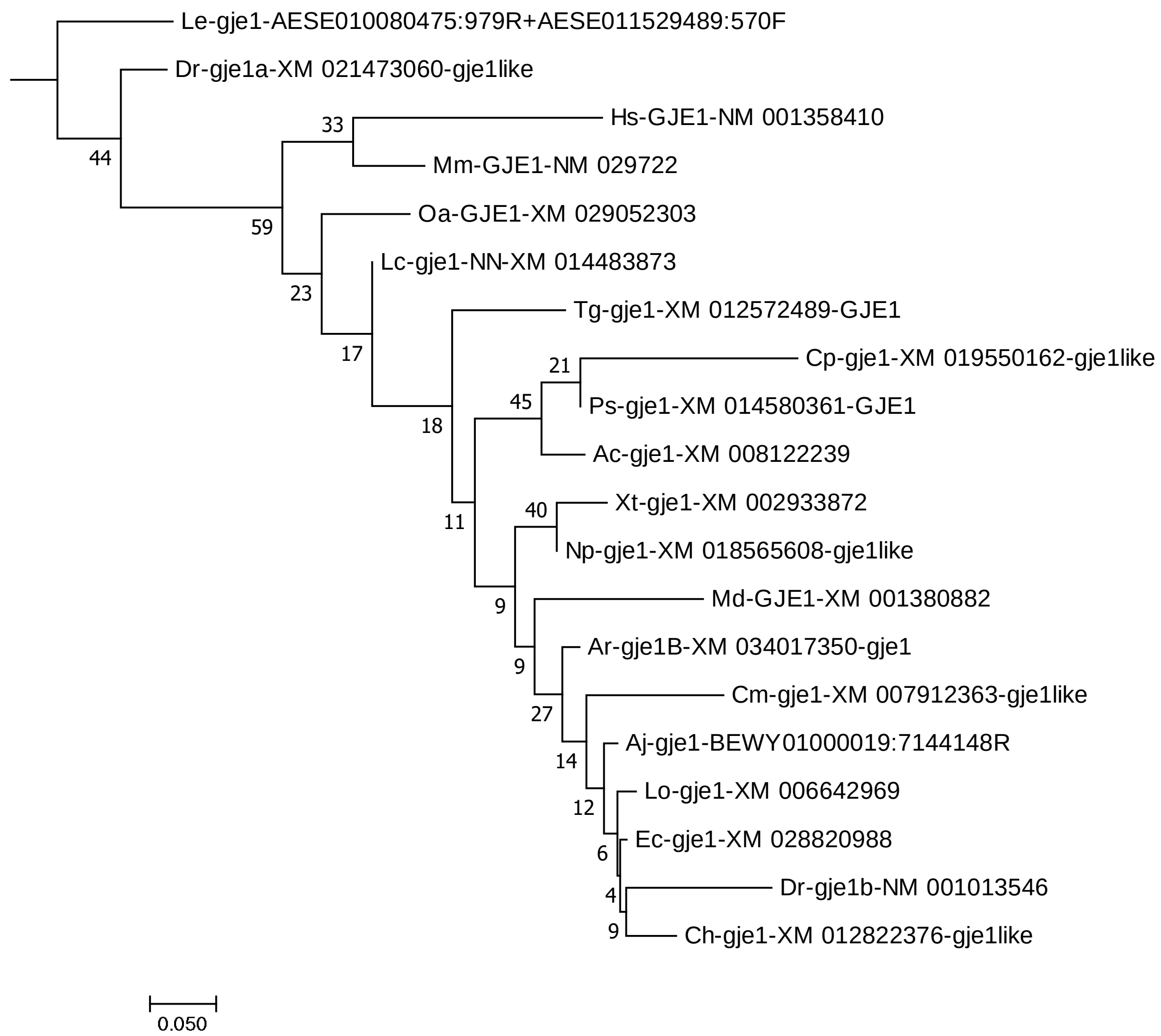

Supplementary Figure S70. Expanded view of *GJE1*.

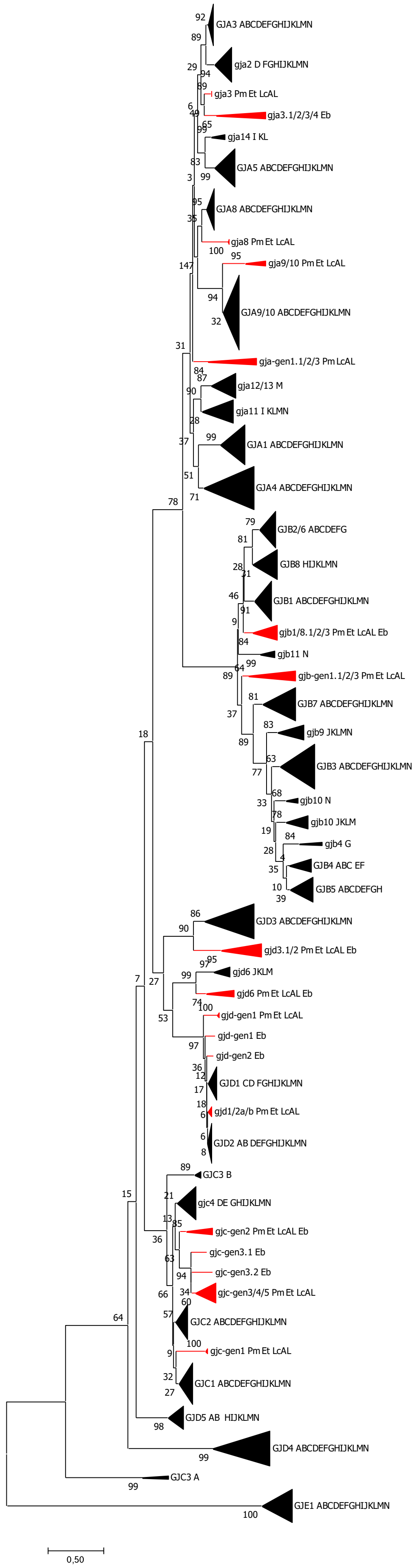

Supplementary Figure S71. Compressed phylogenetic tree (including *GJE1*) for all vertebrate groups including cyclostomes. The orthologous gene groups are indicated by their Greek nomenclature, using upper case letters if mammalian sequences are included in the group, and lower case letters if mammalian sequences are not included in the group. The letters at each branch signify the vertebrate groups as follows: A, Placental mammals; B, Marsupials; C, Monotremata (platypus); D, Aves (birds); E, Crocodylians; F, Testudines (turtles); G, Squamata (lizards and snakes); H, Amphibia; I, Lobe-finned fish (*Latimeria*); J, Chondrostei - Polypteriformes (reedfish); K, Chondrostei - Acipenseriformes (sturgeons); L, Holostei (spotted gar); M, Teleostei; N, Chondrichthyes (cartilaginous fishes).
A space is left in the sequence of the letters to make it more evident that one or more groups are lacking that particular gene. The cyclostomal branches are indicated in red. The cyclostome sequences are indicated with their preliminary gene name (defined as described in the text) followed by the abbreviation describing the cyclostome species: Pm, *Petromyzon marinus* - sea lamprey; Et, *Entosphenus tridentatus*, Pacific lamprey; LcAL, *Lethentheron camtschaticum*, Arctic lamprey, Eb, *Eptatretus burgeri*, inshore hagfish.
The tree was constructed on basis of the amino acid sequence (205 positions, 655 sequences) of the conserved domains by the Neighbor-Joining method using JTT substitution matrix and with rate variation among sites corresponding to a gamma shape parameter = 0.8. Five hundred bootstrap iterations were run.
