# Supplementary material for: Connexins during 500 Million Years—From Cyclostomes to Mammals
Source: Int J Mol Sci. 2021 Feb 4;22(4):1584. doi: 10.3390/ijms22041584 (PMC7914757; doi:10.3390/ijms22041584)
Supplement: Supplementary file 1 [file ijms-22-01584-s001.zip › Additional File 3 Supplementary Tables S1-S8.docx]

**Supplementary Materials**

**Additional File 3: Tables**

**Supplementary Tables S1 – S8**

**Mikalsen, SO; í Kongsstovu, S; Tausen, M.**

**Connexins during 500 million years – from cyclostomes to mammals**

Contents

[Supplementary Table S1. Overview of statistics for clade unity. 2](#_Toc60321969)

[Supplementary Table S2. Eel connexin ohnologs. 2](#_Toc60321970)

[Supplementary Table S3. Atlantic herring connexin ohnologs. 7](#_Toc60321971)

[Supplementary Table S4. Zebrafish connexin ohnologs. 8](#_Toc60321972)

[Supplementary Table S5. Sterlet sturgeon connexins and their ohnologs. 9](#_Toc60321973)

[Supplementary Table S6. Chromosomal locations of connexin genes in cartilaginous fishes. 11](#_Toc60321974)

[Supplementary Table S7. Chromosomal locations of connexin genes in cyclostomes. 13](#_Toc60321975)

[Supplementary Table S8. Total connexin overview in the analyzed vertebrates. 15](#_Toc60321976)

Supplementary Table S1. Overview of statistics for clade unity.

The abbreviations for the vertebrate groups are as follows: A, placental mammals; B, marsupials; C, monotremes; D, birds; E, crocodiles; F, turtles; G, lizards; H, amphibia; I, lobe-finned fish; J, Chondrostei - Polypteriformes (reedfish); K, Chondrostei - Acipenseriformes (sterlet sturgeon); L, Holostei (spotted gar); M, teleosts; N, cartilaginous fishes. To avoid long-branch attraction and sonsequently affected statistics, the *GJE1*/*gje1* orthogroup was omitted in these statistical runs, but in separate analyses, the *GJE1*/*gje1* orthogroup always cluster as a united group. Cyclostomes are not included in these analyses, as their gene family structure is too aberrant to easily present in the table.

|  |  |  | Amino acids | | | |  | | Nucleotides | | | | | | | |
| --- | --- | --- | --- | --- | --- | --- | --- | --- | --- | --- | --- | --- | --- | --- | --- | --- |
| Main model -> |  |  | Neighbor-Joining | | | | Maximum Likelihood | | Maximum Likelihood | | | | Neighbor-Joining | | | |
|  | Vertebrate groups |  | EIM^1^ | JTT08^2^ | JTT1^3^ | JTT1.1^4^ | JTT^5^ | Dayh^6^ | GTR^7^ | GTR^8^ | K2^9^ | T3^10^ | K2^11^ | MLC^12^ | T3^13^ | TN^14^ |
| GJA1 | ABCDEFGHIJKLMN | 14/14 | 100 | 97 | 99 | 99 | 100 | 100 | 100 | 99 | 100 | 100 | 100 | 100 | 100 | 100 |
| gja2 | D FGHIJKLMN | 14/14 | 98 | 93 | 99 | 99 | 99 | 99 | 99 | 99 | 99 | 99 | 98 | 98 | 98 | 97 |
| GJA3 | ABCDEFGHIJKLMN | 14/14 | 92 | 97 | 98 | 99 | 53 | 61 | 99 | 69 | 100 | 100 | 99 | 99 | 99 | 99 |
| (gja2-GJA3) |  | 14/14 | 96 | 87 | 98 | 99 | 77 | 72 | 98 | 66 | 97 | 98 | 96 | 96 | 96 | 98 |
| GJA4 | ABCDEFGHIJKLMN | 7/14 | 100 | 40 | 73 | 73 | 37 | 22 | - | 52 | - | - | - | - | - | - |
| GJA4 | ABCDEFGHI N | 7/14 | - | - | - | - | - | - | 87 | - | 86 | 86 | 82 | 81 | 78 | 81 |
| gja4 | JKLM | 7/14 | - | - | - | - | - | - | 99 | - | 99 | 99 | 99 | 99 | 99 | 99 |
| GJA5 | ABCDEFGHIJKLMN | 14/14 | 99 | 97 | 99 | 99 | 99 | 99 | 99 | 99 | 99 | 99 | 99 | 100 | 99 | 99 |
| GJA8 | ABCDEFGHIJKLMN | 14/14 | 99 | 98 | 100 | 100 | 98 | 98 | 100 | 99 | 100 | 100 | 100 | 100 | 100 | 100 |
| GJA9 | A CDEFGHIJKLMN | 14/14 | 57 | 47 | 57 | 57 | 58 | 56 | 39 | 63 | 36 | 36 | 50 | 50 | 49 | 48 |
| GJA10 | AB DEFGHIJKLMN | 2/14 | - | 53 | - | 39 | ^D^ | ^D^ | - | - | - | - | - | - | - | - |
| GJA10 | AB DEFGHIJK N | 9/14 | 18 | - | 14 | - | - | - | 36 | - | 37 | 51 | 38 | 40 | 39 | 43 |
| GJA10 | AB DEFGHI N | 1/14 | - | - | - | - | - | - | - | 17 | - | - | - | - | - | - |
| gja10 | LM | 9/14 | 17 | - | 16 | - | - | - | 51 | - | 51 | 49 | 49 | 51 | 52 | 53 |
| gja10 | JKLM | 1/14 | - | - | - | - | - | - | - | 17 | - | - | - | - | - | - |
| gja11 | I KLMN | 4/14 | 14^A^ | 27 | 30 | 29 | ^E^ | ^E^ | - | - | - | - |  | - |  | - |
| gja11 | I KL N | 7/14 | - | - | - | - | - | - | 29 | ^M^ | 31 | 30 | 34 | 39 | 38 | 34 |
| gja11 | M | 8/14 | - | - | - | - | - | - | 99 | 99 | 99 | 99 | 99 | 100 | 99 | 99 |
| gja12 | M | 6/14 | 12^B^ | 10^B^ | 48^B^ | 46^B^ | 39 | 40^B^ | ^H^ | ^N^ | ^H^ | ^H^ | ^R^ | ^R^ | ^R^ | ^R^ |
| gja13 | M | 14/14 | 32 | 26 | 55 | 54 | 68 | 62 | 54 | 61 | 53 | 53 | 57 | 57 | 60 | 54 |
| (gja12-gja13) |  | 14/14 | 77 | 48 | 88 | 88 | 66 | 86 | 93 | 95 | 94 | 93 | 86 | 86 | 88 | 89 |
| gja14 | I KL | 14/14 | 99 | 99 | 98 | 98 | 95 | 93 | 97 | 93 | 96 | 98 | 99 | 99 | 99 | 100 |
| (gja14- GJA5) |  | 12/14 | 69 | 67 | 81 | 82 | - | - | 84 | 90 | 82 | 84 | 86 | 88 | 87 | 99 |
| gja14-(GJA9-GJA10) |  | 2/14 | - | - | - | - | 16 | 21 | - | - | - | - | - | - | - | - |
| GJB1 | ABCDEFGHIJKLMN | 14/14 | 98 | 80 | 98 | 98 | 96 | 97 | 100 | 97 | 100 | 100 | 100 | 100 | 100 | 99 |
| GJB2 | ABC | 14/14 | 94 | 94 | 98 | 98 | 58 | 98 | 100 | 99 | 99 | 100 | 100 | 100 | 99 | 99 |
| gjb2 | DEFG | 1/14 | - | - | - | - | 28 | - | - | - | - | - | - | - | - | - |
| gjb2 | DEF | 11/14 | - | - | 25 | 27 | - | 47 | 24 | 48 | 26 | 27 | 28 | 29 | 25 | 28 |
| gjb2 | G | 13/14 | 33 | 67 | 41 | 43 | - | 65 | 69 | 66 | 68 | 69 | 71 | 69 | 69 | 75 |
| GJB3 | ABCDEFGHIJKLMN | 14/14 | 52 | 1 | 63 | 63 | 63 | 61 | 67 | 53 | 67 | 67 | 57 | 53 | 52 | 52 |
| GJB4 | ABC EF I | 2/14 | - | ^S^ | 5 | 5 | - | - | - | - | - | - | - | - | - | - |
| GJB4 | ABC EFG | 11/14 | 27 | ^S^ | - | - | 30 | 34 | 45 | 56 | 41 | 46 | 51 | 50 | 49 | 47 |
| gjb4 | H | 14/14 | 85 | 3 | 88 | 88 | 88 | 84 | 97 | 97 | 96 | 97 | 97 | 99 | 97 | 96 |
| GJB5 | ABCDEFGH | 7/14 | 20 | ^S^ | 29 | 29 | 54 | 46 | - | 66 | - | 53 | - | - | - | - |
| (gjb4 H – GJB5) |  | 13/14 | 26 | - | 45 | 45 | 62 | 59 | 16 | 44 | 17 | 19 | 20 | 19 | 24 | 19 |
| GJB5 | ABCDEFG | 7/14 | - | - | - | - | - | - | 48 | - | 55 | 53 | 44 | 35 | 45 | 43 |
| gjb5 | H | 8/14 | - | 92 | - | - | - | - | 98 | - | 99 | 98 | 99 | 99 | 100 | 98 |
|  |  |  |  |  |  |  |  |  |  |  |  |  |  |  |  |  |
| gjb5 H - (gjb4 H - GJB5) |  |  | - | - | - | - | - | - | 29 | - | 29 | 32 | 32 | 32 | 24 | 32 |
| GJB6 | ABCDEFG | 2/14 | - | - | 17 | 16 | - | - | - | - | - | - | - | - | - | - |
| GJB6 | ABC | 10/14 | - | - | - | - | 100 | 100 | 99 | 99 | 98 | 99 | 100 | 99 | 99 | 100 |
| GJB6 | ABC F | 2/14 | 21 | 13 | - | - | - | - | - | - | - | - | - | - | - | - |
| gjb6 | DEFG | 4/14 | - | - | - | - | - | - | - | - | - | - | 24 | 22 | 23 | 21 |
| gjb6 | DEF | 6/14 | - | - | - | - | 51 | 52 | 53 | 48 | 34 | 37 | - | - | - | - |
| gjb6 | G | 9/14 | 60 | 67 | - | - | 73 | 65 | 35 | 66 | 71 | 68 | - | - | - | - |
| GJB7 | ABCDEFGHIJKLMN | 13/14 | 79 | ^S^ | 83 | 87 | 95 | 96 | 98 | 98 | 98 | 98 | 98 | 98 | 96 | 98 |
| gjb8 | HIJKLMN | 8/14 | - | ^S^ | - | - | 26 | 24 | 31^I^ | - | 33^I^ | - | 38^I^ | 39^I^ | 38^I^ | 37^I^ |
| gjb8 | HIJKLM | 2/14 | - | - | 22 | 22 | - | - | - | - | - | - | - | - | - | - |
| gjb8 | H JKLM | 2/14 | 33 | - | - | - | - | - | - | 34^O^ | - | - | - | - | - | - |
| gjb9 | JKLM | 13/14 | 77 | ^S^ | 82 | 83 | 97 | 97 | 95 | 98 | 95 | 97 | 90 | 90 | 89 | 93 |
| gjb10 | JKLM | 13/14 | 55 | ^S^ | 77 | 77 | 79 | 82 | 61 | 86 | 62 | 61 | 55 | 56 | 55 | 56 |
| gjb10 | I N | 7/14 | ^C^ | - | - | - | - | - | 25^J^ | - | 27^J^ | 24 | 29 | 29 | 27 | 25 |
| gjb10 | N | 5/14 | - | - | 68 | 68 | 92 | 94 | - | 87 | - | - | - | - | - | - |
| gjb10-(GJB4-GJB5) |  | 10/14 | 6 | - | 20 | 20 | - | - | 8 | - | 9 | 9 | 6 | 7 | 9 | 7 |
| gjb10N-GJB3 |  | 2/14 | - | - | - | - | 29 | 27 | - | - |  |  |  |  |  |  |
| gjb11 | N | 14/14 | 99 | 95 | 100 | 100 | 99 | 99 | 100 | 99 | 100 | 100 | 100 | 100 | 100 | 100 |
| GJC1 | ABCDEFGHIJKLMN | 7/14 | 18 | - | 57 | 56 | 34 | 37 | - | - | 20 | 22 | - | - | - | - |
| GJC1 | ABCDEFG IJKLMN | 1/14 | - | - | - | - | - | - | 17^K^ | - | - | - | - | - | - | - |
| GJC1 | ABCDEFG IJKLM | 4/14 | - | - | - | - | - | - | - | - | - | - | 12 | 13 | 13 | 12 |
| GJC1 | ABCDEFGH N | 1/14 | - | - | - | - | - | - | - | 9^P^ | - | - | - | - | - | - |
| GJC2 | ABCDEFGHIJKLMN | 14/14 | 45 | 27 | 56 | 56 | 66 | 70 | 39 | 47 | 40 | 46 | 43 | 41 | 46 | 43 |
| GJC3 | AB | 6/14 | 66 | - | 88 | 88 | 55 | 52 | - | 64 | - | - | - | - | - | - |
| GJC3 | A | 8/14 | - | 100 | - | - | - | - | 100^L^ | - | 100^L^ | 100^L^ | 100^L^ | 100^L^ | 100^L^ | 100^L^ |
| GJC3 | B | 8/14 | - | 100 | - | - | - | - | 100^L^ | - | 100^L^ | 100^L^ | 100^L^ | 100^L^ | 100^L^ | 100^L^ |
| gjc4 | DE GHIJKLMN | 13/14 | 6 | 0 | 33 | 31 | - | 20 | 23 | 28 | 28 | 31 | 28 | 33 | 31 | 32 |
| gjc4 | DE GHIJKLM | 1/14 | - | - | - | - | 53^F^ | - | - | - | - | - | - | - | - | - |
| GJD1 | CD FGHIJKLMN | 4/14 | 26 | 15 | 25 | 24 | ^G^ | ^G^ | ^G^ | ^Q^ | ^G^ | ^G^ | 1 | 2 | ^S^ | ^S^ |
| GJD2 | AB DEFGHIJKLMN | 6/14 | 30 | 11^T^ | 41 | 44 | 68 | 63 | - | - | - | - | - | - | - | - |
| GJD2 | AB DEFGHIJKLM | 8/14 | - | - | - | - | - | - | 14 | 8 | 14 | 11 | 10 | 11 | 13 | 11 |
| gjd2 | N | 8/14 | - | - | - | - | - | - | 11 | 37 | 54 | 45 | 13 | 56 | 58 | 59 |
| GJD3 | ABCDEFGHIJKLMN | 14/14 | 99 | 86 | 100 | 100 | 99 | 98 | 99 | 100 | 100 | 99 | 99 | 98 | 99 | 98 |
| GJD4 | ABCDEFGHIJKLMN | 14/14 | 99 | 100 | 100 | 100 | 100 | 99 | 100 | 100 | 100 | 100 | 100 | 100 | 100 | 100 |
| GJD5 | AB HIJKLMN | 14/14 | 96 | 85 | 99 | 100 | 96 | 98 | 100 | 98 | 100 | 100 | 100 | 100 | 100 | 100 |
| gjd6 | JKLM | 14/14 | 100 | 86 | 100 | 100 | 99 | 99 | 100 | 99 | 100 | 100 | 100 | 100 | 100 | 100 |

1. EIM: Equal Input Model. Gamma parameter was set to 0.8. There were a total of 205 positions in the final dataset, with 559 unique sequences.

2. JTT08: Jones-Taylor-Thornton model. Gamma parameter was set to 0.8. There were a total of 205 positions in the final dataset, with 559 unique sequences. This model is the most deviating of all run models, due tue a considerable degree of splitting up orthogroups.

3. JTT1: Jones-Taylor-Thornton model. Gamma parameter was set to 1. There were a total of 205 positions in the final dataset, with 559 unique sequences.

4. JTT11: Jones-Taylor-Thornton model. Gamma parameter was set to 1.1. There were a total of 205 positions in the final dataset, with 559 unique sequences. This corresponds to Figure 2 in the main text.

5. JTT: Jones-Taylor-Thornton model. Gamma: (5 categories =0.9438). Positions with less than 95% site coverage were eliminated. There were a total of 191 postions in the final dataset, consisting of 559 unique sequences.

6. Dayh: Dayhoff model. Gamma: (5 categories =0.9119). Positions with less than 95% site coverage were eliminated. There were a total of 191 postions in the final dataset, consisting of 559 unique sequences.

7. GTR: General Time Reversible model. Gamma: 5 categories (=0.8459). Rate variation allowed some sites to be invariable (5.5% of sites). Positions with less than 95% site coverage were eliminiated. Only positions 1 and 2 in the codons were included. A total of 382 positions were in the final dataset, with 559 unique sequences.

8. GTR. As 5, but without invariant sites. Only positions 1 and 2 in the codons were included. A total of 382 positions were in the final dataset, with 559 unique sequences.

9. K2: Kimura 2-parameter model. Gamma: 5 categories (=0.8459). Rate variation allowed some sites to be invariable (5.5% of sites). Positions with less than 95% site coverage were eliminiated.. Only positions 1 and 2 in the codons were included. A total of 382 positions were in the final dataset, with 559 unique sequences.

10. T3: Tamura 3-parameter model. Gamma: 5 categories (=0.8459). Rate variation allowed some sites to be invariable (5.5% of sites). Positions with less than 95% site coverage were eliminated. Only positions 1 and 2 in the codons were included. A total of 382 positions were in the final dataset, with 559 unique sequences.

11. K2: Kimura 2-parameter model. Only positions 1 and 2 in the codons were included. A total of 410 positions were in the final dataset, with 559 unique sequences.

12. MLC: Maximum Composite Likelihood. Only positions 1 and 2 in the codons were included. A total of 410 positions were in the final dataset, with 559 unique sequences.

13. T3: Tamura 3-parameter method. Gamma: 5 categories (=0.8459). Positions with less than 95% site coverage were eliminated. Only positions 1 and 2 in the codons were included. A total of 410 positions were in the final dataset, with 559 unique sequences.

14. TN: Tamura-Nei method. Gamma: 5 categories (=0.8459). Positions with less than 95% site coverage were eliminated. Only positions 1 and 2 in the codons were included. A total of 410 positions were in the final dataset, with 559 unique sequences.

A. The sequence from little skate (Le-gja11-AESE0100411979) locates separately at the stem of the (*gja11*-*gja12*-*gja13*) branch.

B. A sequence from eel (Aj-gja12-BEWY01000019) locates outside (*gja12*-*gja13*).

C. The sequences from cartilaginous fishes have split, and the sequence from elephant shark locates together with the *Latimeria* *gjb10 (=gjb4)* sequence.

D. GJA10 splits up in several “paraphyletic” groups.

E. Gja 11 splits up into several “paraphyletic” groups.

F. Gjc4 splits up and cartilaginous fishes locates below (other gjc4 – GJC2 – GJC1).

G. Splits up in many smaller groups, that mostly “multitomize” with GJD2.

H. Splits up, having one zebrafish and one eel sequence outside the other (gja12-gja13) sequences.

I. Two herring sequences locate outside all other (GJB1-gjb8-GJB2-GJB6) sequences

J. Locates dichotomizing with GJB3 with low bootstrap value (<20).

K. The amphibian sequences locate as trichotomizing with the (other GJC1-(GJC2-gjc4)).

L. Both groups are individually branching off from the stem leading to (GJC1-(GJC2-gjc4)).

M. Splits up in subgroups that gradually branch off from the stem leading to *gja12* and *gja13*.

N. One eel sequence locates outside all other (gja12-gja13) sequences.

O. Gjb8 splits up, having Latimeria and cartilaginous fishes located as separate clades the collected (amphibia-ray-finned fish) clade.

P. The ray-finned fish sequences split out, locating below the other (*GJC1*-*GJC2*-*gjc4*) sequences.

Q. *Gjd1* splits up into many closely located subgroups.

R. One eel and one zebrafish sequence locate outside the other (*gja12*-*gja13*) sequences.

S. The orthogroups are highly split up.

T. Two herring sequences locate outside GJD2 and GJD1.

Supplementary Table S2. Eel connexin ohnologs. The start position in the chromosomal (linkage group) location is the A of the ATG codon (regardless whether the gene is on the plus (F) or minus (R) strand). In some cases, we have not been able to recover the full coding sequence. Start is then the first nucleotide for the recovered sequence. The linkage groups are from an *Anguilla japonica* assembly, while the TSAs are from either *Anguilla japonica* (Aj) or *Anguilla anguilla* (Aa). The percentage of identity is for the full-length nucleotide genomic sequences for the two ohnologs in the columns for the linkage groups.

| **Ohnolog a** | |  | **Ohnolog b** | |  |
| --- | --- | --- | --- | --- | --- |
| GenBank TSA (acc.no-name[if any]-Species) | Linkage group: start pos-direction  (GenBank assembly Ajp_01 GCA_003597225.1) |  | GenBank TSA (acc.no-name[if any]-Species) | Linkage group: start pos-direction  (GenBank assembly Ajp_01 GCA_003597225.1) | % id  (DNA) |
| GGGR01006377.1-Aa  GFIC01021932.1-CX43-Aa | 19:8845796-F | ***gja1*** | GFIC01021951.1-CXA1-Aa | 7:31792677-R | 80.2 |
| GFIC01038195.1-Aa | 8:12085749-R | ***gja2*** | GFIC01021931.1-CX39.9-Aa | 15:29899912-F | 78.8 |
| GHAH0129126-Aa | 8:38903519-F | ***gja3*** | GFIC01025831.1-GJA3-Aa | 14:20990510-R | 88.3 |
| GHAH01183299.1-Aa | 7:63404758-F | ***gja4*** | GGGR01021278.1-cx39.4-Aa | 4:25091439-F | 71.4 |
|  |  |  |  |  |  |
| GGGR01003355.1-Aa  GFIC01021933.1-CX45.6-Aa | 8:36502612-F | ***gja5*** | GFIC01021952-CXA5-Aa | 14:14070082-R | 81.0 |
| - | 8:36475103-R | ***gja8*** | - | 14:14093752-F | 90.7 |
| - | 68unplaced:255669-R | ***gja9*** | GFCL01016039.1-Aj | 7:3645320-F | 87.4 |
| GGGR01023409.1-Aa | 8:12093225-R | ***gjb1*** | GGGR01028360.1-cxb1-Aa | 15:29889281-F | 85.0 |
| GFCM01012153.1-Aj  GGCL01059627.1-Aa | 7:63231835-R | ***gjb3*** | GHAH01707820.1-Aa | 4:24893138-R | 77.5 |
| GAGT01007194.1-Aj | 8:38895820-F | ***gjb8*** | GHAH01171652.1-Aa | 14:20998664-R | 86.8 |
| GFIC01035793.1-Aa | 7:63419570-R | ***gjb9*** | GAGT01014339.1-Aj  GHAH01060111.1-Aa | 4:25113309-R | 78.5 |
| GAGT01000730.1-Aj  GFIC01036470.1-Aa | 7:63245117-R | ***gjb10*** | GHAH01134985.1-Aa  GHAH01796839.1-Aa | 4:24900540-R | 76.0 |
| GHAH01012974.1-Aa | 18:912972-F | ***gjc1*** | GFIC01021967.1-CXG1-Aa | 1:29923736-R | 78.8 |
| GGGR01003279.1-Aa | 156unplaced:94450-F(ex2) | ***gjd1*** | GGGR01015808.1-Aa | 15:10141380-F | 85.1 |
| GGCL01029232.1-Aa | 7:8886309-R(ex2) | ***gjd2*** | GGGR01046632.1-gjd2-Aa | 19:19772161-F | 84.5 |

Supplementary Table S3. Atlantic herring connexin ohnologs. The Ensembl gene numbers are abbreviated to the 5 or 6 last digits. Thus G71192.5 should be read as ENSDARG00000071192.5. The names/description of the sequences in GenBank or Ensembl are abbreviations of the spelled-out names. The start position in the chromosomal location is the A of the ATG codon (regardless whether the gene is on the plus (F) or minus (R) strand). In a couple of cases, we do not trust the suggested first exon in the predicted sequences, and the start position is then at the start of exon 2.

| **Ohnolog a** | |  | **Ohnolog b** | |  |
| --- | --- | --- | --- | --- | --- |
| GenBank  (acc.no-name) | Chr: start pos-direction  (GenBank assembly Ch_v2.0.2, GCF_900700415.1 ) |  | GenBank  (acc.no-name) | Chr: start pos-direction  (GenBank assembly Ch_v2.0.2, GCF_900700415.1 ) | % id  (DNA) |
| XM_012829211- gja1 | 15:22839795-F | ***gja1*** | XM_012836783-gja1like | 14:19872005-R | 72.4 |
| XM_012819598-gja3like | 8:955029-R | ***gja2*** | XM_012834366-gja3like | 20:17668503-R | 78.5 |
| XM_012842347-gja3like | 2:13378217-F | ***gja3*** | XM_012840585-gja3 | 21:10442713-R | 76.5 |
| XM_012816449-gja5like | 2:30832286-F^A^ | ***gja5*** | XM_012840593-gja5like | 21:13506697-R | 75.1 |
| XM_031580546-gja8like | 2:3036755 | ***gja8*** | XM_012840595-gja8 | 21:13525875-F | 82.8 |
| XM_012816385-gja9like | 19:2788247-F | ***gja9*** | XM_012824682-gja9like | 11 or 14^B^ | 81.2 |
| XM_012836705-gja10like | 14:19727965:F | ***gja10*** | XM_012821374-gja10 | 15:3936155-R | 70.0 |
| XM_012819602-gjb1like | 8^B^ | ***gjb1*** | XM_012834339-gjb2like | 20:17674129-R | 77.3 |
| XM_012822385-gjb3like | 14:2252431-R | ***gjb3*** | XM_012818491-gjb3like | 19:26458562-F | 71.0 |
| XM_012842299-gjb2like | 2:13372580-F | ***gjb8*** | XM_012820173-gjb2like  XM_012840586-gjb2like | 8:906158-R  21:10448147-R | 73.4  74.0 |
| XM_012822073-gjb4like | 19:3571238-R | ***gjb9*** | XM_012826764-gjb4like | 14:12568042-F | 76.9 |
| XM_012818492-gjb4like | 19:26455502 | ***gjb10*** | XM_012822396-gjb4like | 14:22526131-R | 69.1 |
| XM_012816830-gjc1 | 1:10122555-R | ***gjc1*** | XM_012817598-gjc1like | 1:17348190-F | 74.7 |
| XM_012836489-gjc1like | 2:28922971-F | ***gjc4*** | XM_012821065-gjc1like | 21:4733911-R | 76.9 |
| XM_012819299-gjd2 | 15:9736801-F | ***gjd2*** | XM_012823340-gjd2-cx36 | 14:20656673-R | 81.5 |

A. The genomic assembly is a bit different from the predicted sequence, and under strict Blasting conditions, there is alignment from pos. 43 in the predicted sequence and with some misalignments in the intracellular loop region.

B. See ref. Mikalsen, Tausen, í Kongsstovu (2020) BMC Genomics 2020, 21, 223.

Supplementary Table S4. Zebrafish connexin ohnologs. The Ensembl gene numbers are abbreviated to the 5 or 6 last digits. Thus G71192.5 should be read as ENSDARG00000071192.5. The names/description of the sequences in GenBank or Ensembl are abbreviations of the spelled-out names. The start position in the chromosomal location is the A of the ATG codon (regardless whether the gene is on the plus (F) or minus (R) strand). In a couple of cases, we do not trust the suggested first exon in the predicted sequences, and the start position is then at the start of exon 2.

| **Ohnolog a** | | |  | **Ohnolog b** | | |  |
| --- | --- | --- | --- | --- | --- | --- | --- |
| GenBank  (acc.no-name) | Ensembl  (gene no.-name) | Chr: start pos-direction  (Ensembl and GenBank assembly GRCz11) |  | GenBank  (acc.no-name) | Ensembl  (gene no.-name) | Chr: start pos-direction  (Ensembl and GenBank assembly GRCz11) | % id  (DNA) |
| XM_688906-gja1like | G71192.5-cx40.8 | 17:15415878-F | ***gja1*** | NM_131038-cx43 | G41799.7-cx43 | 20:40717900-R | 71.9 |
| NM_001007213-gja5a  AF531762-cx45.6 | G40065.7-gja5a | 9:30475594-F | ***gja5*** | NM_001034988-gja5b | G69450.4-gja5b  (cx41.8) | 1:47416840-R | 69.7 |
| NM_001128350-gja8a  GQ253466-cx44.1like (cx50.5) | G69451.7-gja8a | 9:30432317-R | ***gja8*** | NM_131809-gja8b  AF304050-cx44.2  AF288817-cx44.1 | G15076.10-gja8b | 1:47446550-F | 74.6 |
| NM_207093-cx52.9 | G86453.3-cx52.9 | 17:53435461-F | ***gja9*** | XM_021466745-cx55.5 | not predicted | 16:33158053-F | 67.9 |
| XM_021467222-cx52.7 | G57792.8-cx52.7 | 17:15746877-R | ***gja10*** | NM_212819-cx52.6 | G34930.8-cx52.6 | 20:23960766-F | 70.1 |
| NM_131811-cx27.5 | G35553.7-cx27.5 | 5:23626921-F | ***gjb1*** | NM_001386510-cx31.7 | G58064.6-cx31.7 | 14:12332792-F | 69.0 |
| NM_001007212-cx28.6 | G03925.6-cx28.6 | 17:14711793-F | ***gjb9*** | NM_001007288-Cx30.9 | G70362.4-cx30.9 | 19:37118595-F | 67.3 |
| NM_131069-cx43.4 | G07099.9-cx43.4  (gja7) | 9:46410326-R | ***gjc4*** | NM_131810-cx44.2 | G-34207.7-cx44.2 | 6:21005731-F (6:21022101-F, 95.6% id)  (6:21062446-F, 94.7% id) | 69.2 |
| NM_001128766-gjd1a | G111977.1-gjd1a  (cx34.1) | 5:36974969-F (ex1) | ***gjd1*** | XM_009291479-gjd2like | G35765.6-no name | 15:6678962-F (ex1) | 74.9 |
| not predicted | G67999.3-gjd2 | 17:50976037-F (ex2) | ***gjd2*** | NM_194420-gjd2b  BC162926-cx35  AF512548-cx35.1 | G70781.6-gjb2b | 20: 9963666-R (ex1) | 82.0 |
| XM_021473060-gje1like | G53062.6-gje1like | 17:6382492-F (ex2) | ***gje1*** | NM_001013546-cx23 | G54150.7-cx23 | 20:37295138-F (ex1) | 75.9 |

Supplementary Table S5. Sterlet sturgeon connexins and their ohnologs. Percentages of identity for ohnologous coding sequences in *Acipenser ruthenus*. Note that for some connexins, there are more than two sequences that are likely associated as ohnologs (*gja3*, *gjb8*, *gjc1*). It appears like some of the ohnologs have evolved into pseudogenes, although sequencing/assembly errors cannot be excluded.

| A ohnolog | | | | | |  | B ohnolog | | | | | |  |
| --- | --- | --- | --- | --- | --- | --- | --- | --- | --- | --- | --- | --- | --- |
| GenBank accession number | Scaffold VTUV01000xxx | Chr | Pos from | Pos to | Dir |  | GenBank accession number | Scaffold VTUV01000xxx | Chr | Pos from | Pos to | Dir | Identity between A and B (%) |
| XM_034004250 | 005 | 5 | 14839659 | 14840804 | F | ***gja1*** | XM_034017373 | 006 | 6 | 56635793 | 56636938 | F | 96.6 |
| XM_034020768 | 021 | 21 | 27925765 | 27926844 | F | ***gja2*** | XM_034051396 | 092 | unpl^B^ | 939874 | 940953 | F | 98.8 |
| XM_034012191 | 008 | 8 | 27352178 | 27353425 | F | ***gja3*** | XM_034923915 | 009 | 9 | 6914431 | 6915645 | F | 94.39+2% gaps |
|  |  |  |  |  |  |  | Not predicted | 235 | unpl | 2086 | 867 | R | 94.08 +2% gaps |
| XM_034023067 | 025 | 25 | 13689697 | 13688789 | R | ***gja4*** | XM_034022433 | 029 | 29 | 12632139 | 12633047 | F | 98.79 |
| XM_034011119 | 008 | 8 | 47939751 | 47940830 | F | ***gja5*** | XM_034011119 | 009 | 9 | 21073312 | 21074391 | F | 97.5 |
| XM_034010978 | 008 | 8 | 47901502 | 47900381 | R | ***gja8*** | XM_034009780 | 009 | 9 | 21029934 | 21028810 | R | 98.22 |
| XM_034023019 | 025 | 25 | 9294626 | 9296167 | F | ***gja9*** | XM_034022526 | 029 | 29 | 16835595 | 16834054 | R | 98.7 |
| XM_034004988 | 005 | 5^A^ | 84191567 | 84190047 | R | ***gja10*** | - | - | - | - | - | - | - |
| XM_034004251 | 005 | 5 | 14830191 | 14831216 | F | ***gja11*** | - | - | - | - | - | - | - |
| XM_034910414 | 034 | 34 | 4758661 | 4757606 | R | ***gja14*** | XM_034913590 | 046 | 46 | 4477260 | 4478315 | F | 98.86 |
| XM_034903629 | 021 | 21 | 27913204 | 27914022 | F | ***gjb1*** | XM_034019835 | 092 | unpl | 927404 | 928222 | F | 99.15 |
| XM_034906208 | 025 | 25 | 12532537 | 12531731 | R | ***gjb3*** | XM_034908573 | 029 | 29 | 13924794 | 13925600 | F | 96.78 |
| XM_034005445 | 005 | 5 | 85224251 | 85225015 | F | ***gjb7*** | XM_034017448 | 006 | 6 | 81326300 | 81327064 | F | 96.71 |
|  |  |  |  |  |  |  | XM_034017449 |  | 6 | 81319391 | 81320131 | F | 97 |
| XM_034011049 | 008 | 8 | 27343950 | 27344729 | F | ***gjb8*** | XM_034919050 | 410 | unpl | 6155 | 5375 | R | 96.04+0.4% gaps |
|  |  |  |  |  |  |  | XM_034009766 | 235 | unpl | 8274 | 7494 | R | 96.04+0.4% gaps |
|  |  |  |  |  |  |  | XM_034008147 | 009 | 9 | 6908186 | 6908967 | F | 95.79+0.5% gaps |
| XM_034024040 | 025 | 25 | 13672250 | 13673131 | F | ***gjb9*** | XM034022435 | 029 | 29 | 12649594 | 12648794 | R | 98.41 |
| XM_034906258 | 025 | 25 | 12542820 | 12542038 | R | ***gjb10*** | XM_034021714 | 029 | 29 | 13914433 | 13915215 | F | 98.08 |
| XM_034057391 | 026 | 26 | 16981823 | 16980495 | R | ***gjc1*** | Not predicted | 2086 | unpl | 23060 | 24056 | F | 91.01 + 4% gaps |
|  |  |  |  |  |  |  | Not predicted | 2085 | unpl | 17978 | 19119 | F | 90.82 + 4% gaps |
| XM_034000763 | 003 | 3 | 23176065 | 23174824 | R | ***gjc2*** | XM_033993461 | 004 | 4 | 23488776 | 23490017 |  | 97.02 |
| XM_034043916 | 012 | 12 | 22218656 | 22219834 | F | ***gjc4*** | XM_034004211 | 010 | 10 | 31227246 | 31228409 | F | 93.72 |
| Not predicted | 007 | 7 ex1  ex2 | 43199321  43194085 | 43199251  43193201 | R | ***gjd1*** | - | - | - | - | - | - | - |
| XM_034037009 | 016 | 16ex1 ex2 | 24577254  24577839 | 24577324  24578676 | F | ***gjd2*** | XM_034906013 | 024 | 24 | 9708586  9708012 | 9708516  9707175 | R | 99.05 |
| XM_034049542 | 027 | 27 | 17869217 | 17868171 | R | ***gjd6*** | XM_034047219 | 2123 | unpl | 28225 | 29271 | F | 98.85 |
| XM_034906705 | 026 | 26 | 25708856 | 25707951 | R | ***gjd3*** | XM_034909316 | 031 | 31 | 52175 | 53110 | F | 88.35 + 3% gaps |
| XM_033999548 | 003 | 3 ex1  ex2 | 5988003  5984894 | 5987940  5983714 | R | ***gjd4*** | XM_033992900 | 004 | 4 | 9349246  9351307 | 9349309  9352487 | F | 96.37 |
| XM_034050044 | 027 | 27 | 6544191 | 6545264 | F | ***gjd5*** | XM_034902567 | 020 | 20 | 22022059 | 22020986 | R | 98.88 |
| XM_034049542 | 027 | 27 | 17869217 | 17868171 | R | ***gjd6*** | XM_034047219 | 2123 | unpl | 28225 | 29271 | F | 98.85 |
| XM_034003916 | 005 | 5 ex1  ex2  ex3 | 49198091  49199541  49200145 | 49198128  49199723  49200540 | F | ***gje1*** | XM_034017350 | 006 | 6 | 22448676  22447911  22447153 | 22448638  22447729  22446758 | R | 96.25 |

A. Not full length. Lacking the 3’-exon.

B. Unpl = unplaced.

Supplementary Table S6. Chromosomal locations of connexin genes in cartilaginous fishes.

The phylogenetic analyses use sequences from other cartilaginous fishes (Callorhinchus milii; Rhincodon typus; Leucoraja erinacea), but their genome assemblies are not chromosome level. We therefore used the sequences from these three species and blasted into the chromosome level assemblies indicated in the Table (the phylogenetic sequences were collected before these chromosomal assemblies became available). Note that the indicated ranges do not necessarily contain the complete reading frame. The assemblies are found in GenBank. The colored cells indicate remarks found below the Table.

|  | *Amblyraja radiata* (sAmbRad1.pri; GCA_010909765) | | | | | *Pristis pectinata* (sPriPec2.pri; GCA_009764475.1) | | | | | *Chiloscyllium plagiosum* (ASM401019v1; GCA_004010195.1) | | | | |  |
| --- | --- | --- | --- | --- | --- | --- | --- | --- | --- | --- | --- | --- | --- | --- | --- | --- |
| Gene | Scaffold | Chr | From | To | Dir | Scaffold | Chr | From | To | Dir | Scaffold | Chr | From | To | Dir |  |
| ***gjc4.1*** | NW_022630392 | un | 1131 | 2125 | F | CM019855 | 1^C^ | 3991124 | 3990011 | R | CM012962 | 7^C^ | 105177438 | 105178563 | F |  |
| ***gjc4.2*^A^** | - | - | - | - | - | - | - | - | - | - | CM012962 | 7^C^ | 105097695 | 105098822 | F |  |
| ***gjd2*** | NC_045964 | 9 | 29347680  29348631 | 29347750  29349468 | F | CM019855 | 1^C^ | 102072417  102072995 | 102072487  102073832 | F | CM012965 | 10^C^ | 69158120  69158644 | 69158190  69159478 | F |  |
| ***gje1*** | NC_045963 | 8 | ?  58395817  58399261 | ?  58395999  58399649 | F | CM019857 | 3 | ?  137748327  137750830 | ?  137748509  137751218 | F | CM012964 | 9 | ?  8424759  8420712 | ?  8424583  8420553 | R |  |
| ***gjc2*** | NC_045957 | 2 | 24803411 | 24804649 | F | CM019859 | 5 | 9220599 | 9221837 | F | CM012960 | 5 | 112052571 | 112051345 | R |  |
| ***gjd4*** | NC_045957 | 2 | 12005452 | 12006169 | F | CM019859 | 5 | 23804463 | 238003474 | R | CM012960 | 5 | 91747387 | 91748634 | F |  |
| ***gjb1*** | NC_045967 | 12 | 52606630 | 52607475 | F | CM019862 | 8 | 83899353^D^ | 83900198 | F | CM012970 | 15 | 55017001 | 55017849 | F |  |
| ***gja2*** | NC_045967 | 12 | 52686460 | 52687476 | F | CM019862 | 8 | 83954486^D^ | 83955534 | F | CM012970 | 15 | 55073646 | 55074719 | F |  |
| ***gjb7*** | NC_045960 | 5 | 27808156 | 27807398 | R | CM019864 | 10 | 28087679 | 28086921 | R | CM012958 | 3 | 98288558 | 98289316 | F |  |
| ***gja10*** | - | - | - | - | - | CM019864 | 10 | 40441089 | 40440192 | R | CM012958 | 3 | 80692018  80696230 | 80692939  80696911 | F | 1 |
| ***gja11*** | NC_045960 | 5 | 50427253 | 50428134 | F | CM019864 | 10 | 46778571^E^ | 46779461 | F | CM012958 | 3 | 71475687 | 71474785 | R | 2 |
| ***gja1*** | NC_045960 | 5 | 50448801 | 50449949 | F | CM019864 | 10 | 46798762^E^ | 46799910 | F | CM012958 | 3 | 71431907 | 71430759 | R | 2 |
| ***gjb8*** | NC_045961 | 6 | 48648518 | 48647698 | R | CM019865 | 11 | 31153046^D^ | 31153830 | F | CM012961 | 6 | 76914210 | 76913424 | R |  |
| ***gja3*** | NC_045961 | 6 | 48637961 | 48636844 | R | CM019865 | 11 | 31168970^D^ | 31169946 | F | CM012961 | 6 | 76888512 | 76887328 | R |  |
| ***gja8*** | NC_045961 | 6 | 15366348 | 15367529 | F | CM019865 | 11 | 61867775 | 61866585 | R | CM012961 | 6 | 47817396 | 47818574 | F |  |
| ***gja5*^B^** | - | - | - | - | - | - | - | - | - | - | - | - | - | - | - |  |
| ***gjd5*** | NC_045983 | 28 | 34028078 | 34026999 | R | CM019875 | 21 | 15194427 | 15195506 | F | CM012983 | 28 | 18695046 | 18695870 | F |  |
| ***gjb11.1*** | NC_045982 | 27 | 928526 | 929162 | F | CM019876 | 22 | 2120602 | 2121236 | F | CM012982 | 27 | 1636775 | 1637443 | F |  |
| ***gjb11.2*** | NC_045982 | 27 | 923307 | 923998 | F | - | - | - | - | - | CM012982 | 27 | 1631217 | 1631990 | F |  |
| ***gja9*** | NC_045982 | 27 | 17280277 | 17281827 | F | CM019876 | 22 | 22301984 | 22303531 | F | CM012982 | 27 | 28716570 | 28718072 | F | 1 |
| ***gja4*** | NC_045982 | 27 | 20295393 | 20294338 | R | CM019876 | 22 | 24578333^D^ | 24577278 | R | CM012982 | 27 | 31867145 | 31866072 | R | 2 |
| ***gjb3.2*** | NC_045982 | 27 | 30344519 | 20343767 | R | CM019876 | 22 | 24621295^D^ | 24620544 | R | CM012982 | 27 | 31983978 | 31983214 | R |  |
| ***gjb3.1*** | NC_045982 | 27 | 20387981 | 20387184 | R | CM019876 | 22 | 24647511^E^ | 24646714 | R | CM012982 | 27 | 32060905 | 32060108 | R |  |
| ***gjb10*** | NC_045982 | 27 | 20491827 | 20491060 | R | CM019876 | 22 | 24725608^E^ | 24724841 | R | CM012982 | 27 | 32252617 | 32251816 | R |  |
| ***gjd3*** | NC_045971 | 16 | 8922168 | 8923028 | F | CM019879 | 25 | 9348092 | 9348943 | F | CM012988 | 33 | 10497586 | 10498437 | F |  |
| ***gjc1*** | NC_045971 | 16 | 29460976 | 29462148 | F | CM019879 | 25 | 22943175 | 22944347 | F | CM012988 | 33 | 28587942 | 28589114 | F |  |
| ***gjd1*** | NC_045996 | 41 | 14742622  14745659 | 14742692  14746511 | F | CM019889 | 35 | 4753174  4760136 | 4753244  4760987 | F | CM012996 | 41 | 5474896  5474898 | 5470507  5475750 | F |  |

A. Duplicated *gjc4* is found in some cartilaginous fishes, like elephant shark (*Callorhincus milii*) and whale shark (*Rhincodon typus*), but not in others, like little skate (*Leucoraja* *erinacea*).

B. *Gja5* is found in elephant shark (*Callorhincus milli*) and small-eyed rabbitfish (*Hydrolagus affinis*), and is on the same scaffold as *gja8*.

C. Among all the connexin genes in these three species, only a single gene, *gjd2*, breaks the chromosomal location pattern. To follow the pattern one of two possibilities should have occurred: (i) In *Chyloscillium plagiosum* *gjd2* should have been on chromosome 7, or (ii) in *Pristis pectinata* *gjd2* should have been on a chromosome different from 1, 3, 5, 8, 10, 11, 21, 22, 25 or 35.

D. Examples of distantly related but closely linked connexin genes.

E. Examples of closely related and closely linked connexin genes.

Supplementary Table S7. Chromosomal locations of connexin genes in cyclostomes.

The genomic positions in sea lamprey (*Petromyzon marinus*) were decided by blasting the predicted sequences in GenBank genome assembly kPetMAr1.pri GCA_010993605.1. The Pacific lamprey (*Entosphenus tridentatus*) genome assembly (GenBank GCA_014621495.2), Arctic lamprey (*Lethentheron camtschaticum*) genome assembly (GCA_000466285.1), and inshore hagfish (*Eptatretus burgeri*) genome assembly were similarly blasted. The yellow indicates genes that with confidence can be syntenically followed across the species.

| *Petromyzon marinus* | | | | | | | | *Entosphenus tridentatus* | | | *Lethentheron camtschaticum* | | *Eptatretus burgeri* | |
| --- | --- | --- | --- | --- | --- | --- | --- | --- | --- | --- | --- | --- | --- | --- |
| Accession no | GenBank  name | Our suggested name | Scaffold | Chr | Position From | Position To | Dir | Scaffold  JAAXL0200 | Chr | Position  From (dir) | Scaffold | Position  From (dir) | Scaffold  FYBX02xxxxx |  |
| XM_032947589 | gjd2like | gjd6 | NC_046069 | 1 | 24962246 | 24963367 | F | 01137 | 2 | 14425250F | KE993697 | 1877082R | 09427 | 3720780 R |
| XM_032959840 | gjd3like | gjd3.2 | NC_046070 | 2 | 6920977  6922148 | 6921165  6922921 | F | 01138 | 4 | 17751319R | KE993731 | 77031F | 05648 | 336761 R |
| XM_032959861 | gjd3like | gjd3.1 | NC_046070 | 2 | 6942165  6943121 | 6942353  6943798 | F | 01138 | 4 | 17731493R | KE993731 | 17731493R | 09698 | 6658961 R |
| XM_032967997 | gjc1like | gjc-gen3.1 | NC_046070 | 2 | 25443716  25444142 | 25444022  25444656 | R | 01138 | 4 | 3365574R | KE993850 | 56743R | 09842 | 3713372 F |
|  |  | gjc-gen3.2 |  |  |  |  |  |  |  |  |  |  | 09576 | 853291F |
| XM_032945854 | gjc1like | gjc-gen2 | NC_046072 | 4 | 9122864 | 9121438 | R | 01139 | 6 | 8085456R | KE995893 | 17729F | 10285 | 115272 F |
| XM_032947994 | gjd2like | gjd1/2a | NC_046074 | 6 | 8203019  8202717 | 8202949  8201877 | R | 01141 | 10 | 9844663F | KE993687 | 248014F | 10628 | 2200396 F |
| XM_032951755 | gjc1like | gjc-gen4 | NC_046078 | 10 | 15405677  15407295  15408931  15409545 | 15405978  15407999  15409077  15409797 | F | 01143 | 44 | 973044F | KE993784 | 162887R |  |  |
| XM_032964245 | gjd2like | gjd1/2b | NC_046099 | 31 | 12766646  12766373 | 12766574  12765521 | R | 01163 | 12 | 281372F | KE993878 | 708535R |  |  |
| XM_032965245 | gjd2like | gjd-gen1 | NC_046101 | 33 | 11932747  11933832 | 11932817  11934852 | F | 01156 | 23 | 10570306F | KE993853 | 10570306F | 10009 | 361025 R |
|  |  | gjd-gen1.2 |  |  |  |  |  |  |  |  |  |  | 10628 |  |
| XM_032972841 | gjb2like | gjb1/8.2 | NC_046117 | 49 | 10444054 | 10444860 | F | 01182 | 39 | 7753125F | KE993718 | 268065F | 10228 | 7053135 F |
| XM_032972781 | gjb2like | gjb1/8.1 | NC_046117 | 49 | 10525498 | 10526517 | F | 01182 | 39 | 7809092F | KE993718 | 2744557F | 10228 | 7093278 F |
| XM_032972719 | gja3 | gja3 | NC_046117 | 49 | 10551503  10553565 | 10551573  10554863 | F | 01559 | - | 218 | KE993718 | 2774835F | 10228 | 7450099 R |
|  |  |  |  |  |  |  |  |  |  |  |  |  | 10228 | 7519880 R |
|  |  |  |  |  |  |  |  |  |  |  |  |  | 10228 | 7550784 R |
|  |  |  |  |  |  |  |  |  |  |  |  |  | 10228 | 7561853 R |
| XM_032975402 | gjc1like | gjc-gen1 | NC_327030 | 56 | 327030 | 325606 | R | 14224 | - | 2509R | KE995893 | 17729F |  |  |
| XM_032977200 | gja8 | gja8 | NC_046130 | 62 | 203739  203258  198538 | 203423  203213  197677 | R | 01809 | - | 5122R | KE994890 | 19855F |  |  |
| XM_032977315 | gja5like | gja-gen1.1 | NC_046130 | 62 | 259689  263357  266524  270875 | 260144  263617  266826  271149 | F |  |  |  | KE995112 | 20887F |  |  |
| XM_032978223 | gjb3like | gjb-gen1.2 | NC_046134 | 66 | 805007  896498  808205  809322  813886  814365 | 805072  806613  808337  809565  814319  814624 | F | 01210 | 73 | 25253 |  |  |  |  |
| XM_032978225 | gjb2like | gjb-gen1.3 | NC_0461134 | 66 | 880388  879470  875118 | 880323  879205  874548 | R |  |  |  |  |  |  |  |
| XM_032978226 | gjb2like | gjb-gen1.1 | NC_046134 | 66 | 951990  955756  960267 | 952379  956059  960430 | F | 6523+  58479 | - |  | KE997332 | 2950F |  |  |
| XM_032979496 | gjb1like | gja-gen1.3 | NC_046141 | 73 | 1411653  1412950  1420567  1421112 | 1411781  1413126  1420936  1421583 | F |  |  |  | KE995112 | 2088F |  |  |
| XM_032981449 | gjb1like | gjb1/8.3 | NW_022638221 | - | 32851 | 33540 | F |  |  |  |  |  |  |  |
| XM_032945543 | gja3like | gja-gen1.2 | NW_022639386 | - | 37291  27585  18703  ? | 37134  27423  18533  ? | R |  |  |  |  |  |  |  |
| XM_032944750 | perhexa | gja9/10 | NW_022639020 | - | 101279  101960  103695  112006 | 101407  102073  103973  112363->? | F | 5439+  09011 | - |  |  |  |  |  |
| XM_032944354 | gjc1like | gjc-gen5.2 | NW_022638921 | - | 168581  170324  175048 | 168882  170788  175228 | F |  |  |  | KE995487 | 1073R |  |  |
| XM_032944365 | gjc1like | gjc-gen3.2 | NW_022638923 | - | 391302  390876  387720 | 390991  390390  387276 | R |  |  |  |  |  |  |  |
| XM_032945405 | gjc1like | gjc-gen5.3 | NW_022639276 | - | 12594  10842 | 12293  10314 | R |  |  |  |  |  |  |  |
|  |  | copy of above | NW_022639276 |  | 29783  28032 | 29482  27503 | R |  |  |  |  |  |  |  |
| XM_032945248 | gjc1like | gjc-gen5.1 | NW_022639200 | - | 27675  26417 | 27373  25937 | R |  |  |  | KE997621 | 4017R |  |  |
|  |  | copy of above | NW_022638943 |  | 143363  144622 | 143664  145102 | F |  |  |  |  |  |  |  |
|  |  | copy of above | NW_022638934 |  | 204985  203715 | 204684  203235 | R |  |  |  |  |  |  |  |

# Supplementary Table S8. Total connexin overview in the analyzed vertebrates.

Gene duplications in specific species are not included in the Table, but ohnologies in sterlet and teleosts are considered (marked by A and B, respectively).

| Group (cf Fig. 2)-> | | A | B | C | D | E | F | G | H | I | J | K | L | M | N |
| --- | --- | --- | --- | --- | --- | --- | --- | --- | --- | --- | --- | --- | --- | --- | --- |
| Gene name by “Greek” (left) or  "size" (right) nomenclatures | | M  a  m  m  a  l | M  a  r  s  u  p | M  o  n  o  t  r | B  i  r  d  s | C  r  o  c  o | T  u  r  t  l  e | L  i  z  a  r  d | A  m  p  h  i  b | L  a  t  i  m | P  o  l  y  p  t  e | A  c  i  p  e  n  s | H  -  o  s  t  e  i | T  e  l  e  o  s  t | C  a  r  t  i  l |
| ***gja1*** | 43 | x | x | x | x | x | x | x | x | x | x | A | x | B | x |
| ***gja2*** | 39.9 | - | - | - | x | - | x | x | x | x | x | A | x | B | x |
| ***gja3*** | 46, 48.5 | x | x | x | x | x | x | x | x | x | x | A | x | B | x |
| ***gja4*** | 37, 39.4 | x | x | x | x | x | x | x | x | x | x | A | x | B | x |
| ***gja5*** | 40,41.8 | x | x | x | x | x | x | x | x | x | x | A | x | B | x |
| ***gja6*** | 33 | x | - | - | - | - | - | - | - | - | - | - | - | - | - |
| ***gja8*** | 50,44.1 | x | x | x | x | x | x | x | x | x | x | A | x | B | x |
| ***gja9*** | 59,52.9 | x | - | x | x | x | x | x | x | x | x | A | x | B | x |
| ***gja10*** | 62,52.6 | x | x | - | x | x | x | x | x | x | x | x | x | B | x |
| ***gja11*** | 32.7,34.5 | - | - | - | - | - | - | - | - | x | x | x | x | x | x |
| ***gja12*** | 28.1,28.9 | - | - | - | - | - | - | - | - | - | - | - | - | x | - |
| ***gja13*** | 32.2,32.3 | - | - | - | - | - | - | - | - | - | - | - | - | x | - |
| ***gja14*** |  | - | - | - | - | - | - | - | - | x | - | A | x | - | - |
| ***gjb1*** | 32, 27.5 | x | x | x | x | x | x | x | x | x | x | A | x | B | x |
| ***gjb2*** | 26 | x | x | x | x | x | x | x | - | - | - | - | - | - | - |
| ***gjb3*** | 31,35.4 | x | x | x | x | x | x | x | x | x | x | A | x | B | x |
| ***gjb4*** | 30.3 | x | x | x | - | x | x | x | x1 | x | - | - | - | - | - |
| ***gjb5*** | 31.1 | x | x | x | x | x | x | x | x1 | - | - | - | - | - | - |
| ***gjb6*** | 30 | x | x | x | x | x | x | x | - | - | - | - | - | - | - |
| ***gjb7*** | 25,28.8 | x | x | x | x | x | x | x | x | x | x | A | x | x | x |
| ***gjb8*** | 30.3 | - | - | - | - | - | - | - | x | x | x | A | x | B | x |
| ***gjb9*** | 28.6,30.9 | - | - | - | - | - | - | - | - | - | x | A | x | B | x1 |
| ***gjb10*** | 34.4 | - | - | - | - | - | - | - | - | x1 | x | A | x | B | x1 |
| ***gjb11*** |  | - | - | - | - | - | - | - | - | - | - | - | - | - | x |
| ***gjc1*** | 45 | x | x | x | x | x | x | x | x | x | x | A | x | B | x |
| ***gjc2*** | 47,47.1 | x | x | x | x | x | x | x | x | x | x | A | x | x | x |
| ***gjc3*** | 31.3 | x | x2 | - | - | - | - | - | - | - | - | - | - | - | - |
| ***gjc4*** | 43.4 | - | - | - | x | x | x | - | x | x | x | A | x | B | x |
| ***gjd1*** |  | - | - | x3 | x | - | x | x | x | x | x | x | x | B | x |
| ***gjd2*** | 36,35 | x | x | - | x | x | x | x | x | x | x | A | x | B | x |
| ***gjd3*** | 31.9 | x | x | x | x | x | x | x | x | x | x | A | x | x | x |
| ***gjd4*** | 40.1 | x | x | x | x | x | x | x | x | x | x | A | x | x | x |
| ***gjd5*** | 39.2 | x | x | - | - | - | - | - | x | x | x | A | x | x | x |
| ***gjd6*** | 36.7 | - | - | - | - | - | - | - | - | - | x | A | x | x | - |
| ***gje1*** | 23 | x | x | x | x | x | x | x | x | x | x | A | x | B | x |

A. Ohnologous pairs exist in sterlet. In some cases (*gja3*, *gjb7*, *gjb8*, *gjc1*), more than two genes are found in the group, but it is not known whether this is a genetic reality or due to erroneous genome assembly.

B. Ohnologous pairs exist in one or another teleost (here we consider zebrafish, Atlantic herring and Japanese eel). Note that the exact number of ohnologous pairs may vary between species.

1. See text (Section 2.6).

2. There are two *GJC3* genes in opossum located on the same chromosome.

3. There are two *GJD1*-like genes in Platypus located on different chromosomes.
